# A new blood based epigenetic age predictor for adolescents and young adults

**DOI:** 10.1038/s41598-023-29381-7

**Published:** 2023-02-09

**Authors:** Håvard Aanes, Øyvind Bleka, Pål Skage Dahlberg, Kristina Totland Carm, Terho Lehtimäki, Olli Raitakari, Mika Kähönen, Mikko Hurme, Veslemøy Rolseth

**Affiliations:** 1 Division of Laboratory Medicine, Department of Forensic Sciences, Oslo University Hospital, Nydalen, P.O. Box 4950, 0424 Oslo, Norway; 2 Department of Clinical Chemistry, Fimlab Laboratories and Finnish Cardiovascular Research Center-Tampere, Faculty of Medicine and Health Technology, Tampere University, Tampere, Finland; 3 Centre for Population Health Research, University of Turku and Turku University Hospital, Turku, Finland; 4 Research Centre of Applied and Preventive Cardiovascular Medicine, University of Turku, Turku, Finland; 5 Department of Clinical Physiology and Nuclear Medicine, Turku University Hospital, Turku, Finland; 6 Department of Clinical Physiology, Faculty of Medicine and Health Technology, Tampere University Hospital and Finnish Cardiovascular Research Center-Tampere, Tampere University, Tampere, Finland; 7 Faculty of Medicine and Health Technology, Tampere University, Tampere, Finland; 8 Tampere University Hospital, Tampere, Finland

**Keywords:** DNA methylation, Statistical methods, DNA methylation, Ageing

## Abstract

Children have special rights for protection compared to adults in our society. However, more than 1/4 of children globally have no documentation of their date of birth. Hence, there is a pressing need to develop biological methods for chronological age prediction, robust to differences in genetics, psychosocial events and physical living conditions. At present, DNA methylation is the most promising biological biomarker applied for age assessment. The human genome contains around 28 million DNA methylation sites, many of which change with age. Several epigenetic clocks accurately predict chronological age using methylation levels at age associated GpG-sites. However, variation in DNA methylation increases with age, and there is no epigenetic clock specifically designed for adolescents and young adults. Here we present a novel age Predictor for Adolescents and Young Adults (PAYA), using 267 CpG methylation sites to assess the chronological age of adolescents and young adults. We compared different preprocessing approaches and investigated the effect on prediction performance of the epigenetic clock. We evaluated performance using an independent validation data set consisting of 18-year-old individuals, where we obtained a median absolute deviation of just below 0.7 years. This tool may be helpful in age assessment of adolescents and young adults. However, there is a need to investigate the robustness of the age predictor across geographical and disease populations as well as environmental effects.

## Introduction

Children are protected by a special subset of the human rights. However, as around 25% of the world’s children do not hold a birth certificate^1^ and therefore cannot document when they were born, age assessment is of great importance to secure children their human rights. Today, the most commonly applied age assessment methods in children and young adults include radiographs of teeth and skeleton, however, these are methods with large biological variation. In the recent decades, epigenetic clocks have emerged as a promising tool to predict both biological and chronological age.

Epigenetic age predictors are utilized to study both biological aging and in forensics. Environmental and psychological stressors affect epigenetic patterns^2^, and the development of epigenetic age predictors assessing biological age due to environmental influences or health issues are a growing field within aging research^3–5^. These predictors can, among other applications, be used to measure the effects of anti-aging interventions^6^. In contrast, in forensic applications, there is a need for predictors that are not affected by genetics, medical conditions or environmental variables (e.g. diet). Hence, epigenetic age predictors can be classified into two categories; one for forensic age assessment (chronological age), and another for health measurements (biological age). Forensic age estimation is of importance in a number of cases like unidentified bodies, suspects of crime, human trafficking, and in age assessment of asylum seekers with unknown age. These individuals may have been under immense physiological stress, and are likely to have experienced traumatic events during their lifetime. They may have also experienced starvation and/or malnutrition. It is plausible that these stressors may influence the epigenetic markers in epigenetic clocks.

Epigenetics is the biology of genetic control without change of the genetic code. Often, chemical molecules are attached to the DNA to alter the way the DNA is read by specific intracellular proteins. Methylation, the attachment of methyl groups to specific sites, primarily cytosines followed by guanines are one of the most studied epigenetic mechanisms. These sites are referred to as CpG dinucleotides, and several repetitive CpGs are defined as a CpG island, often occurring near, or in the promoter region of genes. Among other functions, DNA methylation regulates transcription, most commonly inhibiting gene expression^7^.

DNA methylation plays an important role in gene regulation during development and aging, and consequently many sites are associated with age^8^. Numerous age predictors have been developed to estimate chronological age based on DNA methylation patterns^9^. These epigenetic clocks accurately predict chronological age, but are mainly derived from datasets consisting of individuals with a broad age range (i.e. 0–100 years). Several studies have shown increased variation in age associated methylation CpGs and predicted epigenetic age as chronological age increases^10,11^. Therefore, it is expected that epigenetic age predictions will be more precise for younger individuals.

As DNA methylation patterns change more rapidly in children and adolescents, the development of pediatric epigenetic clocks has been a recent focus, resulting in three available age predictors designed for a pediatric target group^12–14^. As previously mentioned, children are protected by specific rights, and therefore the age group of around 18 years of age is important to investigate in several forensic applications. This age group has, to our knowledge, not yet been specifically targeted by any epigenetic clock. Epigenetic analyses might be both time-consuming and expensive, but can be reduced by decreasing number of CpG sites analysed. Therefore, age predictors developed for forensic use, are typically based on few CpG sites^15,16^. However, such predictors have lower accuracy than models with several hundred methylation sites, and better prediction models are warranted to assess if an individual is a child or an adult.

DNA methylation levels are commonly analysed using Illumina DNA methylation arrays. When it was introduced in 2007, the Illumina array covered 27,000 CpG sites, which was later upgraded to 450,000 sites in 2010 (450 K), and again to 850,000 sites in the latest EPIC array (2015). These arrays have high accuracy and precision^17^.

The analysis of DNA methylation microarrays is complex and there are numerous methods available aiming to improve data quality. These methods can be broadly divided into preprocessing, data normalization and batch correction, and each one can influence the downstream results^18^. The first step, preprocessing, includes image processing, calculation of methylation levels, quality control and filtering of probes and chips. The second step, normalization, intends to remove technical variation between and/or within chips. Normal-exponential out-of-band (Noob) is a commonly used method for background correction, which also includes dye bias correction^19^. Finally, batch effects; e.g. samples analysed on different days, position on the chip or efficiency of the bisulphite conversion, may systematically differ. ComBat^20^ is a widely conducted batch correction method. However as for most batch correction methods, warnings of its use leading to masking of biological differences are frequent^21,22^.

Machine learning methods are commonly applied to make age predictors using DNA methylation data. By far, the most popular method is elastic net (EN)^23^. EN is a regularized regression method that simultaneously selects the best set of methylation sites, and shrinks the coefficients by applying a penalty parameter. During the training of the model, the dependent variable is age, and the methylation sites are the independent variables. The trained model is then used to predict the age of new individuals. Predictive models are prone to overfitting, that is, they are parameterized well to the training data, but can perform poorly when predicting new data. This is due to variation in the training data included in the model, but not truly related to the outcome (e.g. age in our case)^24^. However, the result of the EN analysis is a small number of sites (relative to what you start with) where overfitting is reduced to some extent, and enhanced prediction performance for new data is achieved.

In the present study, we have developed the PAYA age predictor; a blood-based age predictor for adolescents and young adults between 12 and 25 years old, using DNA methylation levels at 267 CpG sites, aimed to assess chronological age without interference from environmental and disease conditions. This predictor might be included as part of a data driven process for assessing the chronological age of individuals in this age group.

## Methods

### Study participants

We identified relevant studies and downloaded data from GEO (Supplementary file 1—Supplementary Table S1). Inclusion criteria were studies using 450 K array data, generated from blood samples of individuals in the age range 10–60 years. Information on age and access to IDAT files (raw data) were also necessary. In addition, we included 450 K array data from the Young Finns study (YFS). The total training dataset consisted of 2316 samples from 1013 males and 1303 females. To test the model, we used 450 K array data from 920 18-year old individuals from the E-risk study^25^, hereby termed the test dataset. This is a birth cohort of twins born in 1994–5 in the United Kingdom. We randomly included one twin from each pair in our test group, in total 454 males and 466 females.

### Methylation analysis pipeline

DNA methylation data (IDAT files) was imported into R with the “read.metharray.exp” command in the Bioconductor package Minfi^26^. The methylation levels were calculated as beta-values: Methylated/(Methylated + Unmethylated + 100). During import we estimated cell counts using the “estimateCellCounts” function in Minfi^27^ and normalised data using “preprocessNoob” (includes background correction and dye-bias correction).

**Figure 1:**
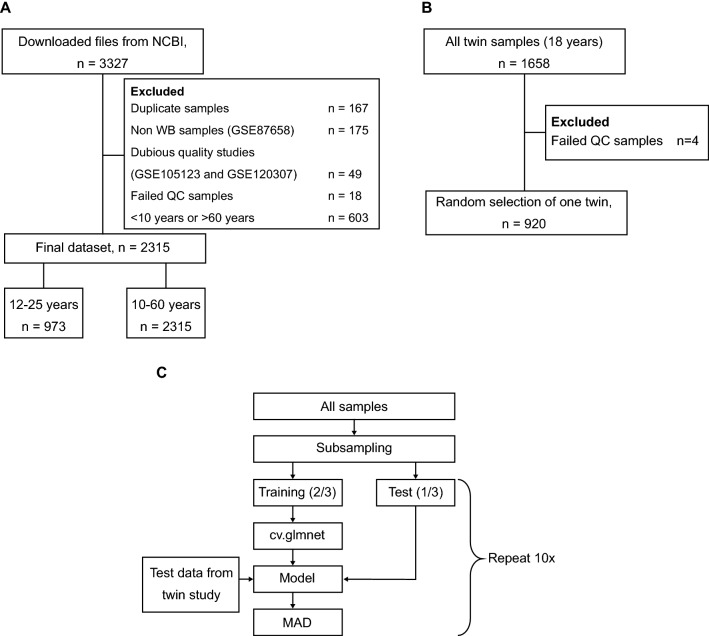
Sample retrieval and filtering. (**A**) Selection and quality control of the training data. (**B**) Selection and quality control of the independent test dataset. (**C**) The performance evaluation procedure. Using the training data, we selected 2/3 of the samples to fit an age prediction model using elastic net and the cross validation method. The remaining 1/3 of the samples were used to test the data, and the test dataset was also tested using the generated model. As a measure of prediction performance we used the median absolute deviation (MAD) in both cases.

We designed a rigorous quality control pipeline to detect samples of low quality (see Supplementary file 1—Supplementary methods), and samples were removed based on manual inspection of the quality control summary (see Supplementary file 1 for an overview of excluded sites and reason for exclusion). Based on this quality control, two studies were left out (Supplementary file 1—Supplementary methods). The final training dataset consisted of 2316 samples (age span 10–60 years old, 1013 males and 1303 females see Fig. 1a and Supplementary figure S1 for details). From the test dataset, we excluded eight samples due to poor quality, and included only one randomly selected individual from each twin pair (Fig. 1b). Probes associated with SNPs, and sites reported to have cross-reactivity, as well as probes not found in the newer Infinium EPIC chip, were removed (See https://github.com/sirselim/illumina450k_filtering for details). A total of 401,484 sites were removed after this filtering.

### Evaluation of prediction performance

To evaluate the impact of different processing pipelines we constructed a function in R to run elastic net cross validation repeatedly: We used the cv.glmnet function from GLMnet R-package (v4.1–3) with default loss metric, alpha = 0.5, and the minimum lambda value chosen (if not otherwise stated). The training data was sampled into a random training subset (2/3 of the samples), and used in the cross validation procedure, and a test set (1/3 of the samples) were used to predict age. In addition, we evaluated the performance of the built model from the cross validation using the twin test dataset, as these data were not part of the cross-validation procedure (Fig. 1c). Hence, we had two test data in this function. As our measure of performance, we used the median absolute deviation (MAD) measure (years between predicted and chronological age). The training and prediction was repeated ten times to obtain a distribution of MAD values. In the final model fitting (i.e. the final predictor) all samples, except the independent twin test dataset, were used as training data.

### Ethical considerations

All methods in the present study were carried out in accordance with the relevant guidelines and regulations. The Young Finns study was approved by the ethical committee of the Hospital District of Southwest Finland on 20 June 2017 (ETMK:68/1801/2017) and Regional Ethics Committee of the Expert Responsibility area of Tampere University Hospital, Helsinki University Hospital Ethical Committee of Medicine, The Research Ethics Committee of the Northern Savo Hospital District and Ethics Committee of the Northern Ostrobothnia Hospital District. The study protocol of each study phase corresponded to the WHO proposal. All participants gave their written informed consent, and the studies were conducted in accordance with the Declaration of Helsinki. At prior follow-ups of the Young Finns Study, informed consent of every participant under the age of 18 was obtained from a parent and/or legal guardian. The remaining datasets used in the present study were collected from previously published sources (Accession numbers in Supplementary table S1), and an approval by an ethics committee in the use of these data was therefore not necessary. In all included studies, authors state that informed consent for each participant was obtained.

## Results

### Age span of the training data and age transformation

**Figure 2:**
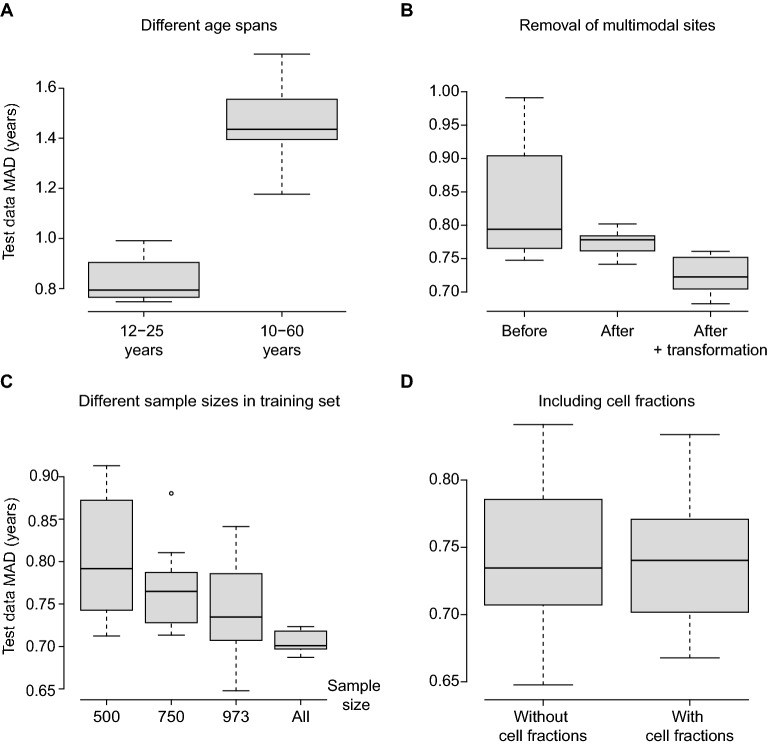
Impact of different methodological factors on the performance of the model. (**A**) Impact of using different age spans. (**B**) Removal of multimodal sites. (**C**) Impact of different sample sizes. (**D**) Including cell fractions in the model.

We tested the impact of building models with a narrow (12–25 years, n = 973 samples) or broad age span (10–60 years, n = 2316 samples). There was a distinct performance difference between the two approaches, where using the more narrow dataset improved performance (Fig. 2a), despite having less than half the number of samples. The age predictor trained on a wider age span was more accurate when we added “Horvath’s transformation”^8^ (described in Supplementary methods and shown in Supplementary Fig. S2a), while the 12–25 years predictions were not substantially affected (Supplementary Fig. S2b). Nonetheless, we chose to use the transformation for further analysis. The intended use of this model is in an adolescent population; we therefore chose to use the approach trained on the narrow age span.

### Removal of multimodal sites

We observed that some DNA methylation sites were included in the models despite not correlating with age, but instead having the appearance of outliers (Supplementary file 2). Consequently, we made a function to detect multimodal sites, and subsequently removed them (implemented in normtools, see Supplementary methods in Supplementary file 1). This reduced the number of sites to 393,821. While this intervention did not influence the median of the MAD much, it did reduce the variability considerably (Fig. 2b), and we decided to use this filtered dataset for further explorations.

### Sample size

The number of samples have been shown to affect the performance of age prediction models^28,29^. Down-sampling of our dataset to 500 and 750 randomly chosen samples, led to a gradual reduction in performance (Fig. 2c). We also measured performance without subsampling, i.e. using all samples for each model building. This resulted in further improvement of performance, with MAD around 0.70 years (min–max 0.69–0.72).

### Using cell counts in the predictions

It has been suggested that cell type composition might be an important variable in explaining DNA methylation patterns^30^. To test this we compared model building with and without predicted cell type fractions^31^. There were no improvement of age predictions for the test dataset when including cell type information, and therefore no cell type fractions were included in the model (Fig. 2d).

### Batch correction

**Figure 3:**
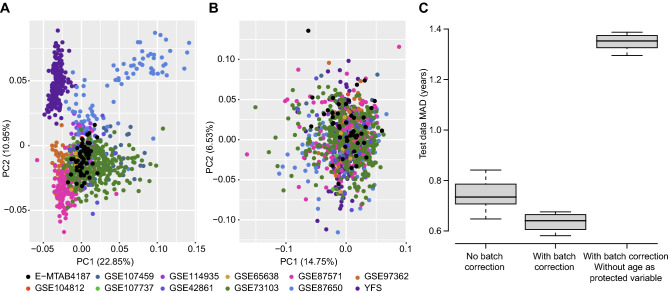
Identification and removal of batch effects. (**A**) Principal component analysis prior to batch effect removal. (**B**) Principal component analysis after batch effect removal. (**C**) Prediction performance in the test dataset before and after batch effect removal, and without age as a “protected variable”.

We applied principal component analysis (PCA) to uncover possible batch effects in our included data. PCA showed clustering of studies, indicative of batch effects (Fig. 3a). To overcome these batch effects, we applied ComBat^20,32^. We first used the default method (parametric version i.e. all studies become relative to each-other), and included age as a protected variable. This resulted in large differences between the training- and the independent test dataset (data not shown). However, after switching to “reference study mode” (adjusting training- and test data using the YFS study), the two datasets became comparable. After batch correction, the training data did no longer cluster by study (Fig. 3b), and predictions improved for the test data, compared to using no batch corrections (Fig. 3c). However, in a forensic application, the age of individuals tested will be unknown, hence we cannot use the chronological age as a “protected variable”. Without this, the use of batch correction did not improve predictions (Fig. 3c), on the contrary, the performance deteriorated considerably.

To by-pass the issue of not knowing chronological age, we instead tried to use the predicted age from the built model as the “protected variable” in ComBat. However, this strategy did not outperform using Noob normalization only (data not shown).

### Building the final model and marginal inspections

After testing various preprocessing and normalisation options (see methods and Supplementary Fig. S3), we opted for the following approach to build PAYA; we normalised data with Noob (dye bias correction included), before we removed samples and probes of low quality. We then removed CpG sites with multinomial modes and transformed the age variable using Horvath’s transformation of age (described in Supplementary methods). We trained the model on a narrow age span, according to the intended application of the method.

**Figure 4:**
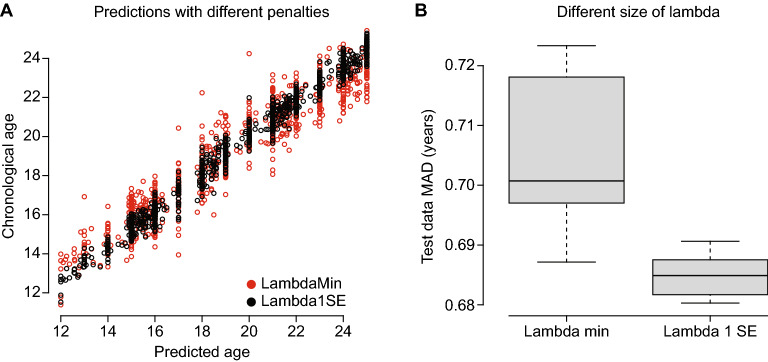
Choosing lambda. (**A**) Scatter plot of predicted and chronological age using different penalties (minimum lambda in black, one standard deviation lambda in red). (**B**) Prediction performance on independent test dataset using the same lambdas.

In EN, cross validation is used to calibrate the lambda penalty values. The lambda is typically chosen such that it returns the smallest cross validation error (i.e. “lambda.min”). When considering only the training data, smaller MAD were observed using “lambda.min” over “lambda.1se” (MAD 0.33 vs 0.73 years) (Fig. 4a). However, using “lambda.1se” returned slightly, but consistently smaller MAD for the test dataset (Fig. 4b): Also, we obtained using only 267 vs 660 CpG sites with the larger lambda.

Methylation level by age in the training dataset were plotted for all sites included in the model (Supplementary file 2). Correlations (Spearman) between degree of methylation and age, together with their associated coefficients, can be found in Supplementary file 3. Despite removing the multimodal sites, we observe some sites included in the final model that appear multimodal (e.g. cg21015022). In addition, we find sites that do not correlate well with age (e.g. cg03846689), and contained outlier values.

All sites in the age predictor were annotated with associated gene names (Supplementary file 4). Functional annotation and disease association analysis in Metascape^33^ was conducted. The analysis revealed no enrichment in the functional annotation categories, however, some disease associated terms were enriched (mental disorders, gait abnormality and smoking), with borderline significant q-values (Supplementary file 5).

We observed that most predictions were within + /− 1 year (of 18.5) (600/920, 65%), but there were also some predictions deviating > 2 years (45, ~ 5%), and five observations deviating > 3 years. Of those deviating > 3 years, three were overestimated (predicted ages 21.6, 22.1 and 22.2) and two underestimated (15.4 and 15.5), with the most severe error deviating 3.7 years. See Supplementary Fig. S4 for histogram of the observed errors. We found no effect of gender on the age-prediction. Using our test data (18 year olds), mean age prediction was 18.2 years for both genders.

### Comparison with other age predictors and studies

We compared the sites in our model with sites in other age predictor models utilizing DNA methylation (see Table 1 for details). Most overlap was observed between the Zhang epigenetic clock^29^ and the cABEC clock^28^, with 47 and 41 sites overlapping with our model, respectively (Table 1). Horvath’s “skin and blood predictor” shared 26 sites with our predictor. Little overlap was found with other blood based paediatric age predictors^13,14^. As expected, little overlap with the telomere age predictor was recognised^34^.

**Table 1:** Overview of epigenetic age predictors and the overlap in CpG sites compared to PAYA (Predictor of Adolescents and Young Adults).

Epigenetic clock	Publication	Number of CpG sites	Age span	Training, number of samples	Tissue	Overlapping CpG sites with PAYA	%
PAYA	Aanes et al., 2023	267	12–25	2315	Blood	–	–
Horvath	Horvath et al., 2013^8^	353	0–100	3931	27 different	4	1.5
Hannum	Hannum et al., 2013^37^	71	19–101	656	Blood	14	5.2
PhenoAge	Levine et al., 2018^3^	513	18–100	926	Blood	6	2.2
PedBE	McEwen et al., 2020^12^	94	0–20	1032	Buccal	5	1.9
Skin and Blood	Horvath et al., 2018^36^	391	0–94	896	Blood, more*	26	9.7
DNAmTL	Lu et al., 2019^34^	140	21–100	2256	Blood	4	1.5
Wu	Wu et al., 2019^13^	111	0–18	716	Blood	1	0.4
Zhang	Zhang et al., 2019^29^	514	2–104	13,661	Blood, saliva	47	17.6
Li	Li et al., 2018^14^	83	6–17	90	Blood	2	0.7
cABEC	Lee et al., 2020^28^	1892	19–88	2227	Blood	41	15.4

*Human fibroblasts, keratinocytes, buccal cells, endothelial cells and skin.

Due to lack of information on the preprocessing steps leading up to beta values in other epigenetic age predictors, direct comparisons are difficult to conduct. However, we ran our test dataset (age 18) through different epigenetic clocks, using the R-package methylClock^35^. Results from four of these clocks (Horvath, Skin and Blood, Hannum and the Elastic Net clock by Zhang et al.^8,29,36,37^) showed that PAYA predicted chronological age with the highest precision and accuracy (Supplementary Fig. S5).

### Assessment of the impact of stress on sites in the age predictor

To evaluate if our age predictor could be affected by childhood trauma and possible physiological stress, we generated a DNA methylation site list from a review of DNA methylation effects from childhood trauma^38^ (Supplementary file 6). Only one CpG site from this list was part of our age predictor, cg07012999. This site has a small positive coefficient of 0.1, and therefore a marginal effect in the age prediction. Two *FKBP5* associated CpG sites, cg20813374 and cg00130530, linked to age and stress^39^, were not found in our model.

To our knowledge, no epigenome wide studies to investigate the impact of starvation and/or malnutrition at the epigenetic level for adolescents or young adults have been conducted. Two studies have, however, investigated locus specific effects on the *IGF2* gene^40,41^. This gene is not present in our cohort of genes associated with the sites of the age predictor.

## Discussion

In this study, we present a novel blood-based epigenetic age predictor, PAYA, based on data from adolescents and young adults between 12 and 25 years old. The predictor includes 267 CpG sites and showed a high degree of accuracy in prediction of chronological age in an independent test set of 18-year-old individuals with an estimate of MAD value below 0.7 years. This is noticeably better than targeted approaches with fewer CpG sites, which typically report MAD values between 5 and 9 years^42^.

Although comparisons with other epigenome-wide age predictors are difficult due to different data and normalization used for training, we find that PAYA perform better in the independent test data compared to other epigenetic clocks (Supplementary Fig. S5). We observed that reduction of the age span of the training data caused selection of other CpG sites, and increased prediction performance considerably compared to a wider age span (Fig. 2A). This is in concordance with previously reported results^12^.

As mentioned, the age range of the included samples might affect the included CpG sites in the predictor. To our surprise, we observed little overlap of CpG sites with other pediatric clocks. Pediatric clocks have been evaluated by others, and were outperformed by the Skin and Blood clock from Horvath et al^43^. In line with this, our model shares more sites with the Skin and Blood clock compared to two pediatric clocks (Table 1). We observe that PAYA assess age more accurate in the independent test population than the Skin and Blood clock (Supplementary Fig. S5), probably due to the targeted age span on which it is trained.

Our model is trained and tested using Infinium HumanMethylation450 BeadChip data, but since new data will originate from the EPIC arrays, we have selected only EPIC compatible CpG sites to build our model. Despite the much larger number of sites included in the EPIC-array, it does not appear that epigenetic age predictors derived from EPIC data achieve higher accuracy than predictors based on the 450 K array^28,44^.

It has been claimed by others that increasing the number of samples will make the model more accurate^28,29^. This was confirmed in our study, as down-sampling of our own data resulted in lower accuracy of the age predictor (Fig. 2C). Notably, we did not observe a horizontal asymptote of the MAD value when all data was included in the training. Hence, the accuracy of the present model might be improved if more IDAT files were available.

The two most important tunable parameters in the EN analysis are the alpha and lambda values. The former decides the amount of Lasso versus Ridge regression, while lambda is the amount of penalty used^45^. We did initial testing of alpha values, and opted for a value of 0.5, similar to most existing models^8,28,29,36^. Lambda was chosen using k-fold cross-validation (with k = 10), and prediction performance on the independent test dataset. Interestingly, we achieved better performance when we chose the largest lambda within one standard error of the minimum prediction error providing less included CpG sites (from > 600 to < 300). We validated the trained age predictor utilizing 920 individuals (18 year olds), from an independent dataset. It is crucial to test the age predictors on an independent dataset since there can be study-specific factors that are explained by the model, but not present in independent data^46^. We observed similar performance between the test data and the independent test dataset, indicating that overfitting has been avoided.

It has been suggested that environmental effects are particularly important in epigenetic variation^47^. This confounding variable, as well as a number of diseases and conditions remains an important area for future research^47,48^. Recently, Mayer and colleagues revealed dependency between epigenetic age markers and growth disorders^49^. The intended use of PAYA is to predict chronological age not influenced by genetic and environmental effects. We tried to overcome this to some degree by including studies from different parts of the world, and only excluded conditions that are readily observable, meaning that not only healthy individuals are part of the data. The sites identified by EN may therefore be independent of some genetic and environmental effects, because if they were not, they would perhaps not explain the observed variation in chronological age well. PAYA might therefore, to some extent capture such effects. However, to fully assess the robustness of the current age predictor across populations with different ethnicities and environmental conditions, we have initiated a study where we sample individuals from different regions of the world, and evenly over the age span between 12 and 25 years of age.

The data used to build our model was from blood samples, and therefore contain several different cell types. The cell type composition of the blood might influence the obtained DNA methylation profiles^50^. It has been suggested that the changes in cell type composition occurring with age, at least in part, may explain the age associated changes of DNA methylation^51^. However, we could not identify any effect by including cell type fractions in the age predictors (Fig. 2D). Other cell type prediction methods could perhaps yield better explanatory variables in the model^52^, but this remains to be tested. There is also a need to evaluate the actual composition of the blood and not only the predicted composition.

Despite our initial tests of the age predictor, a more thorough evaluation of performance is needed, with a full panel of performance metrics^53^. Application of the age predictor in forensic work will require thorough quality control (Ref Supplementary file 1) of new samples. It will also be of interest to test the PAYA age predictor in populations outside the intended age span to investigate the accuracy outside the age range of training.

To conclude, through thorough evaluation and selection of different analytical options, we have developed PAYA, the first blood based age predictor developed specifically for adolescents and young adults. We observe that PAYA outperforms existing epigenetic age predictors, making it eligible for application in forensic age-assessment, either alone or in combination with existing radiographic methods. Future studies is required to reveal if PAYA is robust when applied to diverse populations with different ethnicities and environmental or genetic effects.

## Supplementary Information

Supplementary Information 1.

Supplementary Information 2.

Supplementary Information 3.

Supplementary Information 4.

Supplementary Information 5.

Supplementary Information 6.

Supplementary Information 7.

## Data Availability

The datasets used and/or analysed in this article are freely available datasets from each of the included studies (Supplementary table S1), except for the dataset obtained from the Cardiovascular Risk In Young Finns study (YFS) after submission and approval of our study plan by the YFS coordinators. The YFS dataset comprises health related participant data and their use is therefore restricted under the regulations on professional secrecy (Act on the Openness of Government Activities, 612/1999) and on sensitive personal data (Personal Data Act, 523/1999, implementing the EU data protection directive 95/46/EC). Due to these legal restrictions, the data from this study can not be stored in public repositories or otherwise made publicly available. However, data access may be permitted on a case by case basis upon request only. Data sharing outside the group is done in collaboration with YFS group and requires a data-sharing agreement. Investigators can submit an expression of interest to the chairman of the publication committee (Prof Mika Kähönen, Tampere University, Finland).
