# Supplementary material for: A new blood based epigenetic age predictor for adolescents and young adults
Source: Sci Rep. 2023 Feb 9;13:2303. doi: 10.1038/s41598-023-29381-7 (PMC9911637; doi:10.1038/s41598-023-29381-7)
Supplement: Supplementary file 1 — Supplementary Information 1. [file 41598_2023_29381_MOESM1_ESM.pdf]

# A new blood based epigenetic age predictor for adolescents and young adults

Aanes et al.

Supplementary file 1

# Supplementary Figures

## Figure S1

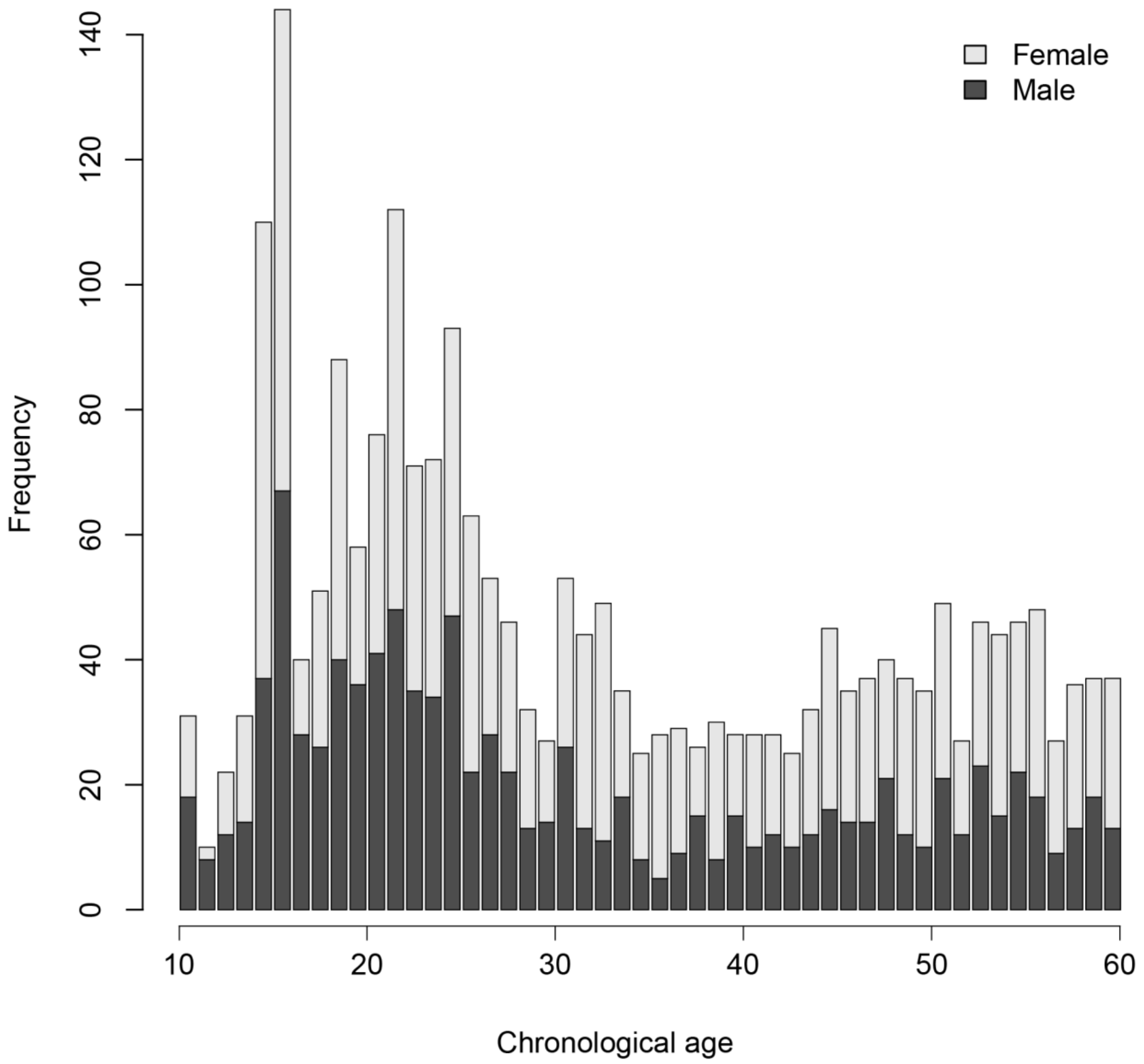


**Supplementary Figure S1.** Age distribution by gender in the test dataset (2316 samples).

Figure S2

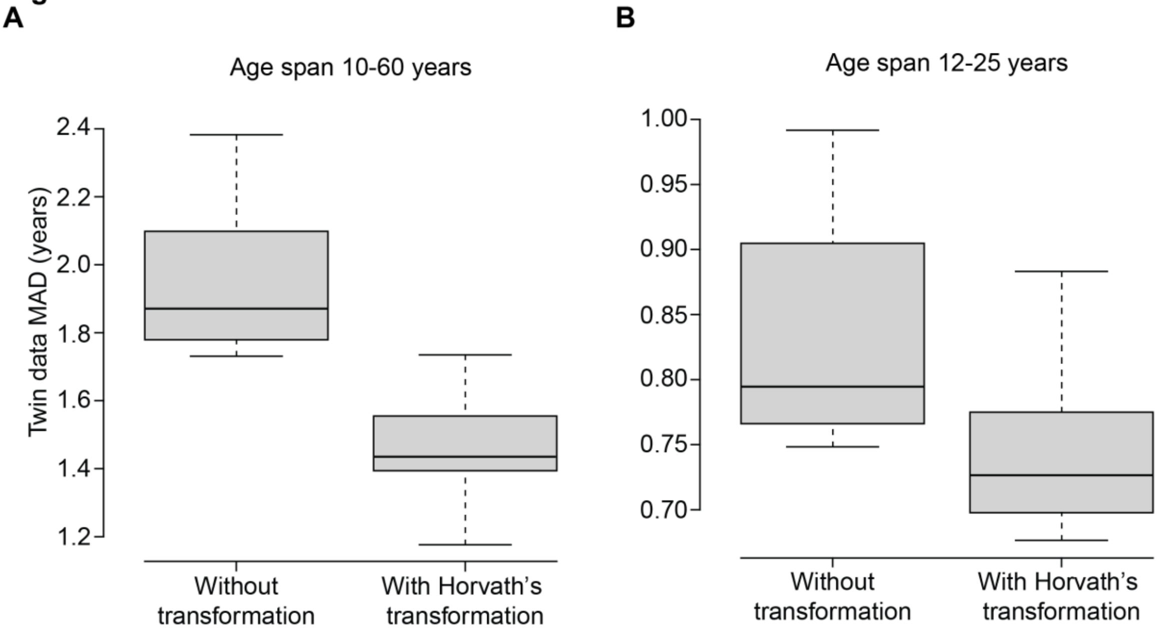


**Supplementary Figure S2. Impact of transformation in different age groups.** Impact of transformation of age on prediction performance A) when using samples with individuals between 10-60 years. B) when using samples with individuals between 12-25 years.

**Figure S3**

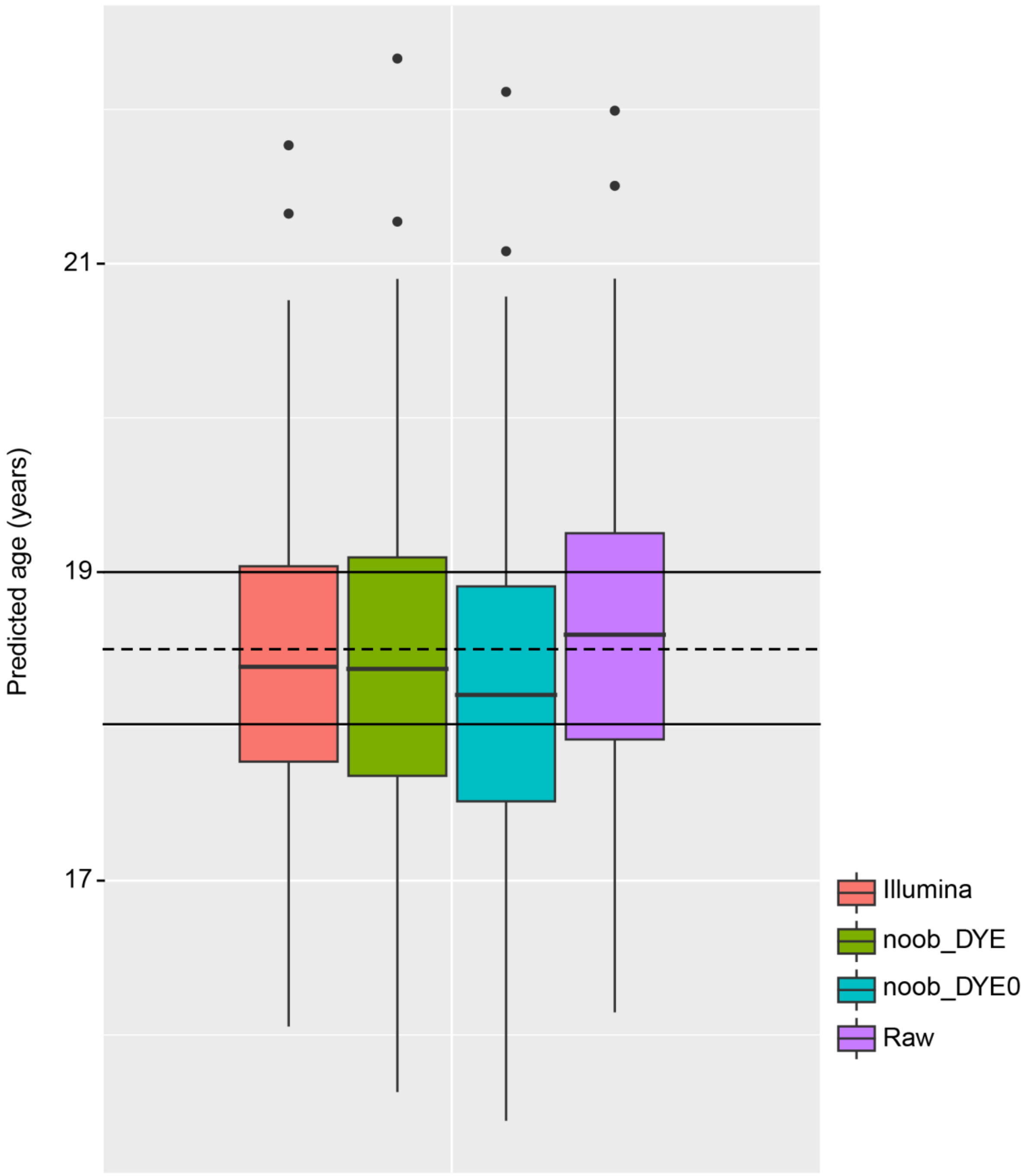


**Supplementary Figure S3.** Different normalisation methods and impact on prediction performance.

## Figure S4

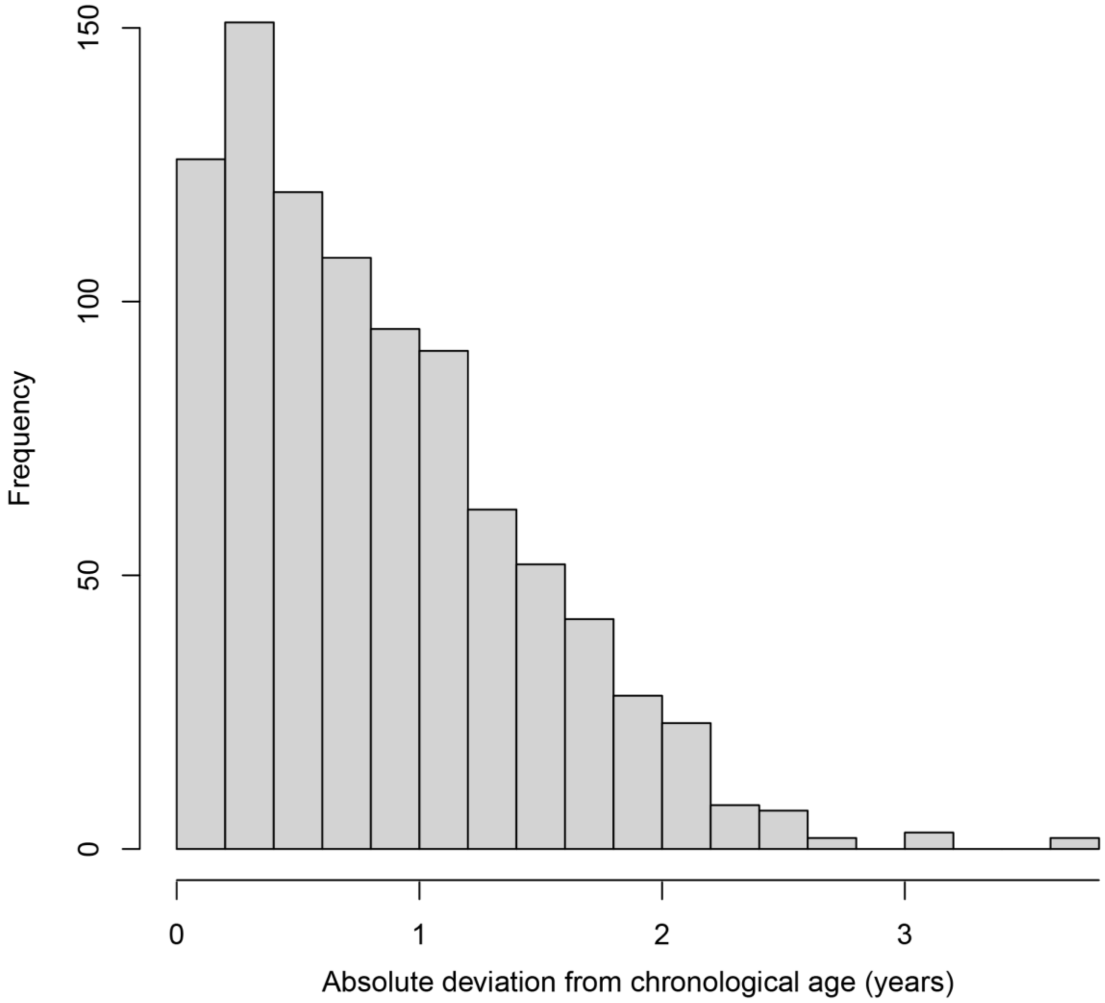


**Supplementary Figure S4.** Absolute deviation (in years) between predicted and chronological age using the final model on test dataset (n=920, 18 years).

Figure S5

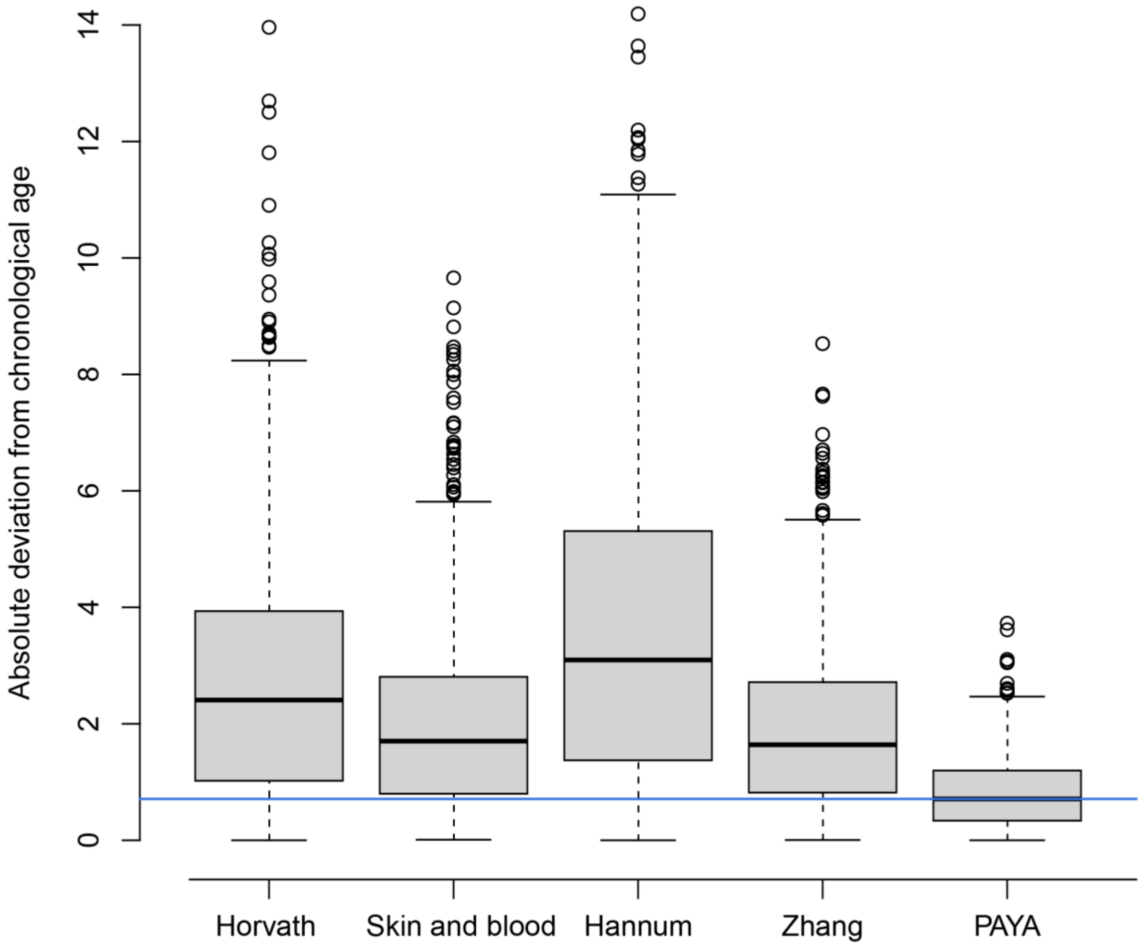


**Supplementary Figure S5.** Comparison of absolute deviations from chronological age between four existing epigenetic predictors and PAYA. Blue line indicates the median absolute deviation of PAYA.

## Supplementary Tables

**Supplementary Table S1.** Overview of studies and datasets included in the analysis.

| Dataset number | GEO Accession nr | Number of samples (male/female) | Study | Publication year | Country | Age range | Ethnicity |
|---|---|---|---|---|---|---|---|
| 1 | E-MTAB4187 | 51 (31/20) | *Almstrup et al Sci Rep [1]* | 2016 | Denmark | 12.2-16.4 | Danish/European |
| 2 | GSE104812 | 3 (2/1) | *Shi et al FSI Genetics [2]* | 2018 | China | 12.1-14.6 | Han-Chinese |
| 3 | GSE107459 | 44 (0/44) | *Knight et al Epigenetics [3]* | 2018 | USA | 18.2-25.0 | African American |
| 4 | GSE107737 | 18 (18/0) | *Fang et al Int J of Mol Med [4]* | 2019 | China | 18.0-25.0 | Chinese |
| 5 | GSE114935 | 13 (0/13) | *Knight et al Sci Rep [5]* | 2018 | USA | 19.0-25.0 | Mixed ethnicities (Caucasian, Hispanic, African American, Other) |
| 6 | GSE42861 | 19 (2/17) | *Liu et al Nat Biotechnol [6]* | 2013 | Sweden | 18.0-25.0 | Swedish |
| 7 | GSE65638 | 5 (0/5) | *Xu et al Sci Rep [7]* | 2015 | China | 21.0-25.0 | Han-Chinese |
| 8 | GSE73103 | 304 (168/136) | *Voisin et al Genome Med [8]* | 2015 | Sweden | 14.0-25.0 | Caucasian |
| 9 | GSE87571 | 163 (74/89) | *Johansson et al Plos One [9]* | 2013 | Sweden | 14.0-25.0 | Swedish |
| 10 | GSE87650 | 99 (56/43) | *Ventham et al Nat Commun [10]* | 2016 | UK | 17.0-25.0 | N/A |
| 11 | GSE97362 | 69 (47/22) | *Butcher et al Am J Hum Gen [11]* | 2017 | UK | 12.0-19.0 | N/A |
| 12 | YFS | 185 (113/72) | *Kananen et al Age [12]* | 2016 | Finland | 14.8-24.8 | Finnish |

# Supplementary Methods

## Horvath's transformation of age

Horvath's transformation of age is applied due to an observed logarithmic dependence between methylation and age up until adulthood, and a linear dependence later in life, as described by Horvath (2013) [13]. Hence, childhood ages (up until 20 years) are transformed with a logarithmic function, while ages over 20 years are transformed using a linear function;

Fage = function(x,adult.age=20) { x=(x+1)/(1+adult.age); y=ifelse(x<=1, log( x),x-1);y }

invFage = function(x,adult.age=20) { ifelse(x<0, (1+adult.age)*exp(x)-1, (1+adult.age)*x+adult.age) }

## Details about R-scripts for quality control pipeline

We have implemented a comprehensive quality control pipeline by including several R-functions into one R-script (see Supplementary file 7). These functions execute several quality control functions that are part of the following R-packages: ewastools, wateRmelon and sesame. The following table gives an overview of the functions.

**Supplementary Table S2.** Overview of R-functions included in the quality control pipeline.

| **R-function** | **Package** | **Updated R-function** | **Comments** |
|---|---|---|---|
| outlyx | wateRmelon | wm_outlyx | Efficient matrix scaling and avoid calling prcomp. ~(1.5 – 7)x speed |
| pwod | wateRmelon | wm_pwod | Utilizing fastR::rowSort for faster calculation of fivenum (rowFivenum). ~14x speed |
| detectionPnegEcdf OOBAH | sesame | sesame_detectionPecdf | oob or neg |
| detectionP | ewastools | ewastools_detectionP | oob |
| detectionP.neg/ ewastools | ewastools/minfi | ewastools_detectionP.neg | neg: User can choose ewastools or minfi approach |
| dasen | wateRmelon | wm_dasen | |
| plotQC | minfi | showQC | Quality Control |
| bscon_minfi | wateRmelon | wm_bscon | Quality Control |
| bisConversionControl | sesame | sesame_bisConversionControl | Quality Control |

In addition, we have included a function called *estNmodes* that estimates the number of modes in a site (efficient implementation for a large scale of sites).

**Supplementary Table S3.** Overview of the different functions used to flag whether a sample fails or not.

| # | Function call (ordered) | R-package | Details | Flag threshold |
|---|---|---|---|---|
| 1 | control_metrics | ewastools | Provides the 17 QC metrics (Illumina) | |
| 2 | sample_failure | ewastools | Returns flagged samples (same thresholds as in ewastools) | Illumina |
| 3 | wm_bscon | normtools | Obtain common bisulfitscore | Threshold=85 |
| 4 | sesame_bisConversionControl<br>getZscoreOutlier | normtools | Alternative bisulfit score<br>Obtaining Zscores | Zscore > 0.1/nsamples |
| 5 | showQC | normtools | | badSampleCutoff =10.5 |
| 6 | estNmodes<br>RemoveXY_SNPetc | several | **Site filtering** | |
| 7 | wm_outlyx | normtools | See [14] | wateRmelon default thresholds |
| 8 | getBetaDistrDev | normtools | Automated beta-distribution check: Calculates a deviation measure from median of all samples for given beta-values. Type I and Type II separately. | Thresholds >[0.08,0.6] [TypeI,TypeII] |
| 9 | checkBeadCounts | normtools | Obtain distribution of the number of failed sites (nbeads less than 3 used as criterion) | Zscore > 0.1/nsamples |
| 10 | ewastools_detectionP(oob) | normtools | Pvalue-calculations (method 1) | Failed if Pval>0.05 Sample with >5% of sites failing |
| 11 | ewastools_detectionP.neg | normtools | Pvalue-calculations (method 2) | |
| 12 | sesame_detectionPecdf(oob) | normtools | Pvalue-calculations (method 3) | |
| 13 | sesame_detectionPecdf(neg) | normtools | Pvalue-calculations (method 4) | |

## Considered quality control pipeline (QC)

We have implemented the wrapper R-function *doQC* for running the full QC pipeline that flags potentially poor samples. The function takes a “meth” object returned from ewastools::read_idats (ewastools) as input. The returned output is a list of failed samples due to specified flags. These were further investigated manually. Note: The current version only support 450k. We used the default function call.

## Removed samples

The evaluation of poor quality samples was based on several criterion, for instance the failed flags from quality control, or identified as a sample look up (wrong predicted age or sex).

**Supplementary Table S4.** Overview of samples excluded in the final model.

| GEO Accession | n | Removed sample | Comment |
|---|---|---|---|
| GSE65638 | 2 | GSM1602320<br>GSM1602321 | Indicated as 32 years but predicted as 7 years<br>- |
| GSE73103 | 2 | GSM1886364<br>GSM1886544 | Failed Pvalue-calculation checks<br>- |
| GSE87571 | 1 | GSM2334163 | Failed bisulfite control check (bscon) |
| GSE87650 | 2 | GSM2337570<br>GSM2337461<br>GSM2562710<br>GSM2562878<br>GSM2337253<br>GSM2337382 | Failing beta-distr, outlyx, bscon (also extra outliers in PCA)<br>-<br>High nbeads fails<br>-<br>Wrong predicted sex<br>- |
| GSE97362 | 4 | GSM2562804<br>GSM2562792<br>GSM2562929<br>GSM2562861 | Wrong age meta data?<br>-<br>Extreme outlier in age prediction<br>Wrong predicted sex |
| YFS | 1 | 9989536011 | Wrong predicted sex |
| GSE105018 (Twin) | | | Beta-distribution outliers |

## Removed datasets

Two datasets was excluded in the analysis leading up to the final model.

Dataset GSE105123 was excluded due to failed bisulphite conversion (BCII Illumina metric) for most samples. Further, many of the samples had numerous sites with nbeads<3 e.g. high number of failed beads. Interestingly, visual inspection showed that the methylation values overlapped poorly with other datasets for age related sites.

Dataset GSE120307 was excluded due to observed failed bisulphite conversion (discovered by wm_bscon) for almost all samples.

## Supplementary references


1. Almstrup, K., et al., *Pubertal development in healthy children is mirrored by DNA methylation patterns in peripheral blood.* Sci Rep, 2016. **6**: p. 28657.
2. Shi, L., et al., *DNA methylation markers in combination with skeletal and dental ages to improve age estimation in children.* Forensic Science International: Genetics, 2018. **33**: p. 1-9.
3. Knight, A.K., et al., *SLC9B1 methylation predicts fetal intolerance of labor.* Epigenetics, 2018. **13**(1): p. 33-39.
4. Fang, X., et al., *Genome-wide methylation study of whole blood cells DNA in men with congenital hypopituitarism disease.* Int J Mol Med, 2019. **43**(1): p. 155-166.
5. Knight, A.K., et al., *Association between one-carbon metabolism indices and DNA methylation status in maternal and cord blood.* Scientific Reports, 2018. **8**(1): p. 16873.
6. Liu, Y., et al., *Epigenome-wide association data implicate DNA methylation as an intermediary of genetic risk in rheumatoid arthritis.* Nat Biotechnol, 2013. **31**(2): p. 142-7.
7. Xu, C., et al., *A novel strategy for forensic age prediction by DNA methylation and support vector regression model.* Scientific Reports, 2015. **5**(1): p. 17788.
8. Voisin, S., et al., *Many obesity-associated SNPs strongly associate with DNA methylation changes at proximal promoters and enhancers.* Genome Med, 2015. **7**: p. 103.
9. Johansson, Å., S. Enroth, and U. Gyllensten, *Continuous Aging of the Human DNA Methylome Throughout the Human Lifespan.* PLOS ONE, 2013. **8**(6): p. e67378.
10. Ventham, N.T., et al., *Integrative epigenome-wide analysis demonstrates that DNA methylation may mediate genetic risk in inflammatory bowel disease.* Nature Communications, 2016. **7**(1): p. 13507.
11. Butcher, D.T., et al., *CHARGE and Kabuki Syndromes: Gene-Specific DNA Methylation Signatures Identify Epigenetic Mechanisms Linking These Clinically Overlapping Conditions.* Am J Hum Genet, 2017. **100**(5): p. 773-788.

12. Kananen, L., et al., *The trajectory of the blood DNA methylome ageing rate is largely set before adulthood: evidence from two longitudinal studies.* Age (Dordr), 2016. **38**(3): p. 65.
13. Horvath, S., *DNA methylation age of human tissues and cell types.* Genome Biol, 2013. **14**.
14. Gorrie-Stone, T.J., *DNA Methylation: Methods and Analyses*. 2019, University of Essex.
