# Supplementary material for: A new blood based epigenetic age predictor for adolescents and young adults
Source: Sci Rep. 2023 Feb 9;13:2303. doi: 10.1038/s41598-023-29381-7 (PMC9911637; doi:10.1038/s41598-023-29381-7)
Supplement: Supplementary file 2 — Supplementary Information 2. [file 41598_2023_29381_MOESM2_ESM.pdf]

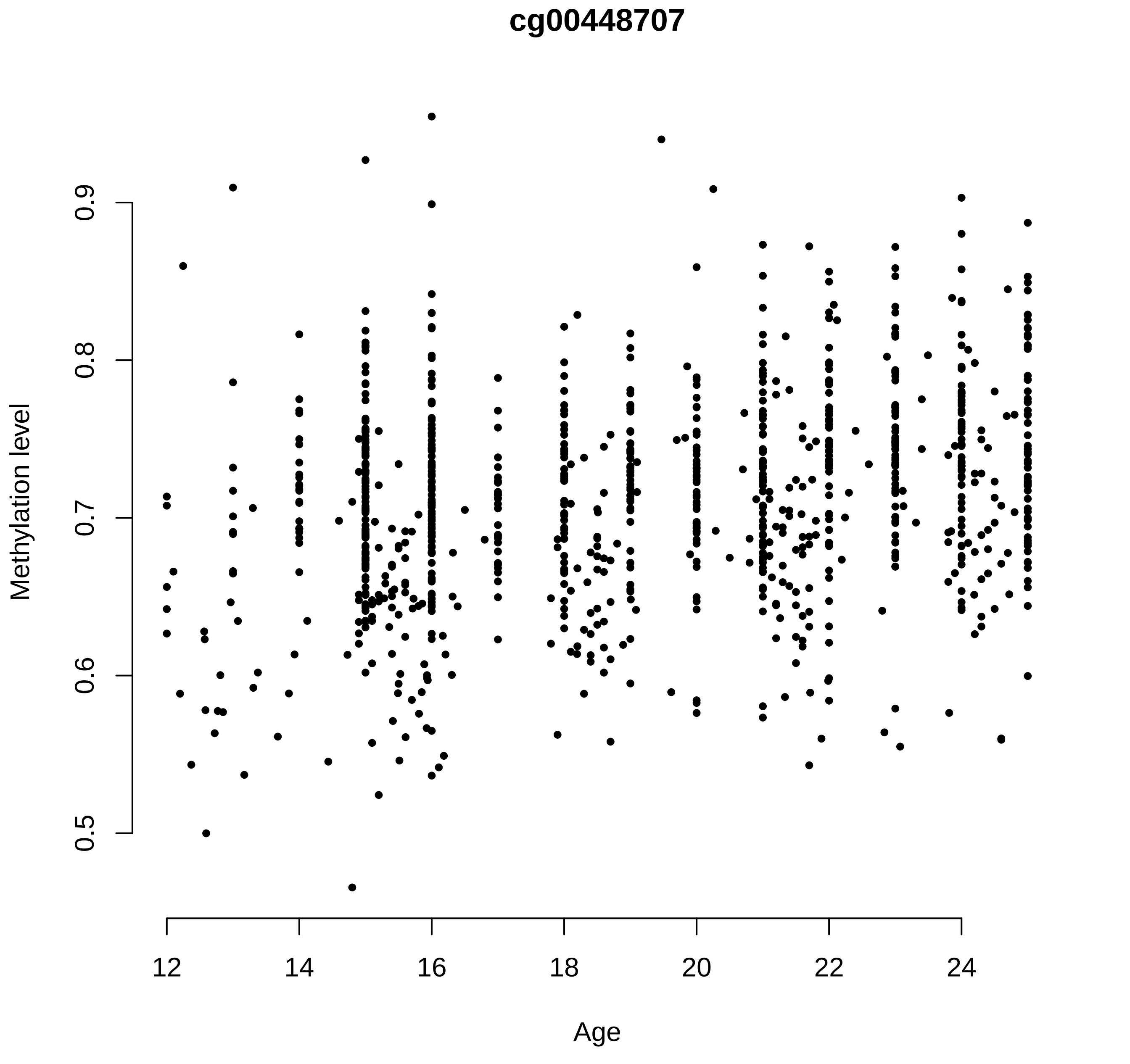
cg00448707
Methylation level
0.5
0.6
0.7
0.8
0.9
Age
12
14
16
18
20
22
24

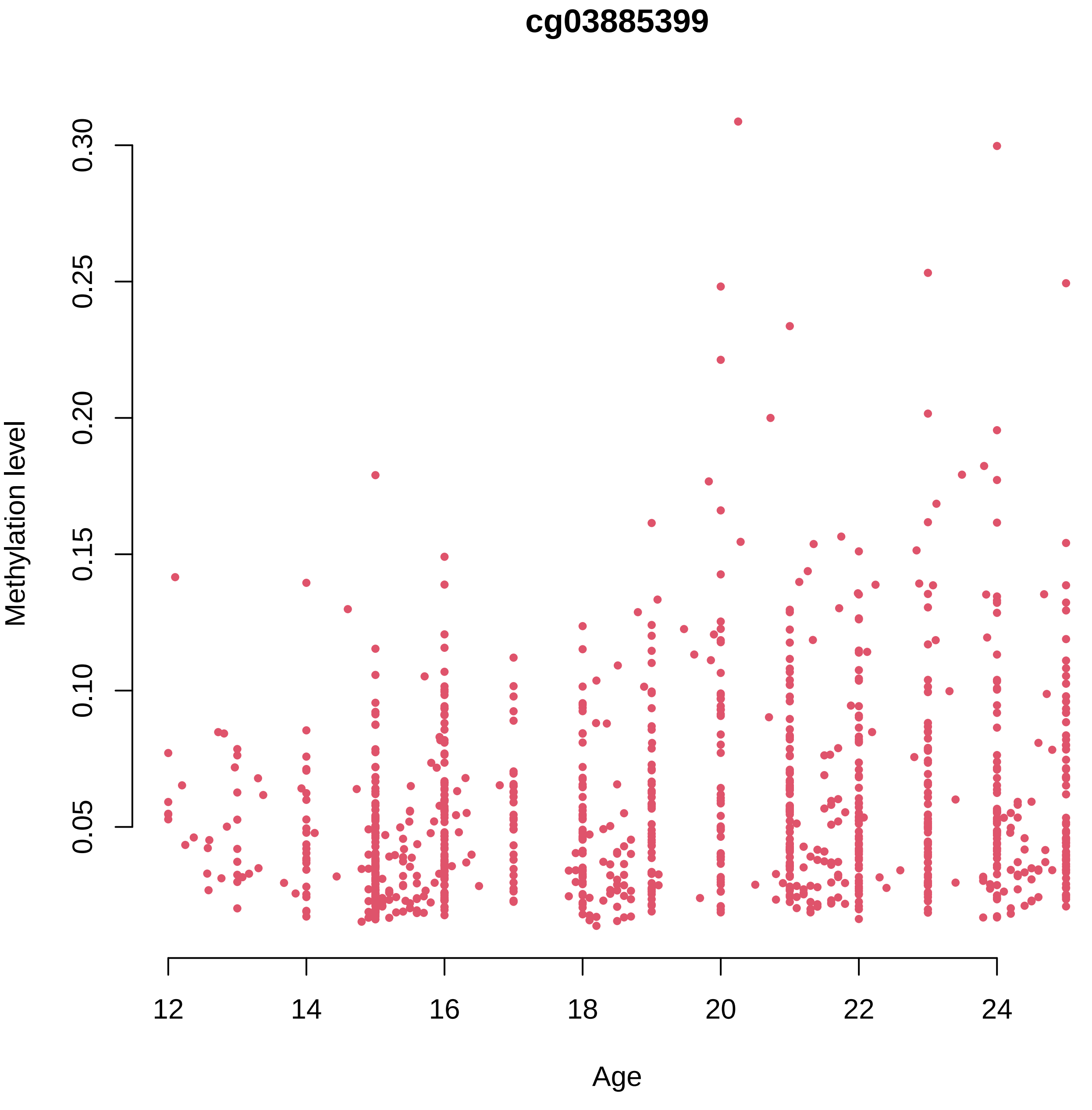
cg03885399
Methylation level
0.05
0.10
0.15
0.20
0.25
0.30
12
14
16
18
20
22
24
Age

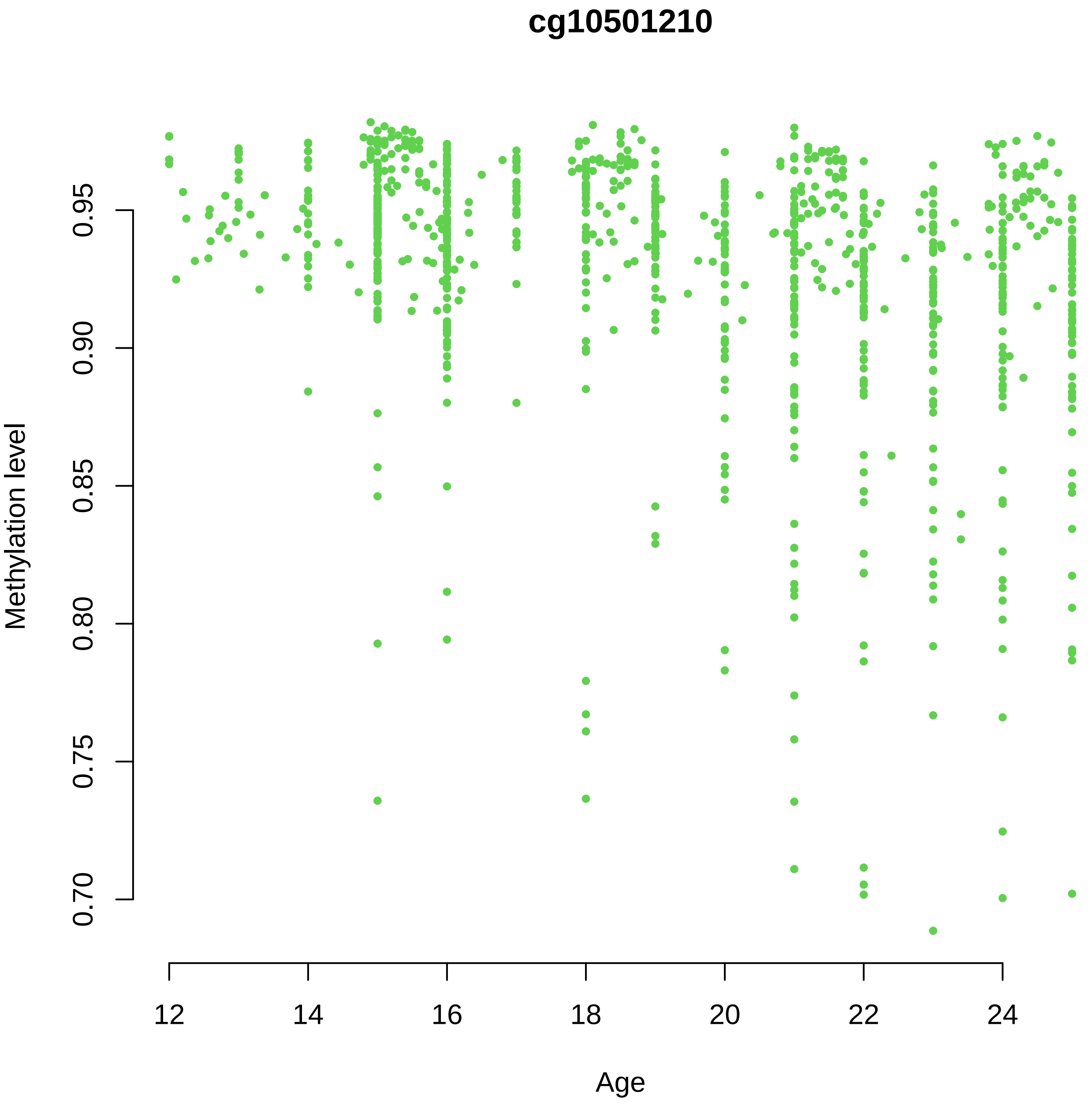

cg10501210
Methylation level
0.70
0.75
0.80
0.85
0.90
0.95
12
14
16
18
20
22
24
Age

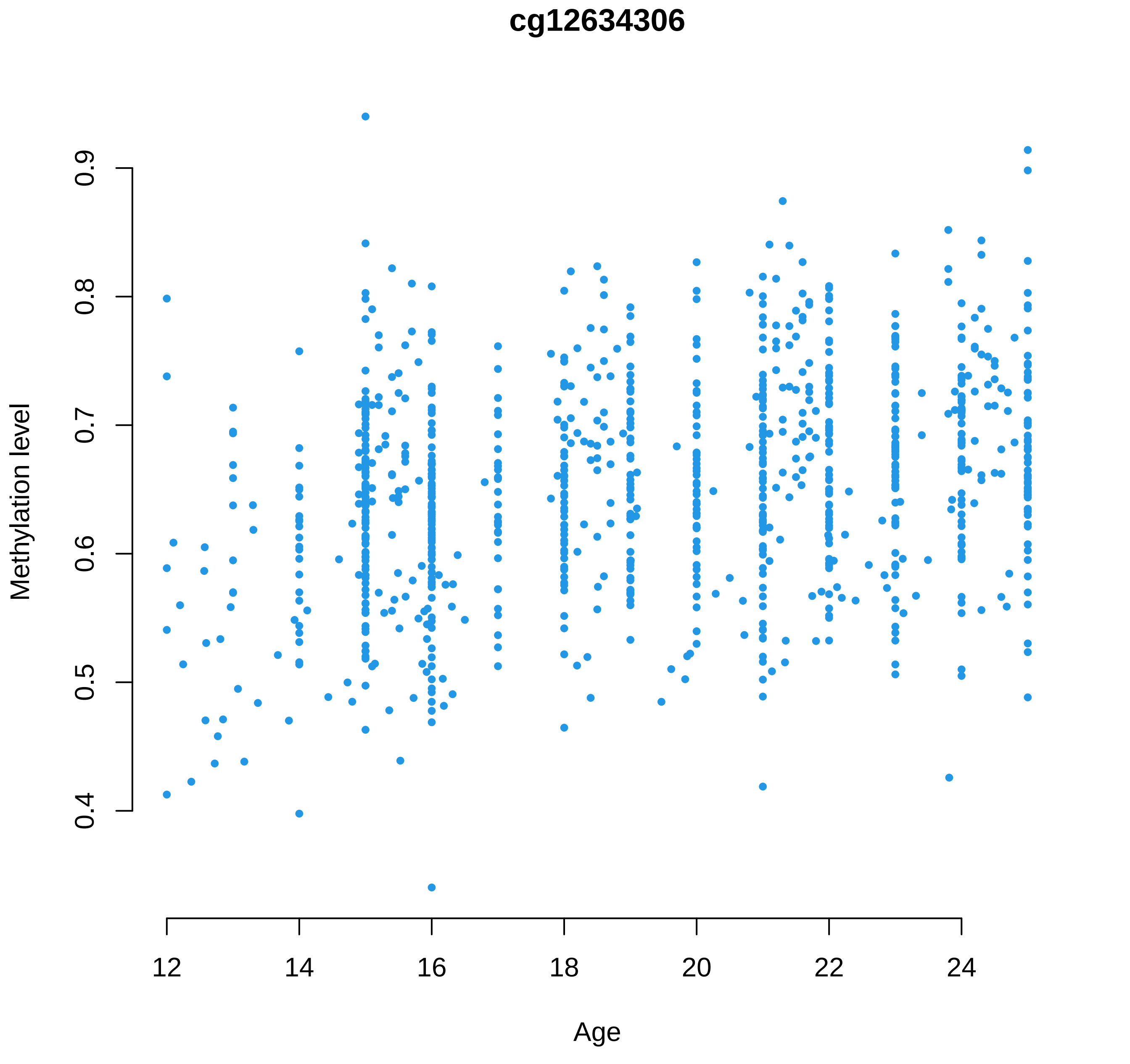

cg12634306
Methylation level
0.4
0.5
0.6
0.7
0.8
0.9
12
14
16
18
20
22
24
Age

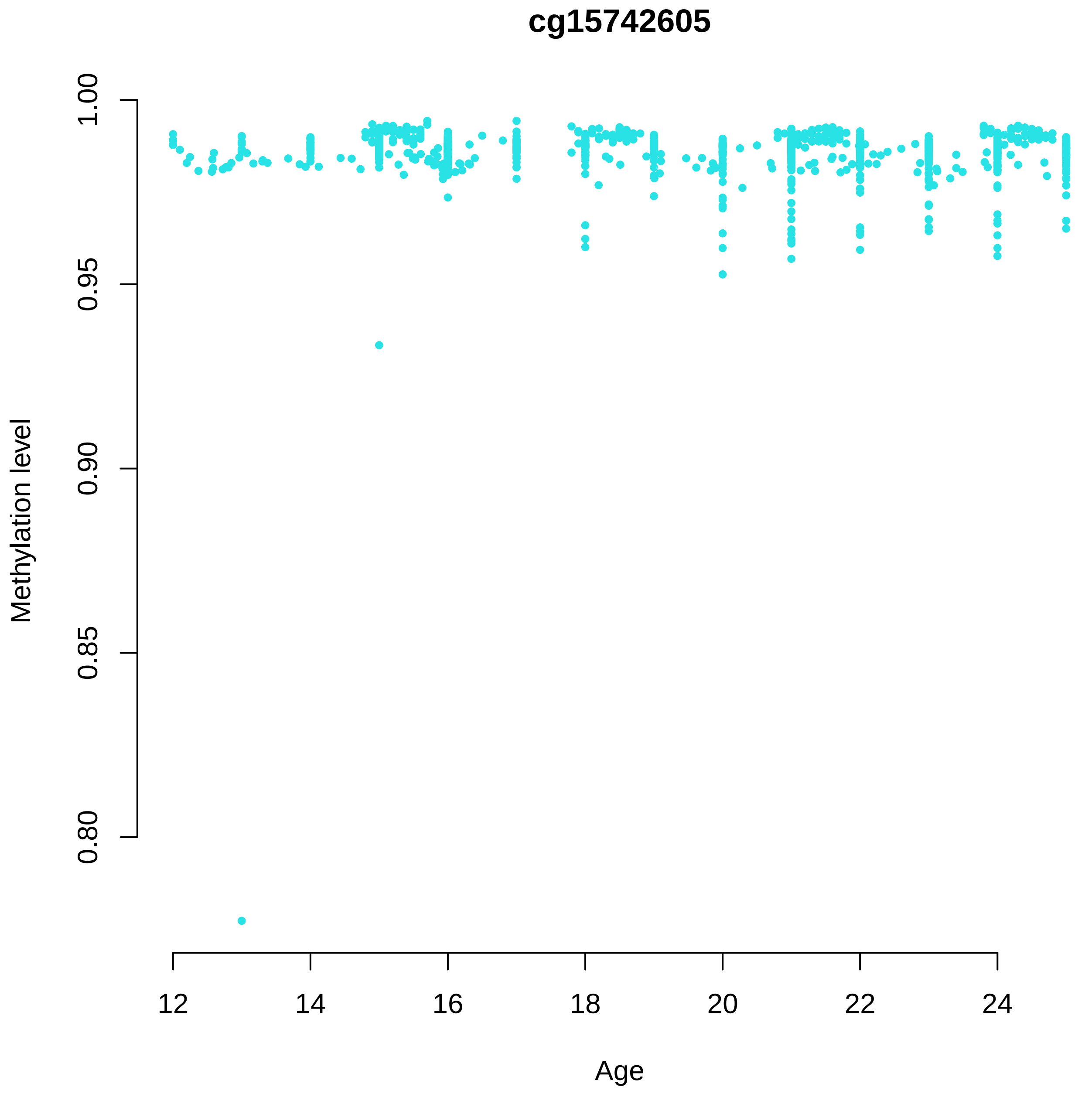
cg15742605
Methylation level
1.00
0.95
0.90
0.85
0.80
12
14
16
18
20
22
24
Age

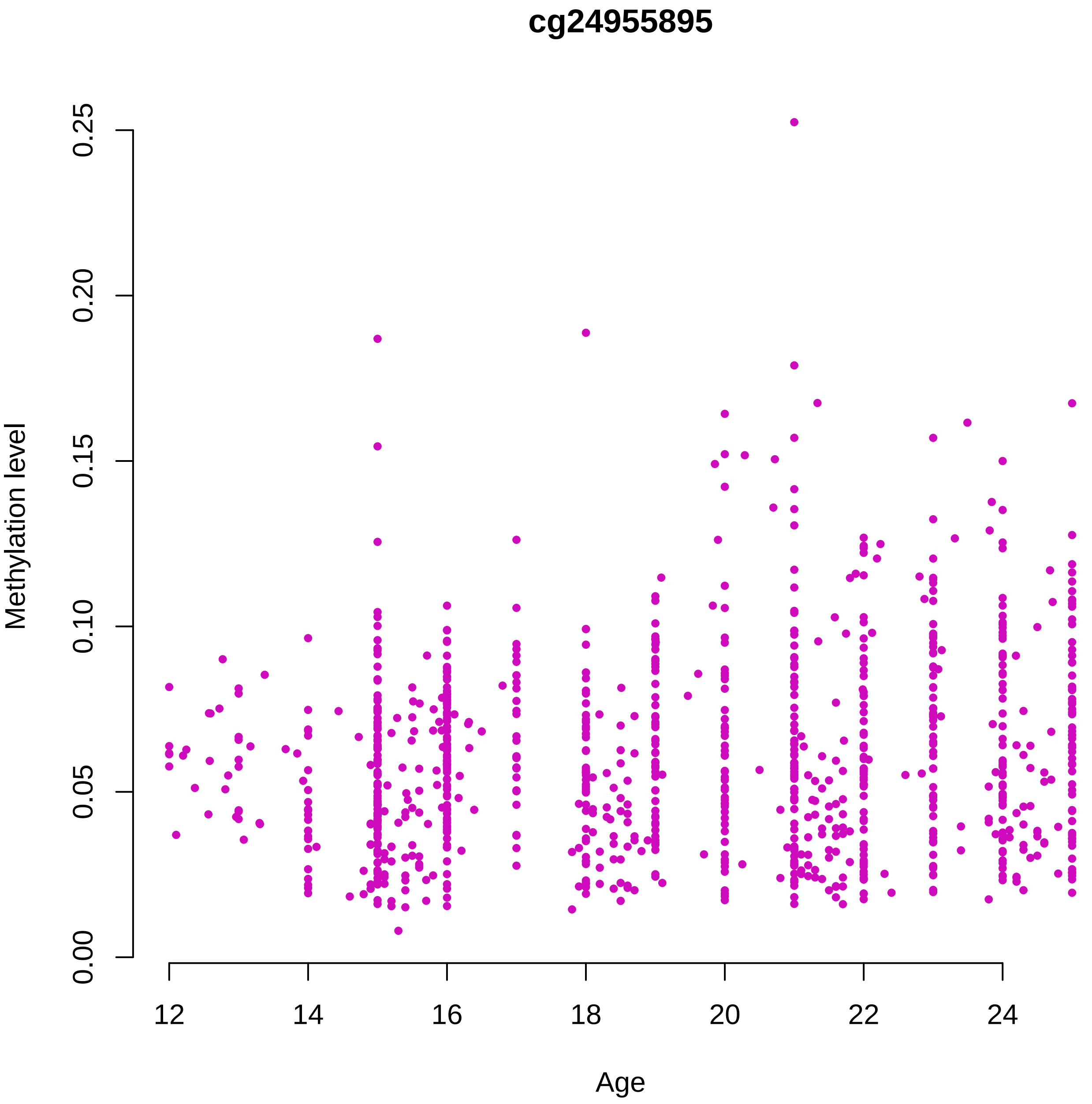
cg24955895
Methylation level
0.00
0.05
0.10
0.15
0.20
0.25
12
14
16
18
20
22
24
Age

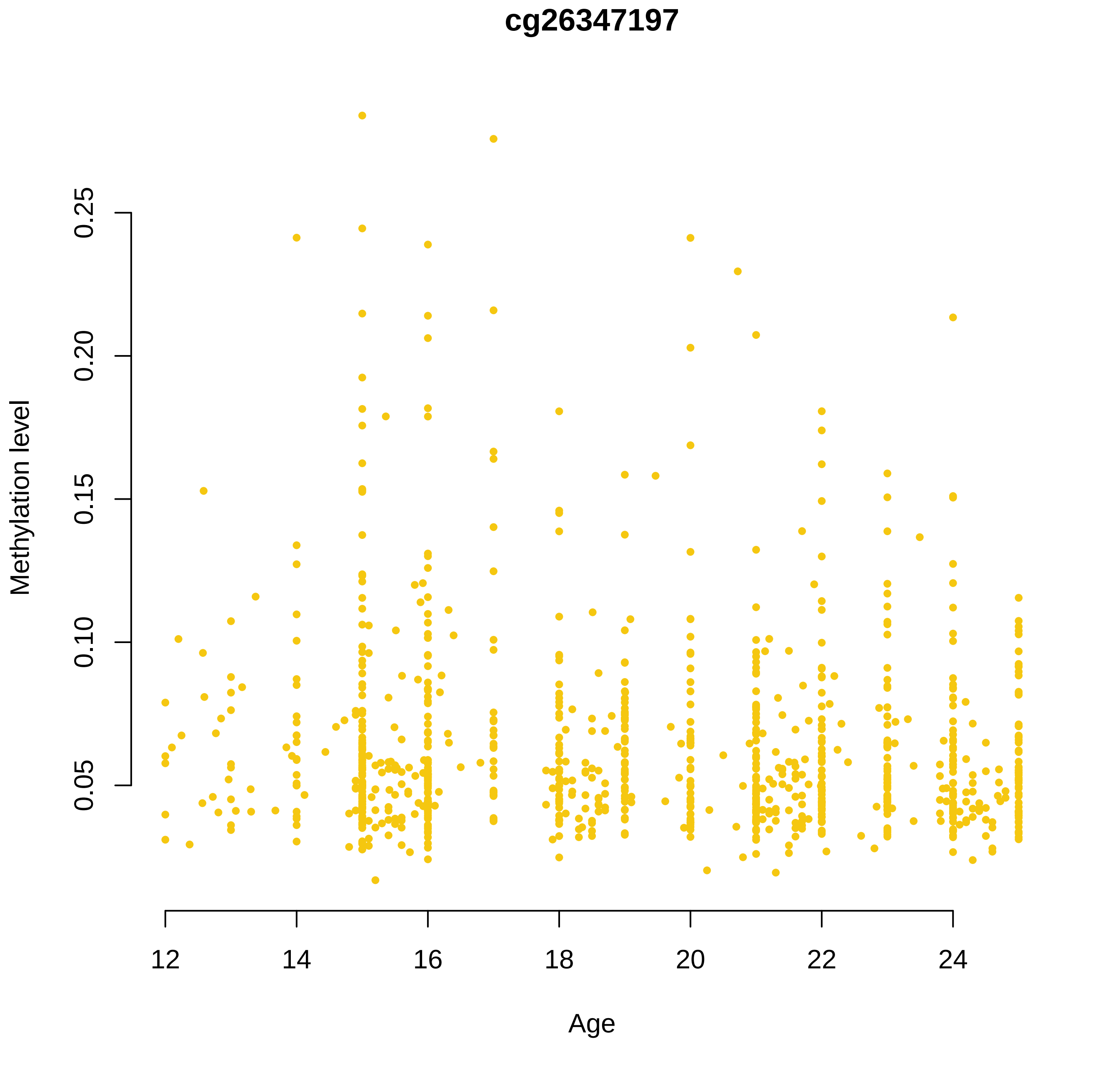
cg26347197
Methylation level
0.05
0.10
0.15
0.20
0.25
12
14
16
18
20
22
24
Age

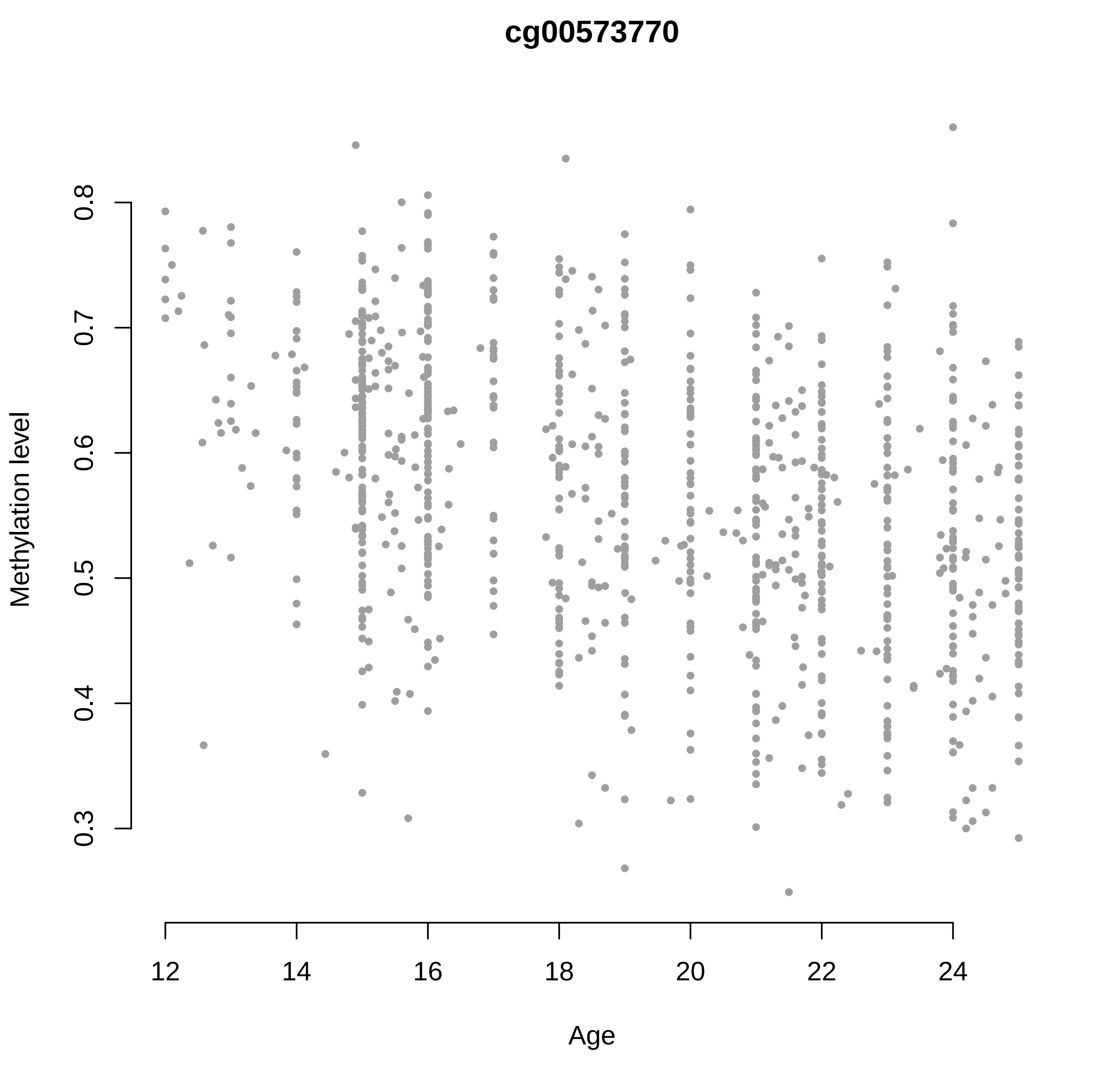
cg00573770
Methylation level
0.3
0.4
0.5
0.6
0.7
0.8
Age
12
14
16
18
20
22
24

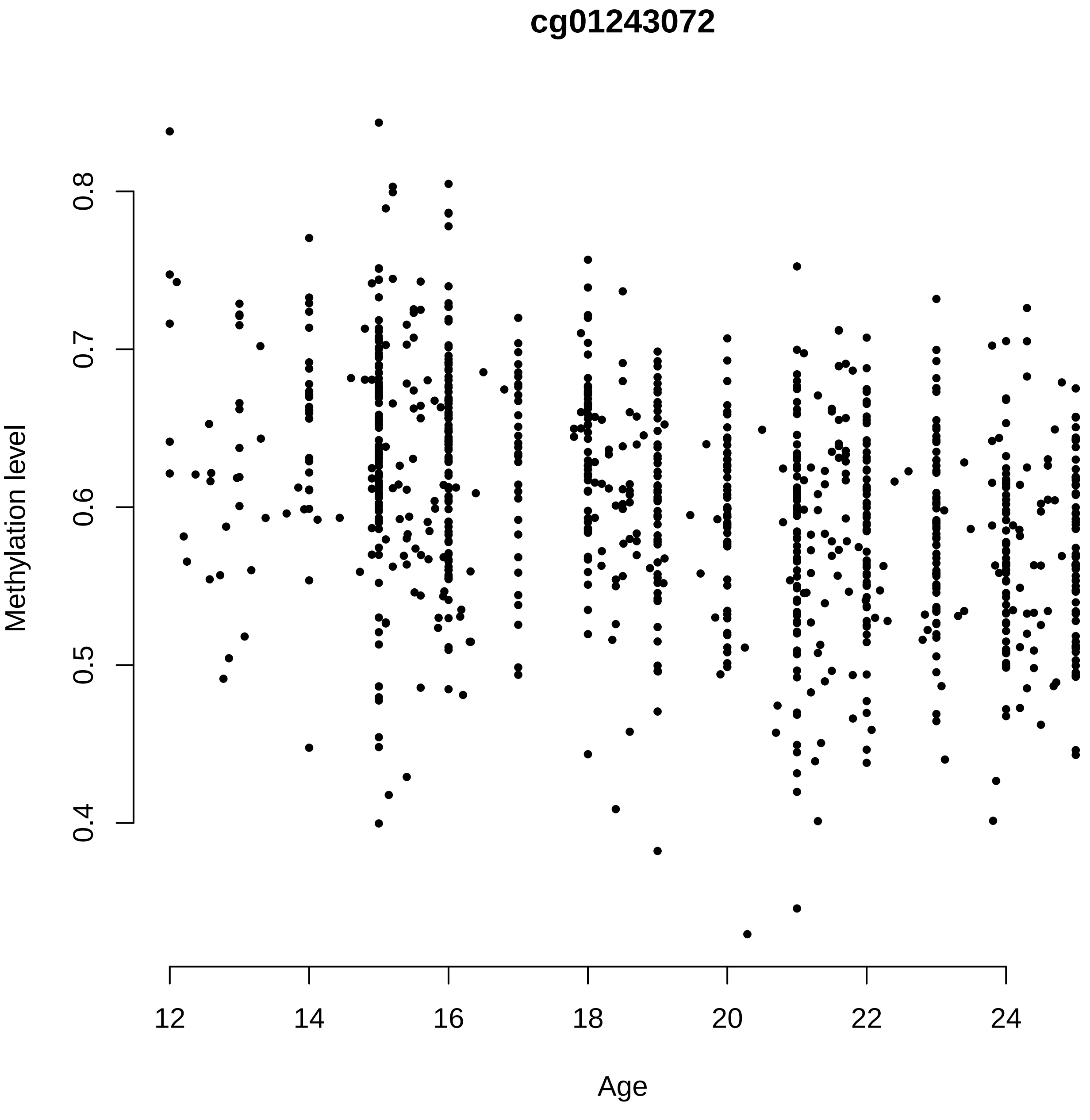
cg01243072
Methylation level
0.4
0.5
0.6
0.7
0.8
Age
12
14
16
18
20
22
24

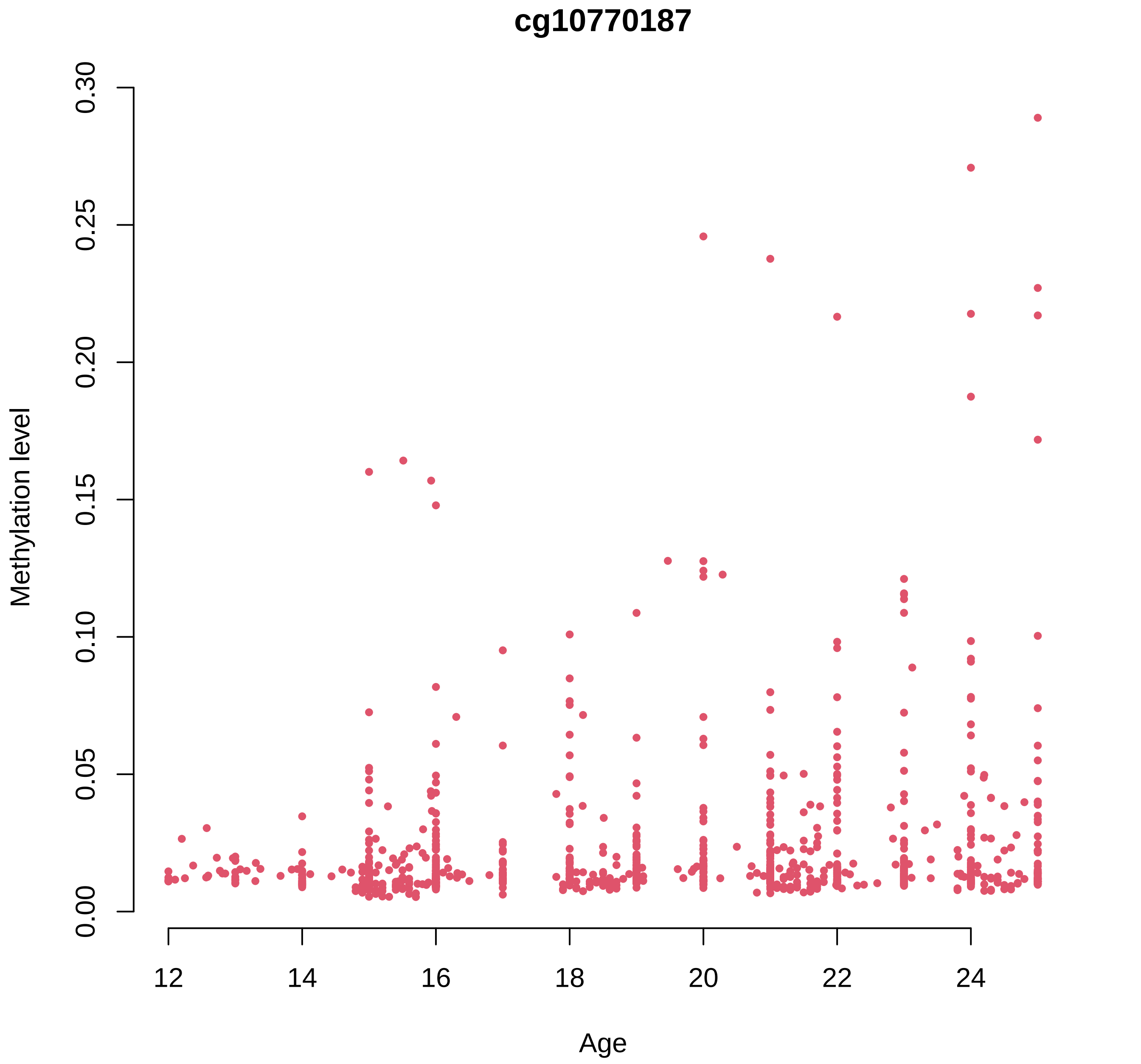
cg10770187
Methylation level
0.00
0.05
0.10
0.15
0.20
0.25
0.30
Age
12
14
16
18
20
22
24

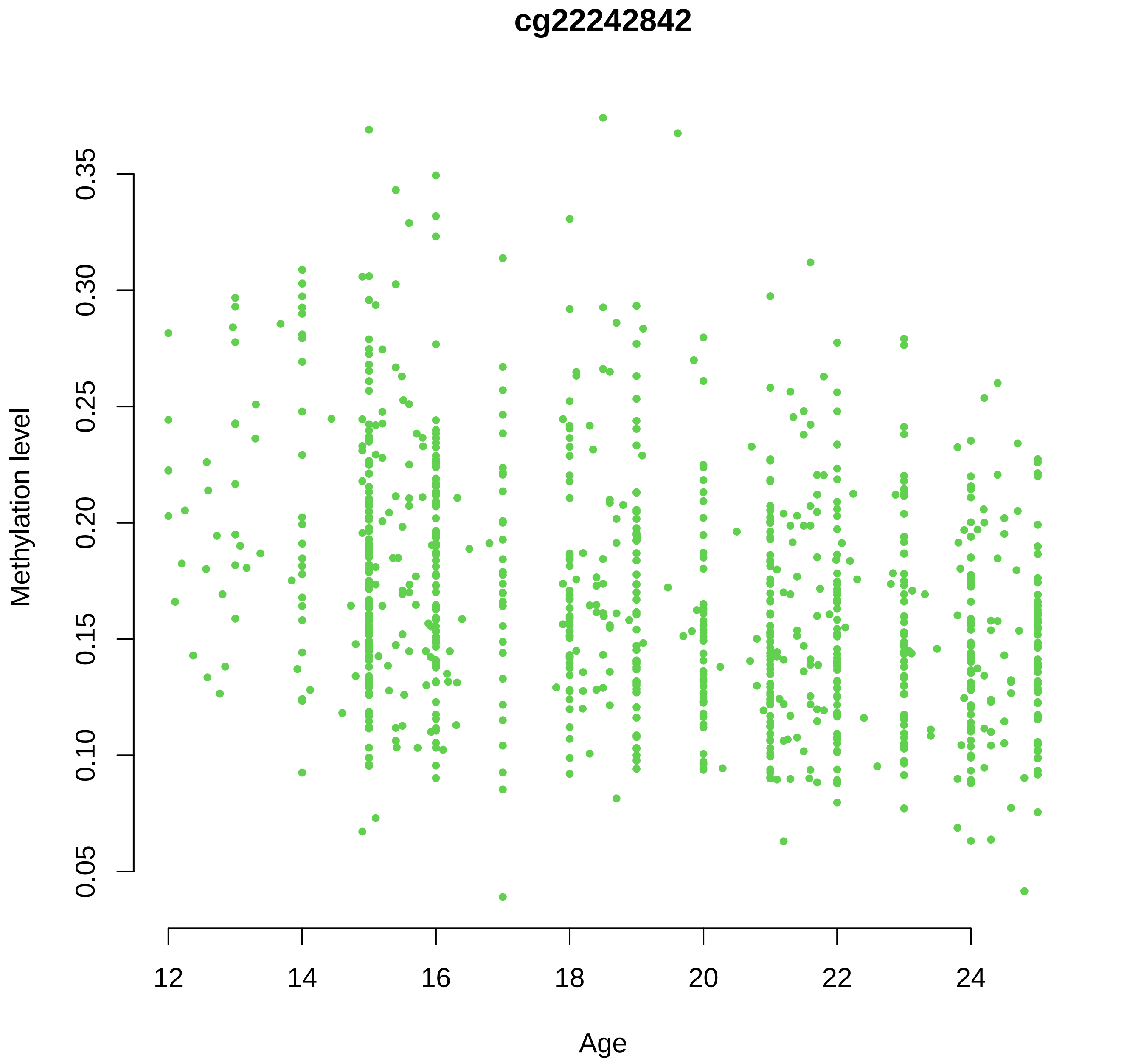

cg22242842
Methylation level
0.05
0.10
0.15
0.20
0.25
0.30
0.35
Age
12
14
16
18
20
22
24

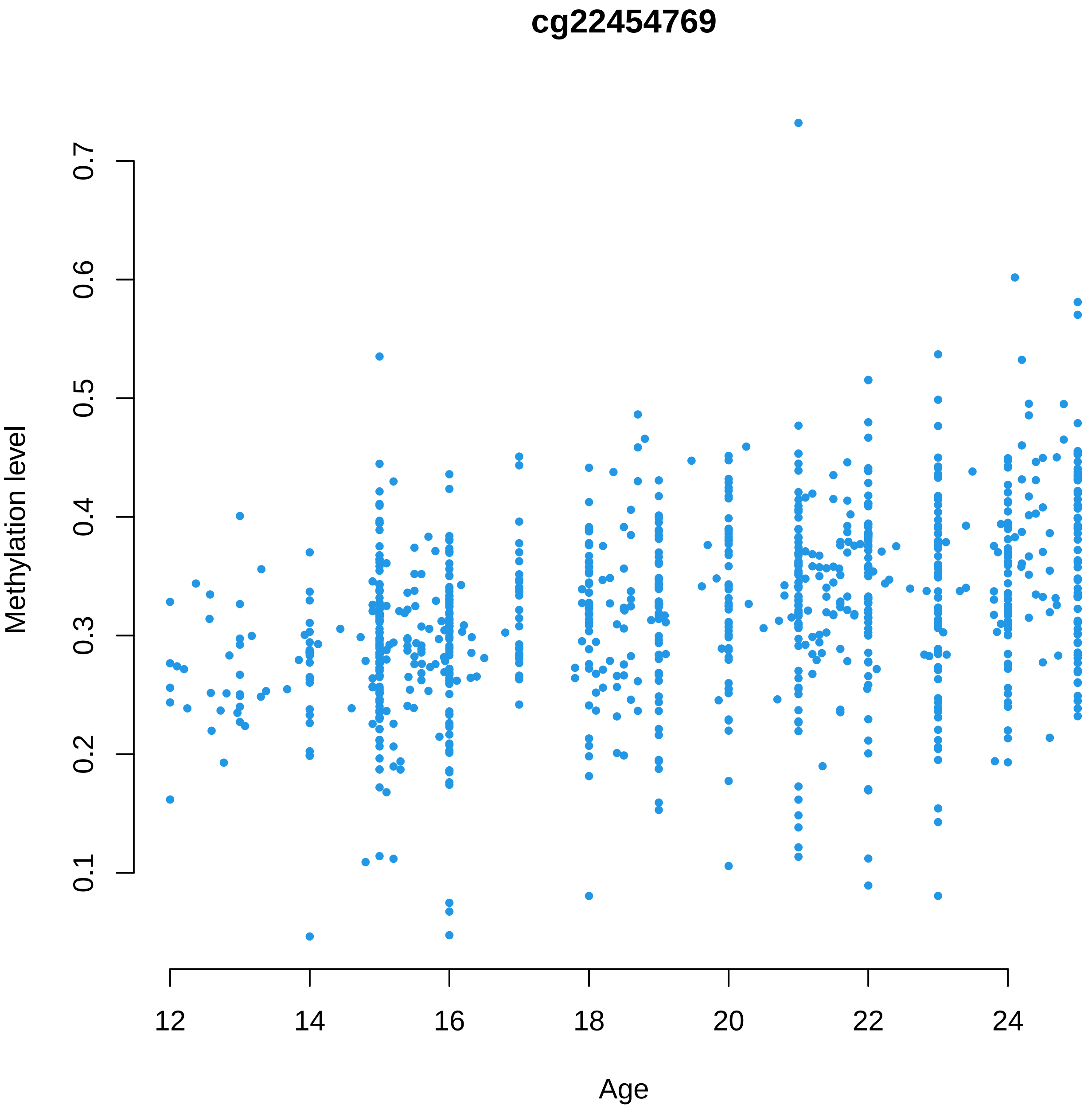
cg22454769
Methylation level
0.1
0.2
0.3
0.4
0.5
0.6
0.7
Age
12
14
16
18
20
22
24

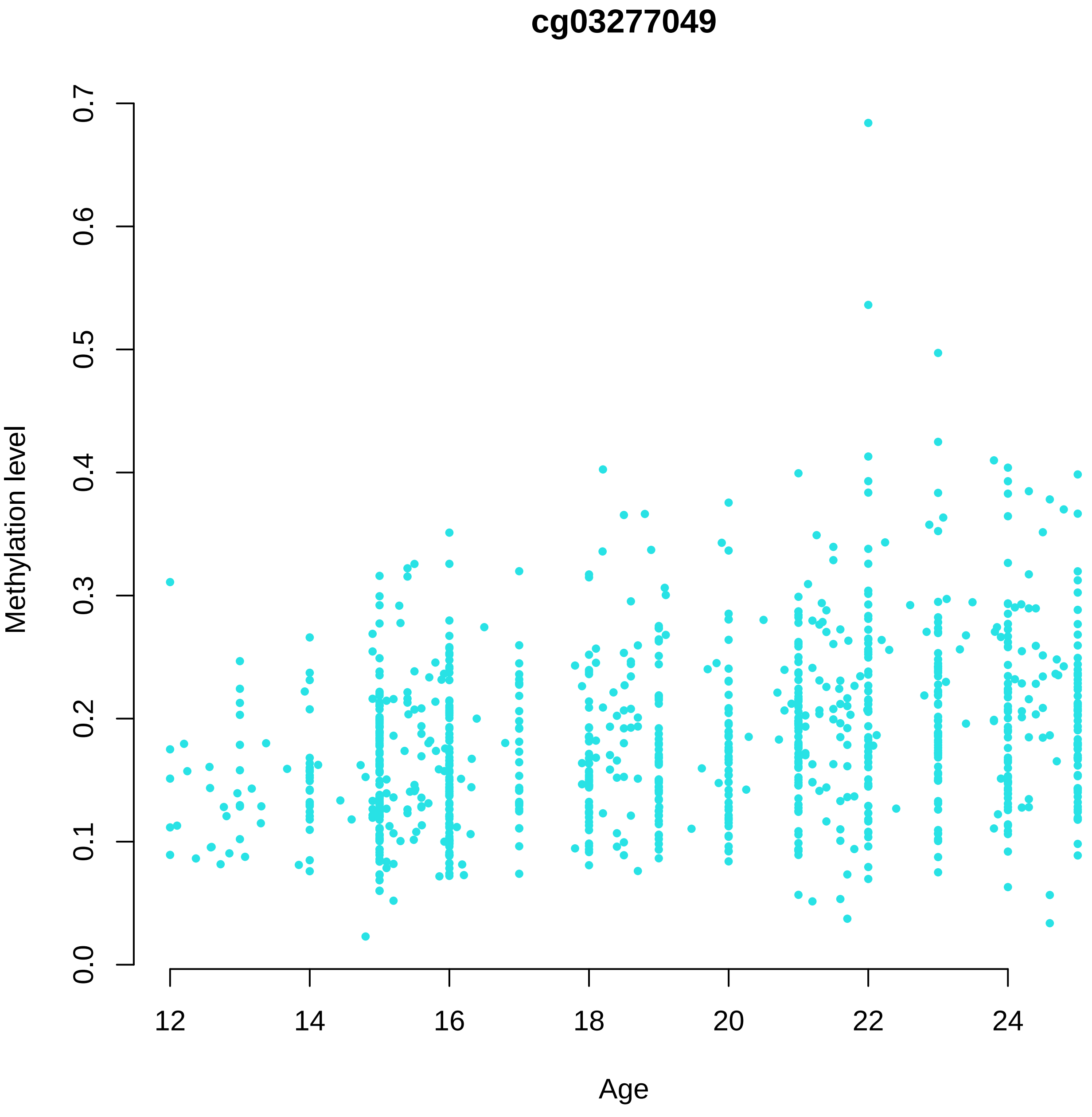
cg03277049
Methylation level
0.0
0.1
0.2
0.3
0.4
0.5
0.6
0.7
12
14
16
18
20
22
24
Age

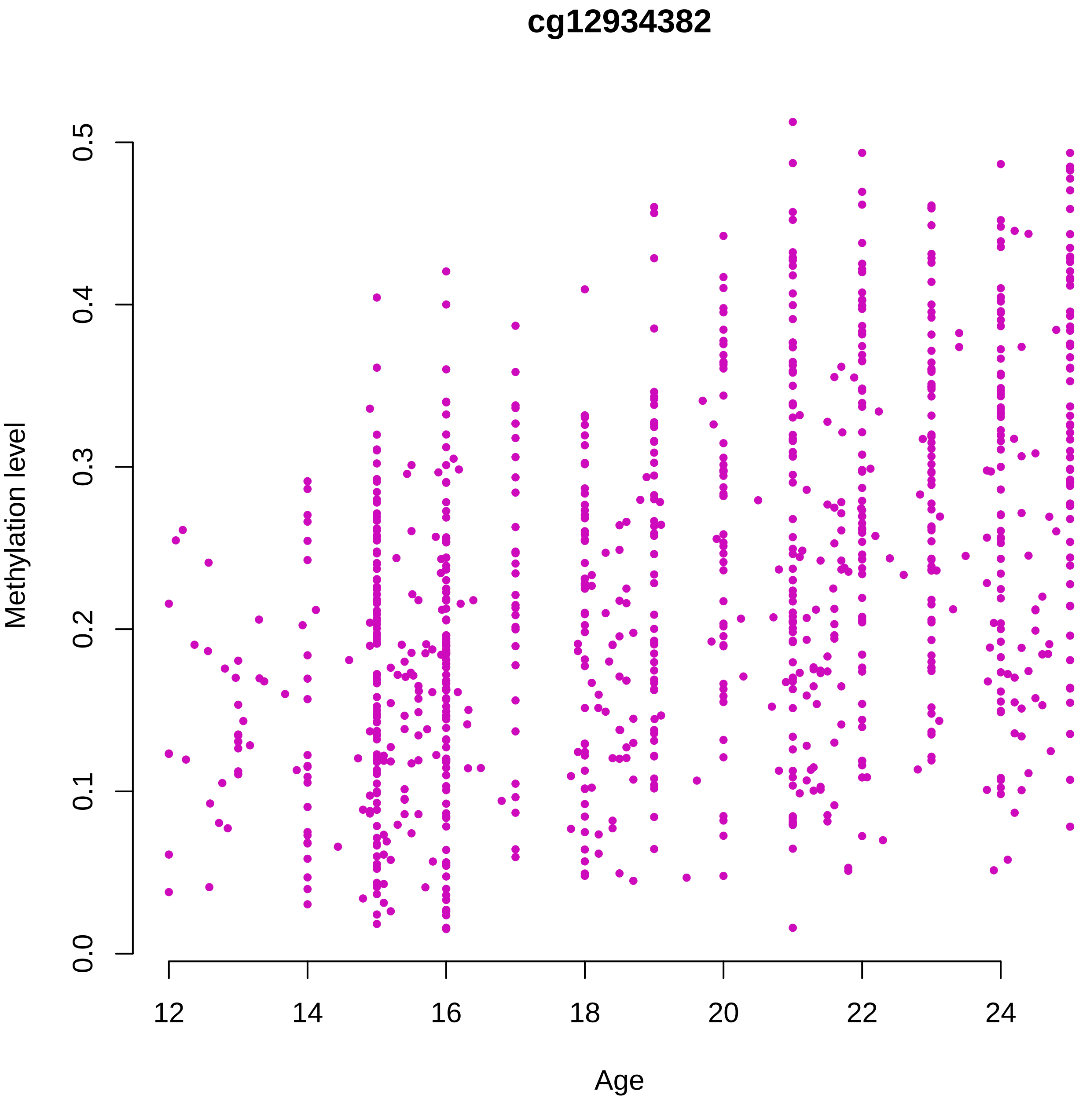

cg12934382
Methylation level
Age
0.0
0.1
0.2
0.3
0.4
0.5
12
14
16
18
20
22
24

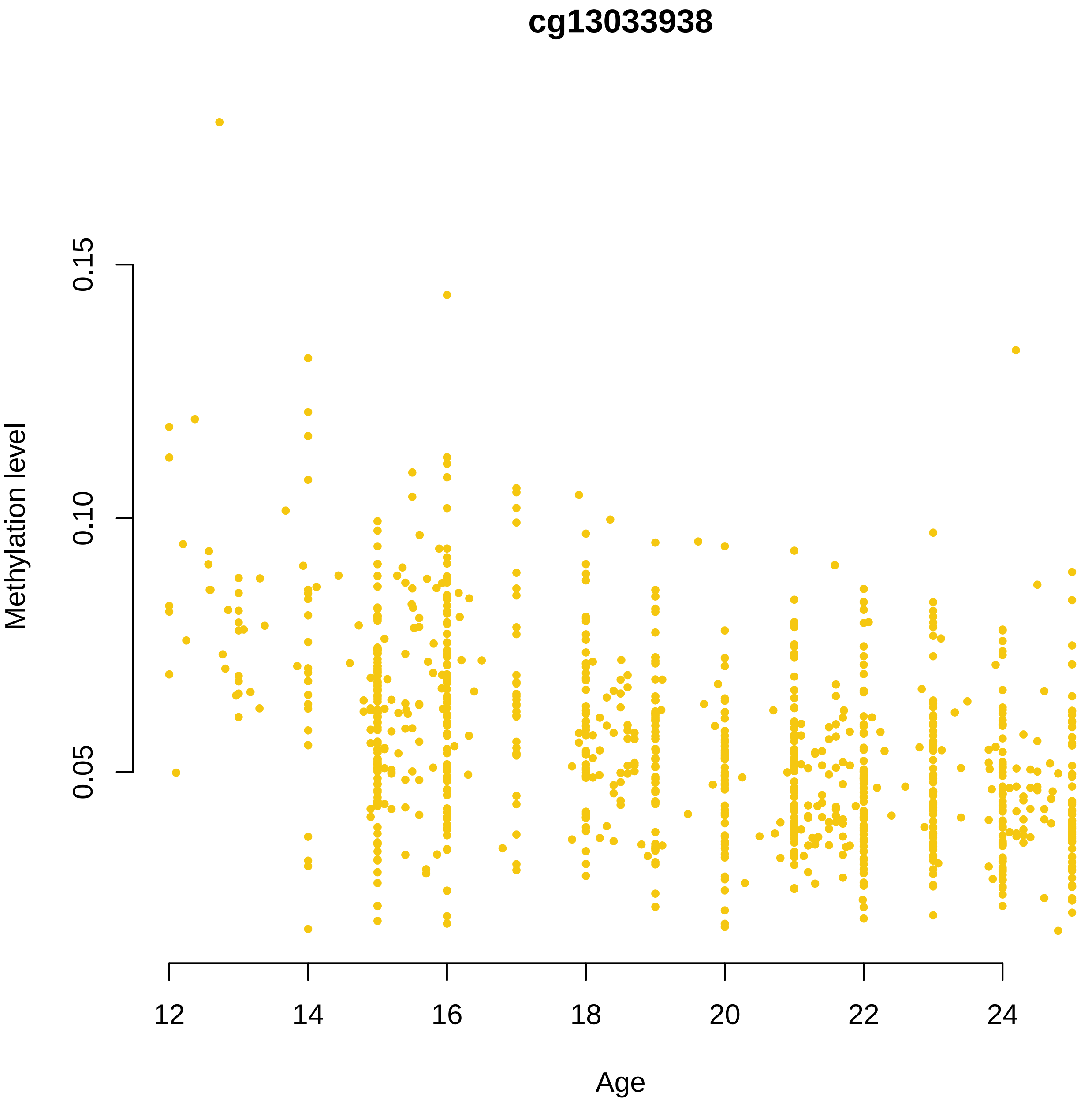
cg13033938
Methylation level
0.05
0.10
0.15
Age
12
14
16
18
20
22
24

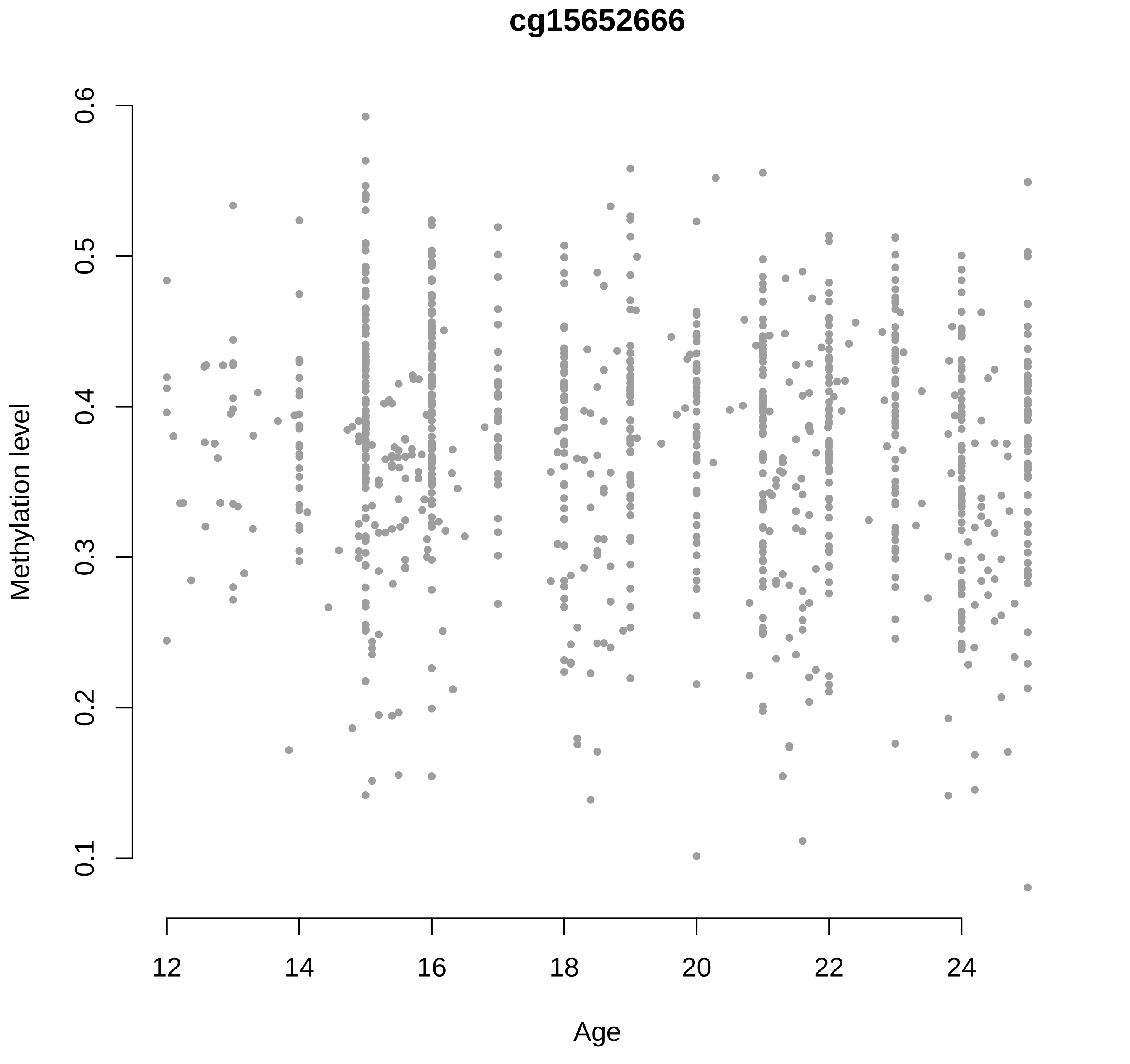
cg15652666
Methylation level
0.1
0.2
0.3
0.4
0.5
0.6
Age
12
14
16
18
20
22
24

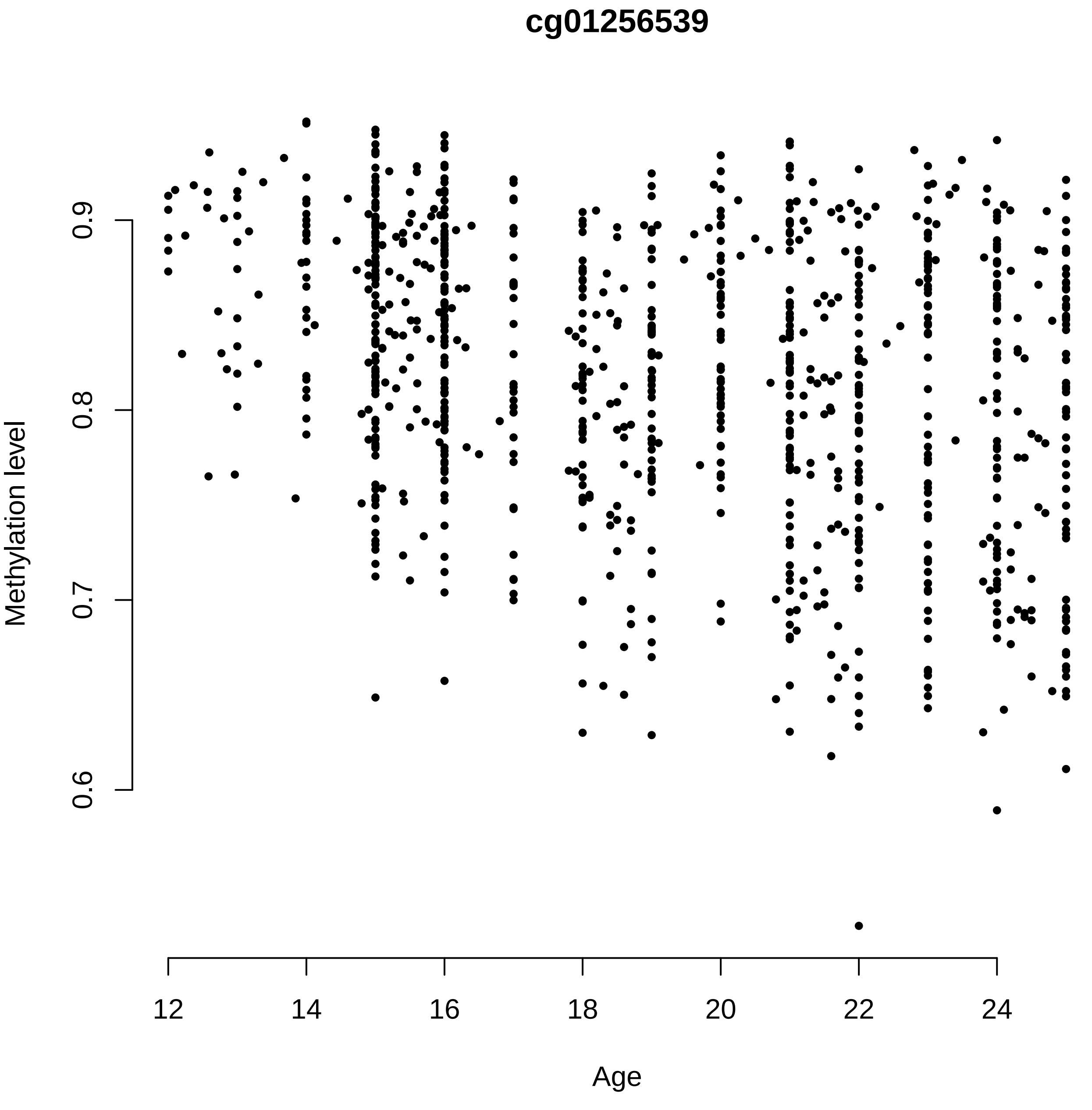
cg01256539
Methylation level
0.6
0.7
0.8
0.9
Age
12
14
16
18
20
22
24

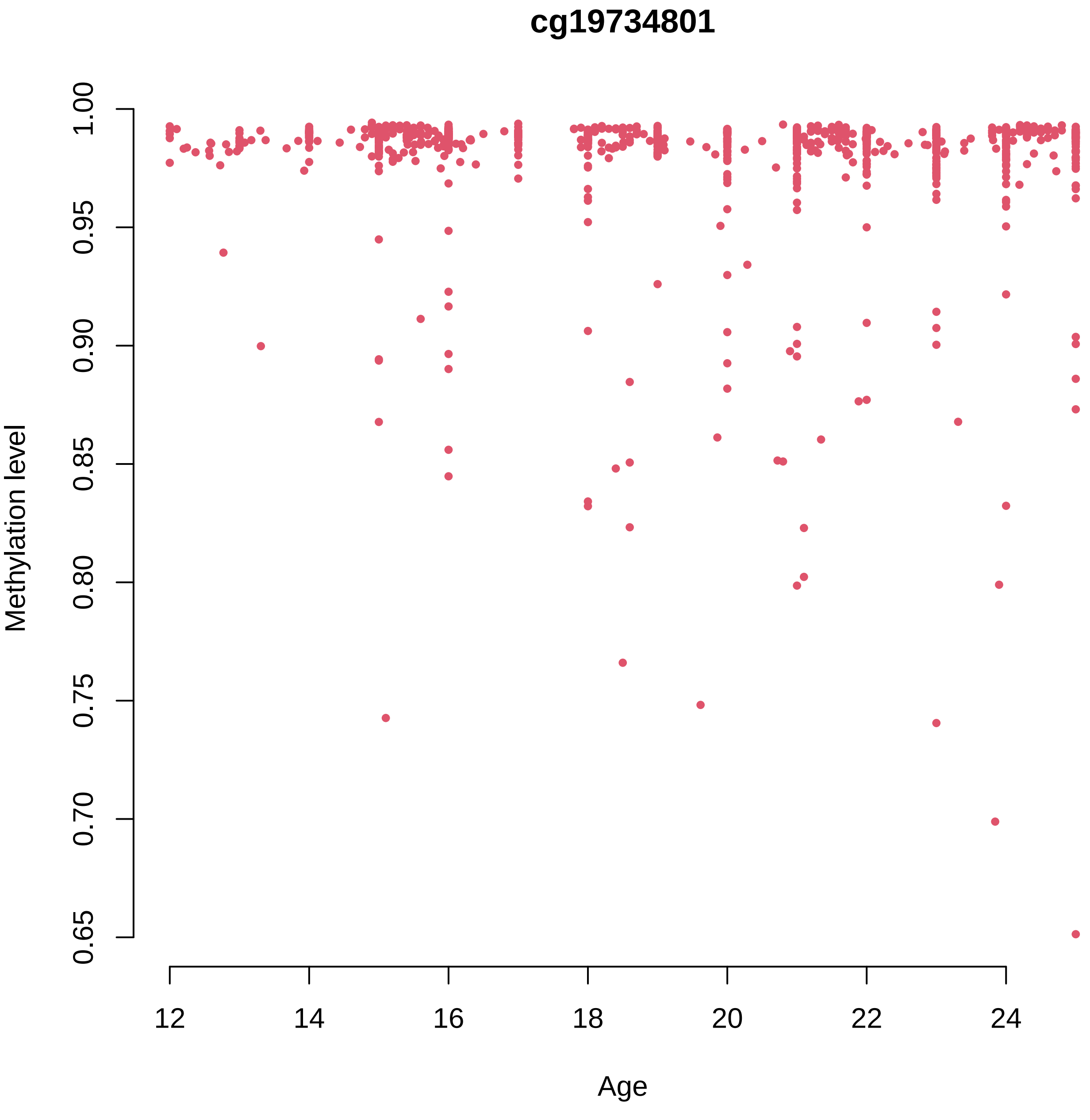

cg19734801
Methylation level
Age
0.65
0.70
0.75
0.80
0.85
0.90
0.95
1.00
12
14
16
18
20
22
24

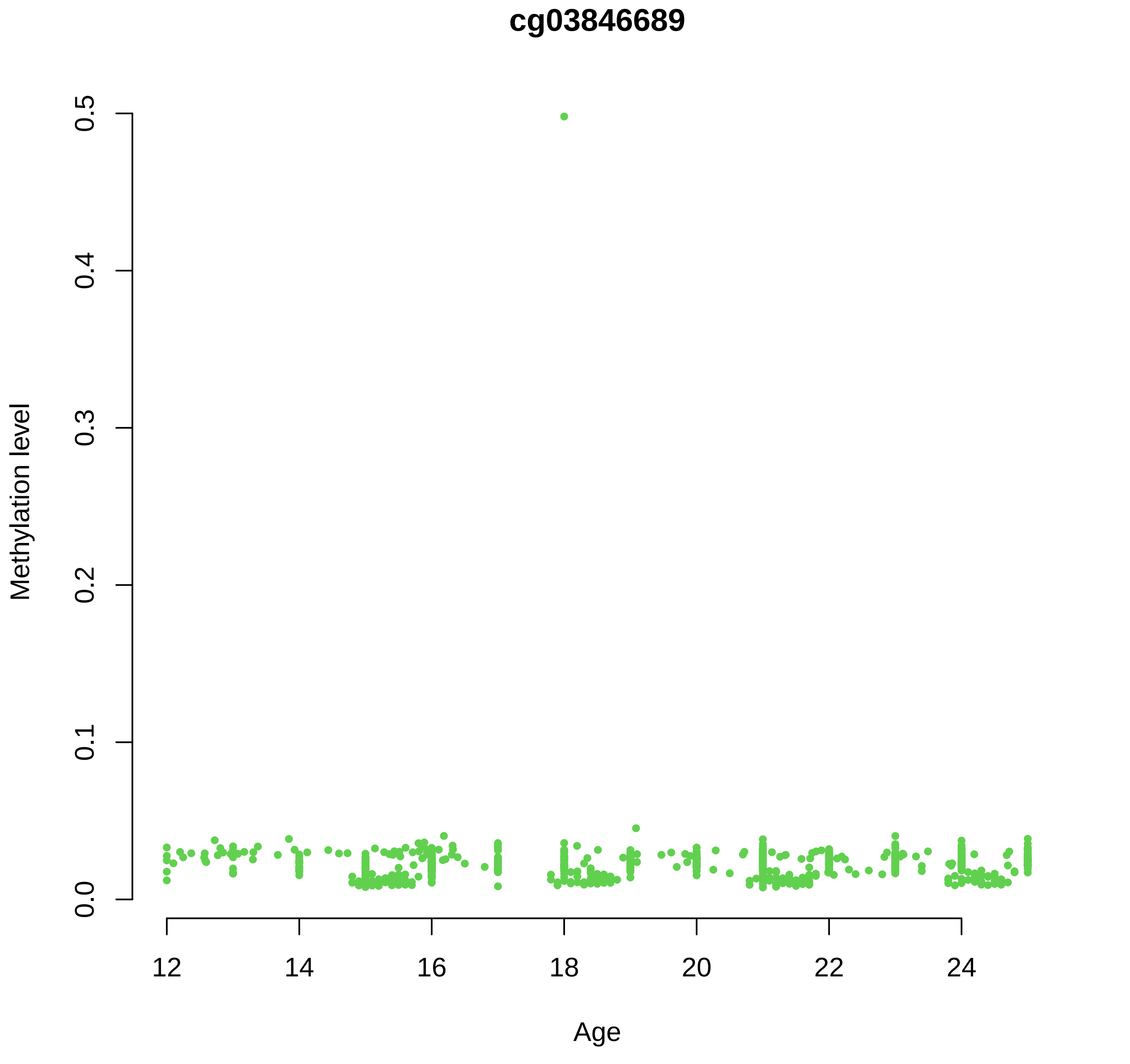

cg03846689
Methylation level
0.0
0.1
0.2
0.3
0.4
0.5
12
14
16
18
20
22
24
Age

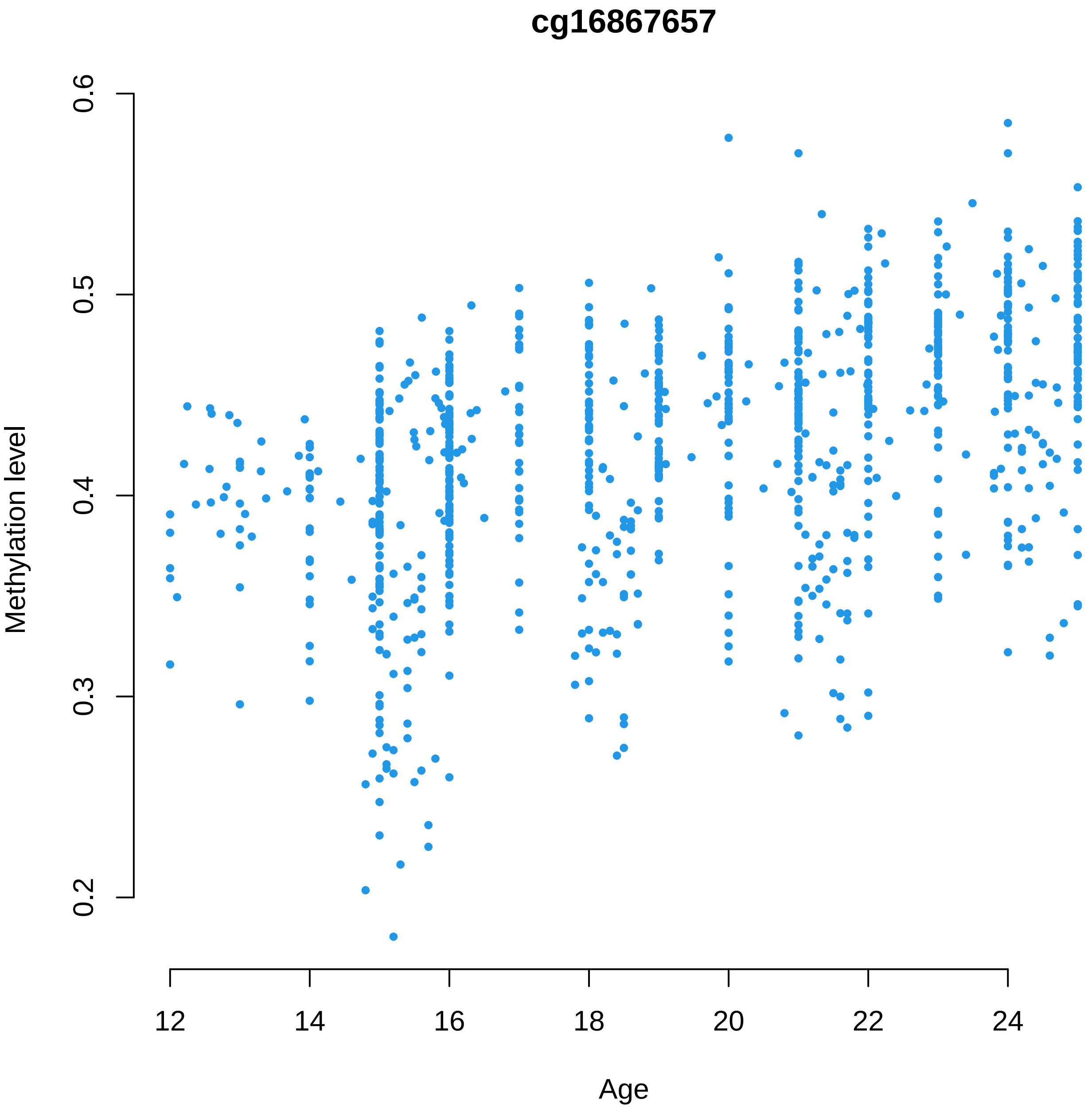
cg16867657
Methylation level
0.2
0.3
0.4
0.5
0.6
Age
12
14
16
18
20
22
24

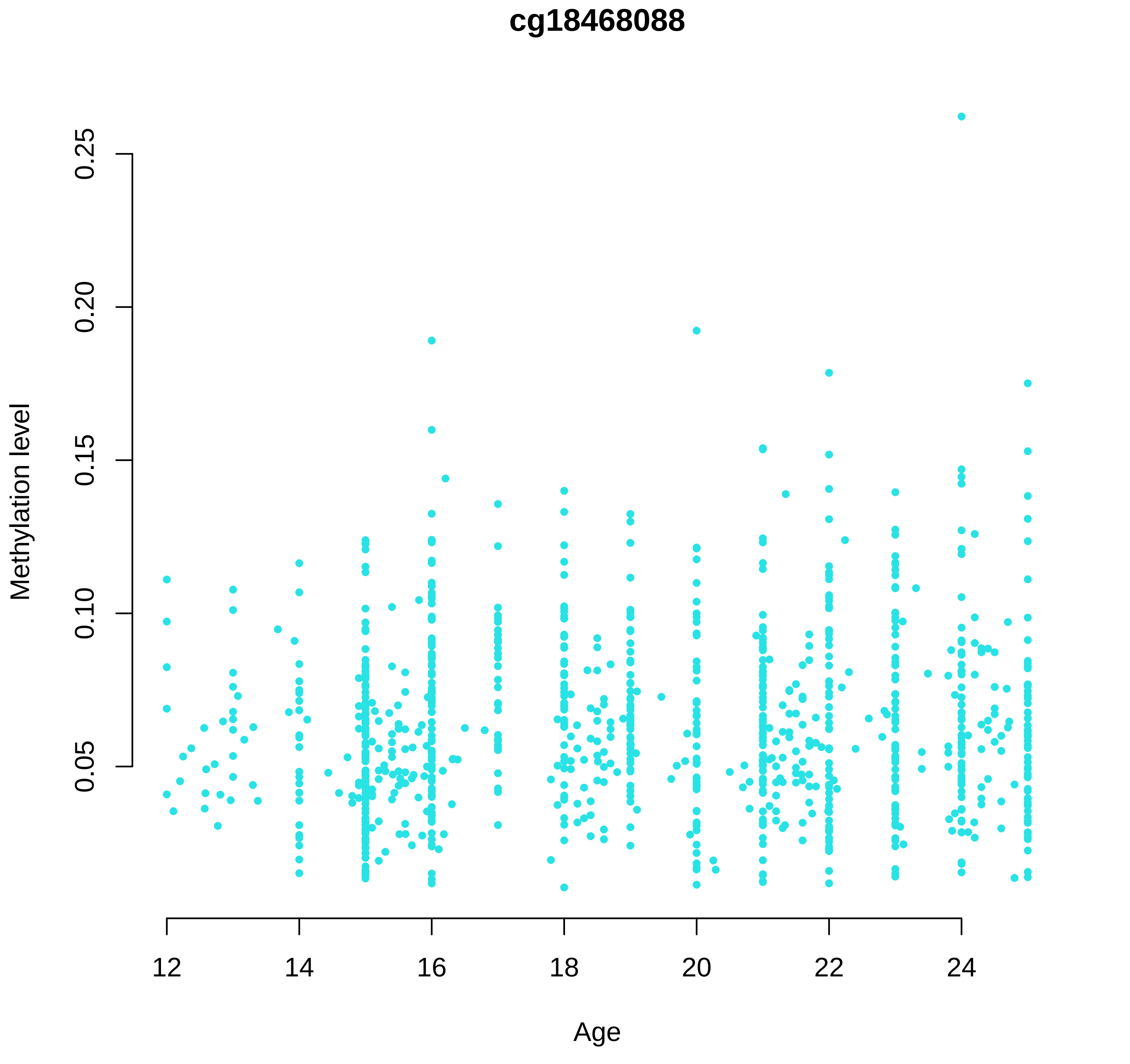
cg18468088
Methylation level
0.05
0.10
0.15
0.20
0.25
12
14
16
18
20
22
24
Age

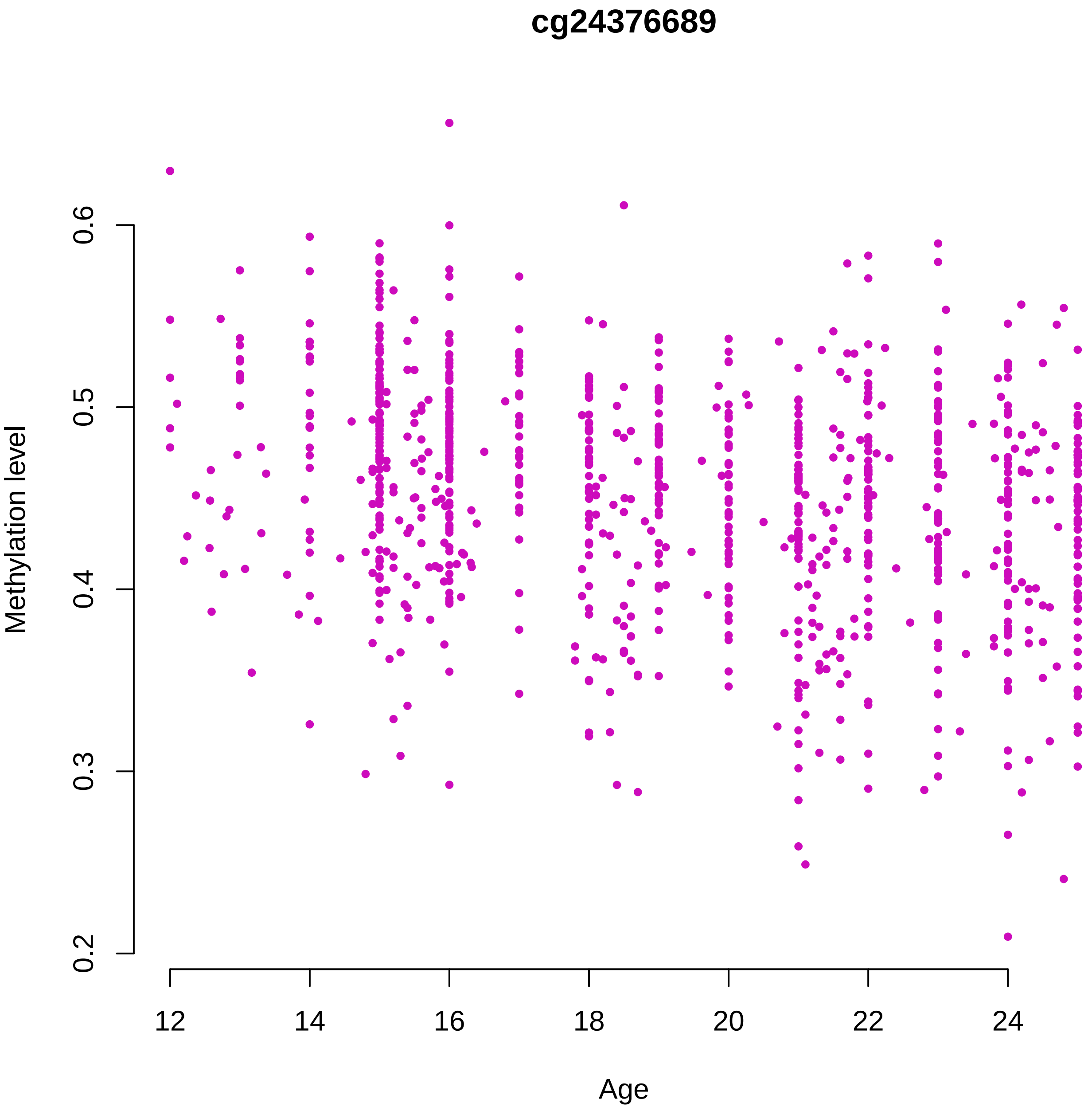
cg24376689
Methylation level
0.2
0.3
0.4
0.5
0.6
Age
12
14
16
18
20
22
24

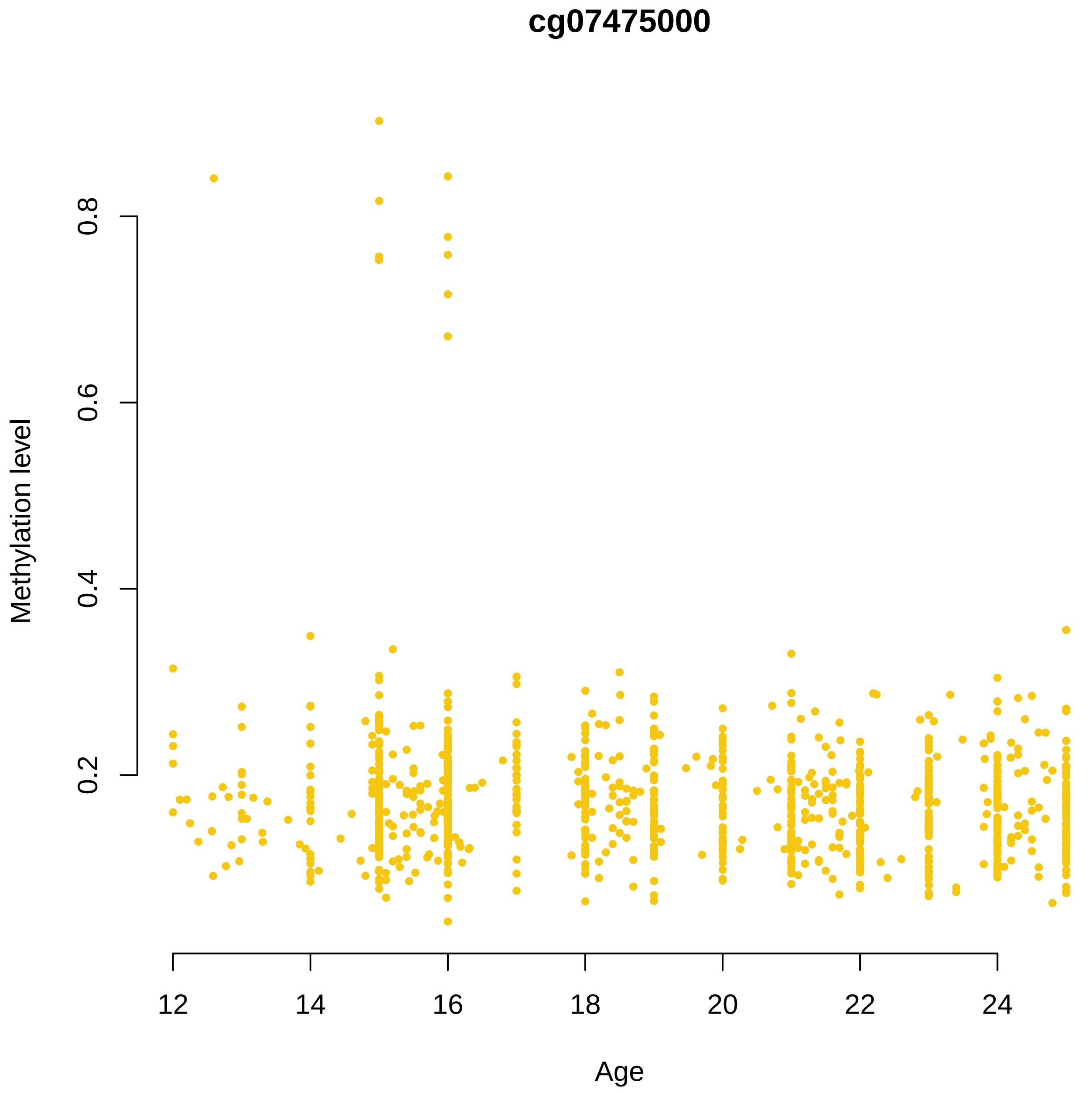

cg07475000
Methylation level
0.2
0.4
0.6
0.8
12
14
16
18
20
22
24
Age

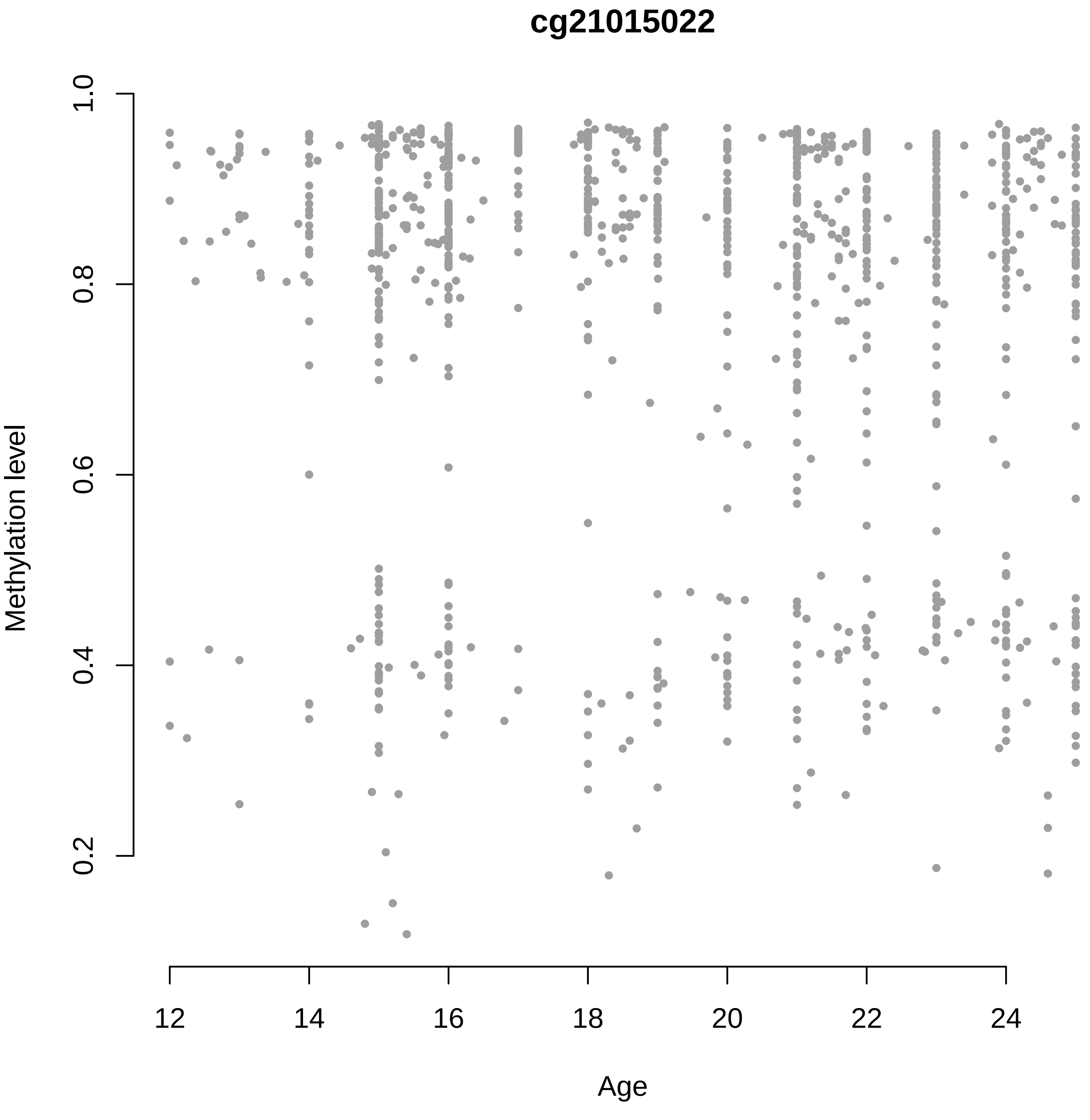
cg21015022
Methylation level
0.2
0.4
0.6
0.8
1.0
12
14
16
18
20
22
24
Age

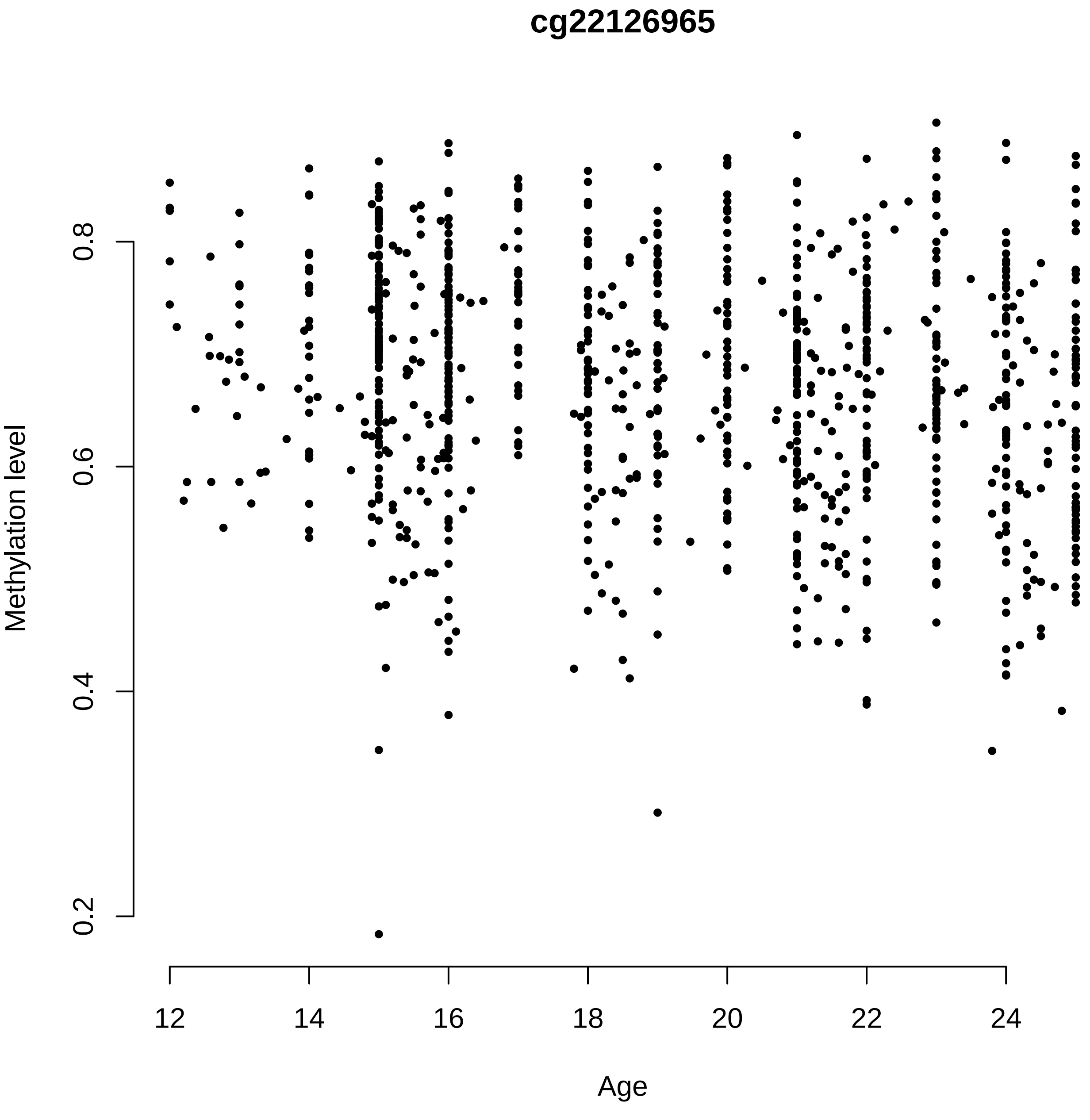
cg22126965
Methylation level
0.2
0.4
0.6
0.8
12
14
16
18
20
22
24
Age

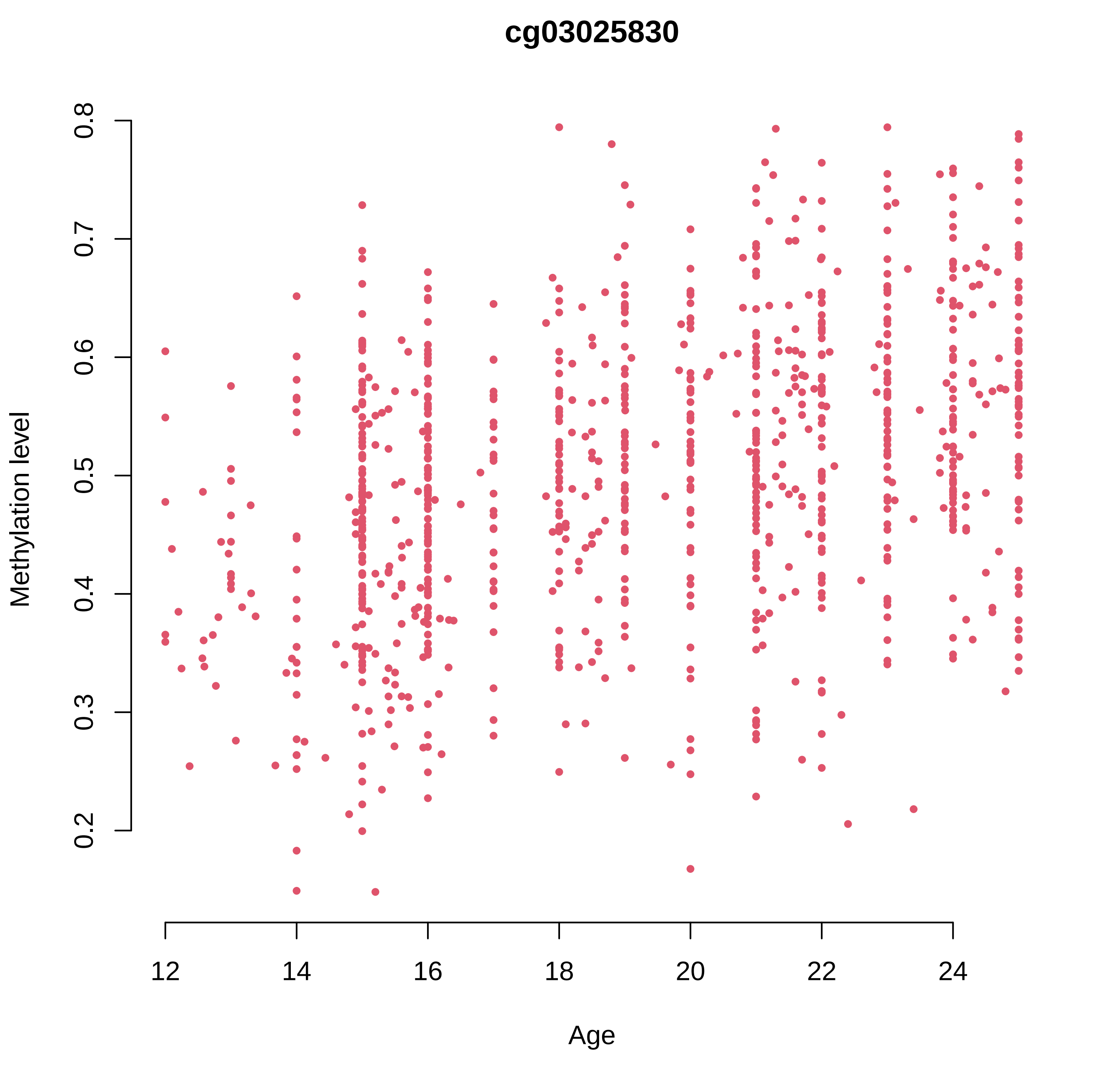
cg03025830
Methylation level
0.2
0.3
0.4
0.5
0.6
0.7
0.8
12
14
16
18
20
22
24
Age

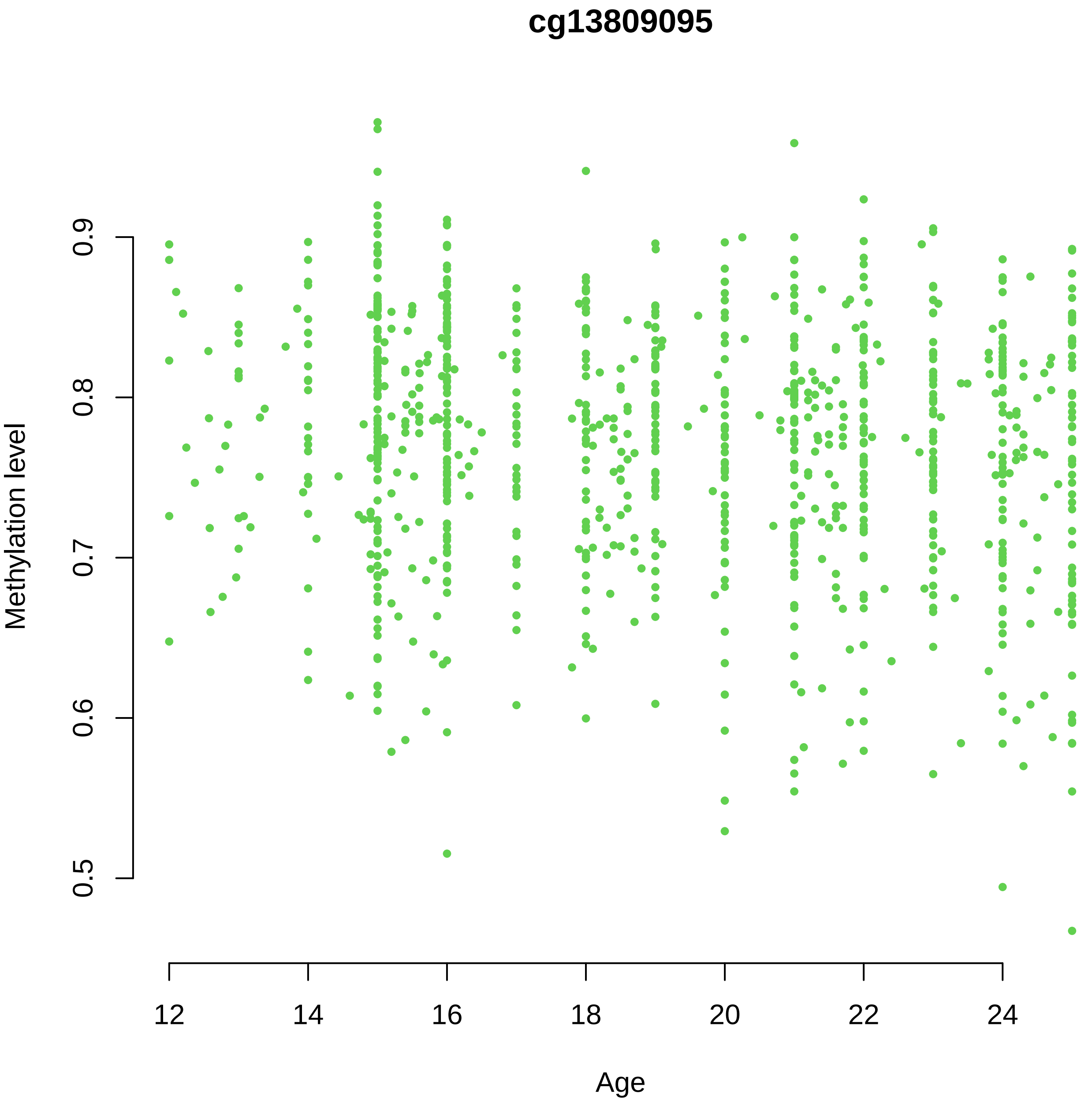
cg13809095
Methylation level
0.5
0.6
0.7
0.8
0.9
Age
12
14
16
18
20
22
24

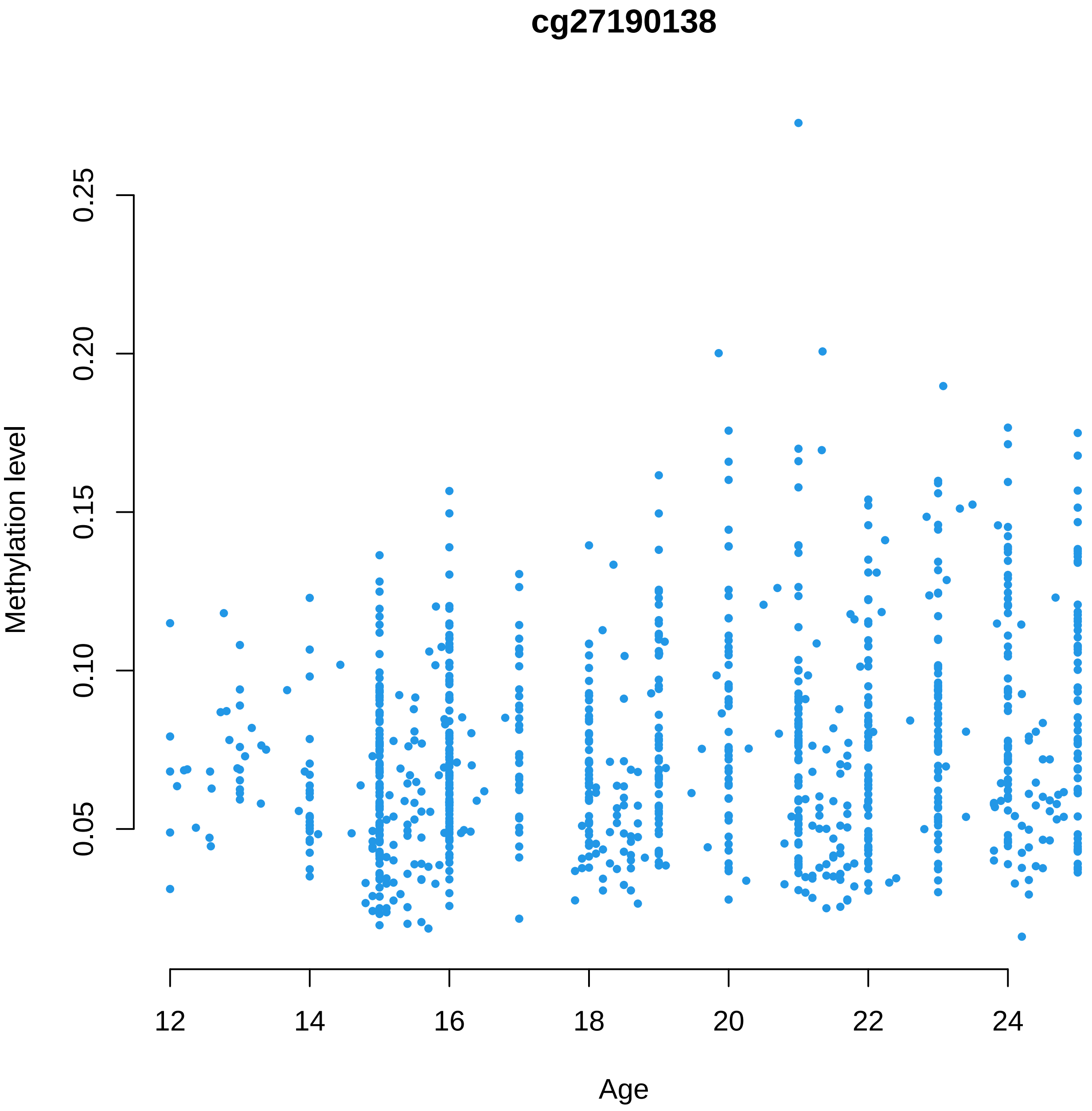
cg27190138
Methylation level
0.05
0.10
0.15
0.20
0.25
Age
12
14
16
18
20
22
24

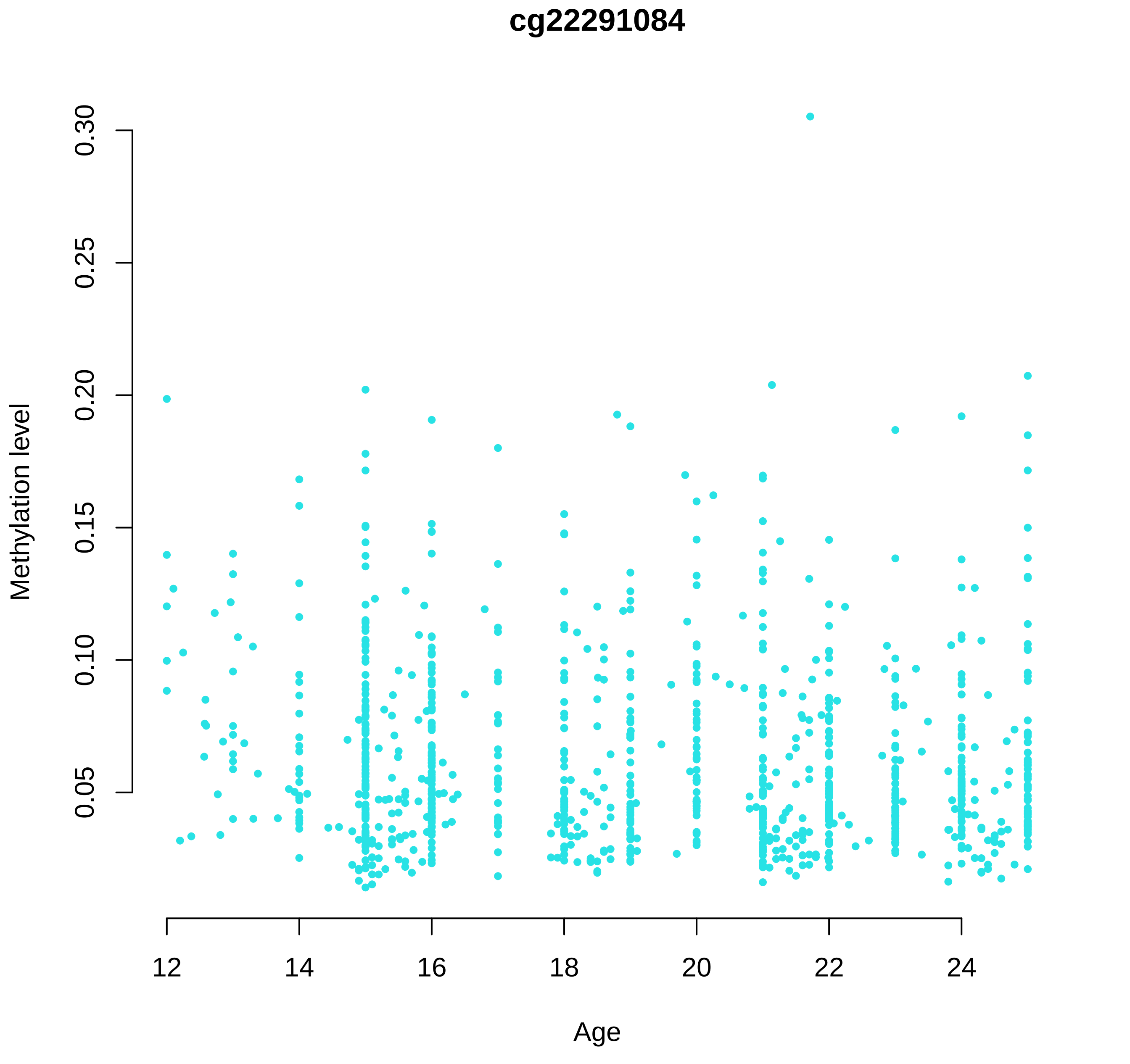

cg22291084
Methylation level
0.05
0.10
0.15
0.20
0.25
0.30
Age
12
14
16
18
20
22
24

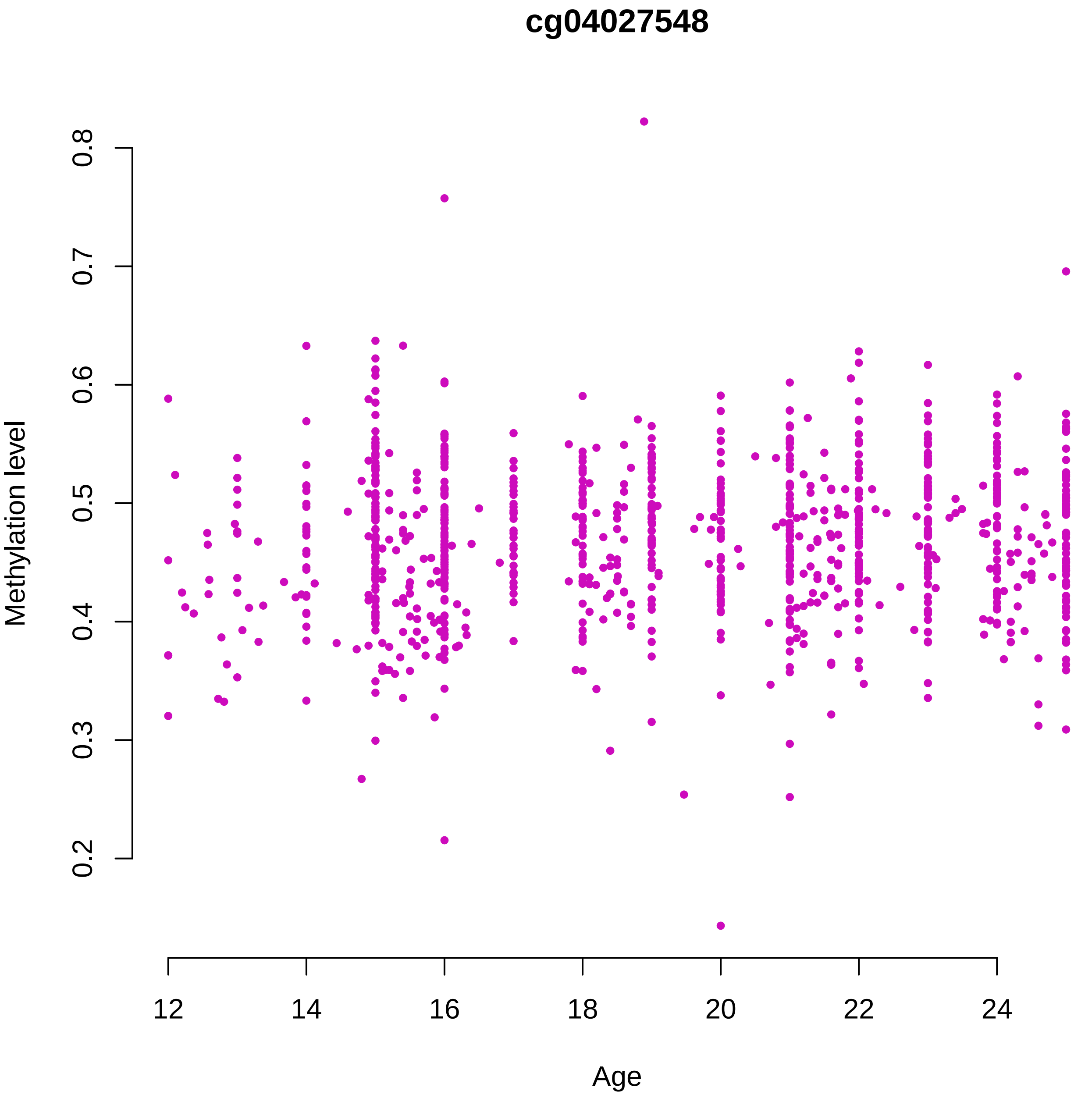

cg04027548
Methylation level
0.2
0.3
0.4
0.5
0.6
0.7
0.8
Age
12
14
16
18
20
22
24

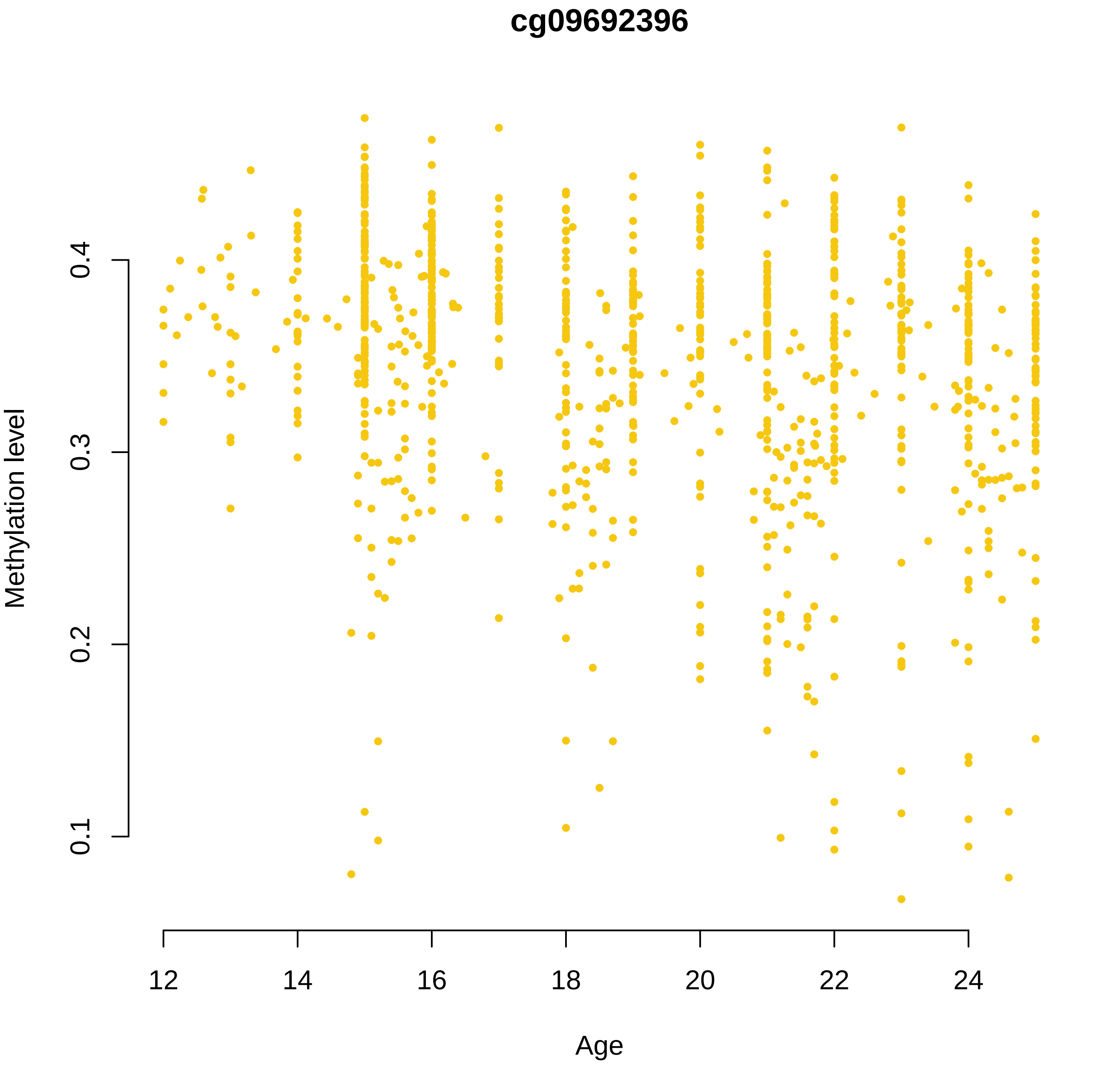

cg09692396
Methylation level
0.1
0.2
0.3
0.4
12
14
16
18
20
22
24
Age

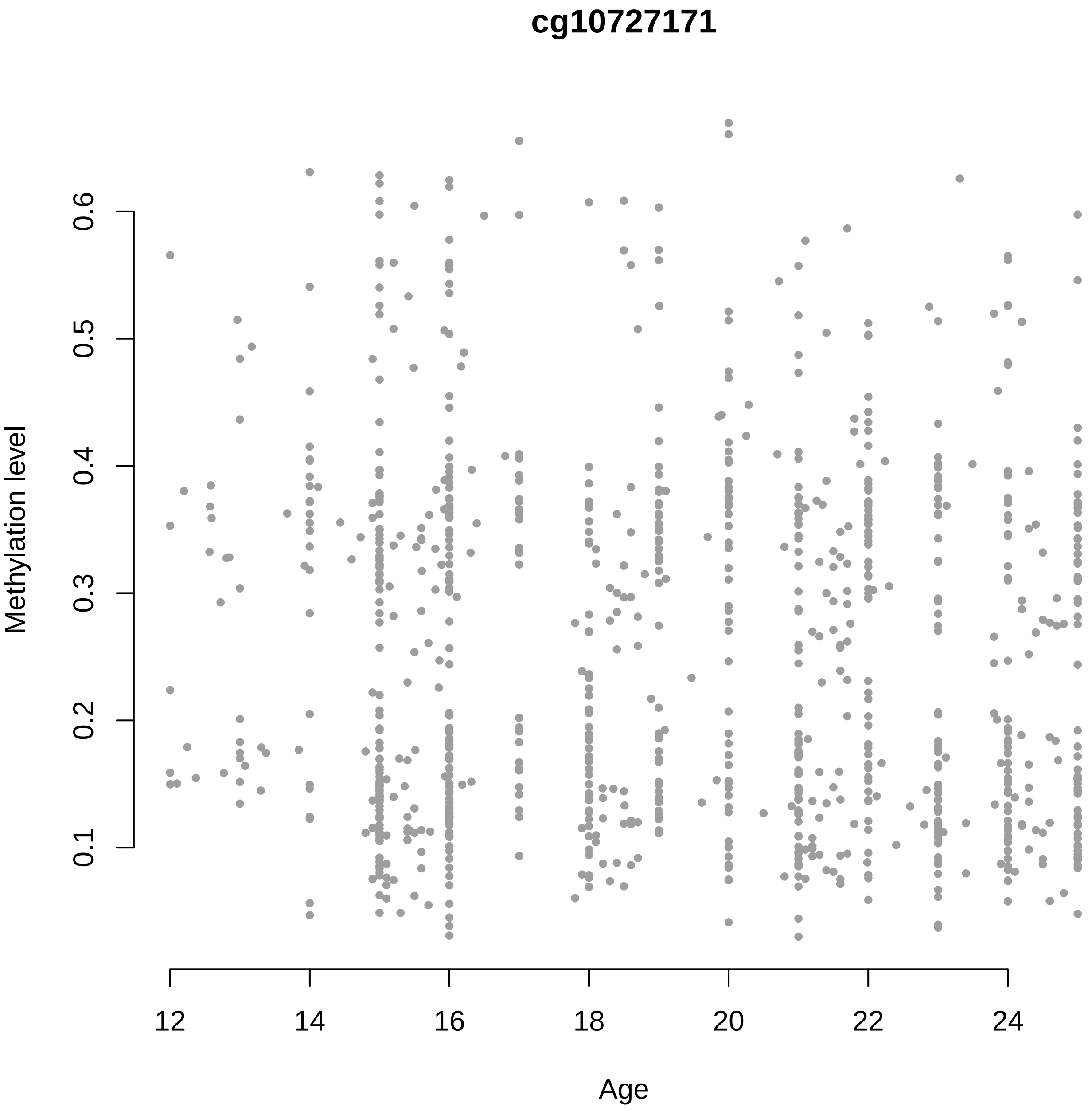
cg10727171
Methylation level
0.1
0.2
0.3
0.4
0.5
0.6
Age
12
14
16
18
20
22
24

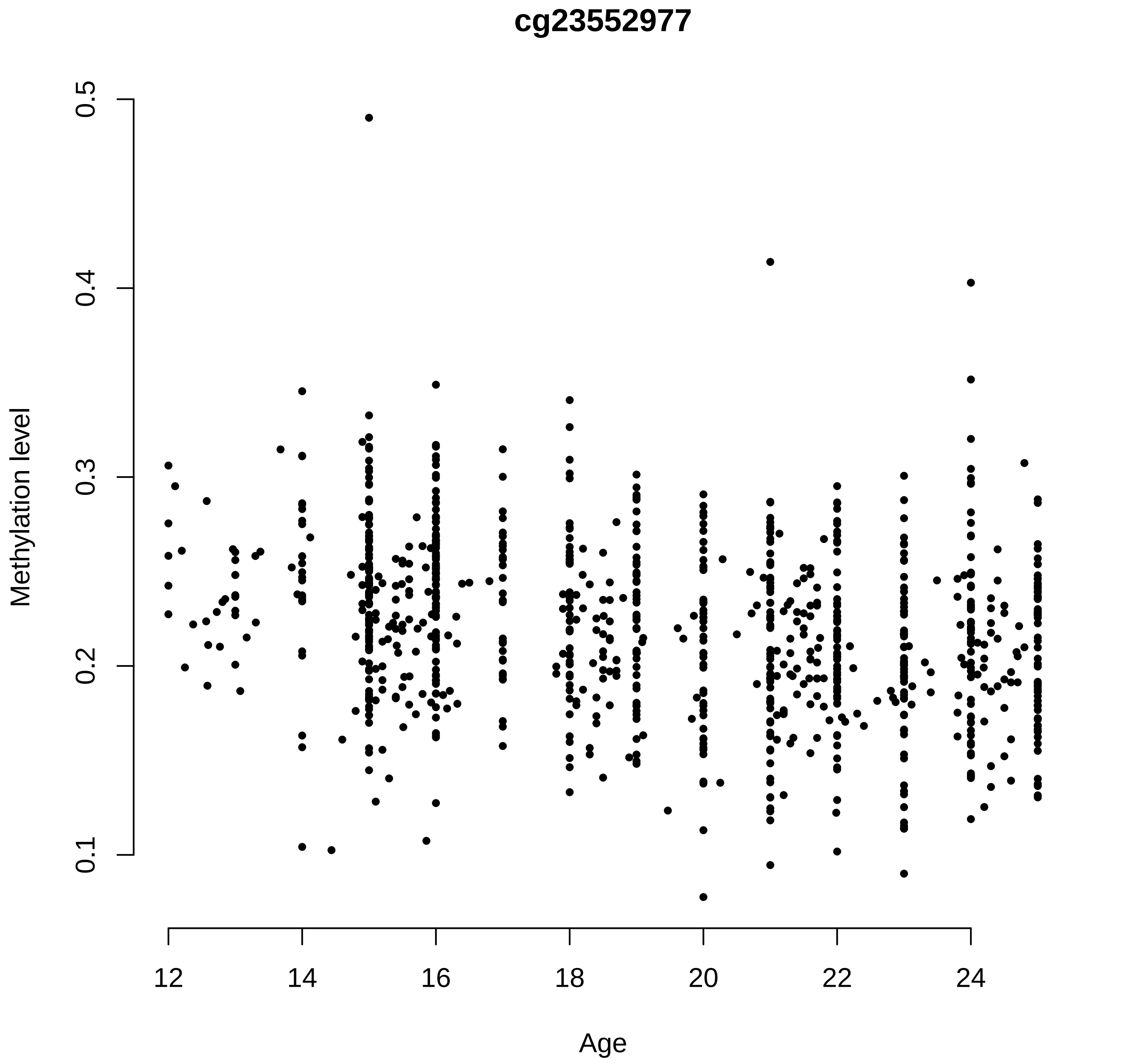
cg23552977
Methylation level
0.1
0.2
0.3
0.4
0.5
Age
12
14
16
18
20
22
24

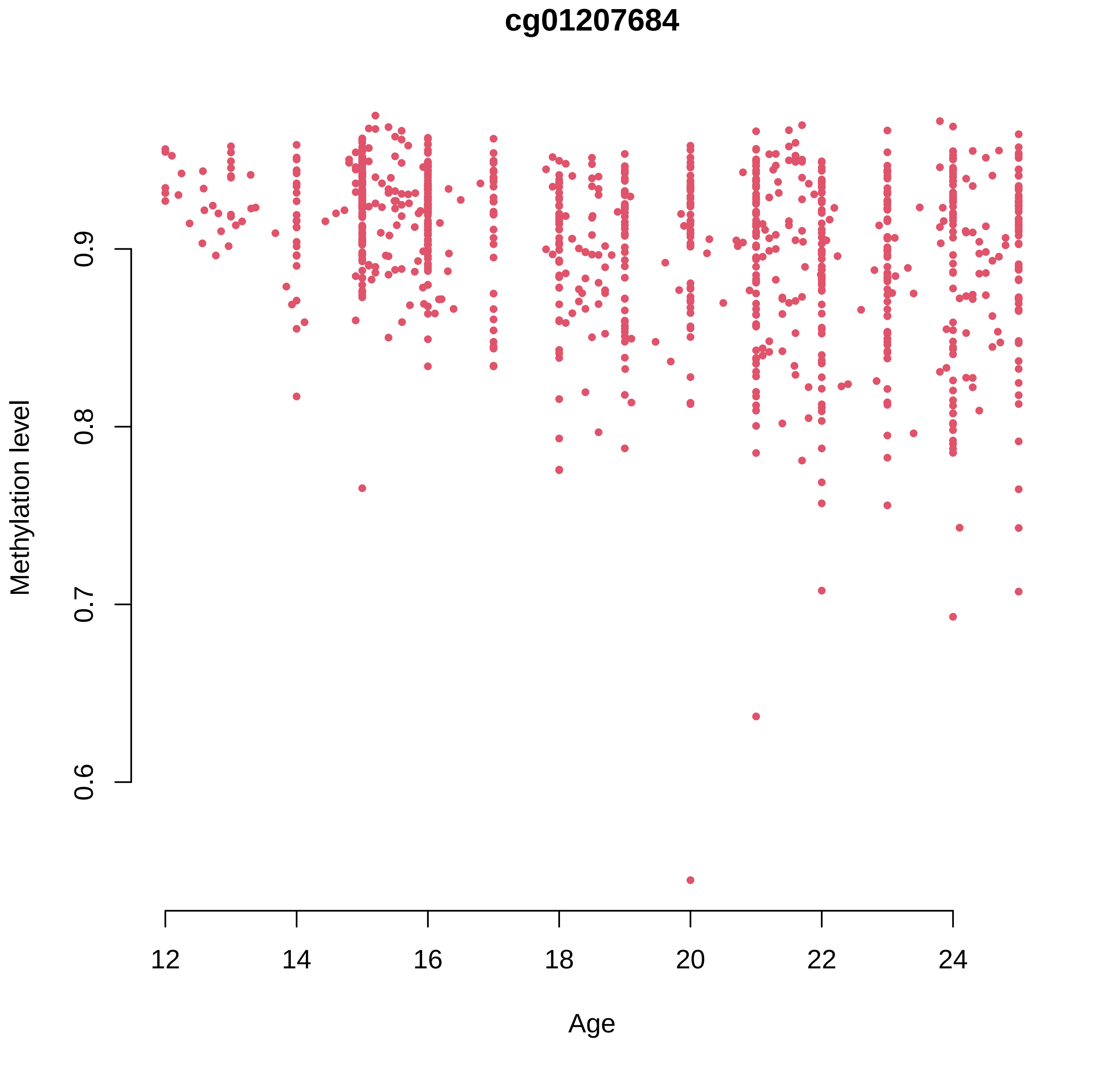
cg01207684
Methylation level
0.6
0.7
0.8
0.9
Age
12
14
16
18
20
22
24

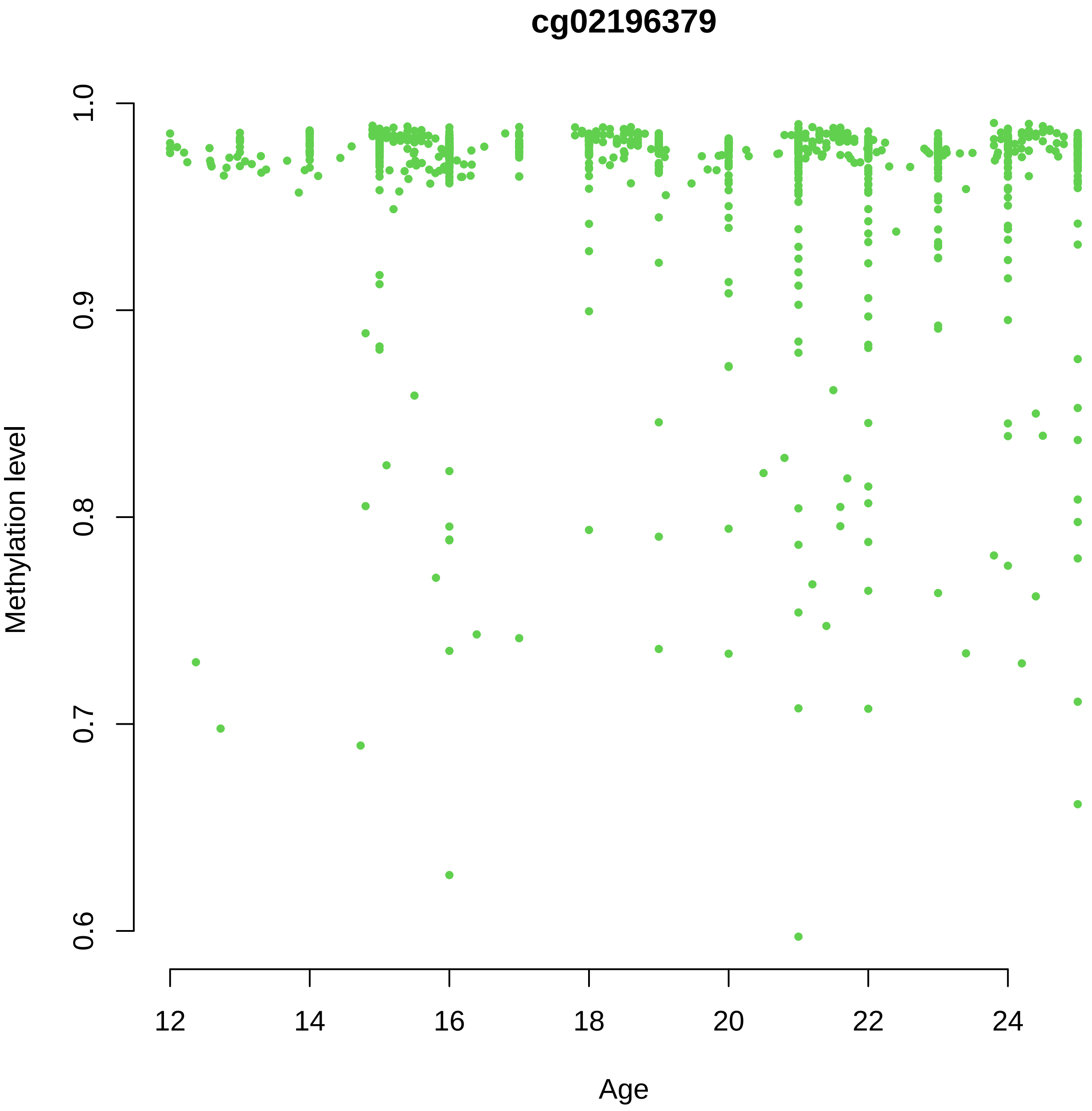
cg02196379
Methylation level
1.0
0.9
0.8
0.7
0.6
Age
12
14
16
18
20
22
24

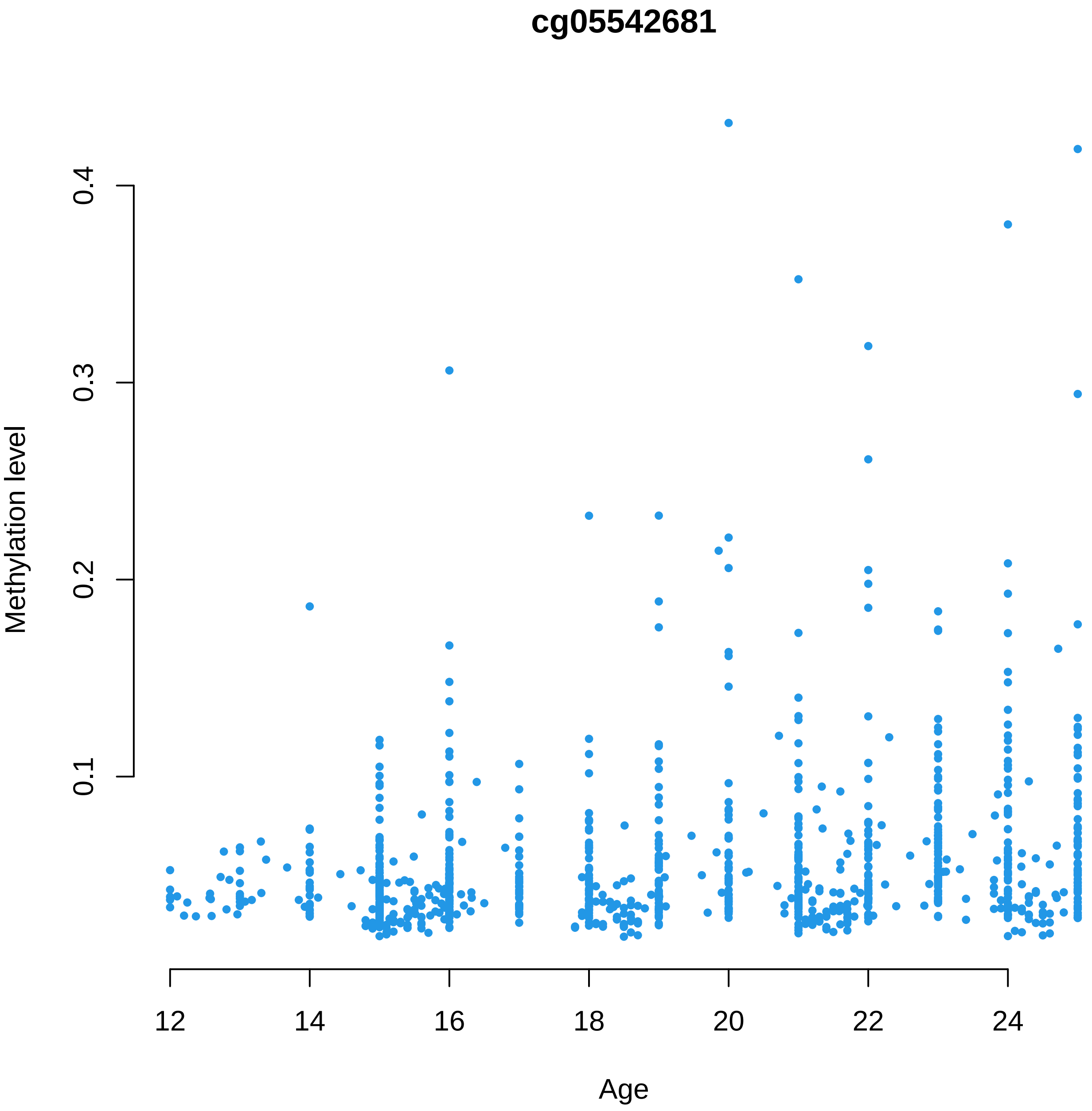
cg05542681
Methylation level
0.1
0.2
0.3
0.4
Age
12
14
16
18
20
22
24

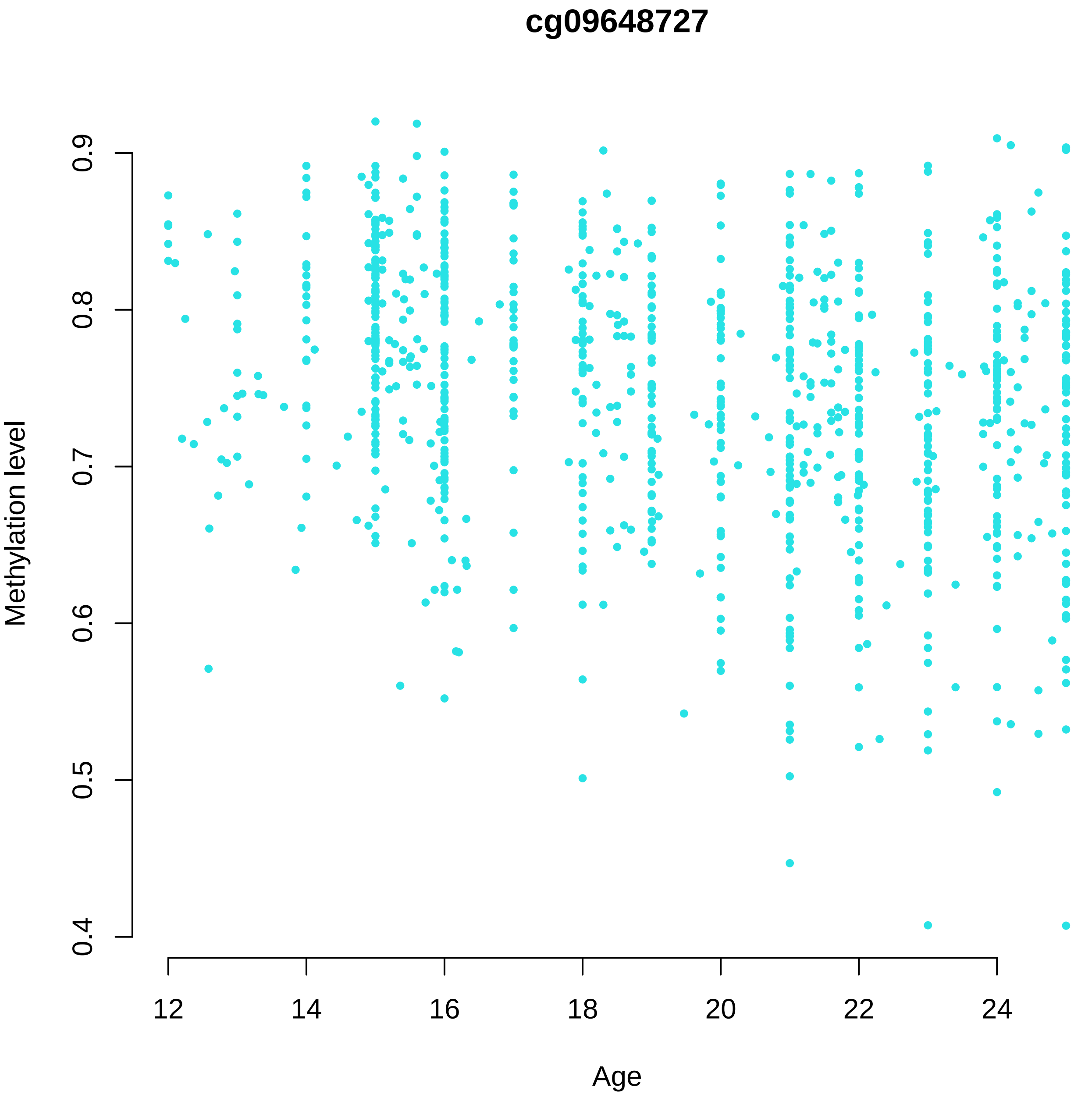

cg09648727
Methylation level
0.4
0.5
0.6
0.7
0.8
0.9
Age
12
14
16
18
20
22
24

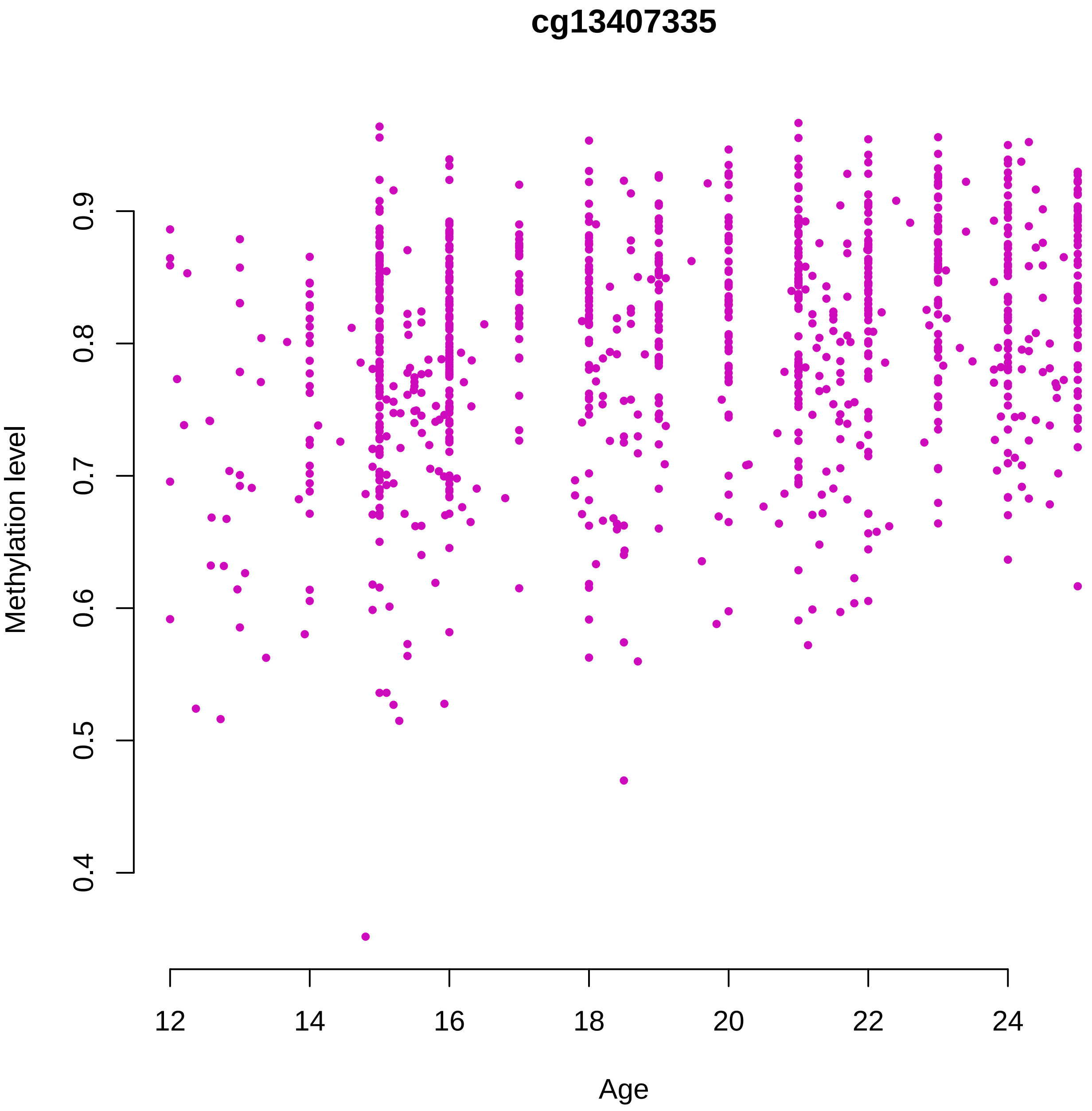
cg13407335
Methylation level
0.4
0.5
0.6
0.7
0.8
0.9
Age
12
14
16
18
20
22
24

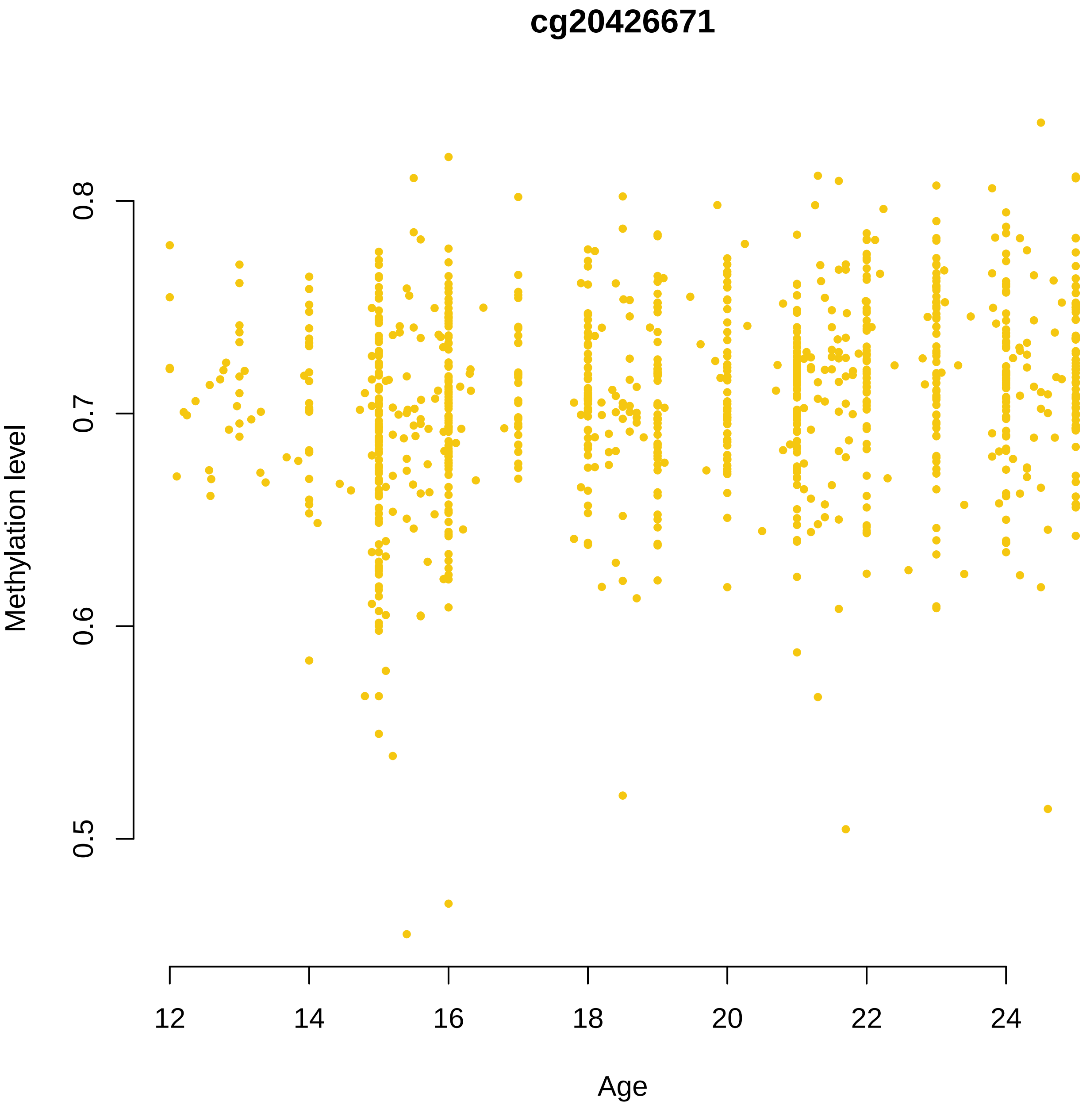
cg20426671
Methylation level
0.5
0.6
0.7
0.8
Age
12
14
16
18
20
22
24

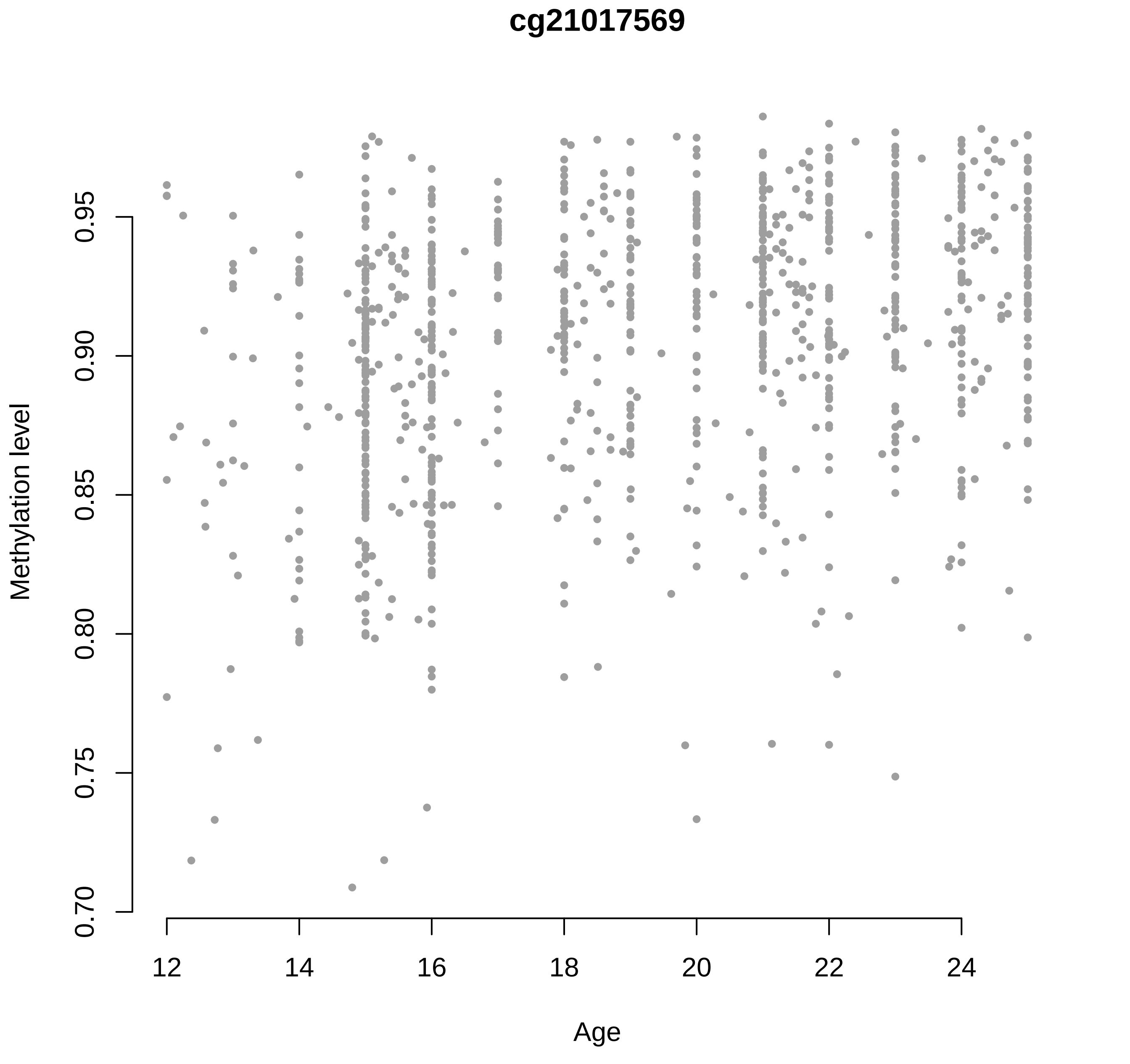

cg21017569
Methylation level
0.70
0.75
0.80
0.85
0.90
0.95
12
14
16
18
20
22
24
Age

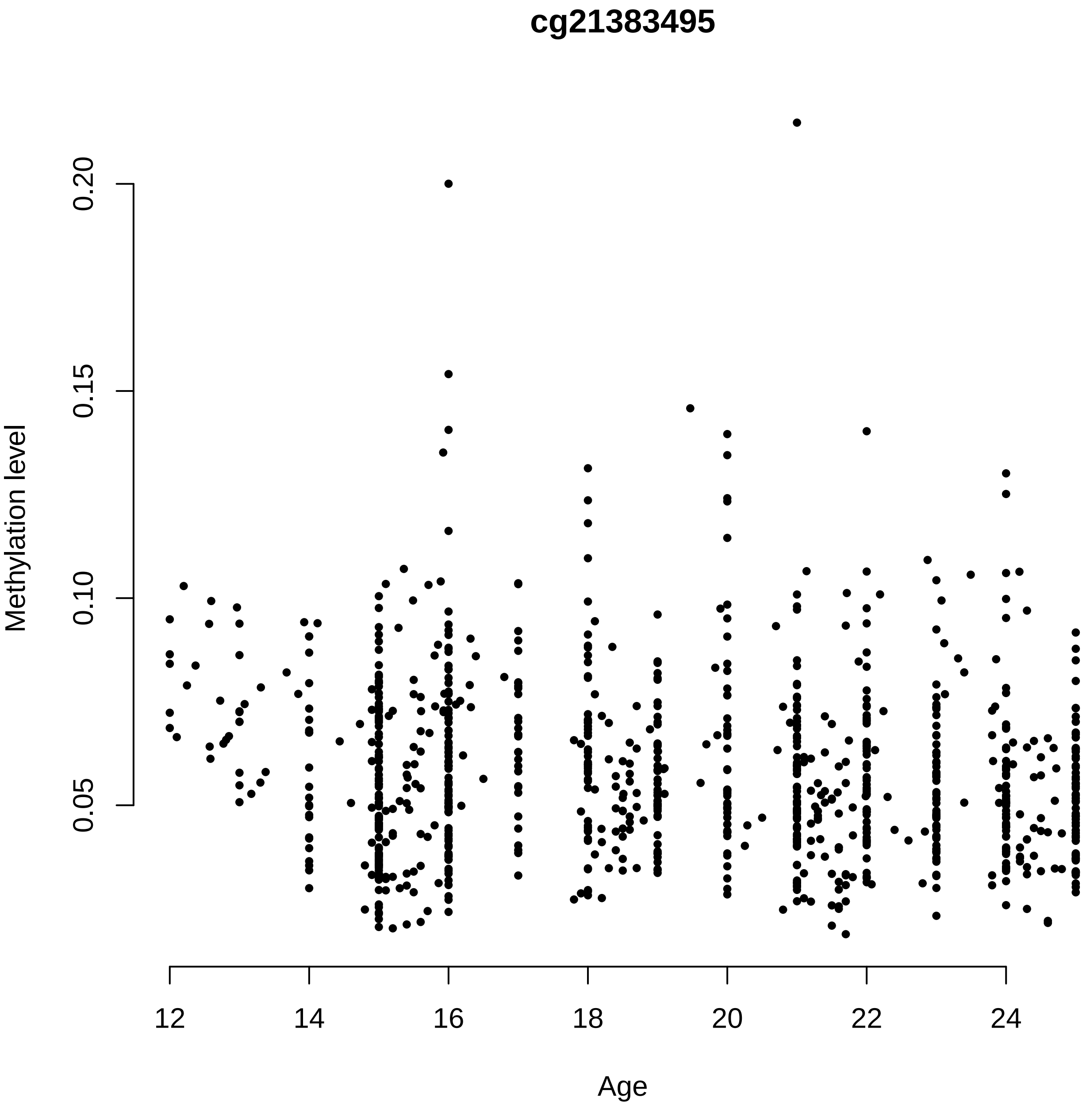

cg21383495
Methylation level
0.05
0.10
0.15
0.20
Age
12
14
16
18
20
22
24

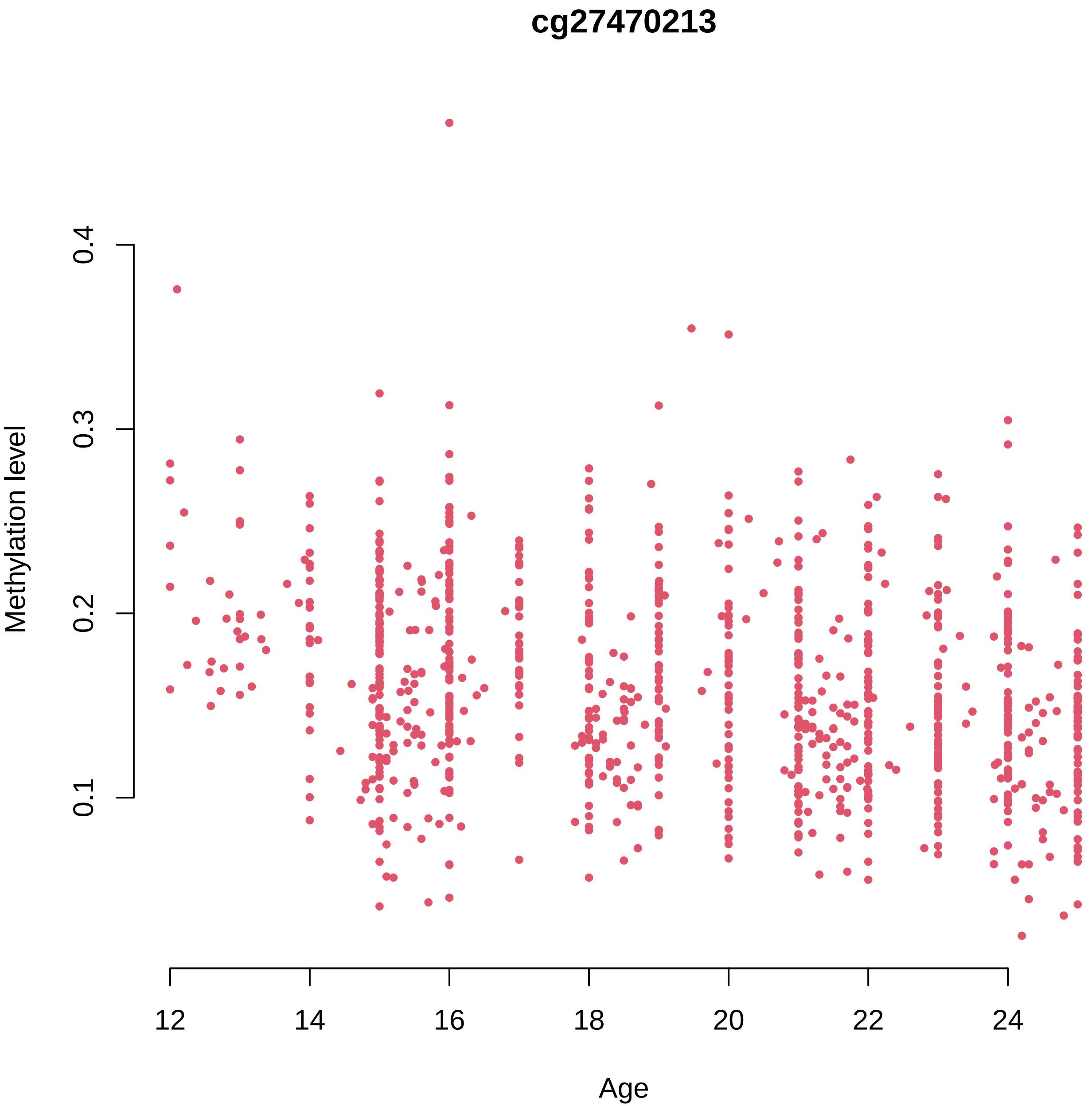

cg27470213
Methylation level
0.1
0.2
0.3
0.4
Age
12
14
16
18
20
22
24

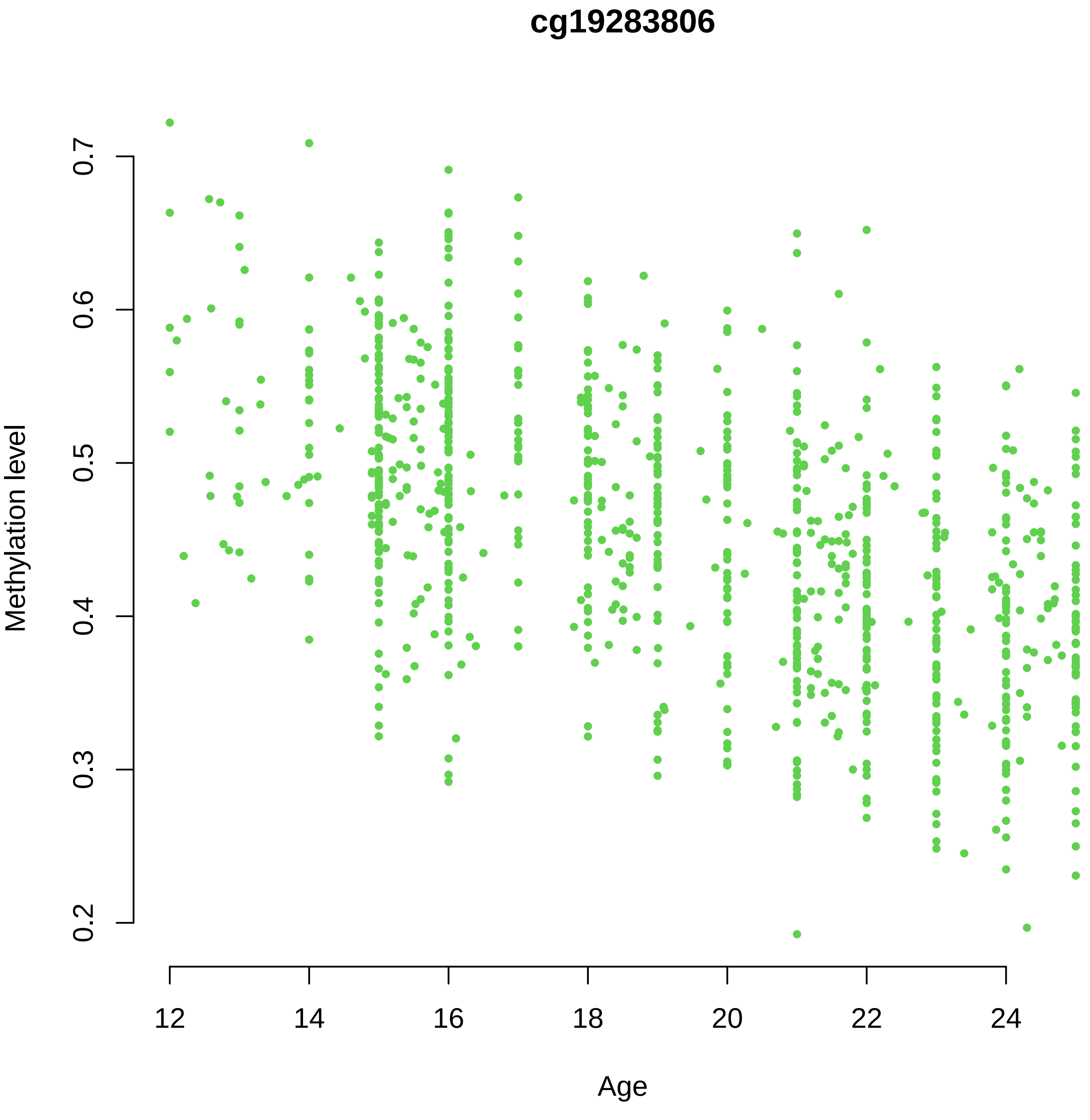
cg19283806
Methylation level
0.2
0.3
0.4
0.5
0.6
0.7
Age
12
14
16
18
20
22
24

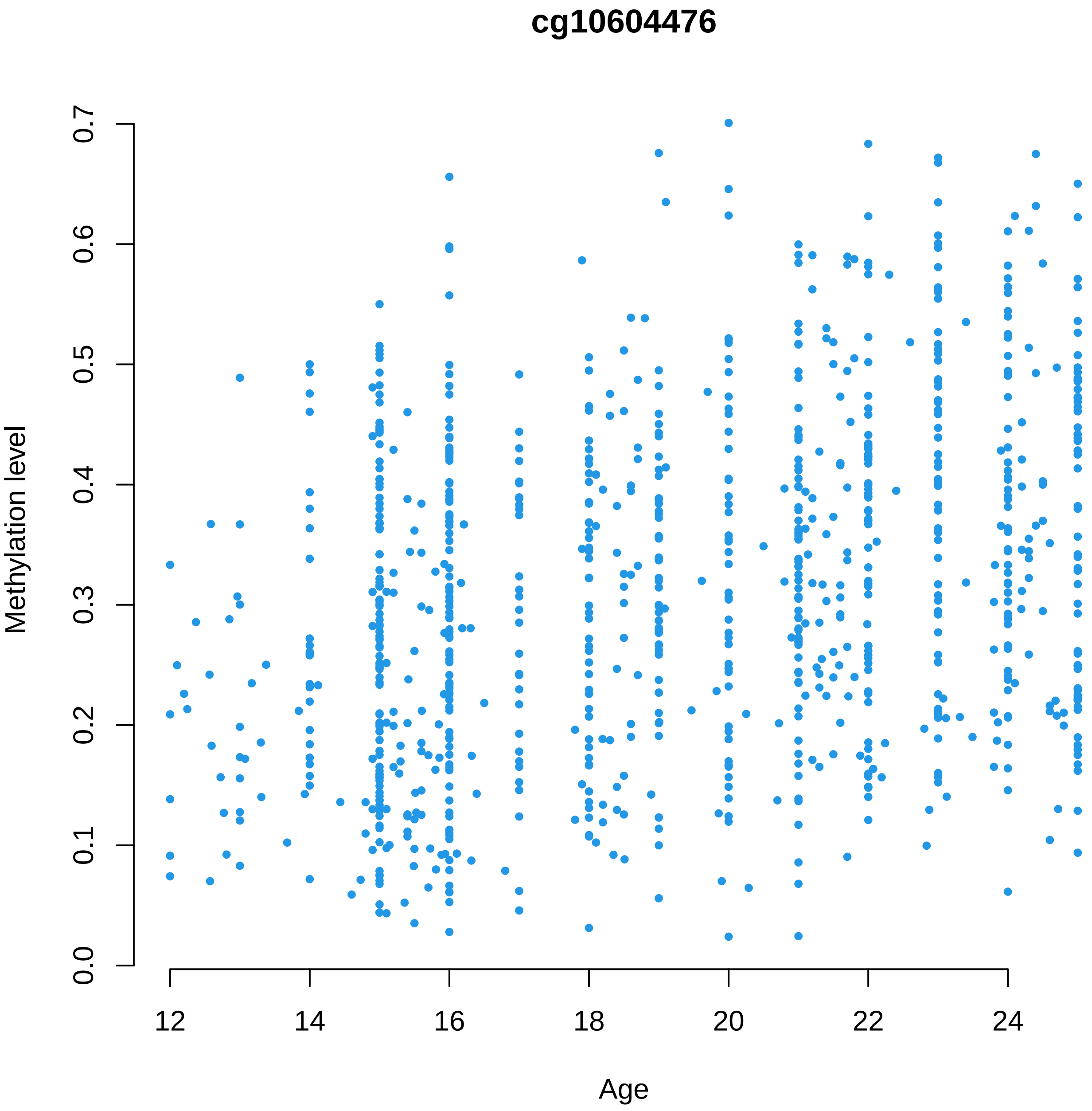
cg10604476
Methylation level
0.0
0.1
0.2
0.3
0.4
0.5
0.6
0.7
Age
12
14
16
18
20
22
24

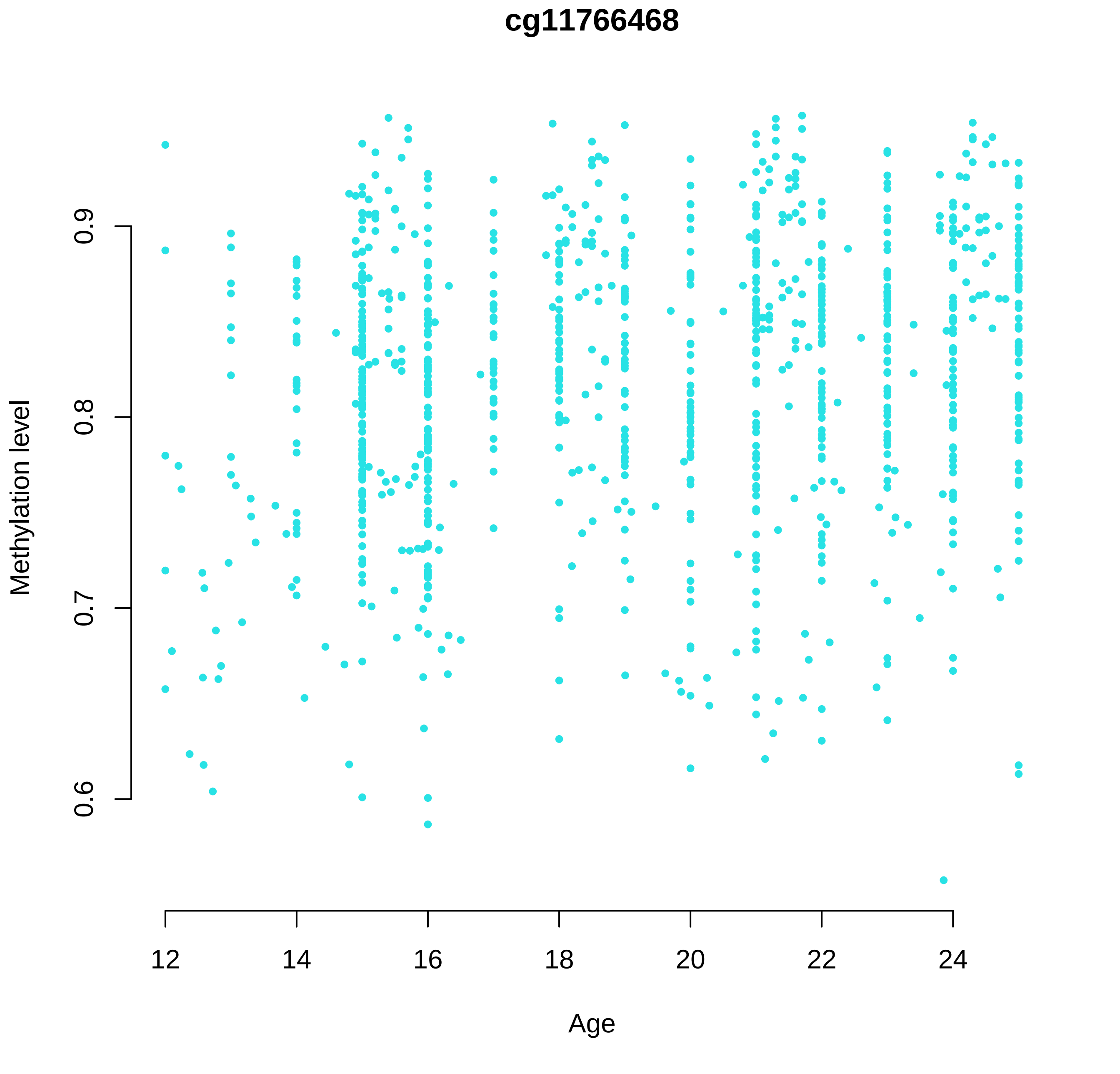
cg11766468
Methylation level
0.6
0.7
0.8
0.9
12
14
16
18
20
22
24
Age

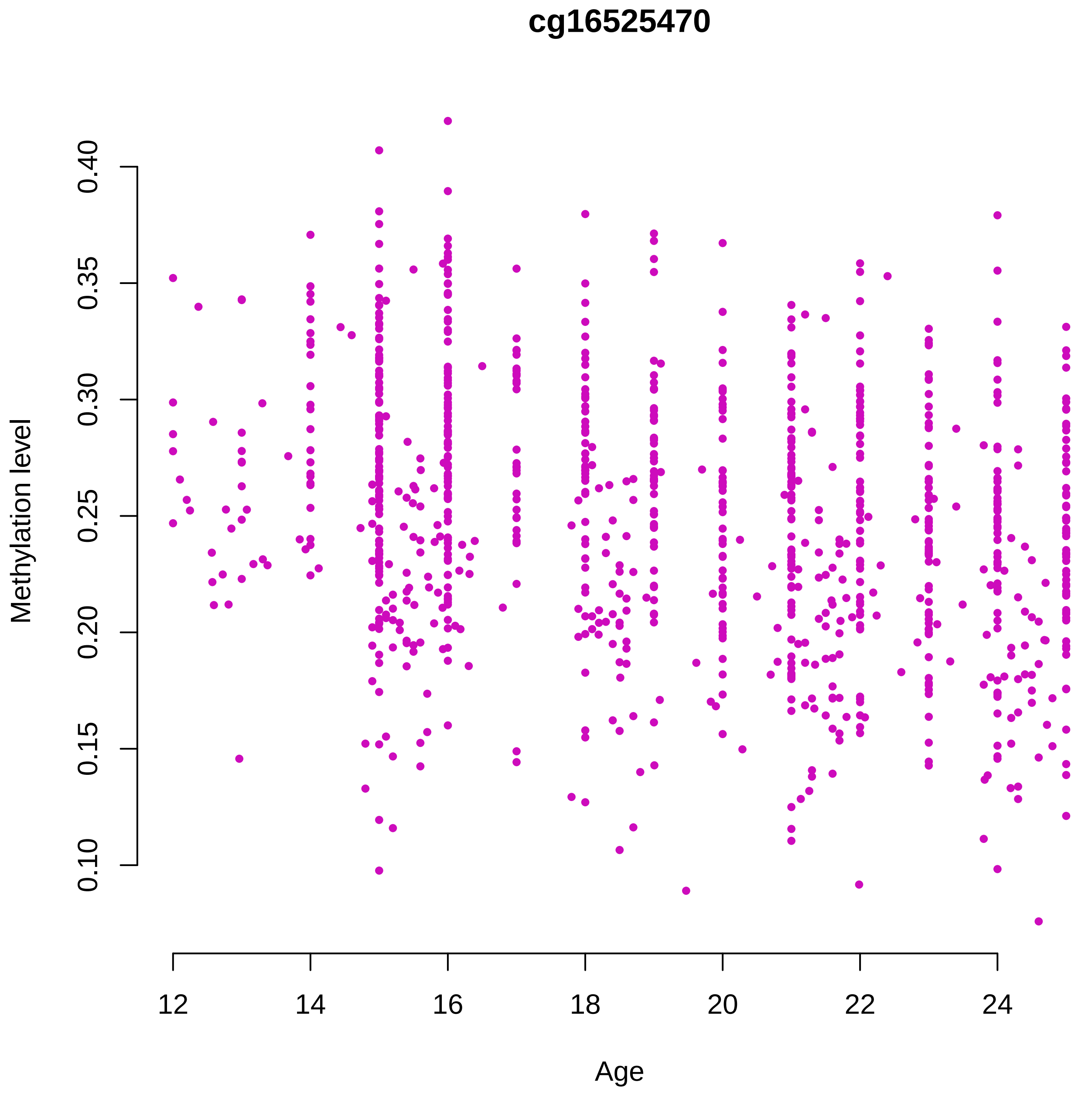

cg16525470
Methylation level
0.10
0.15
0.20
0.25
0.30
0.35
0.40
12
14
16
18
20
22
24
Age

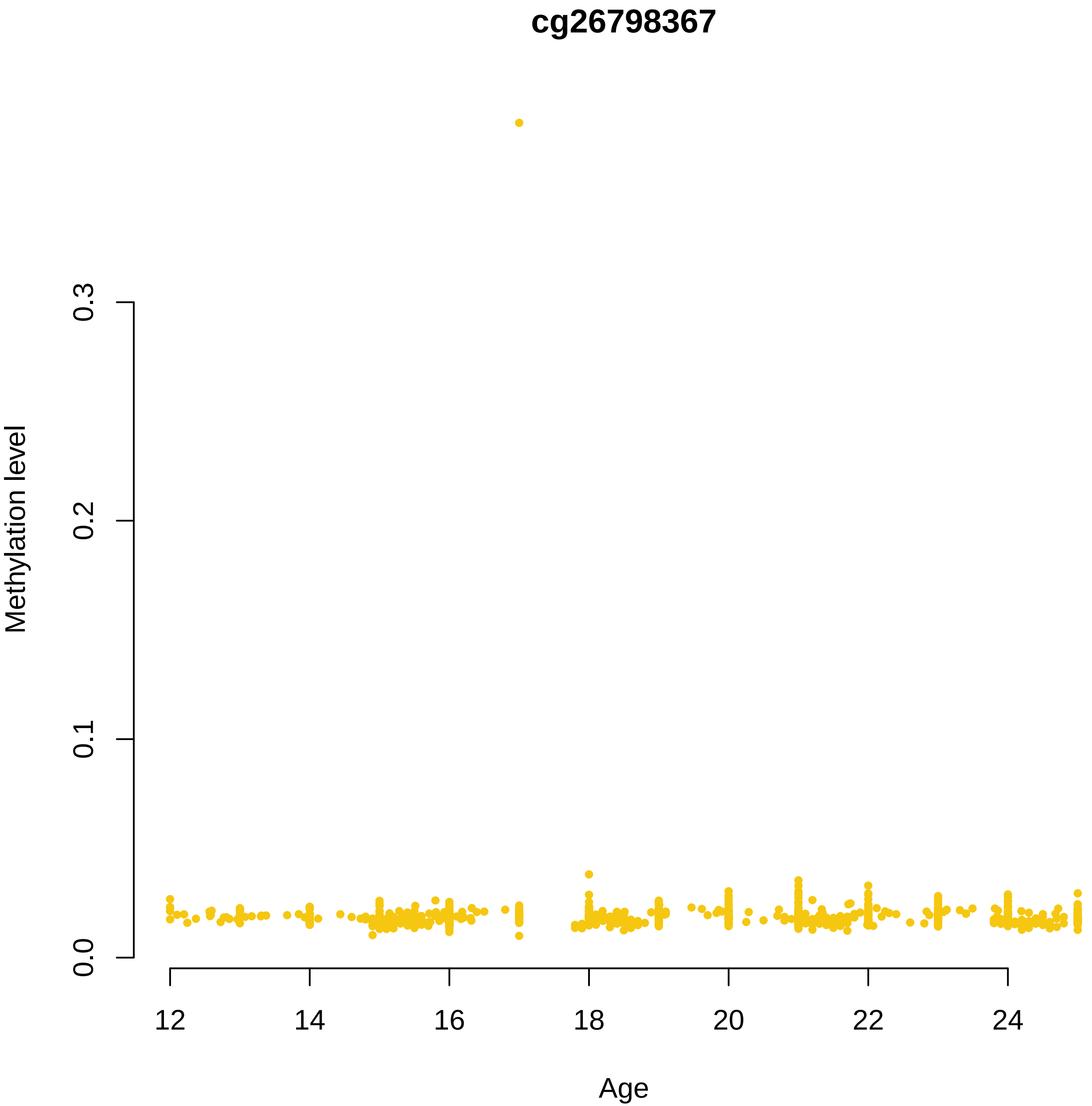
cg26798367
Methylation level
0.0
0.1
0.2
0.3
Age
12
14
16
18
20
22
24

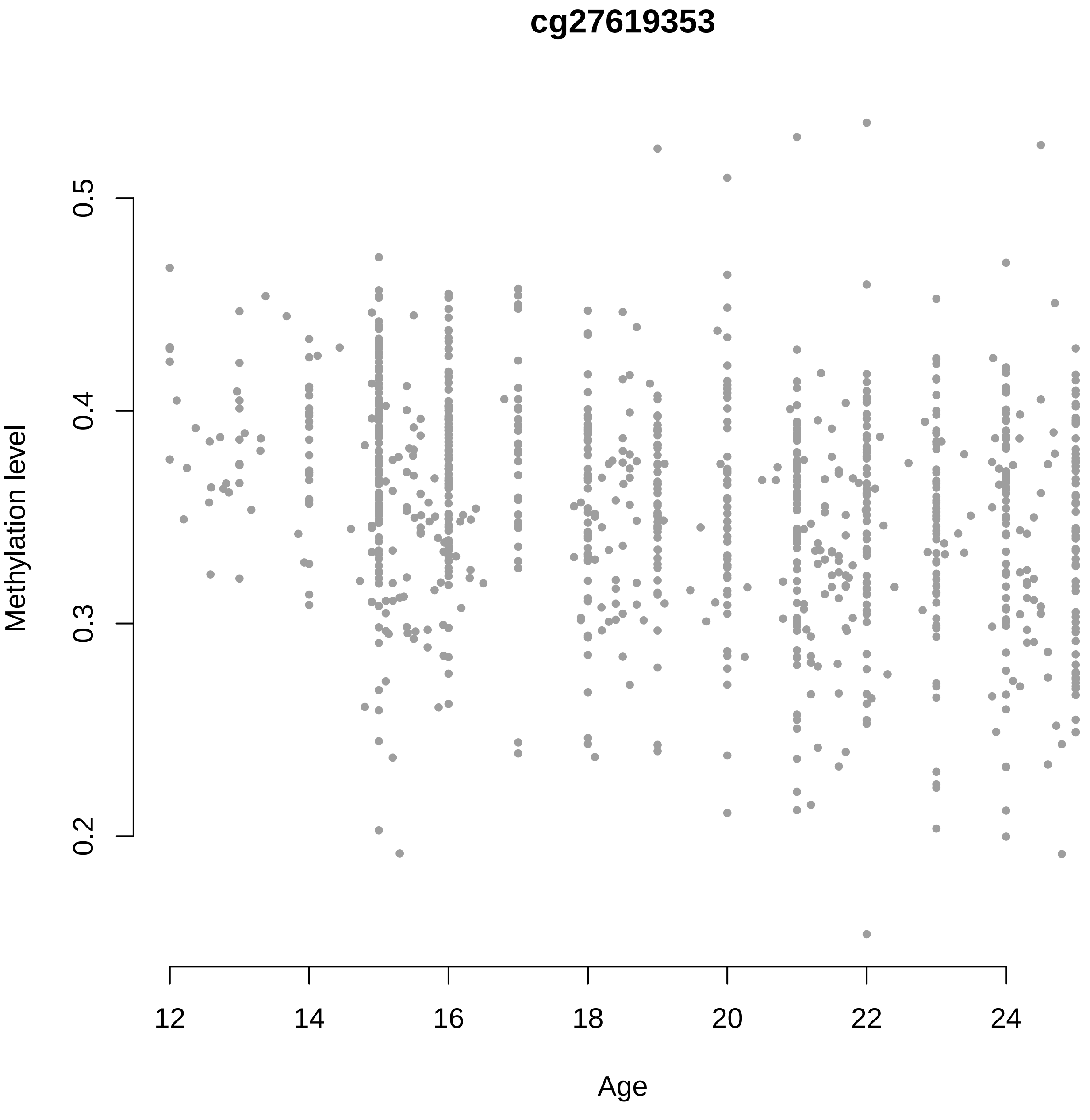

cg27619353
Methylation level
0.2
0.3
0.4
0.5
Age
12
14
16
18
20
22
24

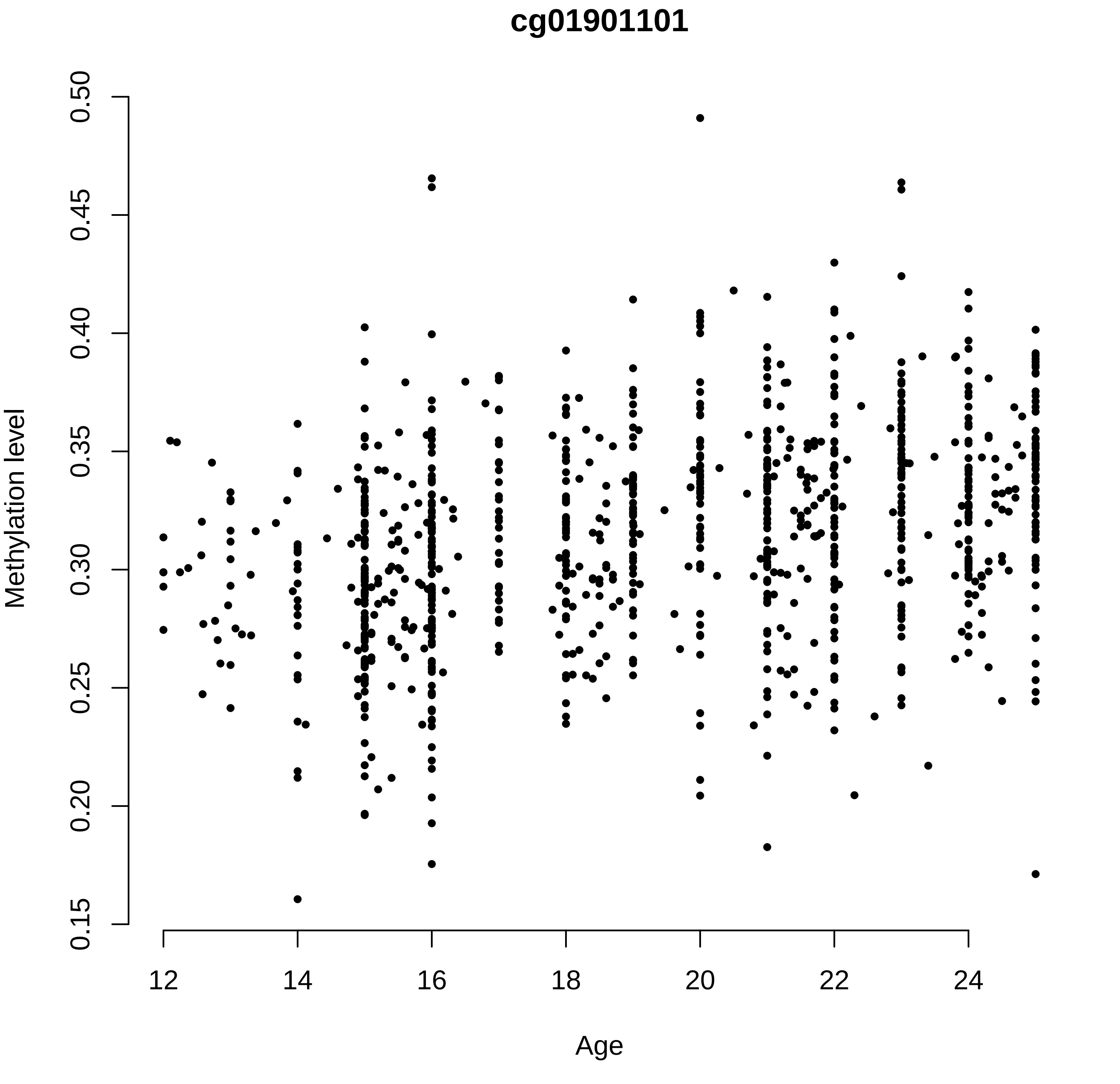
cg01901101
Methylation level
0.15
0.20
0.25
0.30
0.35
0.40
0.45
0.50
Age
12
14
16
18
20
22
24

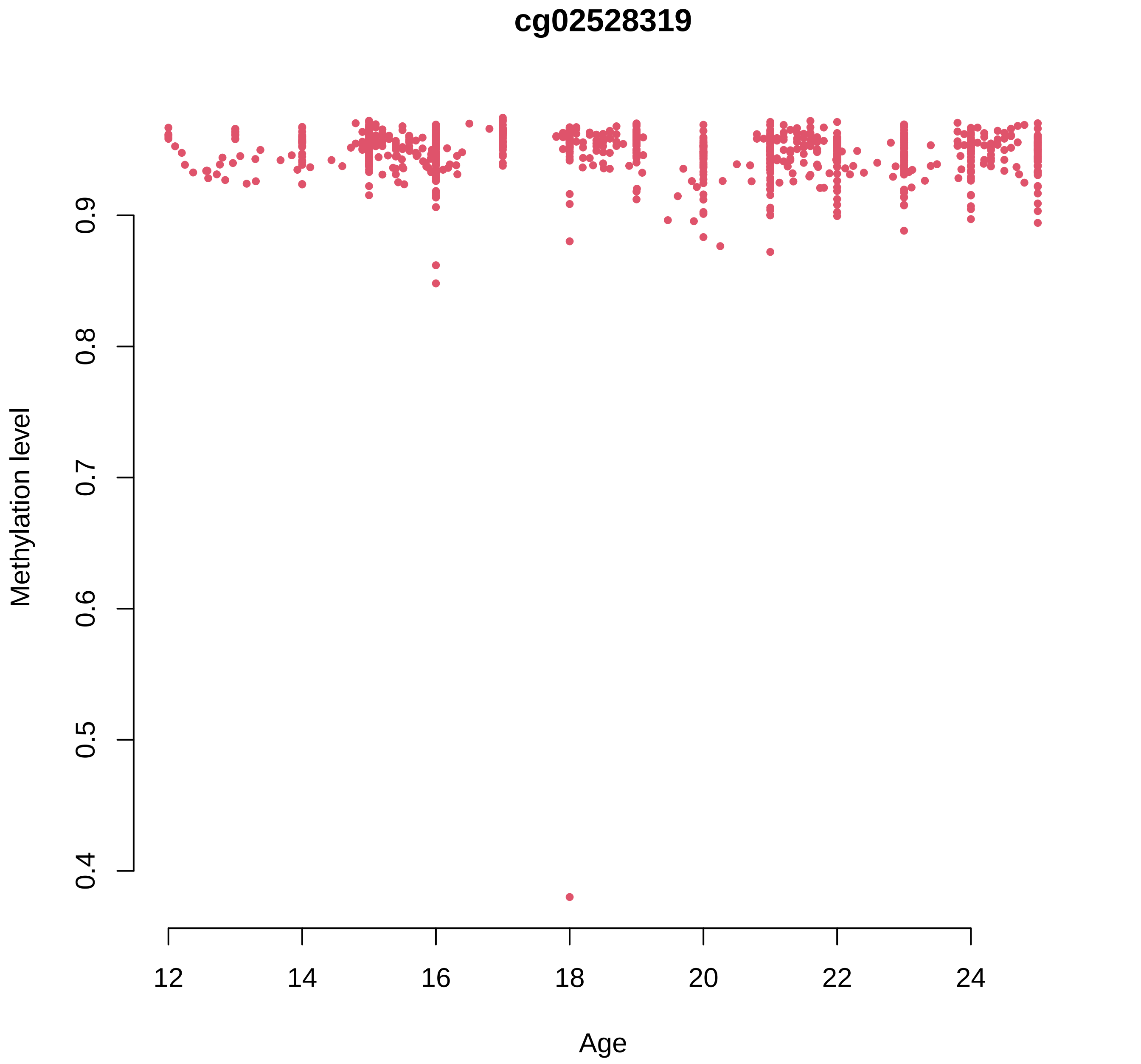

cg02528319
Methylation level
0.4
0.5
0.6
0.7
0.8
0.9
12
14
16
18
20
22
24
Age

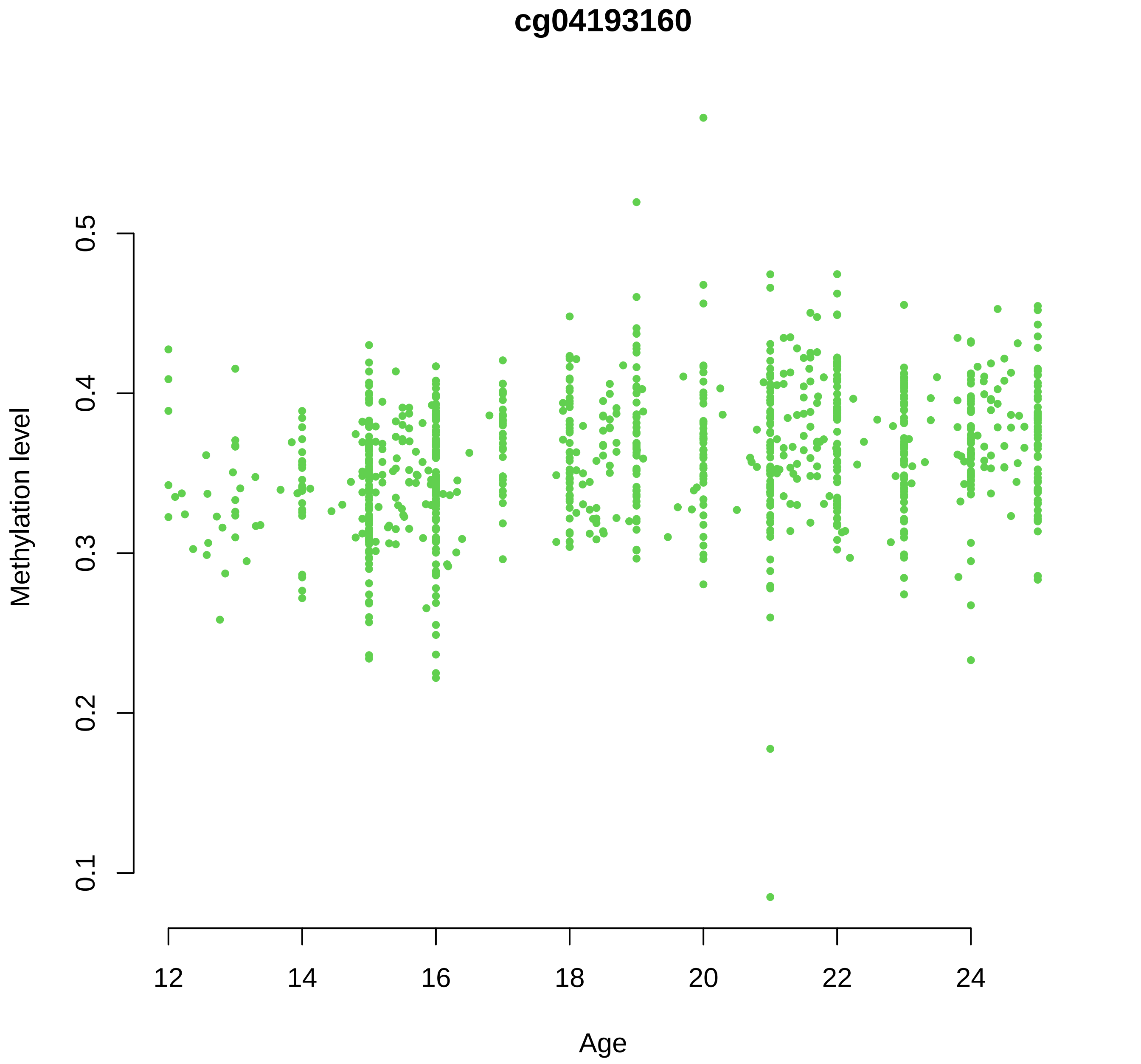

cg04193160
Methylation level
0.1
0.2
0.3
0.4
0.5
Age
12
14
16
18
20
22
24

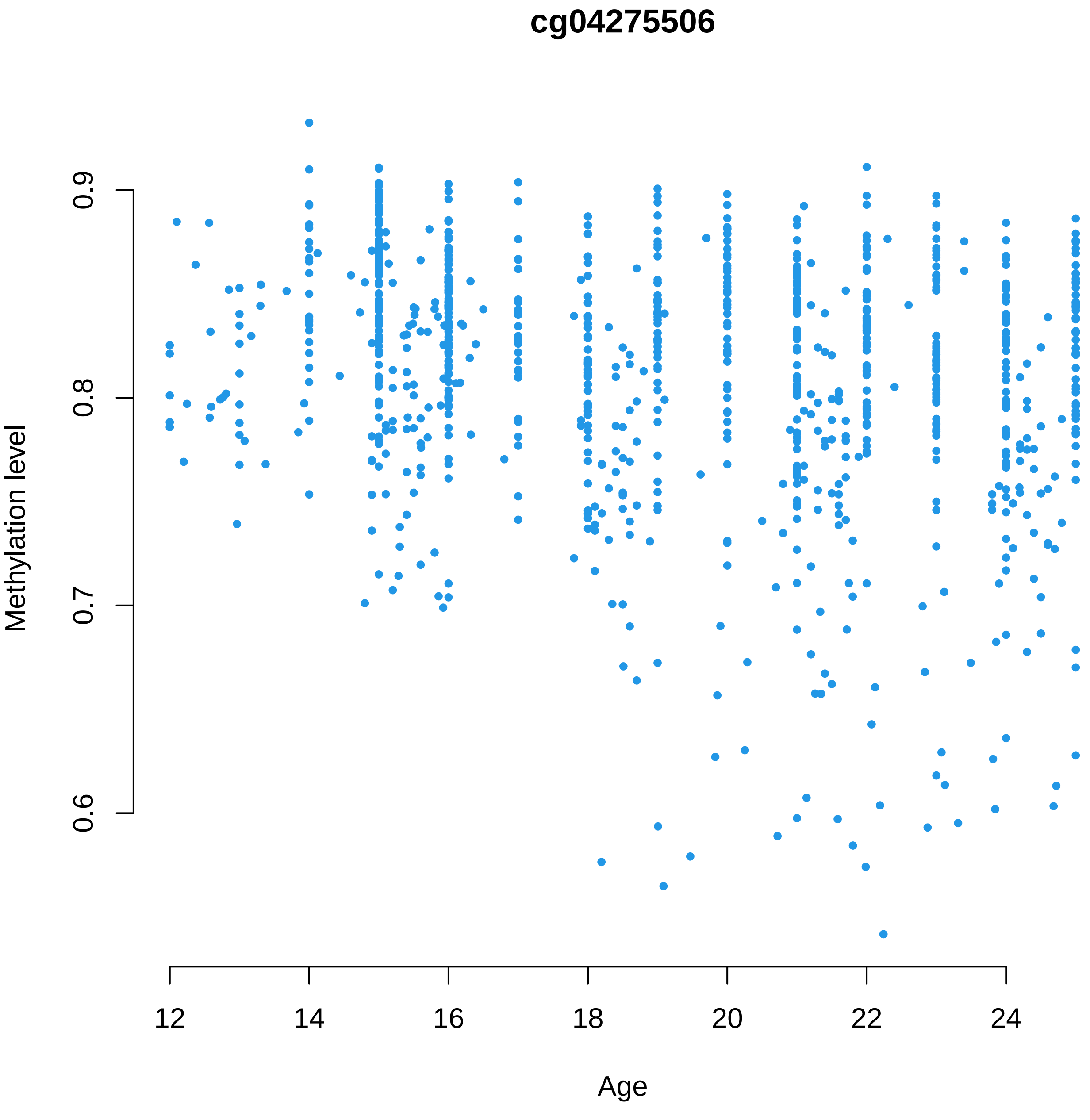
cg04275506
Methylation level
0.6
0.7
0.8
0.9
Age
12
14
16
18
20
22
24

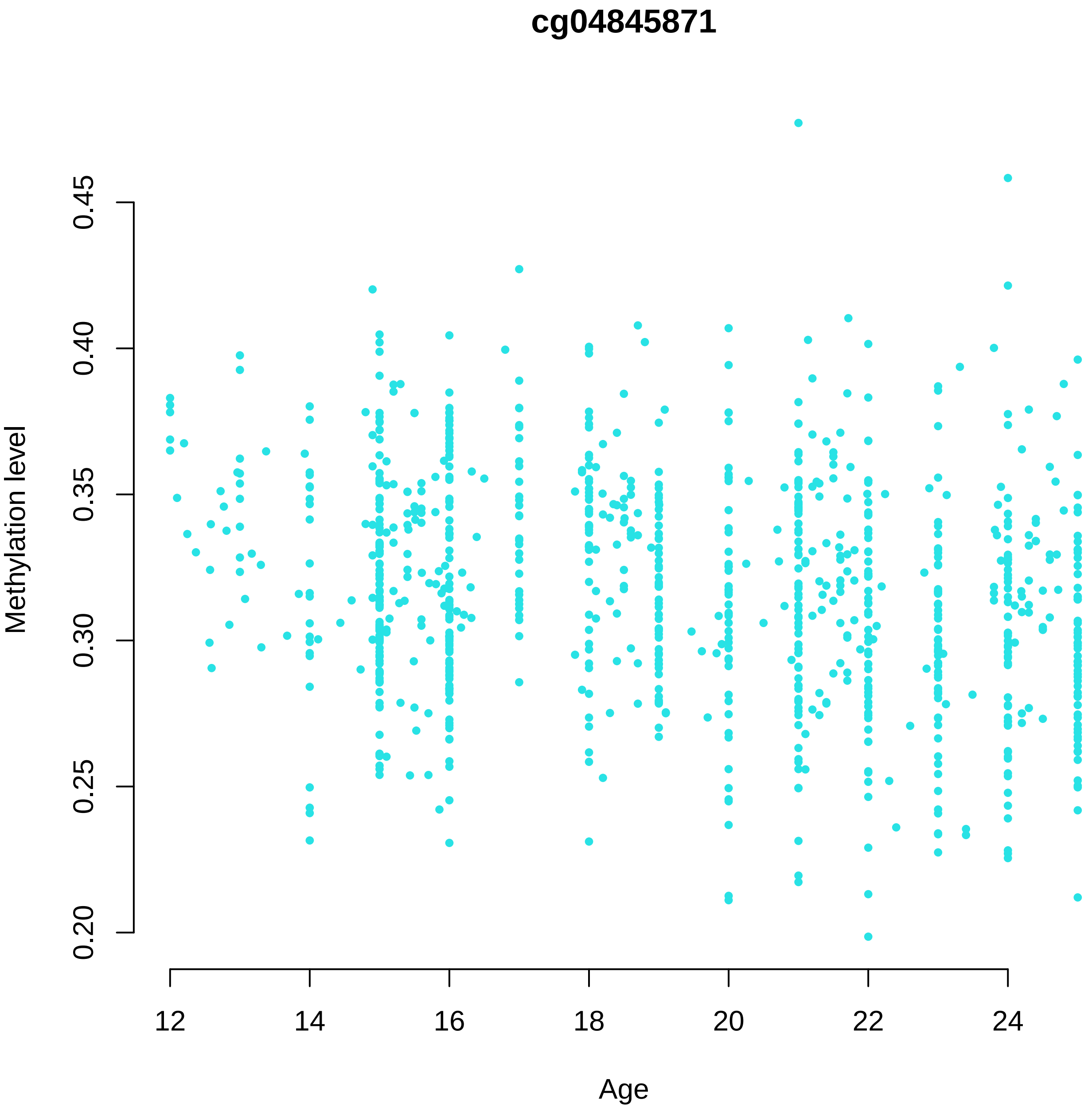
cg04845871
Methylation level
0.20
0.25
0.30
0.35
0.40
0.45
Age
12
14
16
18
20
22
24

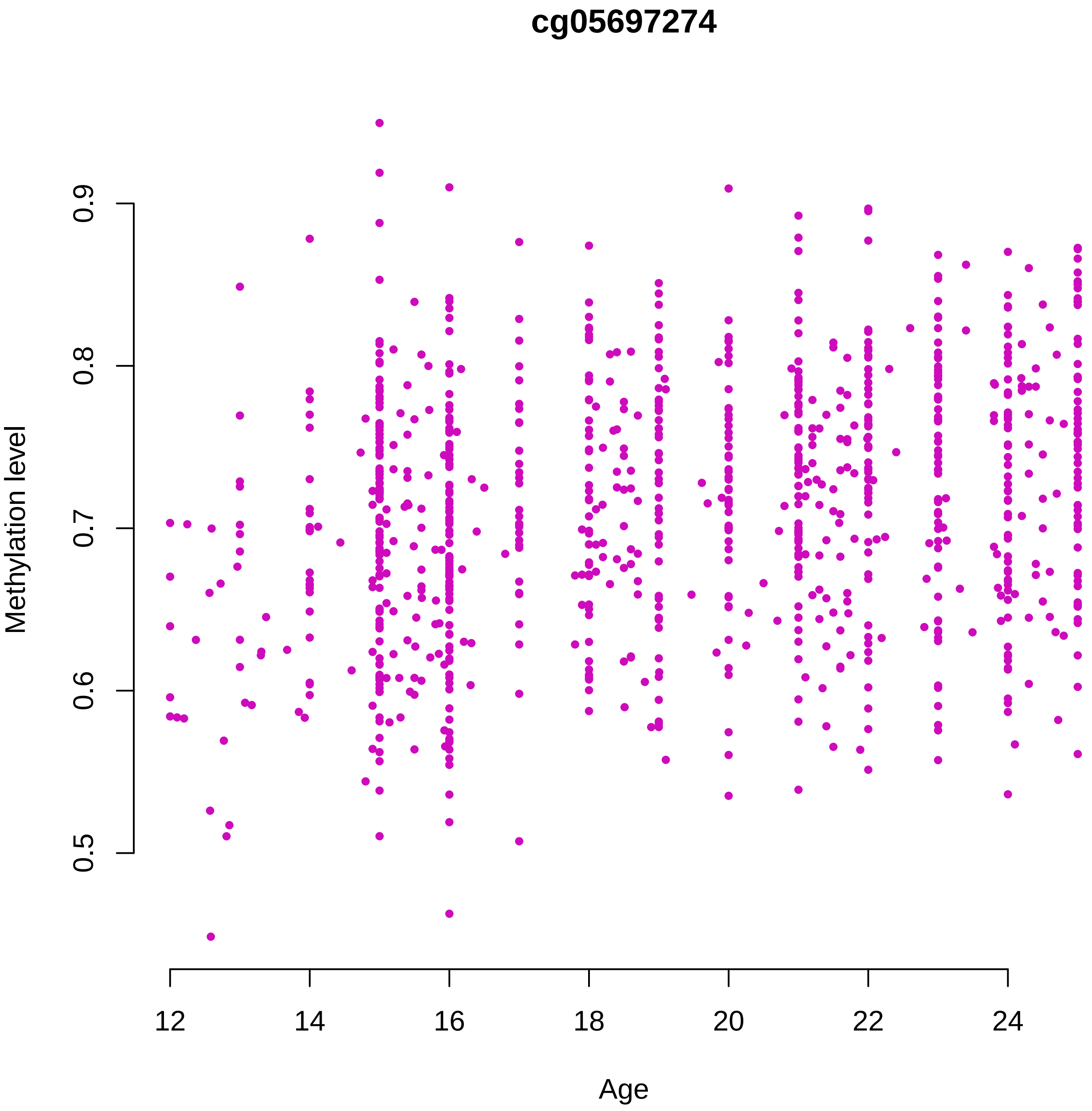
cg05697274
Methylation level
0.5
0.6
0.7
0.8
0.9
Age
12
14
16
18
20
22
24

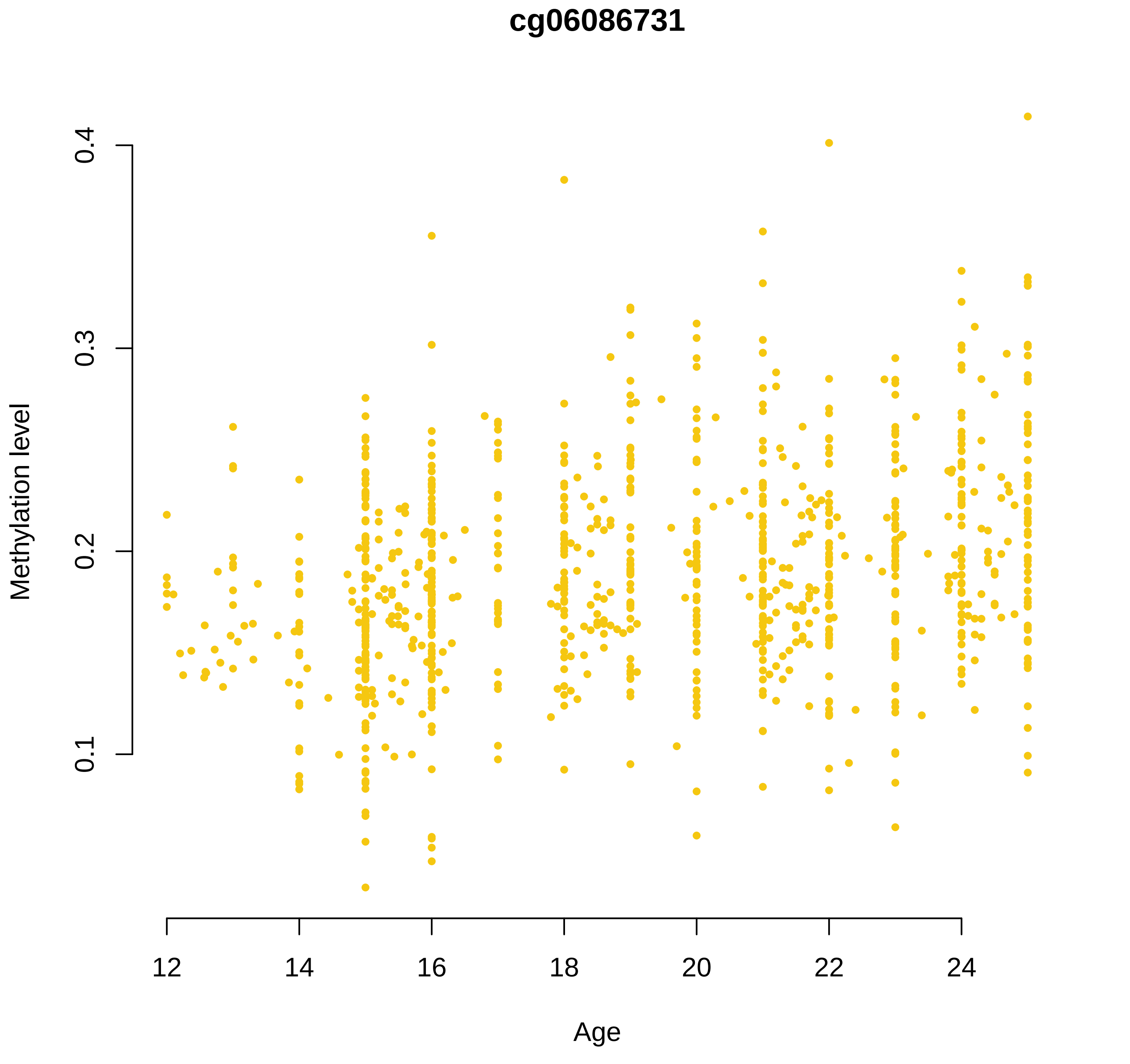

cg06086731
Methylation level
0.1
0.2
0.3
0.4
12
14
16
18
20
22
24
Age

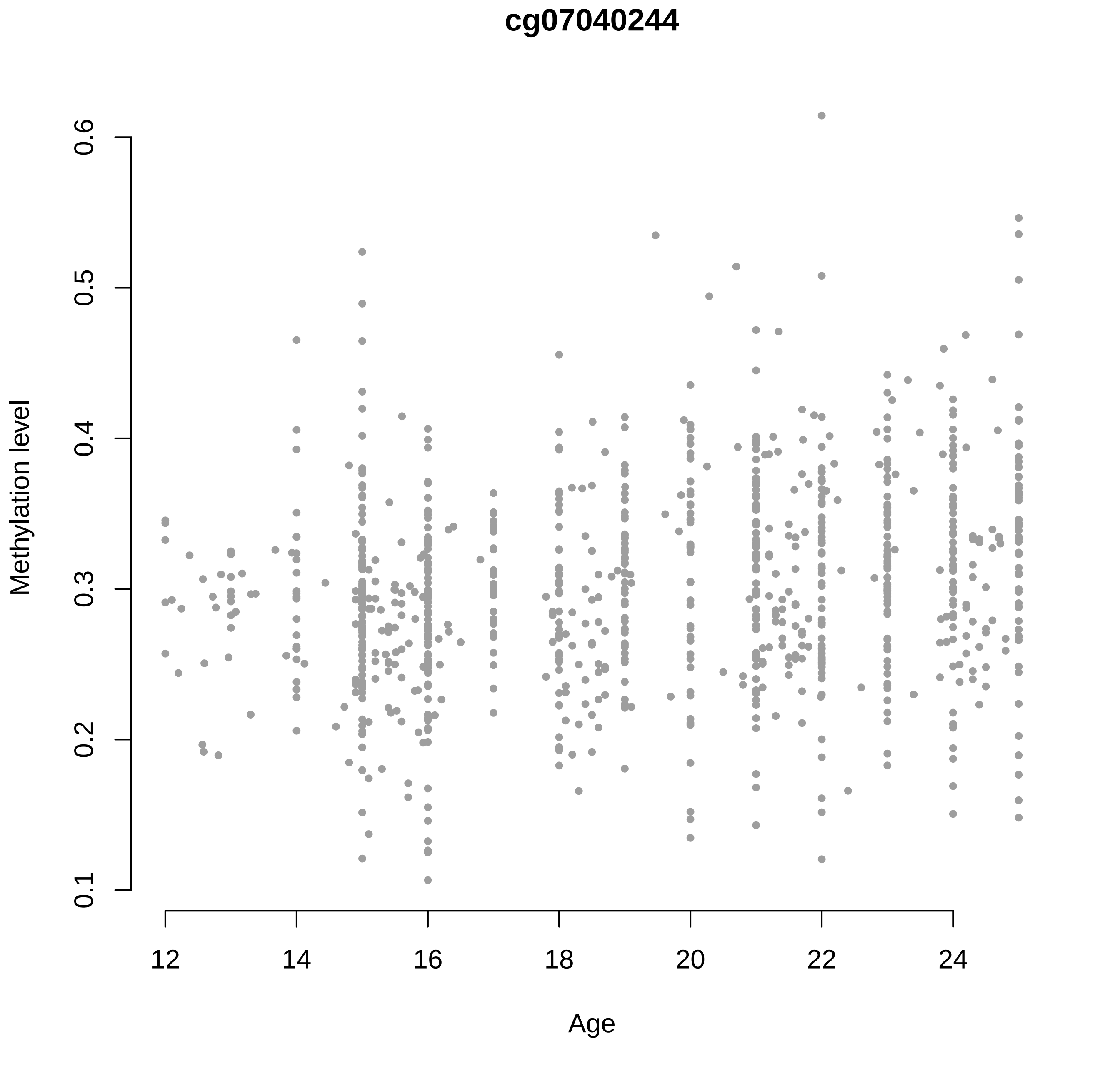
cg07040244
Methylation level
0.1
0.2
0.3
0.4
0.5
0.6
Age
12
14
16
18
20
22
24

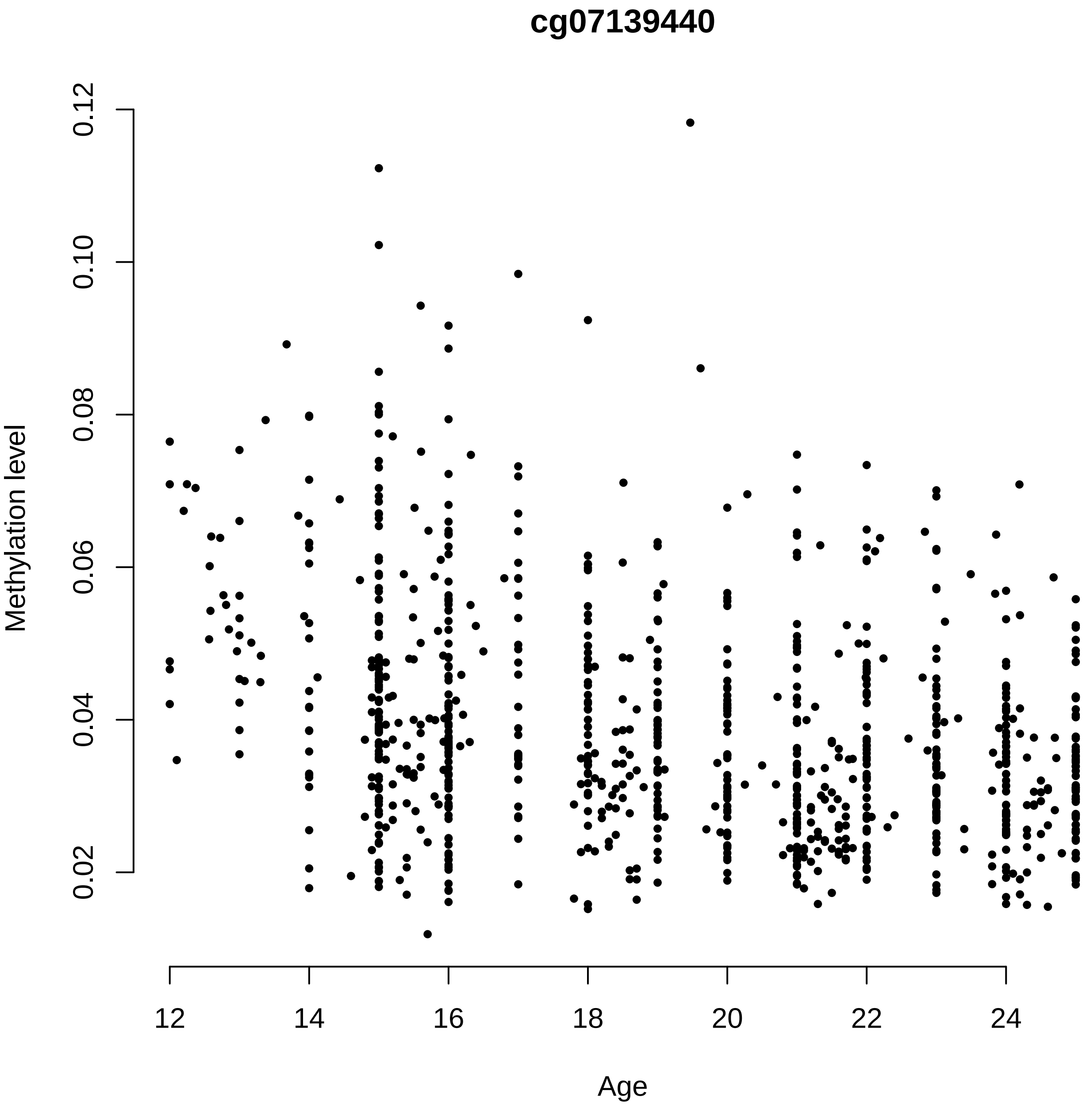
cg07139440
Methylation level
0.02
0.04
0.06
0.08
0.10
0.12
Age
12
14
16
18
20
22
24

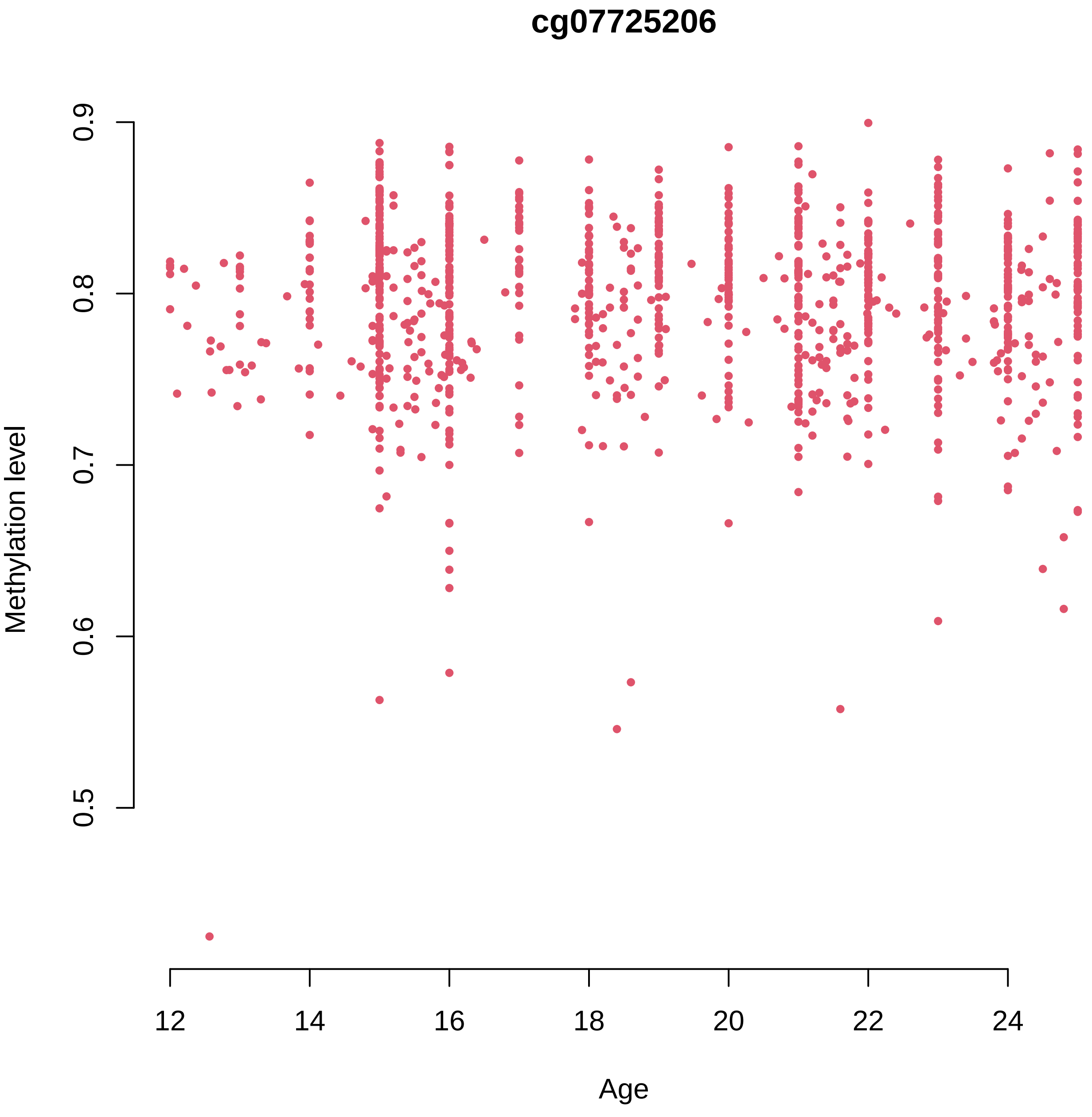
cg07725206
Methylation level
0.5
0.6
0.7
0.8
0.9
Age
12
14
16
18
20
22
24

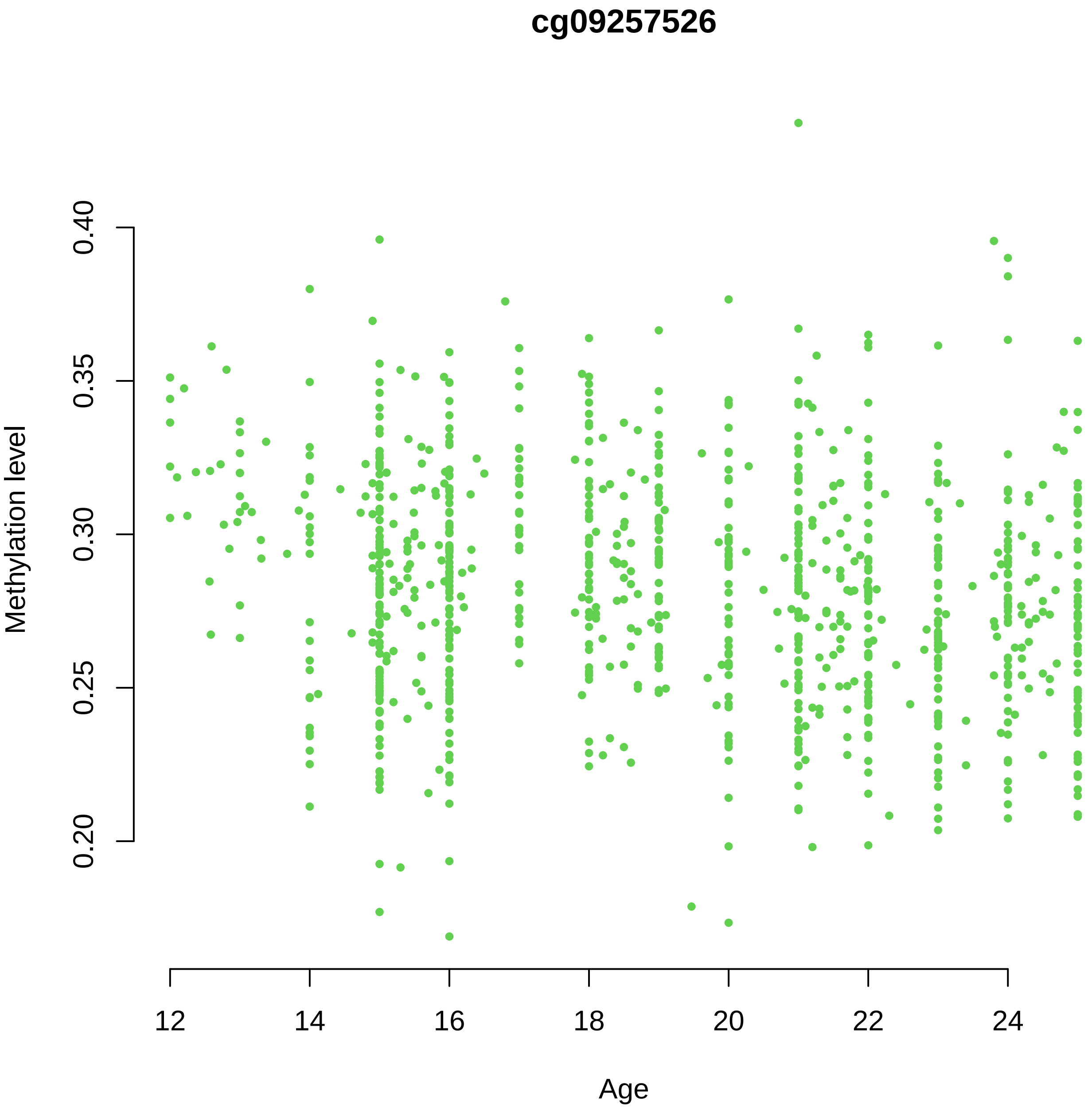
cg09257526
Methylation level
0.20
0.25
0.30
0.35
0.40
Age
12
14
16
18
20
22
24

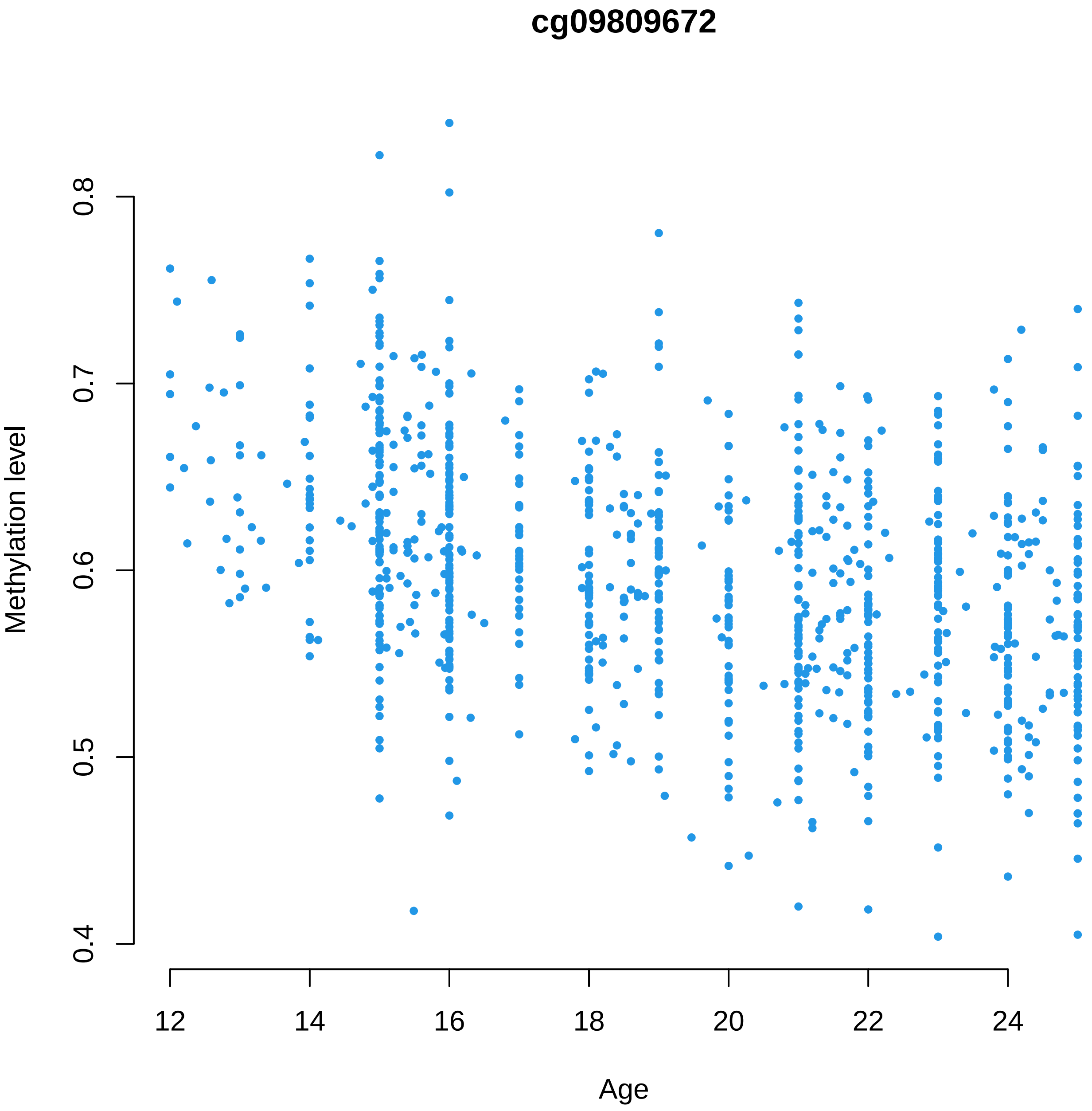
cg09809672
Methylation level
0.4
0.5
0.6
0.7
0.8
12
14
16
18
20
22
24
Age

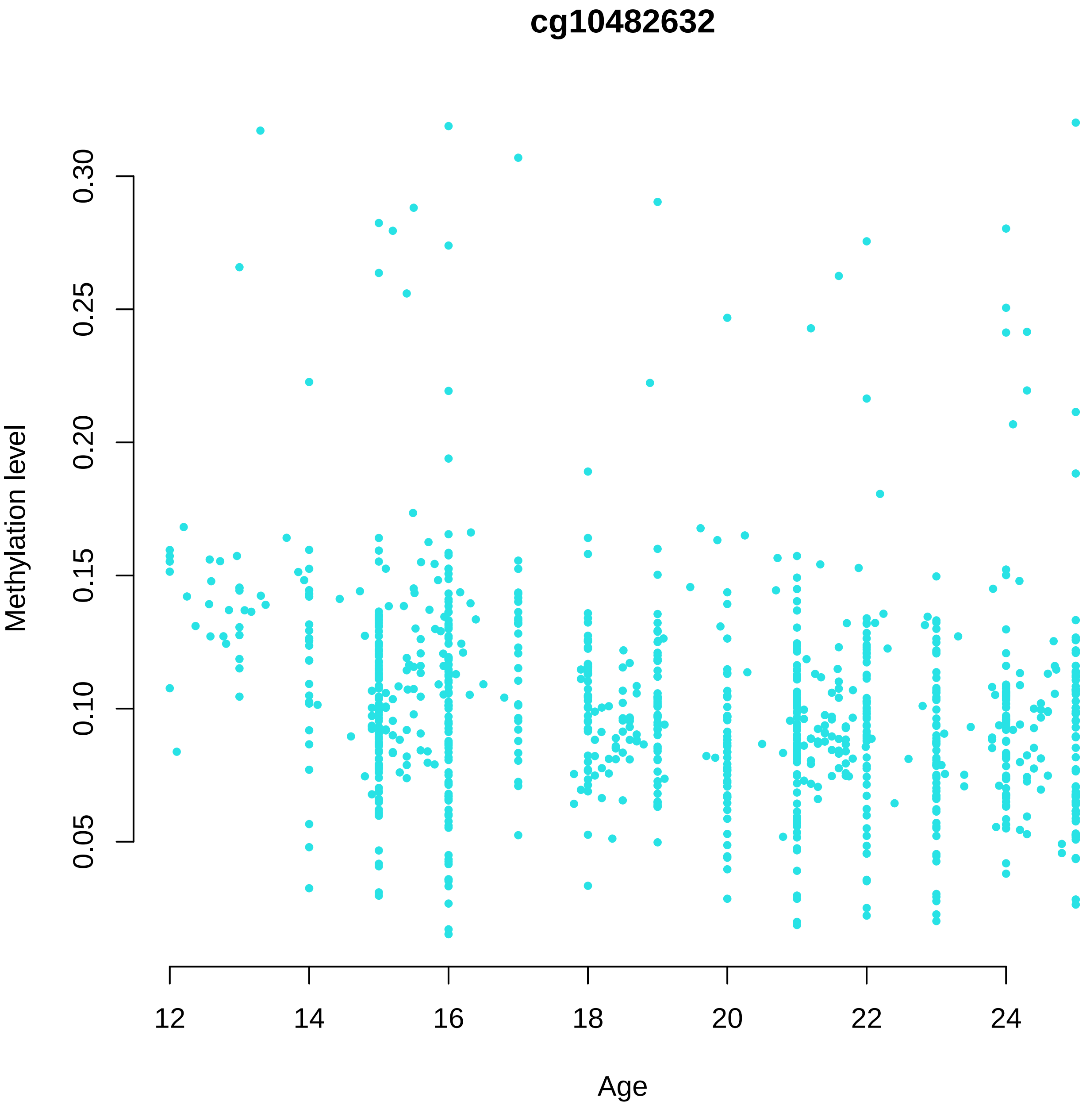
cg10482632
Methylation level
0.05
0.10
0.15
0.20
0.25
0.30
Age
12
14
16
18
20
22
24

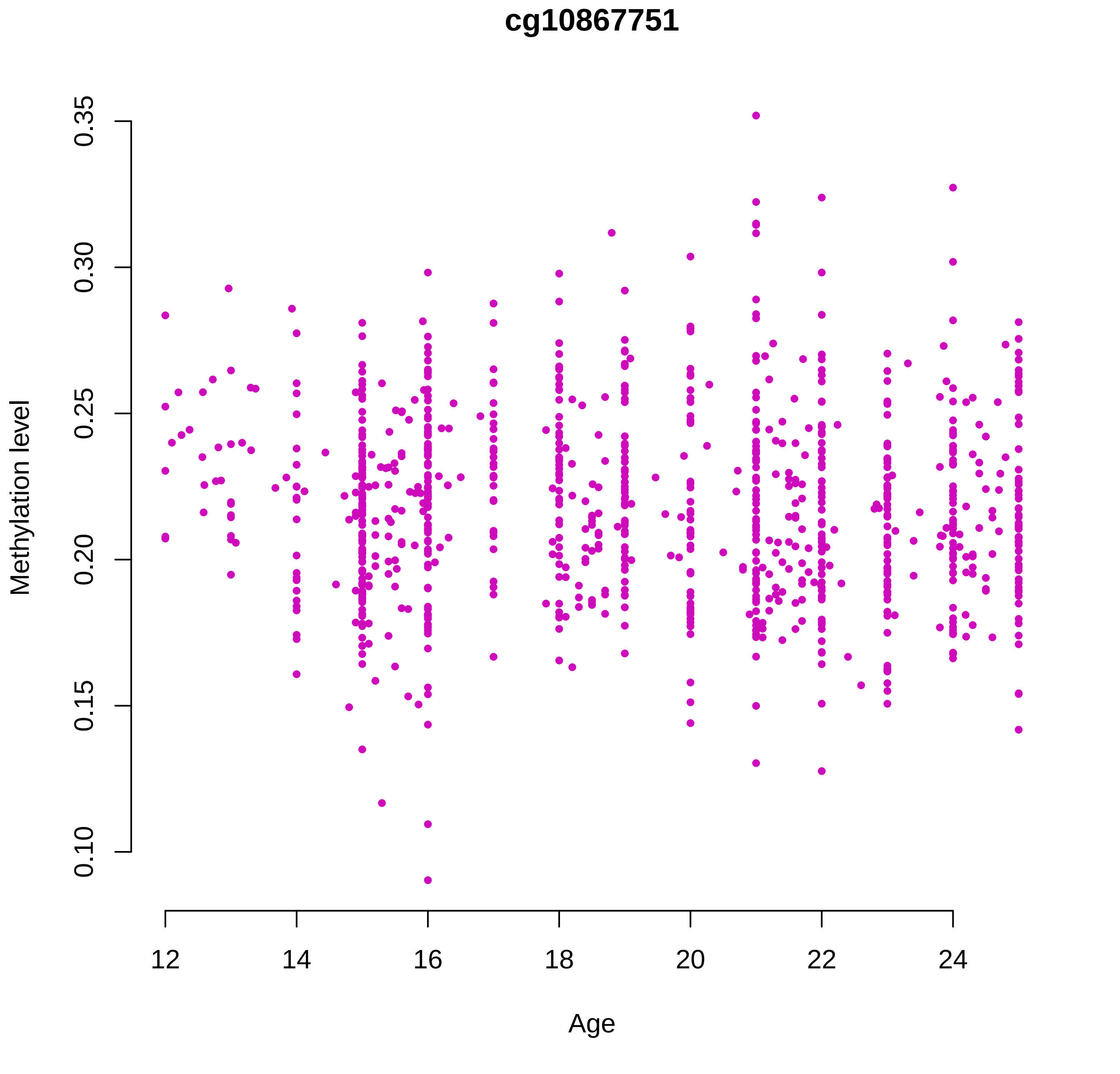
cg10867751
Methylation level
0.10
0.15
0.20
0.25
0.30
0.35
12
14
16
18
20
22
24
Age

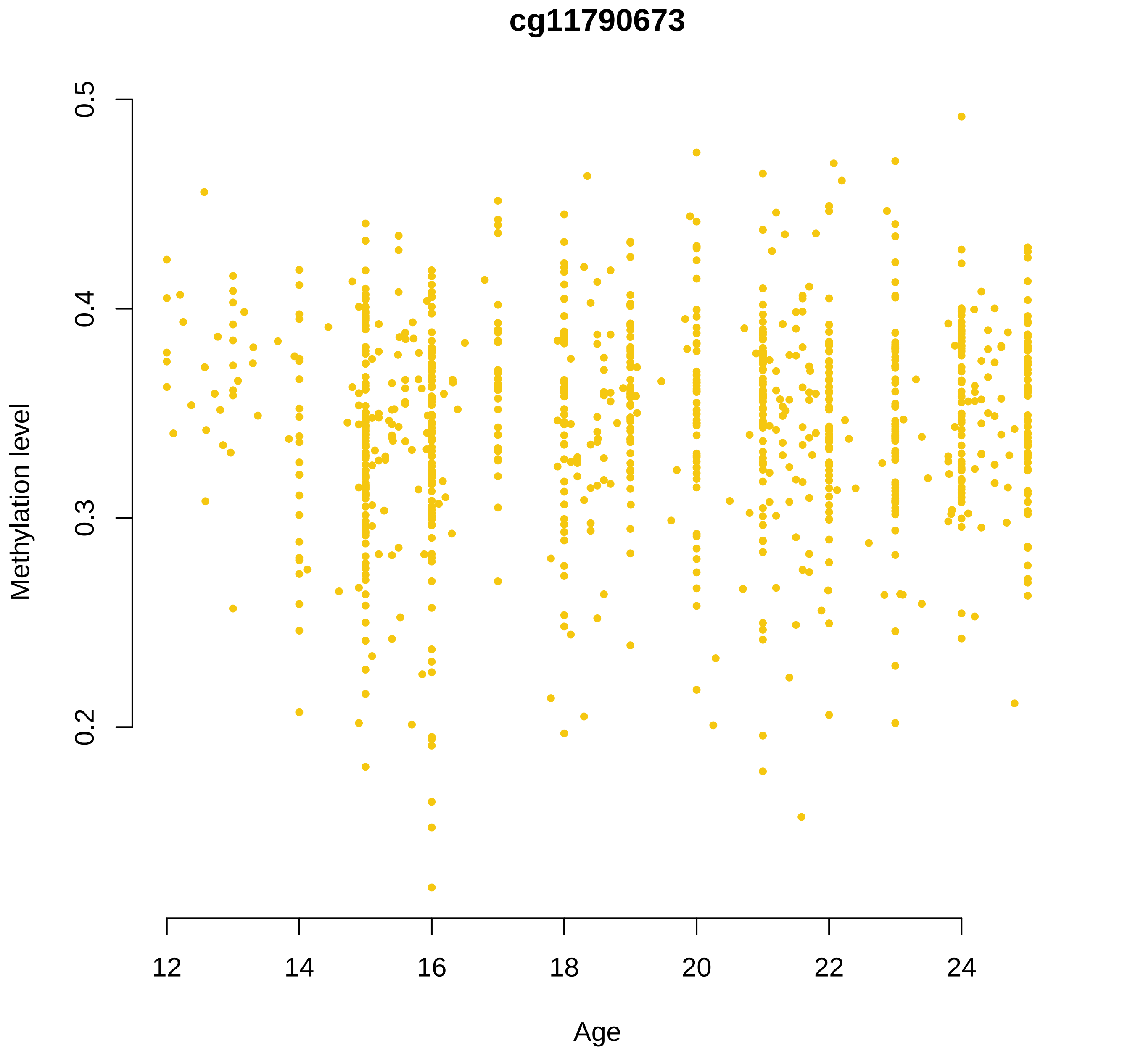
cg11790673
Methylation level
0.2
0.3
0.4
0.5
Age
12
14
16
18
20
22
24

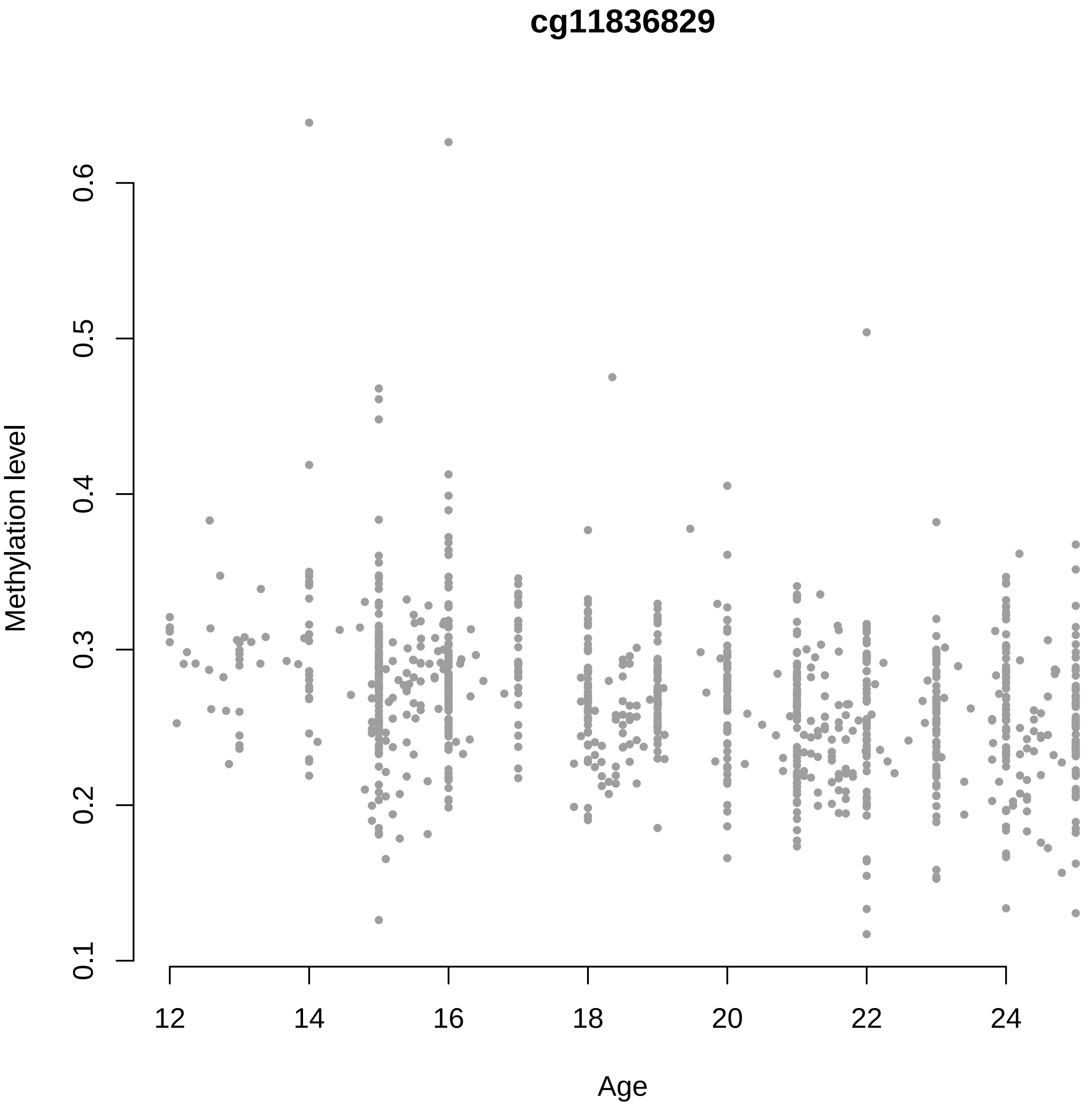
cg11836829
Methylation level
0.1
0.2
0.3
0.4
0.5
0.6
Age
12
14
16
18
20
22
24

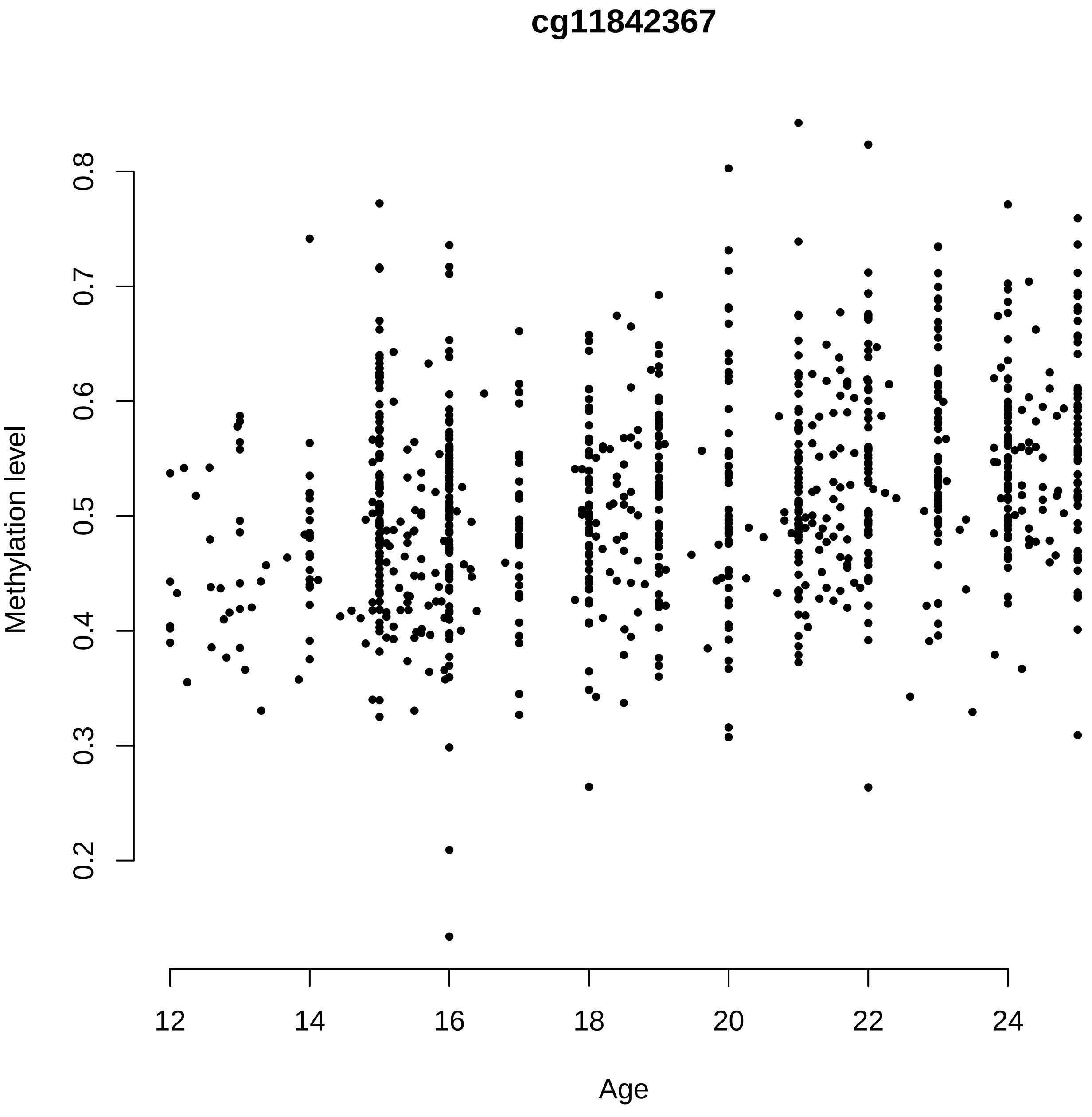

cg11842367
Methylation level
0.2
0.3
0.4
0.5
0.6
0.7
0.8
12
14
16
18
20
22
24
Age

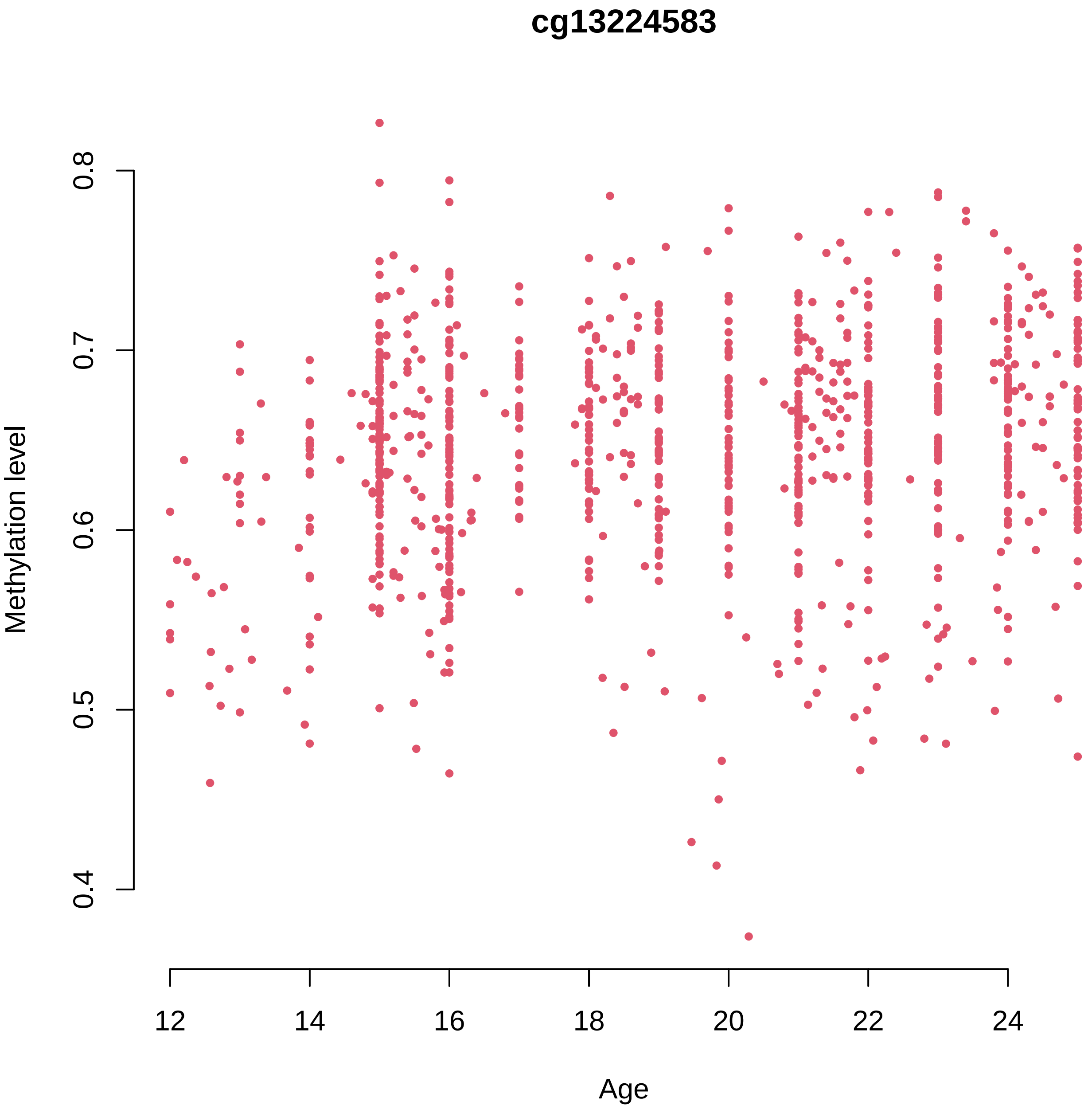
cg13224583
Methylation level
0.4
0.5
0.6
0.7
0.8
12
14
16
18
20
22
24
Age

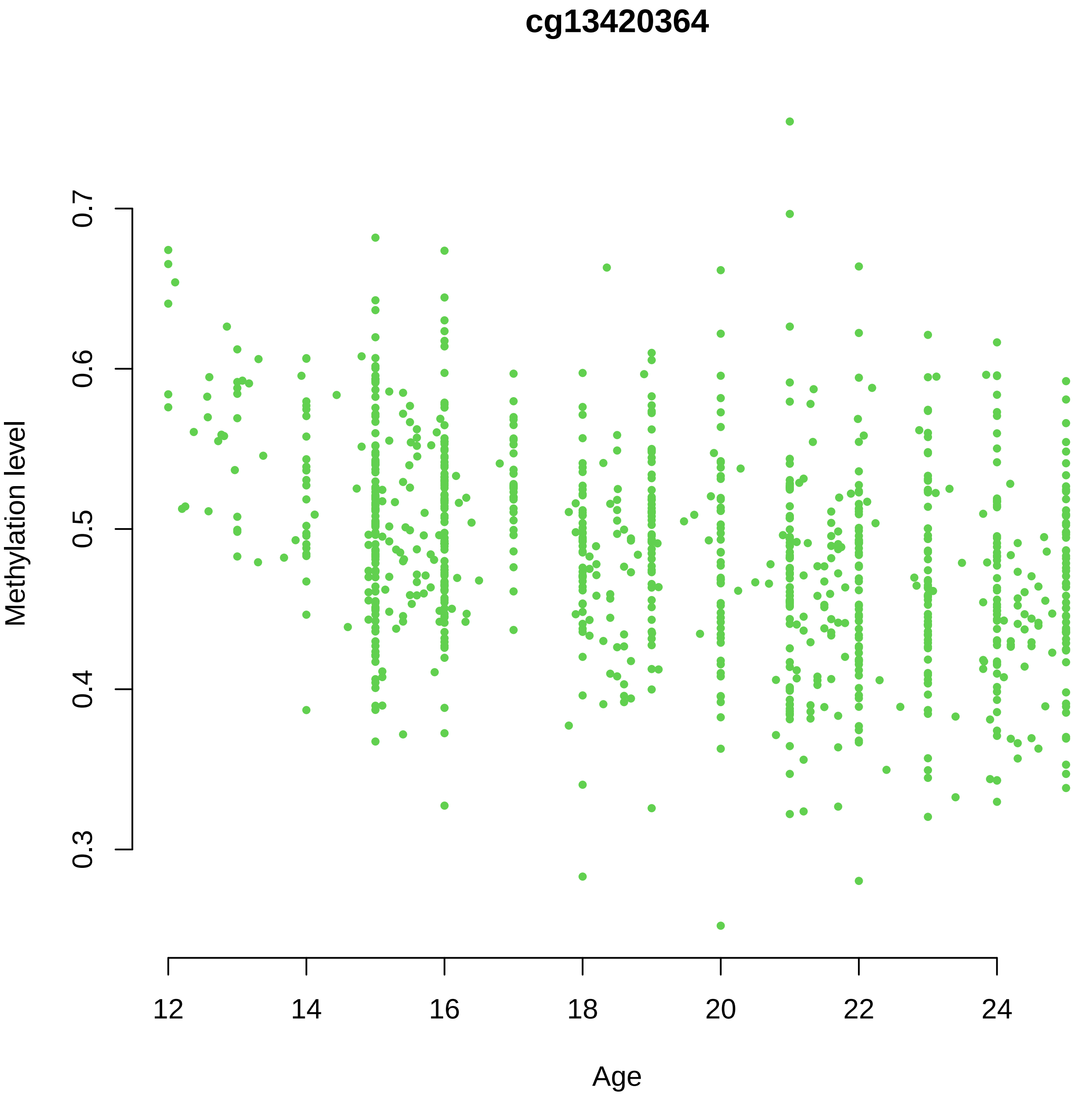

cg13420364
Methylation level
0.3
0.4
0.5
0.6
0.7
Age
12
14
16
18
20
22
24

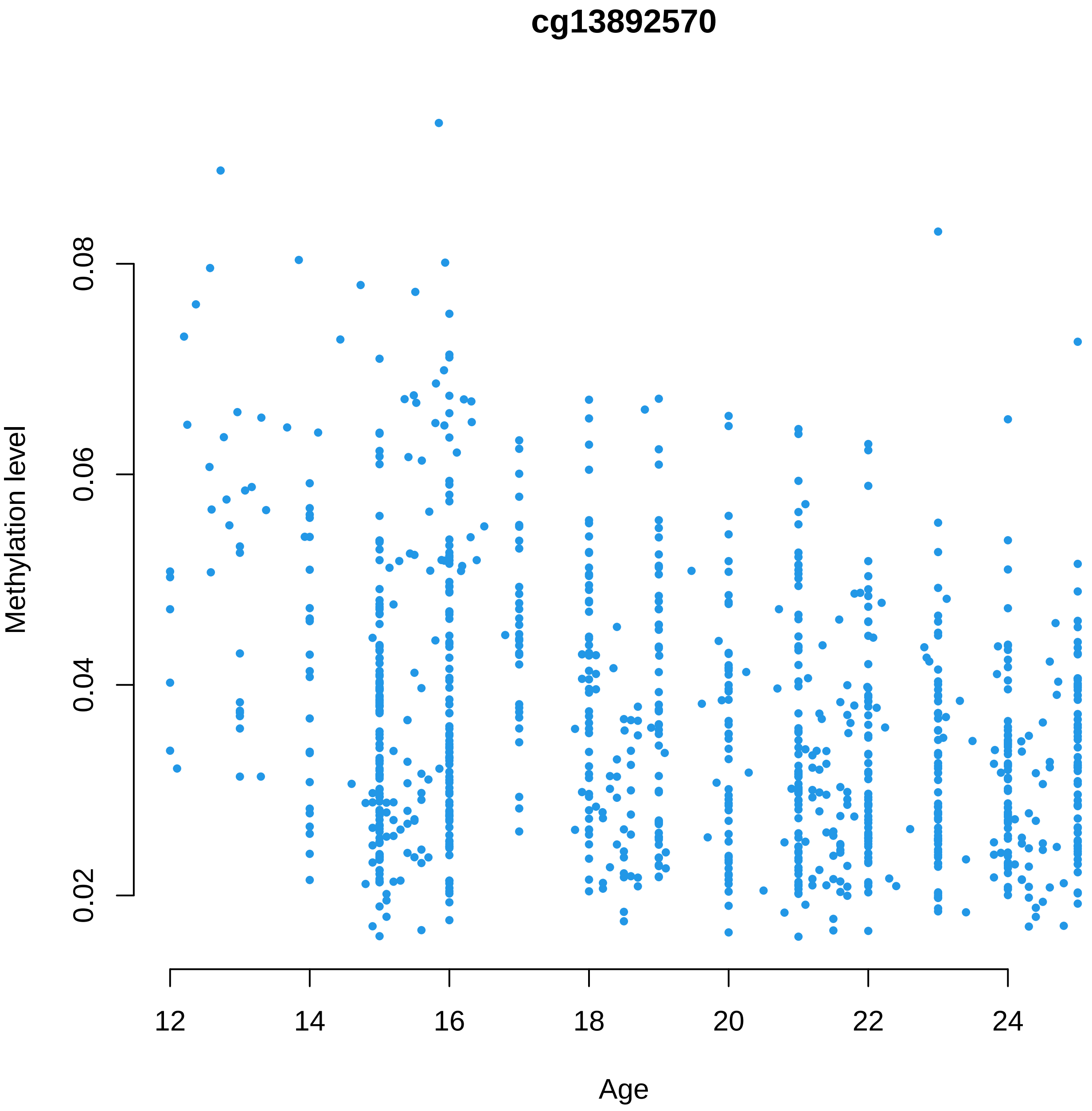
cg13892570
Methylation level
0.02
0.04
0.06
0.08
Age
12
14
16
18
20
22
24

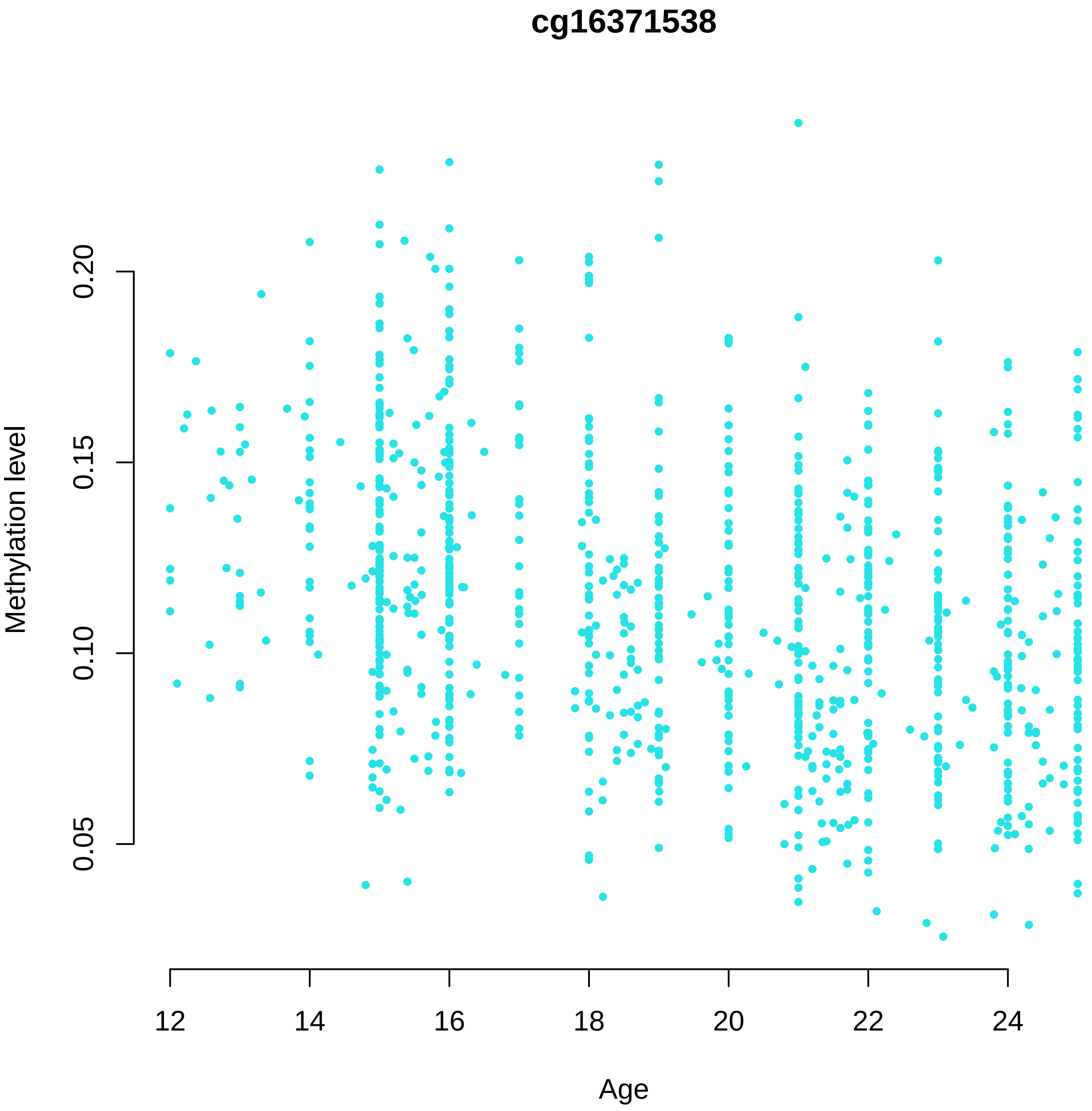

cg16371538
Methylation level
0.05
0.10
0.15
0.20
12
14
16
18
20
22
24
Age

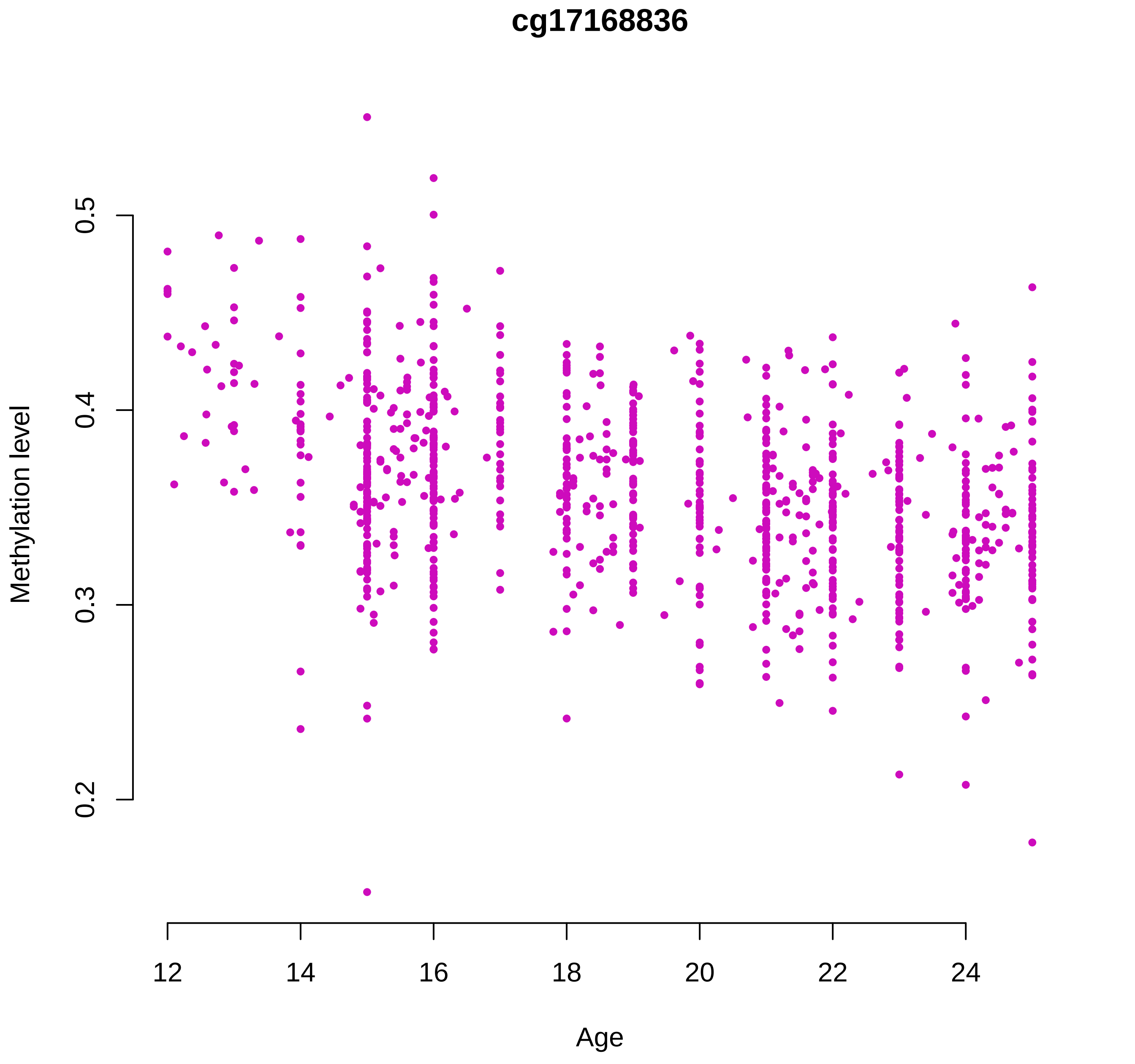
cg17168836
Methylation level
0.2
0.3
0.4
0.5
12
14
16
18
20
22
24
Age

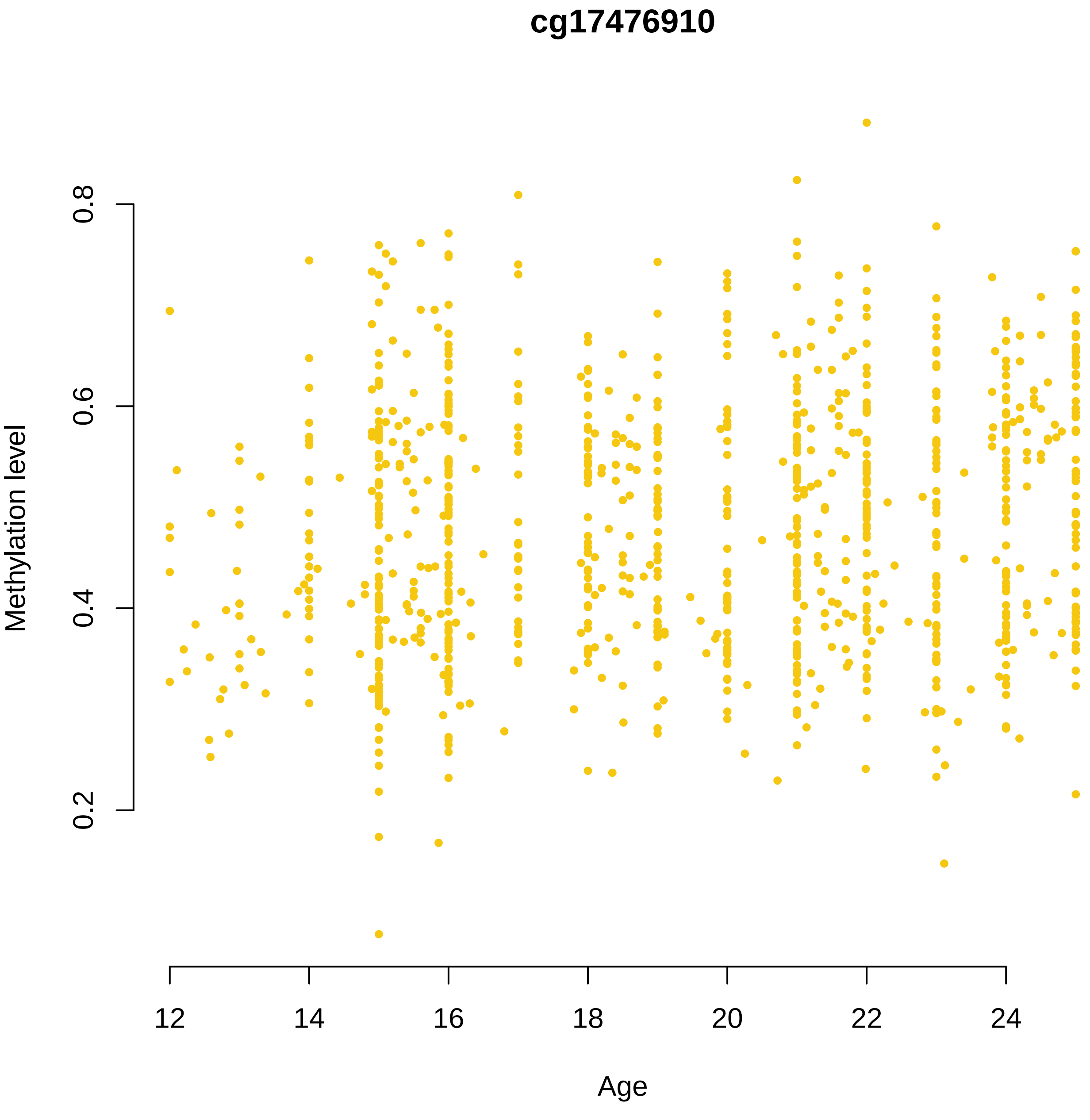
cg17476910
Methylation level
0.2
0.4
0.6
0.8
Age
12
14
16
18
20
22
24

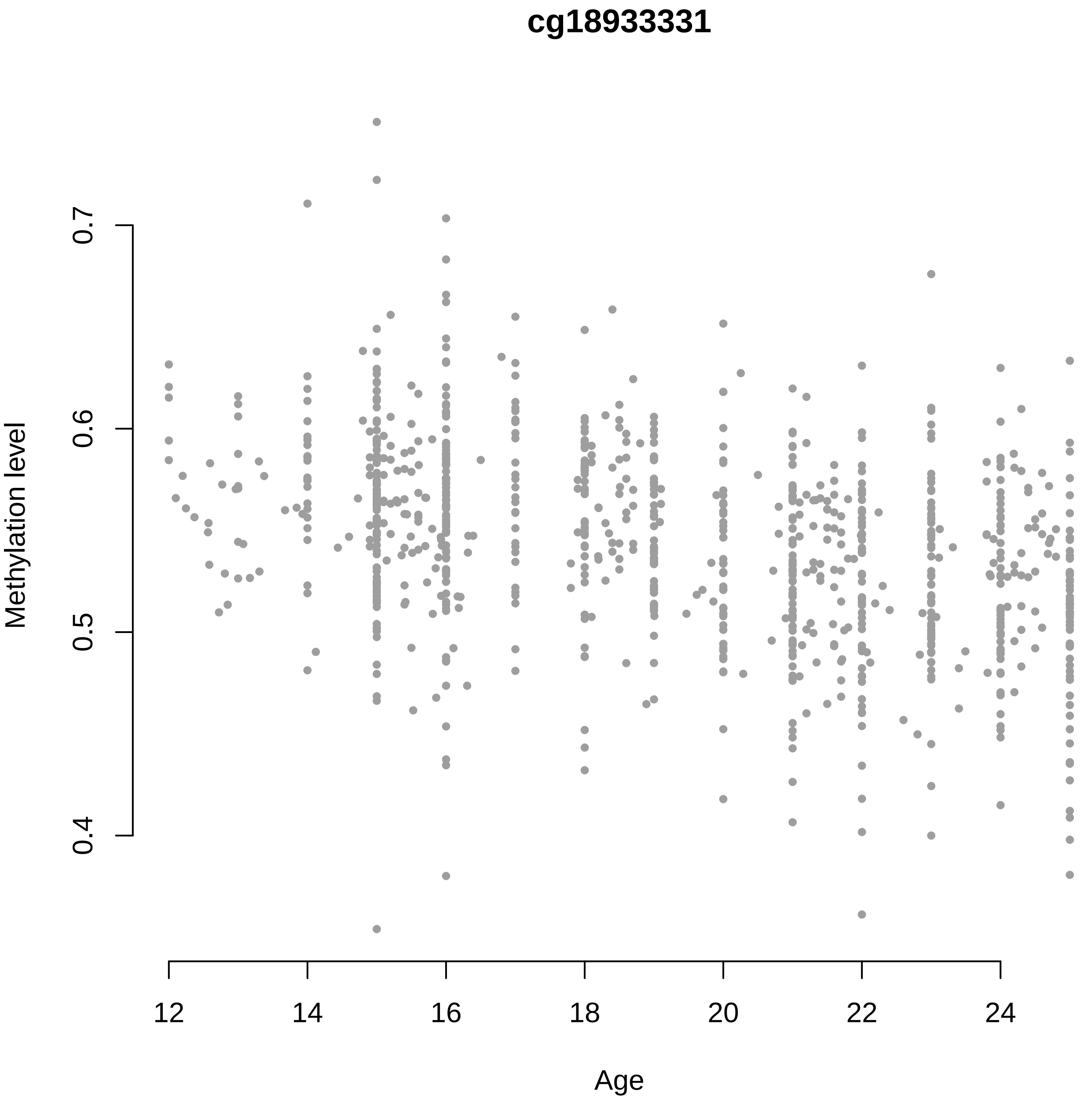
cg18933331
Methylation level
0.4
0.5
0.6
0.7
Age
12
14
16
18
20
22
24

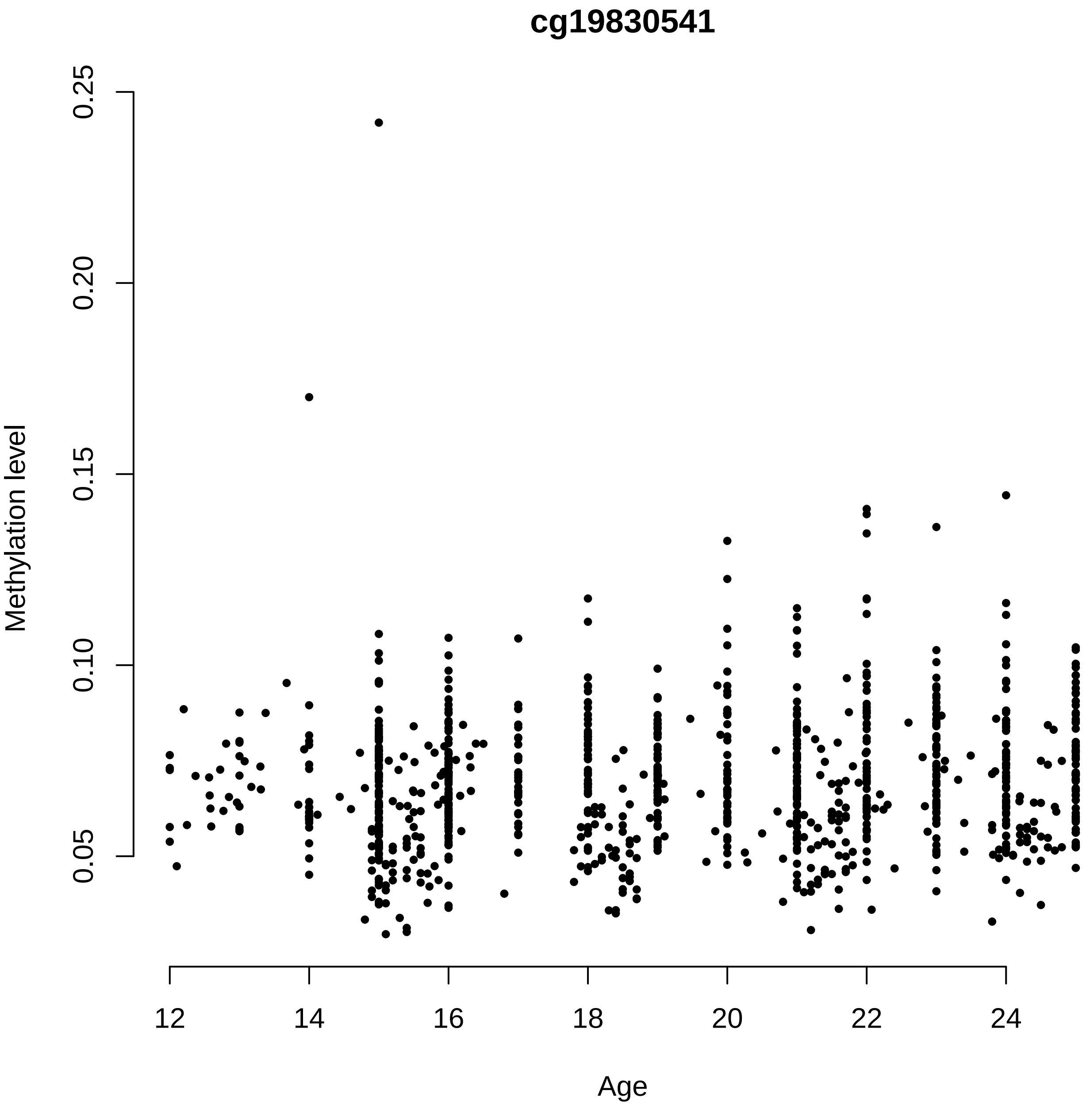
cg19830541
Methylation level
0.05
0.10
0.15
0.20
0.25
Age
12
14
16
18
20
22
24

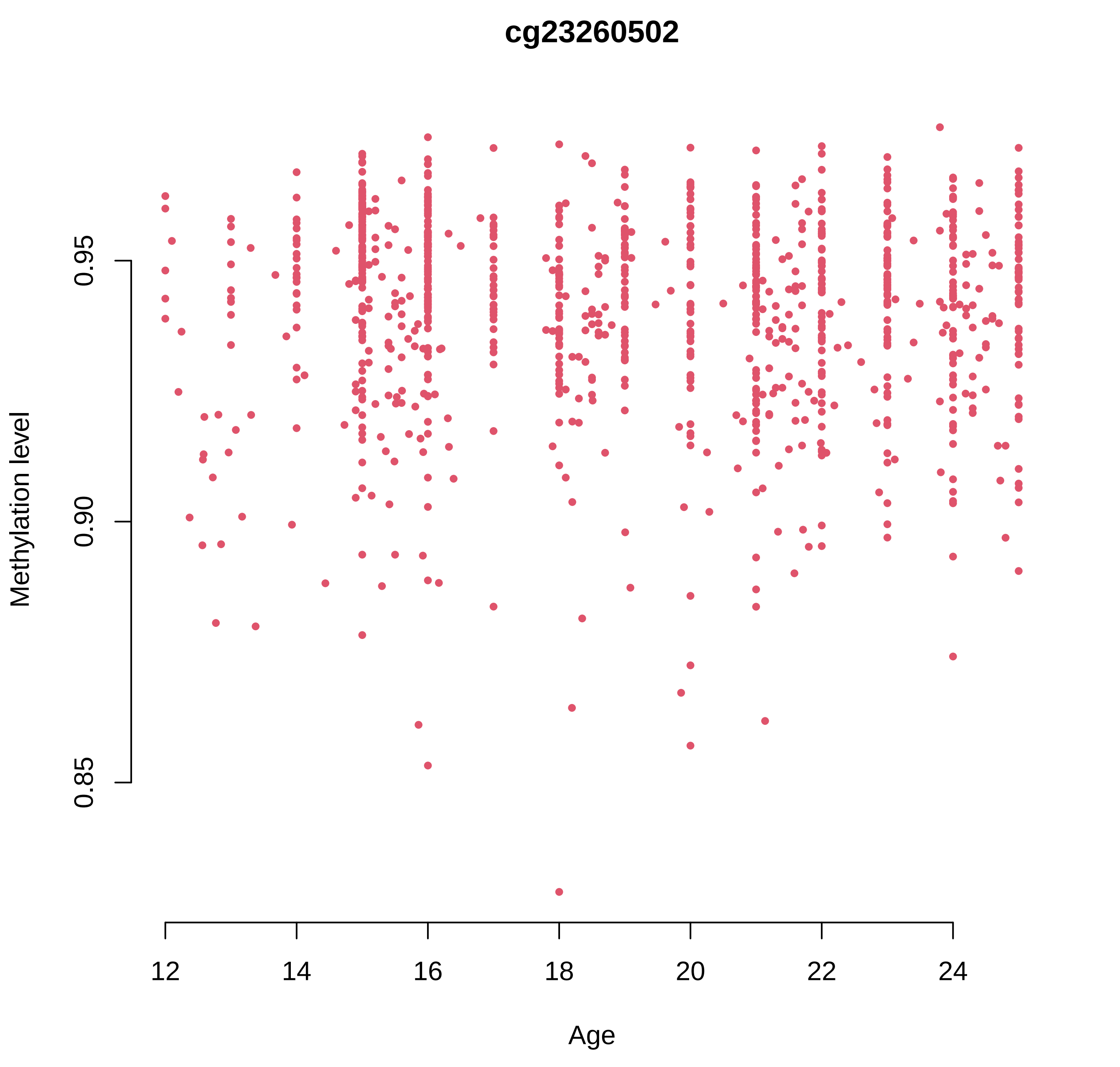
cg23260502
Methylation level
0.85
0.90
0.95
Age
12
14
16
18
20
22
24

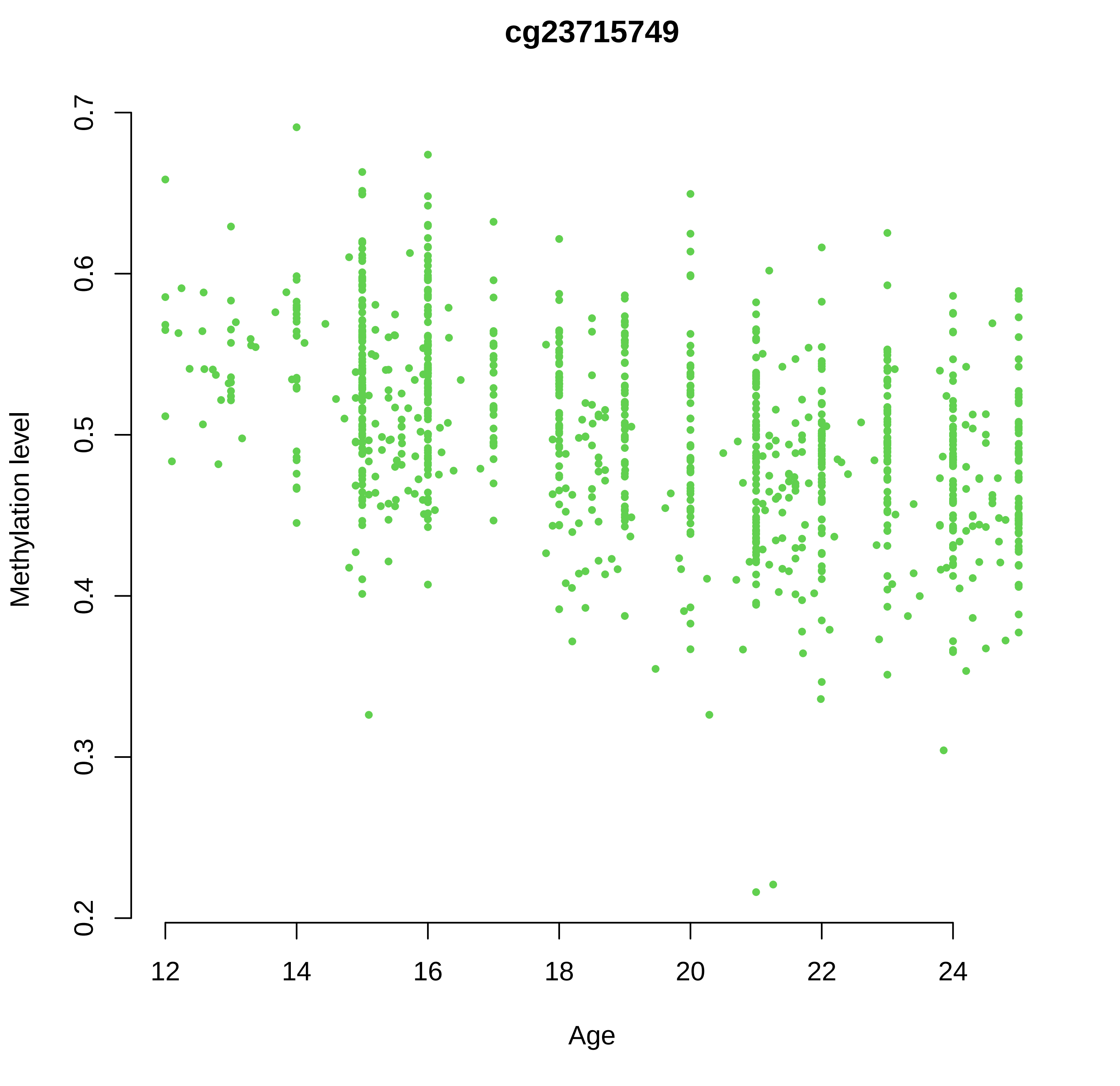
cg23715749
Methylation level
0.2
0.3
0.4
0.5
0.6
0.7
Age
12
14
16
18
20
22
24

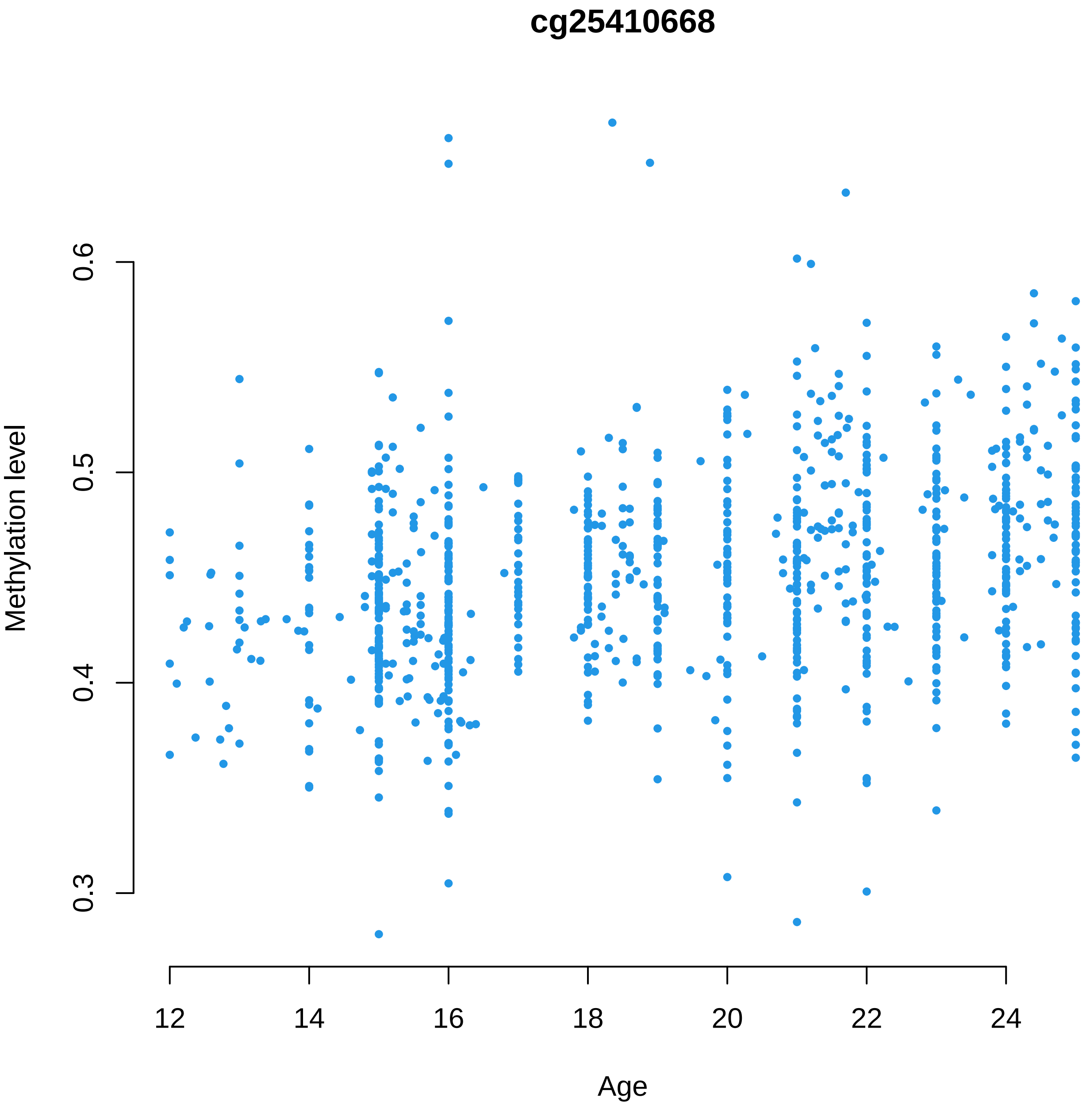
cg25410668
Methylation level
0.3
0.4
0.5
0.6
12
14
16
18
20
22
24
Age

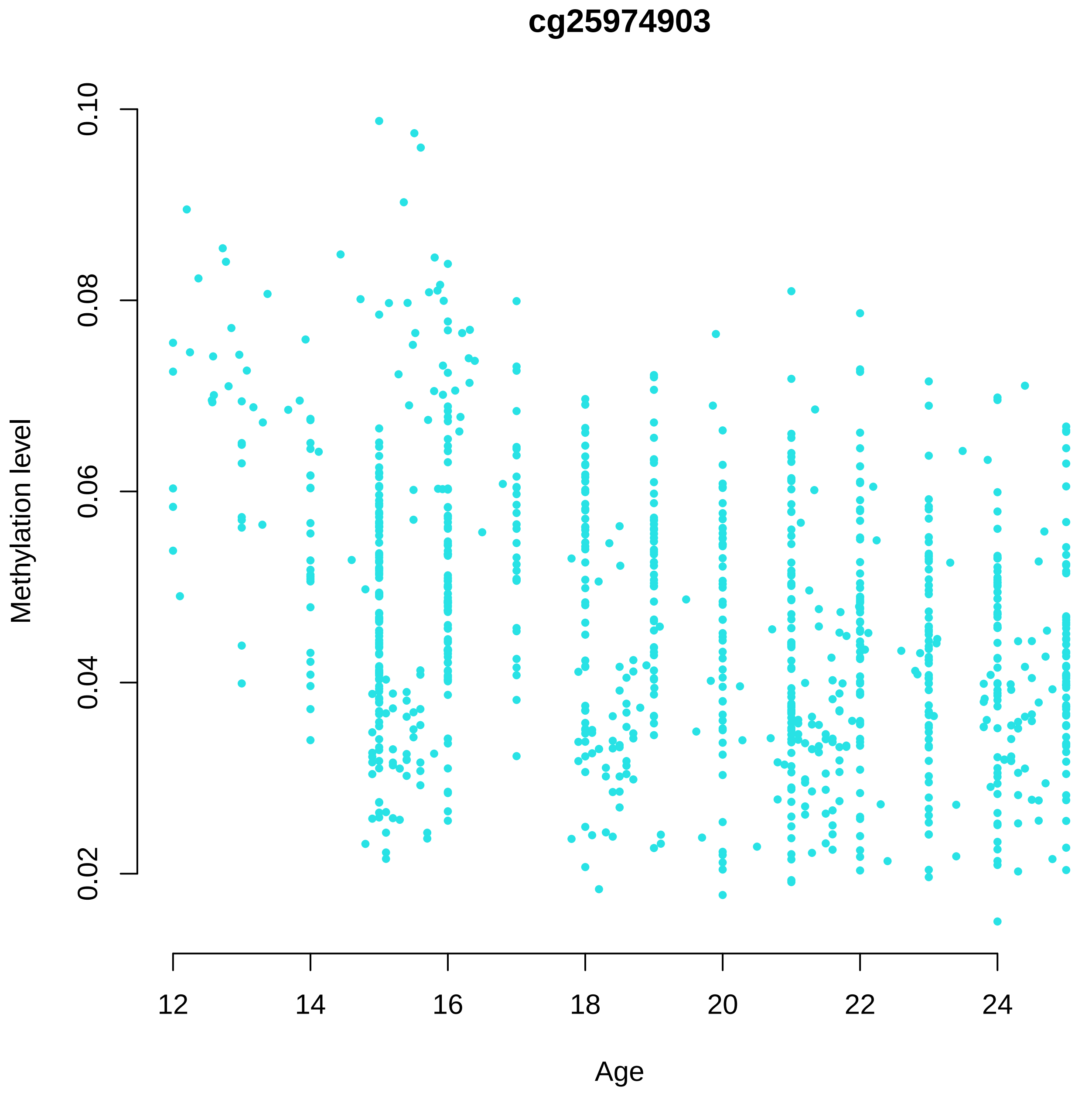
cg25974903
Methylation level
0.02
0.04
0.06
0.08
0.10
Age
12
14
16
18
20
22
24

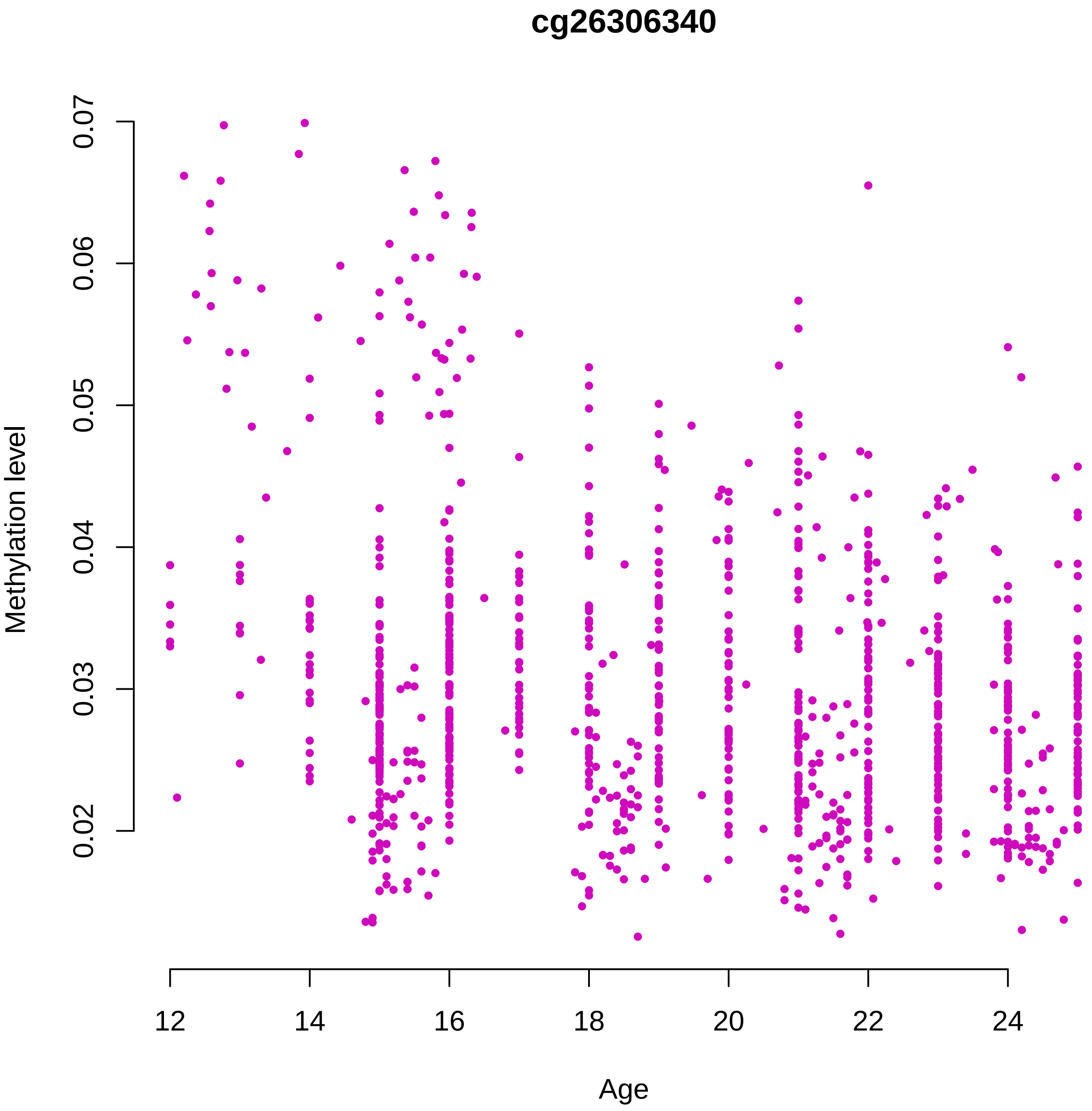

cg26306340
Methylation level
0.02
0.03
0.04
0.05
0.06
0.07
12
14
16
18
20
22
24
Age

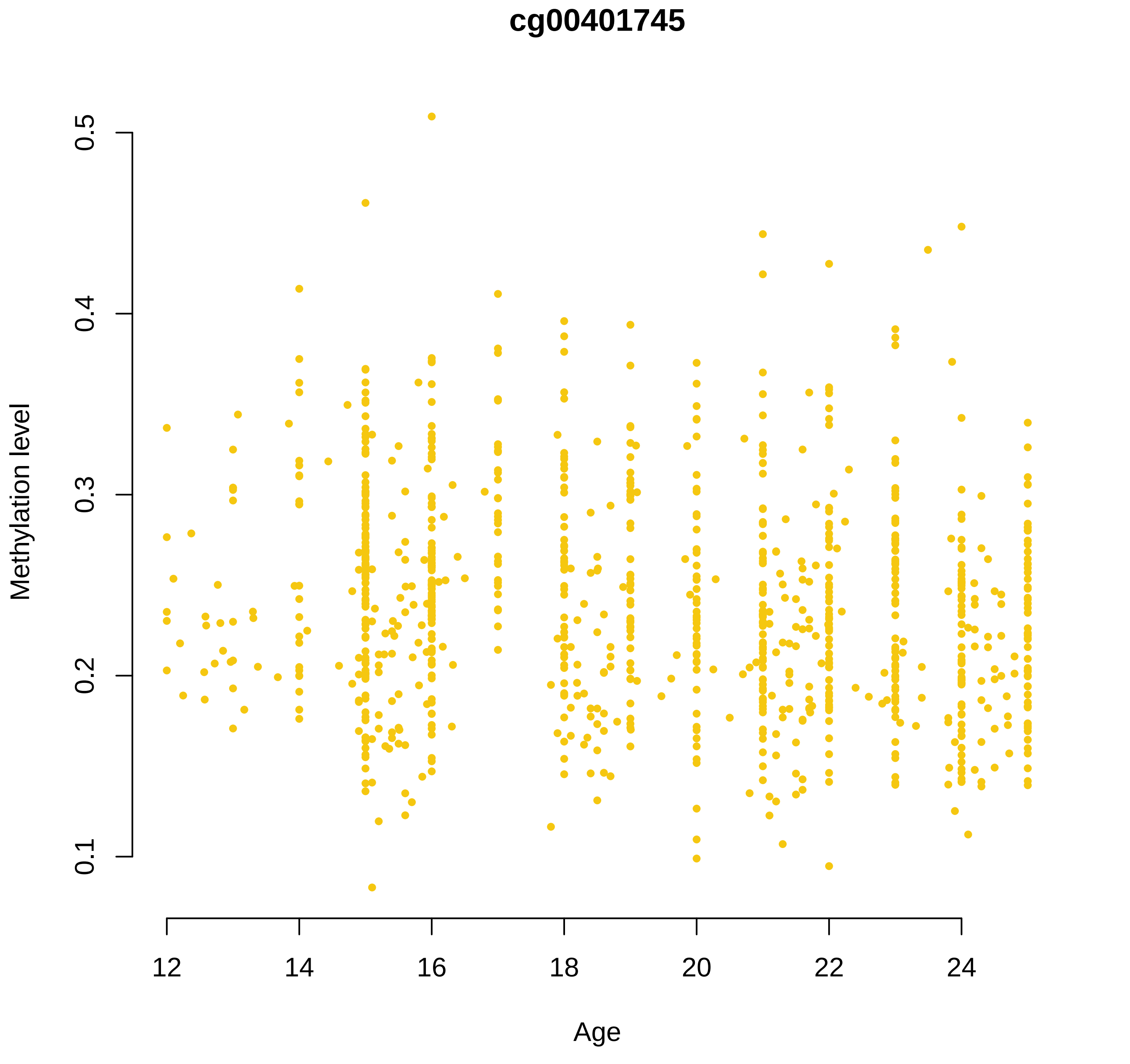
cg00401745
Methylation level
0.1
0.2
0.3
0.4
0.5
Age
12
14
16
18
20
22
24

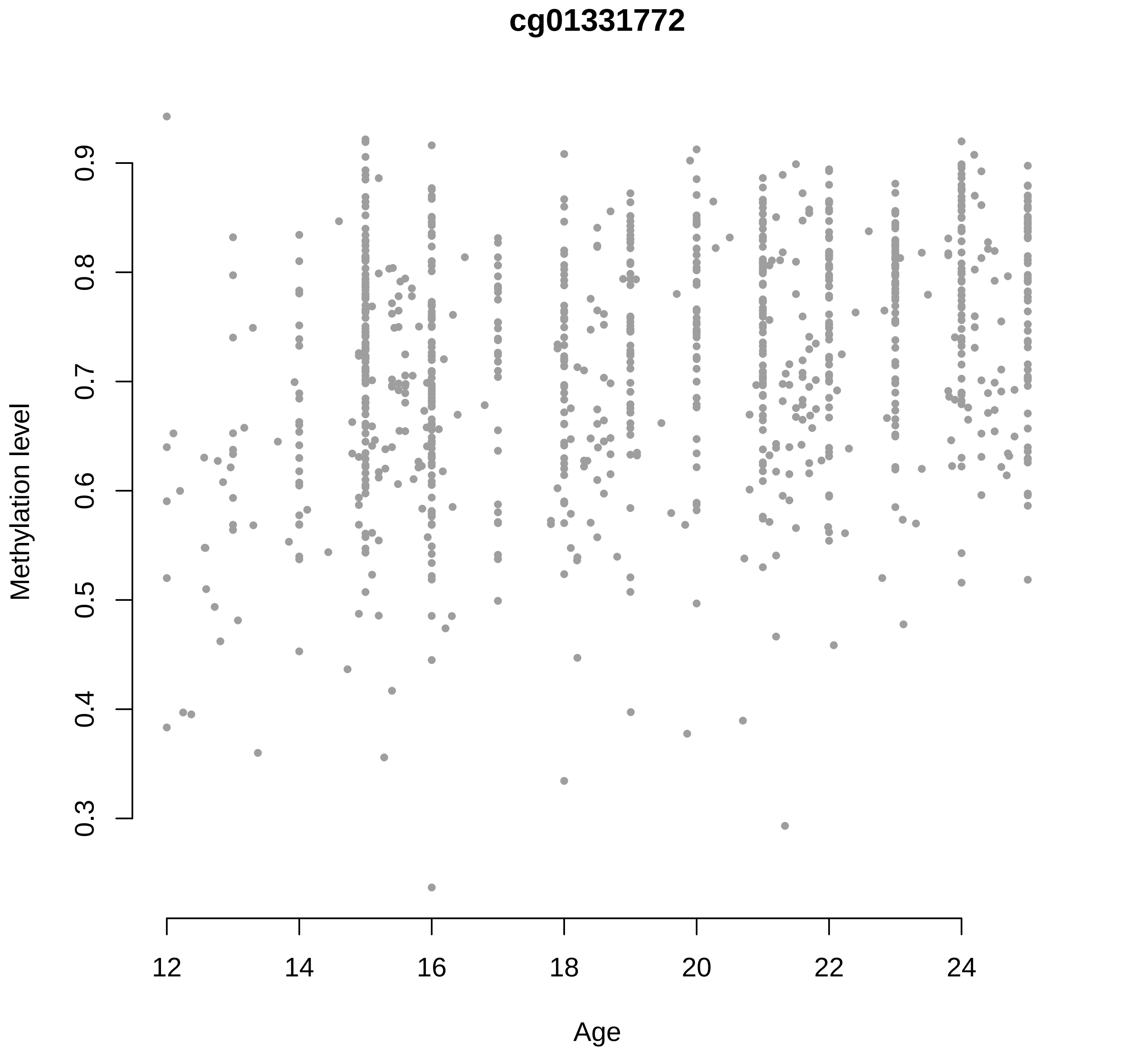

cg01331772
Methylation level
0.3
0.4
0.5
0.6
0.7
0.8
0.9
Age
12
14
16
18
20
22
24

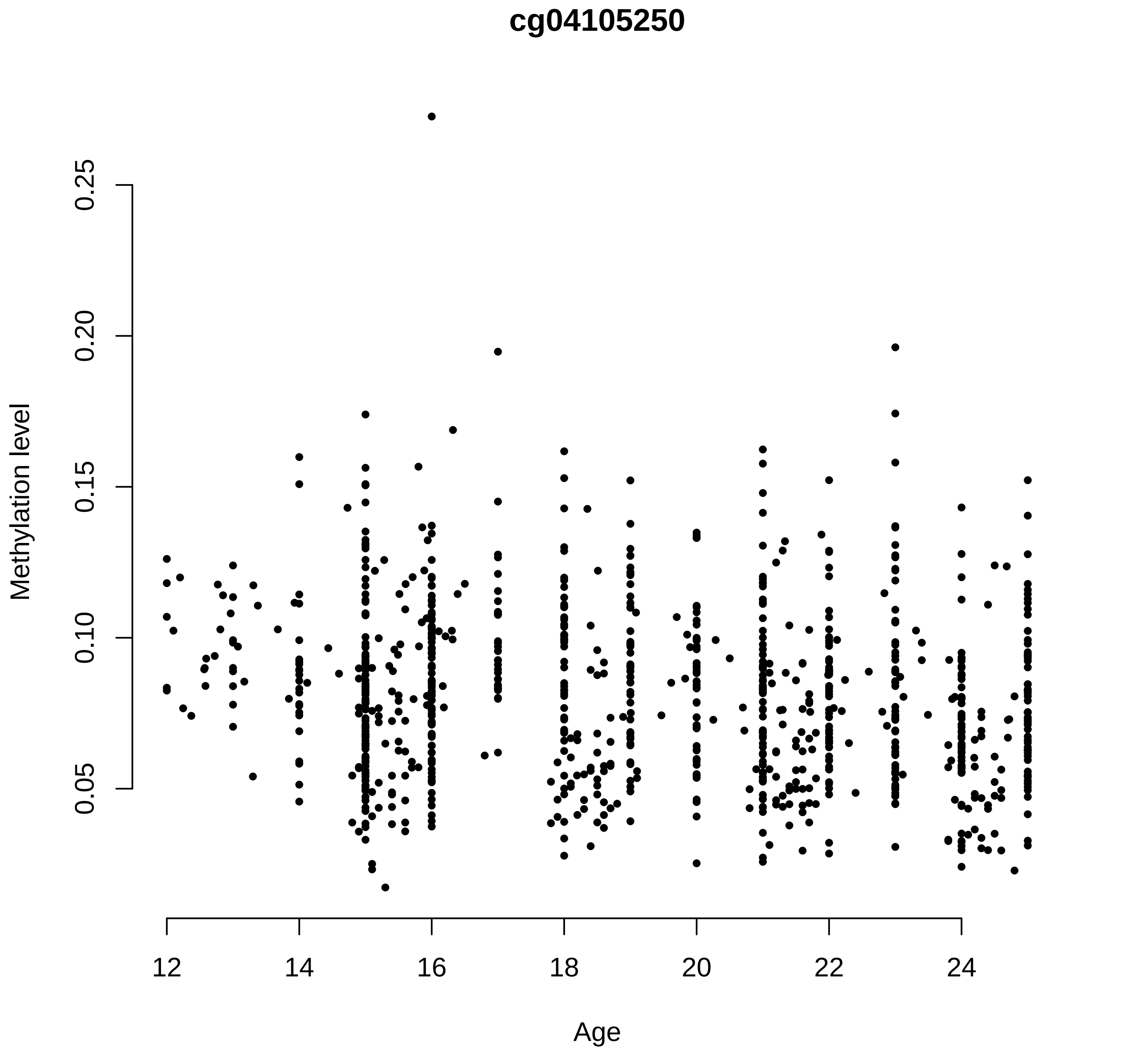
cg04105250
Methylation level
0.05
0.10
0.15
0.20
0.25
12
14
16
18
20
22
24
Age

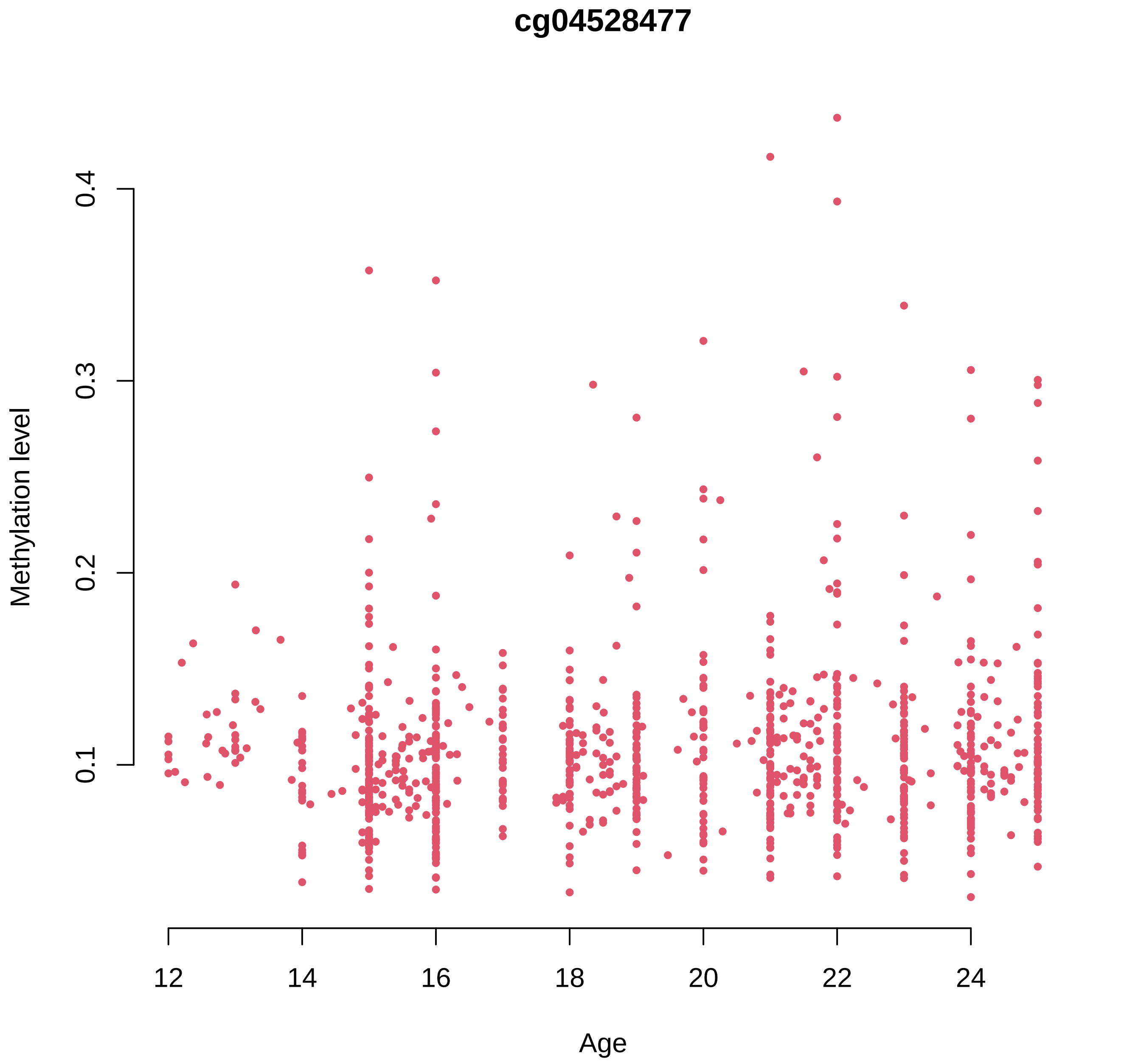

cg04528477
Methylation level
0.1
0.2
0.3
0.4
Age
12
14
16
18
20
22
24

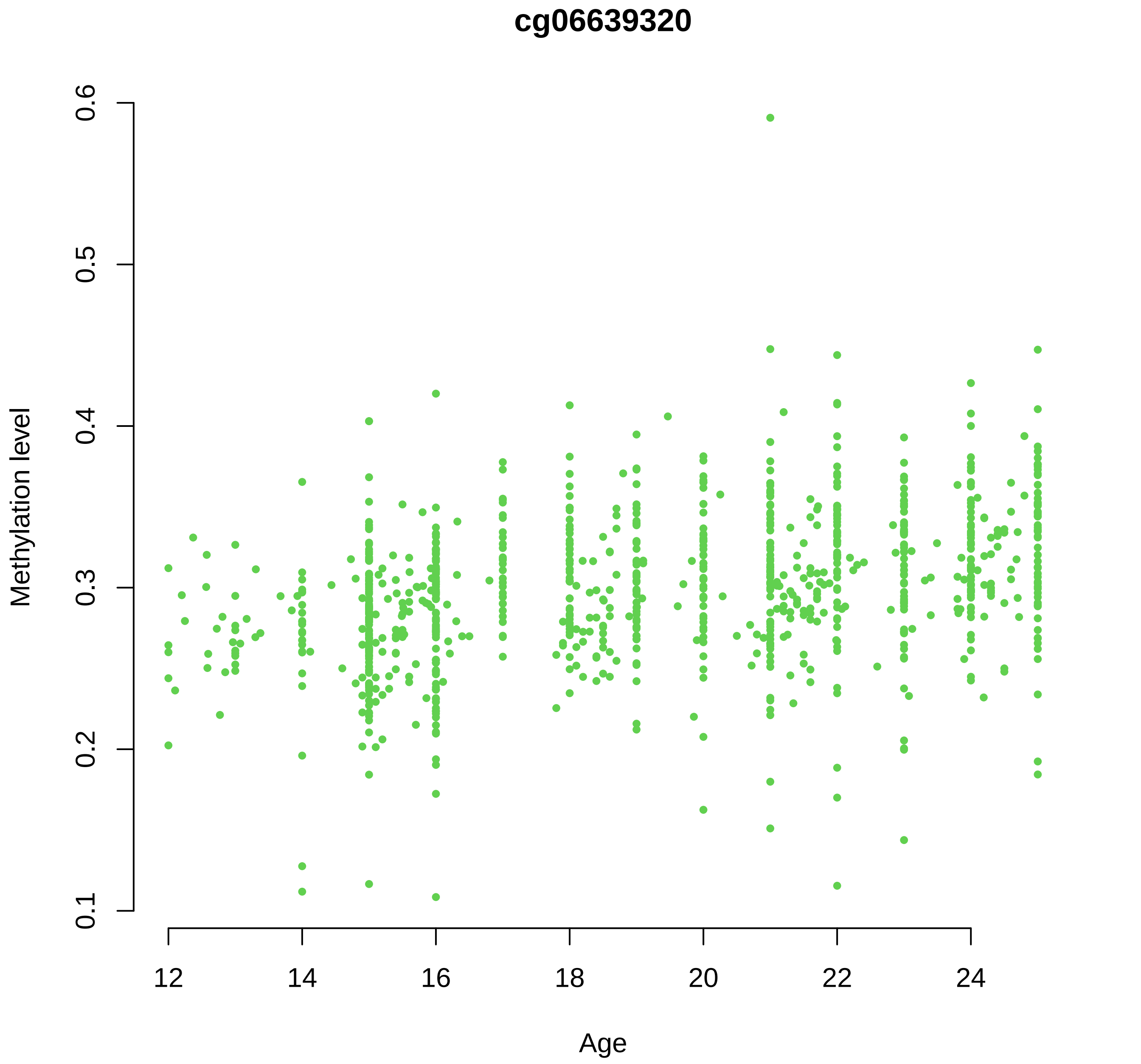

cg06639320
Methylation level
0.1
0.2
0.3
0.4
0.5
0.6
12
14
16
18
20
22
24
Age

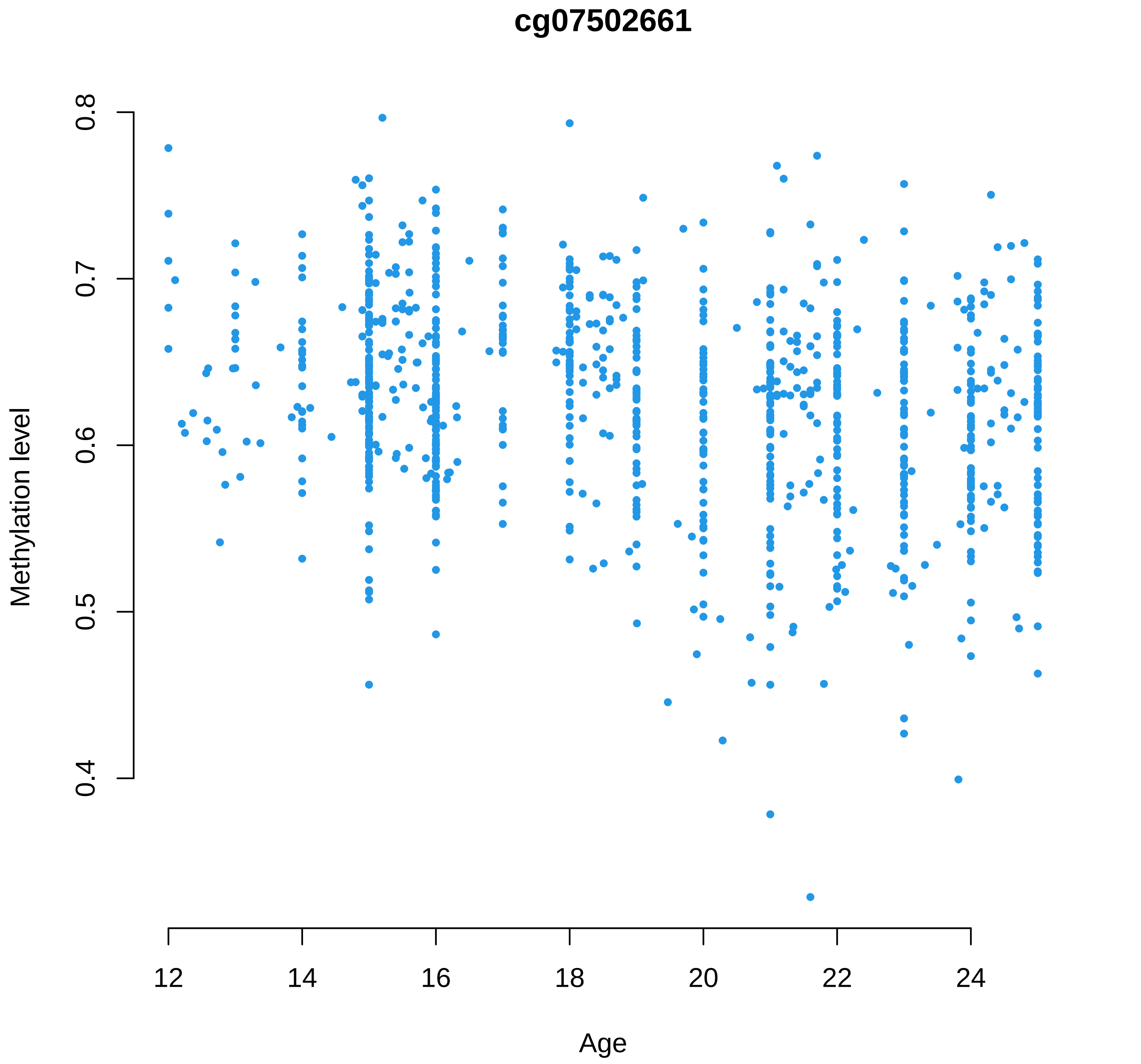
cg07502661
Methylation level
0.4
0.5
0.6
0.7
0.8
Age
12
14
16
18
20
22
24

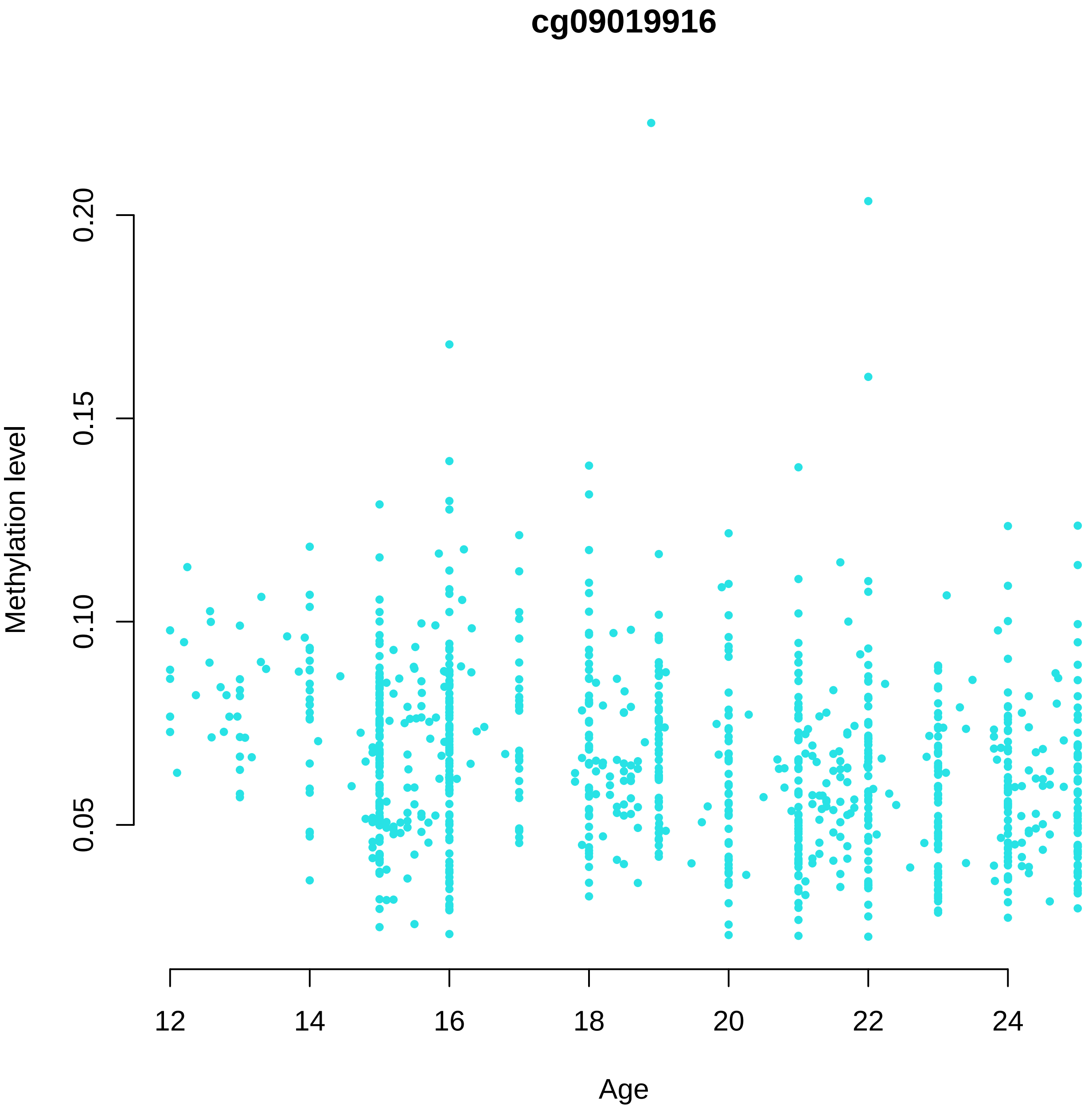
cg09019916
Methylation level
0.05
0.10
0.15
0.20
12
14
16
18
20
22
24
Age

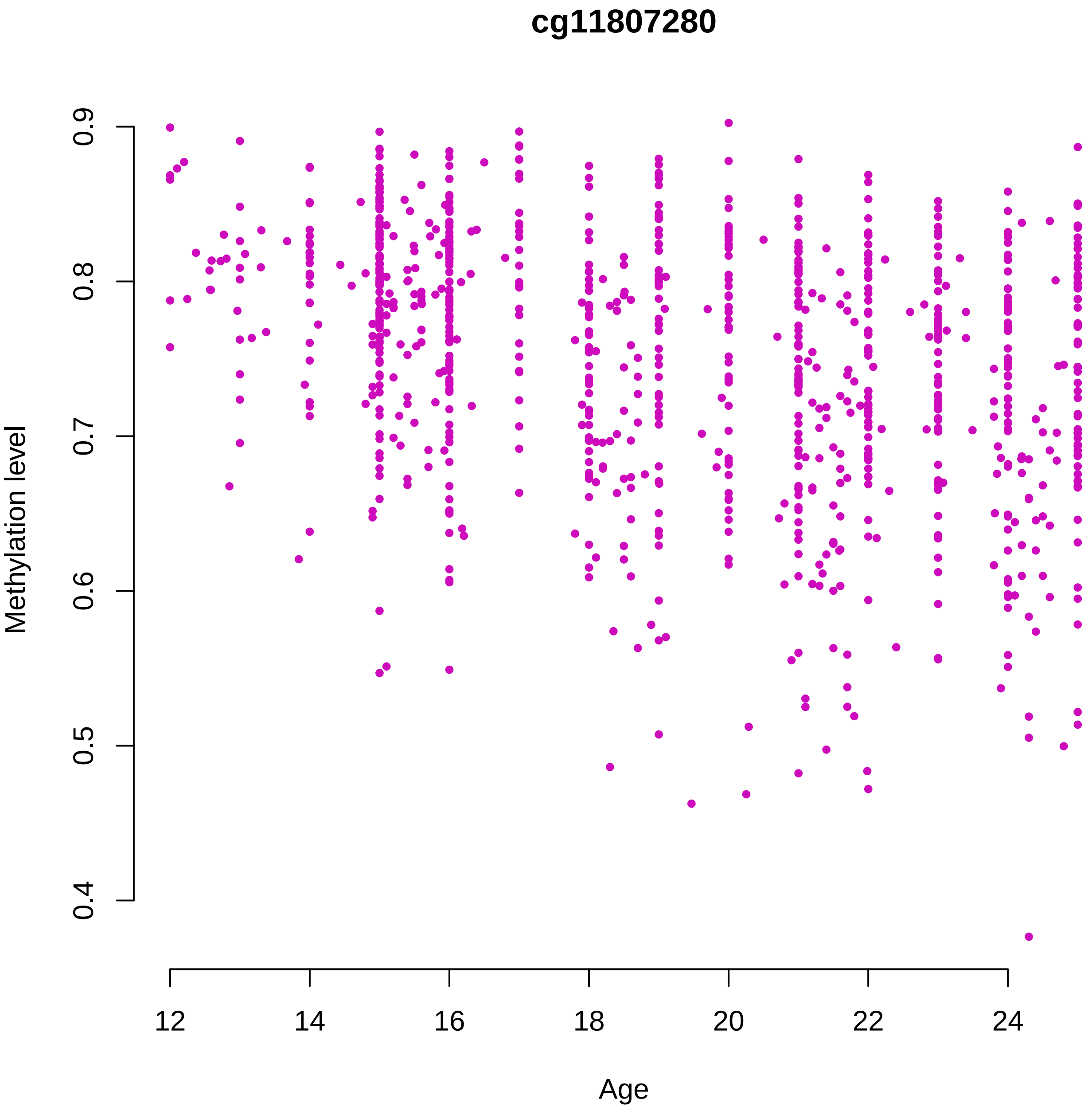
cg11807280
Methylation level
0.4
0.5
0.6
0.7
0.8
0.9
Age
12
14
16
18
20
22
24

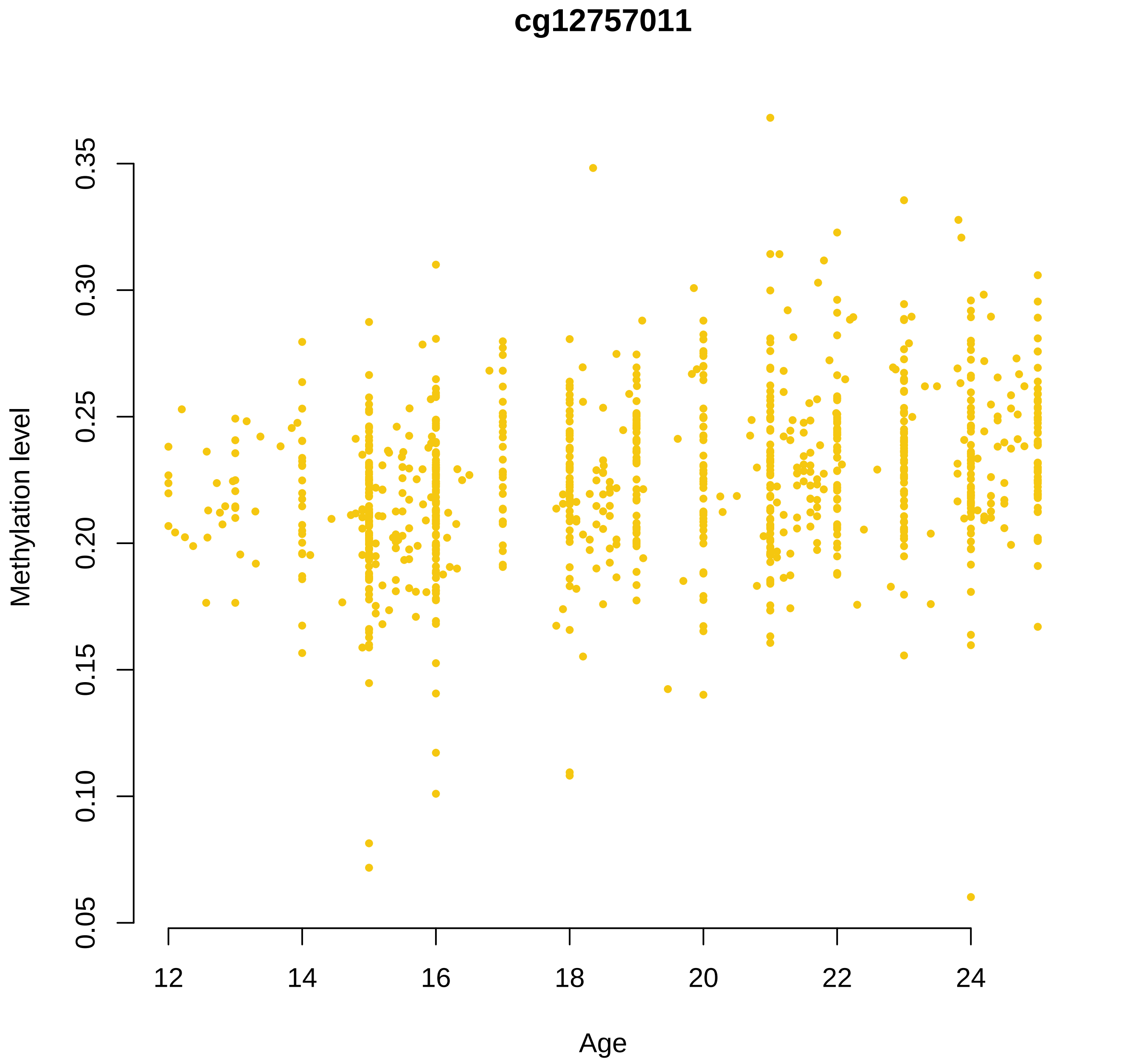

cg12757011
Methylation level
0.05
0.10
0.15
0.20
0.25
0.30
0.35
Age
12
14
16
18
20
22
24

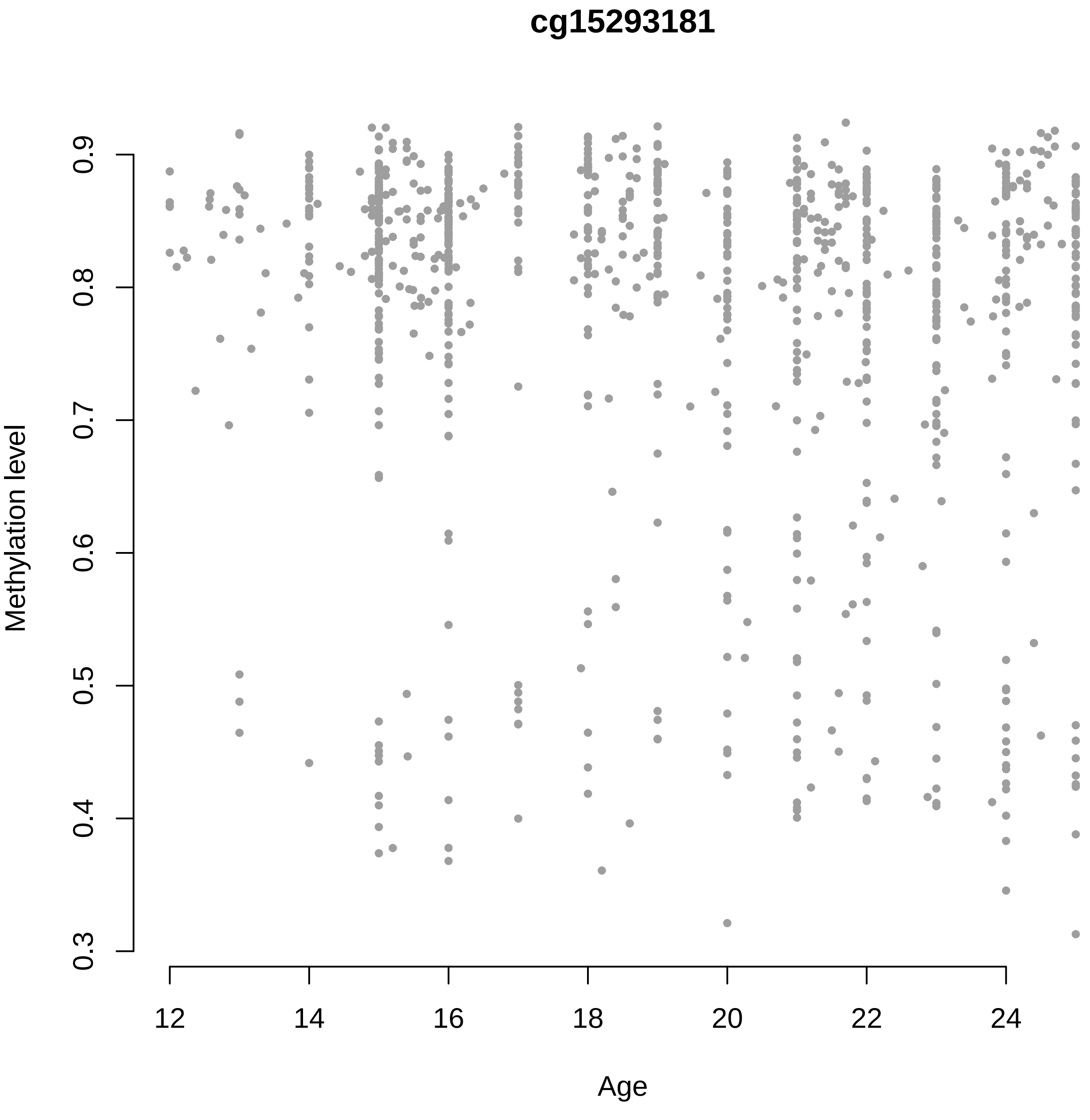
cg15293181
Methylation level
0.3
0.4
0.5
0.6
0.7
0.8
0.9
Age
12
14
16
18
20
22
24

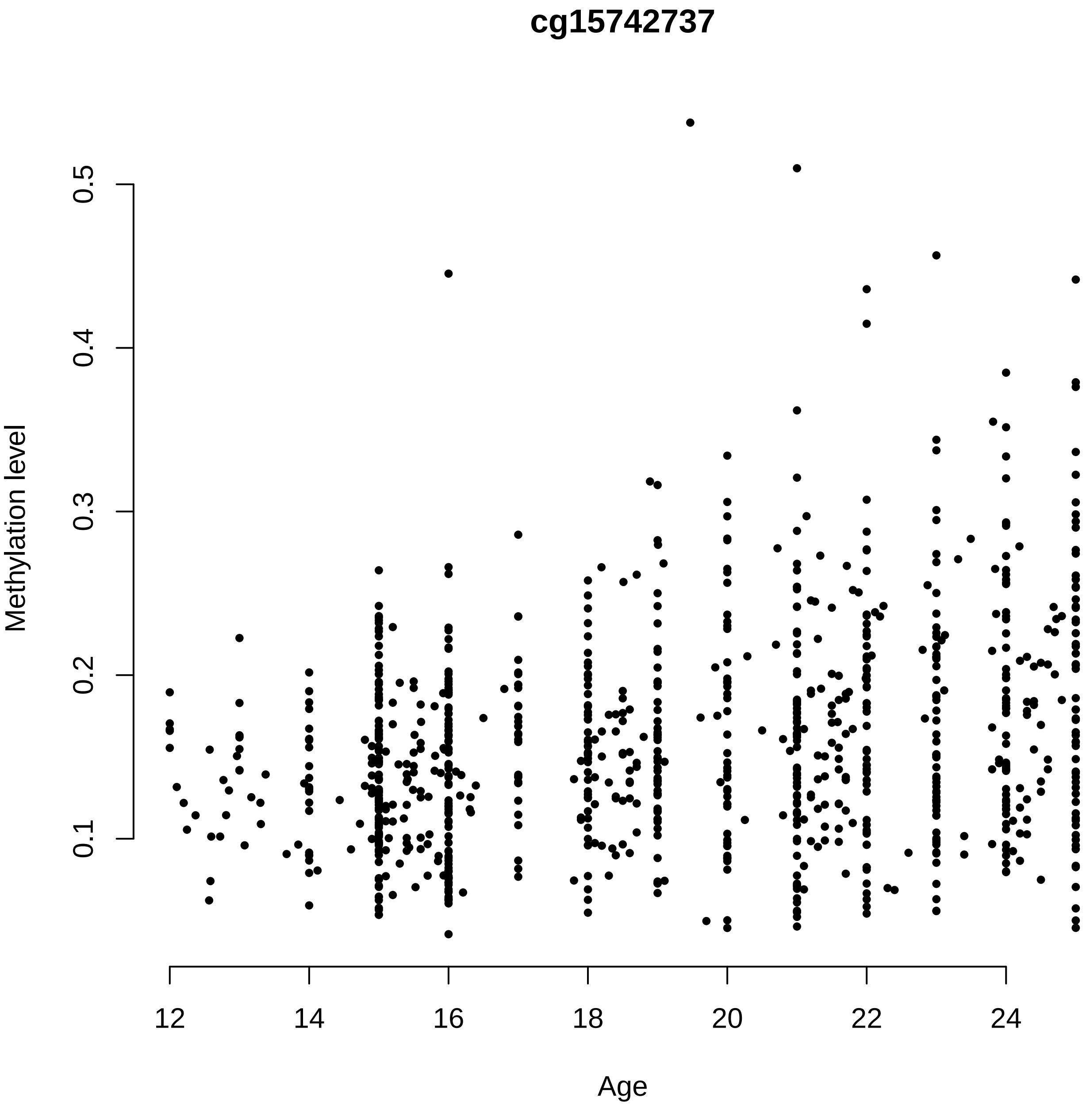
cg15742737
Methylation level
0.1
0.2
0.3
0.4
0.5
Age
12
14
16
18
20
22
24

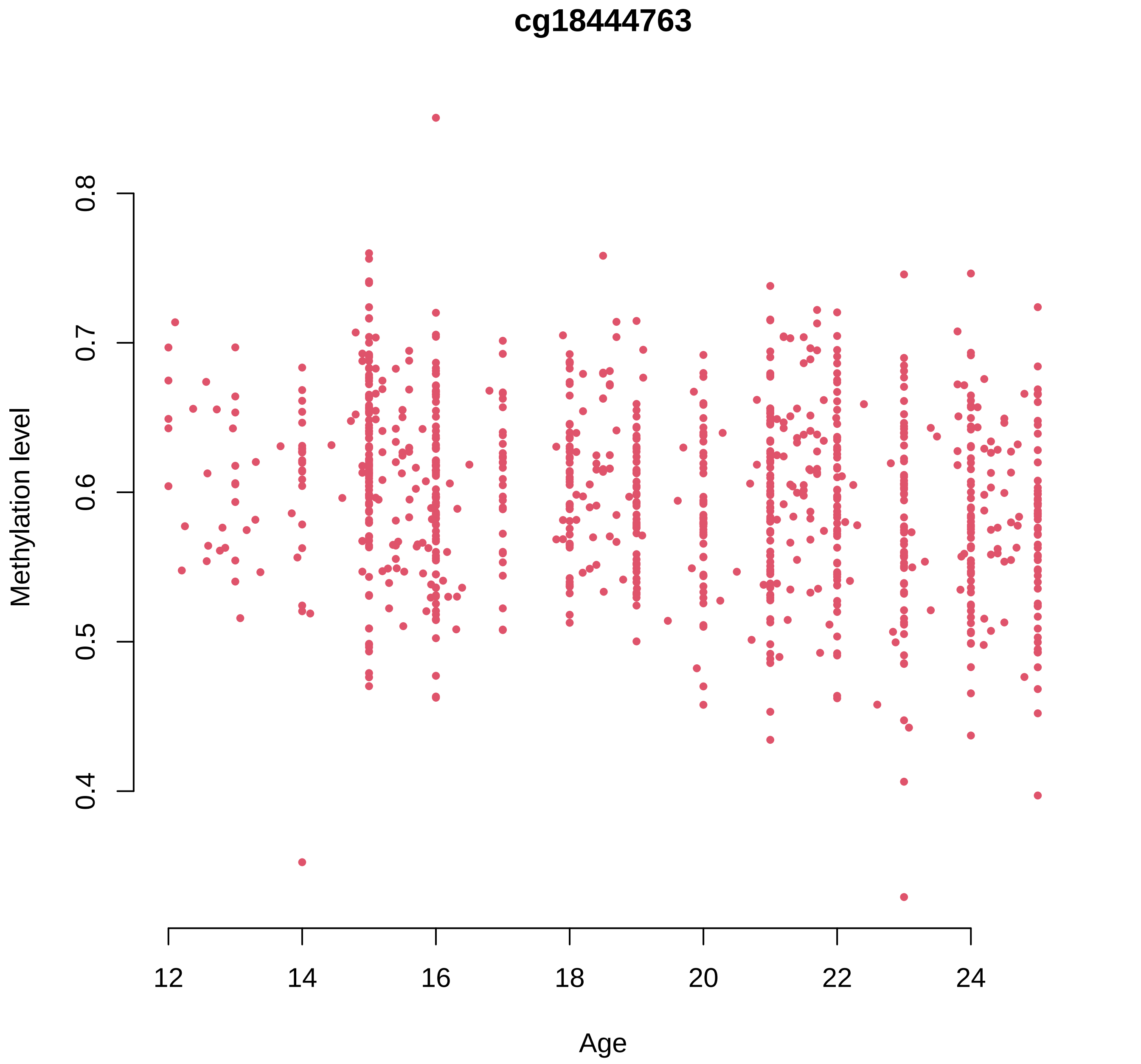

cg18444763
Methylation level
0.4
0.5
0.6
0.7
0.8
12
14
16
18
20
22
24
Age

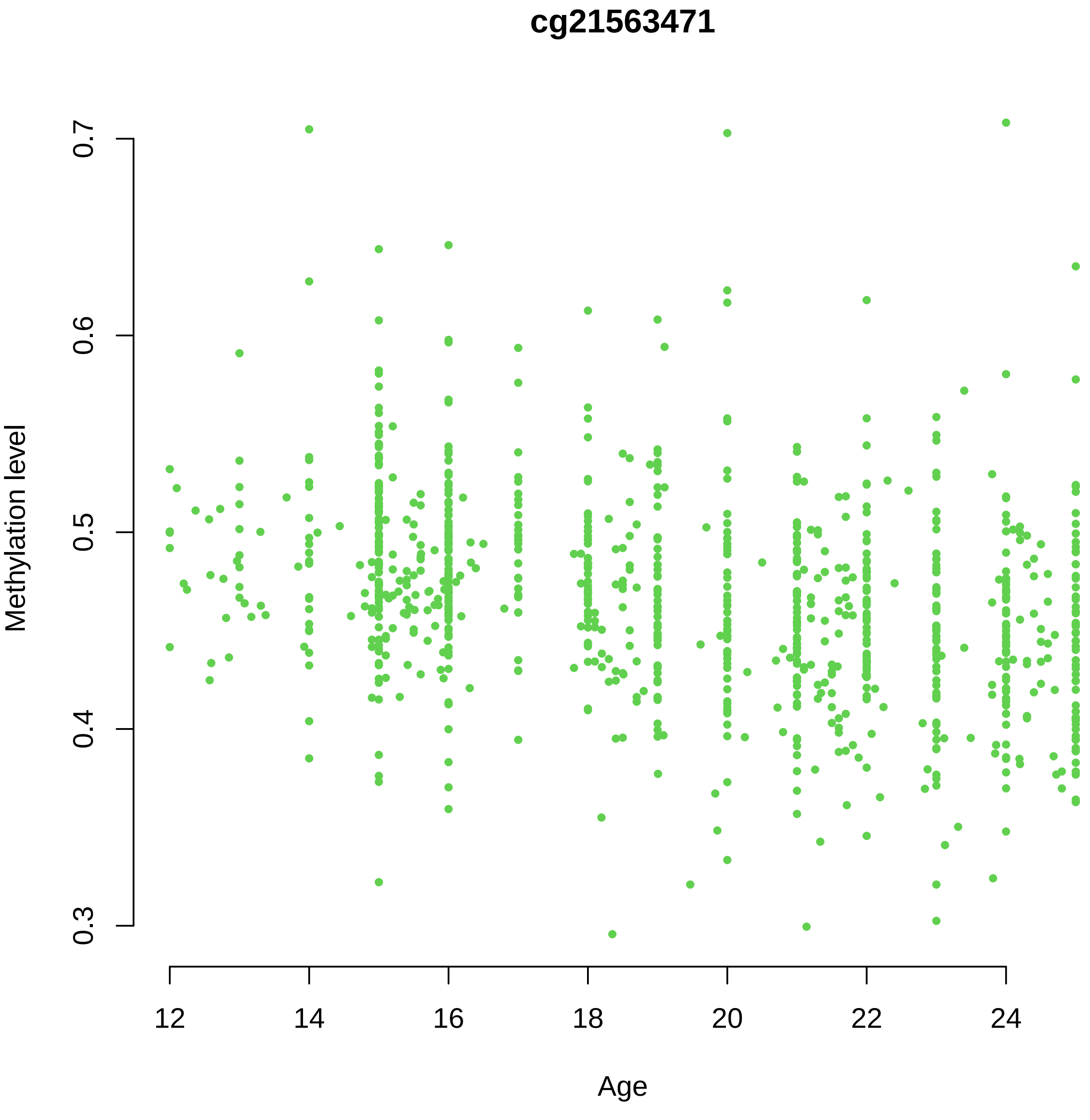
cg21563471
Methylation level
0.3
0.4
0.5
0.6
0.7
Age
12
14
16
18
20
22
24

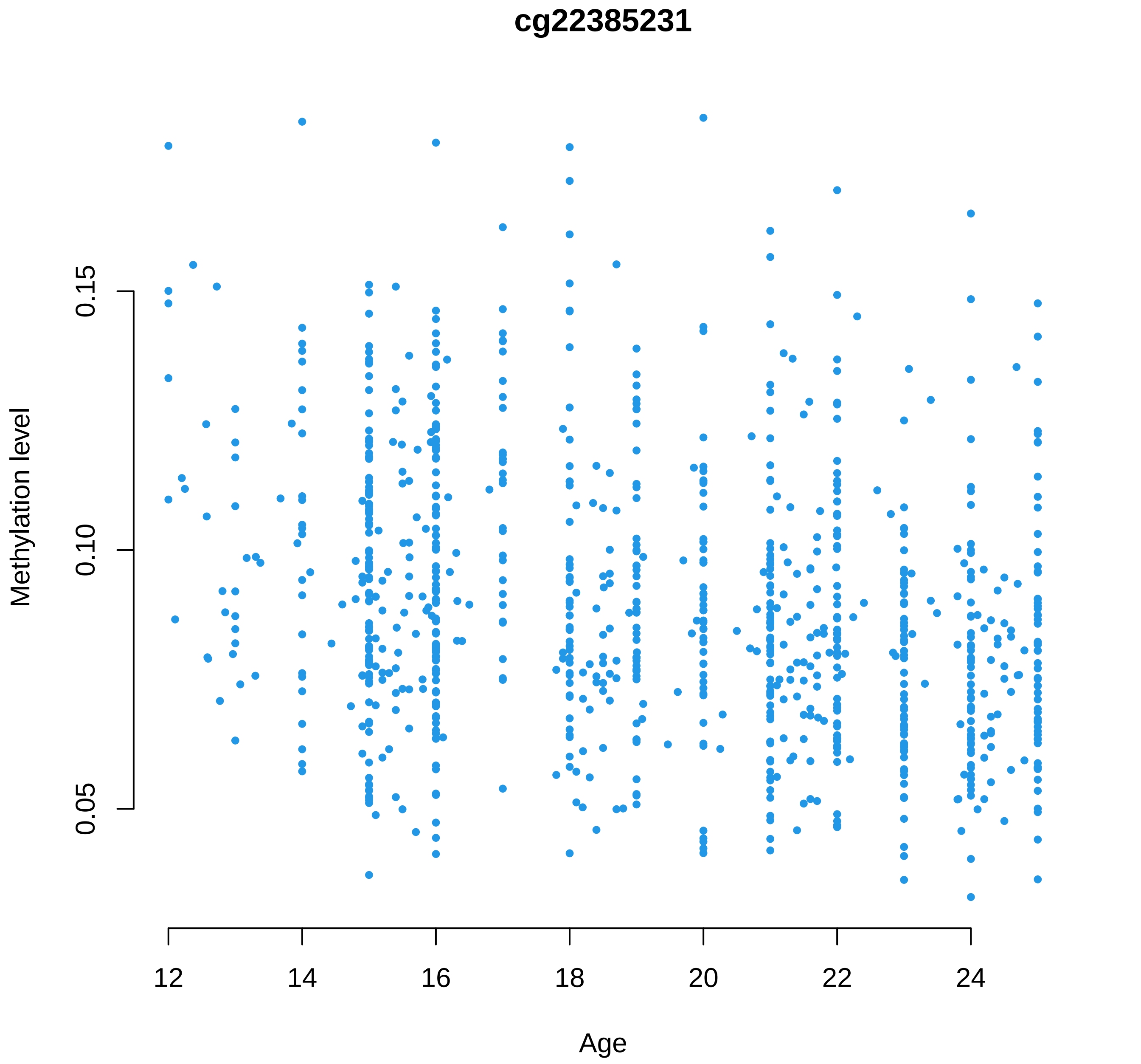

cg22385231
Methylation level
0.05
0.10
0.15
12
14
16
18
20
22
24
Age

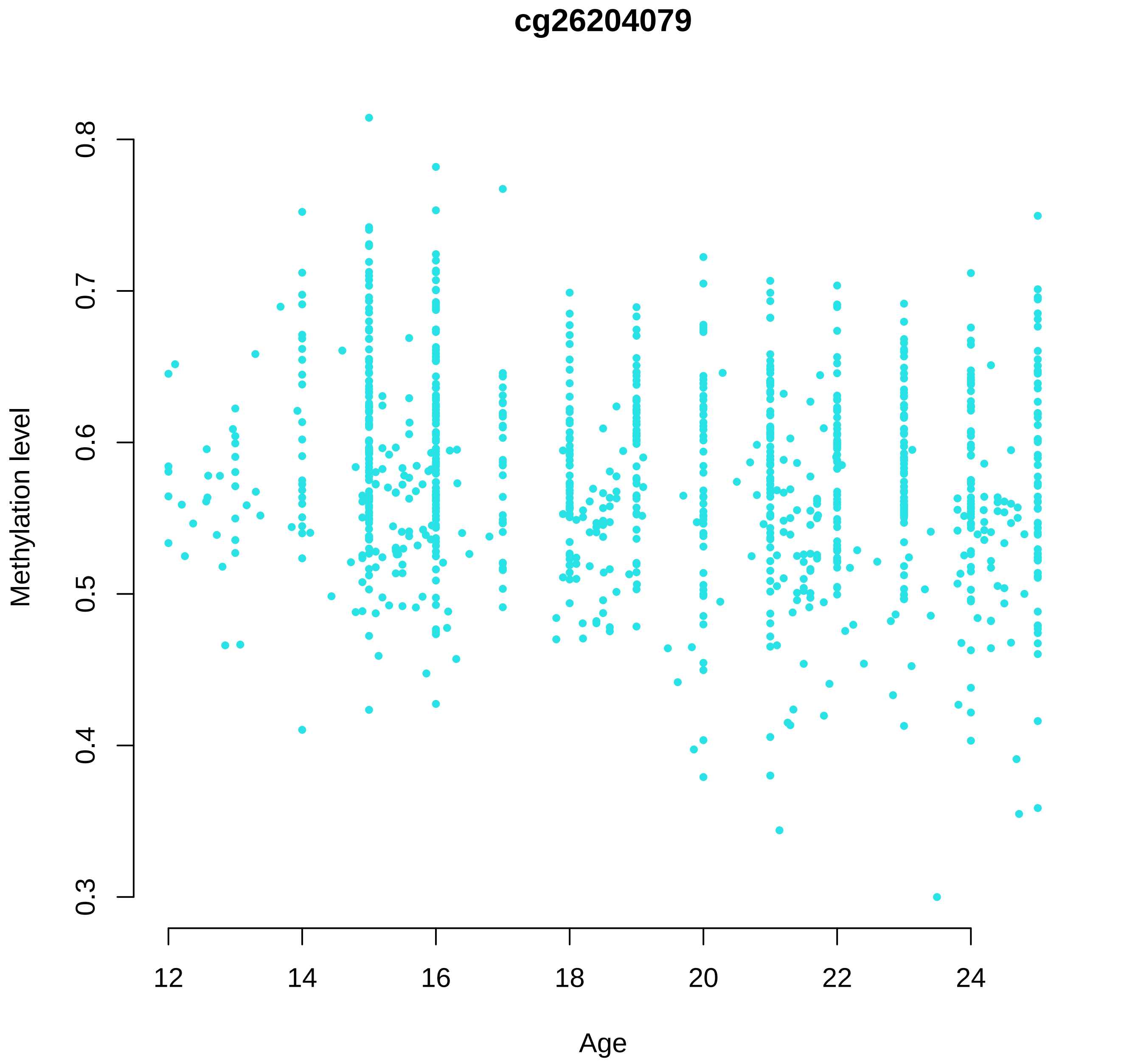
cg26204079
Methylation level
0.3
0.4
0.5
0.6
0.7
0.8
Age
12
14
16
18
20
22
24

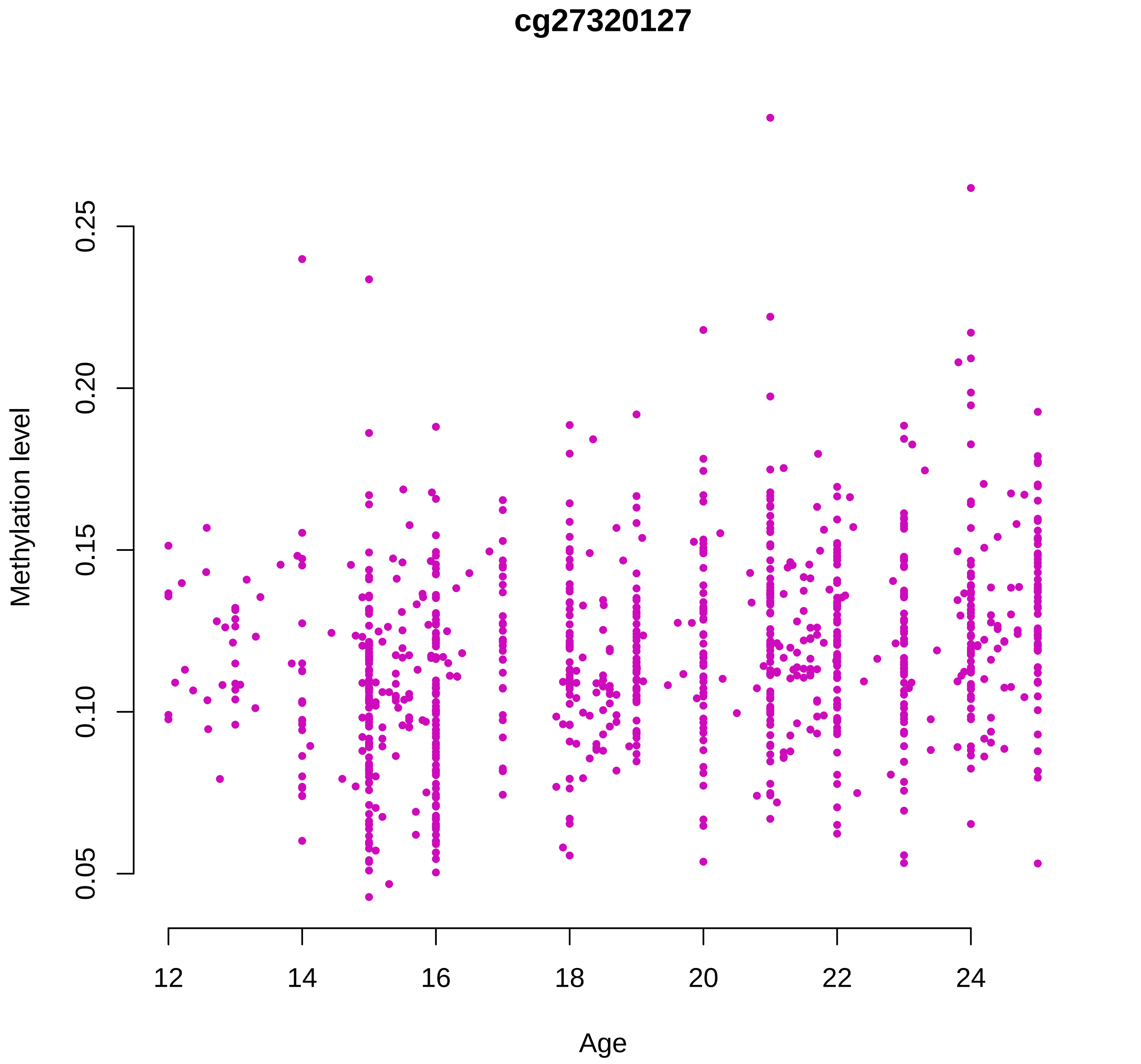
cg27320127
Methylation level
0.05
0.10
0.15
0.20
0.25
Age
12
14
16
18
20
22
24

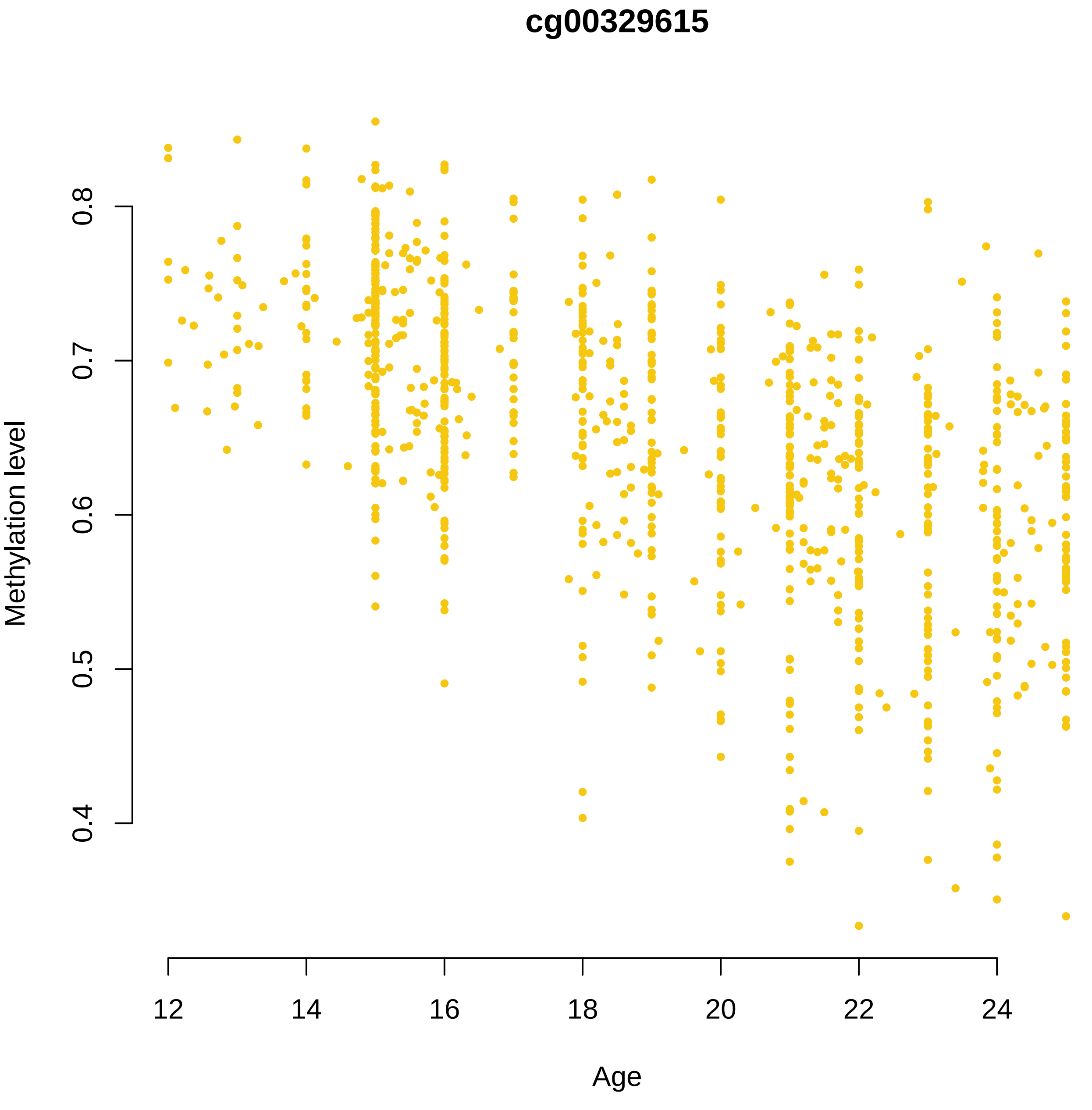

cg00329615
Methylation level
0.4
0.5
0.6
0.7
0.8
Age
12
14
16
18
20
22
24

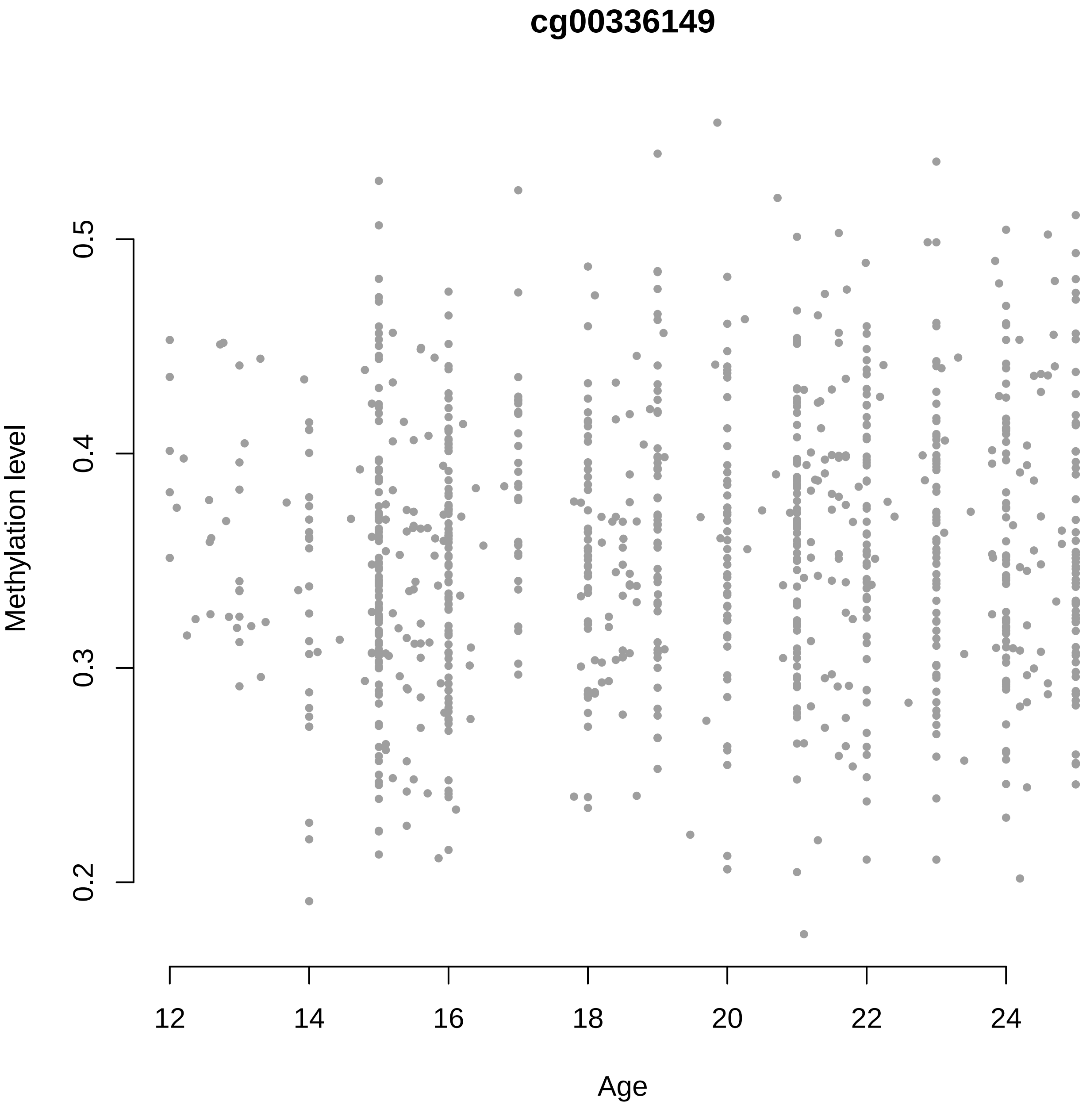
cg00336149
Methylation level
0.2
0.3
0.4
0.5
Age
12
14
16
18
20
22
24

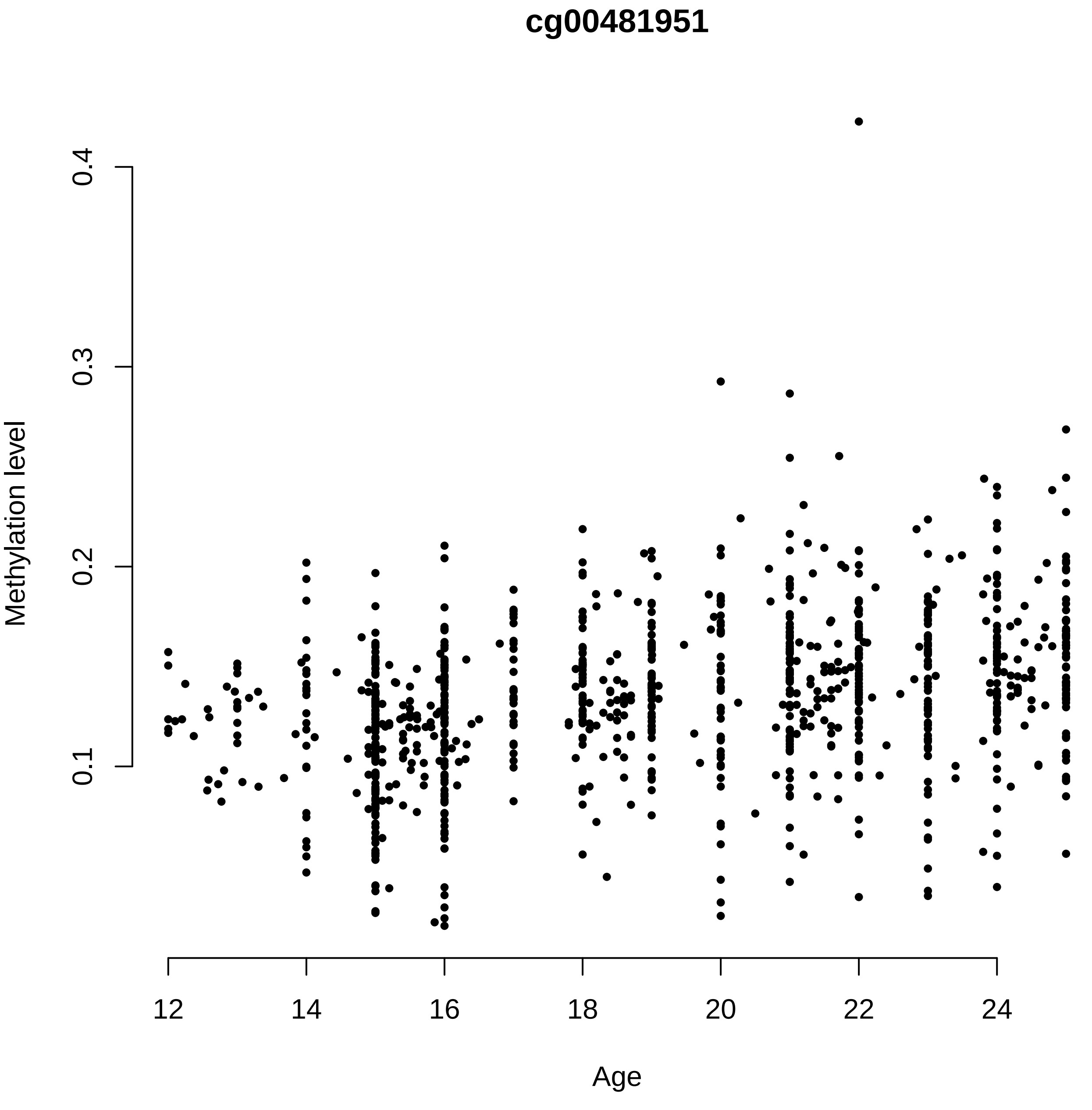

cg00481951
Methylation level
0.1
0.2
0.3
0.4
Age
12
14
16
18
20
22
24

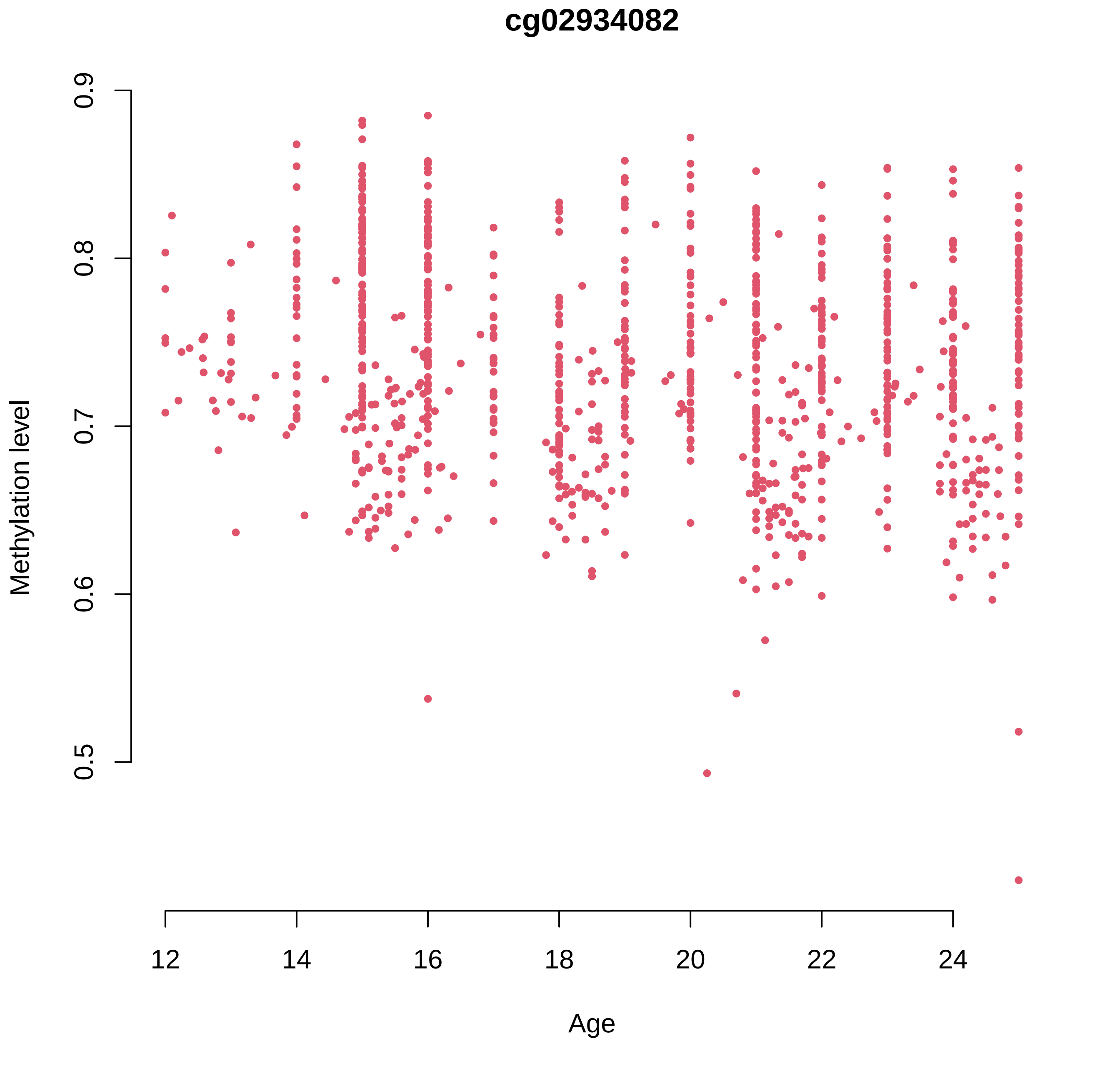
cg02934082
Methylation level
0.5
0.6
0.7
0.8
0.9
Age
12
14
16
18
20
22
24

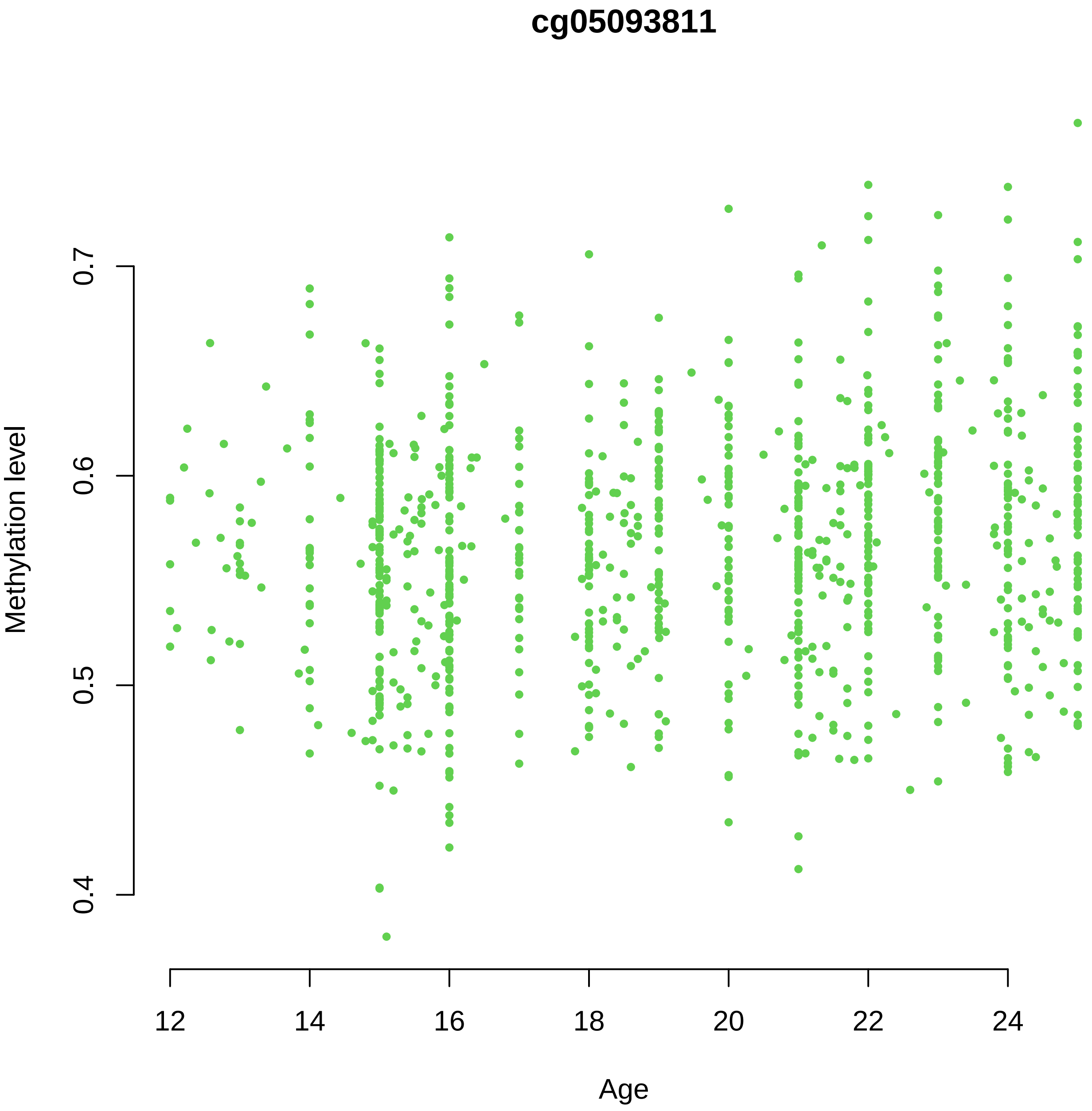
cg05093811
Methylation level
0.4
0.5
0.6
0.7
Age
12
14
16
18
20
22
24

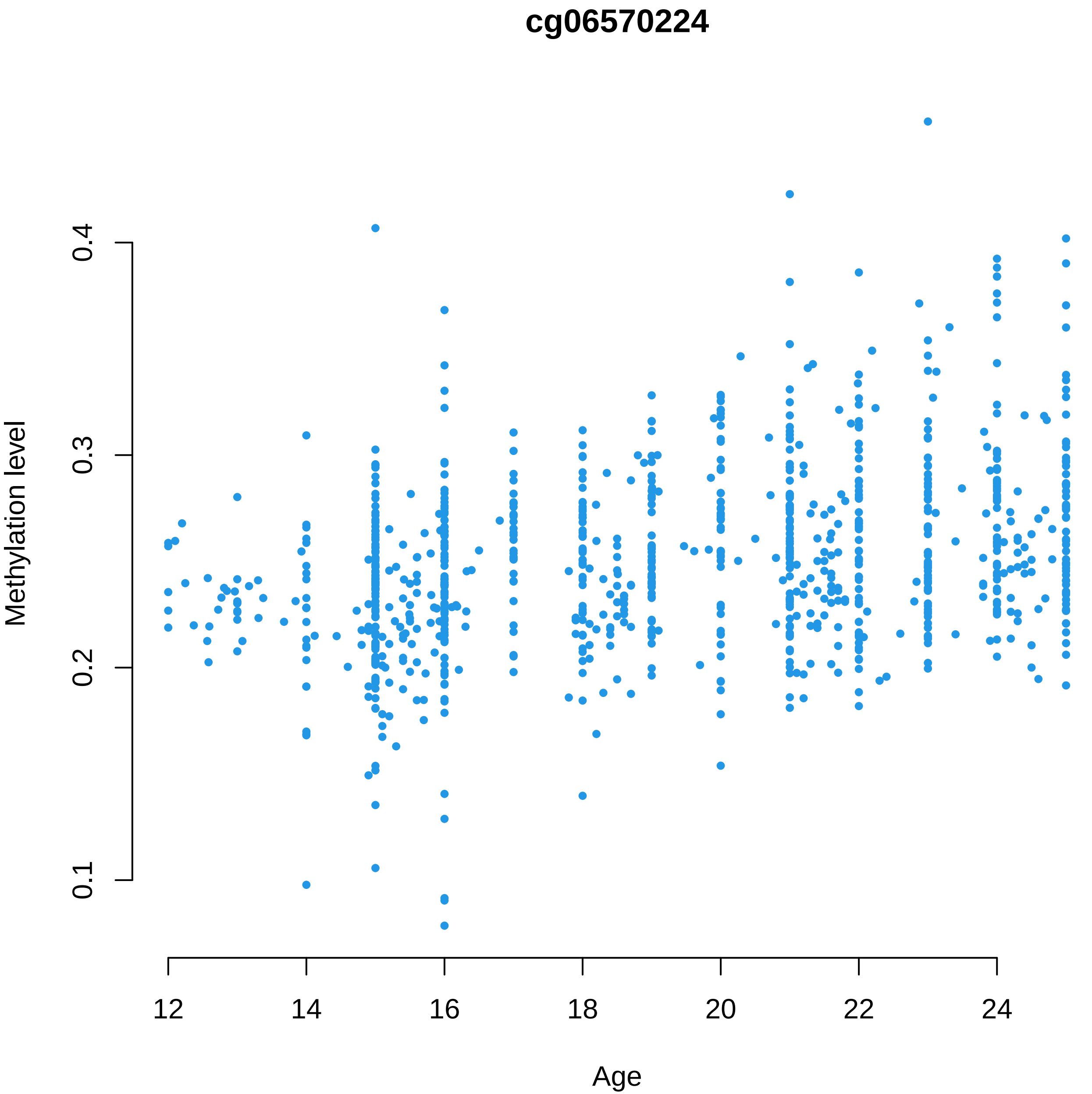

cg06570224
Methylation level
0.1
0.2
0.3
0.4
Age
12
14
16
18
20
22
24

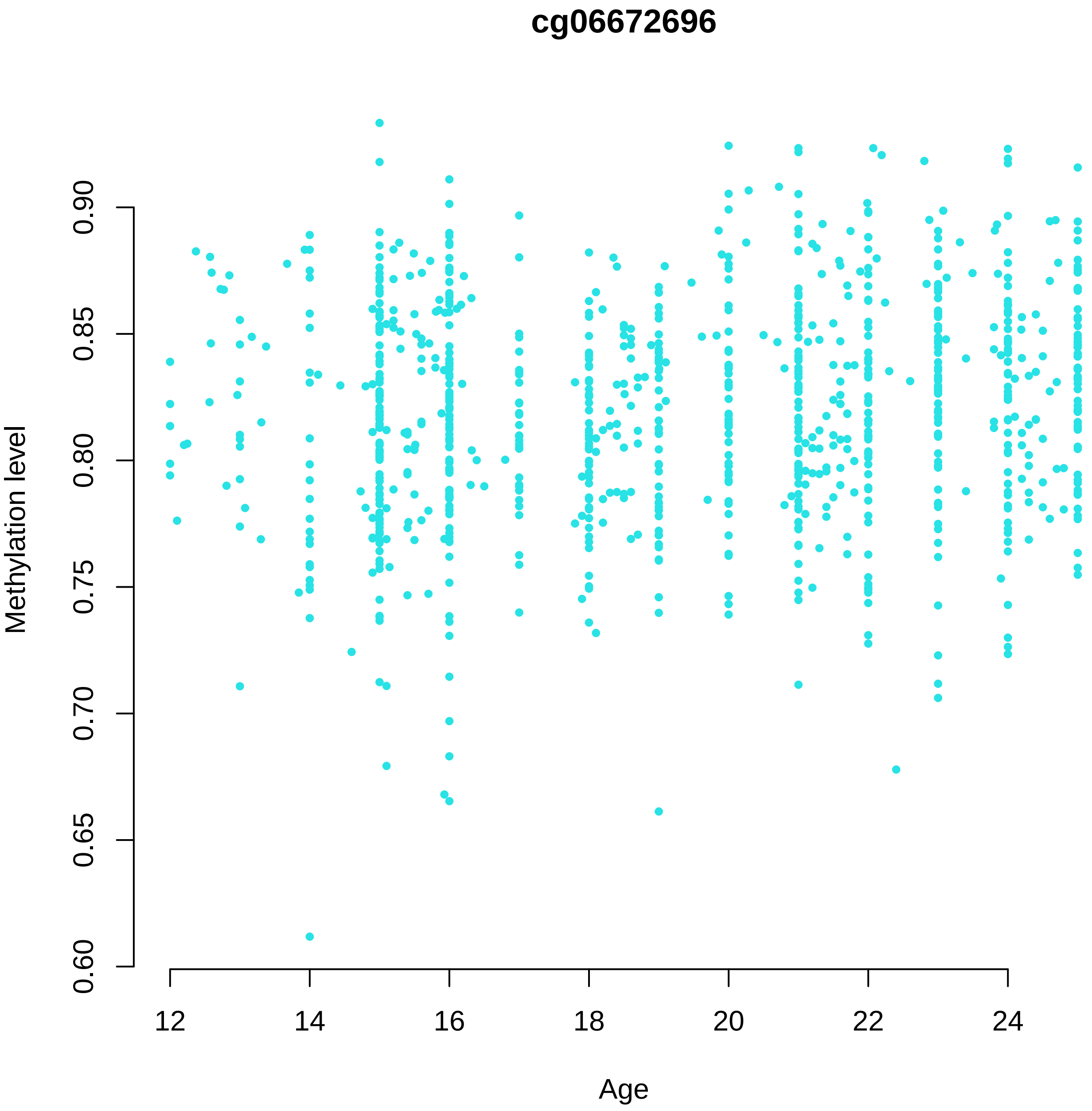
cg06672696
Methylation level
0.60
0.65
0.70
0.75
0.80
0.85
0.90
Age
12
14
16
18
20
22
24

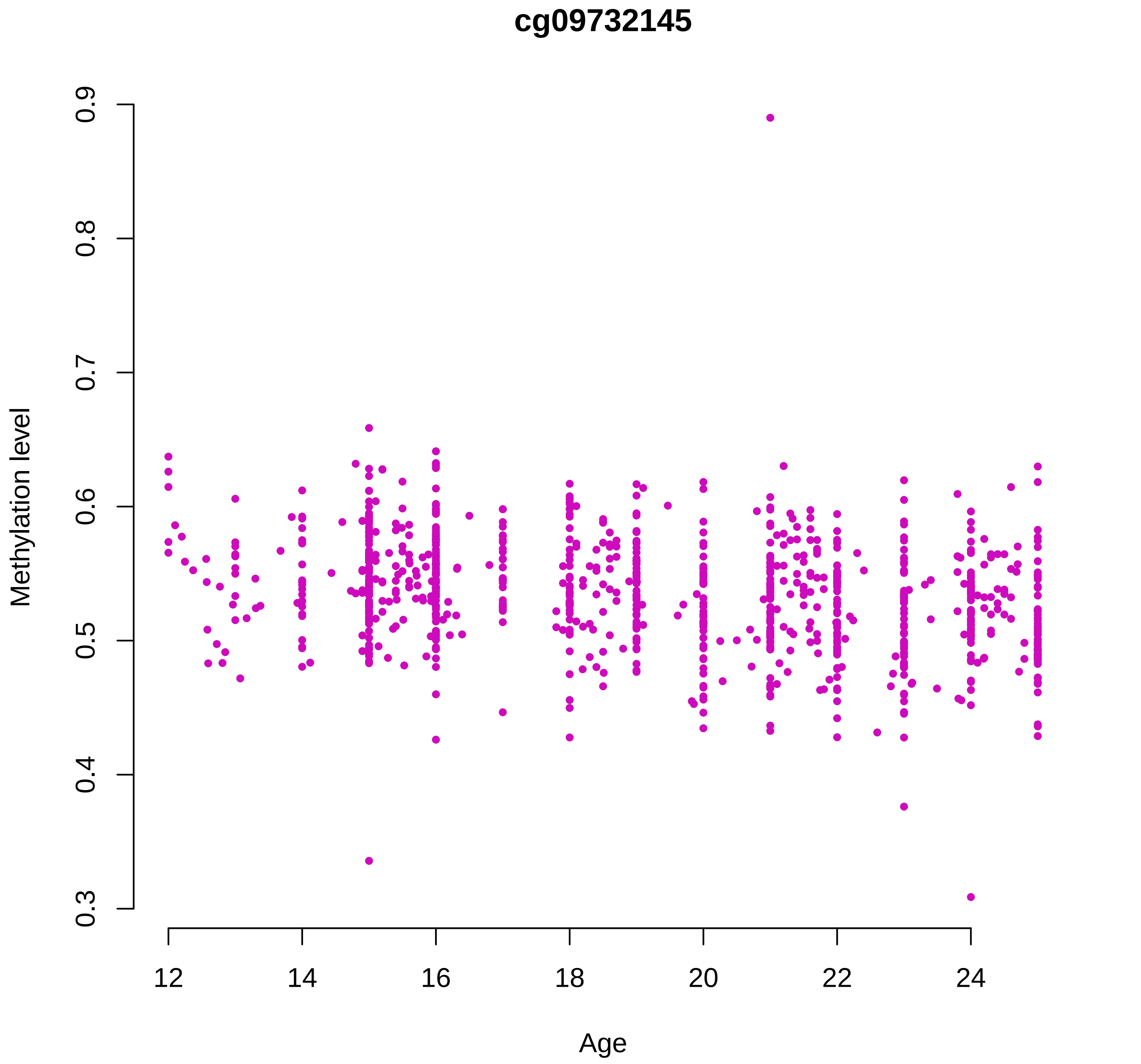

cg09732145
Methylation level
0.3
0.4
0.5
0.6
0.7
0.8
0.9
Age
12
14
16
18
20
22
24

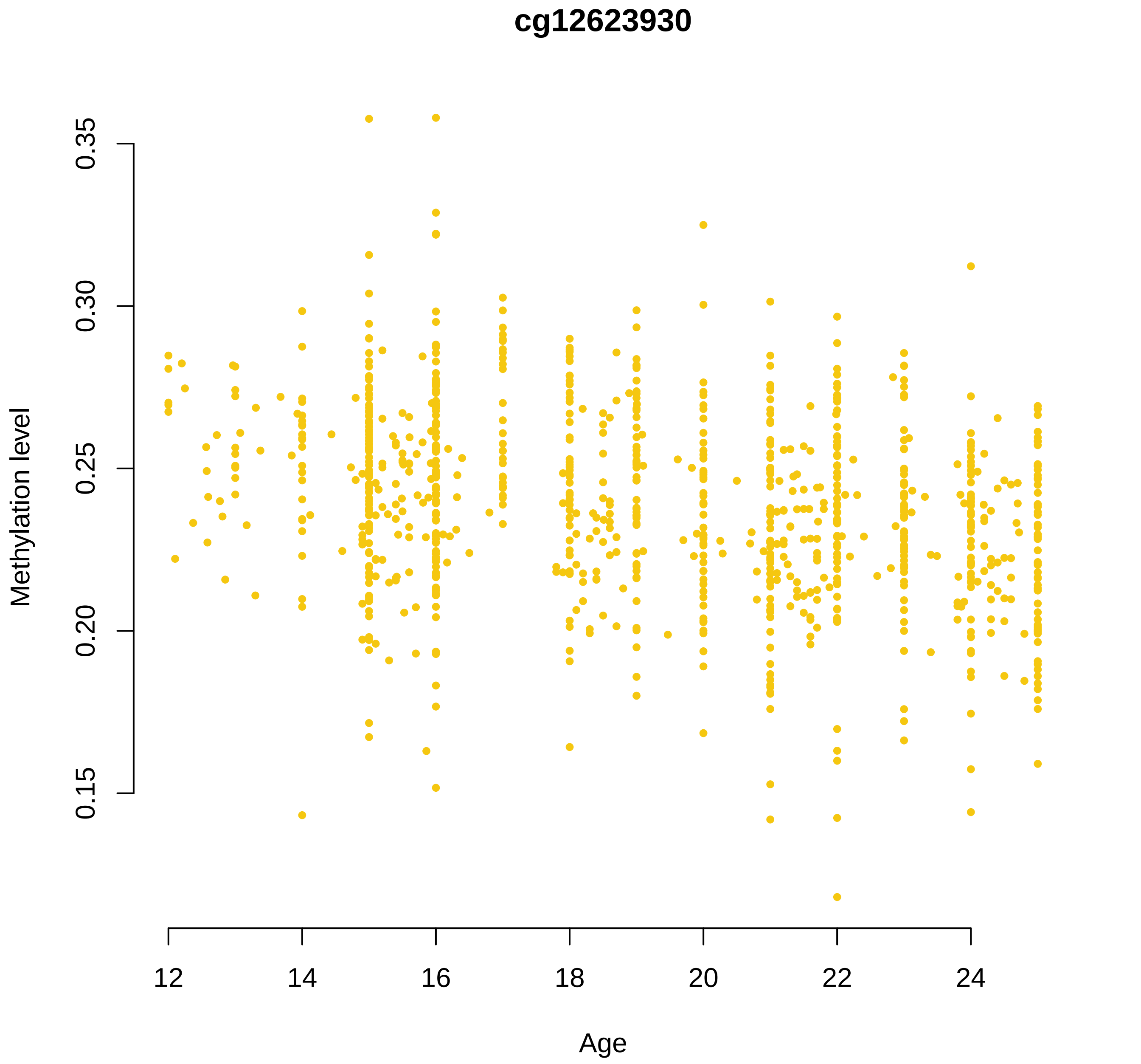
cg12623930
Methylation level
0.15
0.20
0.25
0.30
0.35
Age
12
14
16
18
20
22
24

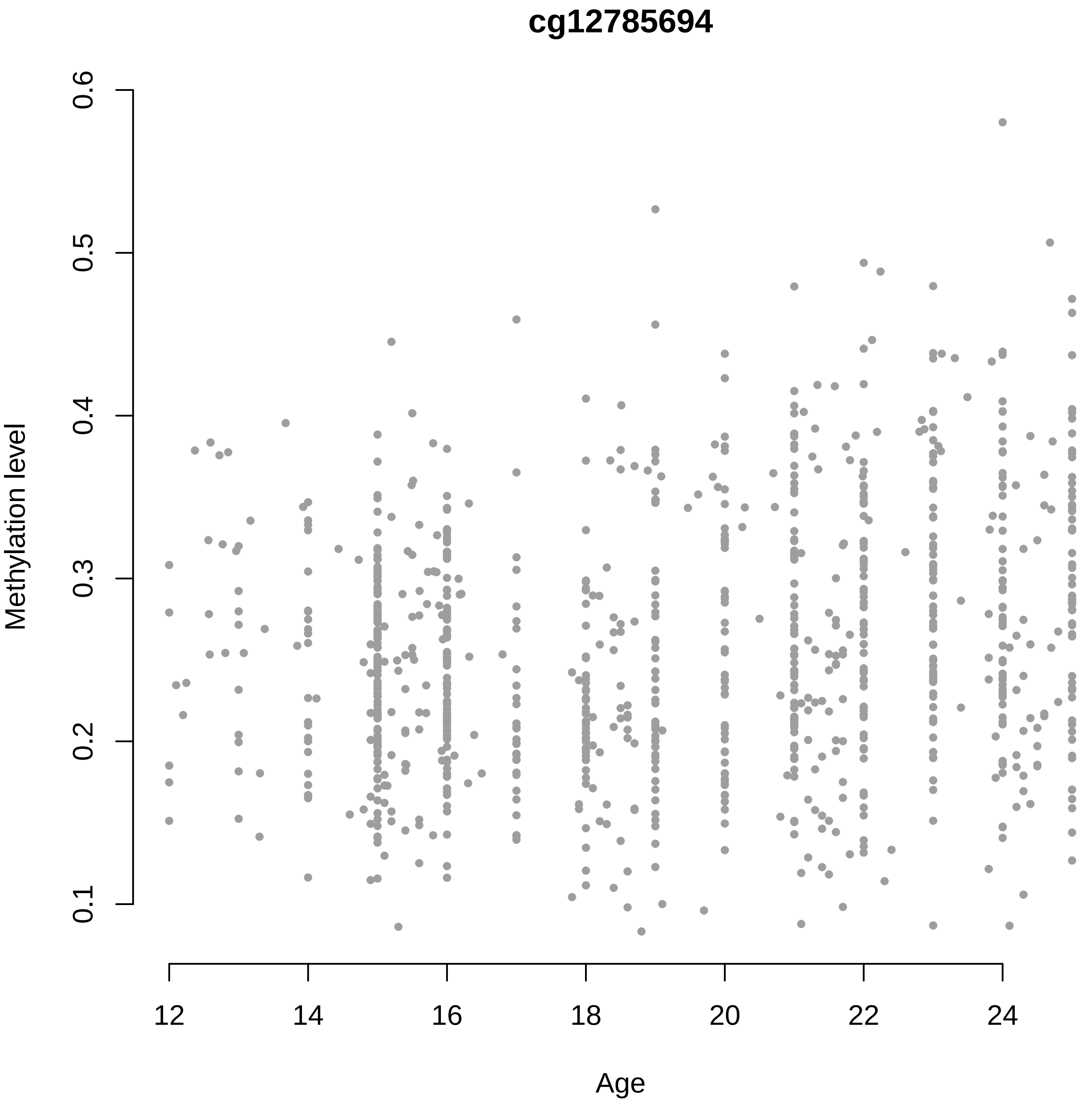
cg12785694
Methylation level
0.1
0.2
0.3
0.4
0.5
0.6
Age
12
14
16
18
20
22
24

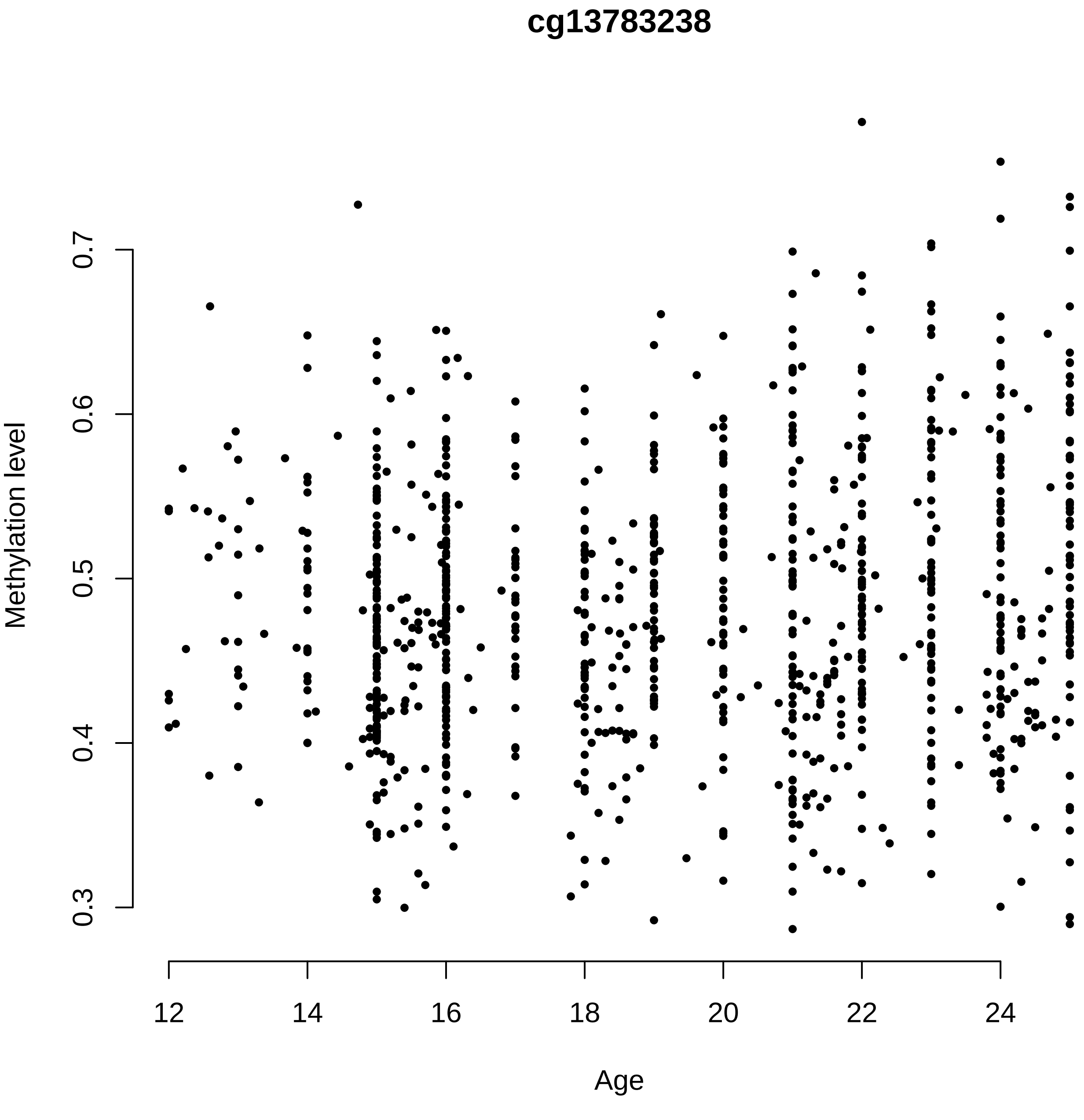
cg13783238
Methylation level
0.3
0.4
0.5
0.6
0.7
Age
12
14
16
18
20
22
24

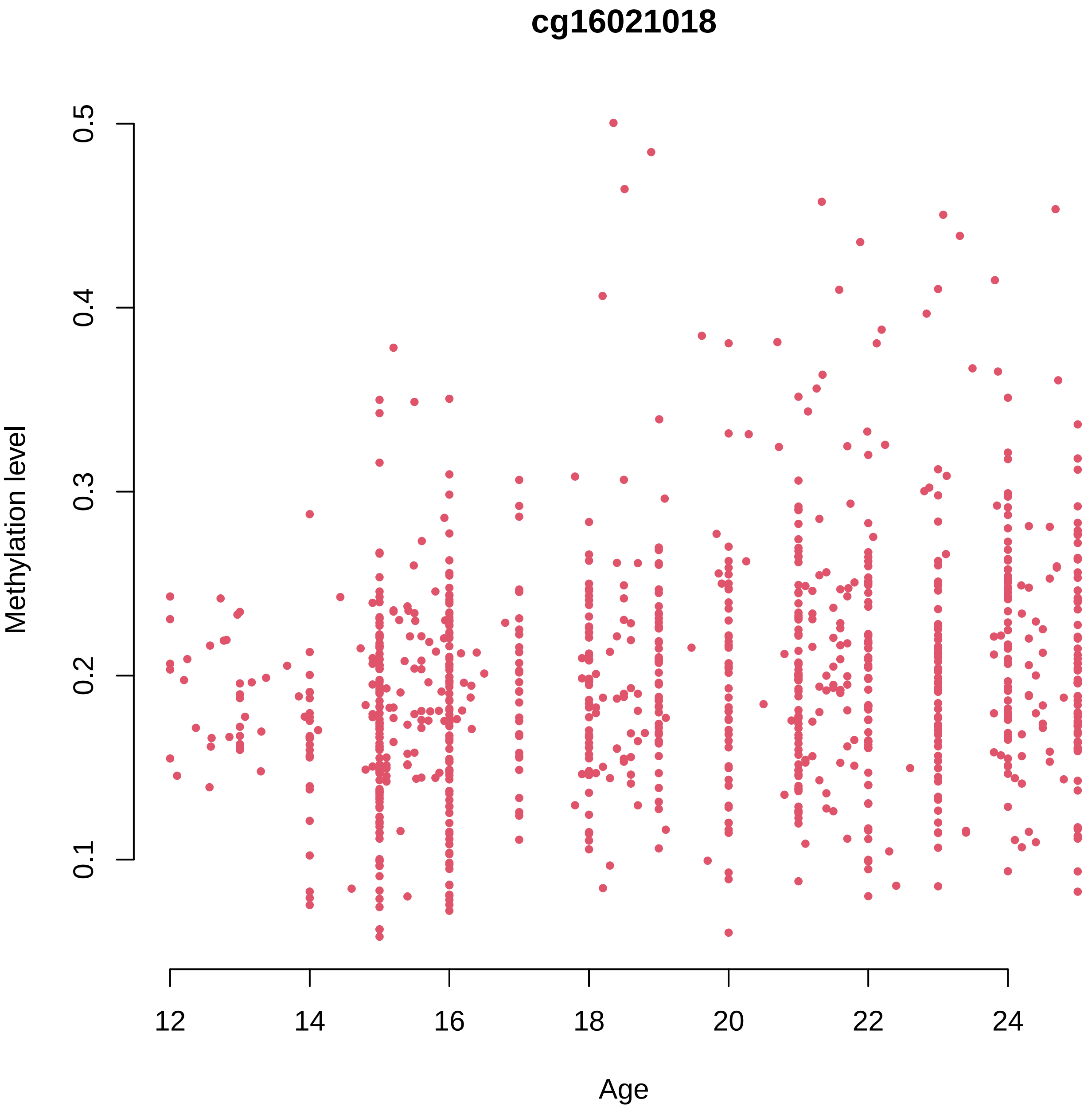

cg16021018
Methylation level
0.1
0.2
0.3
0.4
0.5
Age
12
14
16
18
20
22
24

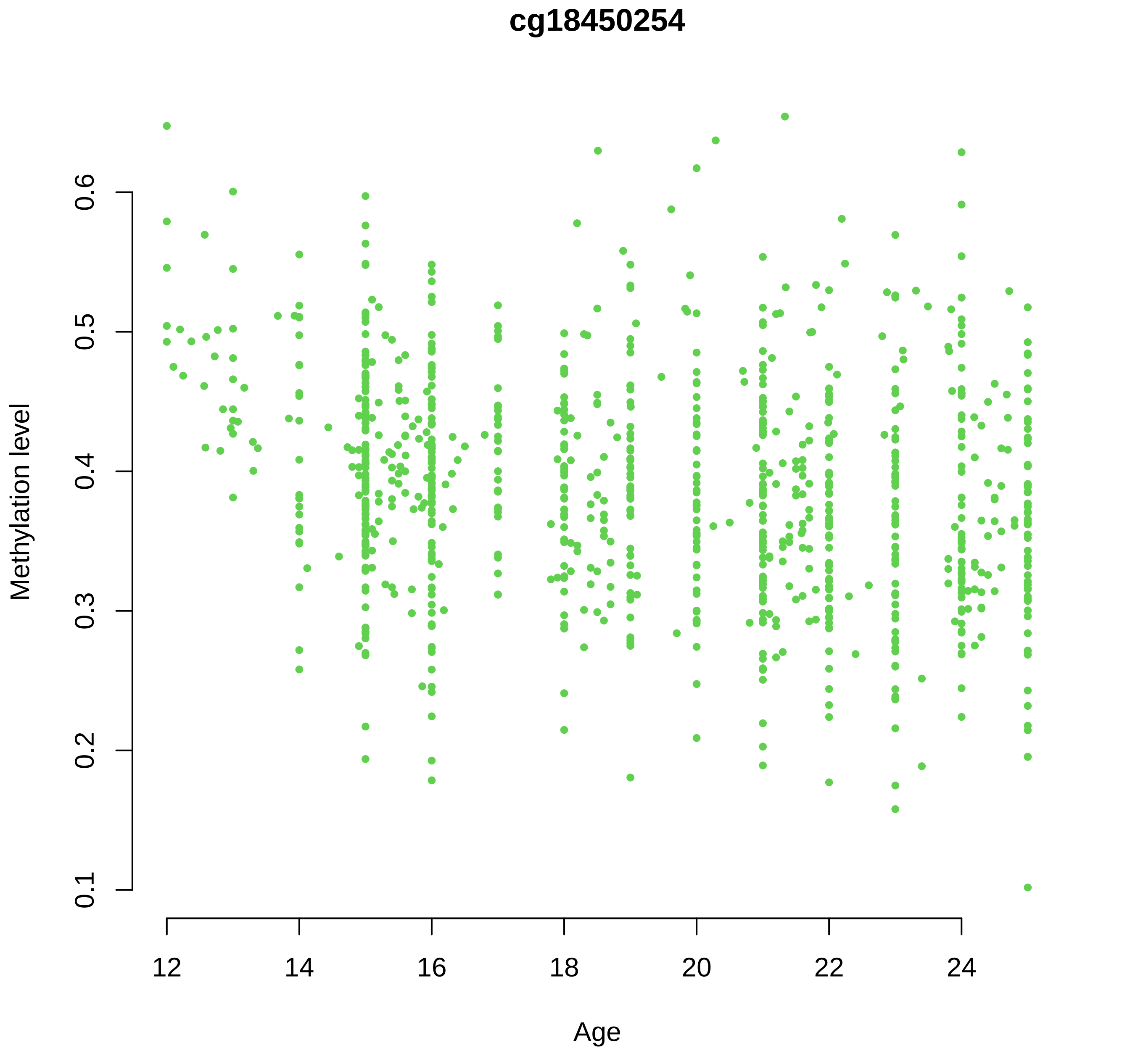

cg18450254
Methylation level
0.1
0.2
0.3
0.4
0.5
0.6
Age
12
14
16
18
20
22
24

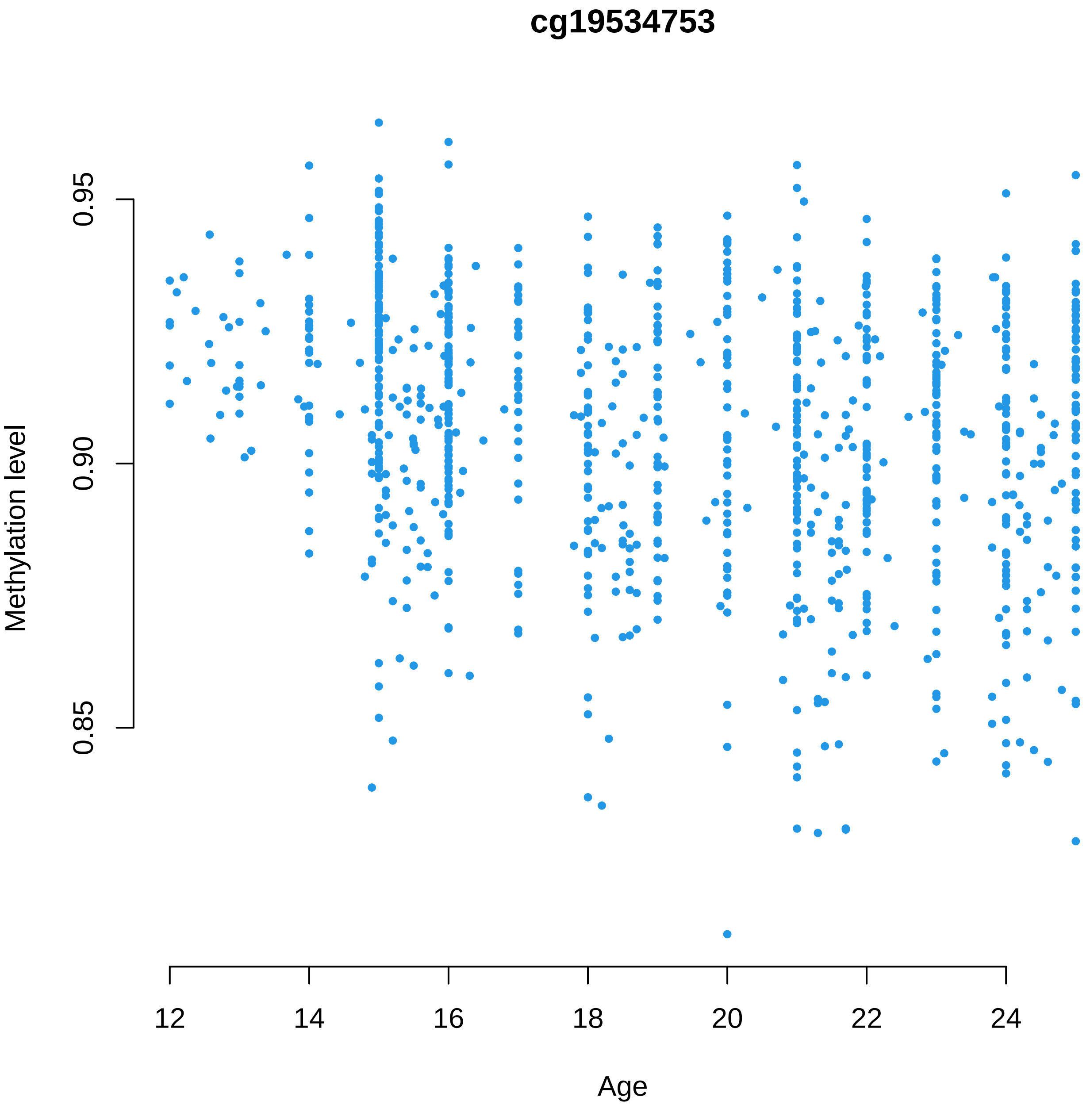
cg19534753
Methylation level
0.85
0.90
0.95
12
14
16
18
20
22
24
Age

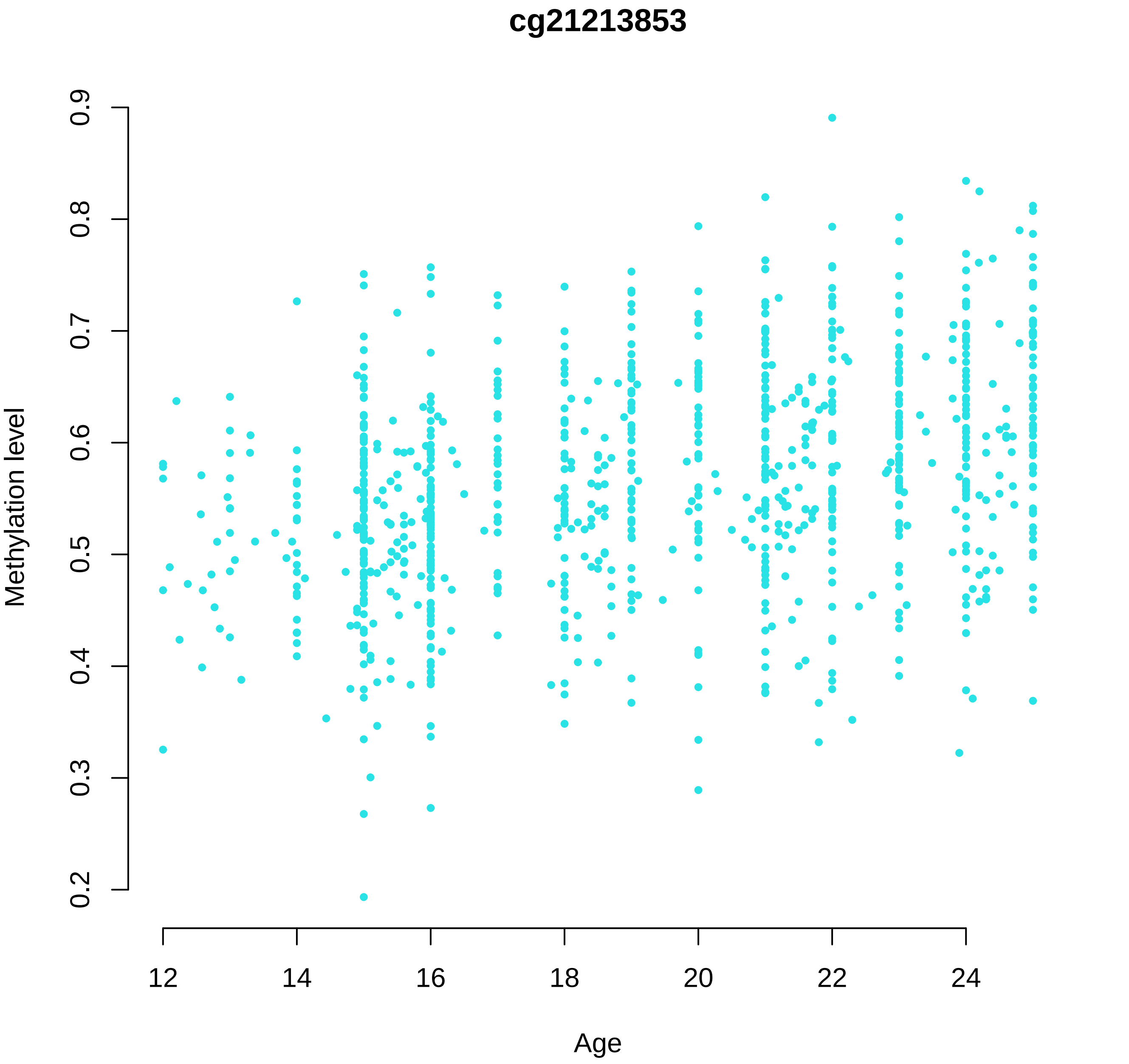

cg21213853
Methylation level
0.2
0.3
0.4
0.5
0.6
0.7
0.8
0.9
Age
12
14
16
18
20
22
24

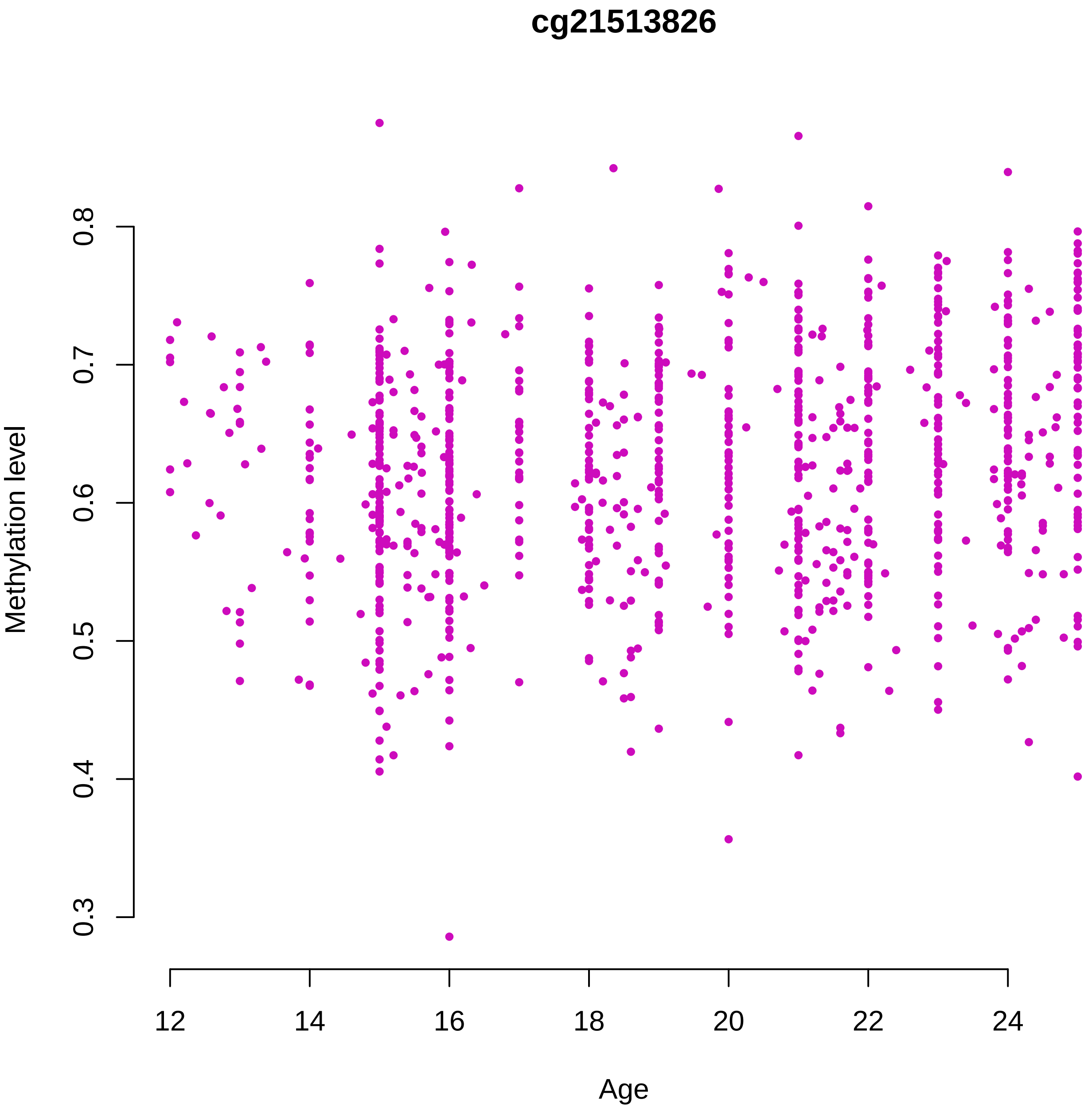
cg21513826
Methylation level
0.3
0.4
0.5
0.6
0.7
0.8
Age
12
14
16
18
20
22
24

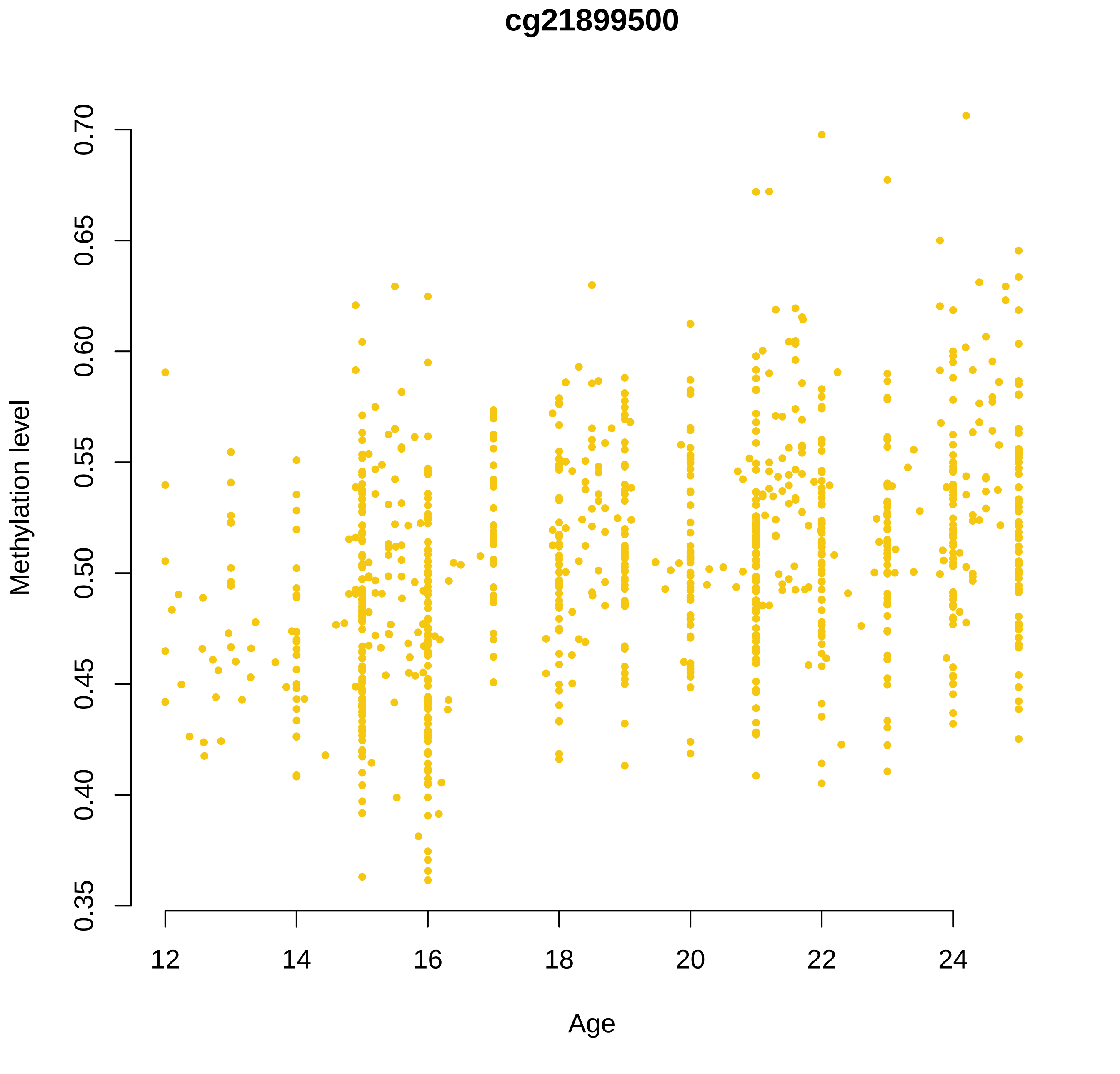
cg21899500
Methylation level
0.35
0.40
0.45
0.50
0.55
0.60
0.65
0.70
12
14
16
18
20
22
24
Age

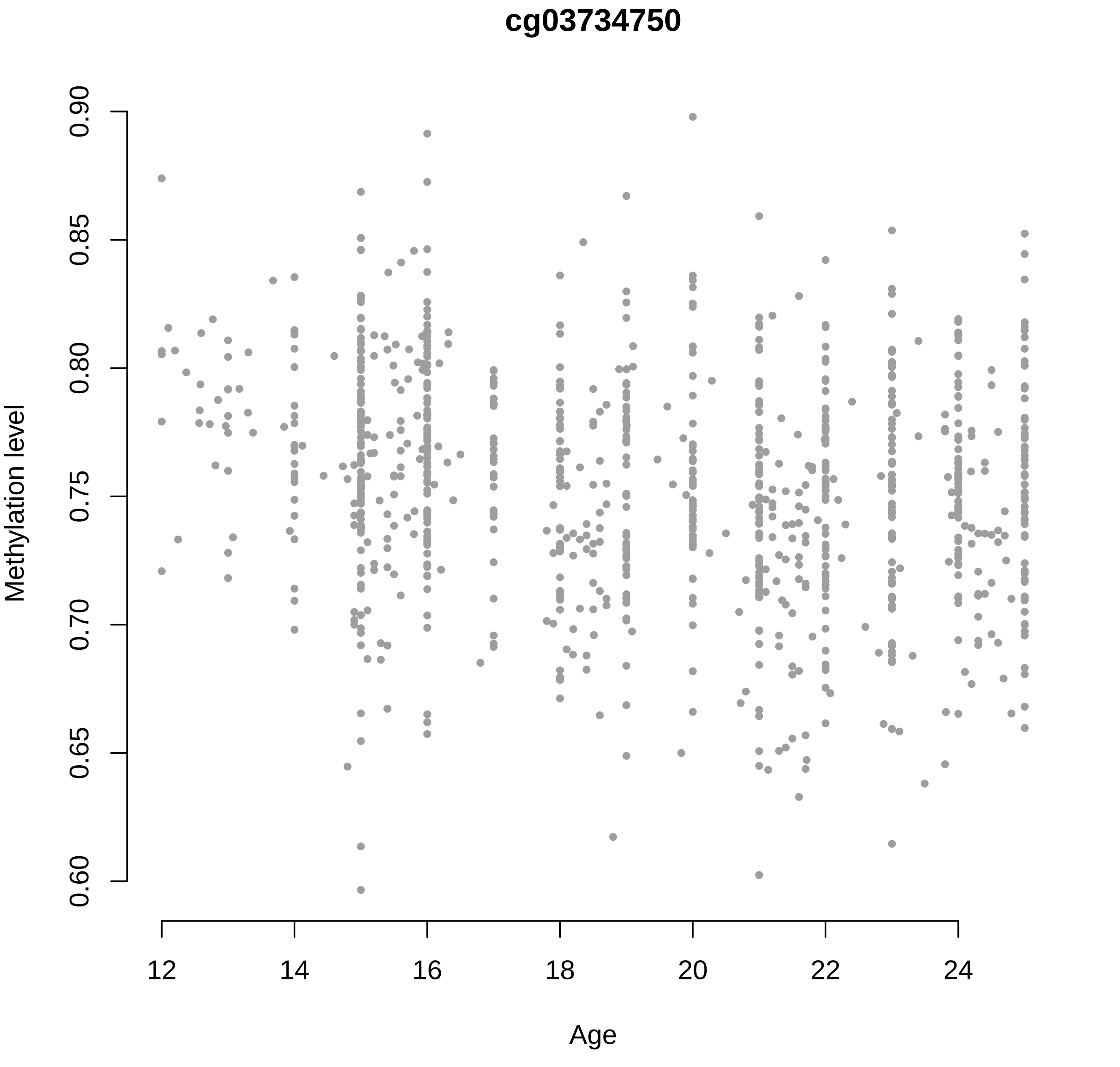
cg03734750
Methylation level
0.60
0.65
0.70
0.75
0.80
0.85
0.90
12
14
16
18
20
22
24
Age

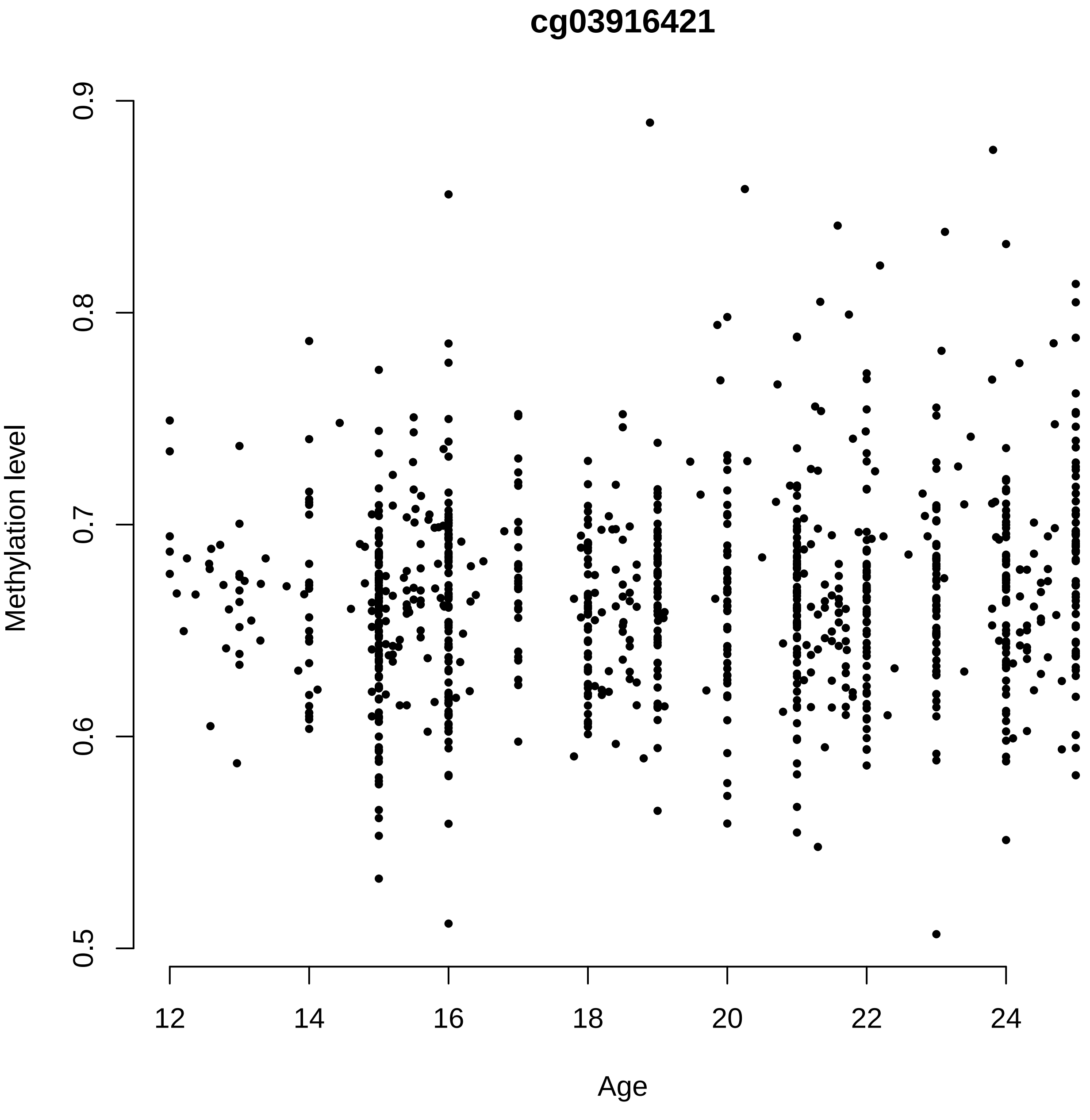
cg03916421
Methylation level
0.5
0.6
0.7
0.8
0.9
Age
12
14
16
18
20
22
24

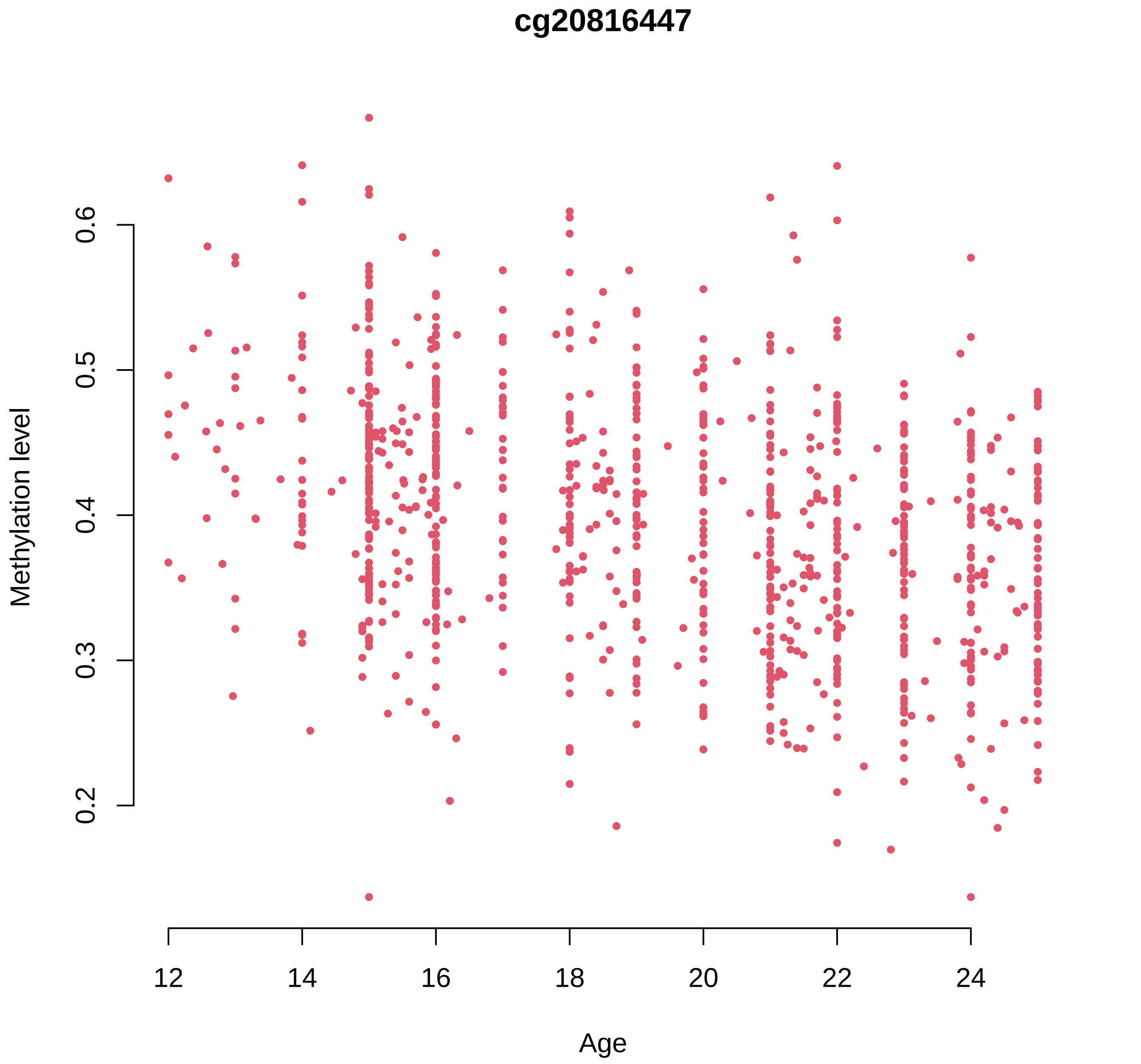
cg20816447
Methylation level
0.2
0.3
0.4
0.5
0.6
12
14
16
18
20
22
24
Age

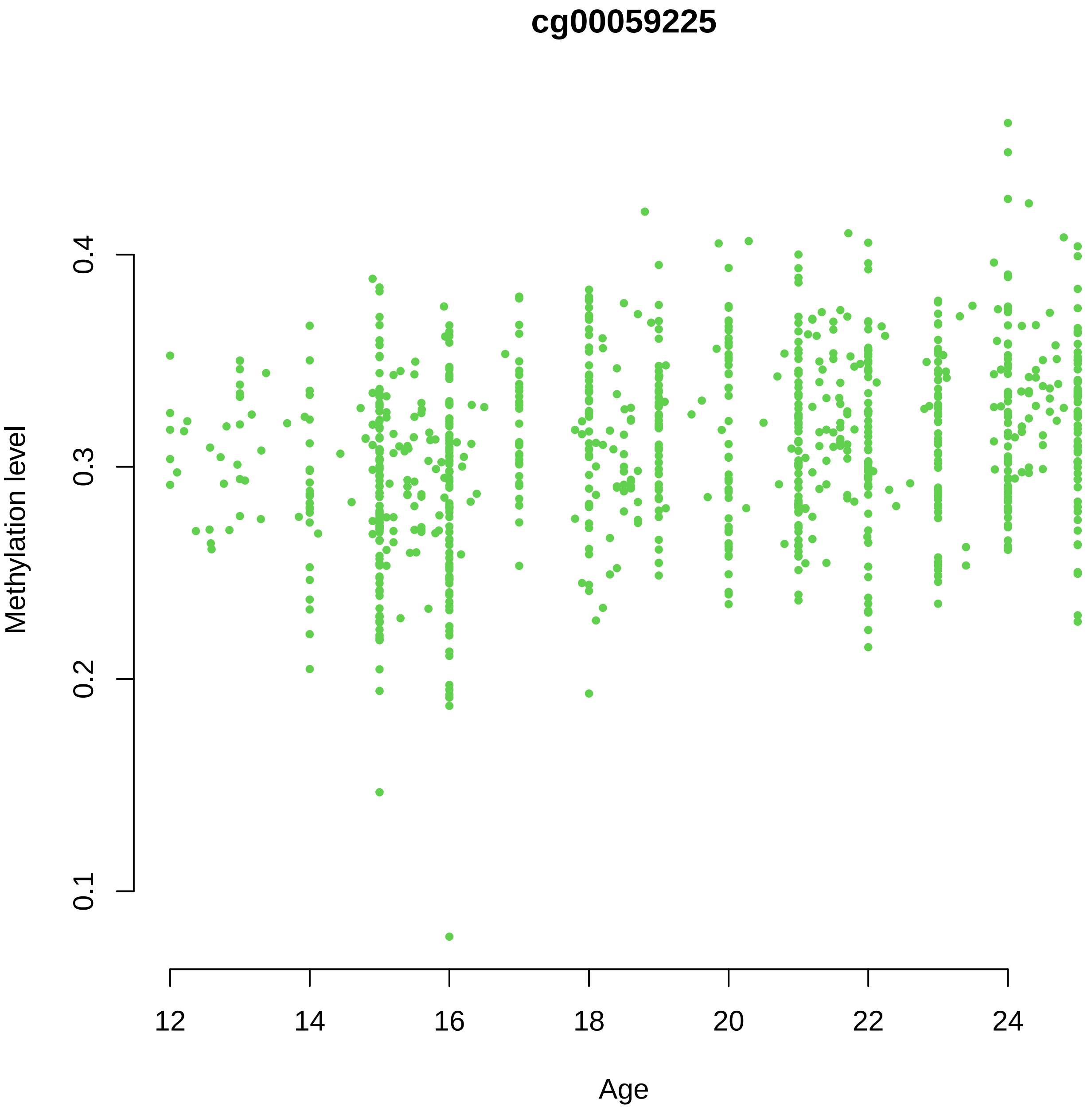
cg00059225
Methylation level
0.1
0.2
0.3
0.4
Age
12
14
16
18
20
22
24

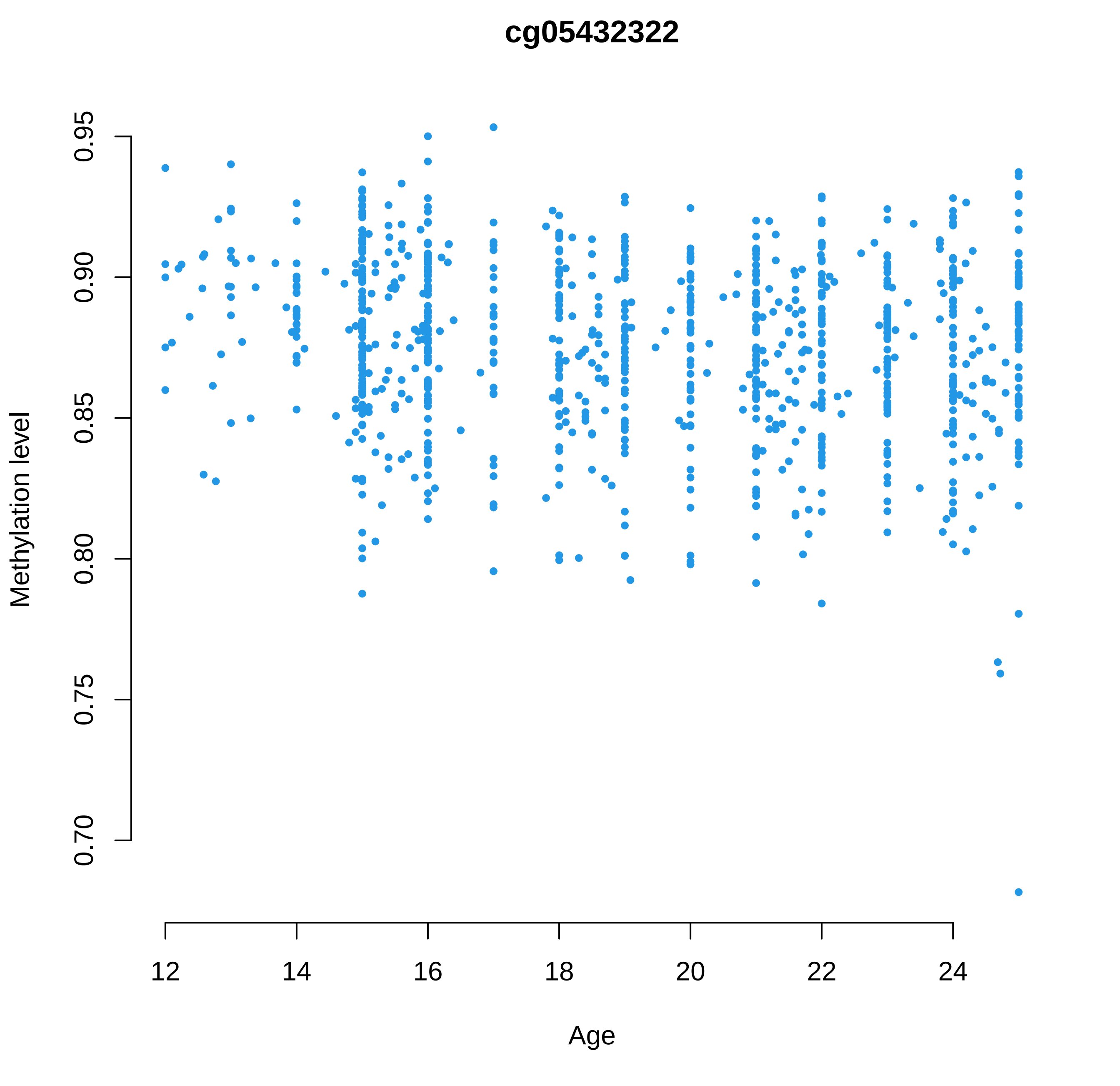
cg05432322
Methylation level
0.95
0.90
0.85
0.80
0.75
0.70
12
14
16
18
20
22
24
Age

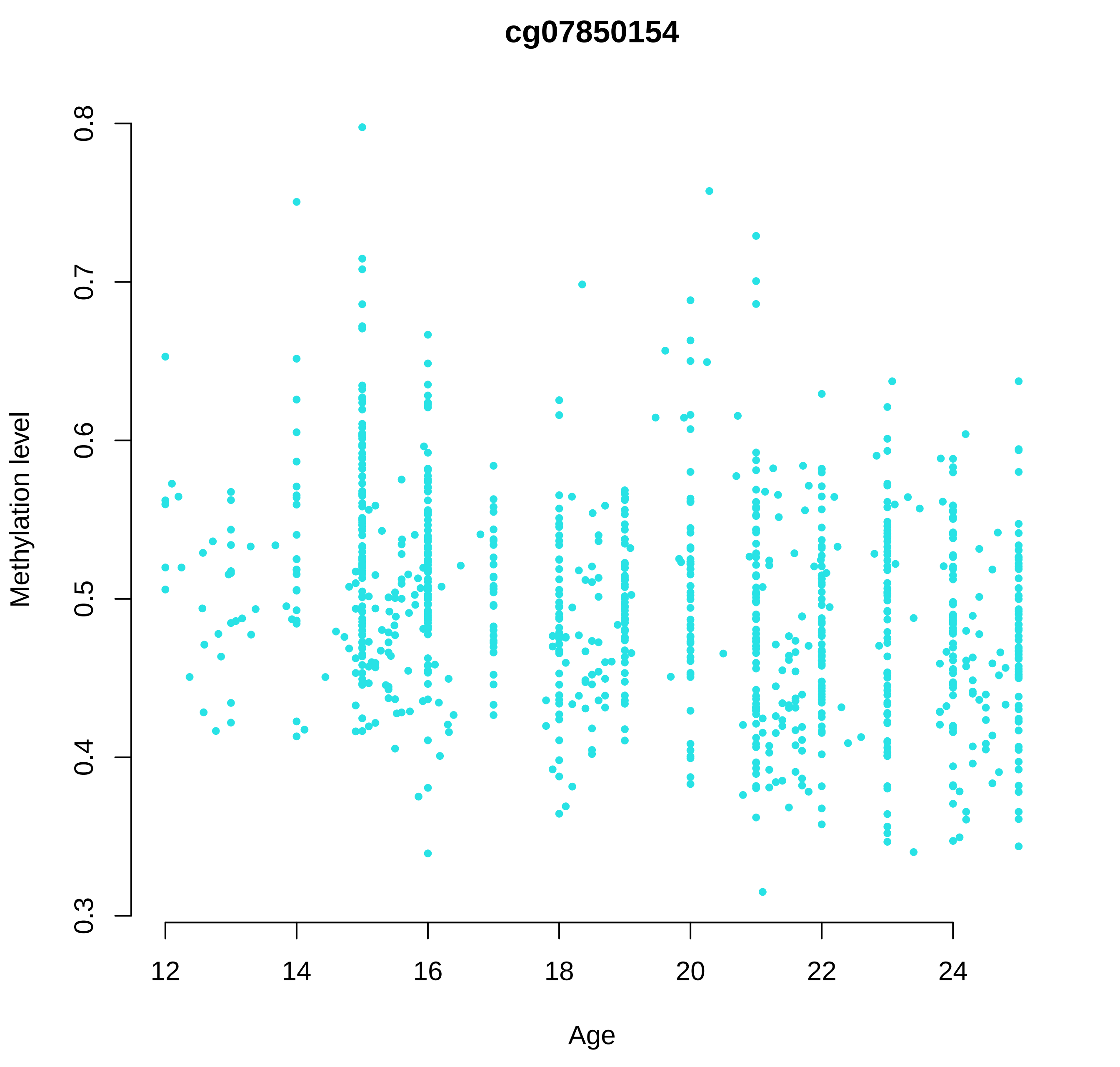

cg07850154
Methylation level
0.3
0.4
0.5
0.6
0.7
0.8
12
14
16
18
20
22
24
Age

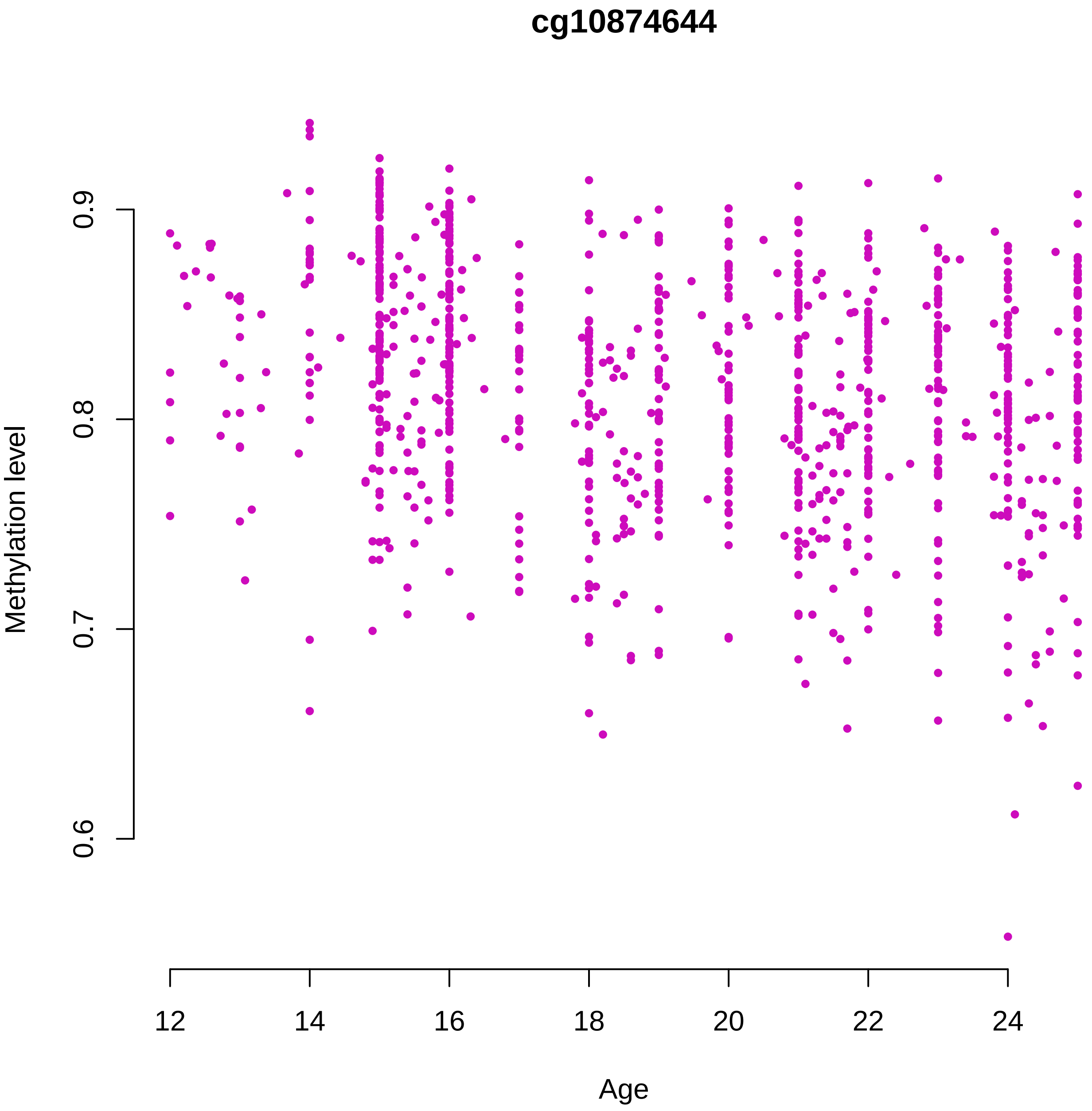

cg10874644
Methylation level
0.6
0.7
0.8
0.9
Age
12
14
16
18
20
22
24

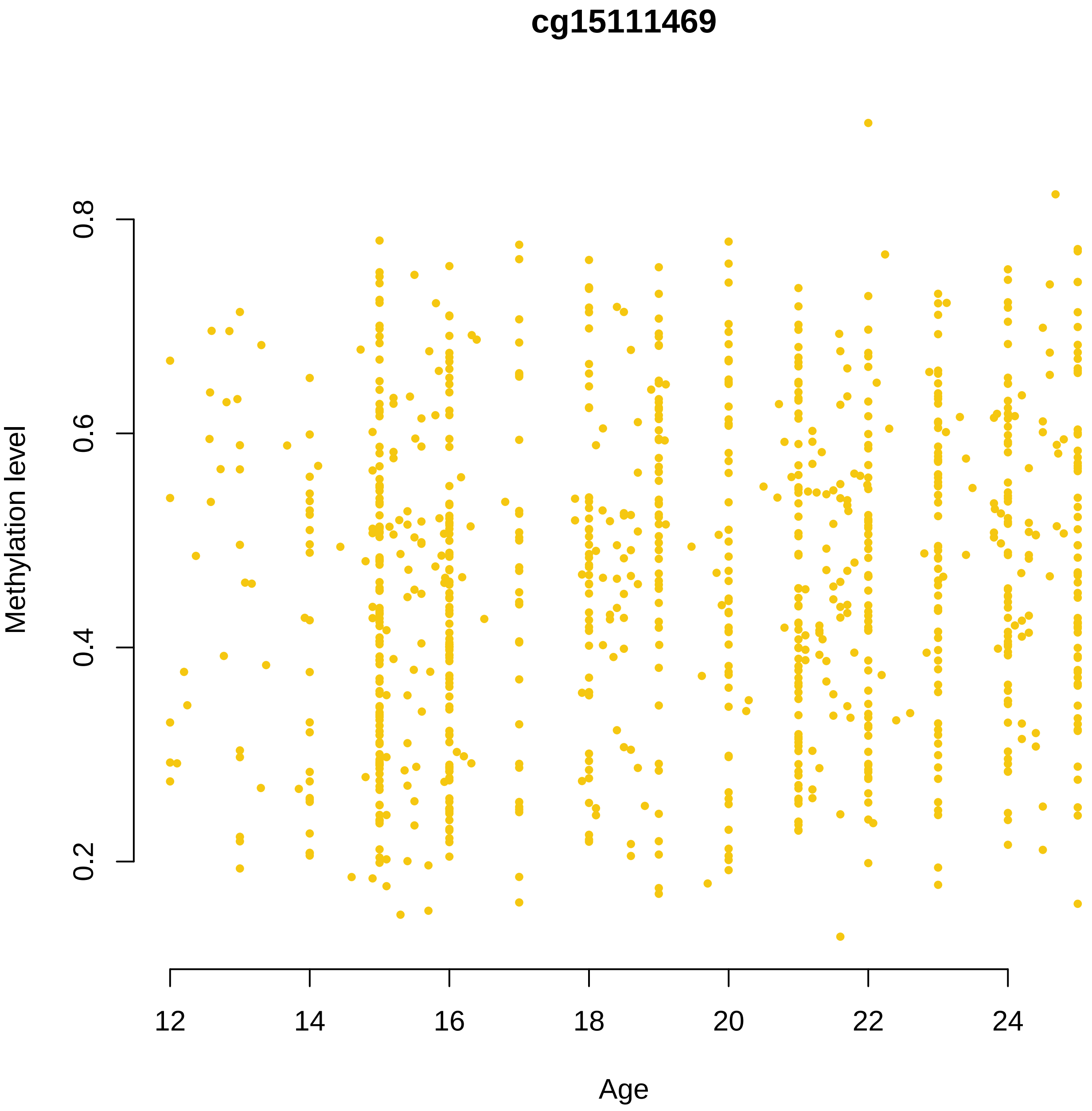
cg15111469
Methylation level
0.2
0.4
0.6
0.8
Age
12
14
16
18
20
22
24

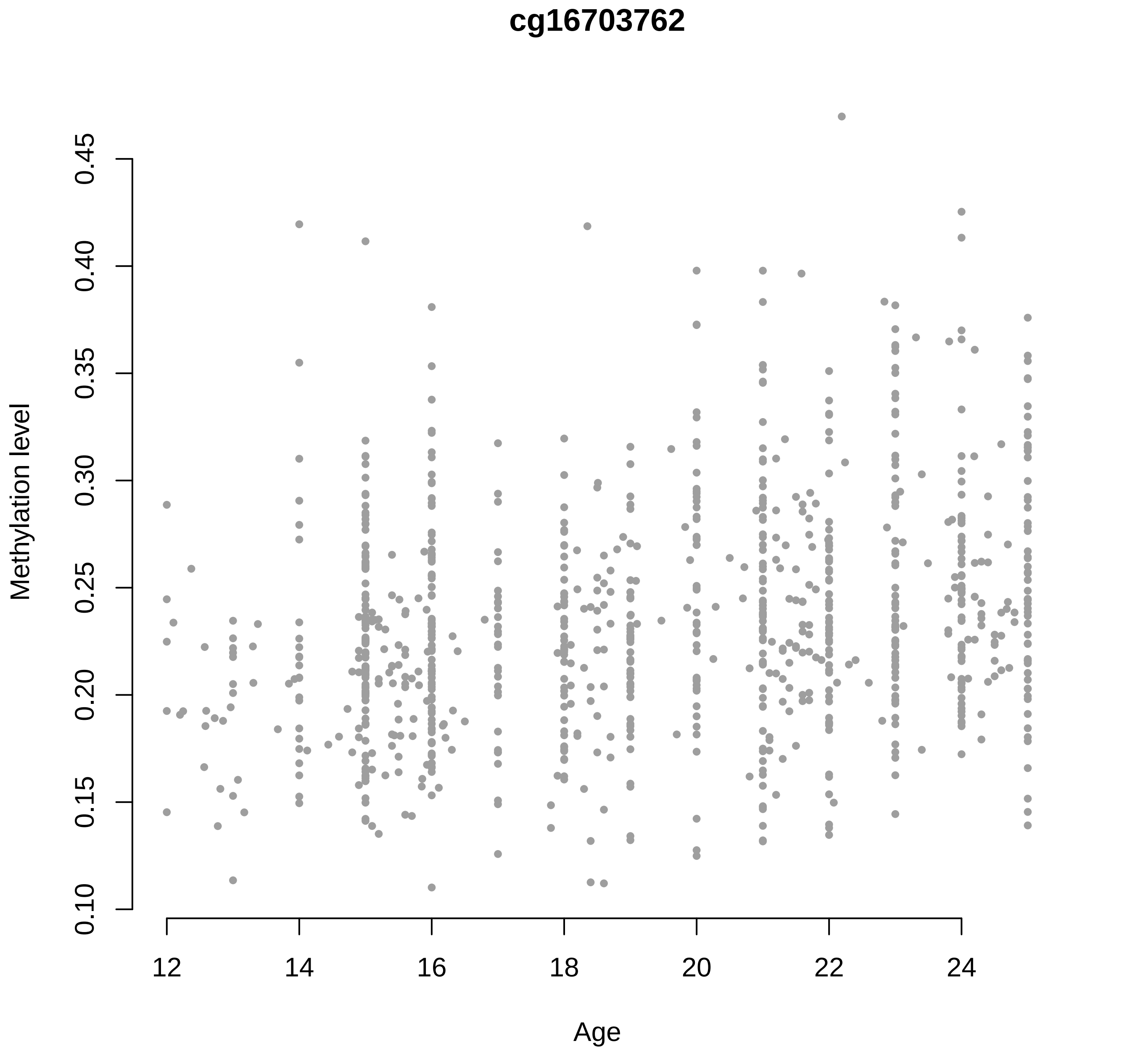

cg16703762
Methylation level
0.10
0.15
0.20
0.25
0.30
0.35
0.40
0.45
Age
12
14
16
18
20
22
24

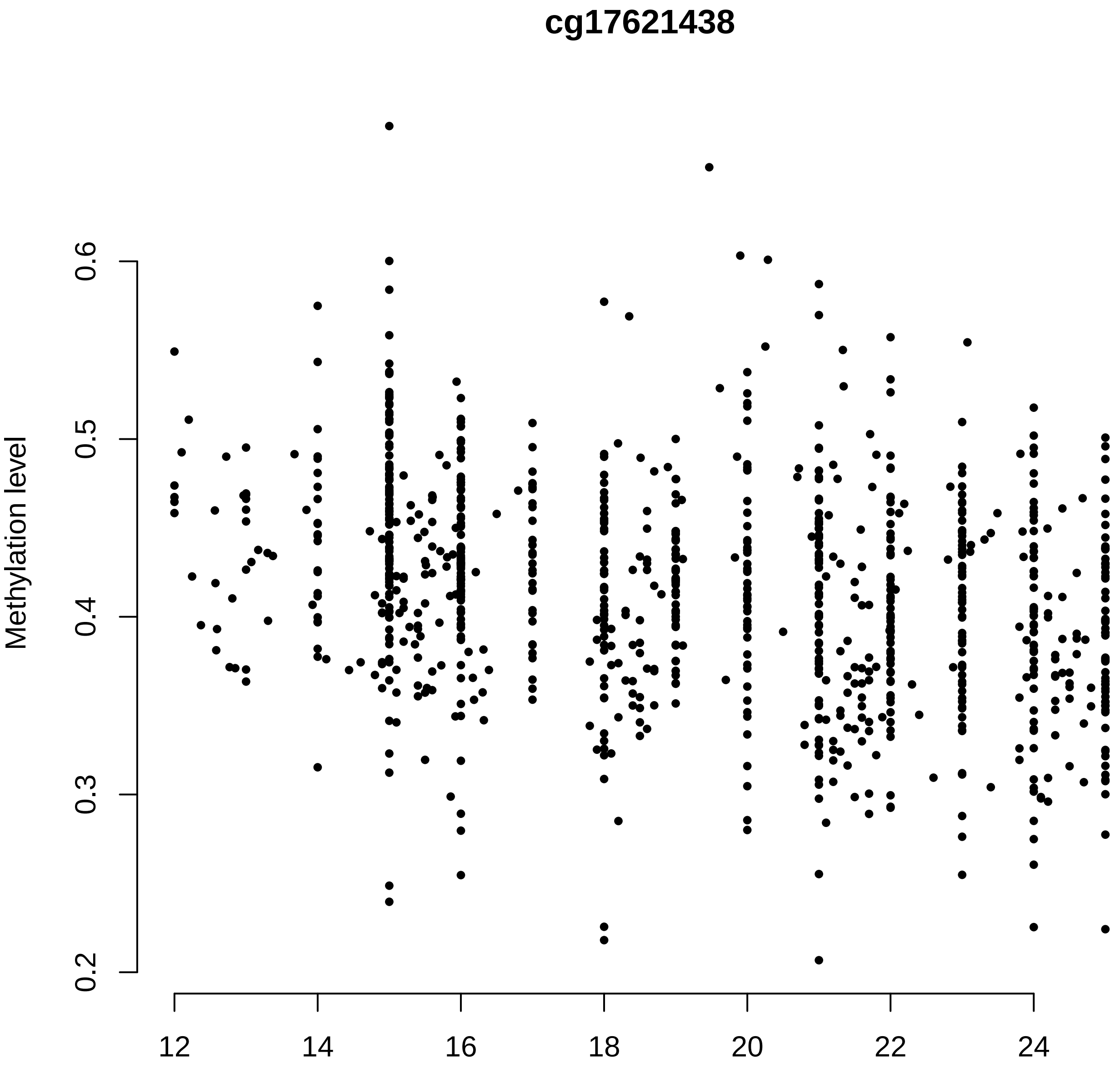

cg17621438
Methylation level
0.2
0.3
0.4
0.5
0.6
Age
12
14
16
18
20
22
24

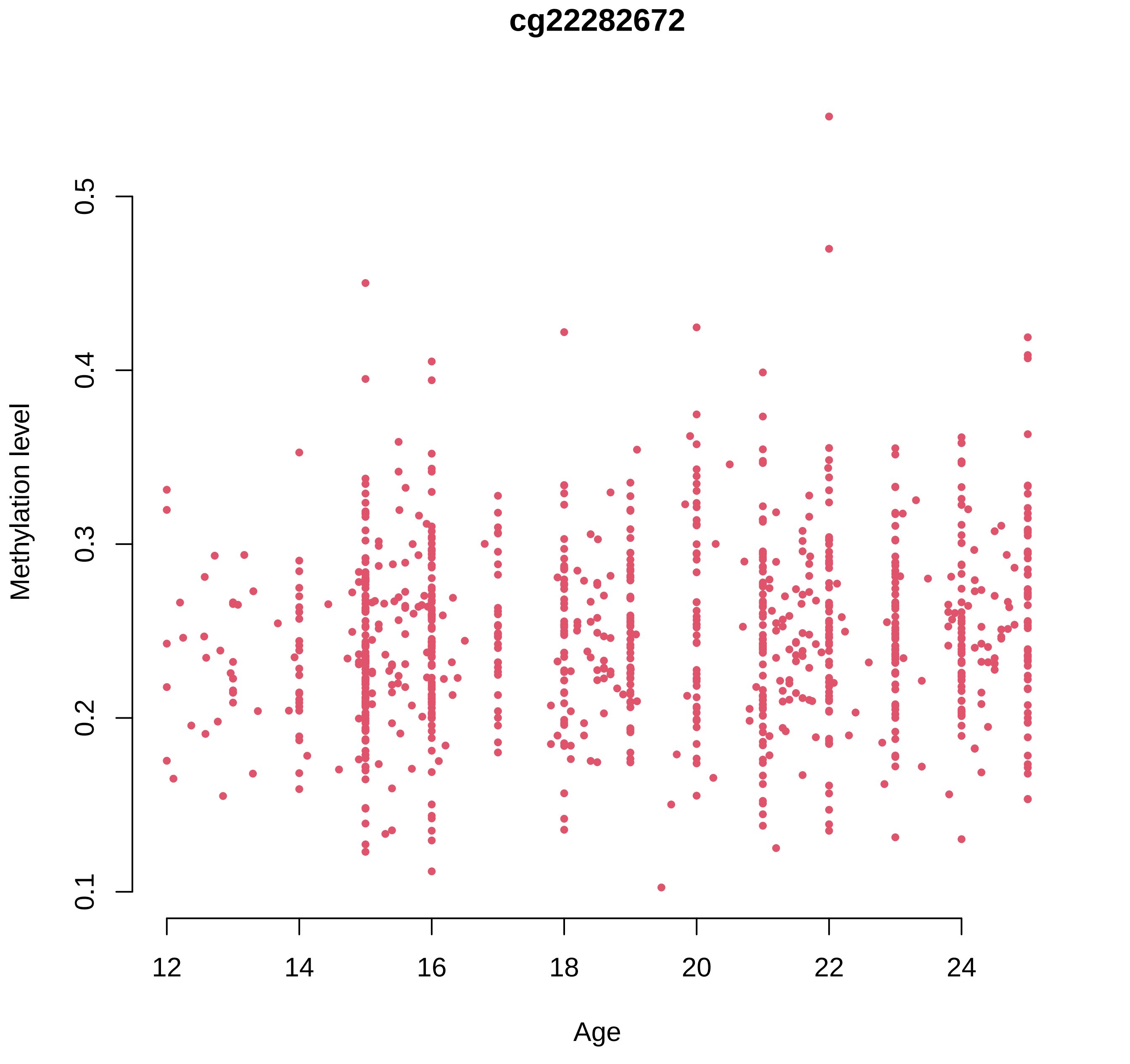
cg22282672
Methylation level
0.1
0.2
0.3
0.4
0.5
Age
12
14
16
18
20
22
24

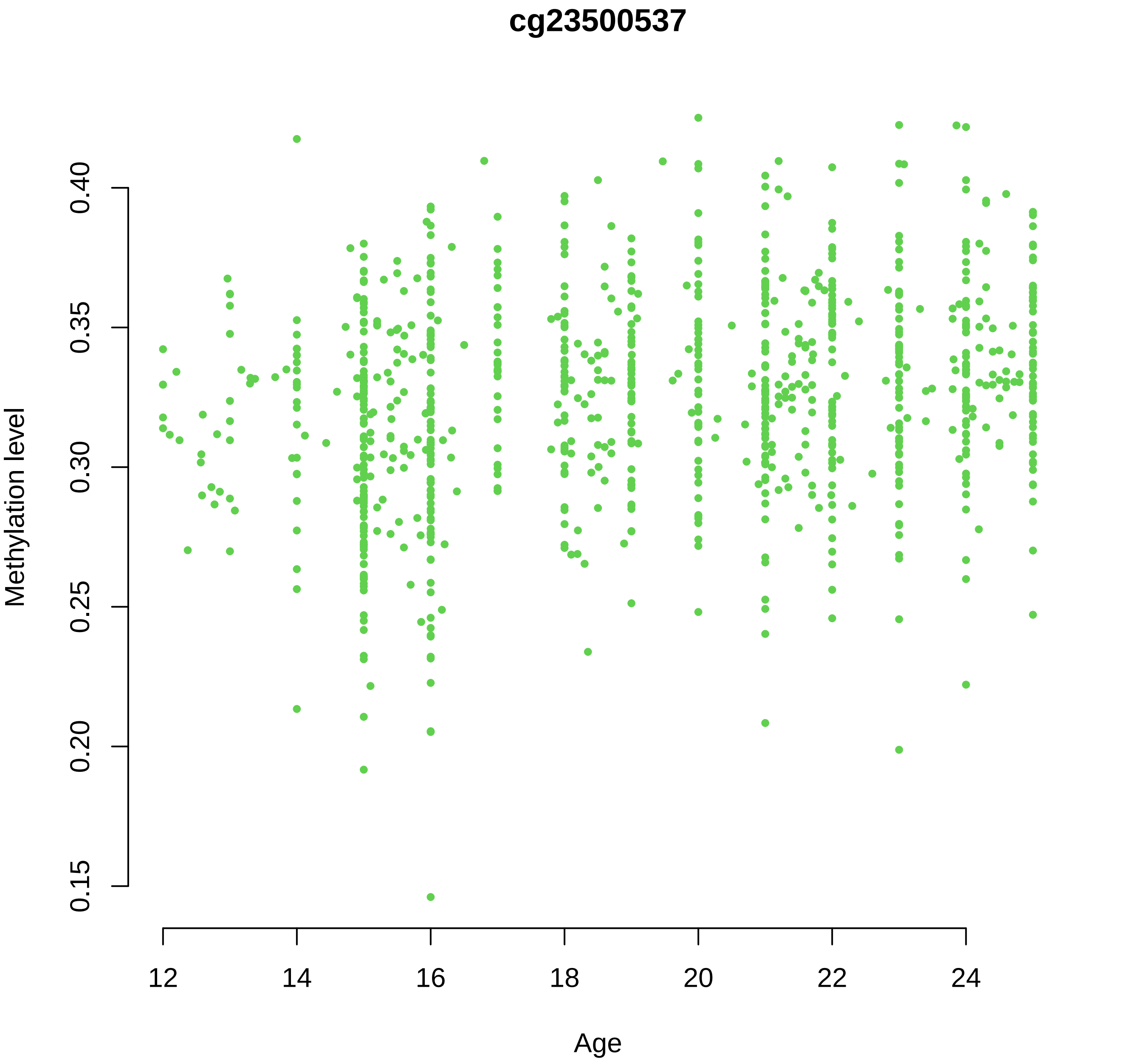
cg23500537
Methylation level
0.15
0.20
0.25
0.30
0.35
0.40
12
14
16
18
20
22
24
Age

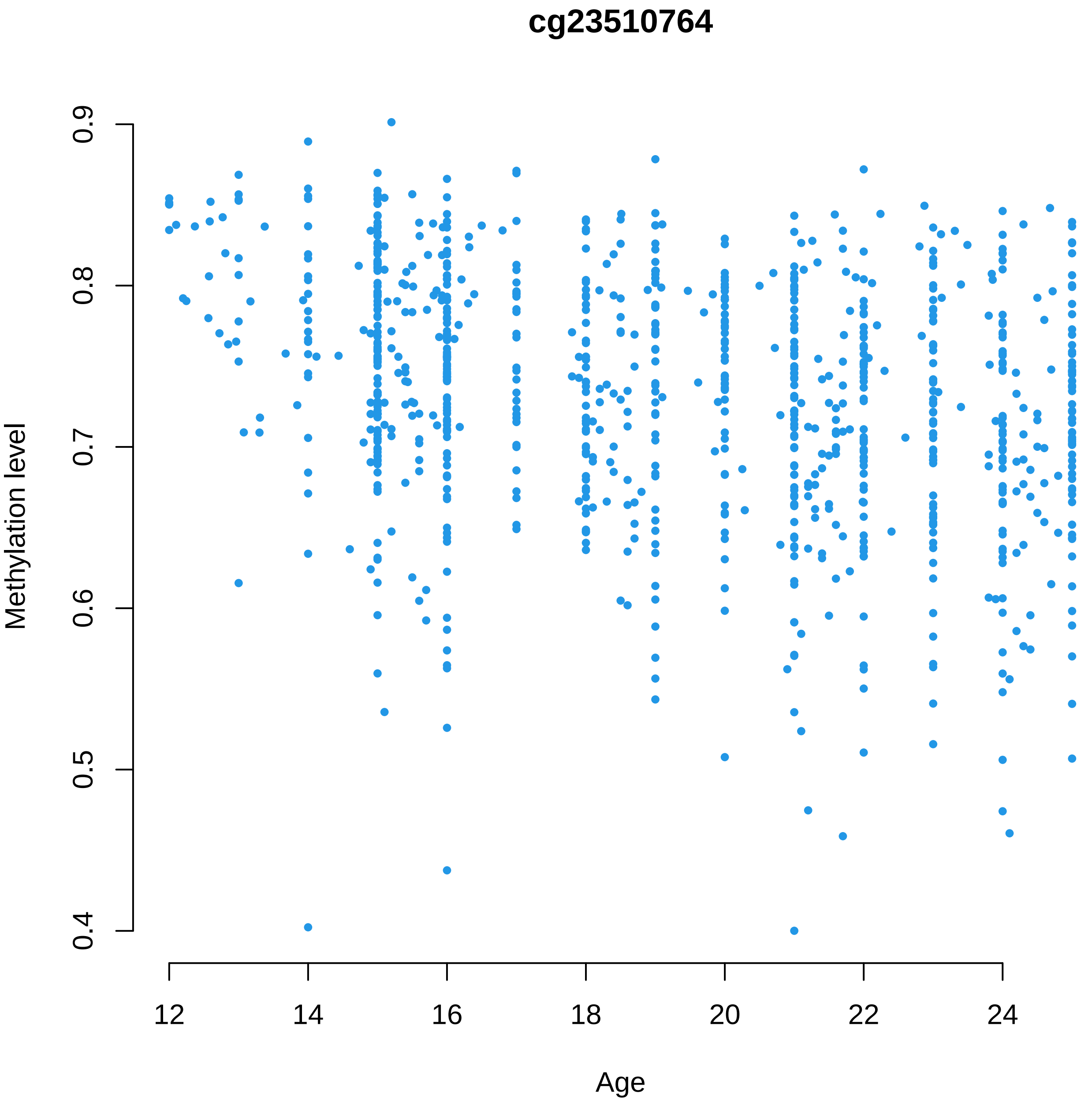

cg23510764
Methylation level
0.4
0.5
0.6
0.7
0.8
0.9
Age
12
14
16
18
20
22
24

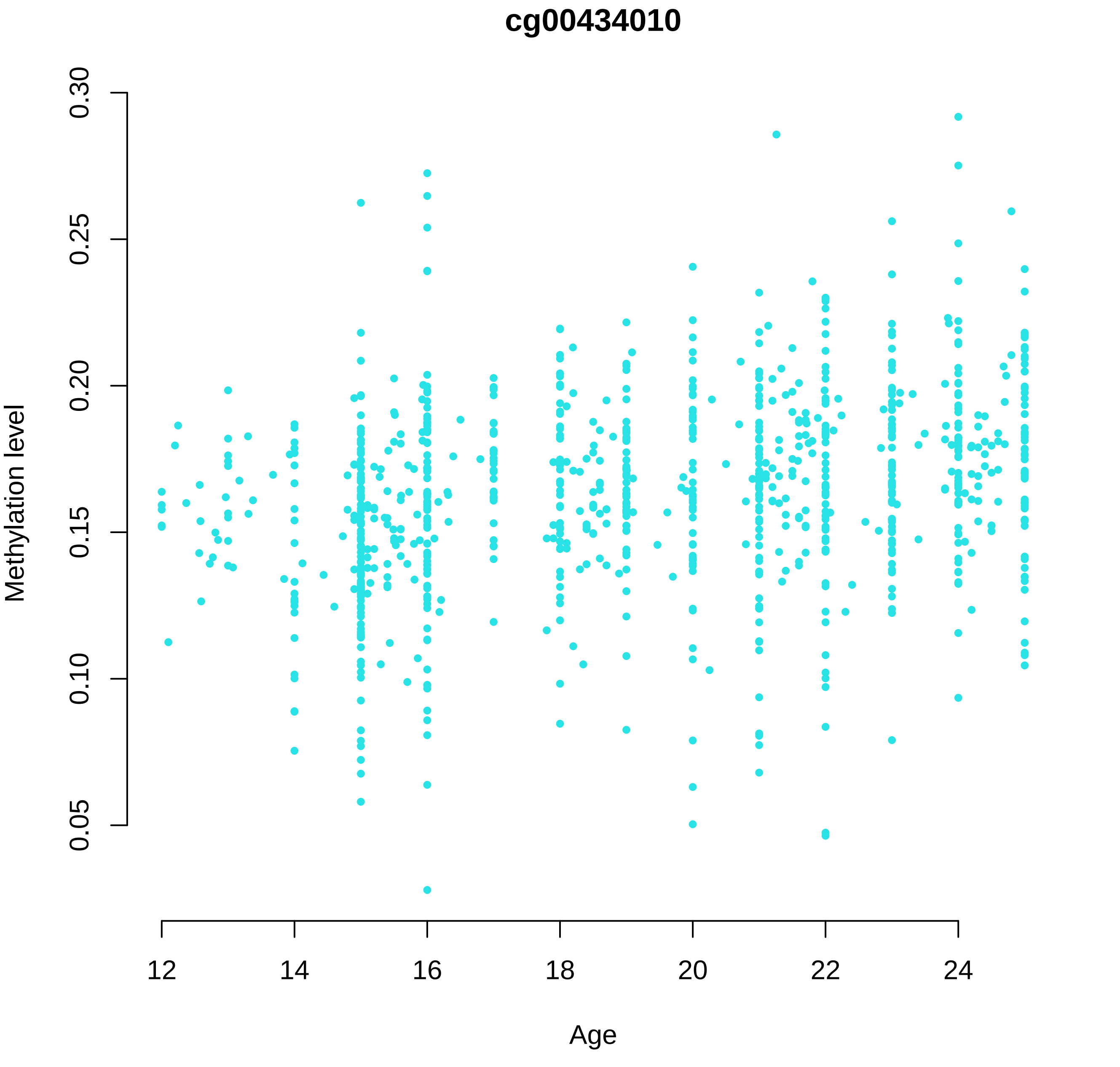
cg00434010
Methylation level
0.05
0.10
0.15
0.20
0.25
0.30
Age
12
14
16
18
20
22
24

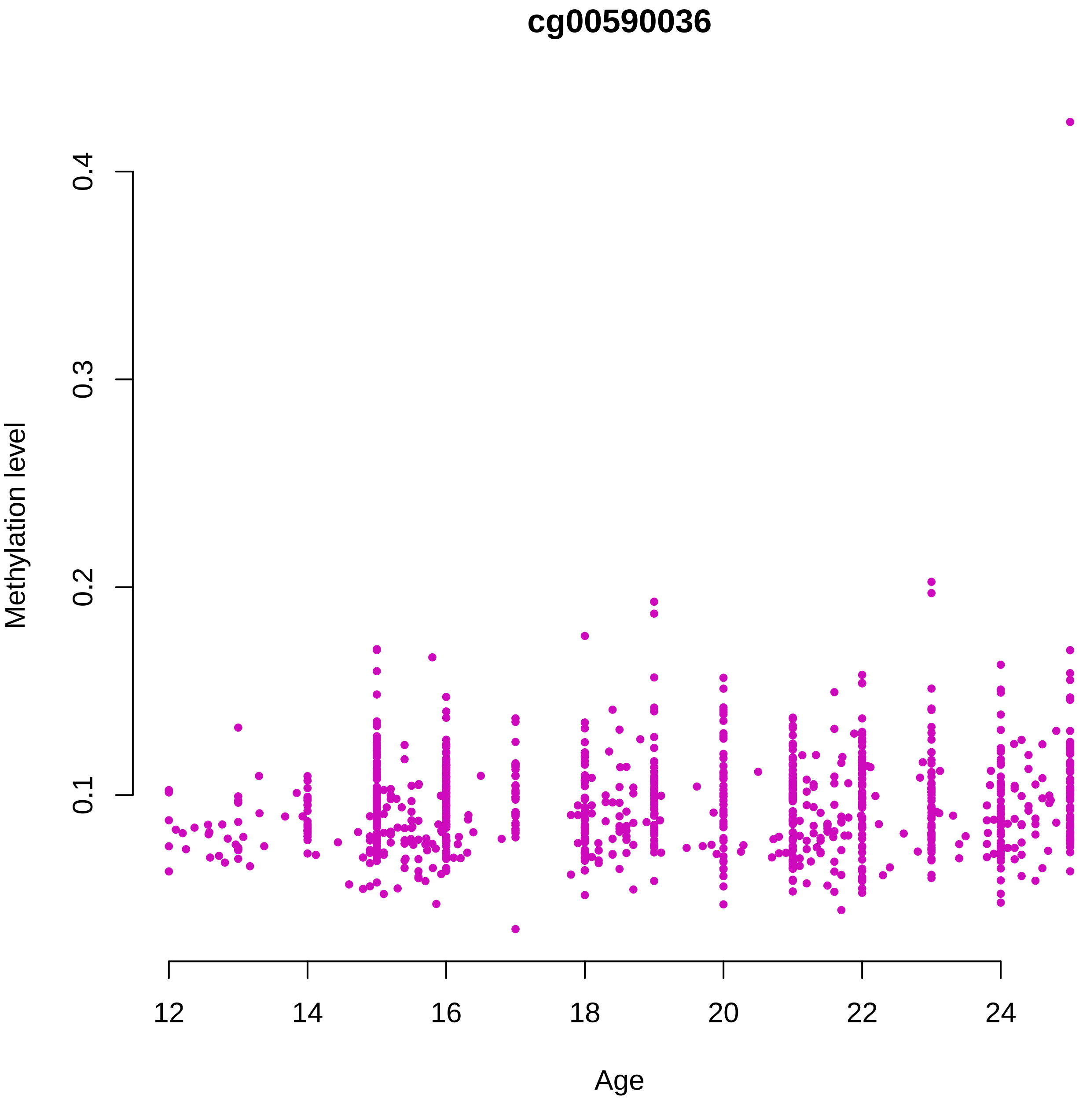
cg00590036
Methylation level
0.1
0.2
0.3
0.4
12
14
16
18
20
22
24
Age

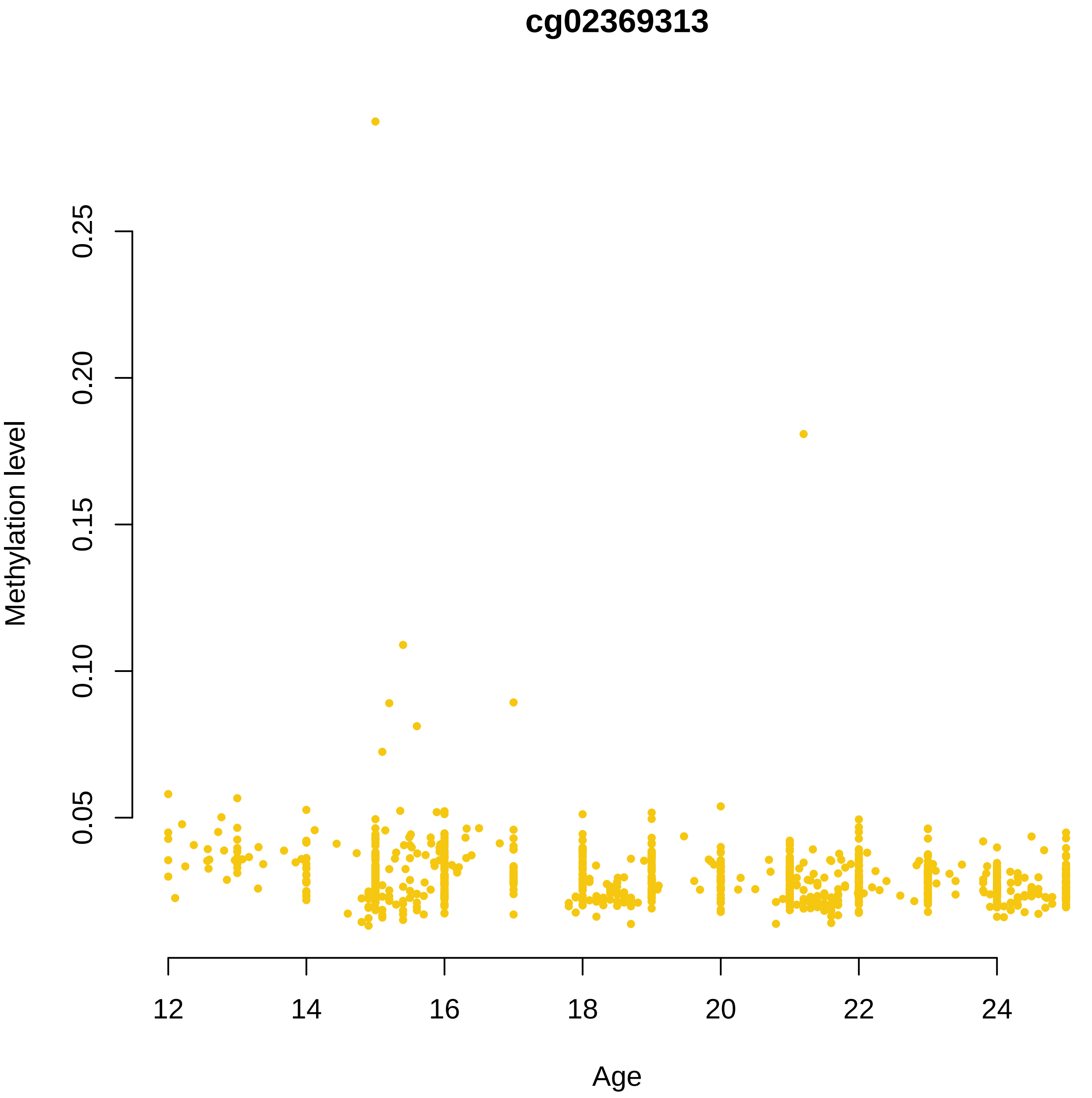
cg02369313
Methylation level
0.05
0.10
0.15
0.20
0.25
12
14
16
18
20
22
24
Age

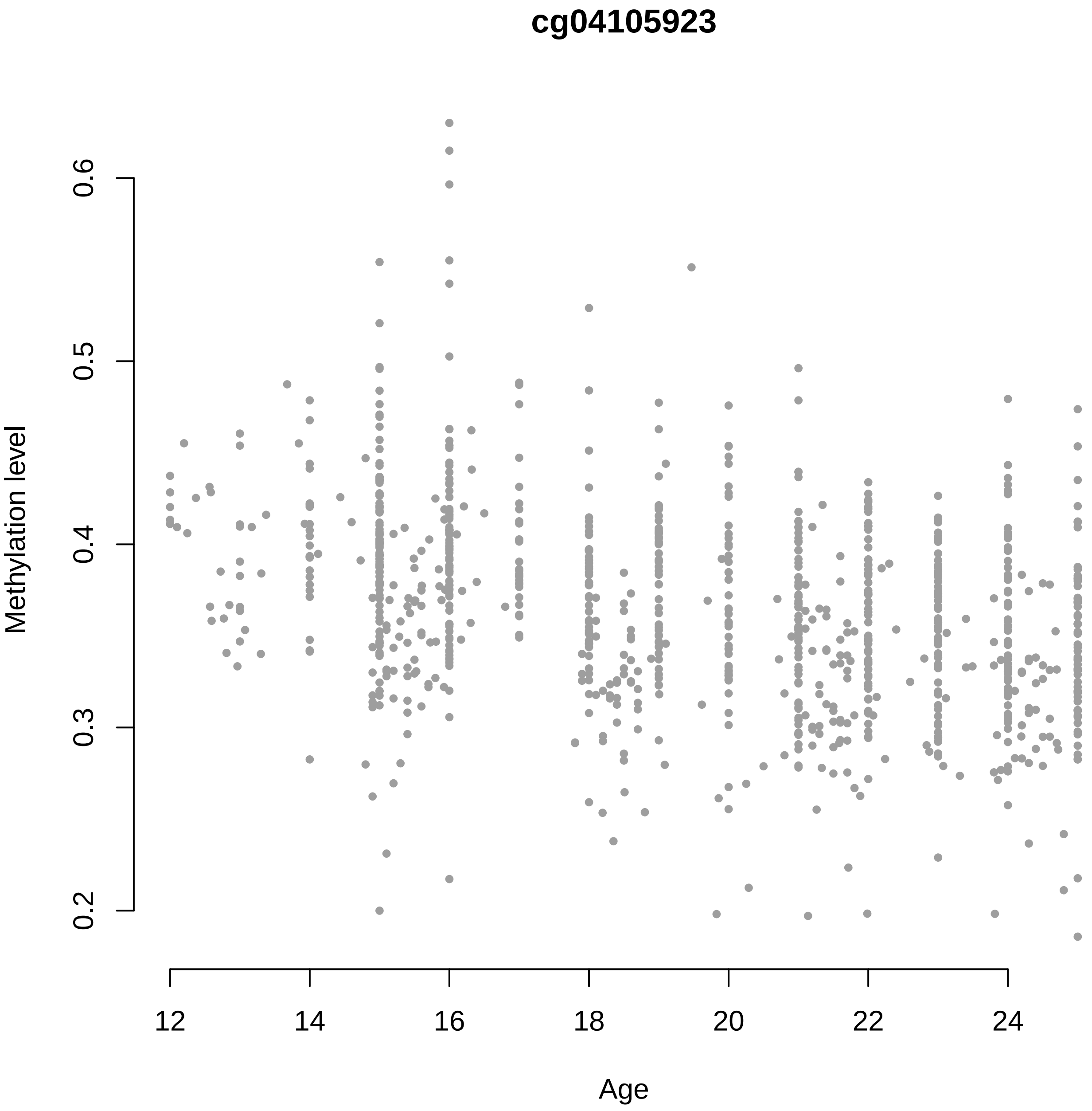
cg04105923
Methylation level
0.2
0.3
0.4
0.5
0.6
Age
12
14
16
18
20
22
24

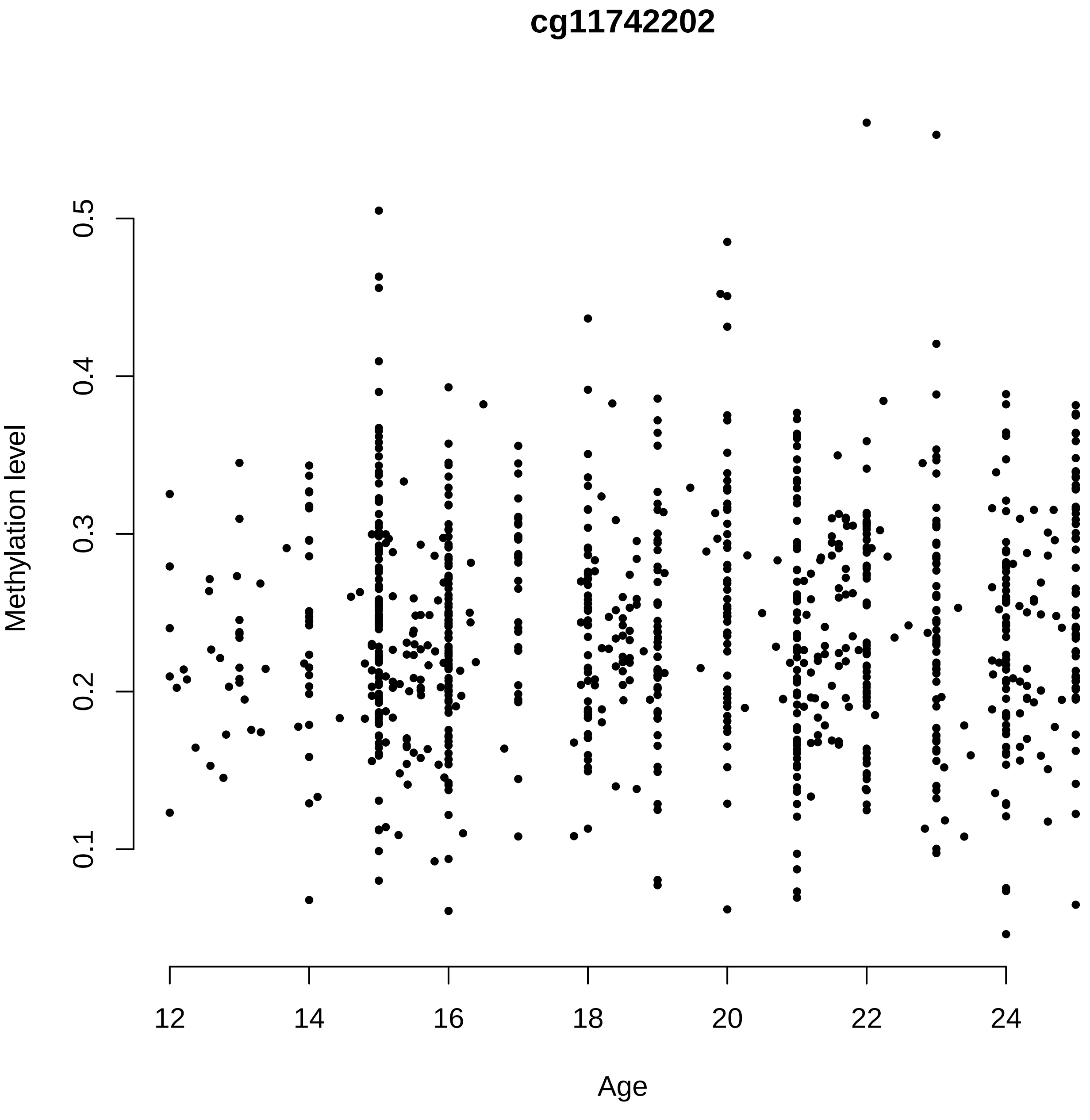

cg11742202
Methylation level
0.1
0.2
0.3
0.4
0.5
Age
12
14
16
18
20
22
24

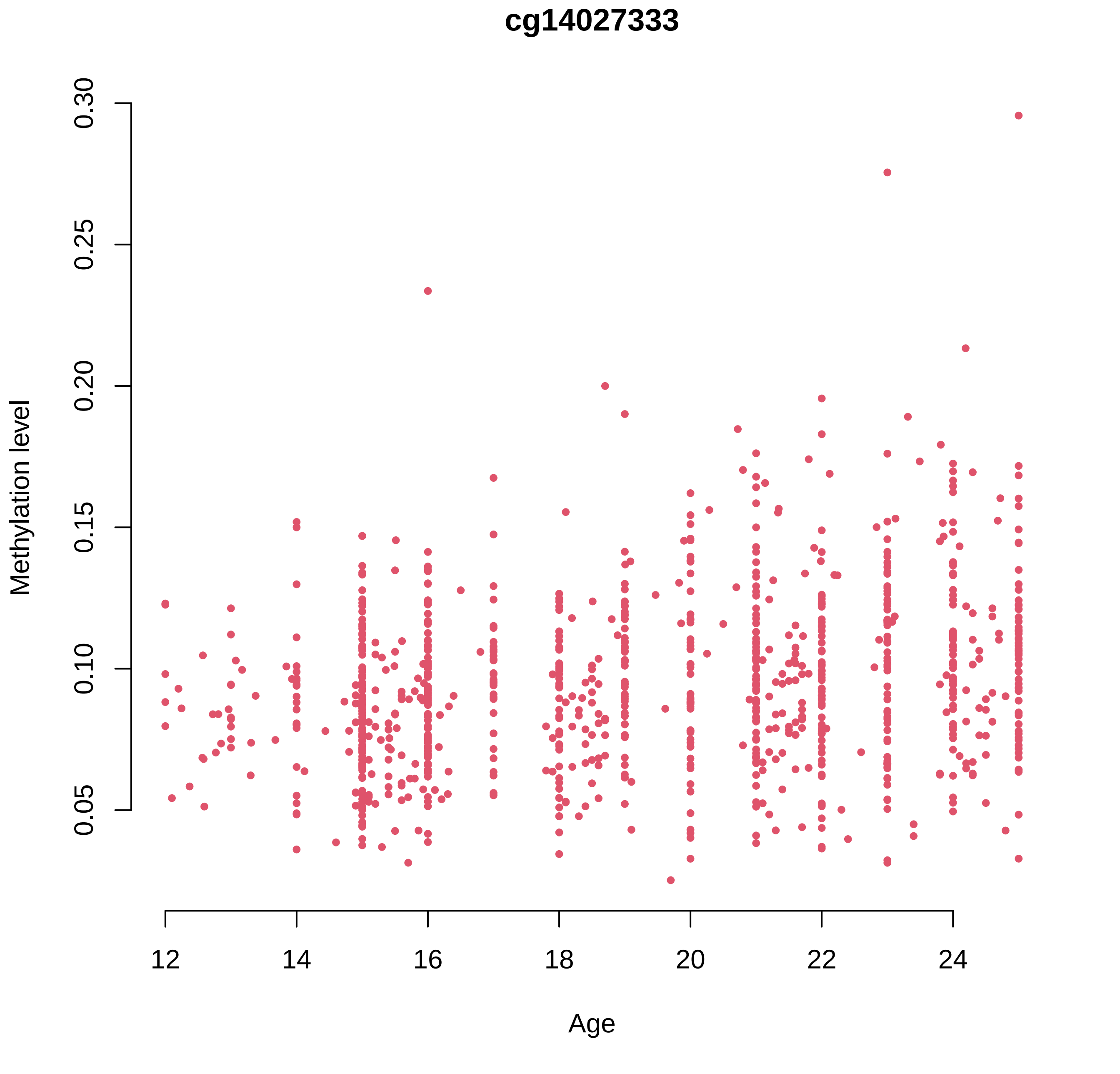
cg14027333
Methylation level
0.05
0.10
0.15
0.20
0.25
0.30
Age
12
14
16
18
20
22
24

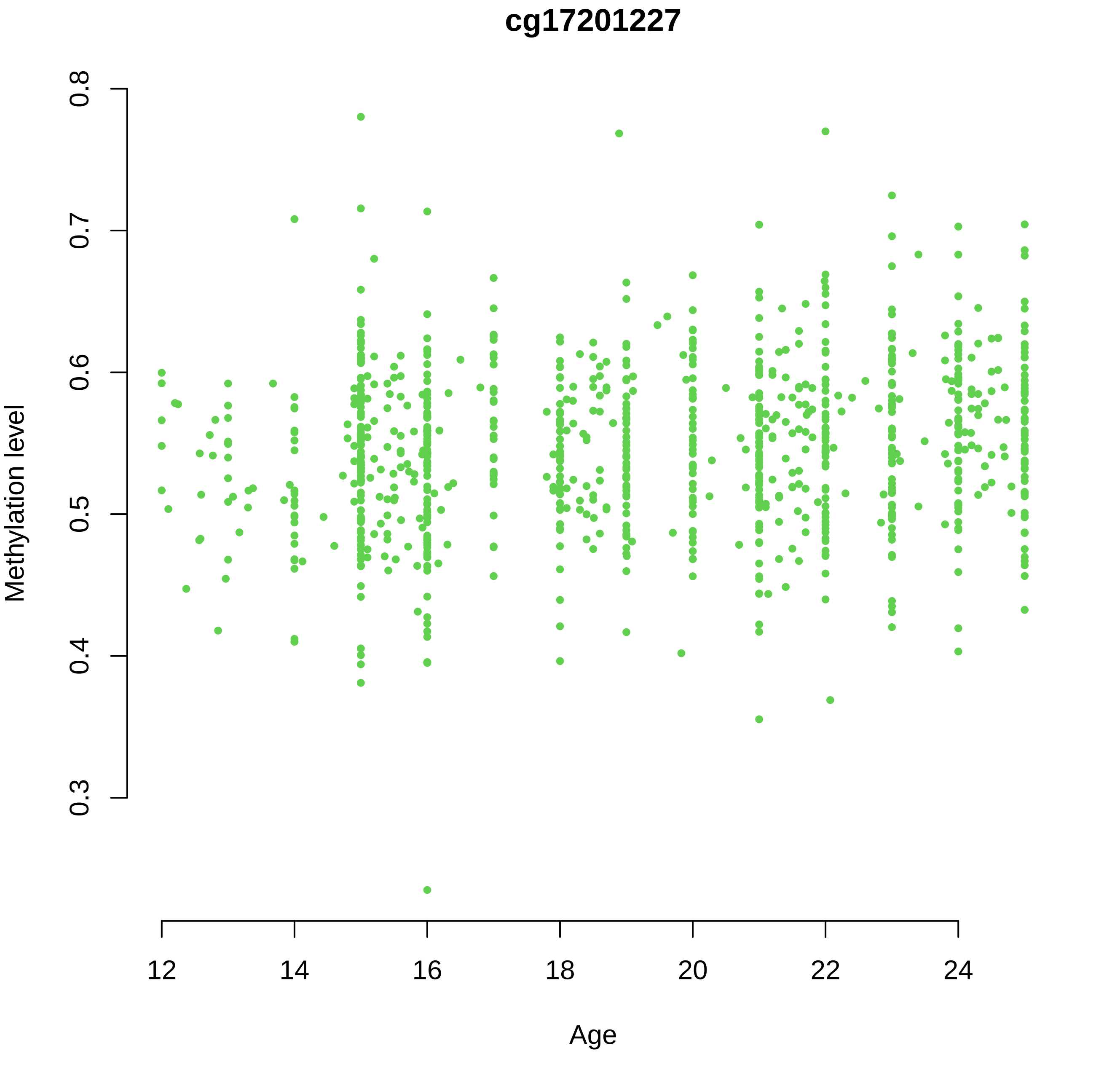
cg17201227
Methylation level
0.3
0.4
0.5
0.6
0.7
0.8
12
14
16
18
20
22
24
Age

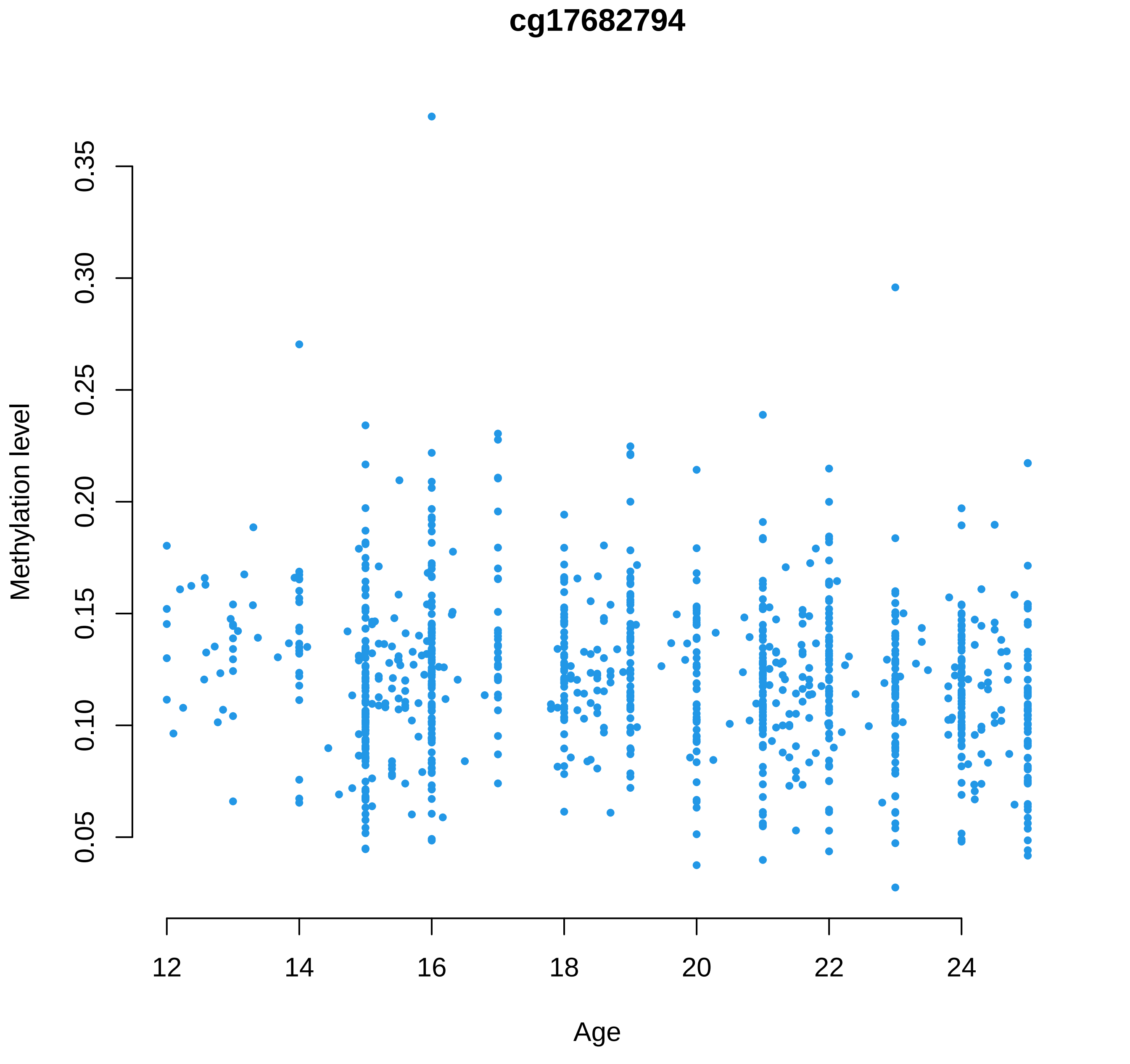

cg17682794
Methylation level
0.05
0.10
0.15
0.20
0.25
0.30
0.35
12
14
16
18
20
22
24
Age

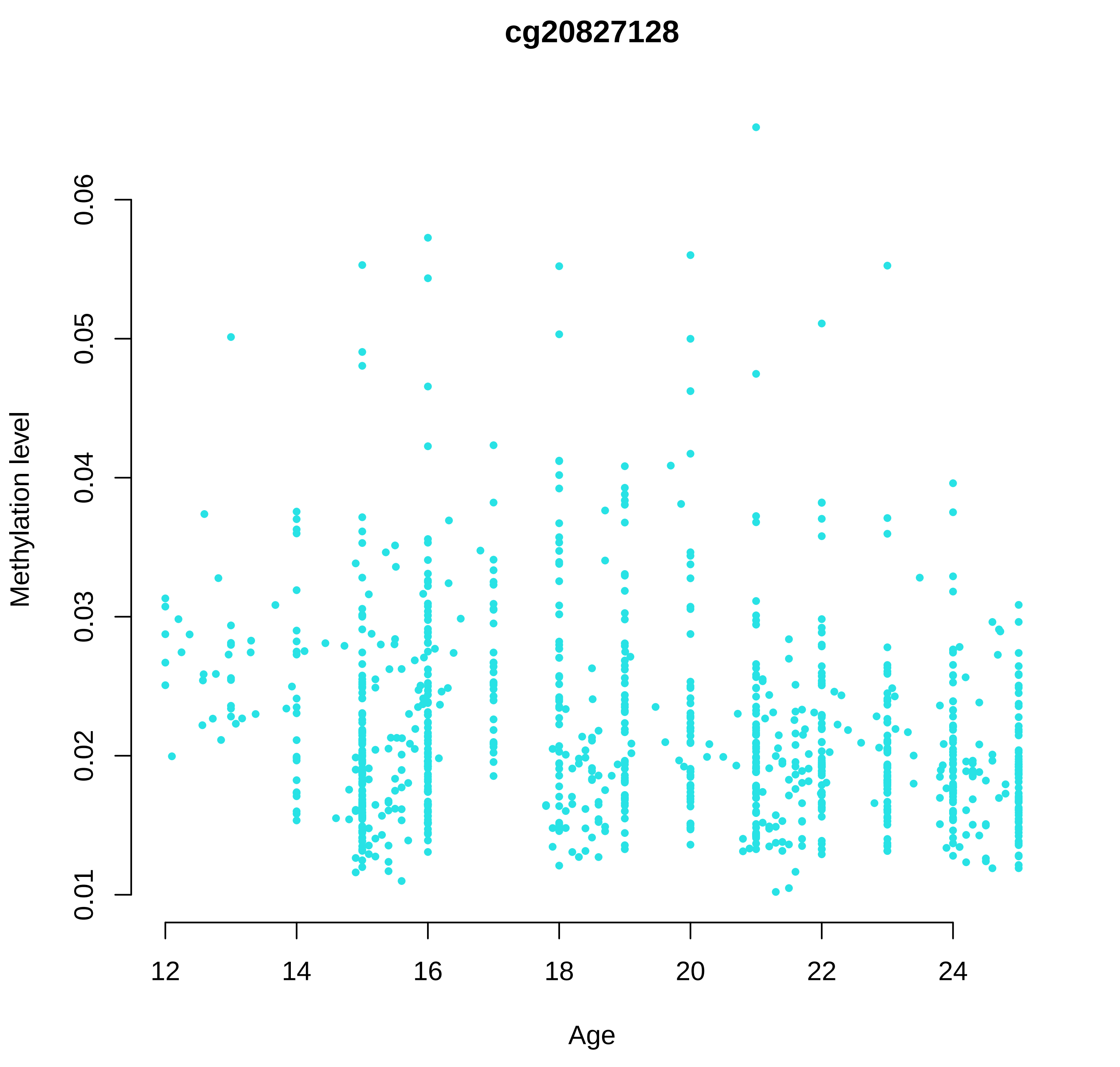
cg20827128
Methylation level
0.01
0.02
0.03
0.04
0.05
0.06
Age
12
14
16
18
20
22
24

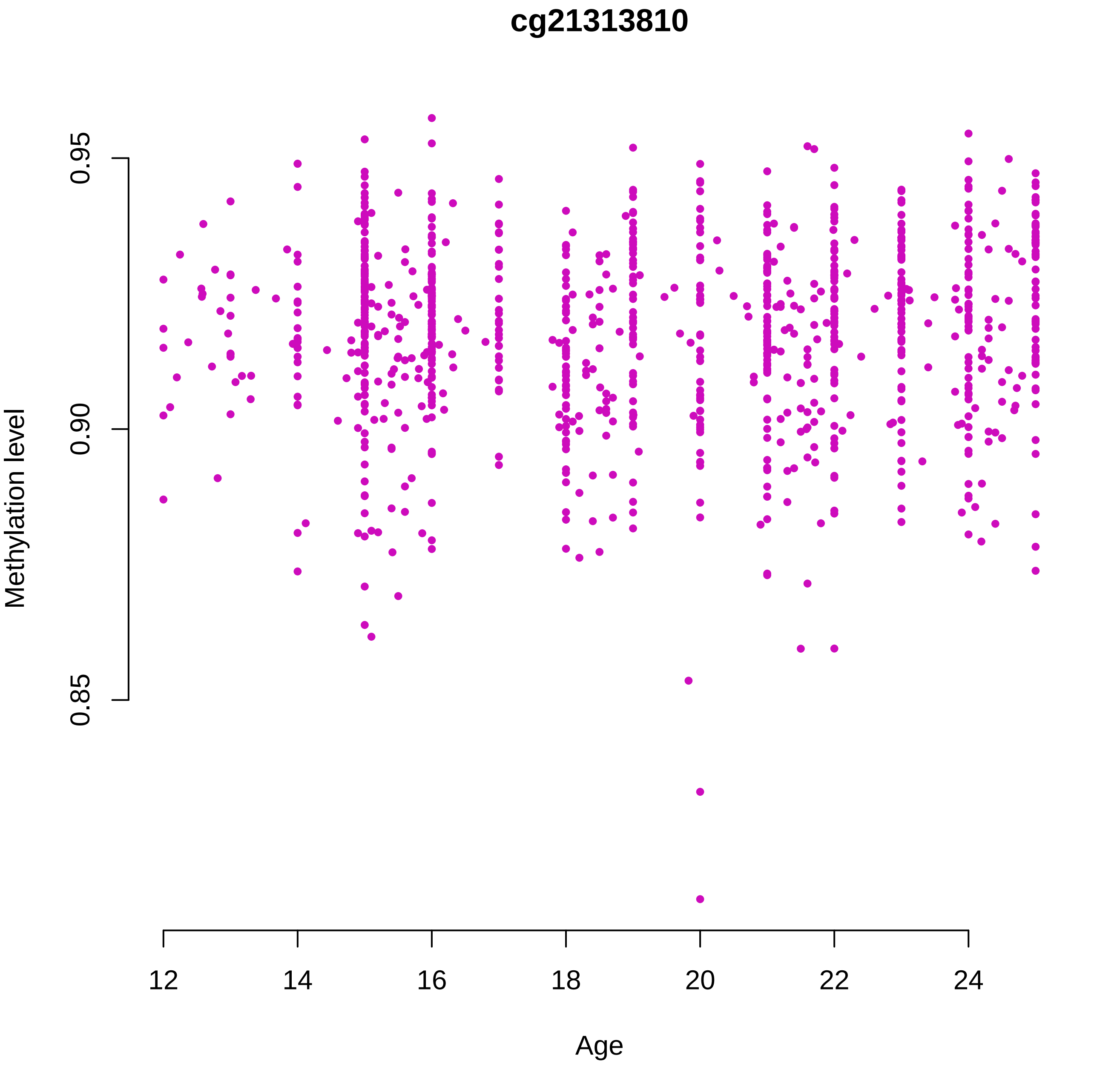
cg21313810
Methylation level
0.85
0.90
0.95
Age
12
14
16
18
20
22
24

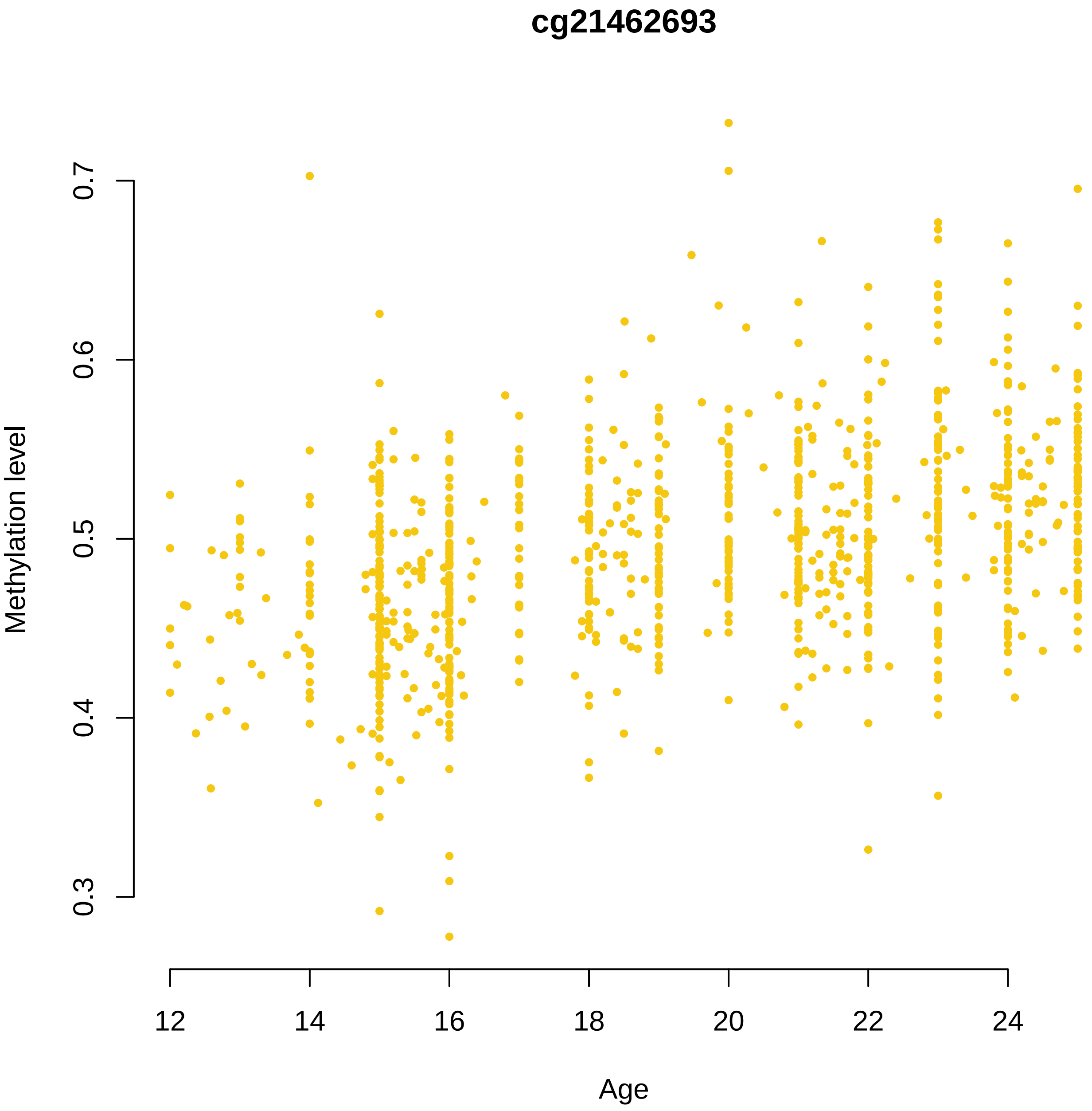
cg21462693
Methylation level
0.3
0.4
0.5
0.6
0.7
Age
12
14
16
18
20
22
24

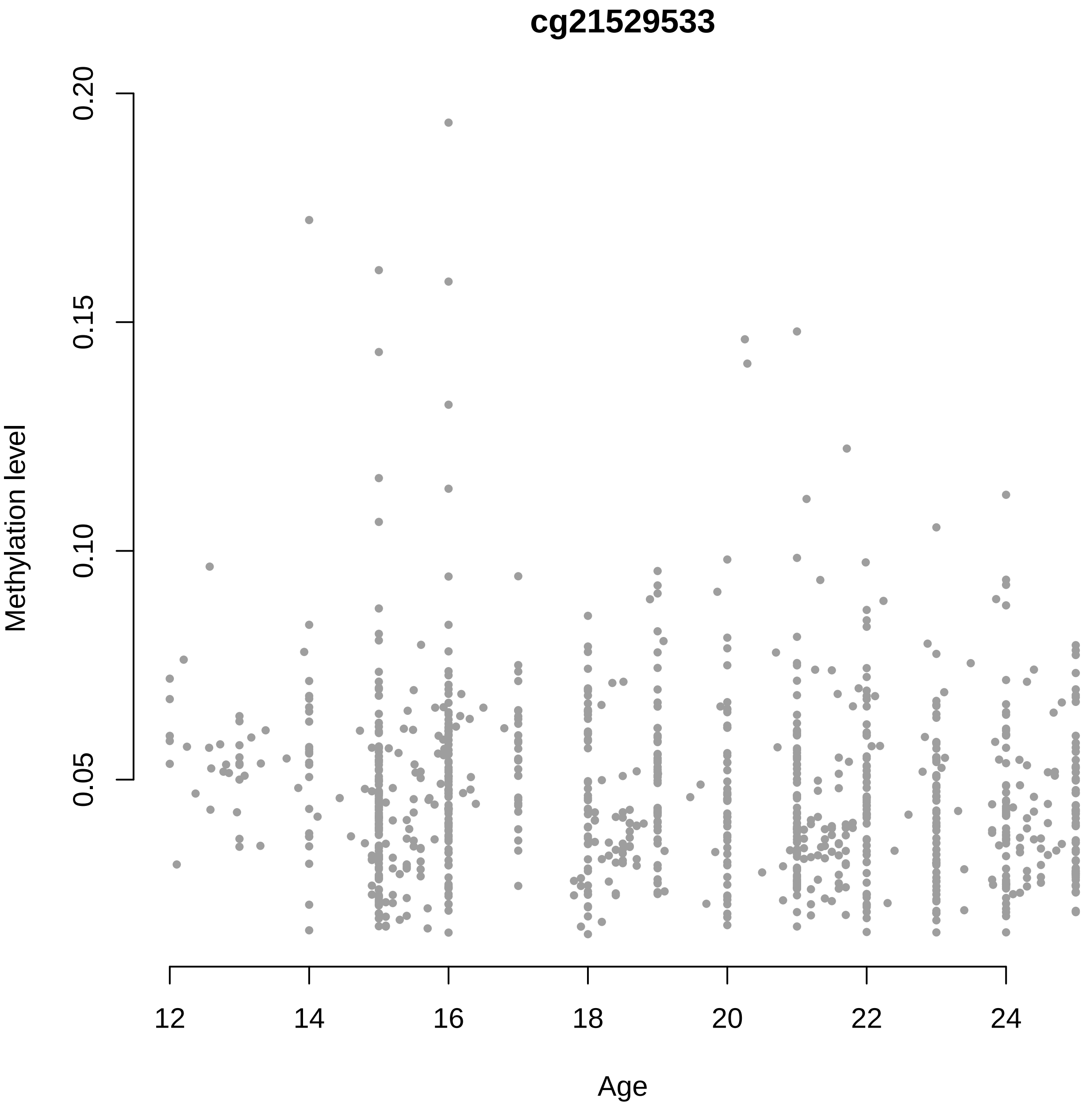
cg21529533
Methylation level
0.05
0.10
0.15
0.20
Age
12
14
16
18
20
22
24

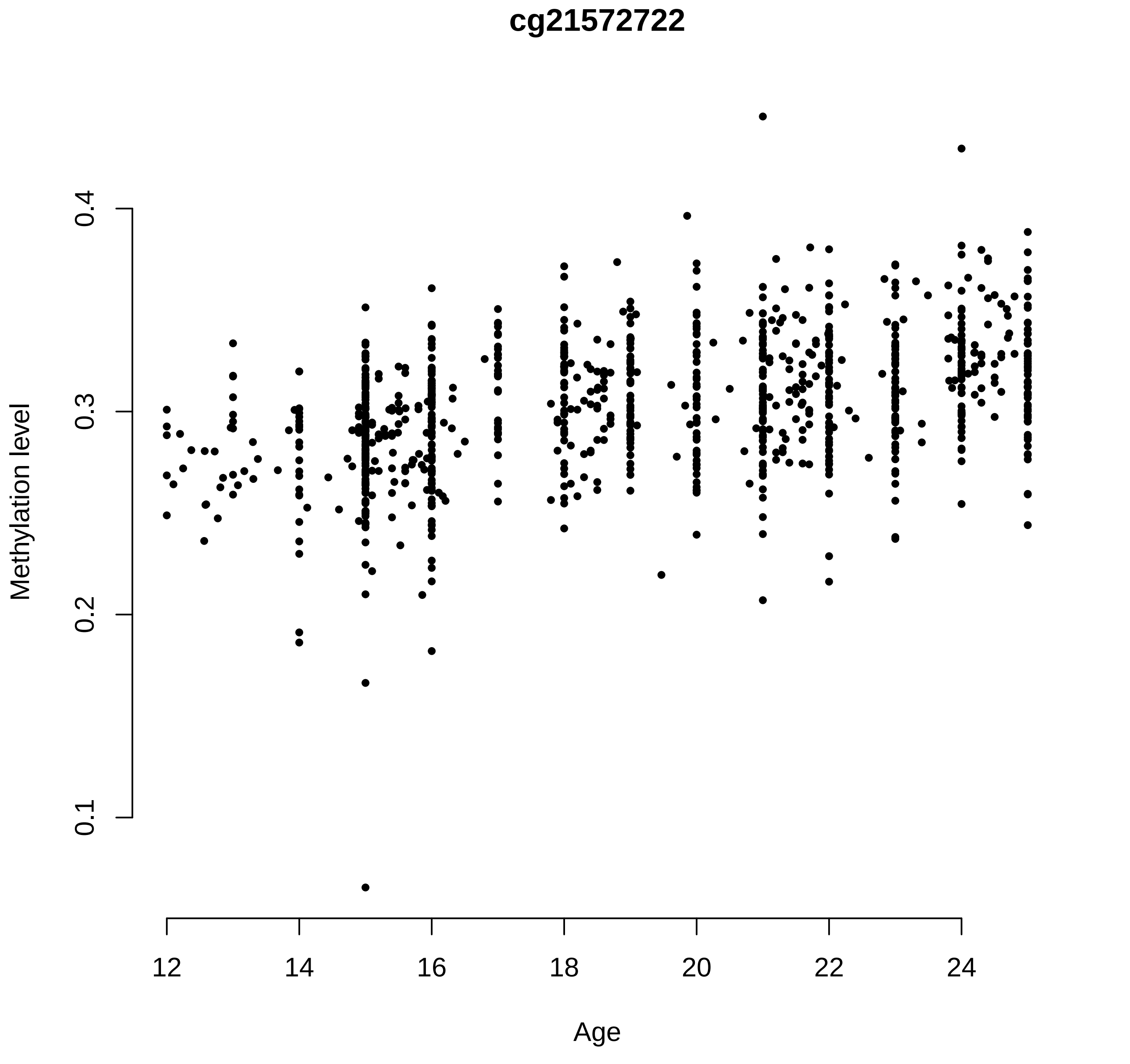

cg21572722
Methylation level
0.1
0.2
0.3
0.4
Age
12
14
16
18
20
22
24

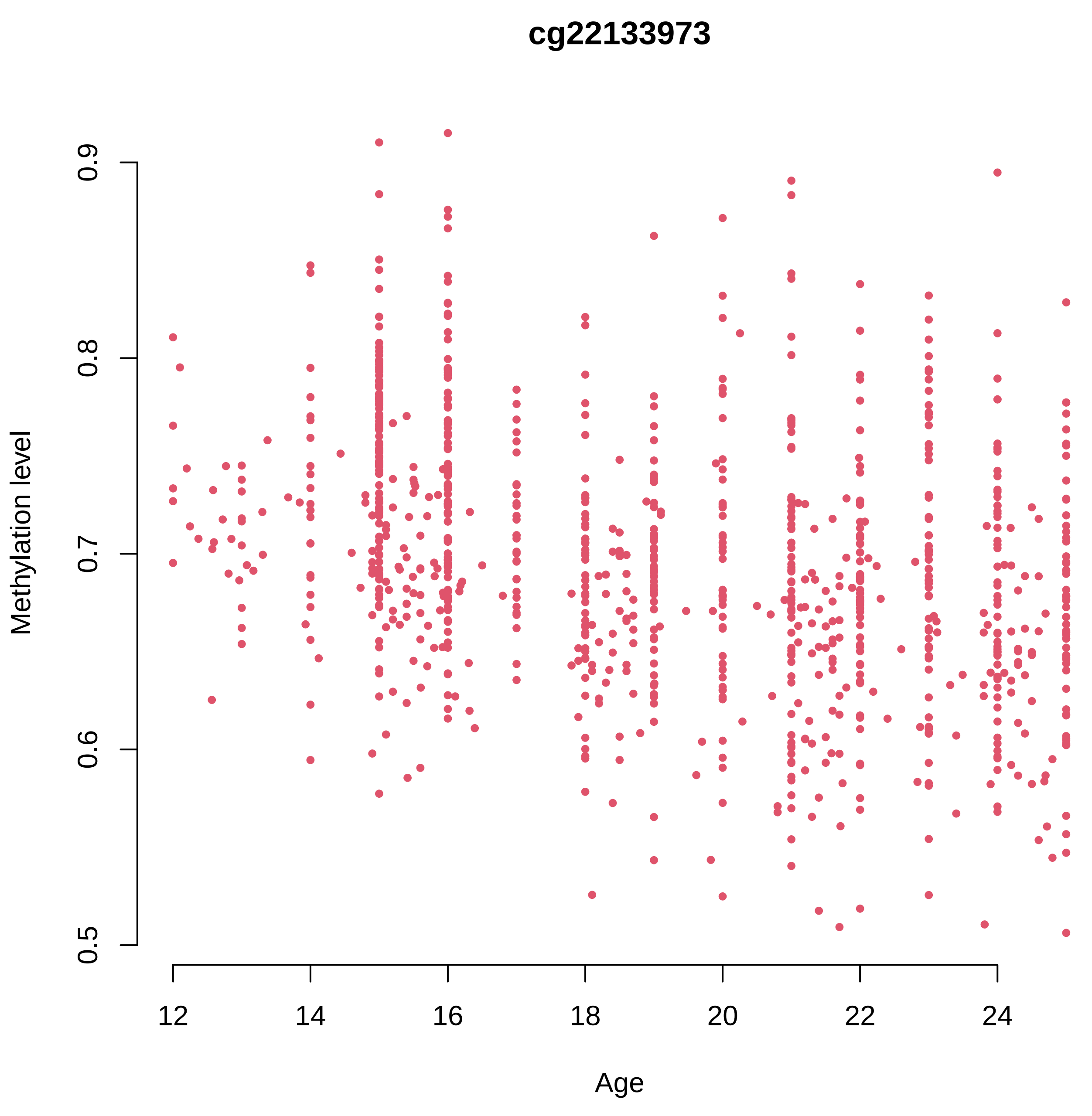
cg22133973
Methylation level
0.5
0.6
0.7
0.8
0.9
Age
12
14
16
18
20
22
24

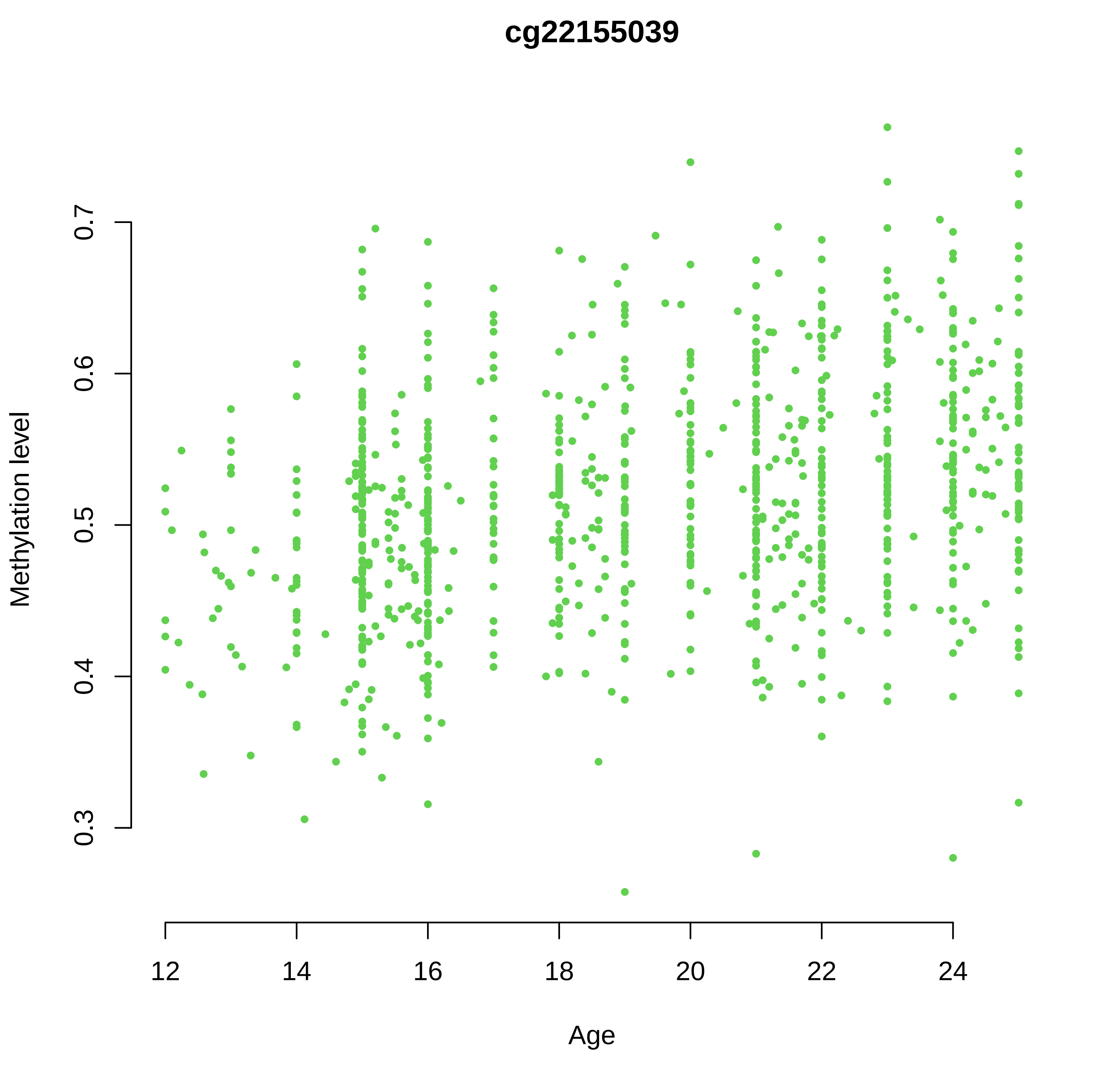
cg22155039
Methylation level
0.3
0.4
0.5
0.6
0.7
Age
12
14
16
18
20
22
24

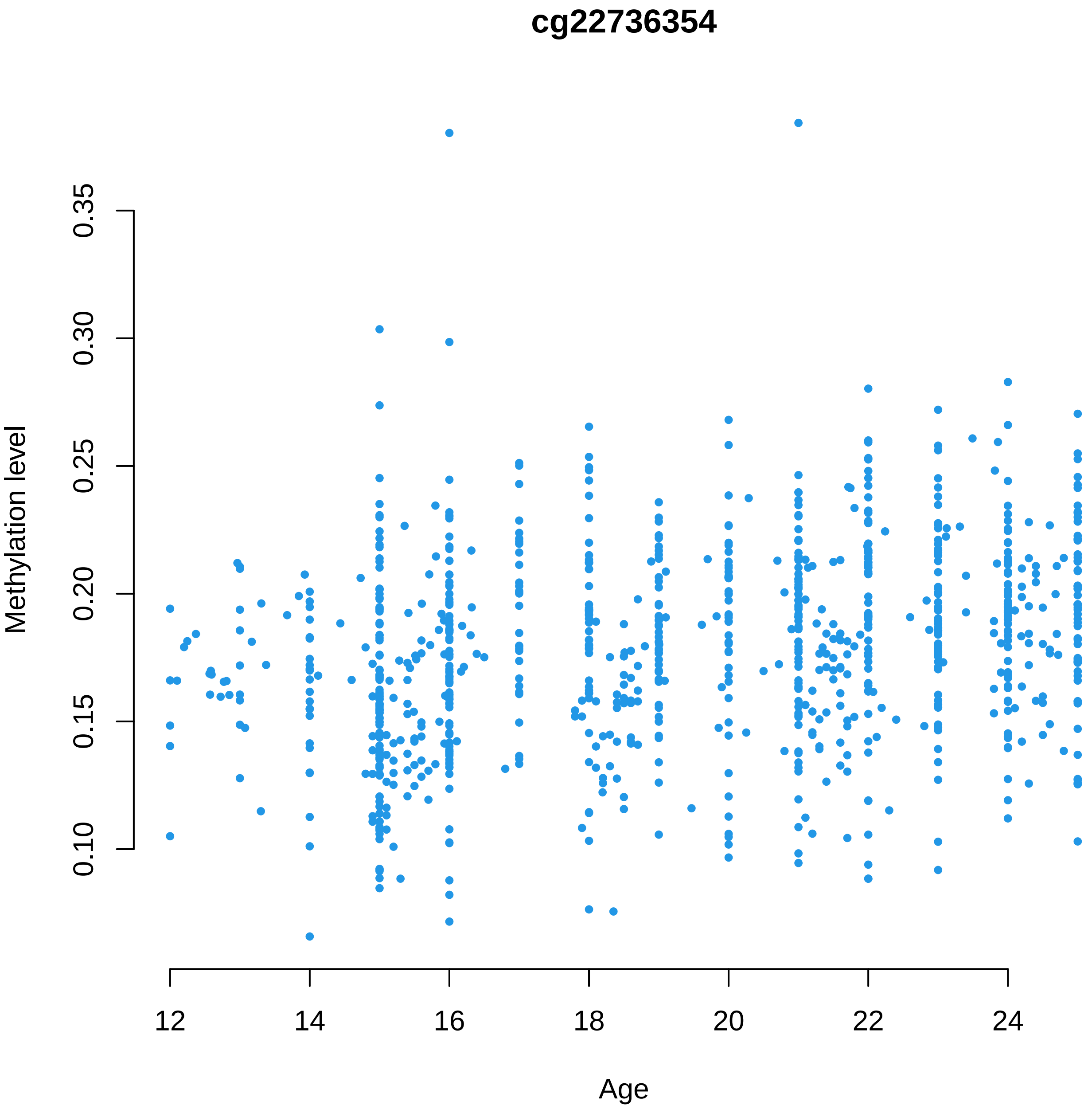

cg22736354
Methylation level
0.10
0.15
0.20
0.25
0.30
0.35
Age
12
14
16
18
20
22
24

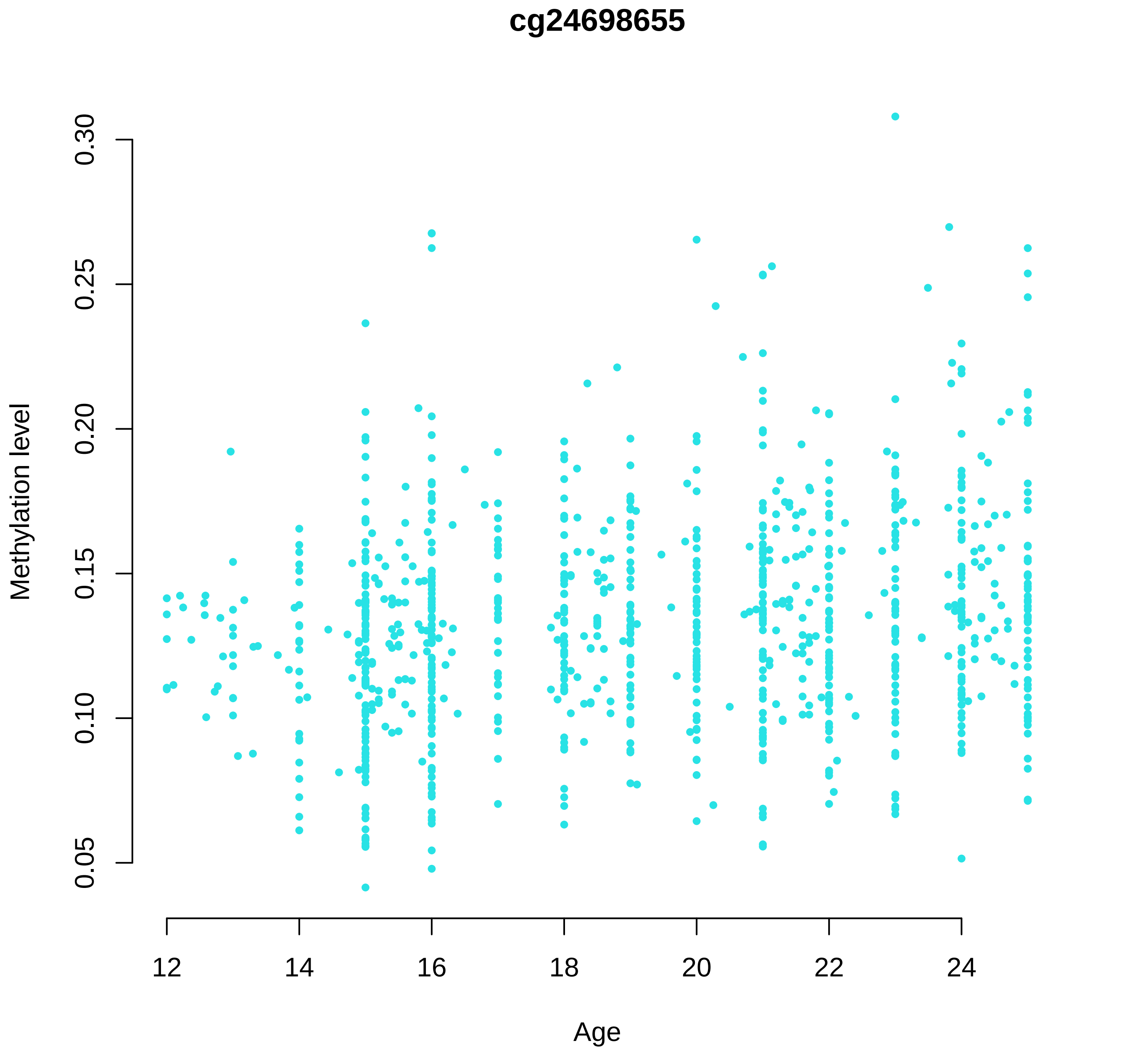
cg24698655
Methylation level
0.05
0.10
0.15
0.20
0.25
0.30
Age
12
14
16
18
20
22
24

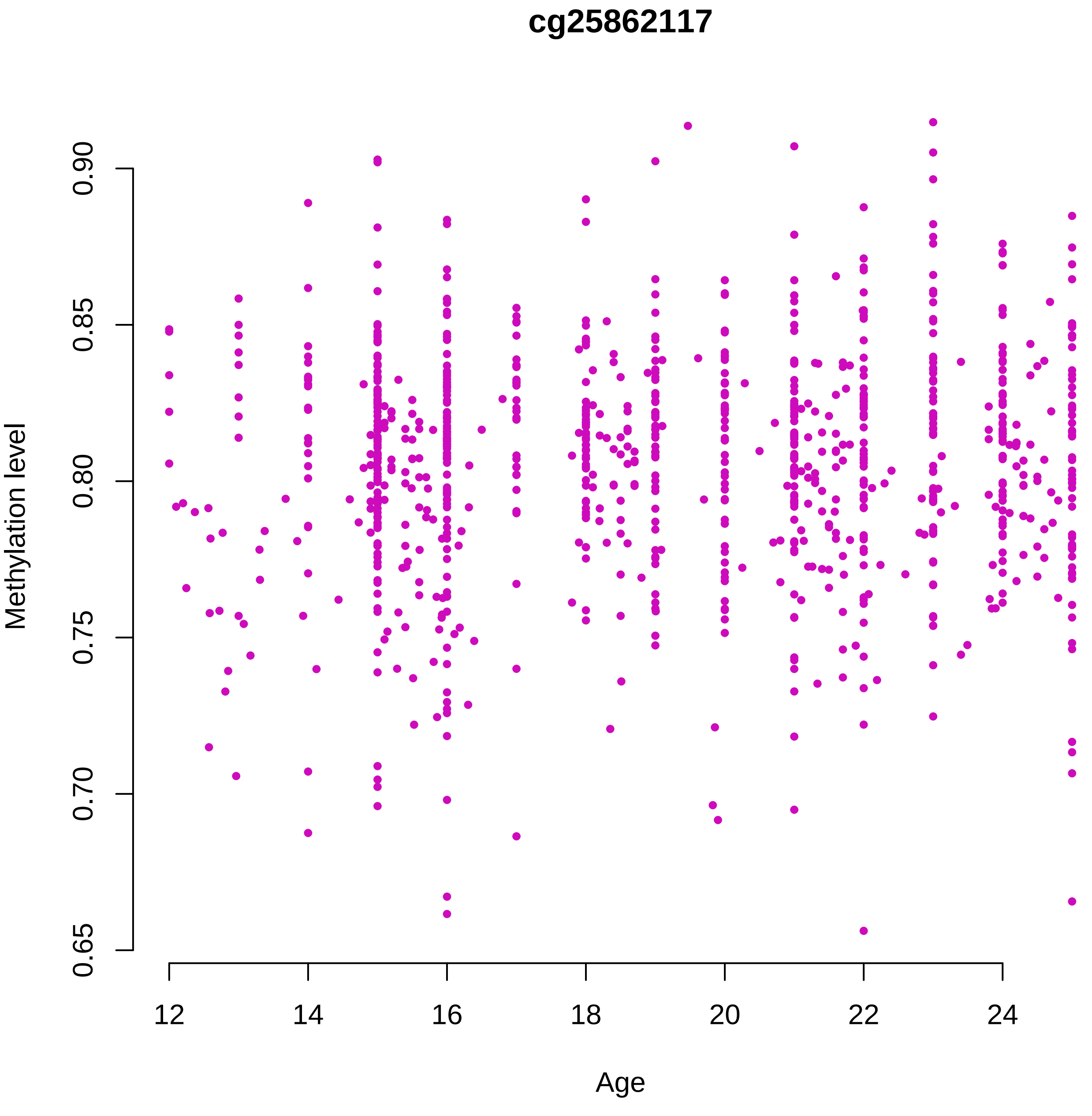
cg25862117
Methylation level
0.65
0.70
0.75
0.80
0.85
0.90
Age
12
14
16
18
20
22
24

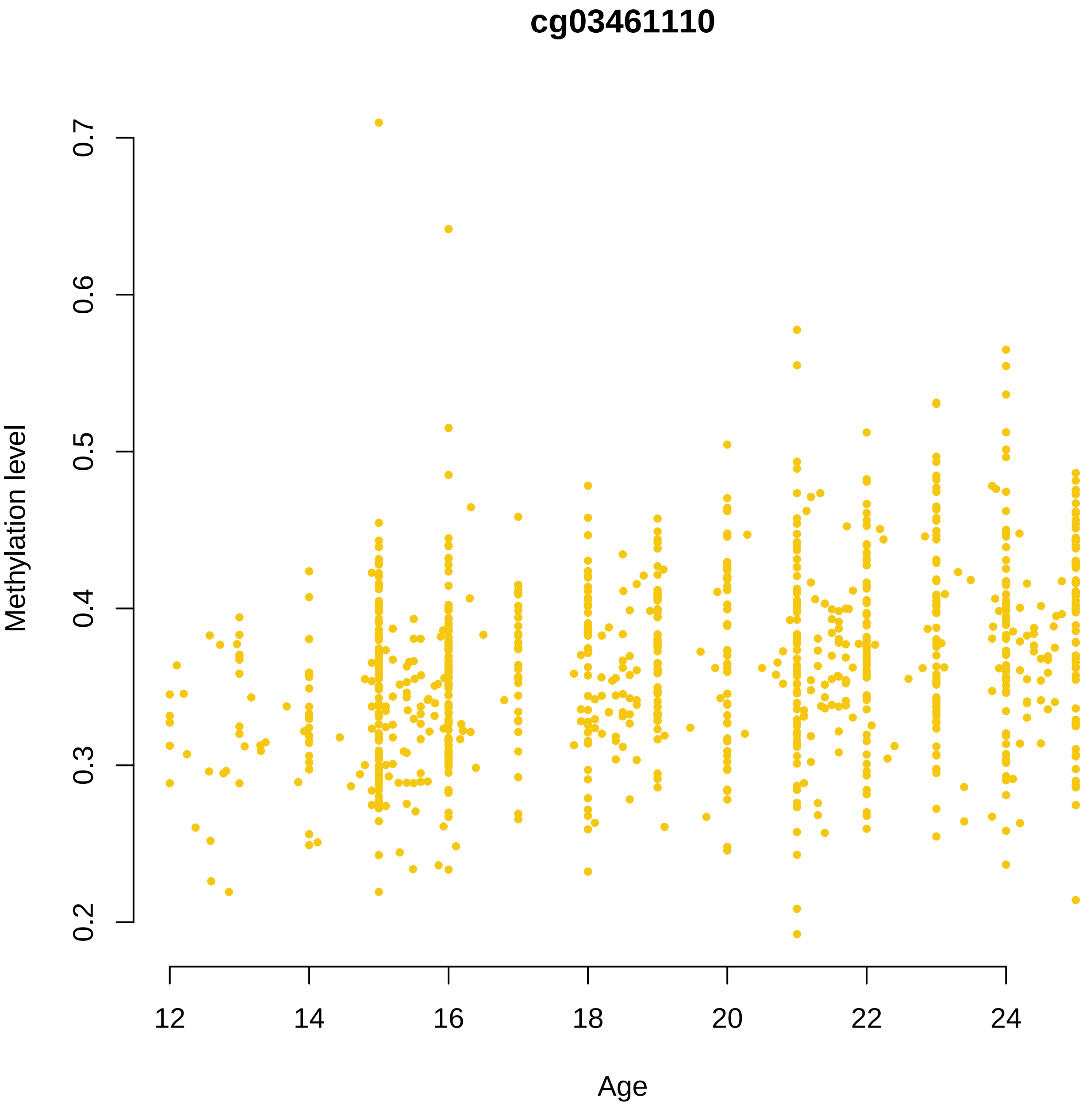
cg03461110
Methylation level
0.2
0.3
0.4
0.5
0.6
0.7
Age
12
14
16
18
20
22
24

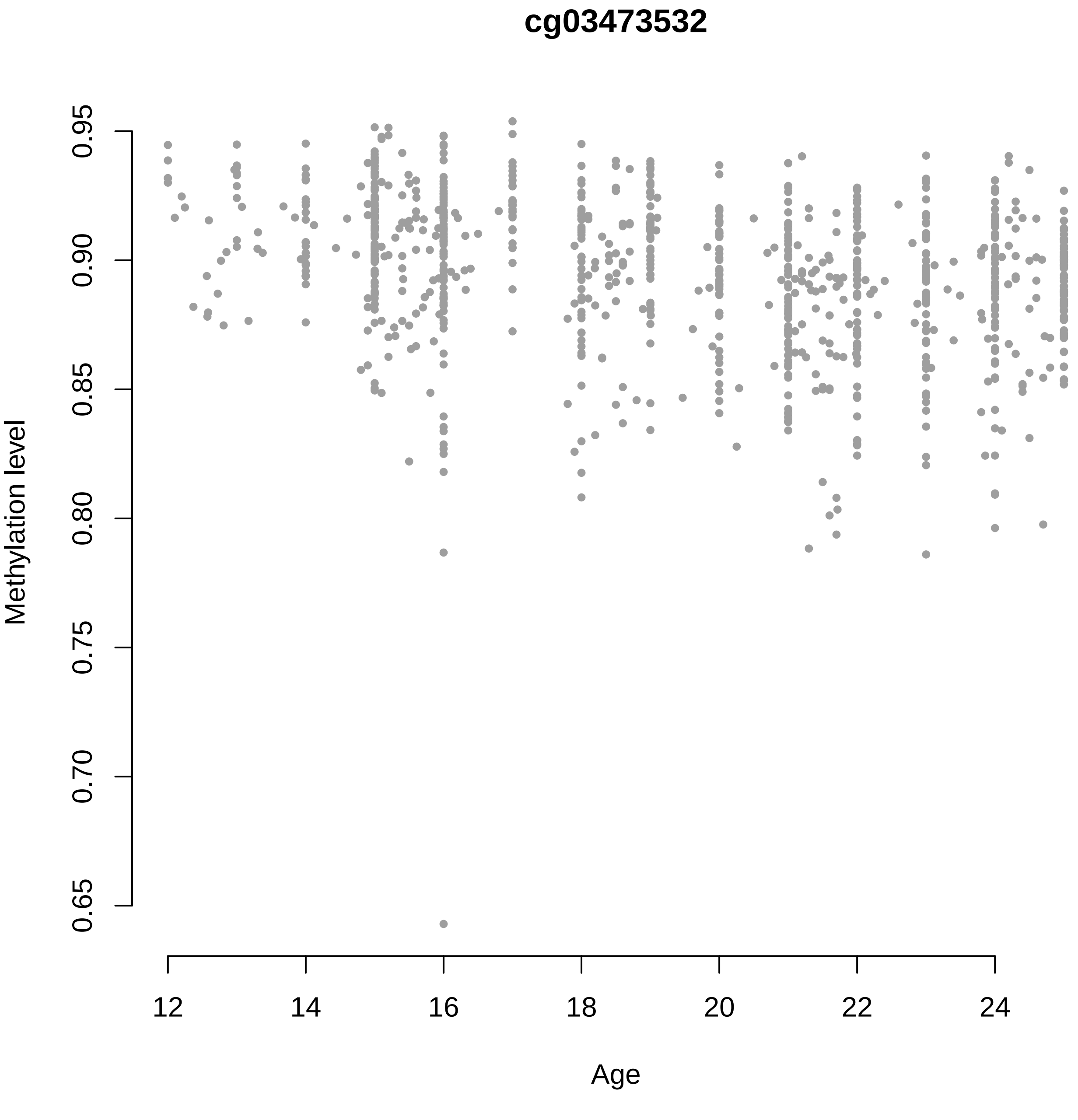
cg03473532
Methylation level
0.65
0.70
0.75
0.80
0.85
0.90
0.95
Age
12
14
16
18
20
22
24

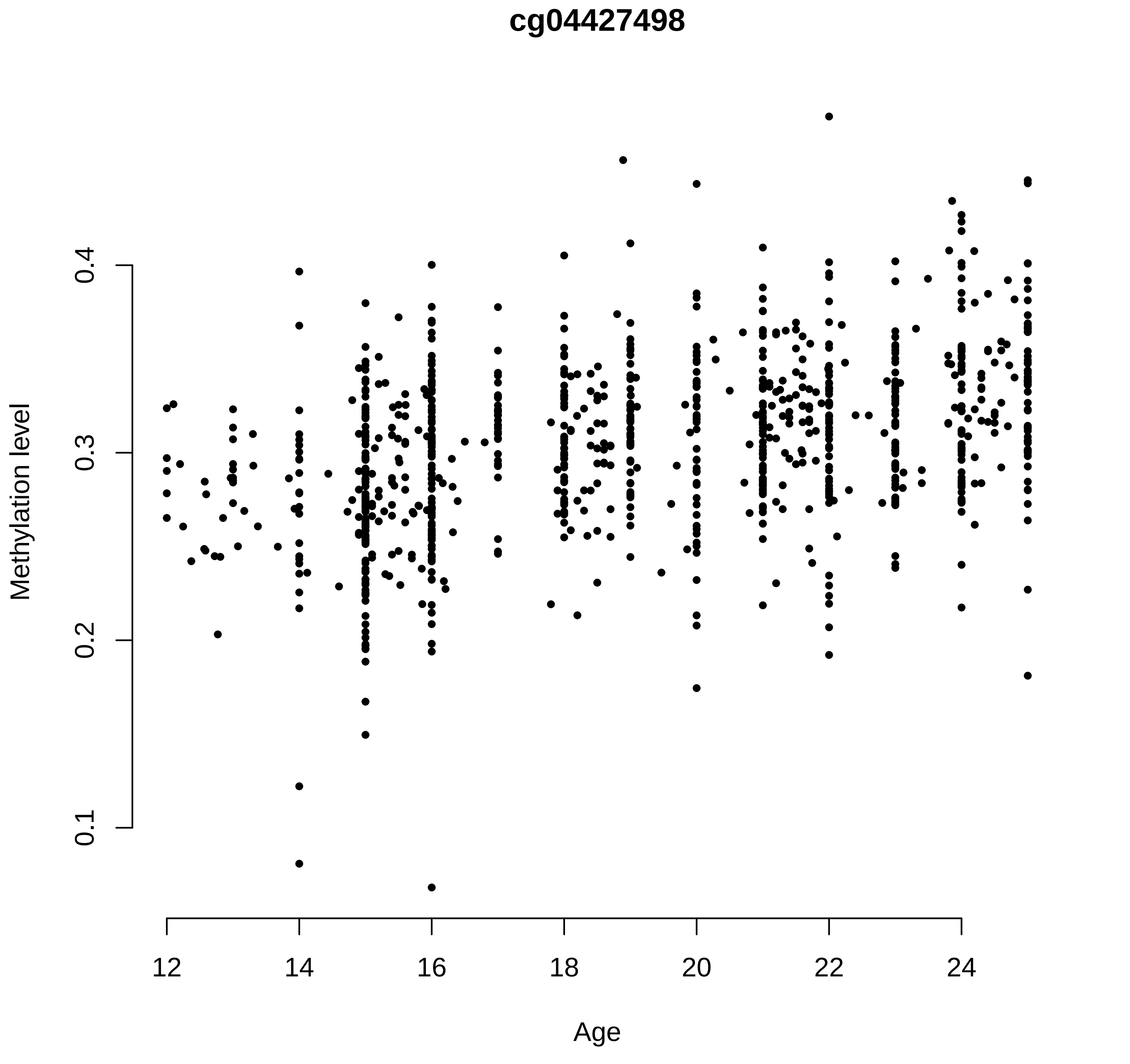
cg04427498
Methylation level
0.1
0.2
0.3
0.4
12
14
16
18
20
22
24
Age

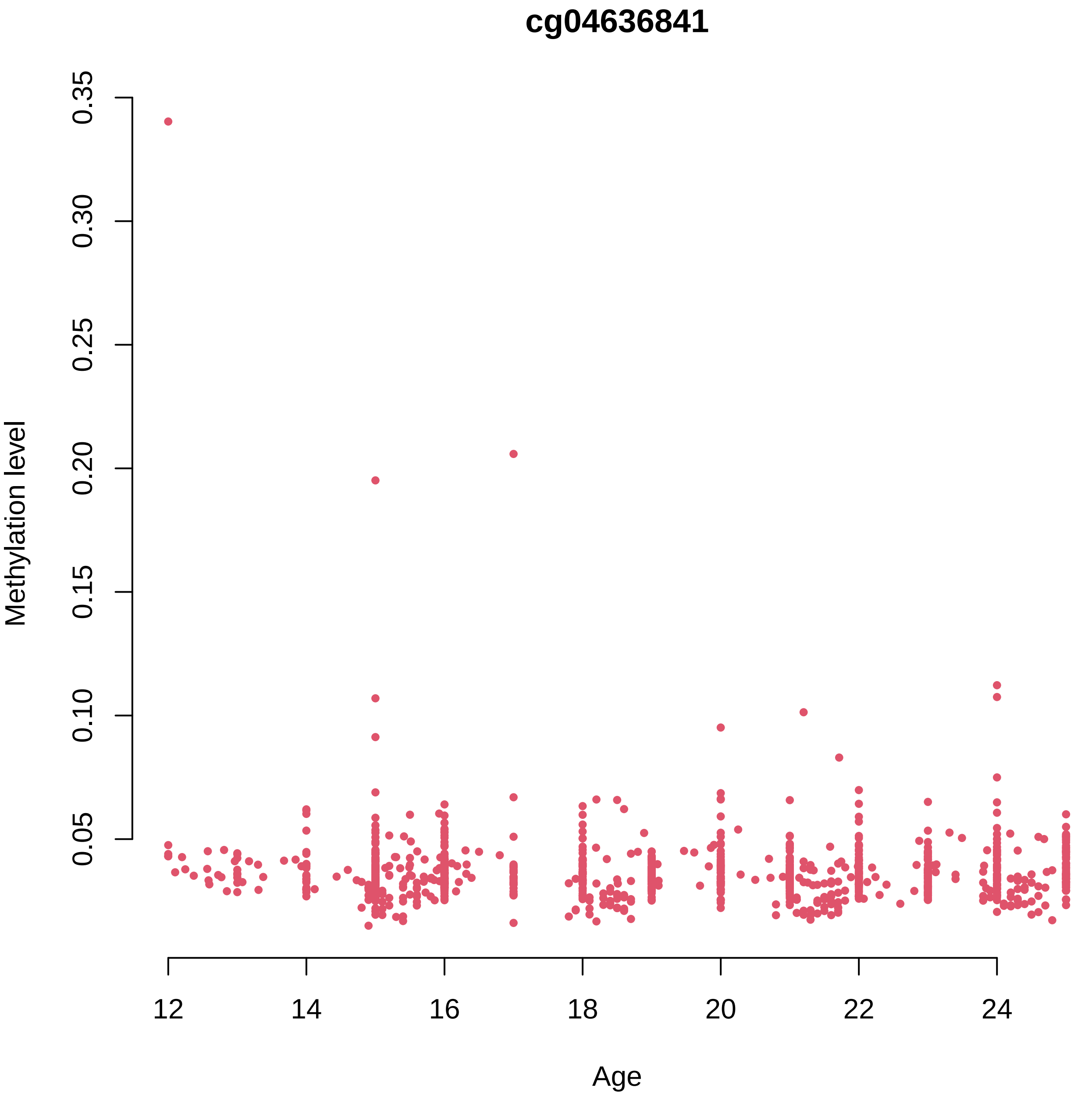
cg04636841
Methylation level
0.05
0.10
0.15
0.20
0.25
0.30
0.35
12
14
16
18
20
22
24
Age

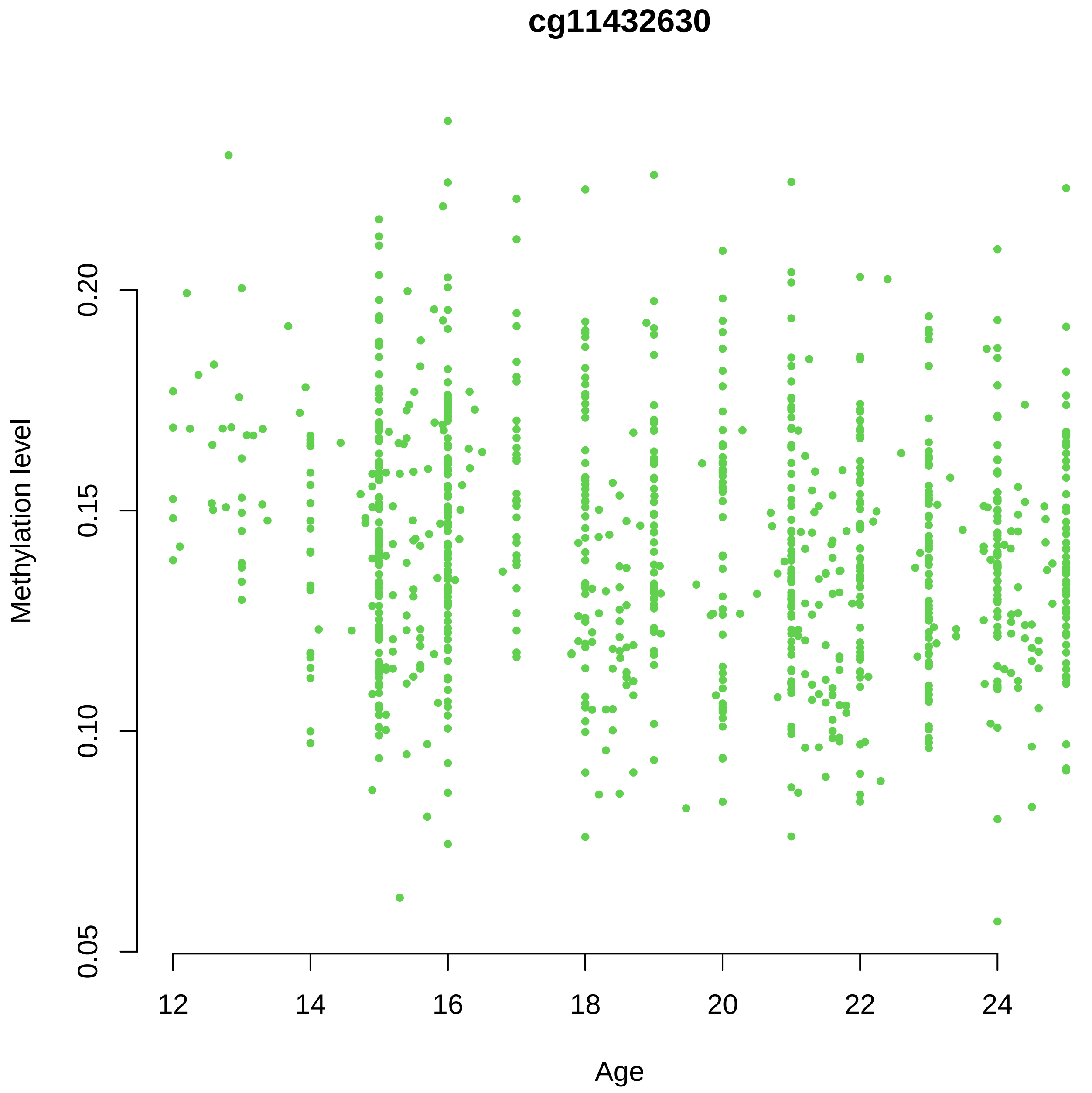
cg11432630
Methylation level
0.05
0.10
0.15
0.20
12
14
16
18
20
22
24
Age

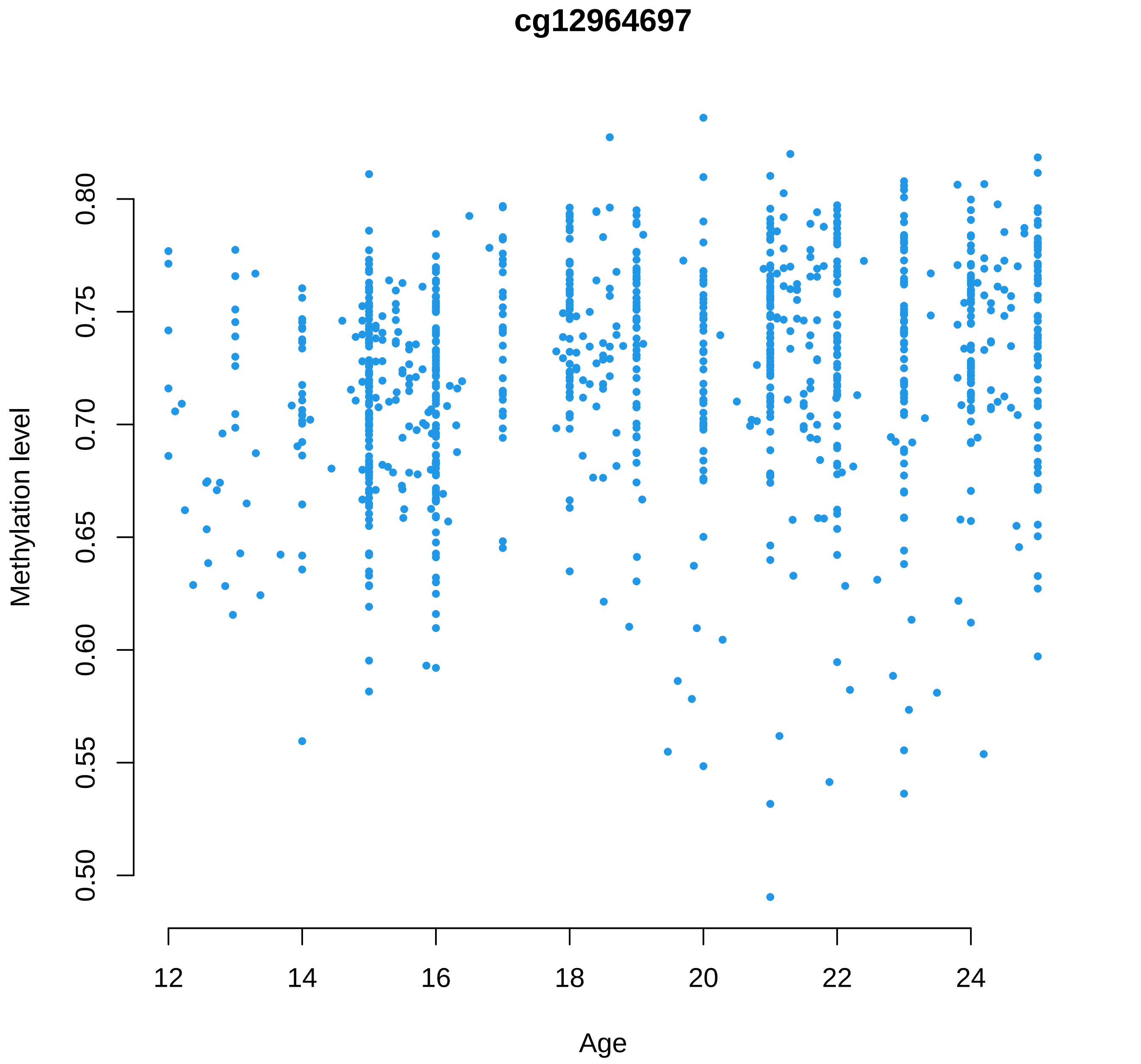
cg12964697
Methylation level
Age
0.50
0.55
0.60
0.65
0.70
0.75
0.80
12
14
16
18
20
22
24

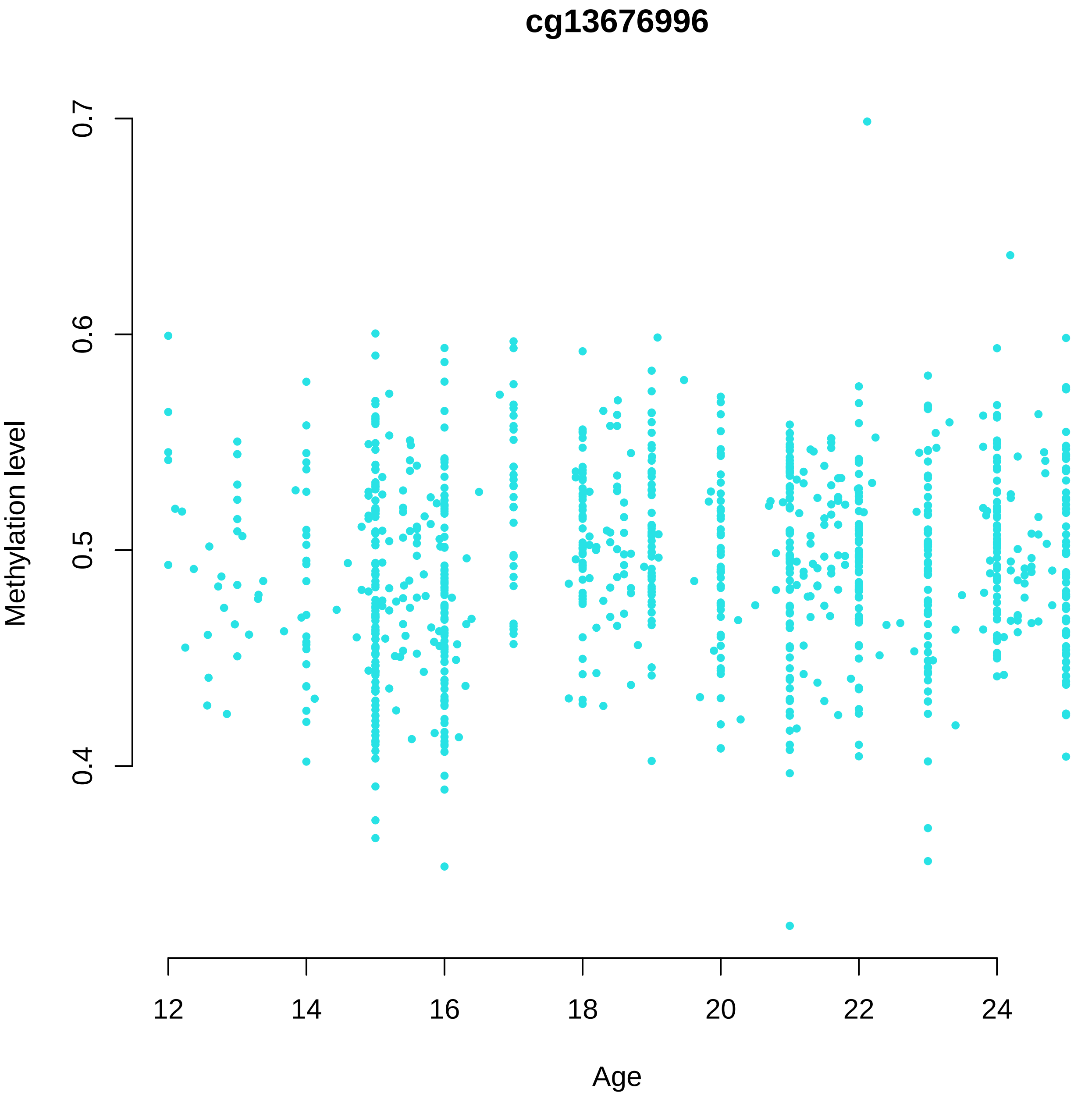

cg13676996
Methylation level
0.4
0.5
0.6
0.7
12
14
16
18
20
22
24
Age

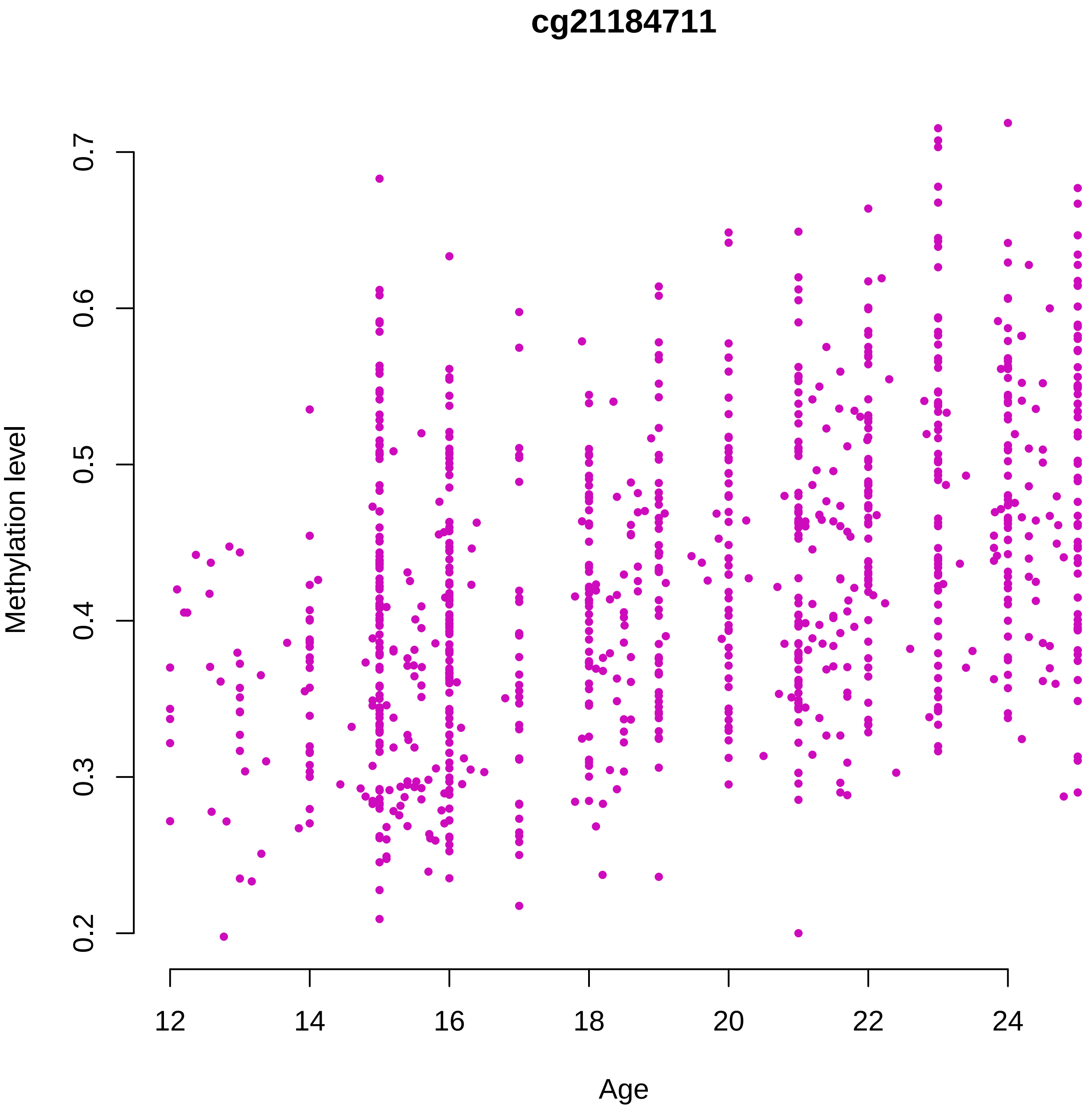

cg21184711
Methylation level
0.2
0.3
0.4
0.5
0.6
0.7
Age
12
14
16
18
20
22
24

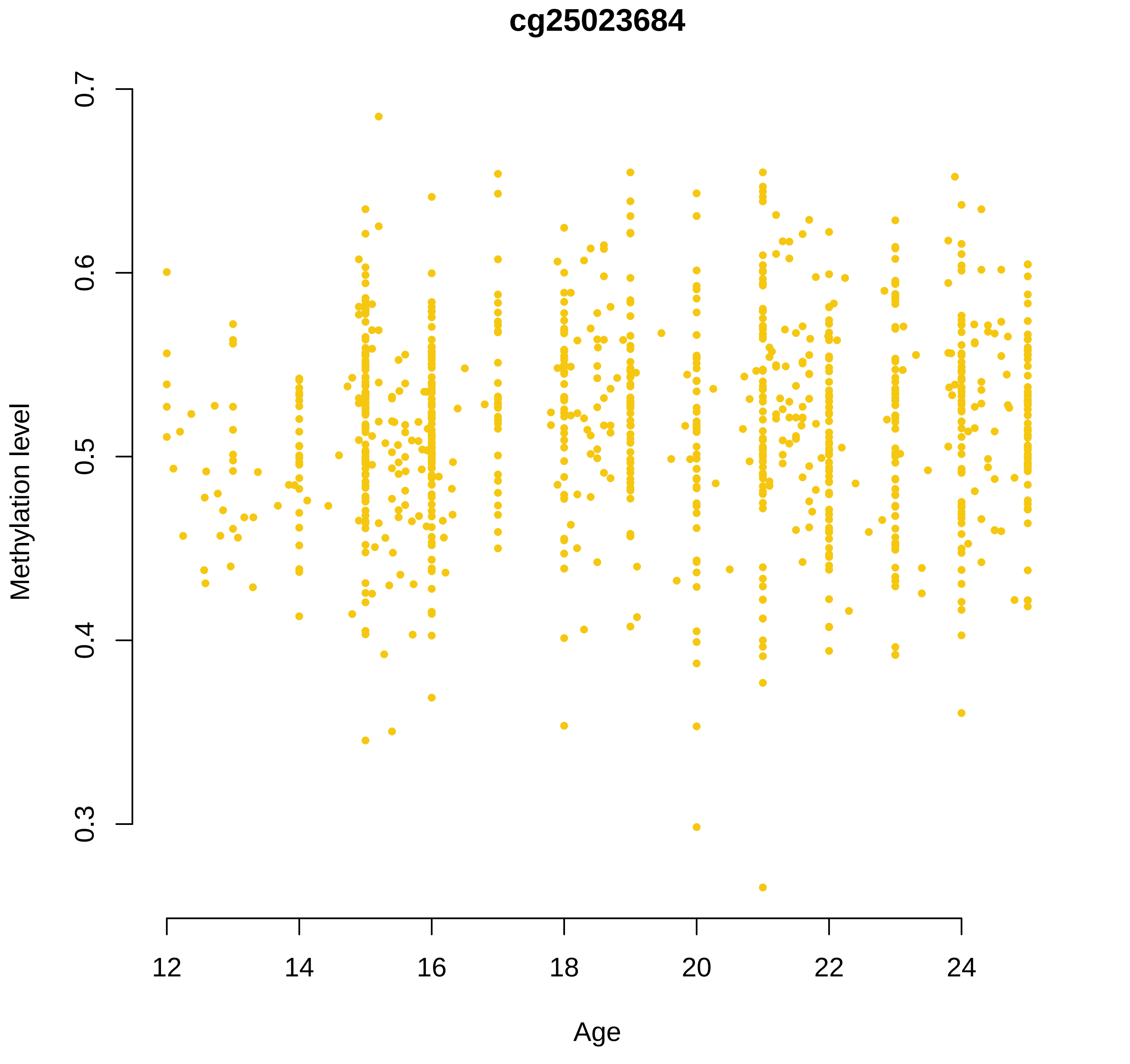
cg25023684
Methylation level
0.3
0.4
0.5
0.6
0.7
Age
12
14
16
18
20
22
24

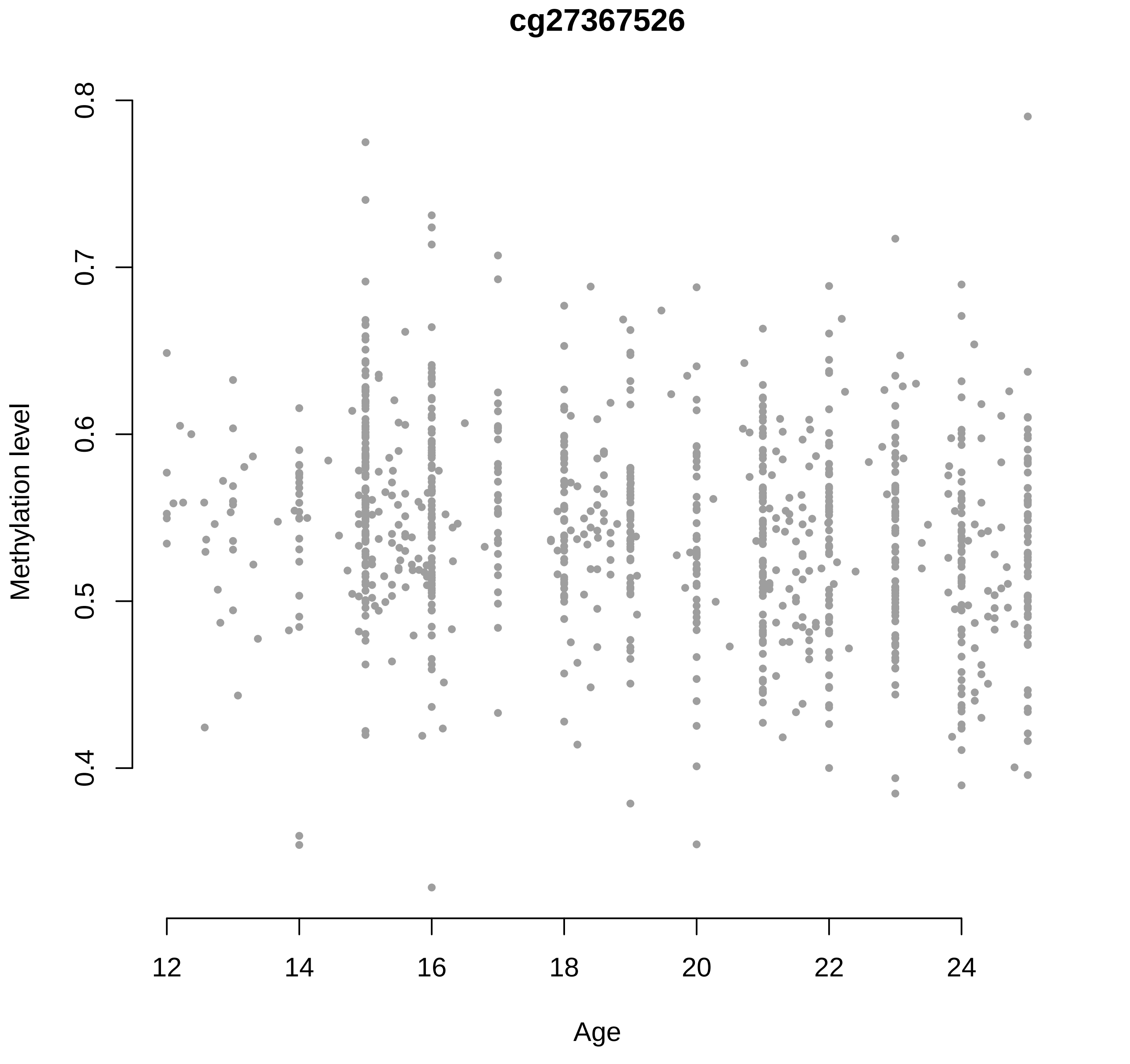
cg27367526
Methylation level
0.4
0.5
0.6
0.7
0.8
Age
12
14
16
18
20
22
24

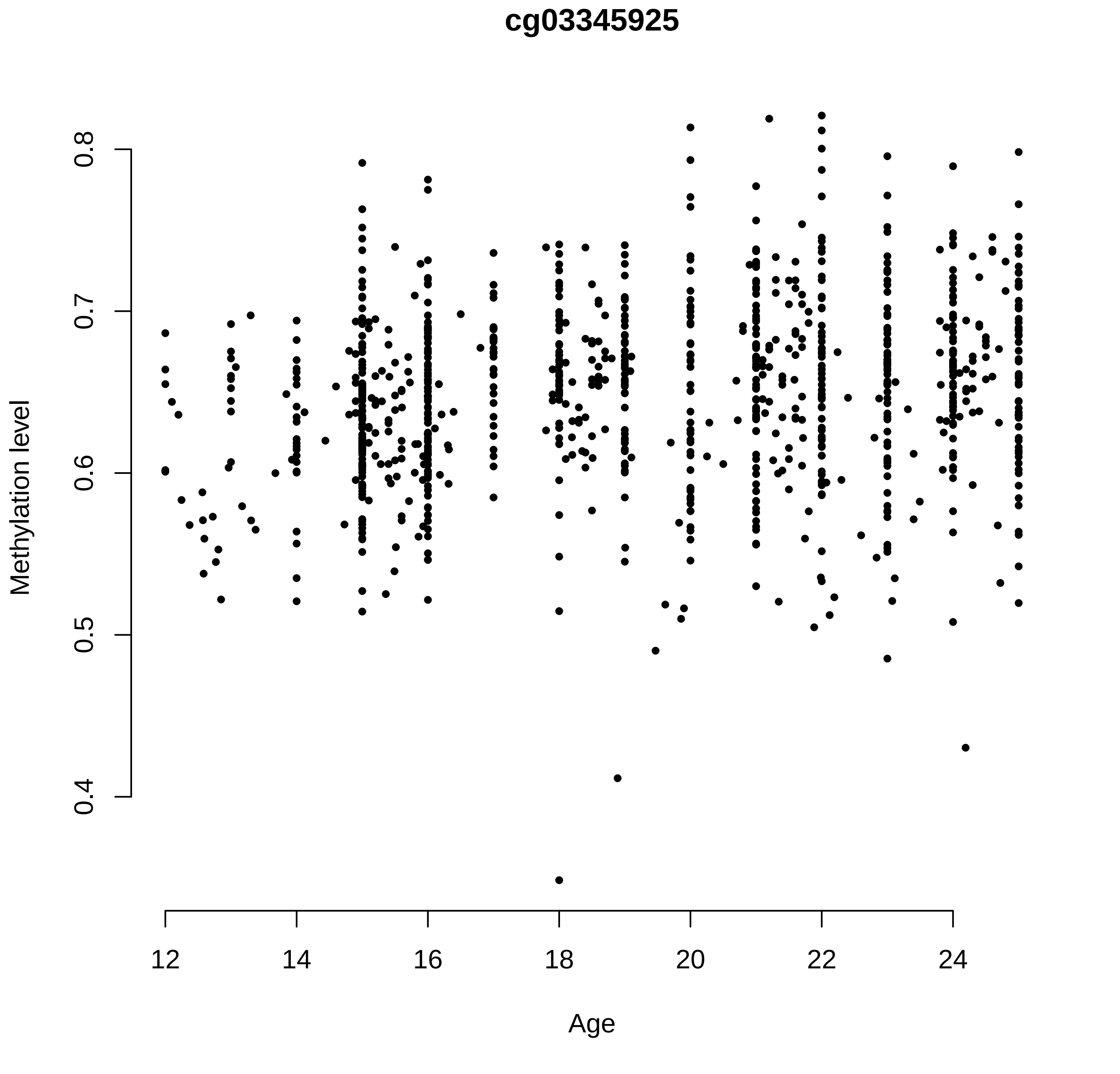
cg03345925
Methylation level
0.4
0.5
0.6
0.7
0.8
Age
12
14
16
18
20
22
24

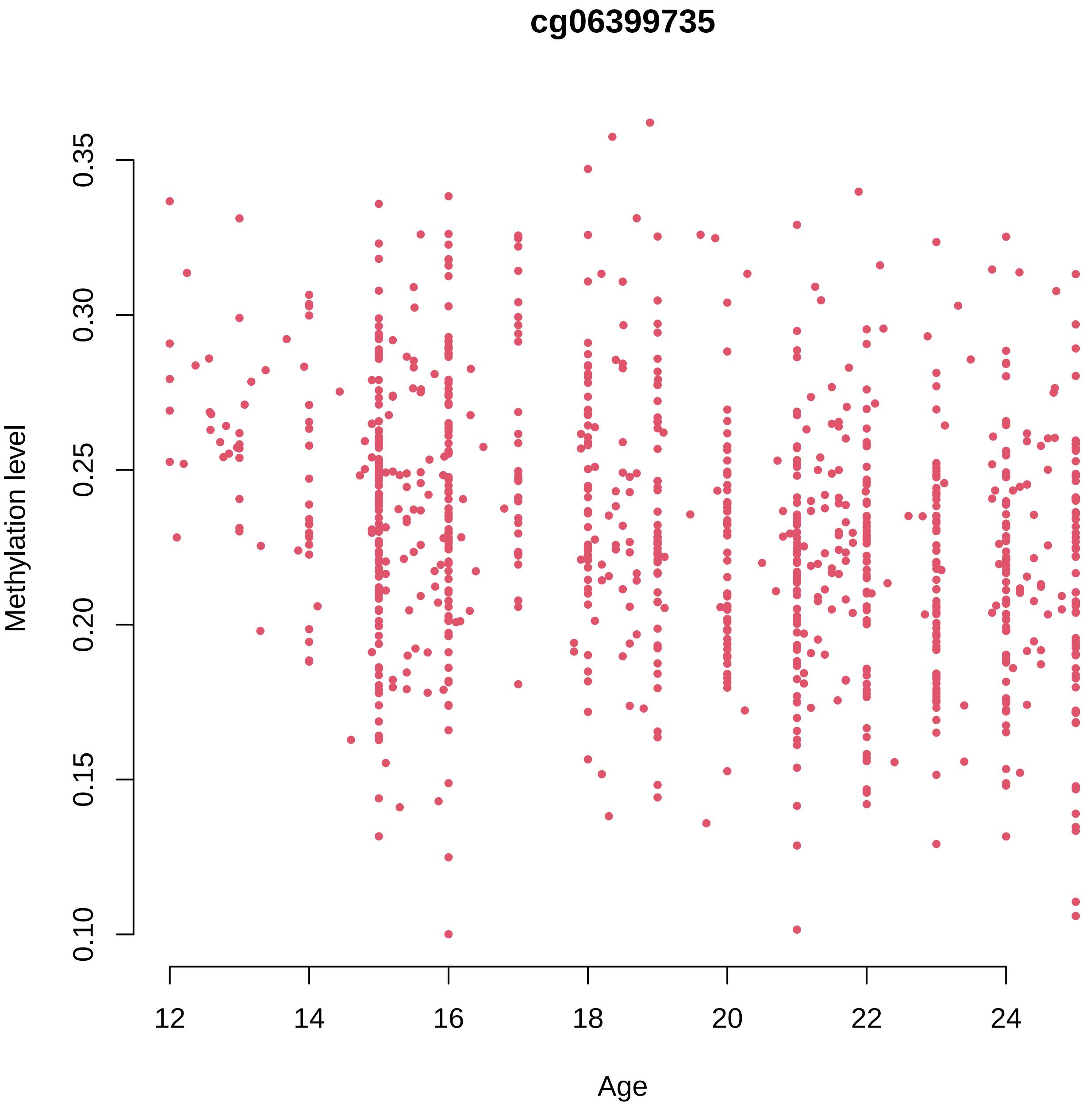
cg06399735
Methylation level
0.10
0.15
0.20
0.25
0.30
0.35
Age
12
14
16
18
20
22
24

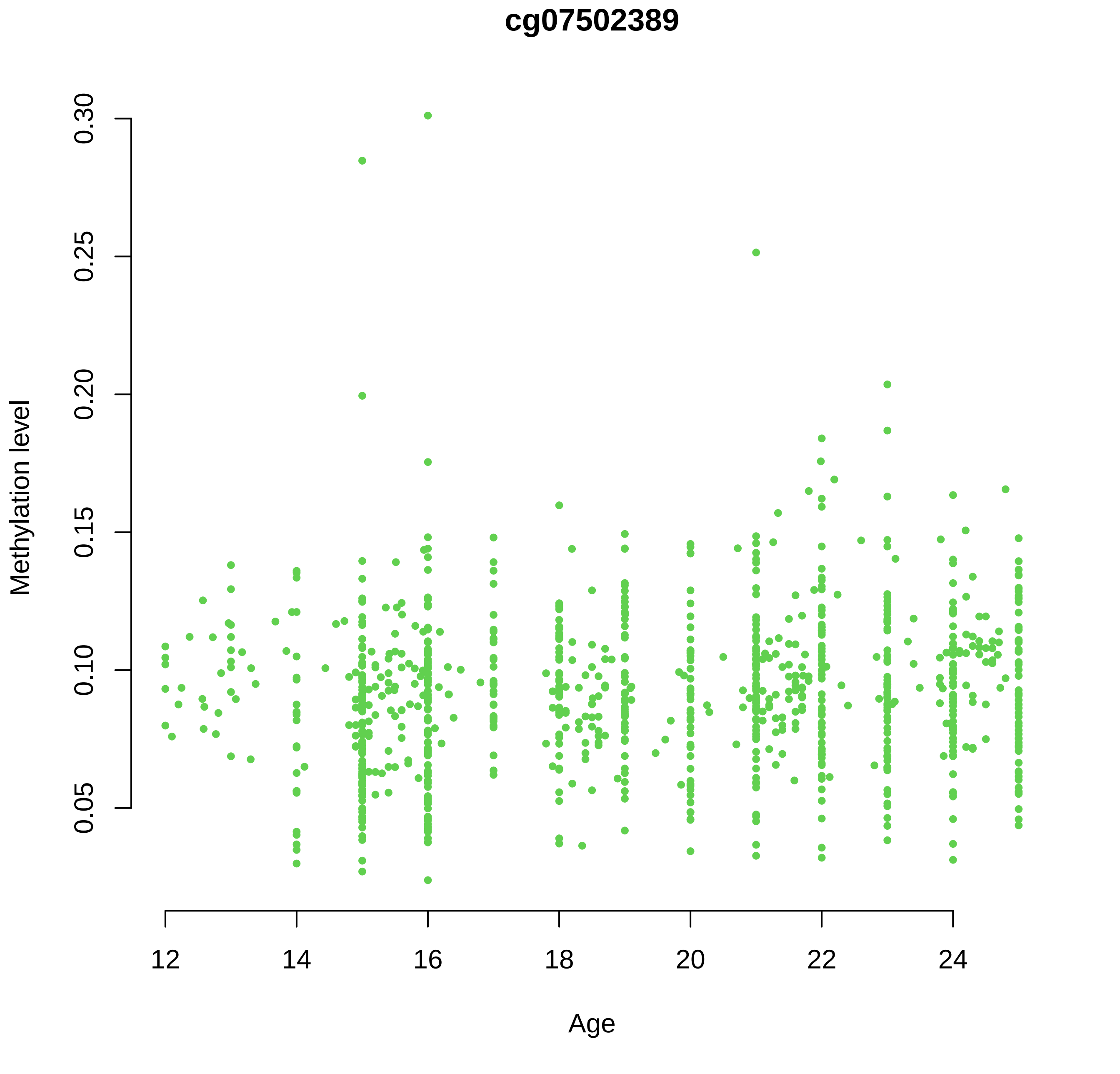

cg07502389
Methylation level
0.05
0.10
0.15
0.20
0.25
0.30
Age
12
14
16
18
20
22
24

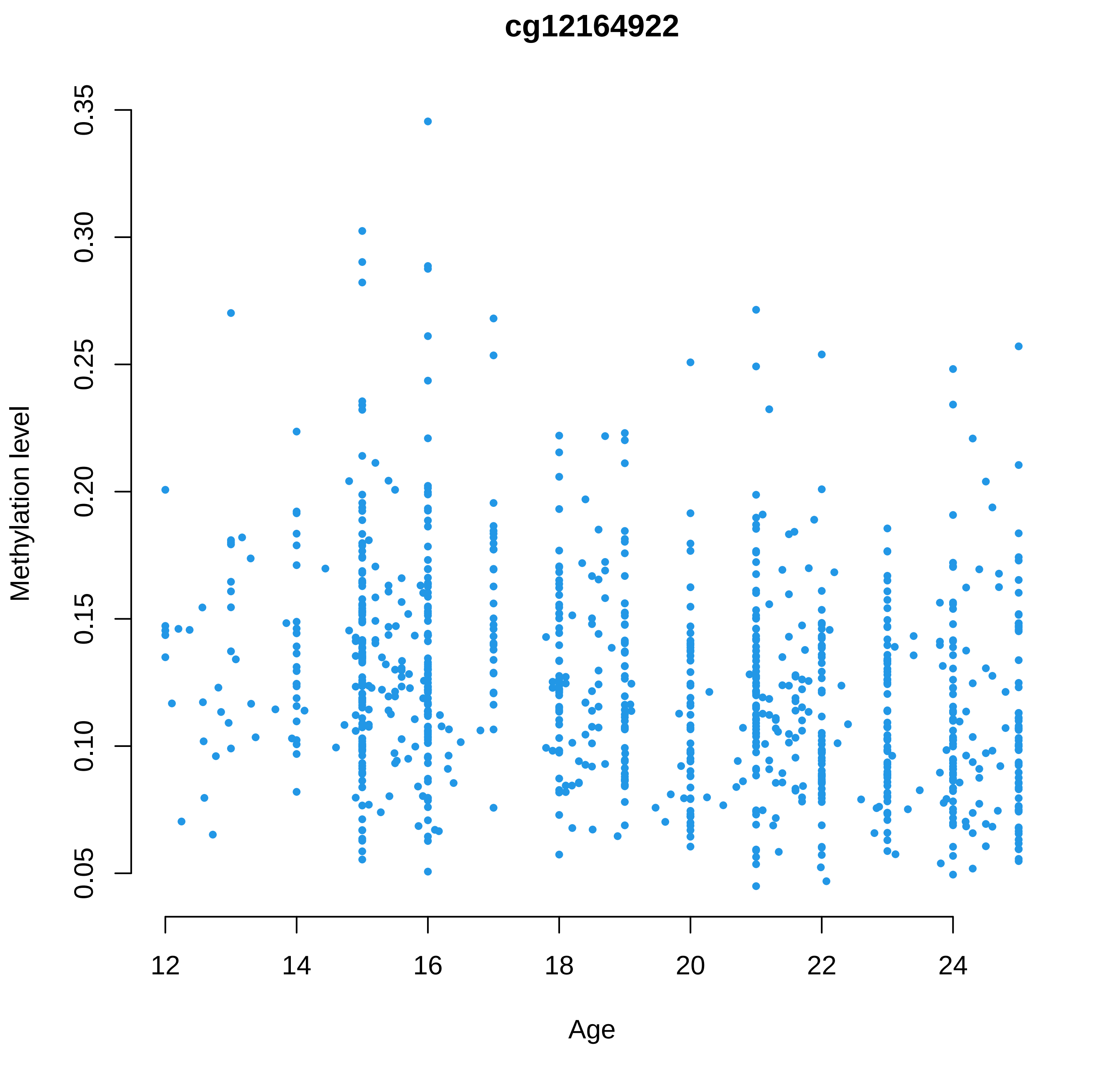

cg12164922
Methylation level
0.35
0.30
0.25
0.20
0.15
0.10
0.05
Age
12
14
16
18
20
22
24

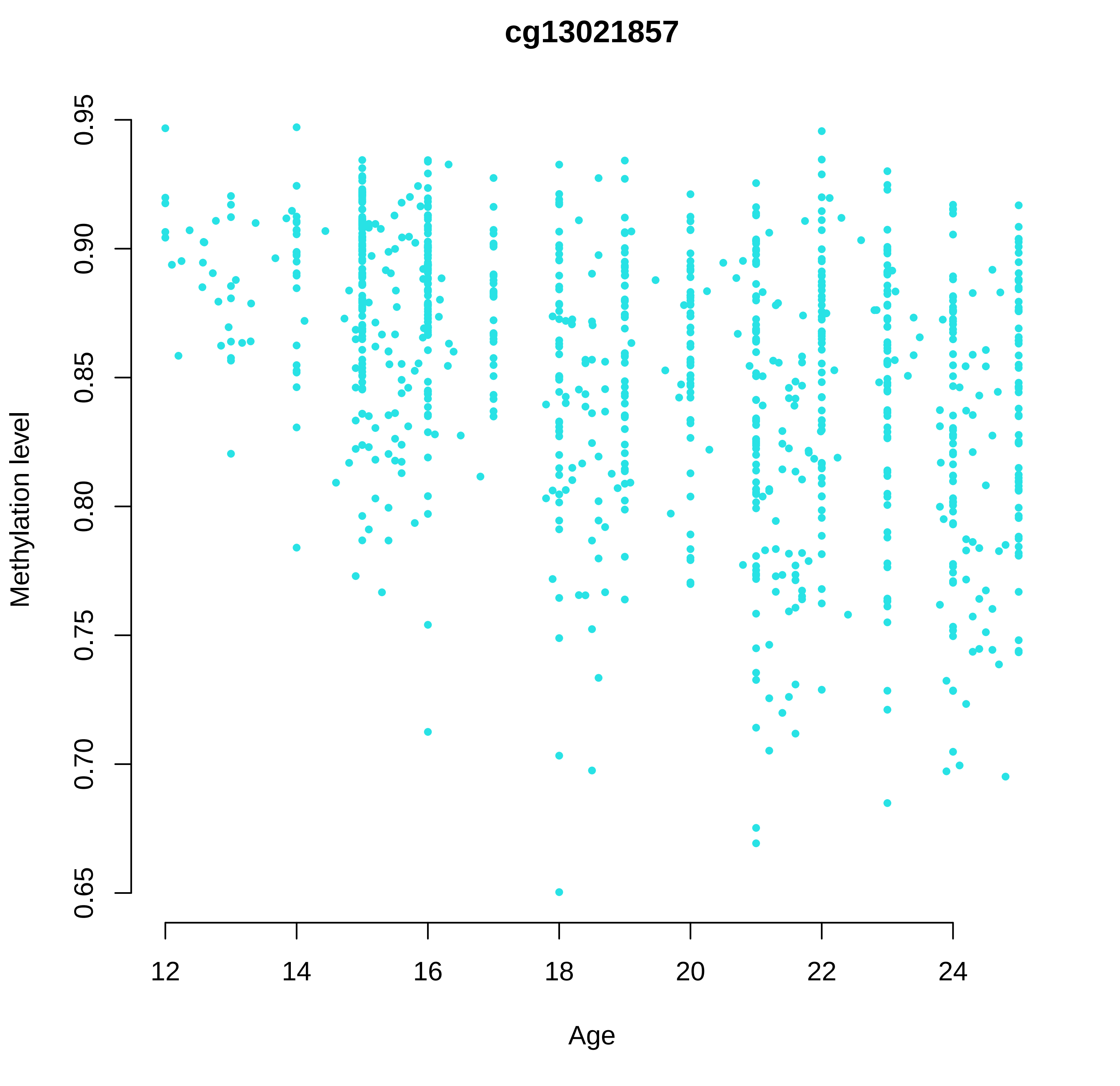
cg13021857
Methylation level
0.65
0.70
0.75
0.80
0.85
0.90
0.95
Age
12
14
16
18
20
22
24

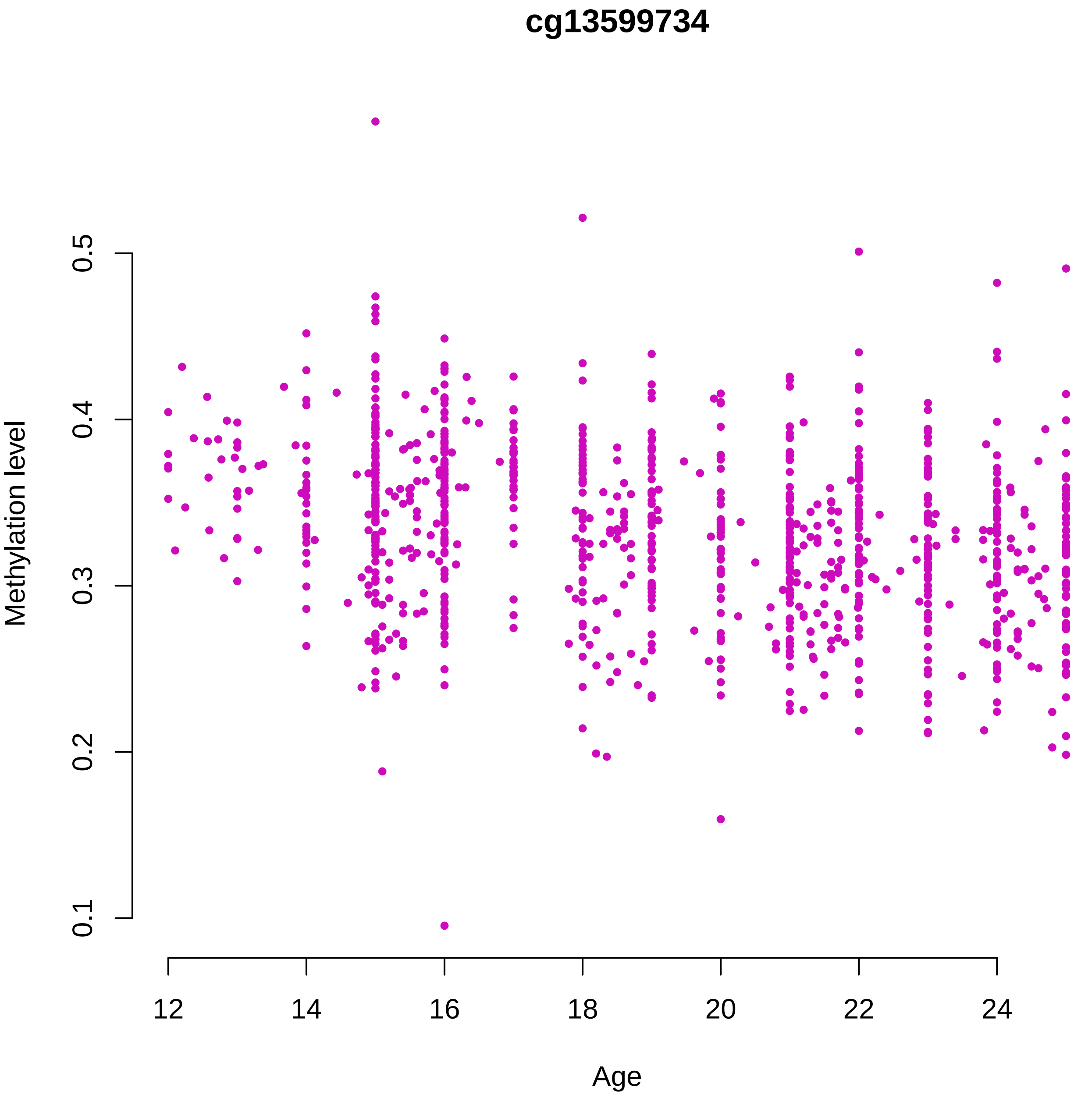
cg13599734
Methylation level
0.1
0.2
0.3
0.4
0.5
12
14
16
18
20
22
24
Age

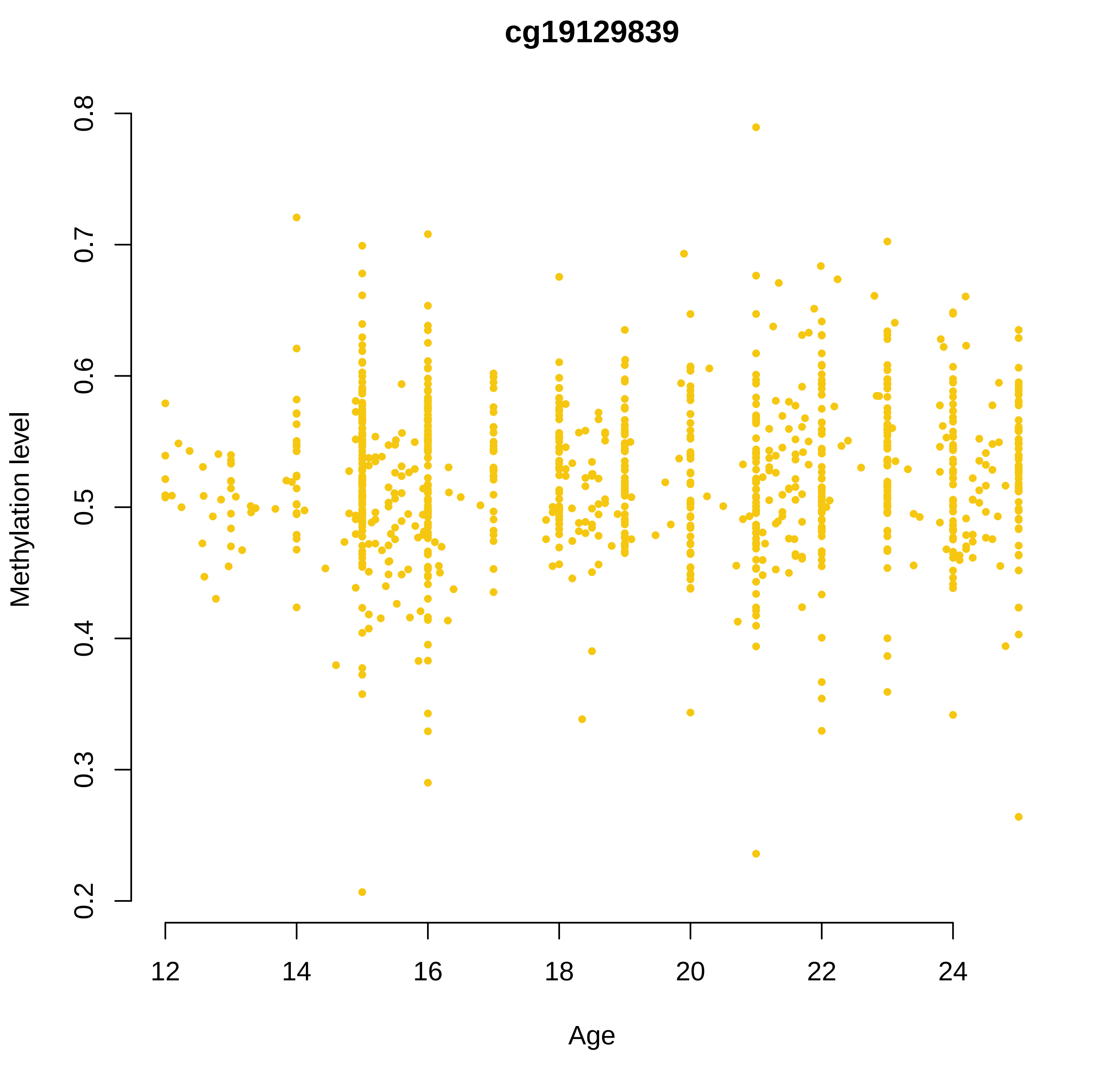

cg19129839
Methylation level
0.2
0.3
0.4
0.5
0.6
0.7
0.8
Age
12
14
16
18
20
22
24

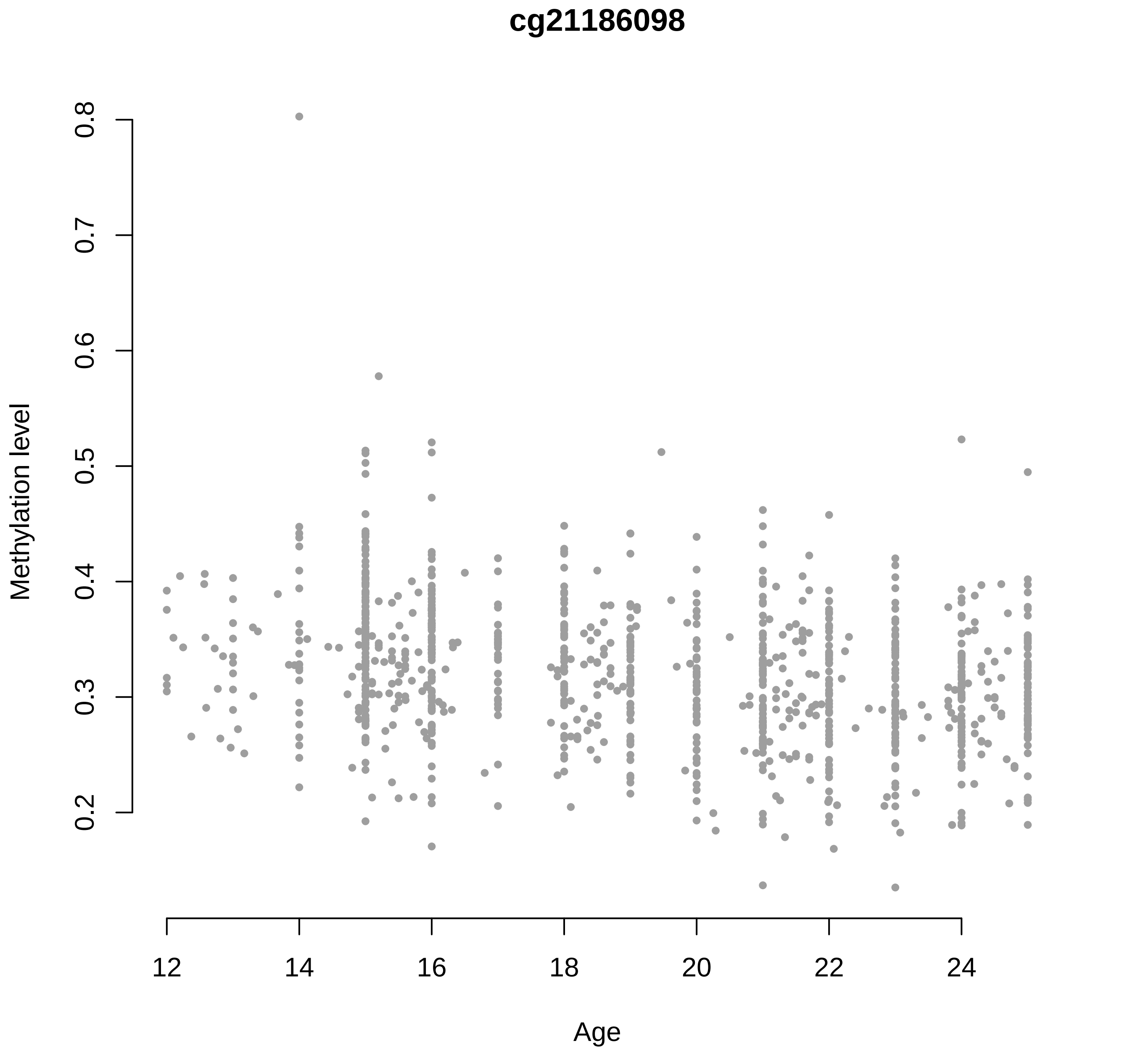
cg21186098
Methylation level
0.2
0.3
0.4
0.5
0.6
0.7
0.8
Age
12
14
16
18
20
22
24

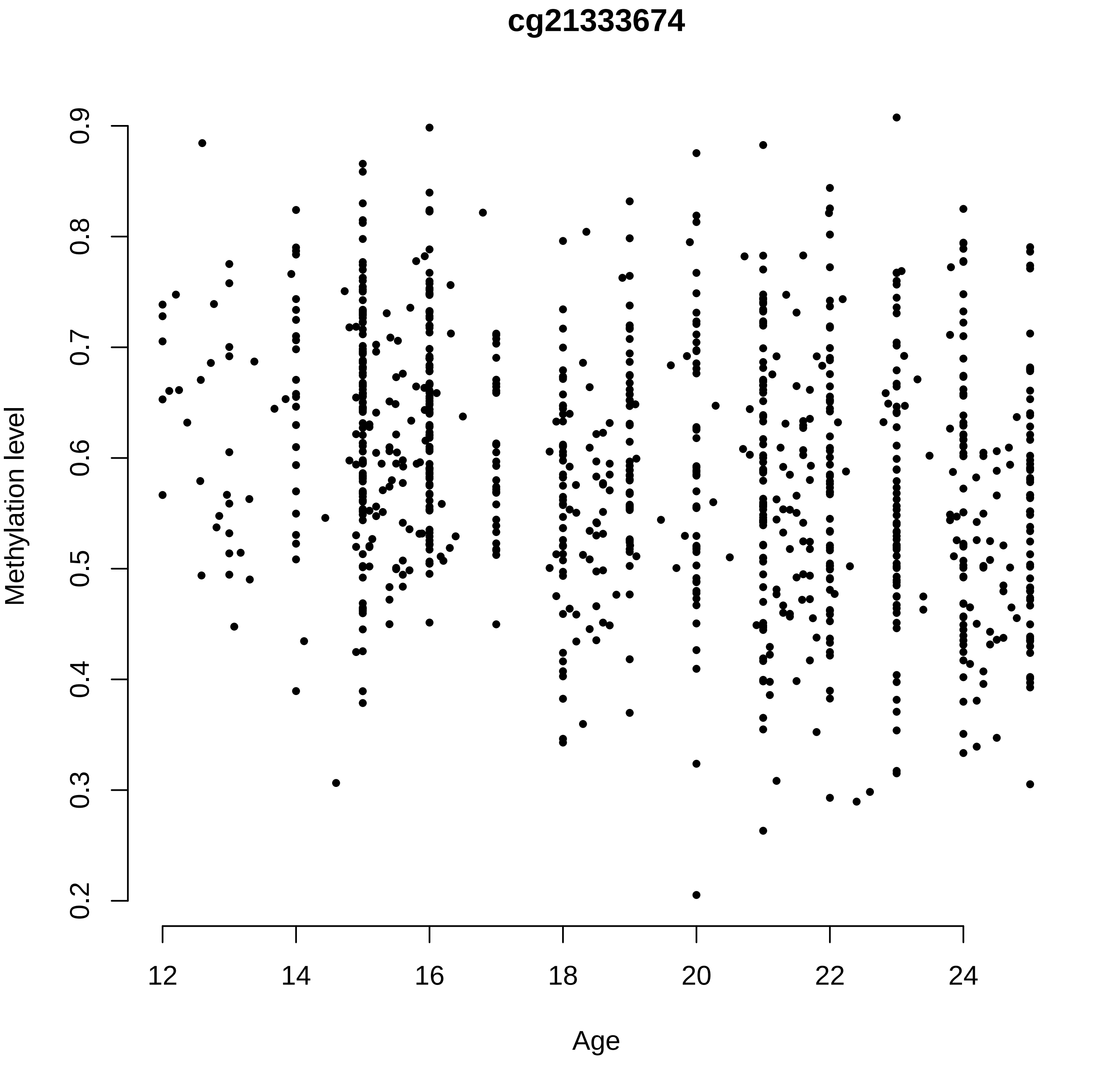

cg21333674
Methylation level
0.2
0.3
0.4
0.5
0.6
0.7
0.8
0.9
Age
12
14
16
18
20
22
24

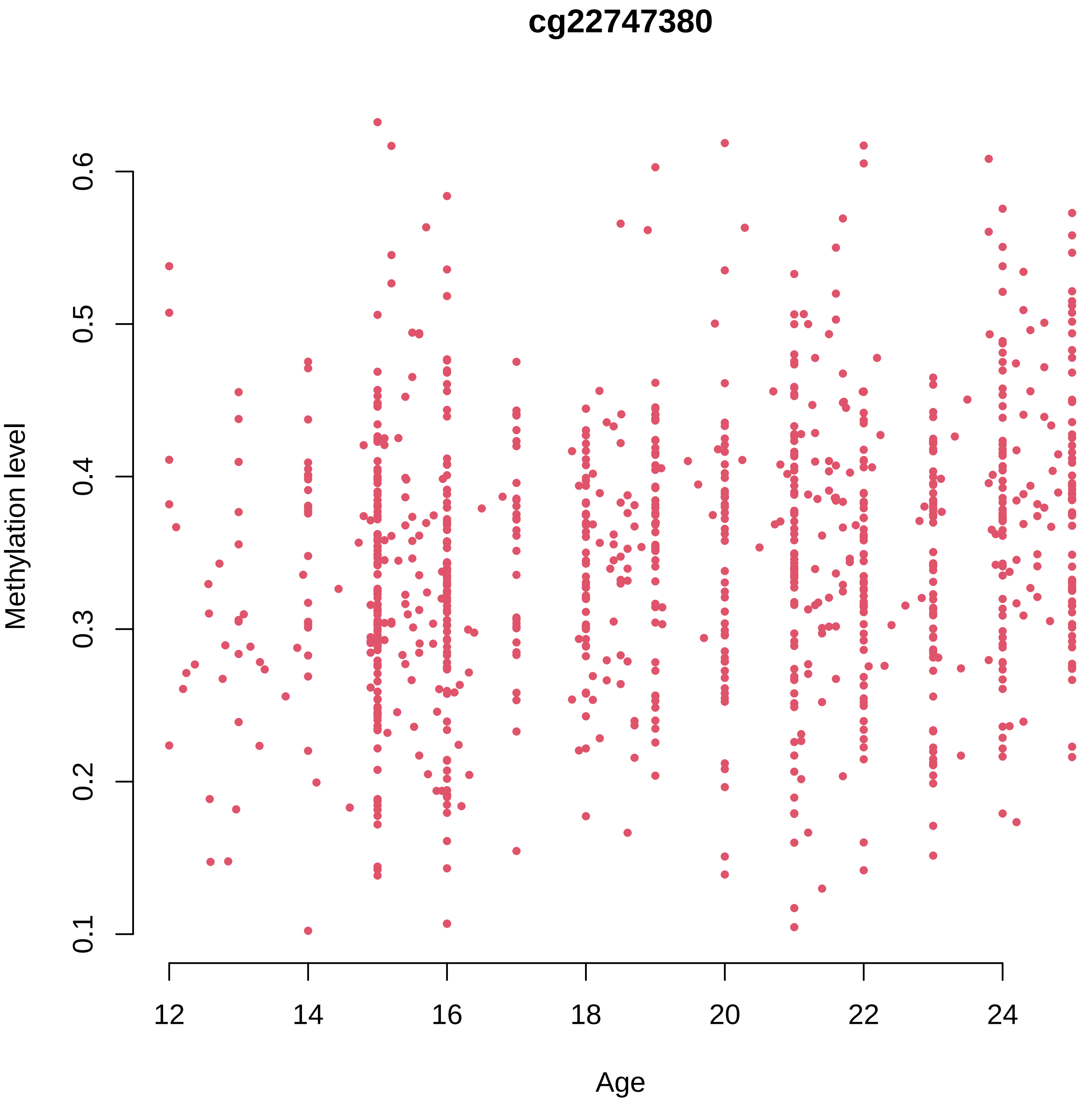
cg22747380
Methylation level
0.1
0.2
0.3
0.4
0.5
0.6
12
14
16
18
20
22
24
Age

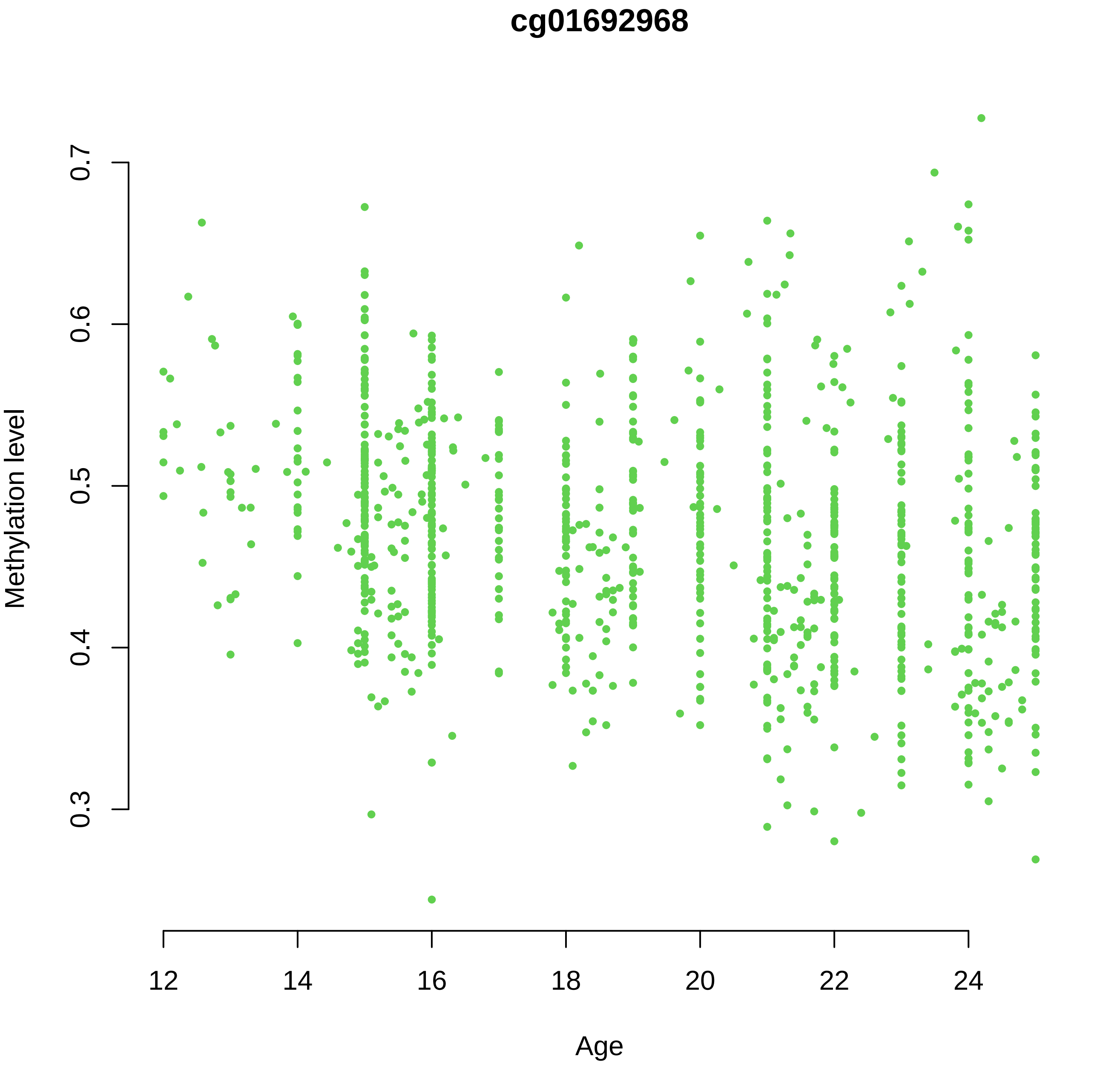
cg01692968
Methylation level
0.3
0.4
0.5
0.6
0.7
Age
12
14
16
18
20
22
24

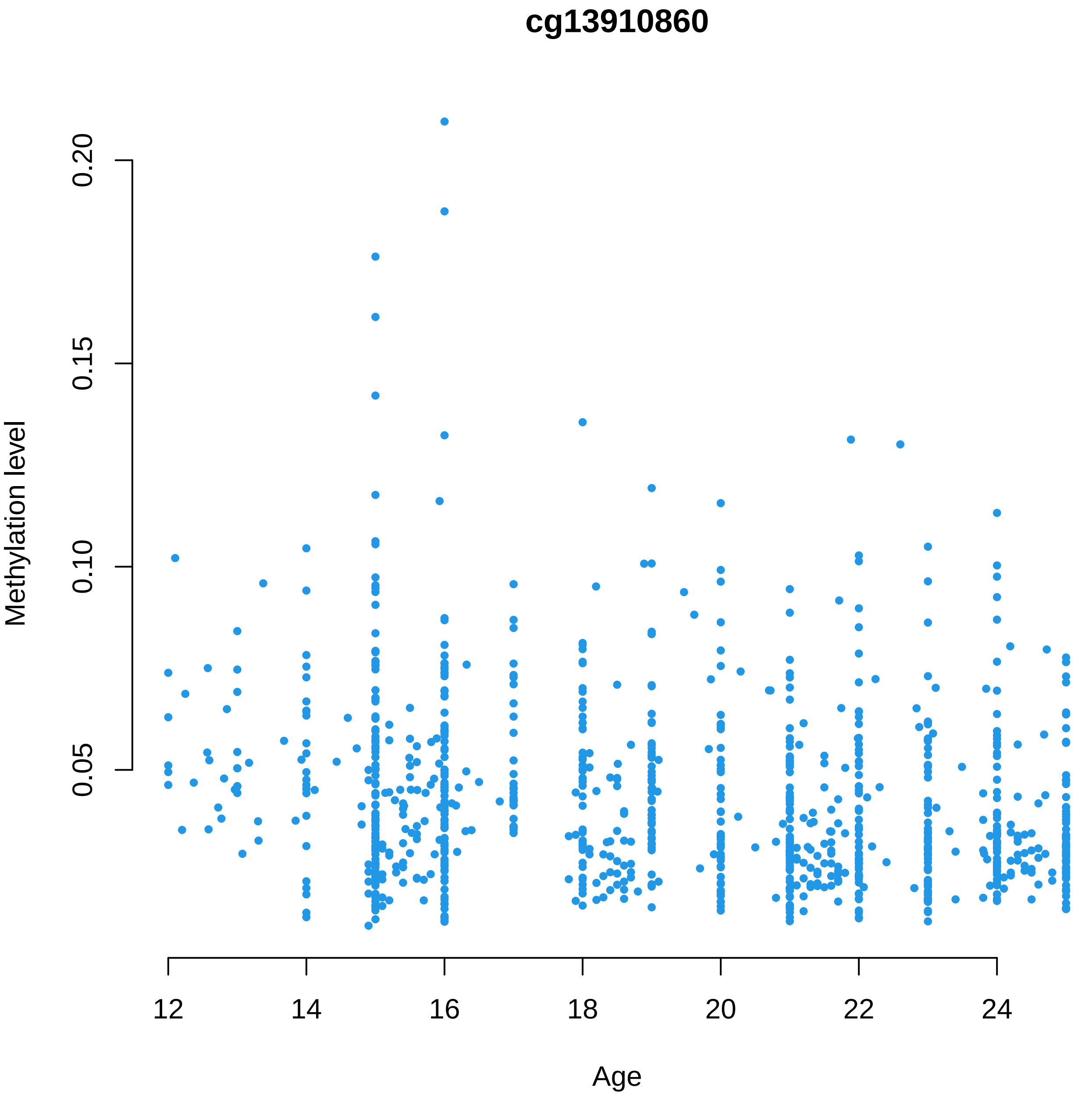
cg13910860
Methylation level
0.05
0.10
0.15
0.20
Age
12
14
16
18
20
22
24

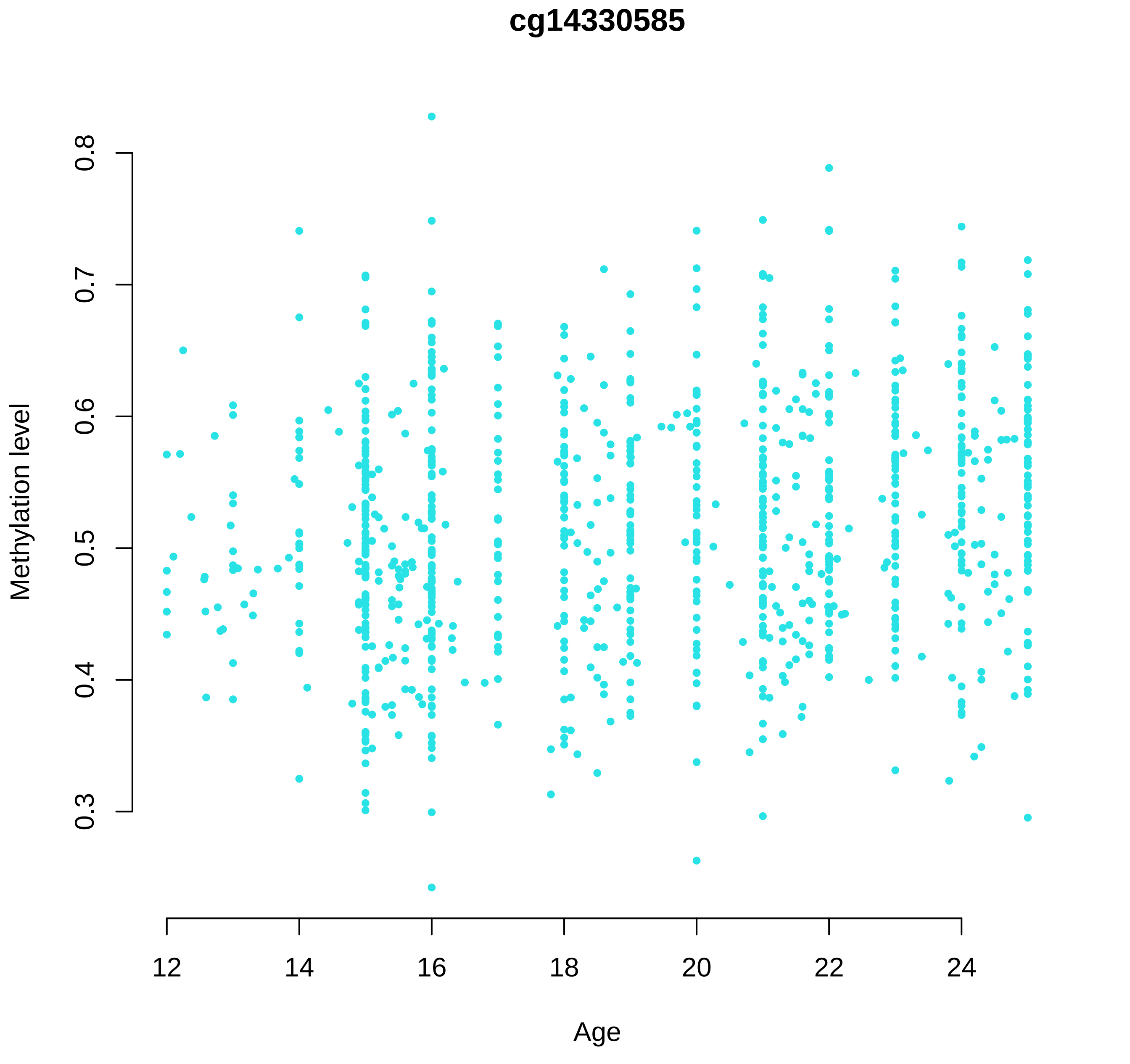
cg14330585
Methylation level
0.3
0.4
0.5
0.6
0.7
0.8
Age
12
14
16
18
20
22
24

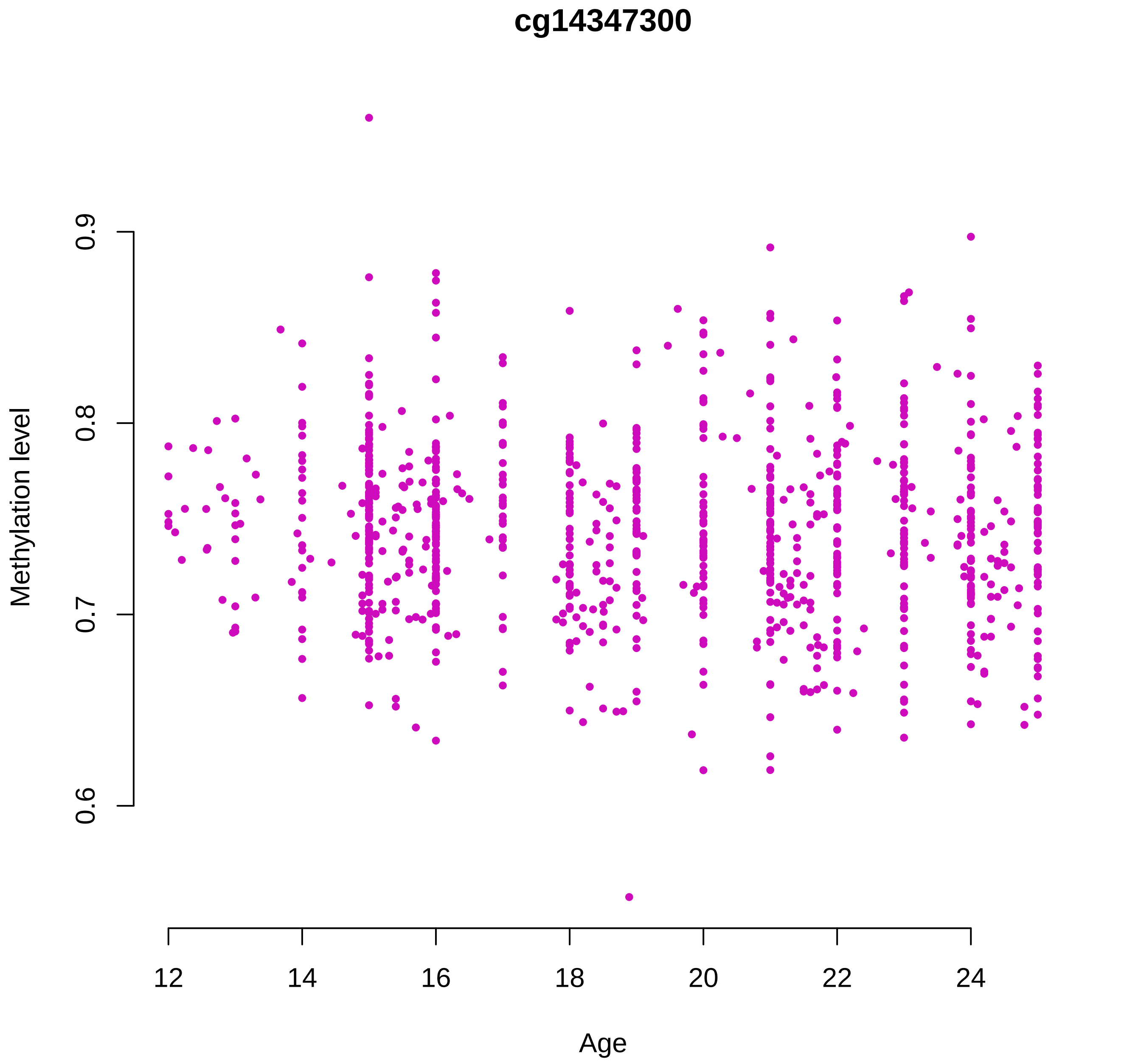

cg14347300
Methylation level
0.6
0.7
0.8
0.9
Age
12
14
16
18
20
22
24

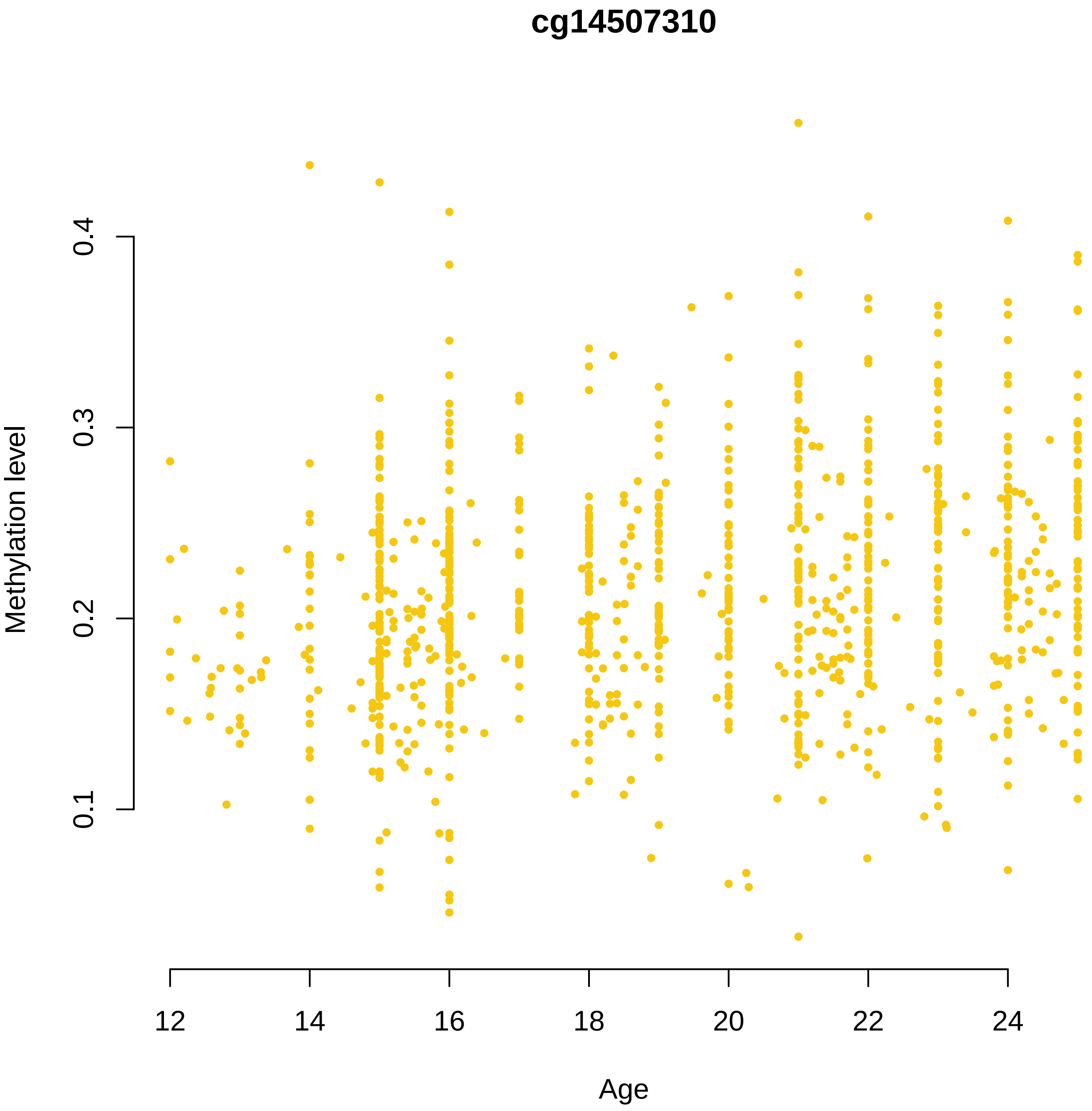

cg14507310
Methylation level
0.1
0.2
0.3
0.4
Age
12
14
16
18
20
22
24

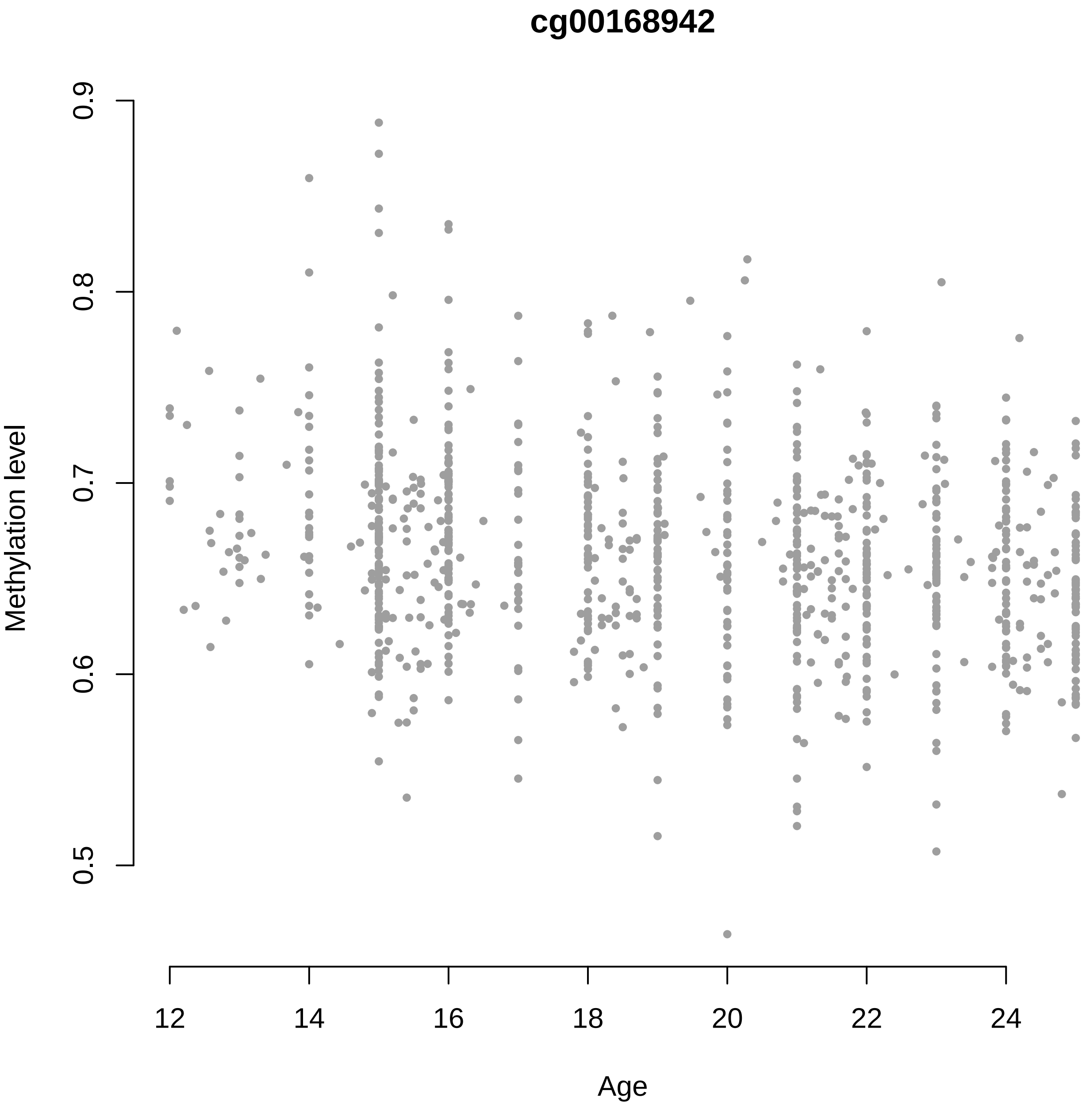

cg00168942
Methylation level
0.5
0.6
0.7
0.8
0.9
Age
12
14
16
18
20
22
24

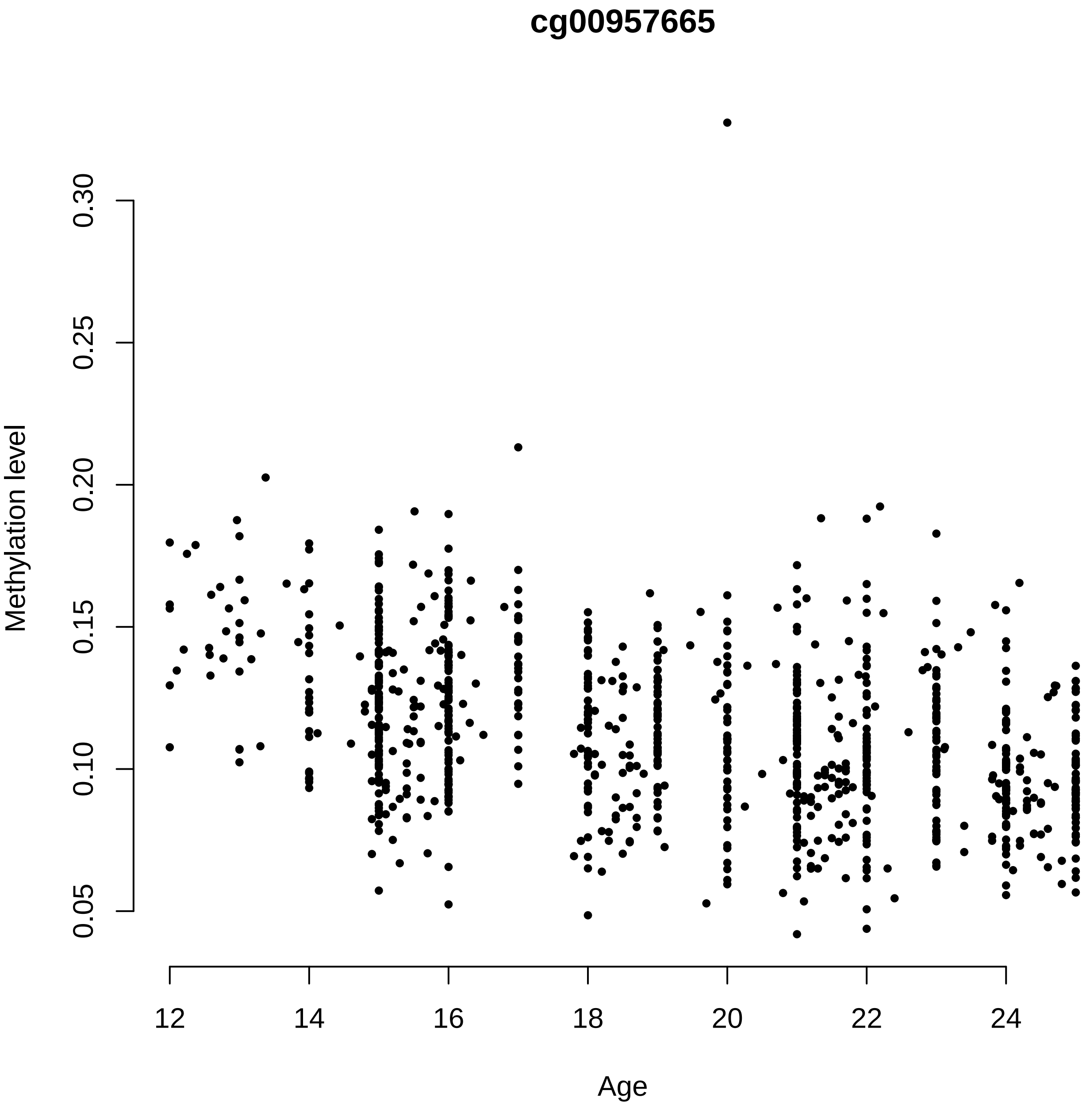

cg00957665
Methylation level
0.05
0.10
0.15
0.20
0.25
0.30
12
14
16
18
20
22
24
Age

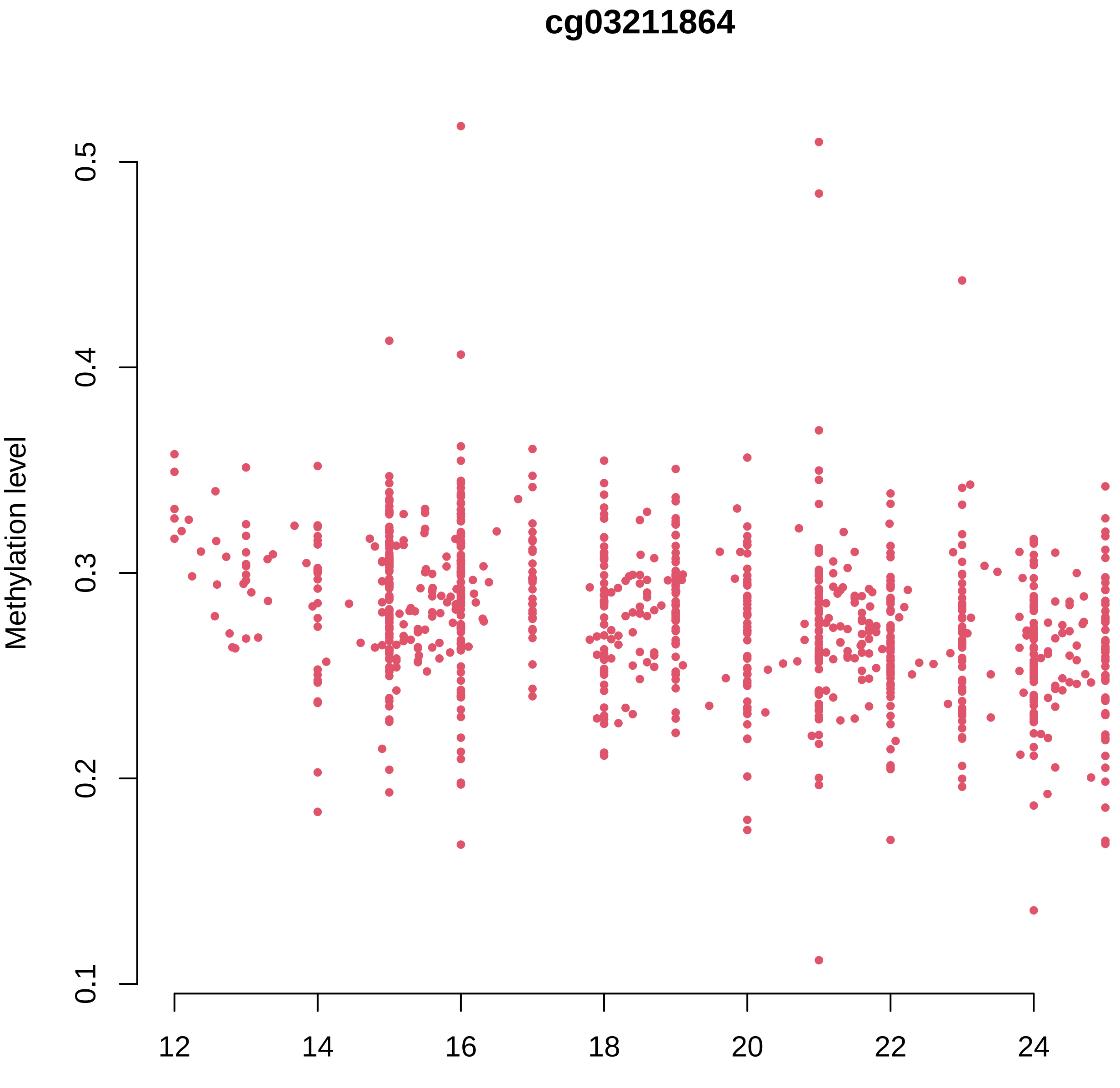
cg03211864
Methylation level
0.1
0.2
0.3
0.4
0.5
12
14
16
18
20
22
24
Age

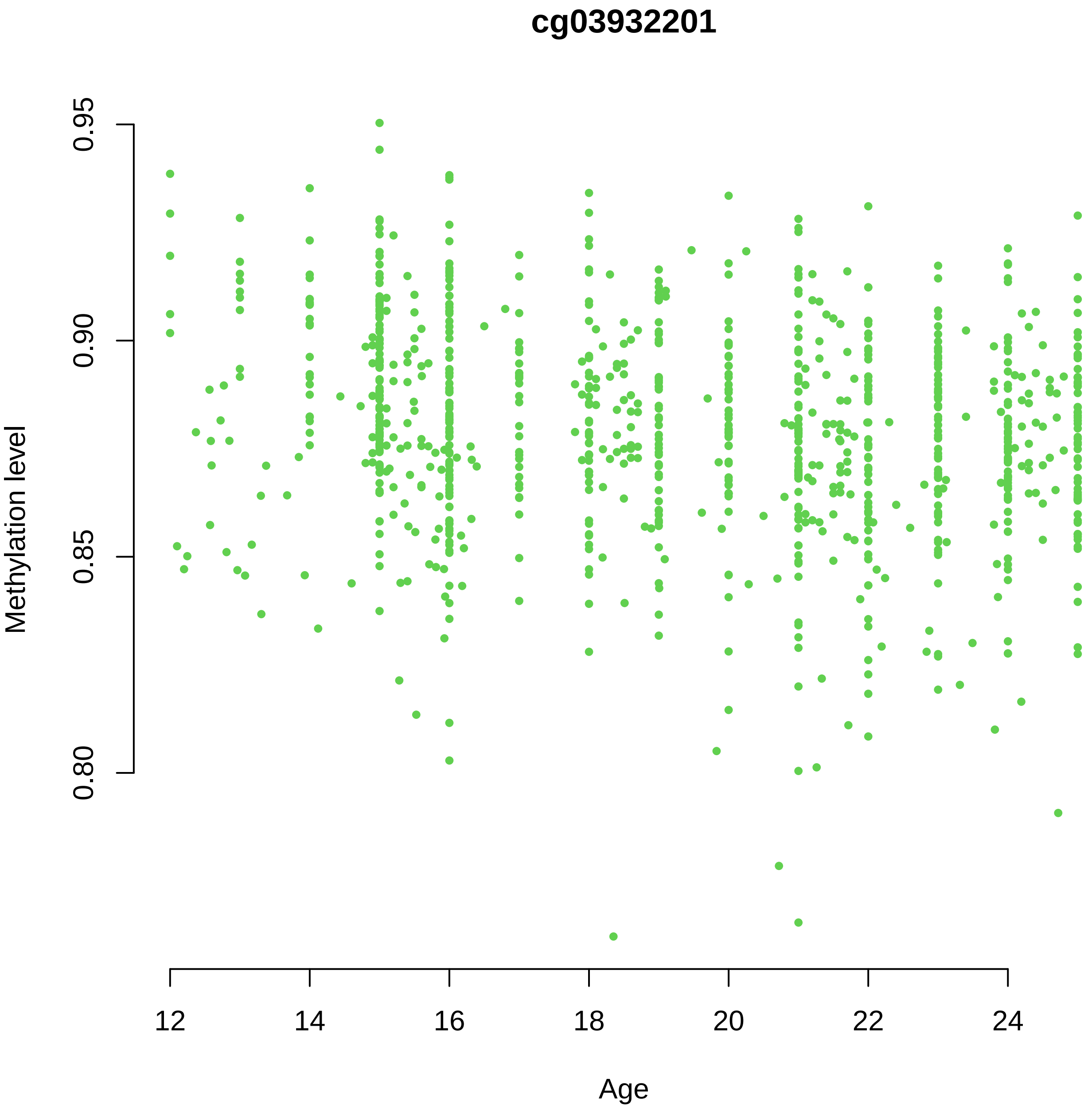
cg03932201
Methylation level
0.80
0.85
0.90
0.95
Age
12
14
16
18
20
22
24

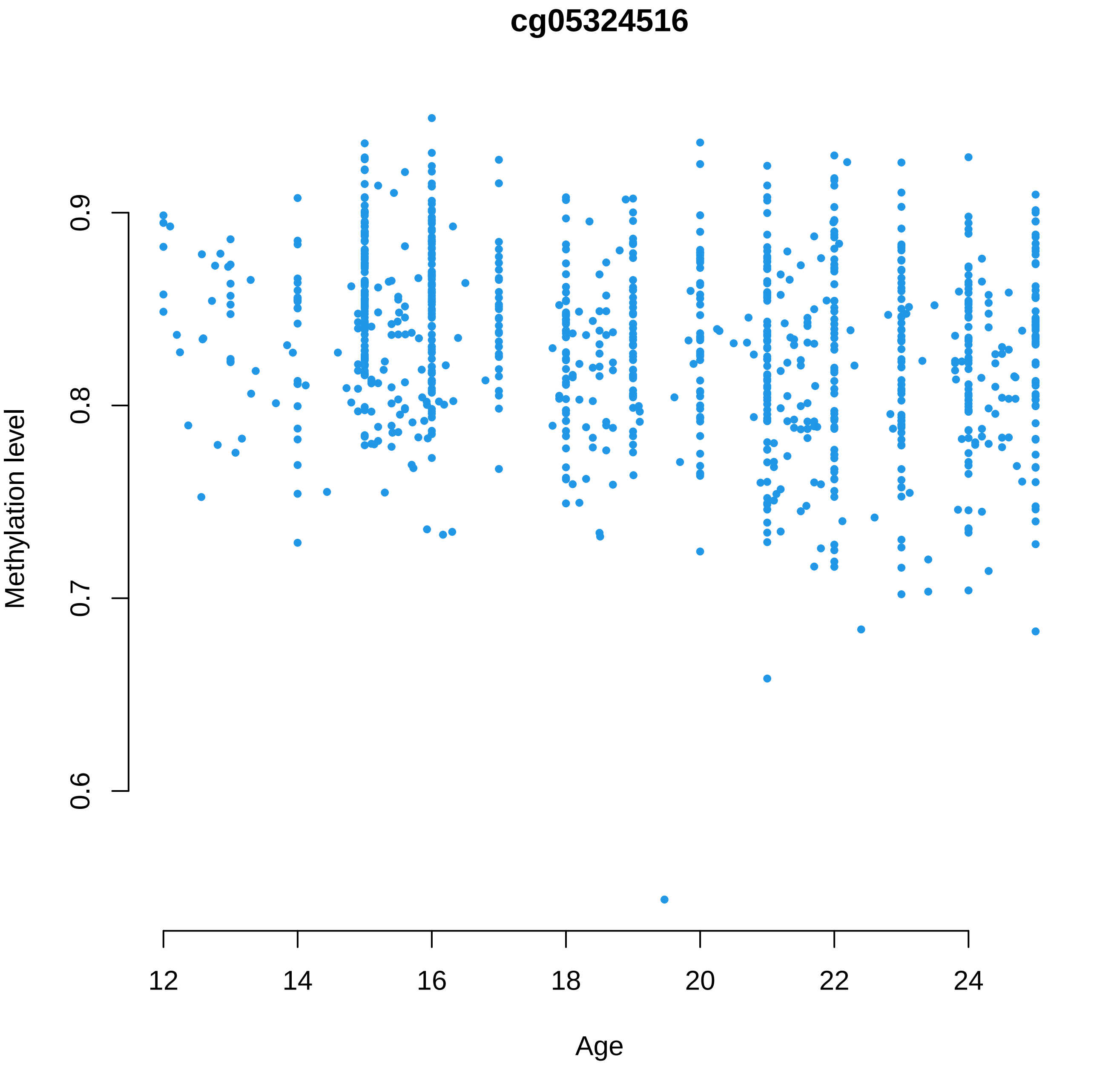
cg05324516
Methylation level
0.6
0.7
0.8
0.9
12
14
16
18
20
22
24
Age

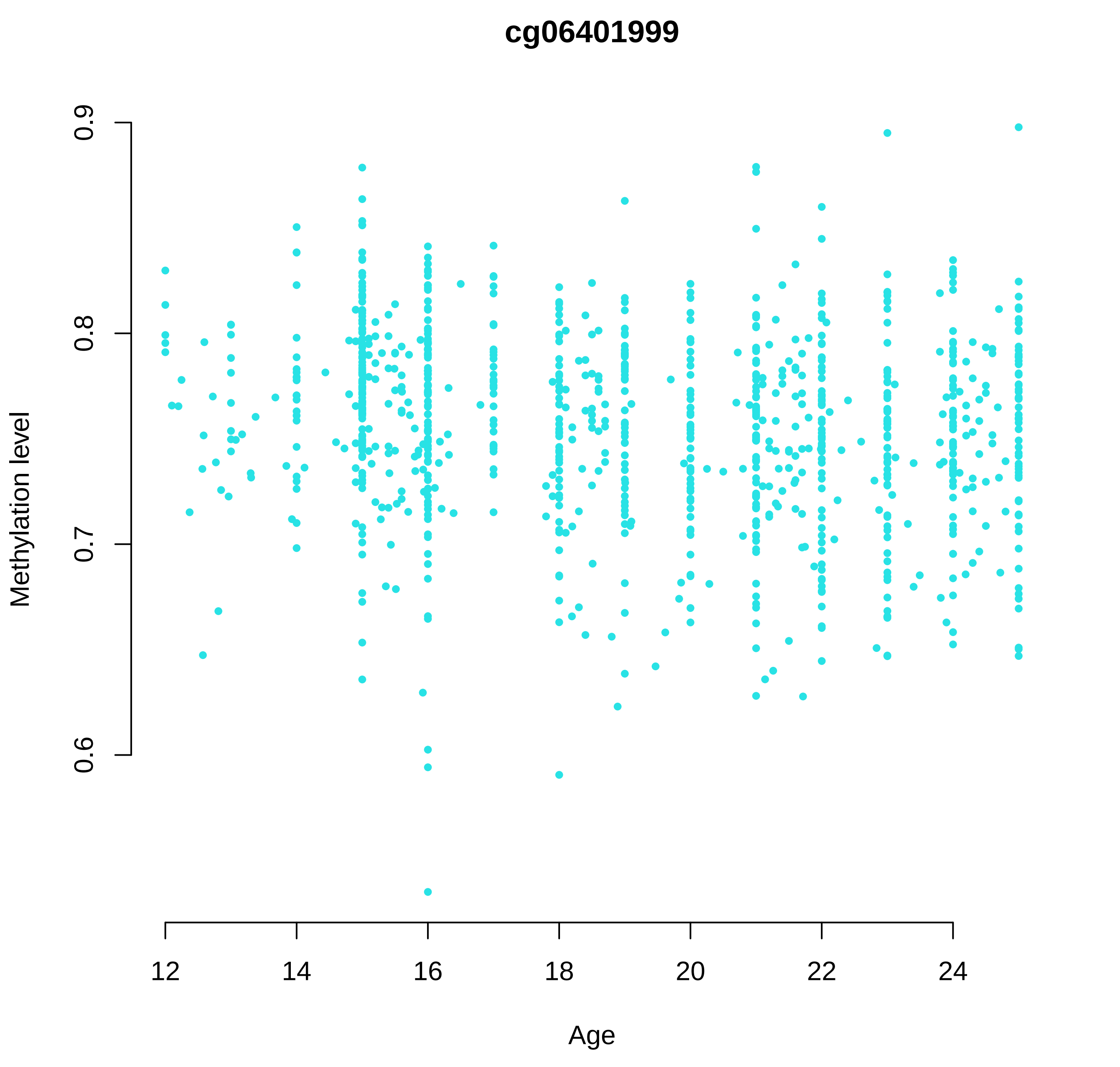
cg06401999
Methylation level
0.6
0.7
0.8
0.9
12
14
16
18
20
22
24
Age

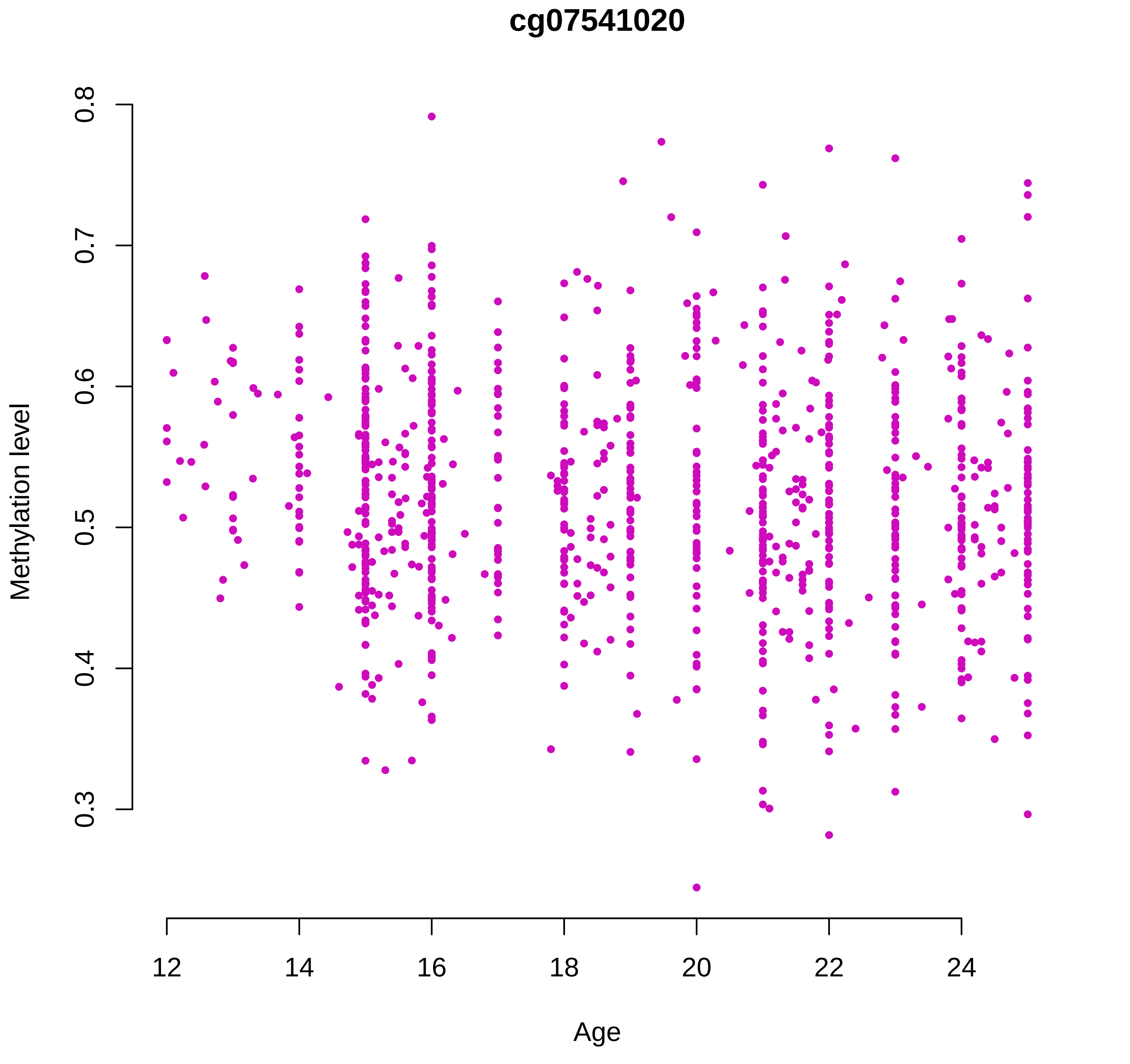
cg07541020
Methylation level
0.3
0.4
0.5
0.6
0.7
0.8
Age
12
14
16
18
20
22
24

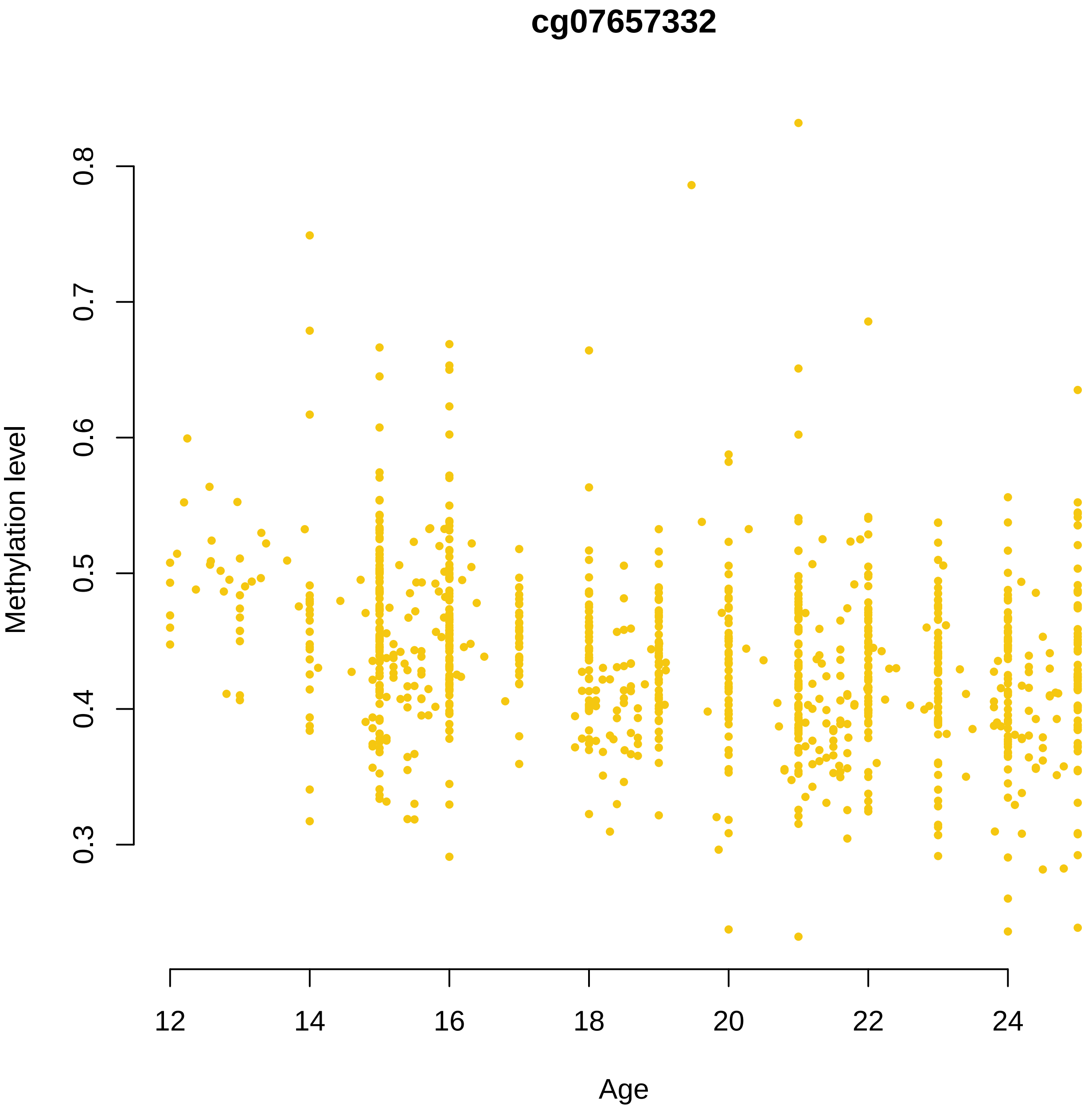
cg07657332
Methylation level
0.3
0.4
0.5
0.6
0.7
0.8
Age
12
14
16
18
20
22
24

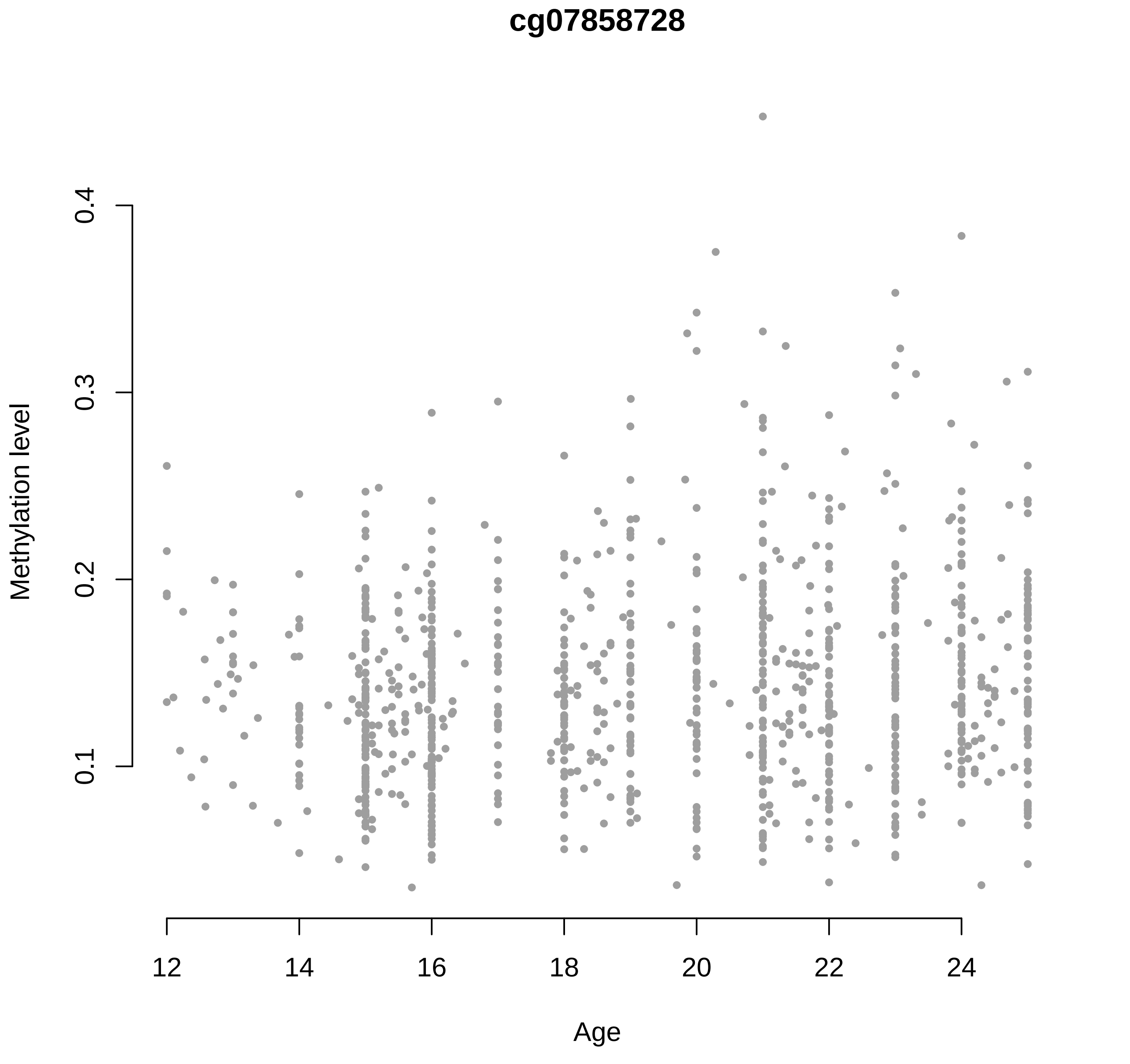
cg07858728
Methylation level
0.1
0.2
0.3
0.4
Age
12
14
16
18
20
22
24

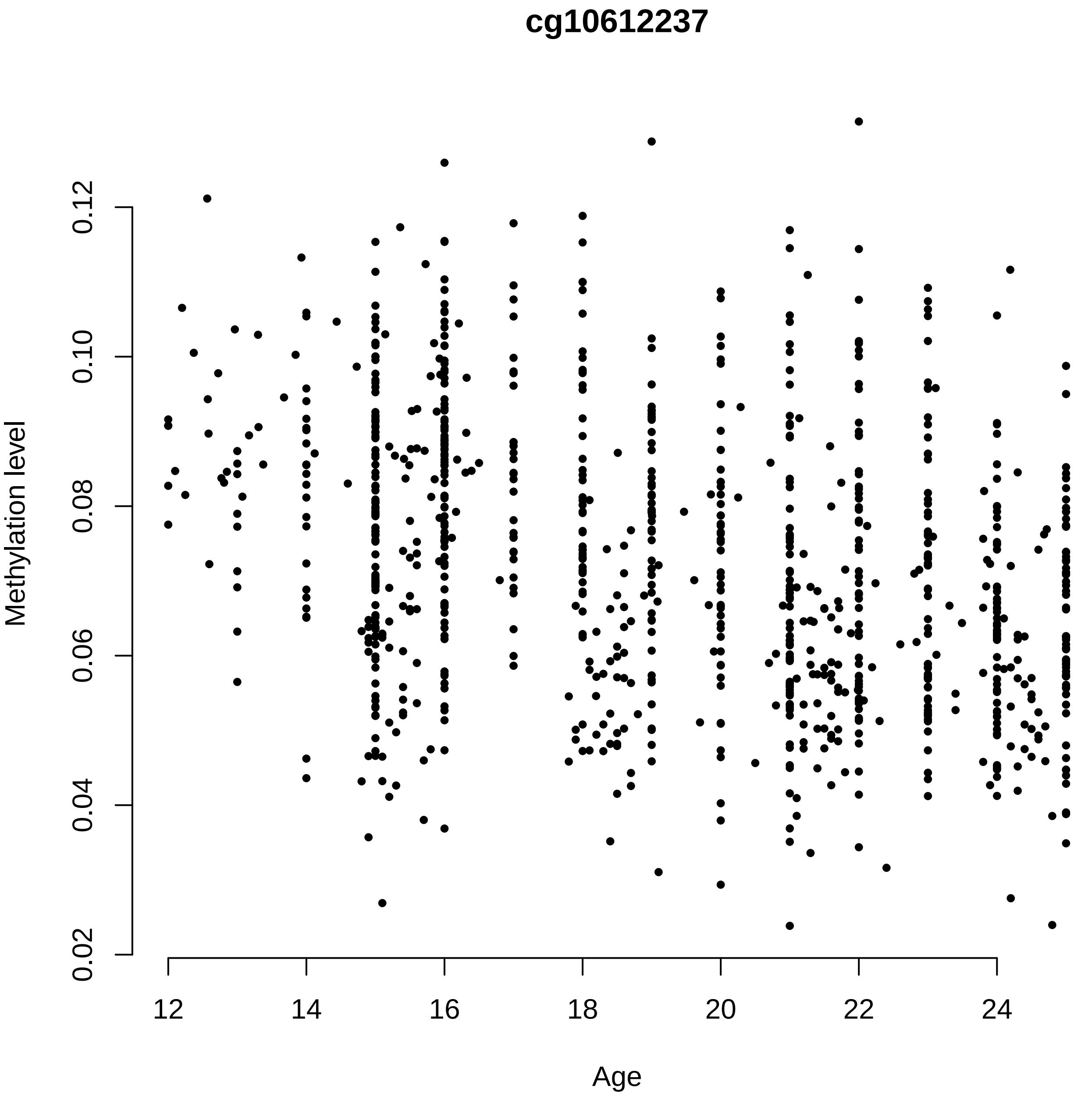
cg10612237
Methylation level
Age
0.02
0.04
0.06
0.08
0.10
0.12
12
14
16
18
20
22
24

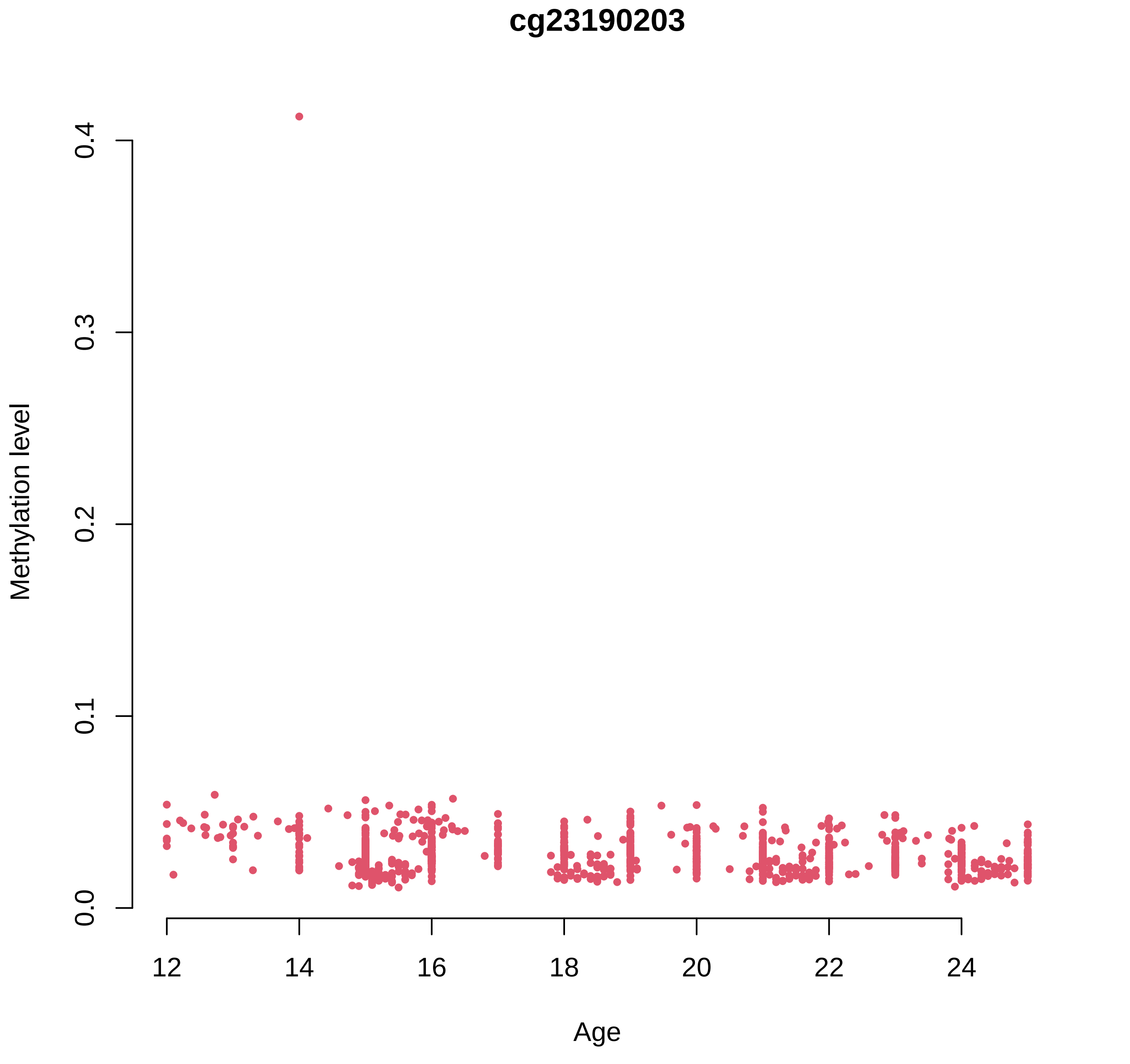
cg23190203
Methylation level
0.0
0.1
0.2
0.3
0.4
Age
12
14
16
18
20
22
24

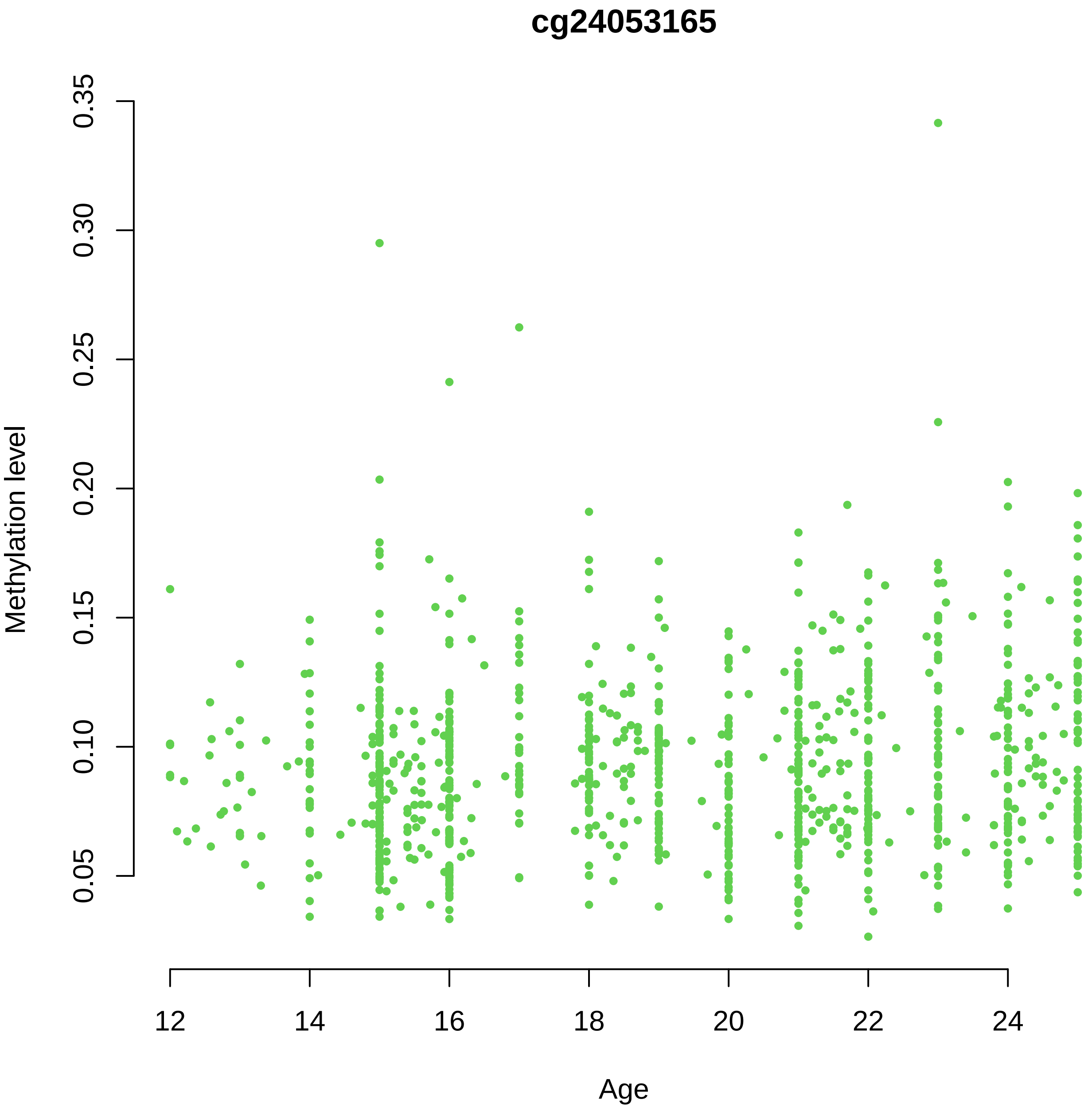

cg24053165
Methylation level
0.05
0.10
0.15
0.20
0.25
0.30
0.35
Age
12
14
16
18
20
22
24

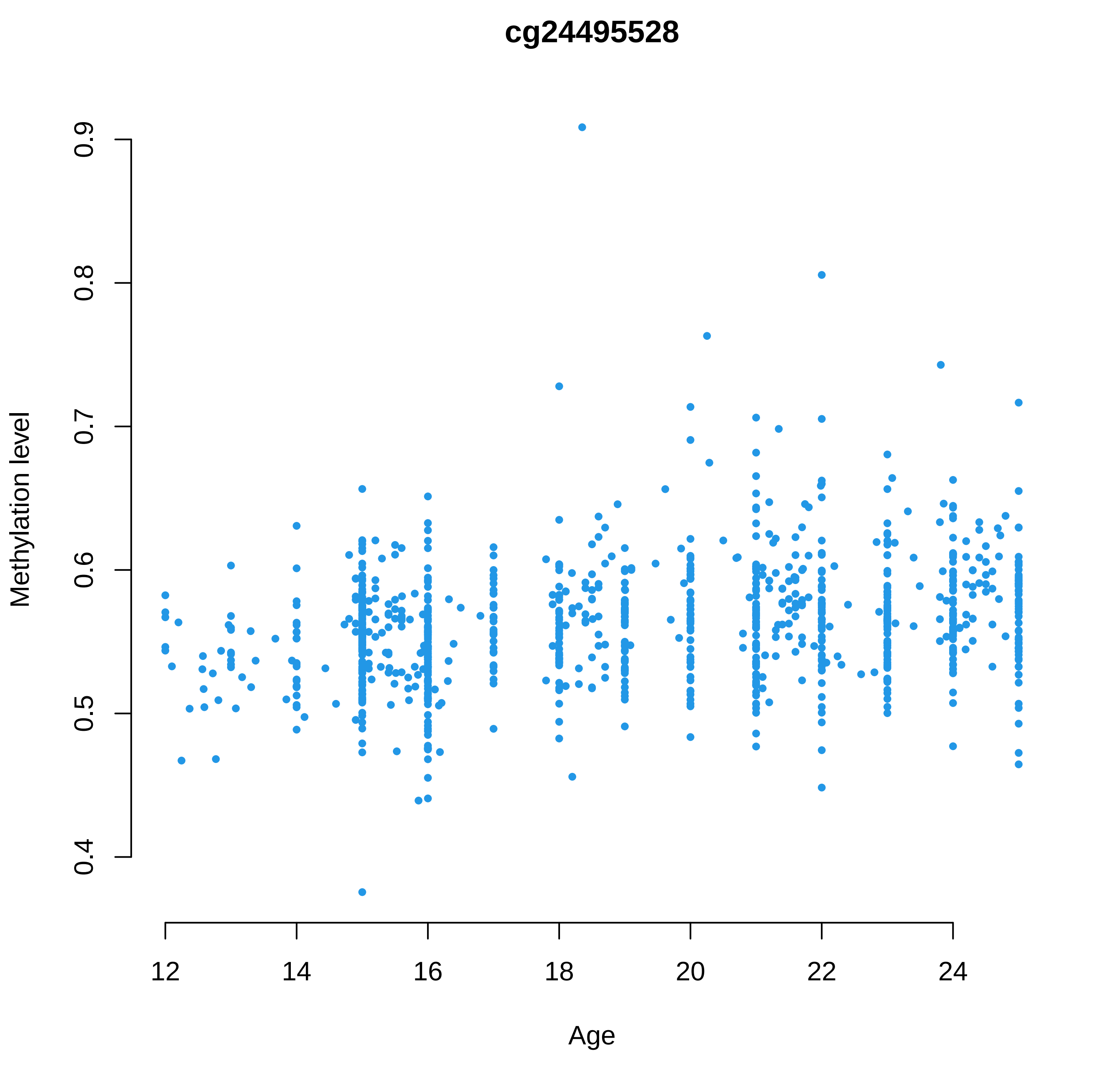
cg24495528
Methylation level
0.4
0.5
0.6
0.7
0.8
0.9
Age
12
14
16
18
20
22
24

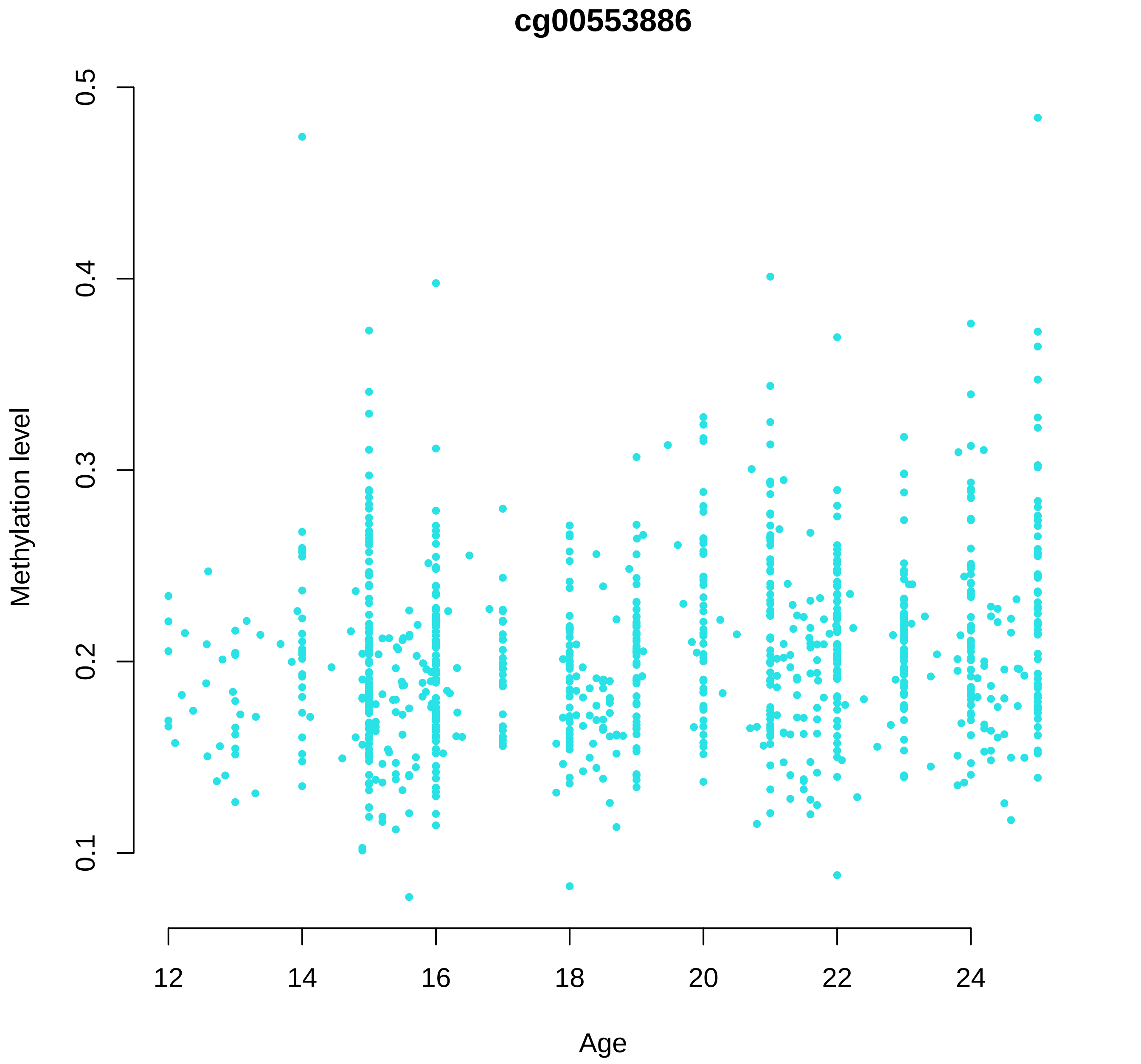
cg00553886
Methylation level
0.1
0.2
0.3
0.4
0.5
12
14
16
18
20
22
24
Age

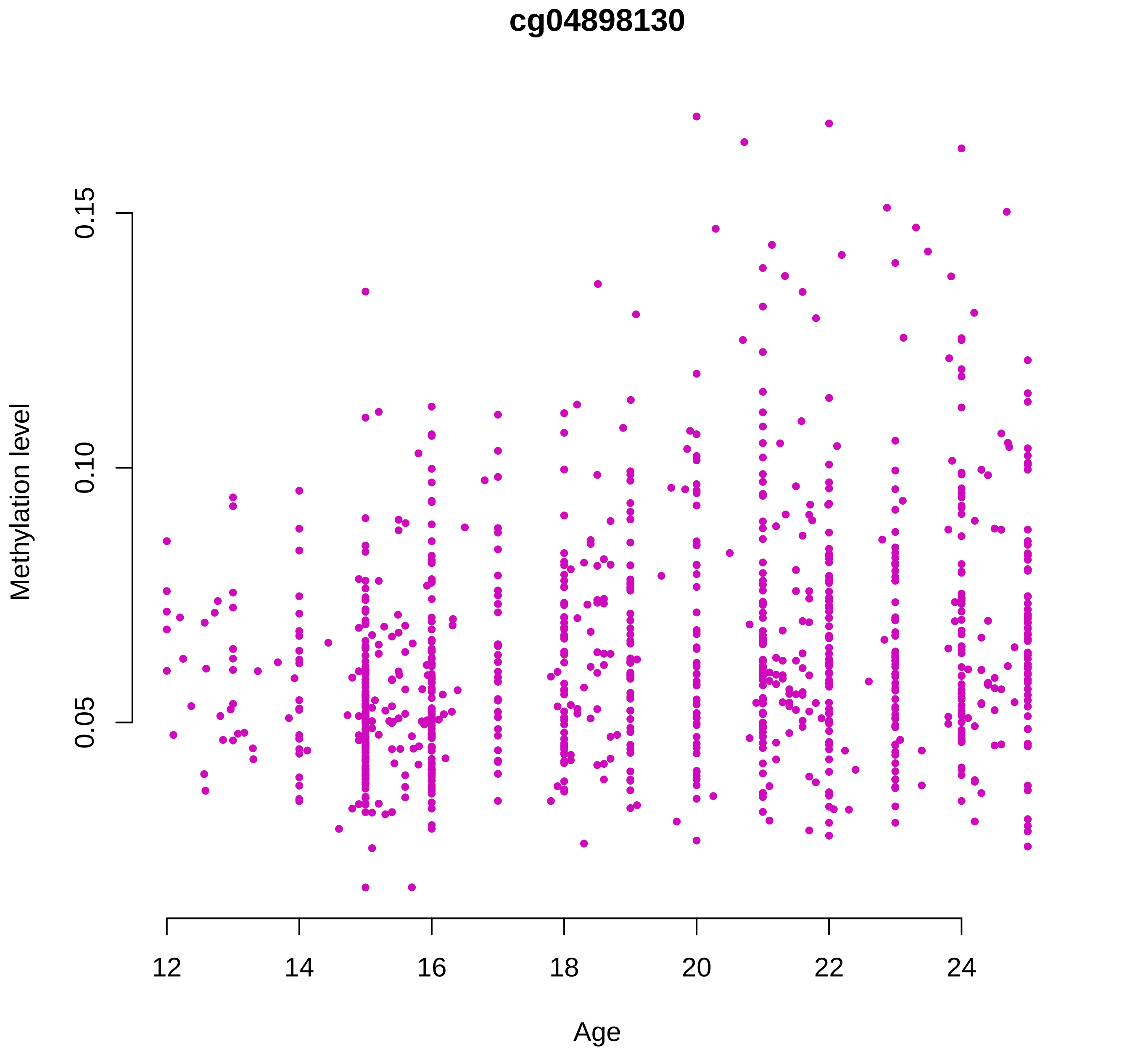
cg04898130
Methylation level
0.05
0.10
0.15
12
14
16
18
20
22
24
Age

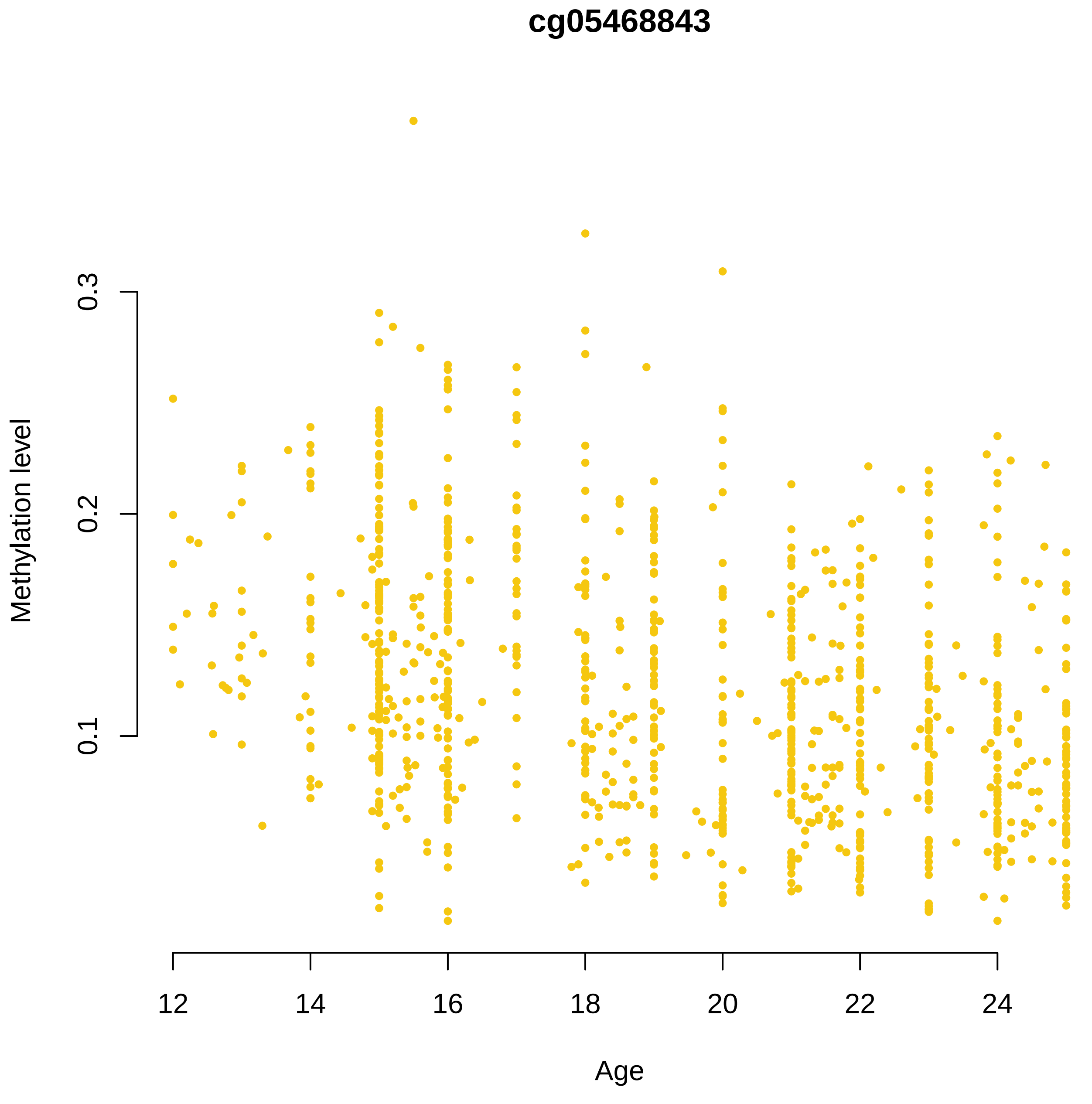
cg05468843
Methylation level
0.1
0.2
0.3
Age
12
14
16
18
20
22
24

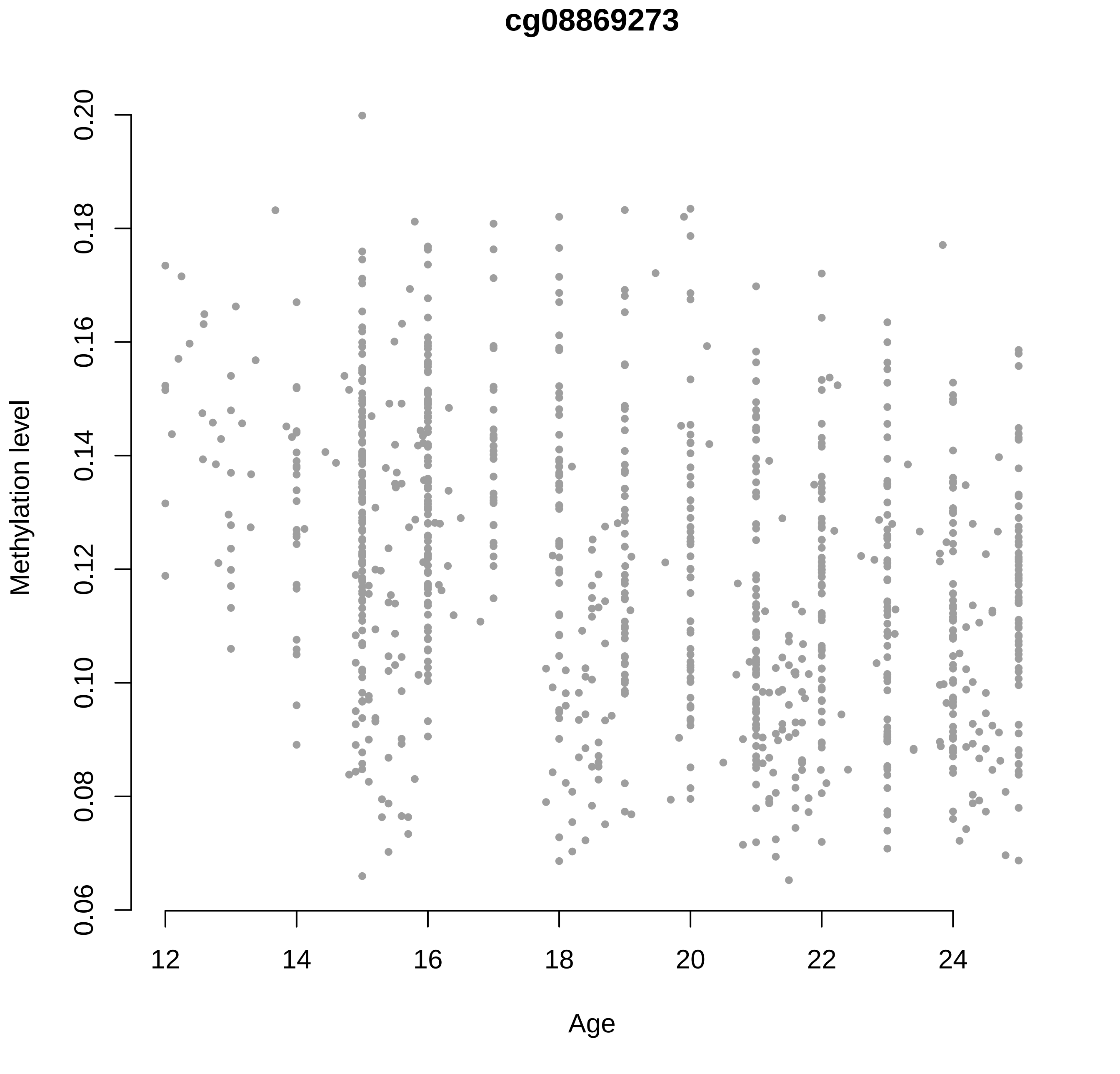

cg08869273
Methylation level
0.06
0.08
0.10
0.12
0.14
0.16
0.18
0.20
12
14
16
18
20
22
24
Age

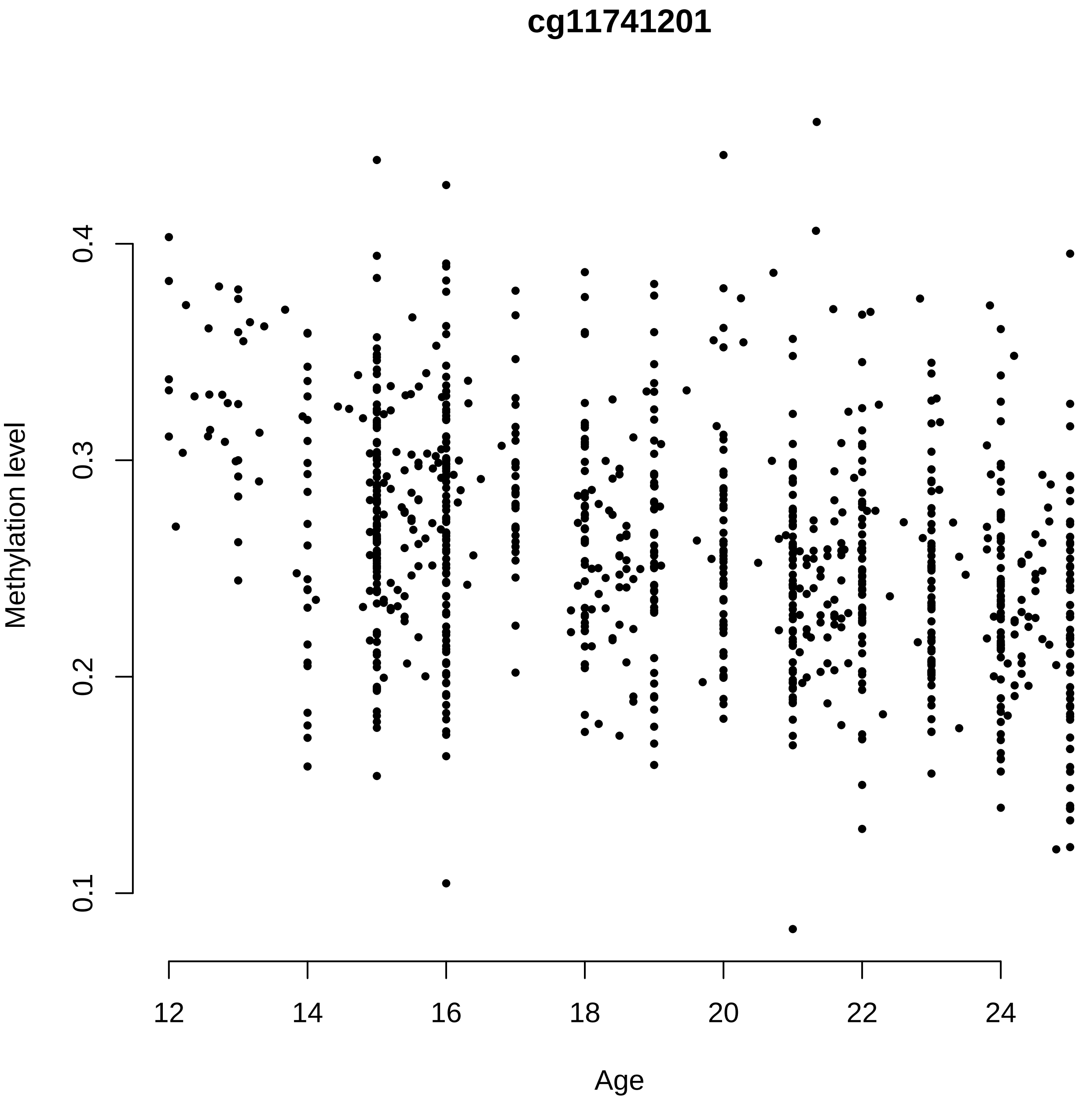
cg11741201
Methylation level
0.1
0.2
0.3
0.4
Age
12
14
16
18
20
22
24

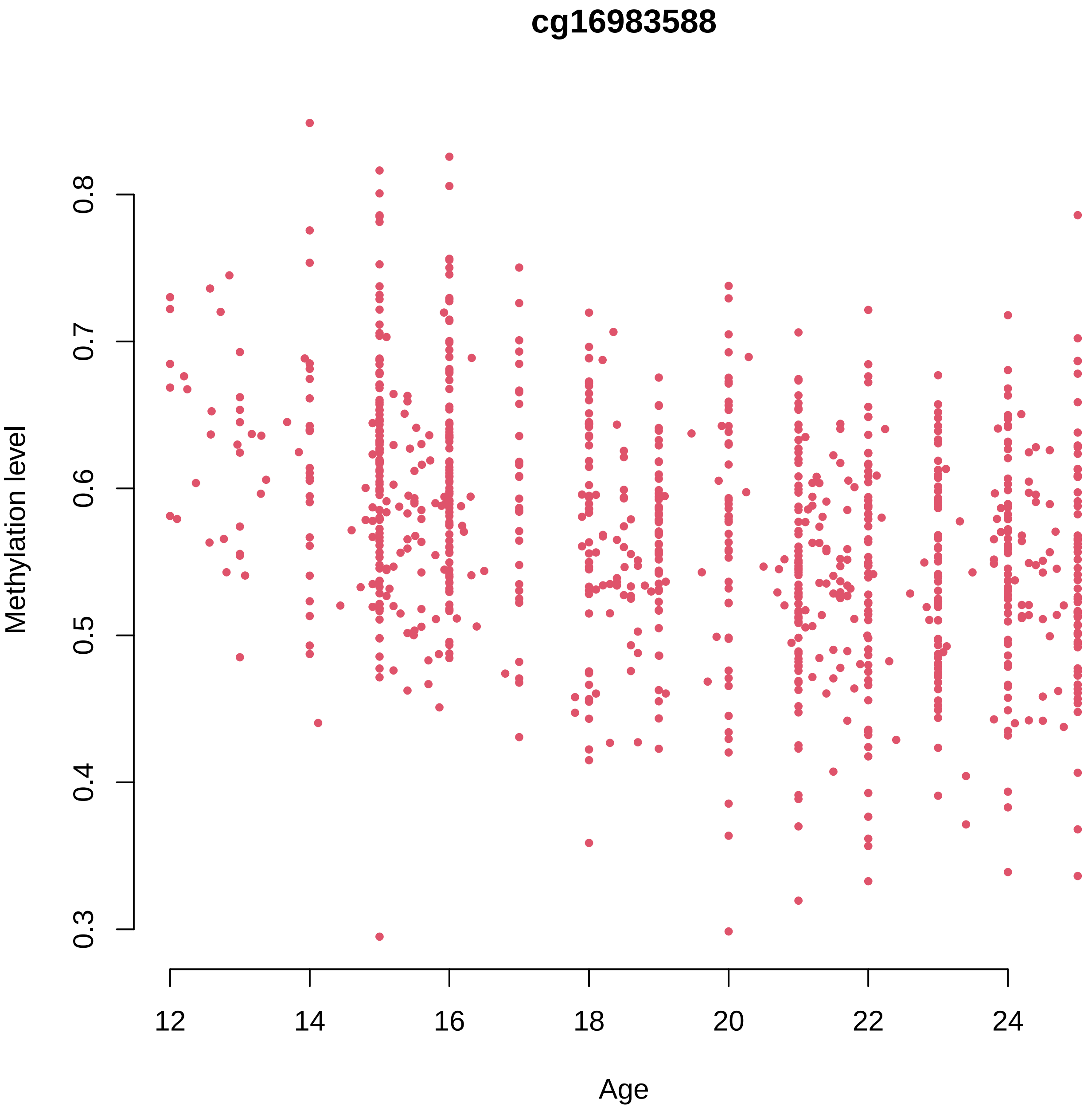
cg16983588
Methylation level
0.3
0.4
0.5
0.6
0.7
0.8
Age
12
14
16
18
20
22
24

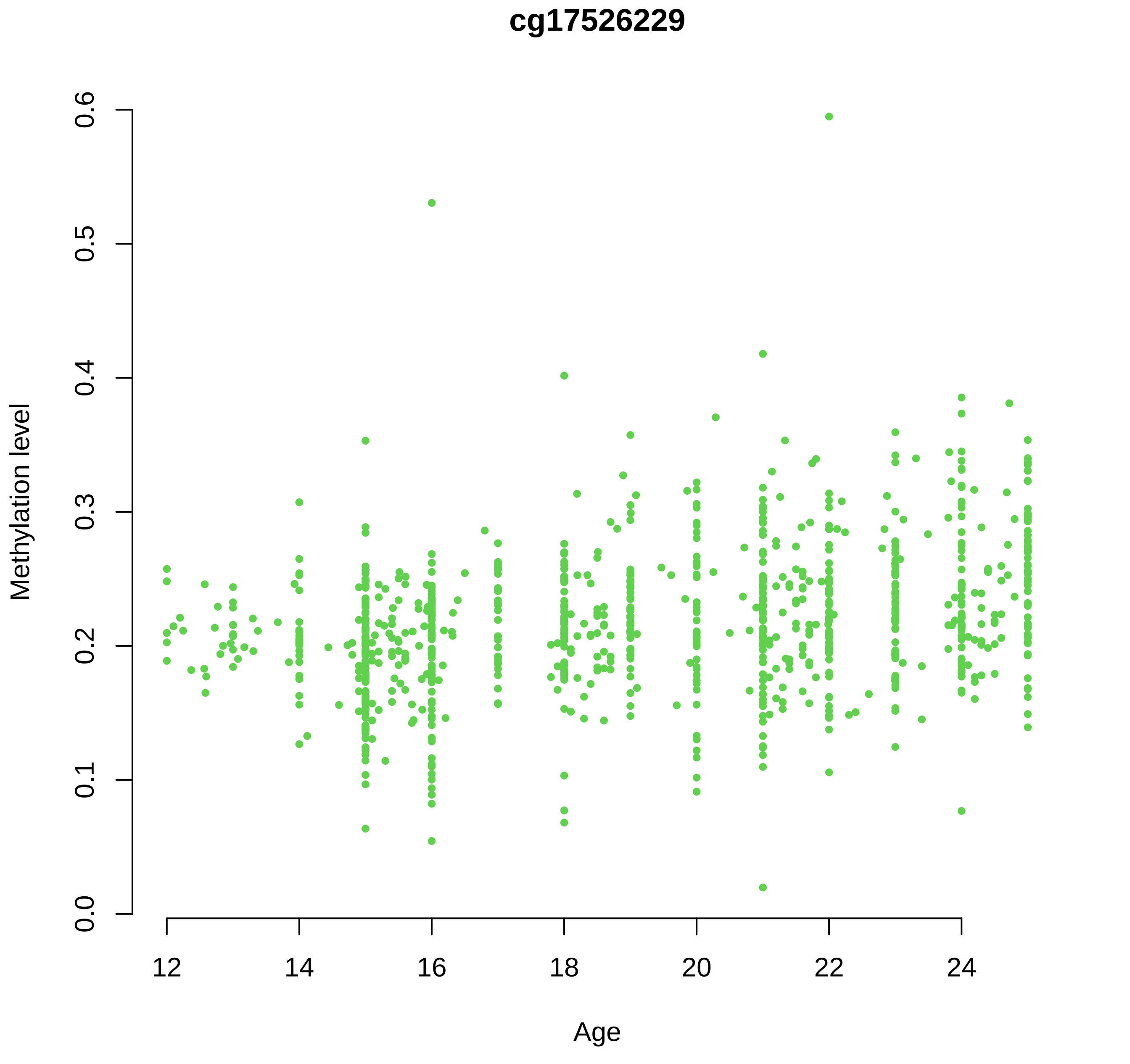

cg17526229
Methylation level
0.0
0.1
0.2
0.3
0.4
0.5
0.6
Age
12
14
16
18
20
22
24

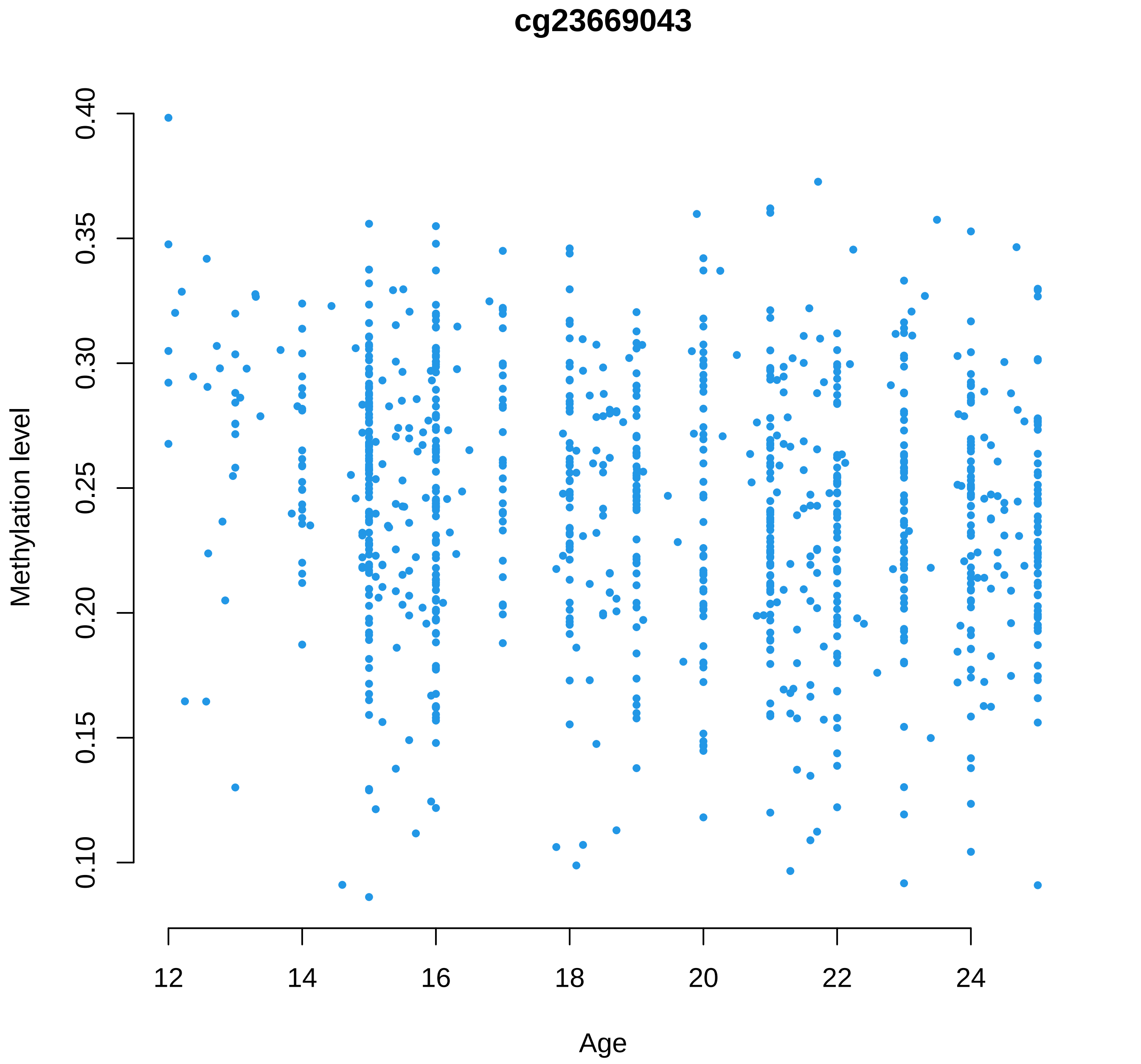
cg23669043
Methylation level
0.10
0.15
0.20
0.25
0.30
0.35
0.40
Age
12
14
16
18
20
22
24

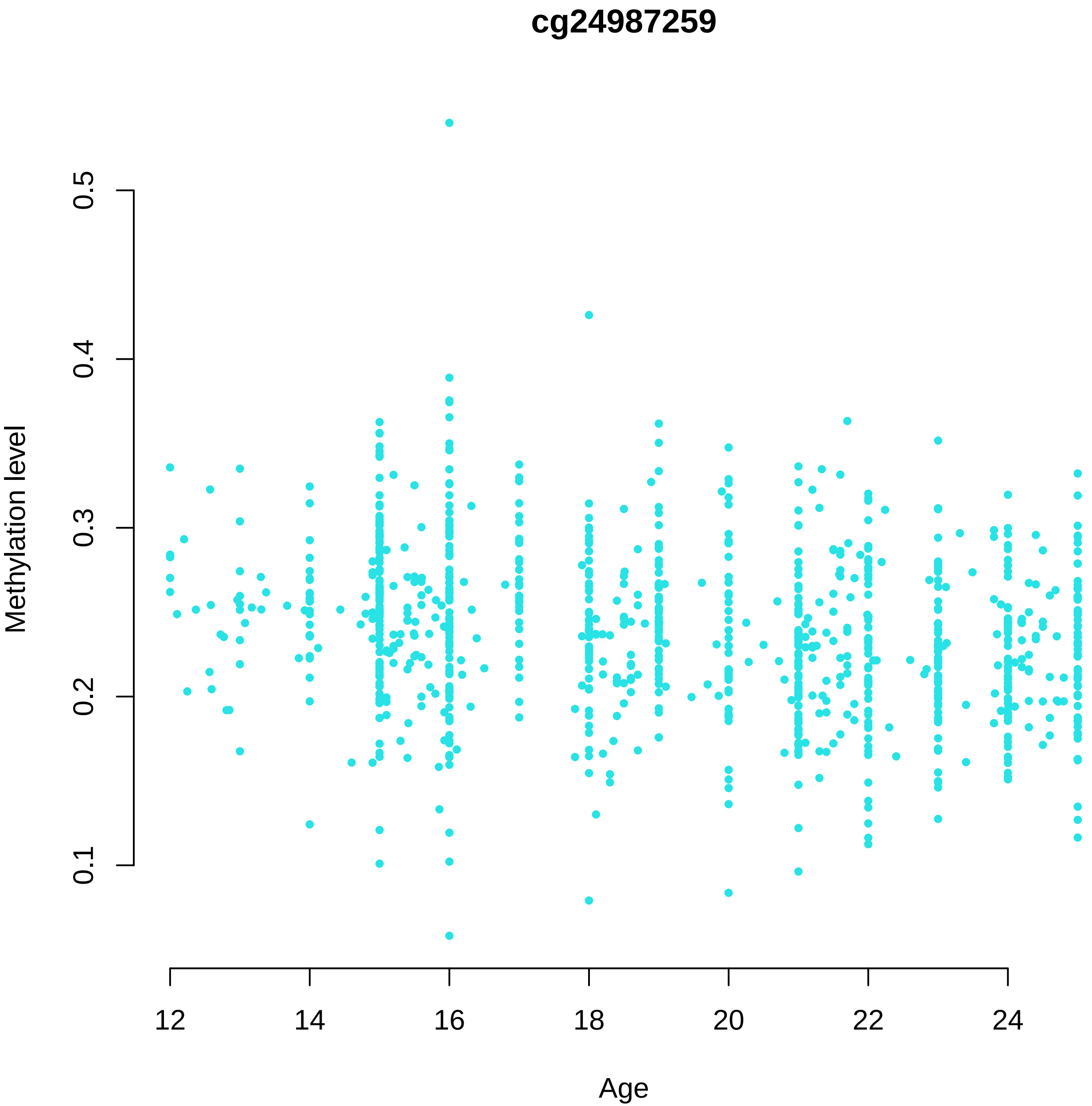

cg24987259
Methylation level
0.1
0.2
0.3
0.4
0.5
12
14
16
18
20
22
24
Age

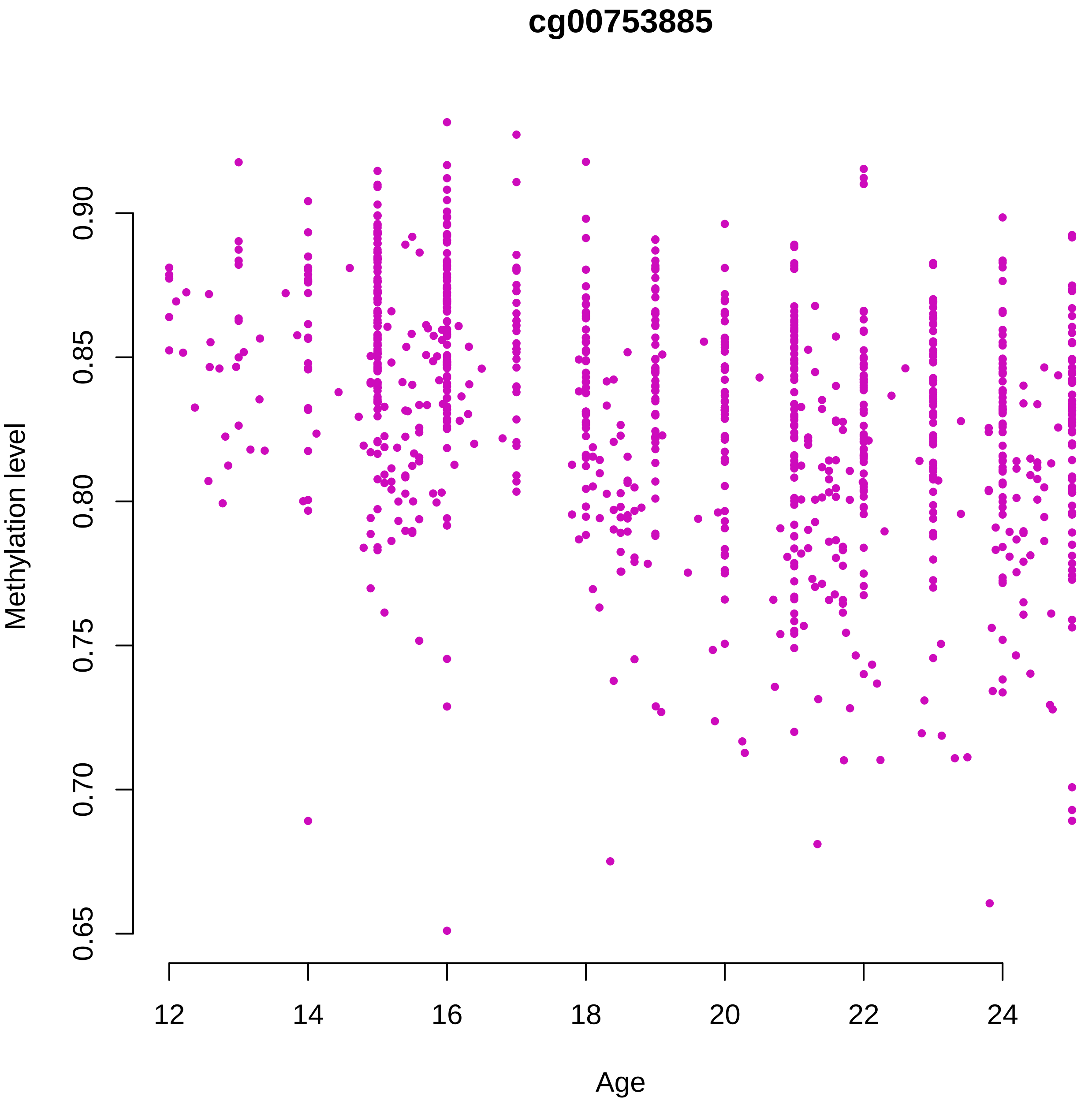

cg00753885
Methylation level
0.65
0.70
0.75
0.80
0.85
0.90
Age
12
14
16
18
20
22
24

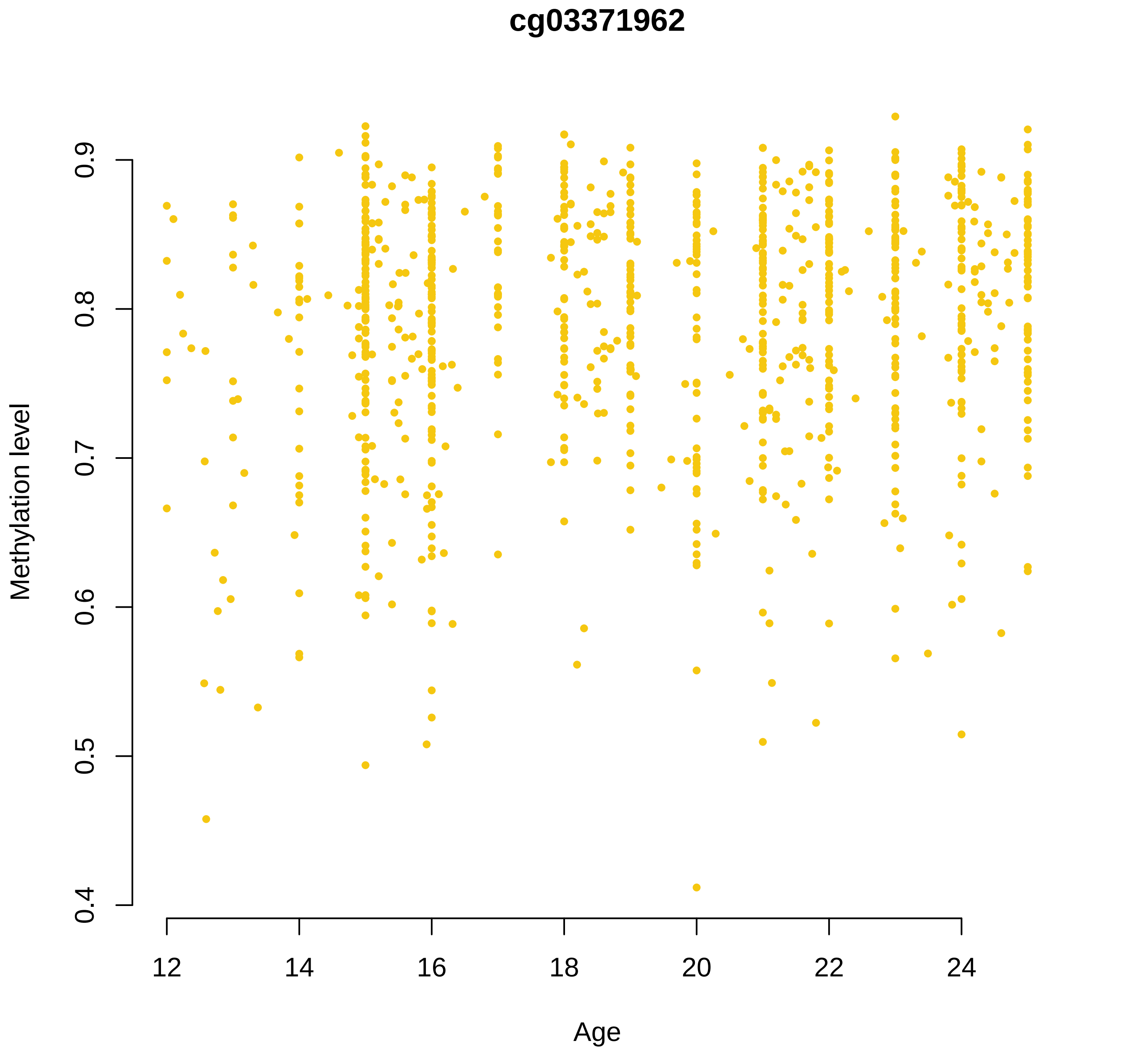
cg03371962
Methylation level
0.4
0.5
0.6
0.7
0.8
0.9
Age
12
14
16
18
20
22
24

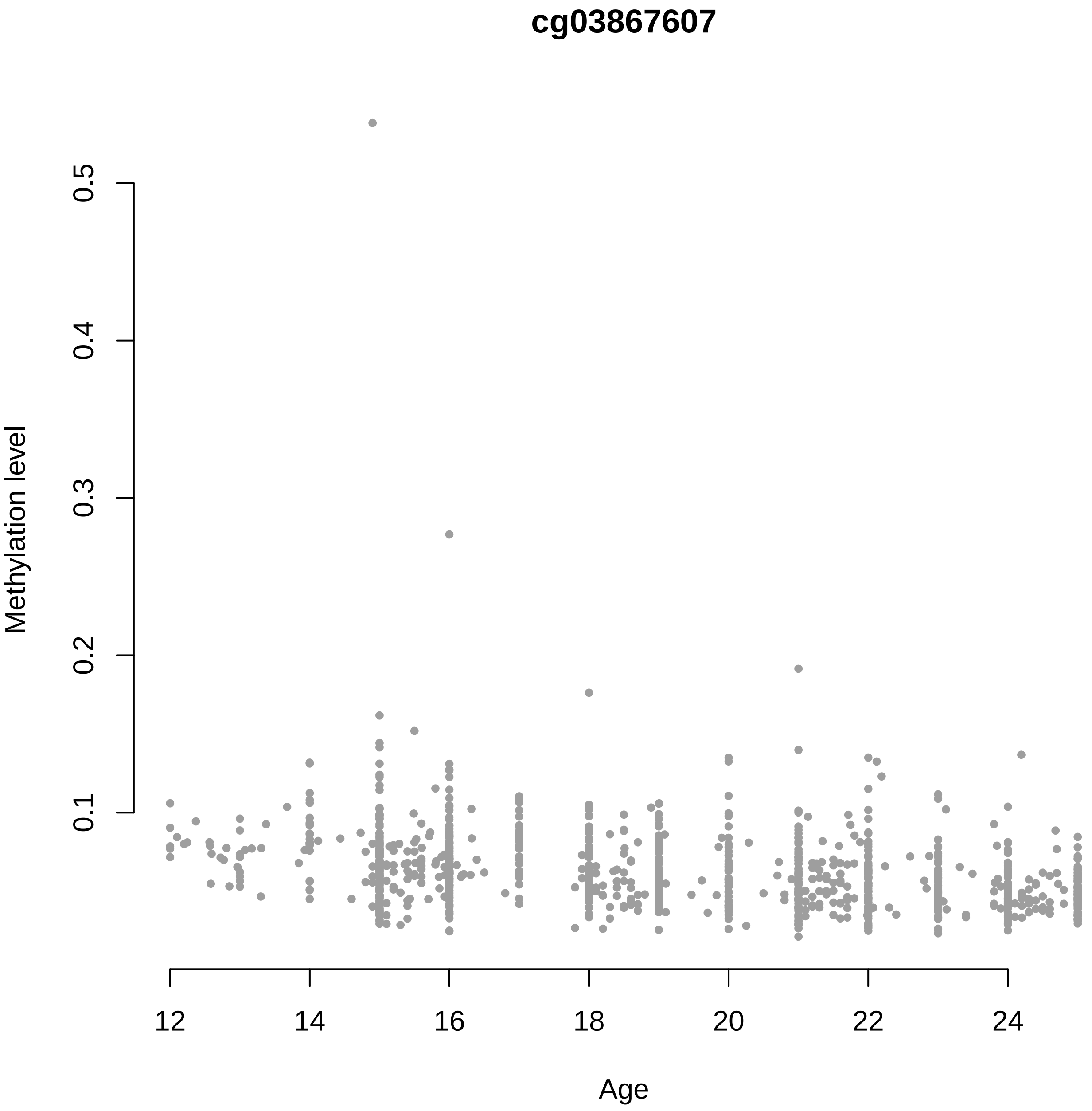
cg03867607
Methylation level
0.1
0.2
0.3
0.4
0.5
Age
12
14
16
18
20
22
24

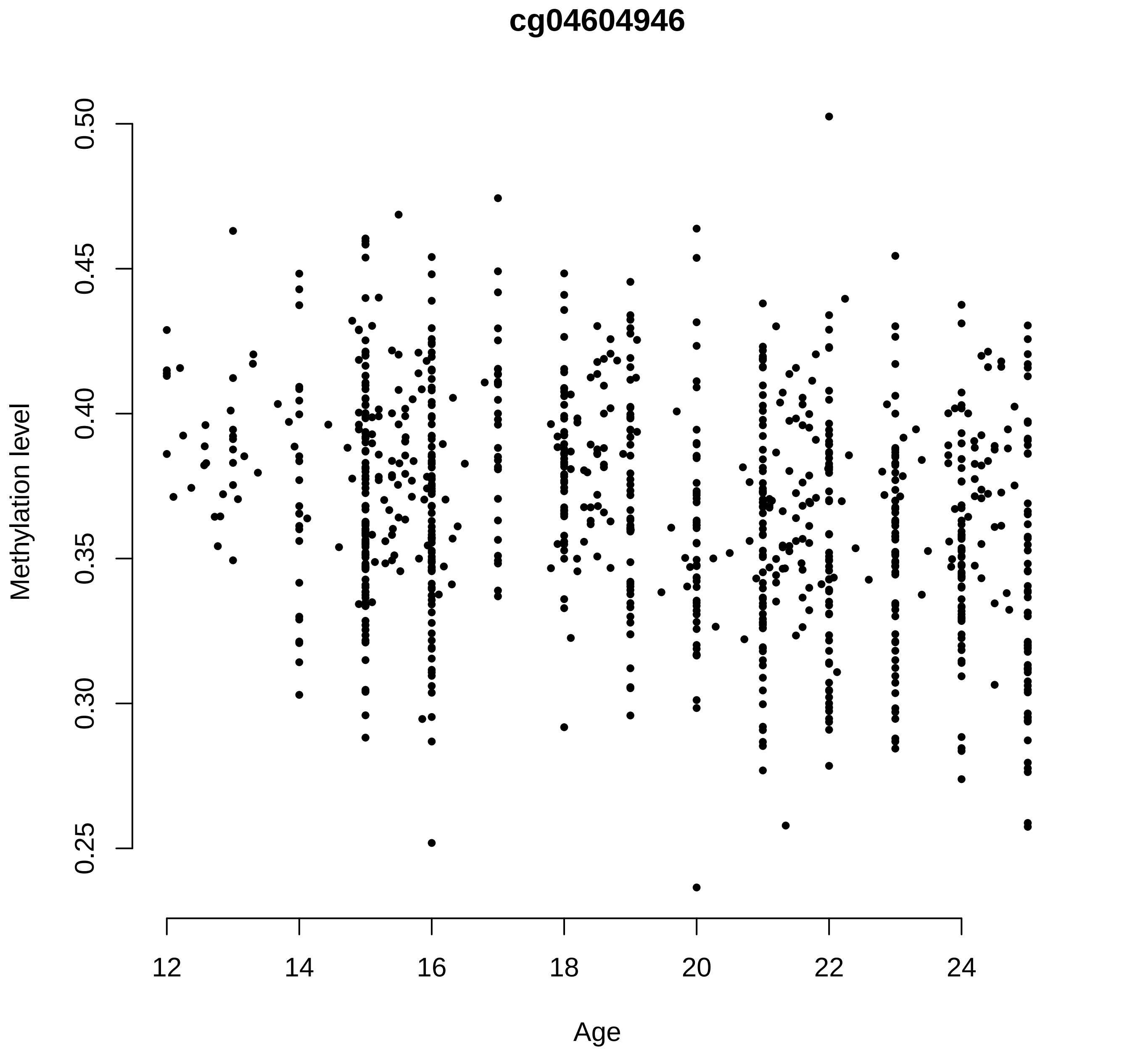
cg04604946
Methylation level
0.50
0.45
0.40
0.35
0.30
0.25
Age
12
14
16
18
20
22
24

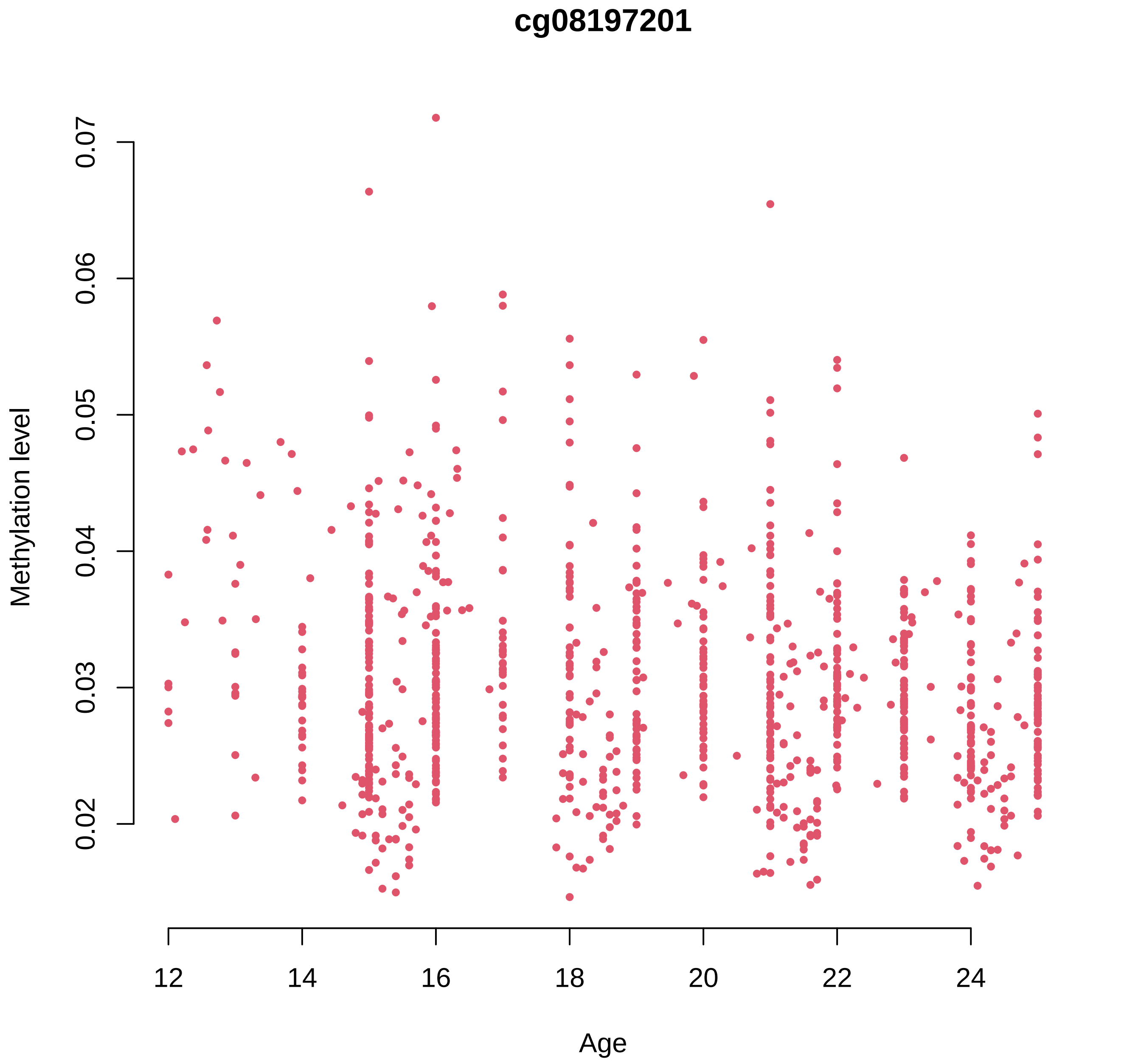
cg08197201
Methylation level
0.02
0.03
0.04
0.05
0.06
0.07
Age
12
14
16
18
20
22
24

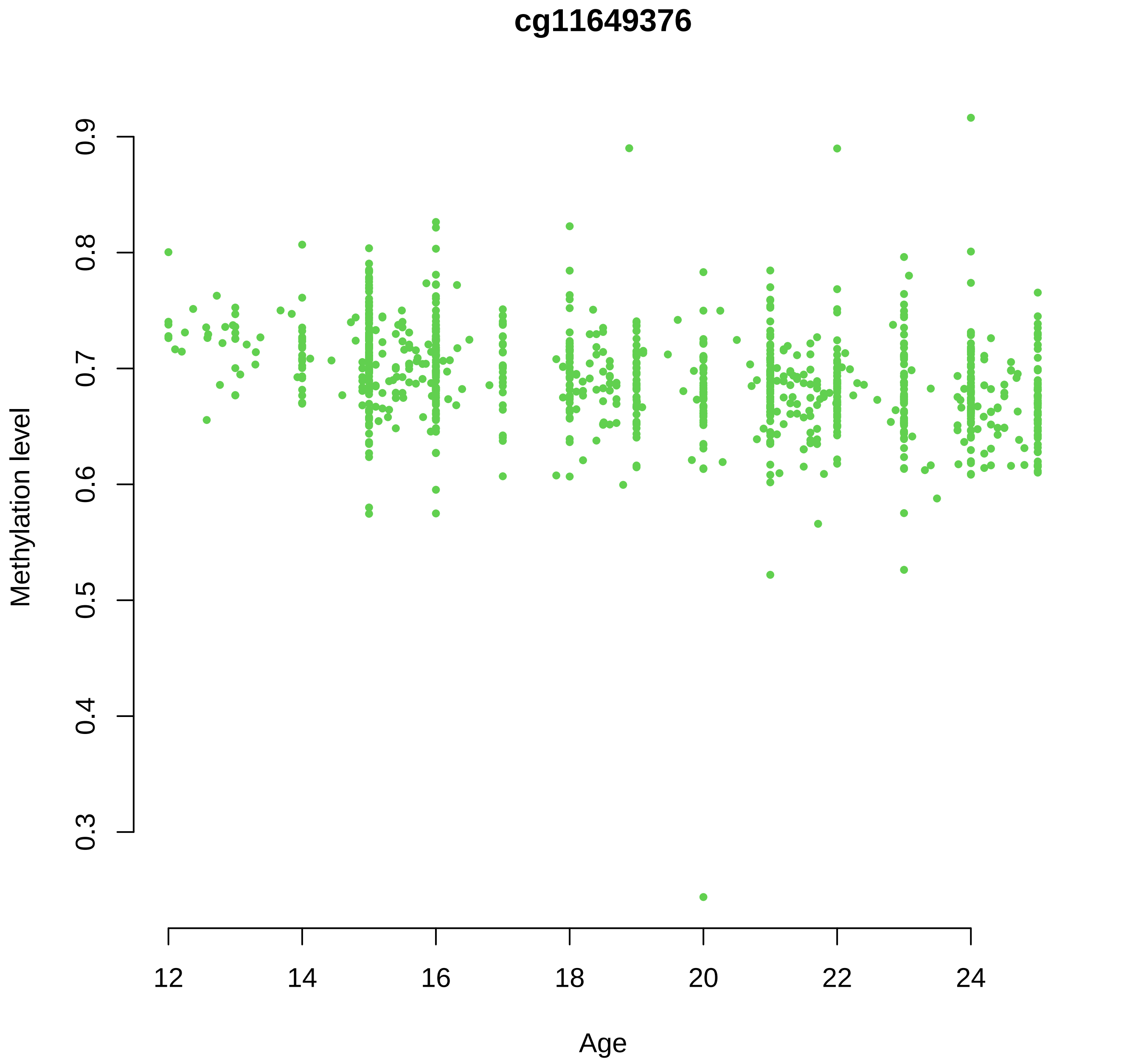
cg11649376
Methylation level
0.3
0.4
0.5
0.6
0.7
0.8
0.9
Age
12
14
16
18
20
22
24

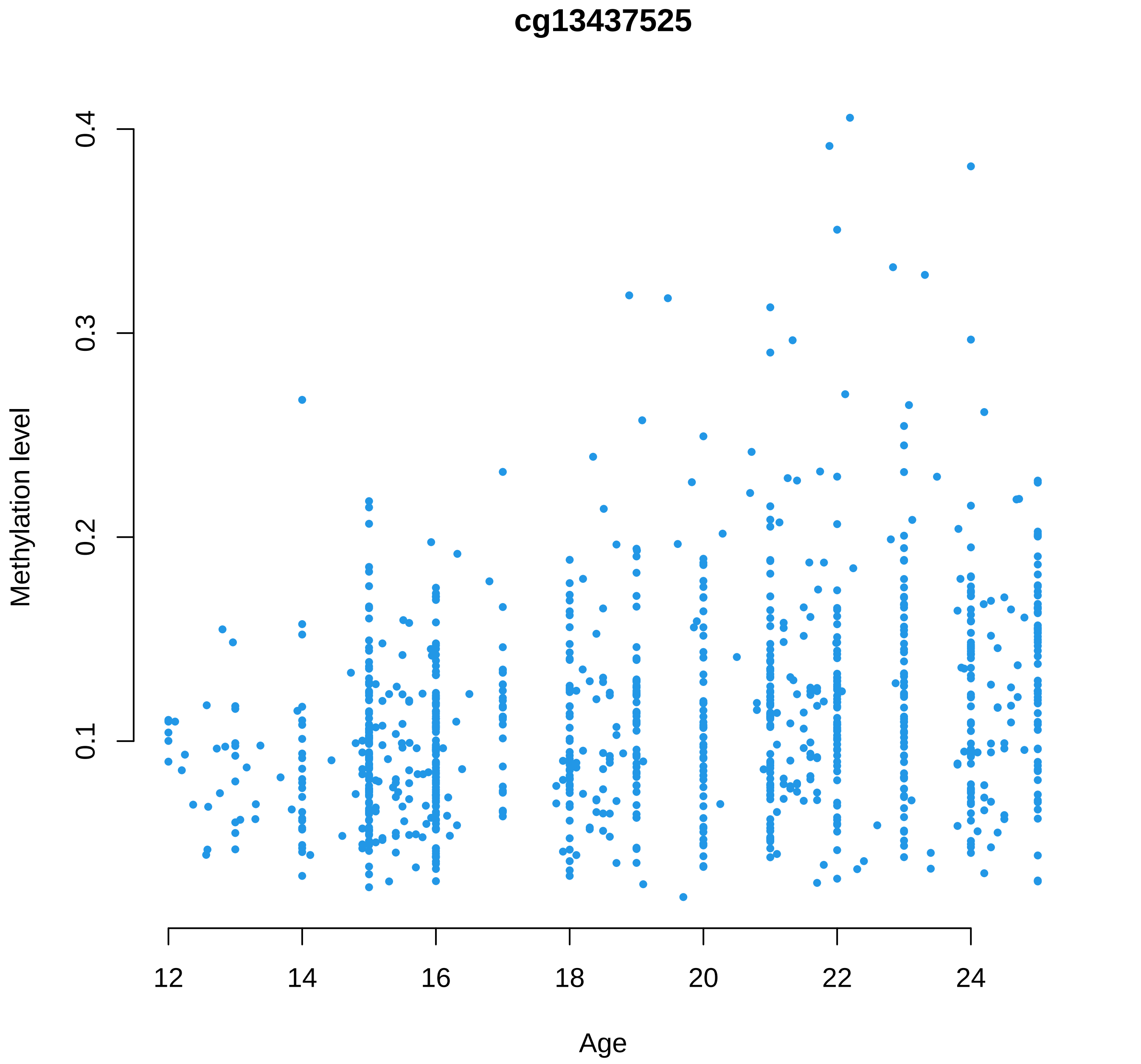
cg13437525
Methylation level
0.1
0.2
0.3
0.4
12
14
16
18
20
22
24
Age

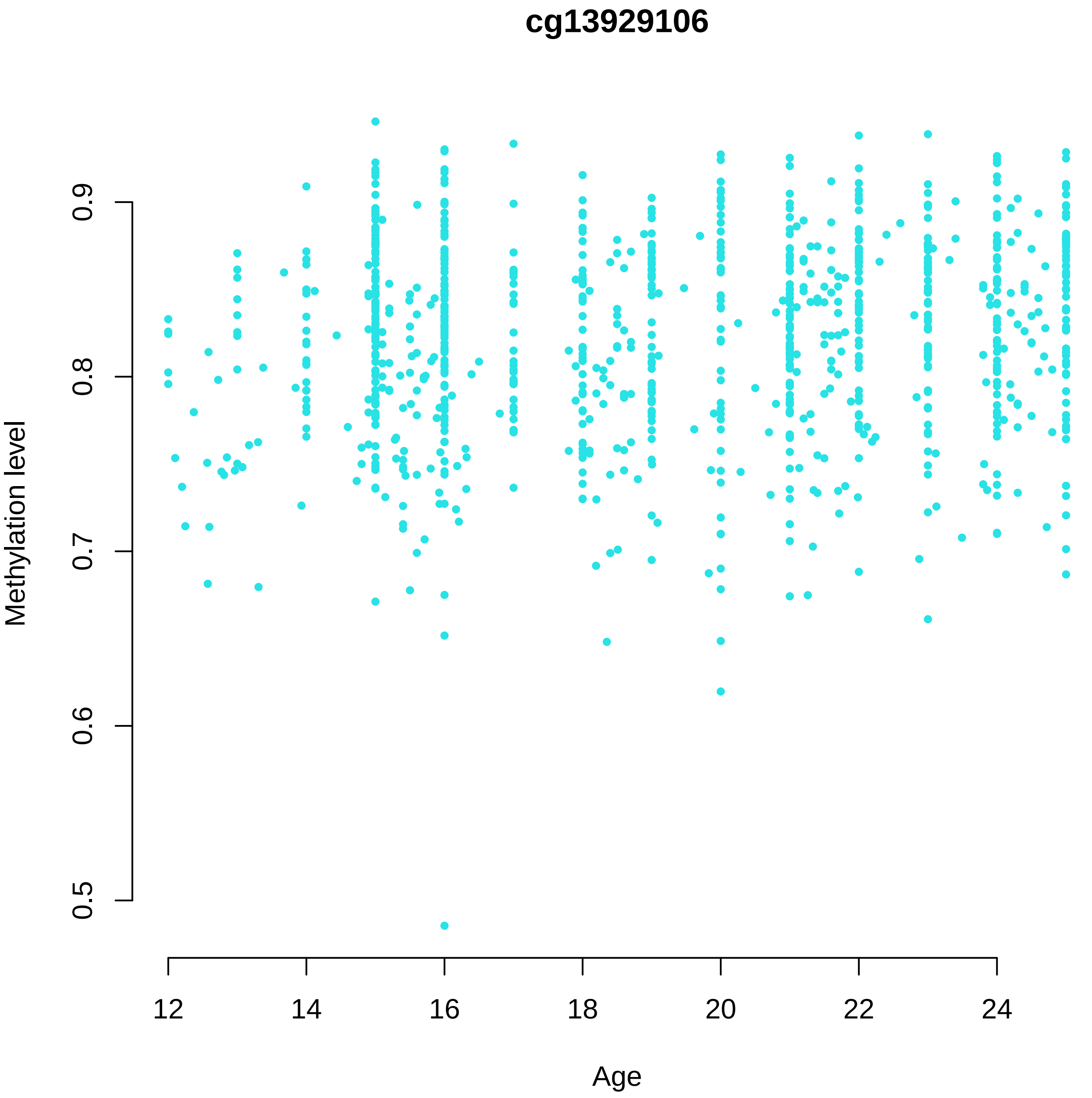

cg13929106
Methylation level
0.5
0.6
0.7
0.8
0.9
12
14
16
18
20
22
24
Age

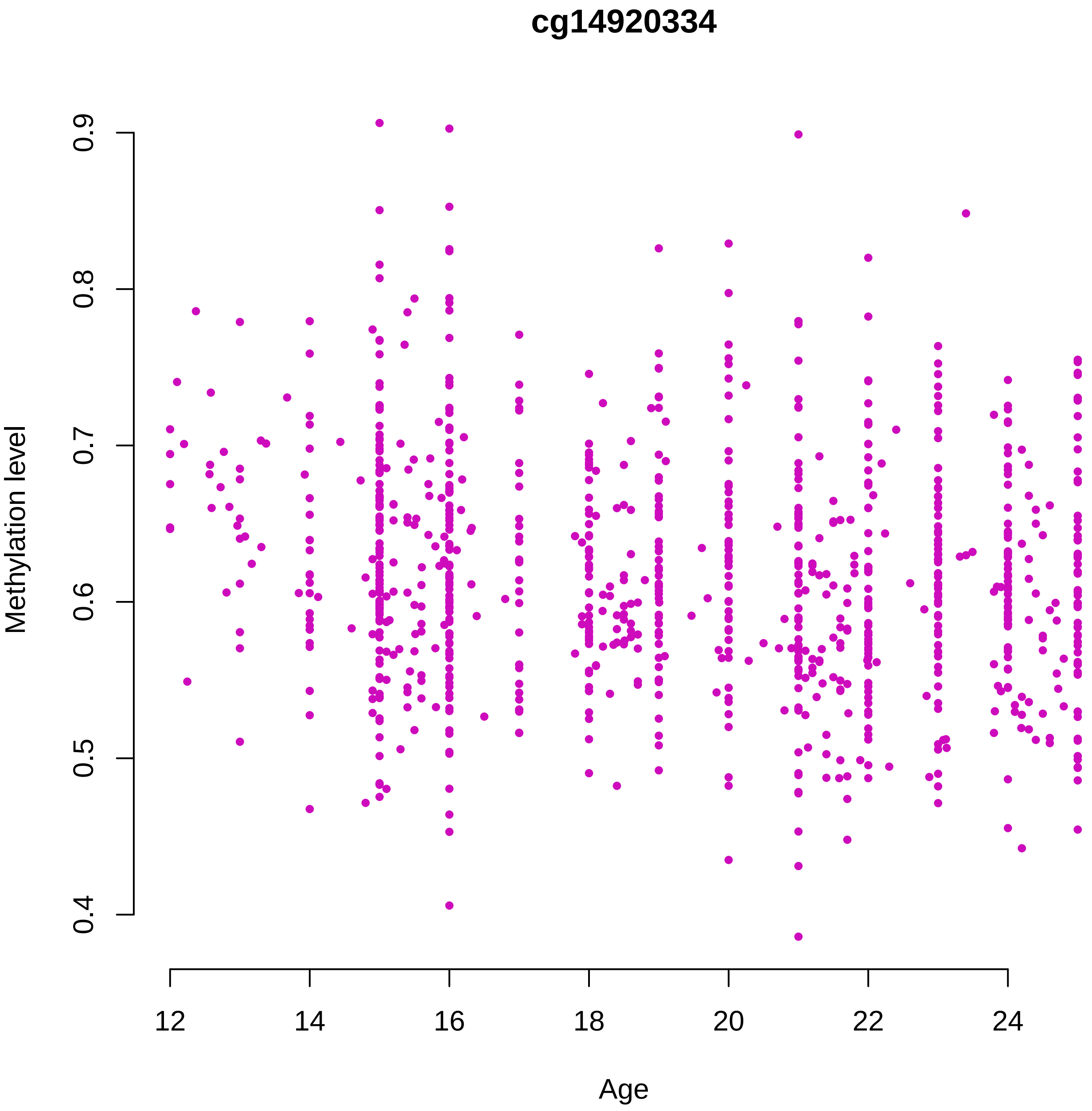
cg14920334
Methylation level
0.4
0.5
0.6
0.7
0.8
0.9
12
14
16
18
20
22
24
Age

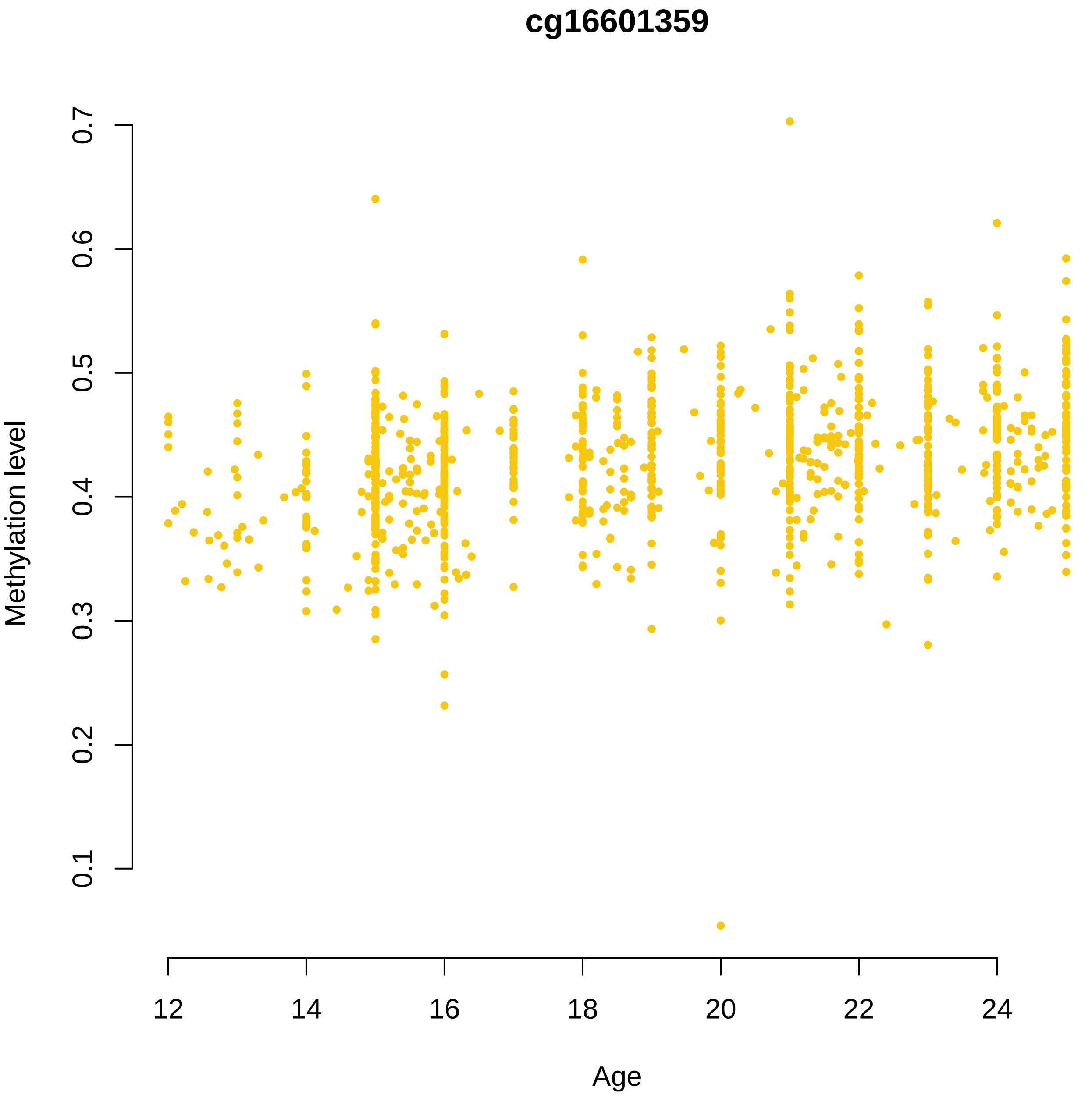

cg16601359
Methylation level
0.1
0.2
0.3
0.4
0.5
0.6
0.7
Age
12
14
16
18
20
22
24

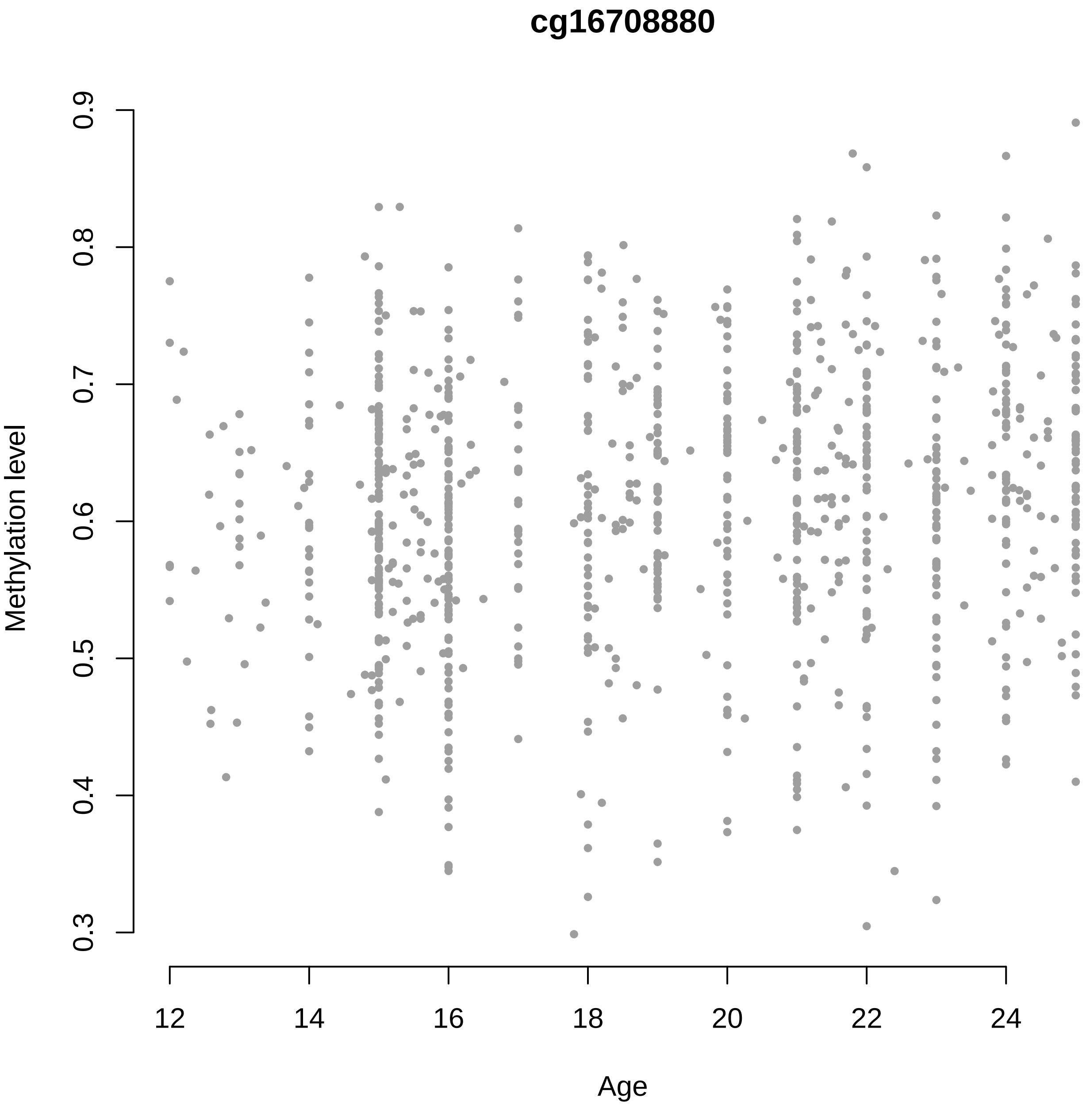
cg16708880
Methylation level
0.3
0.4
0.5
0.6
0.7
0.8
0.9
Age
12
14
16
18
20
22
24

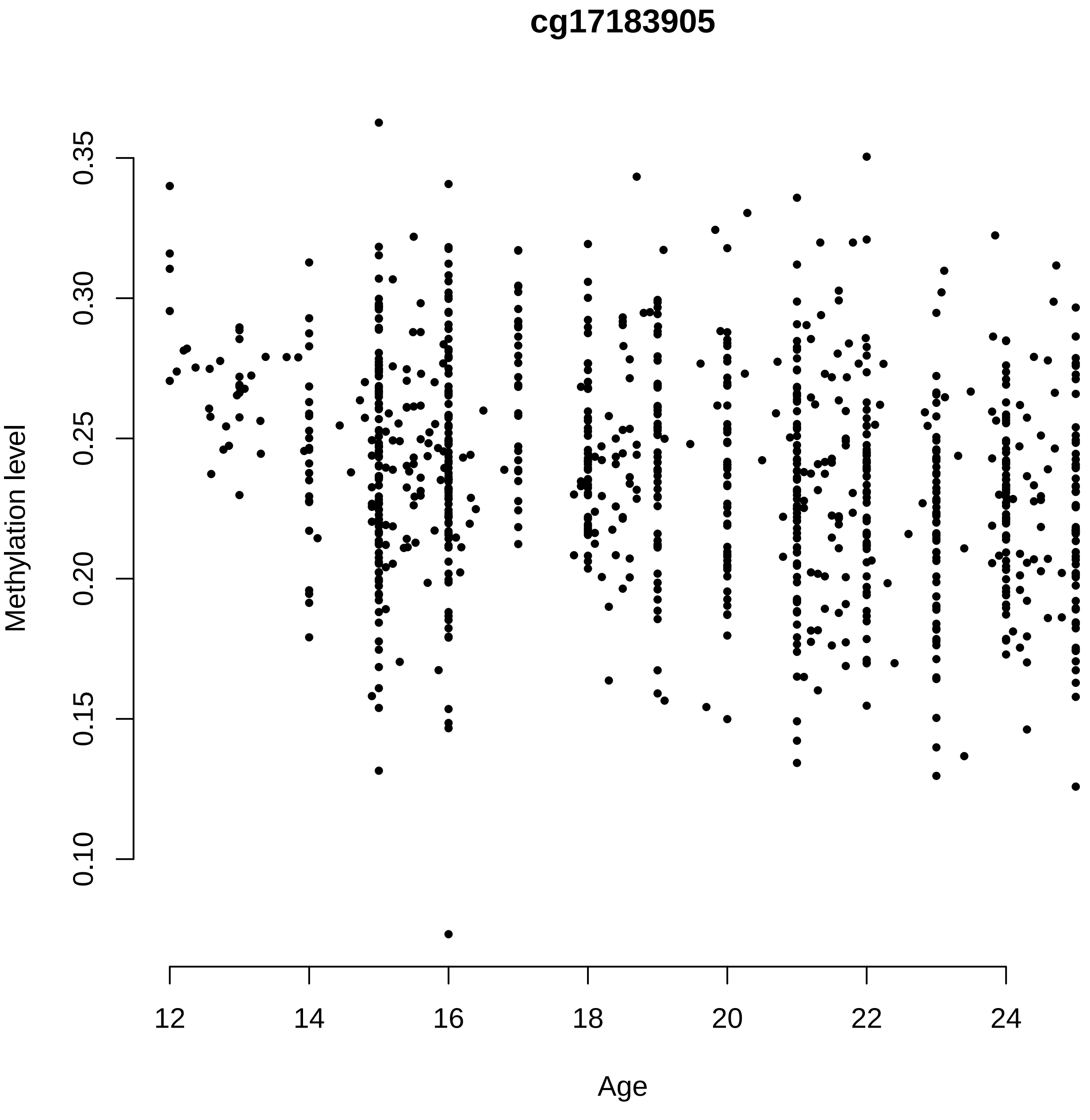
cg17183905
Methylation level
0.10
0.15
0.20
0.25
0.30
0.35
12
14
16
18
20
22
24
Age

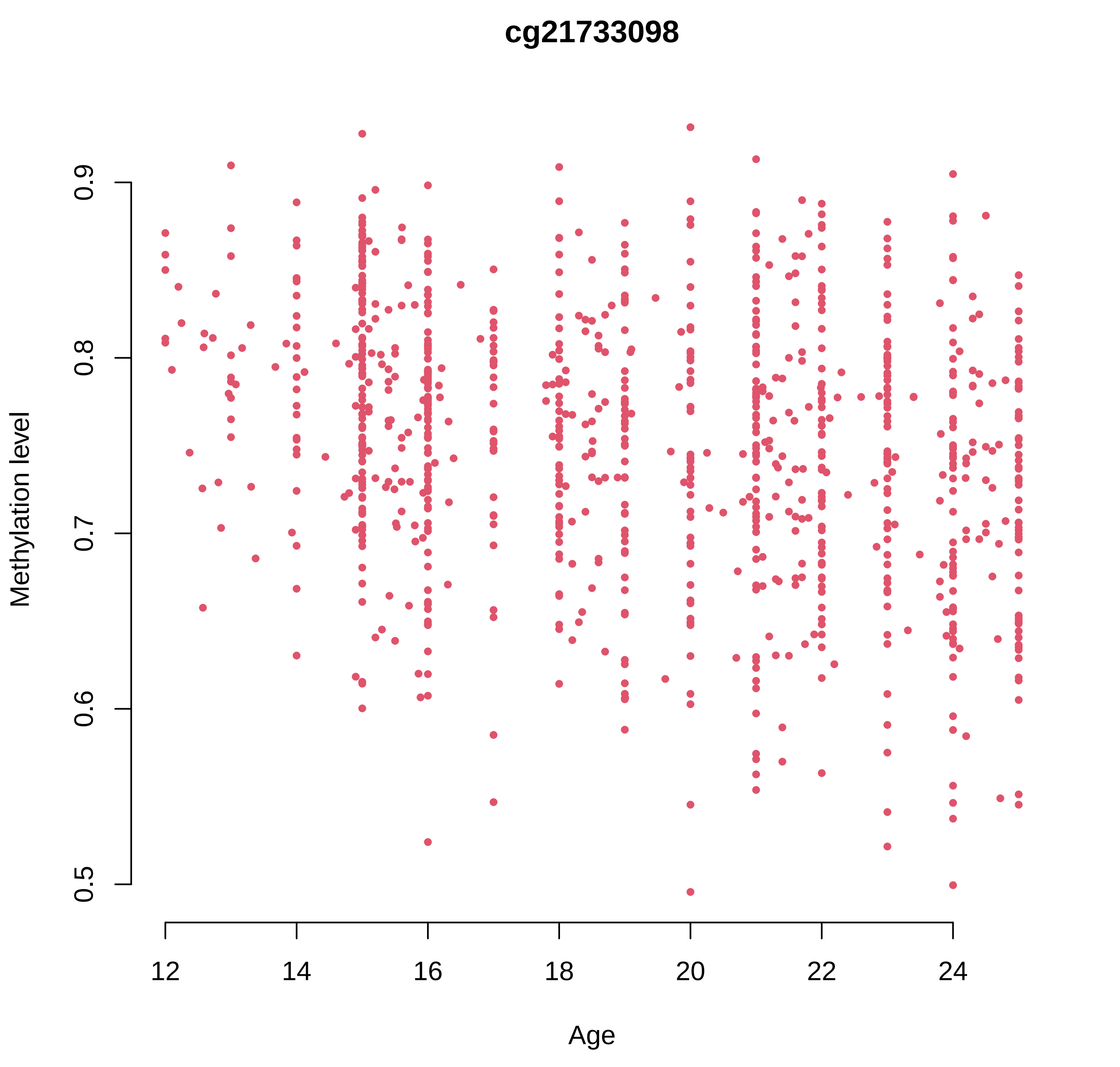
cg21733098
Methylation level
0.5
0.6
0.7
0.8
0.9
Age
12
14
16
18
20
22
24

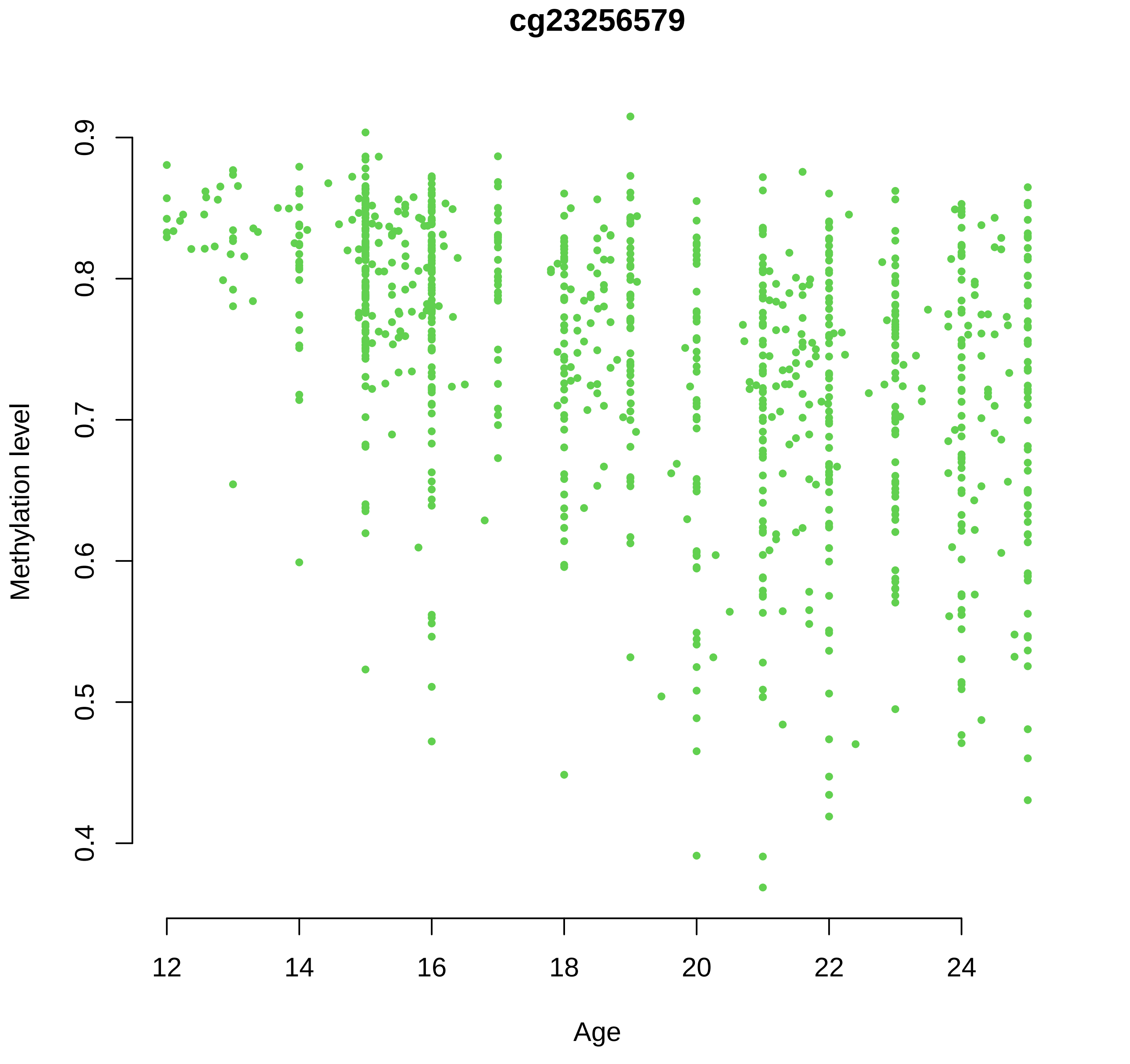

cg23256579
Methylation level
0.4
0.5
0.6
0.7
0.8
0.9
Age
12
14
16
18
20
22
24

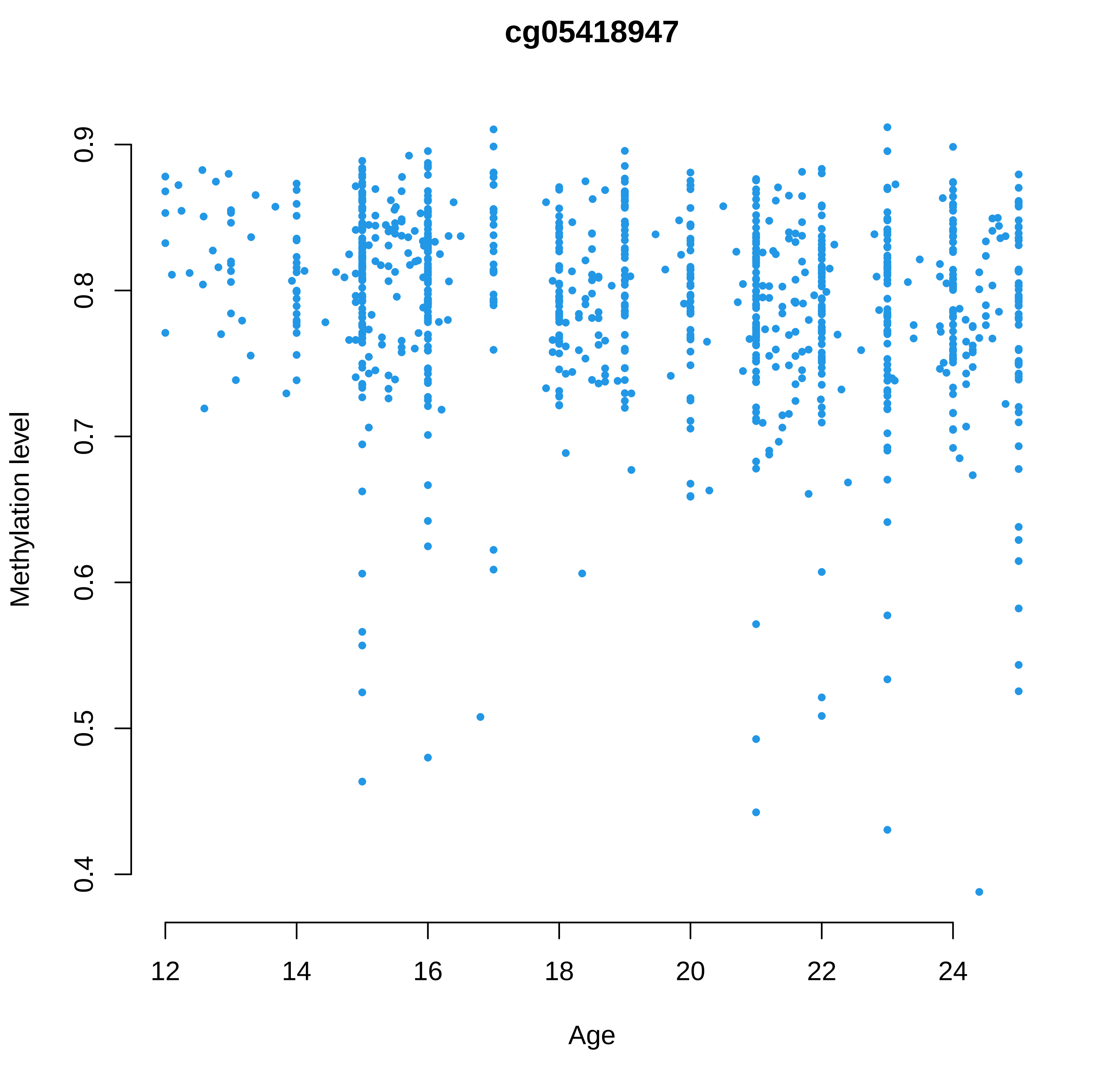

cg05418947
Methylation level
0.4
0.5
0.6
0.7
0.8
0.9
Age
12
14
16
18
20
22
24

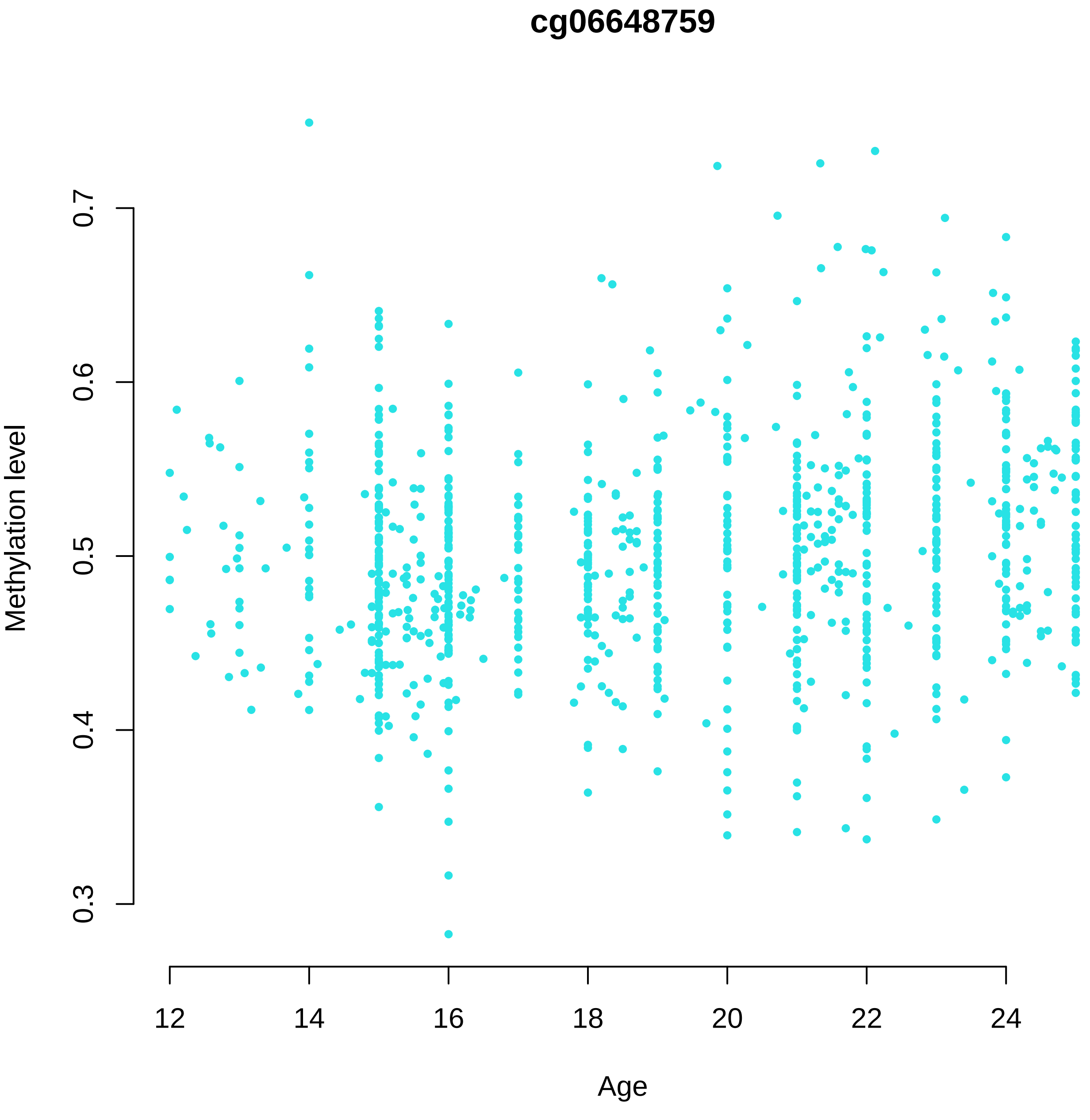
cg06648759
Methylation level
0.3
0.4
0.5
0.6
0.7
Age
12
14
16
18
20
22
24

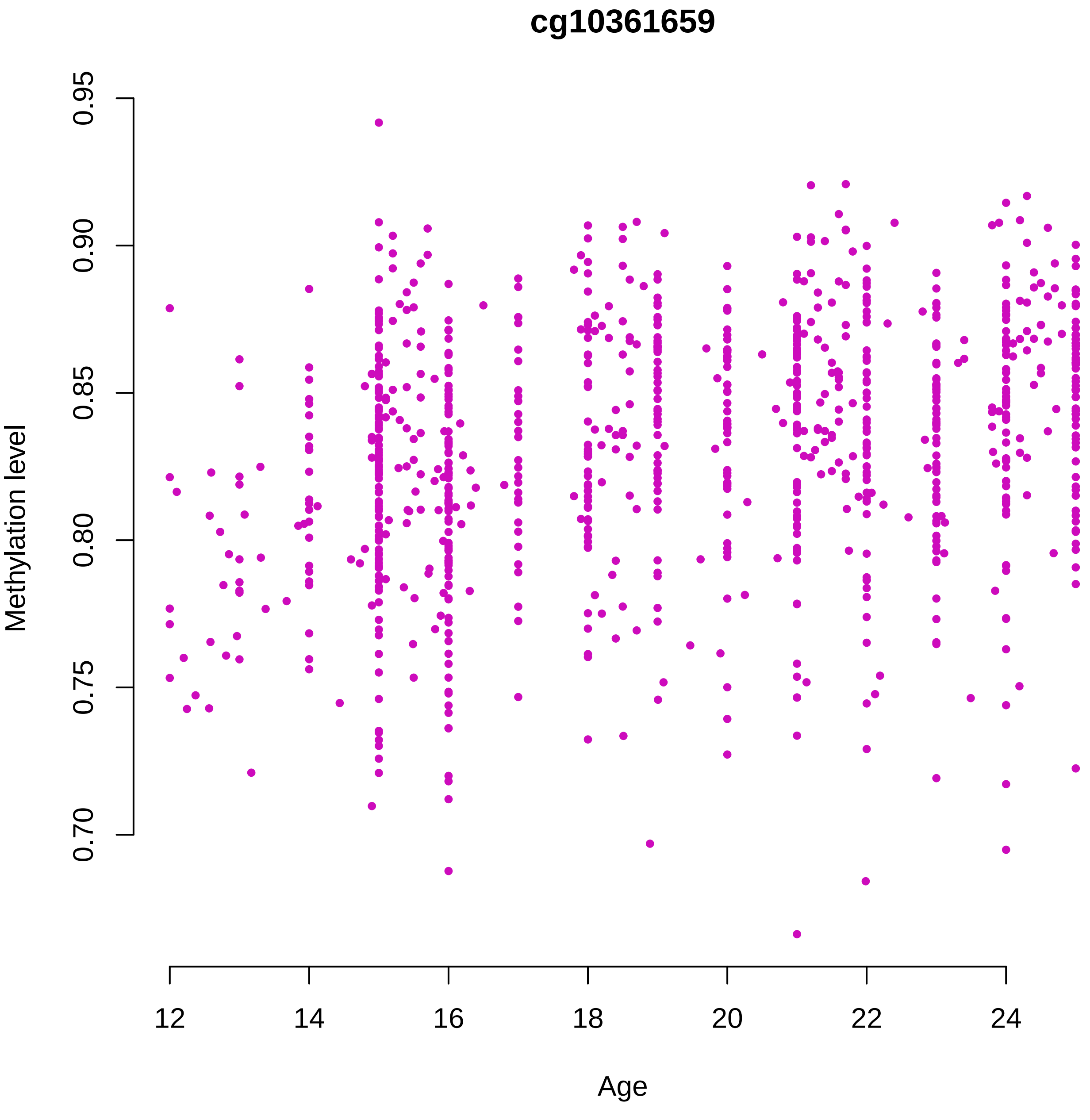
cg10361659
Methylation level
0.70
0.75
0.80
0.85
0.90
0.95
Age
12
14
16
18
20
22
24

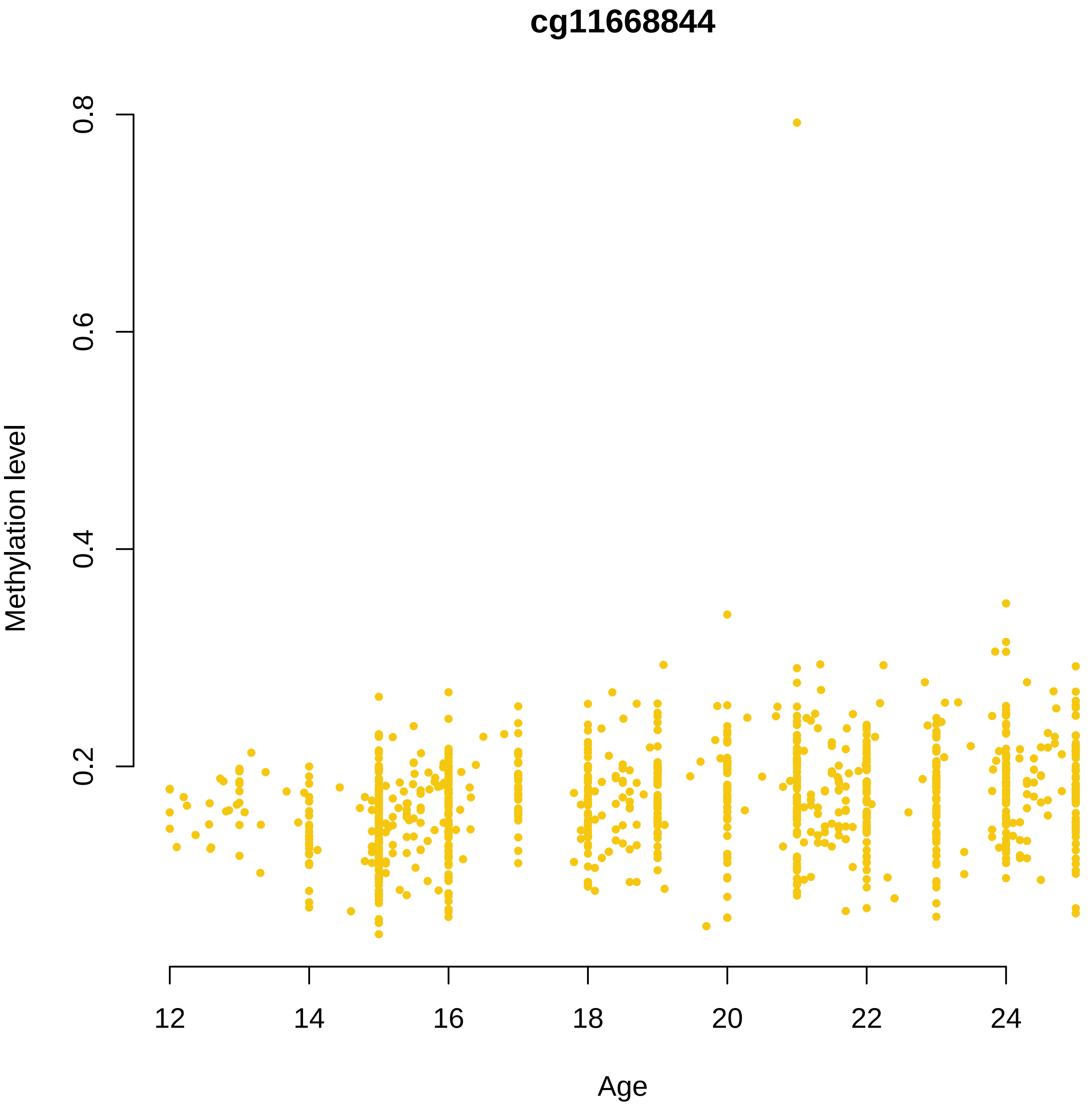
cg11668844
Methylation level
0.2
0.4
0.6
0.8
Age
12
14
16
18
20
22
24

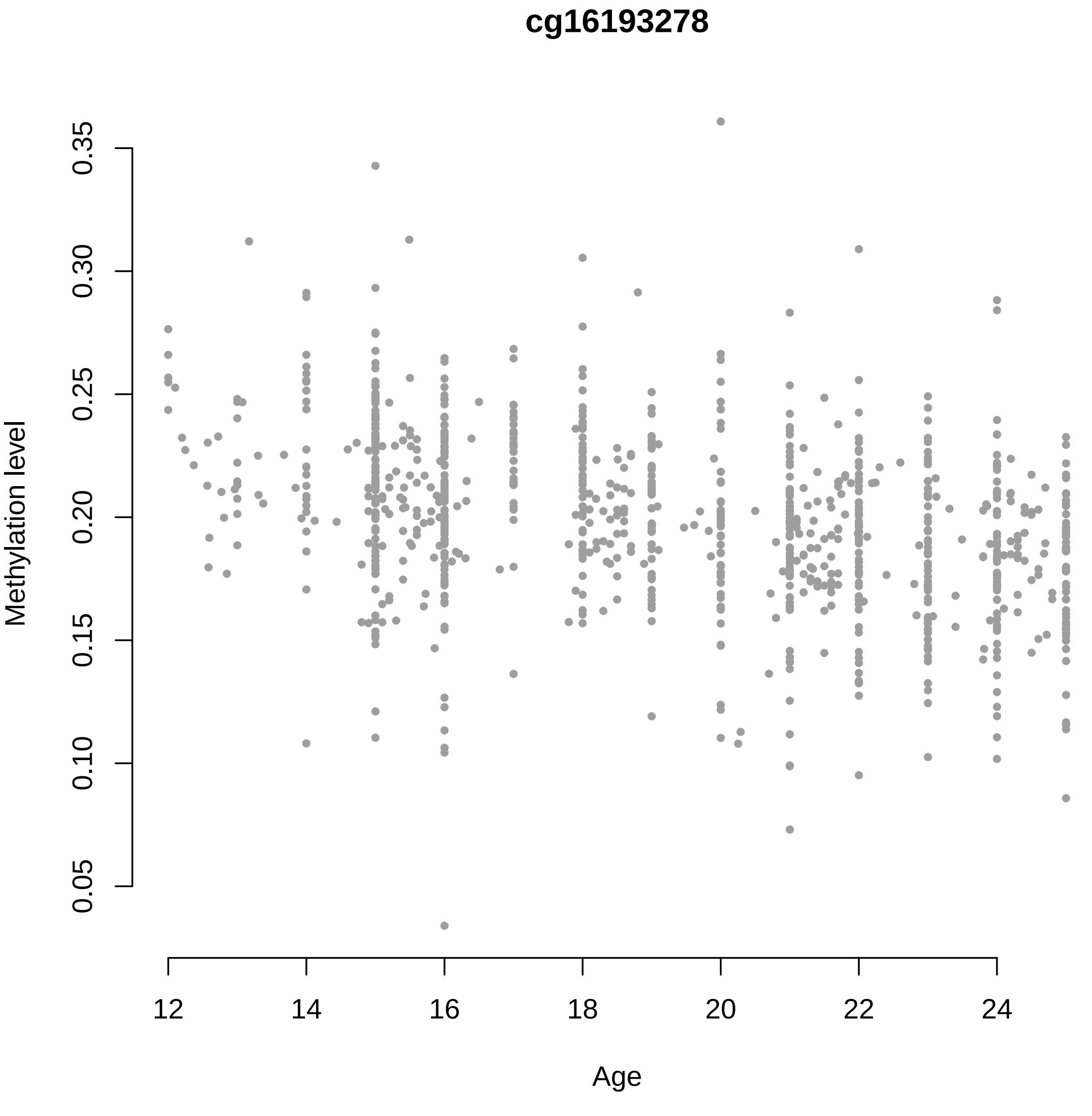
cg16193278
Methylation level
0.05
0.10
0.15
0.20
0.25
0.30
0.35
Age
12
14
16
18
20
22
24

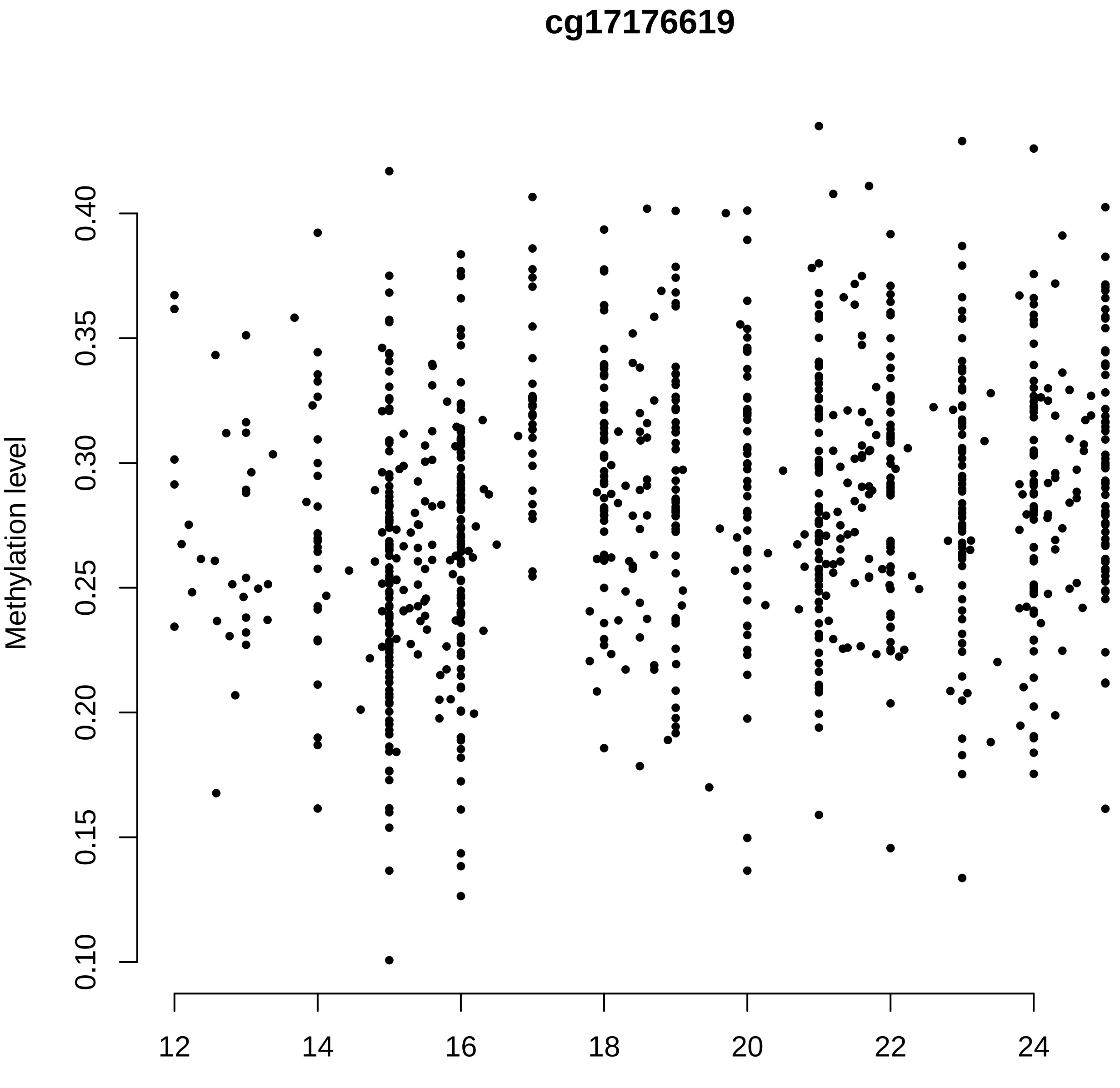
cg17176619
Methylation level
0.10
0.15
0.20
0.25
0.30
0.35
0.40
Age
12
14
16
18
20
22
24

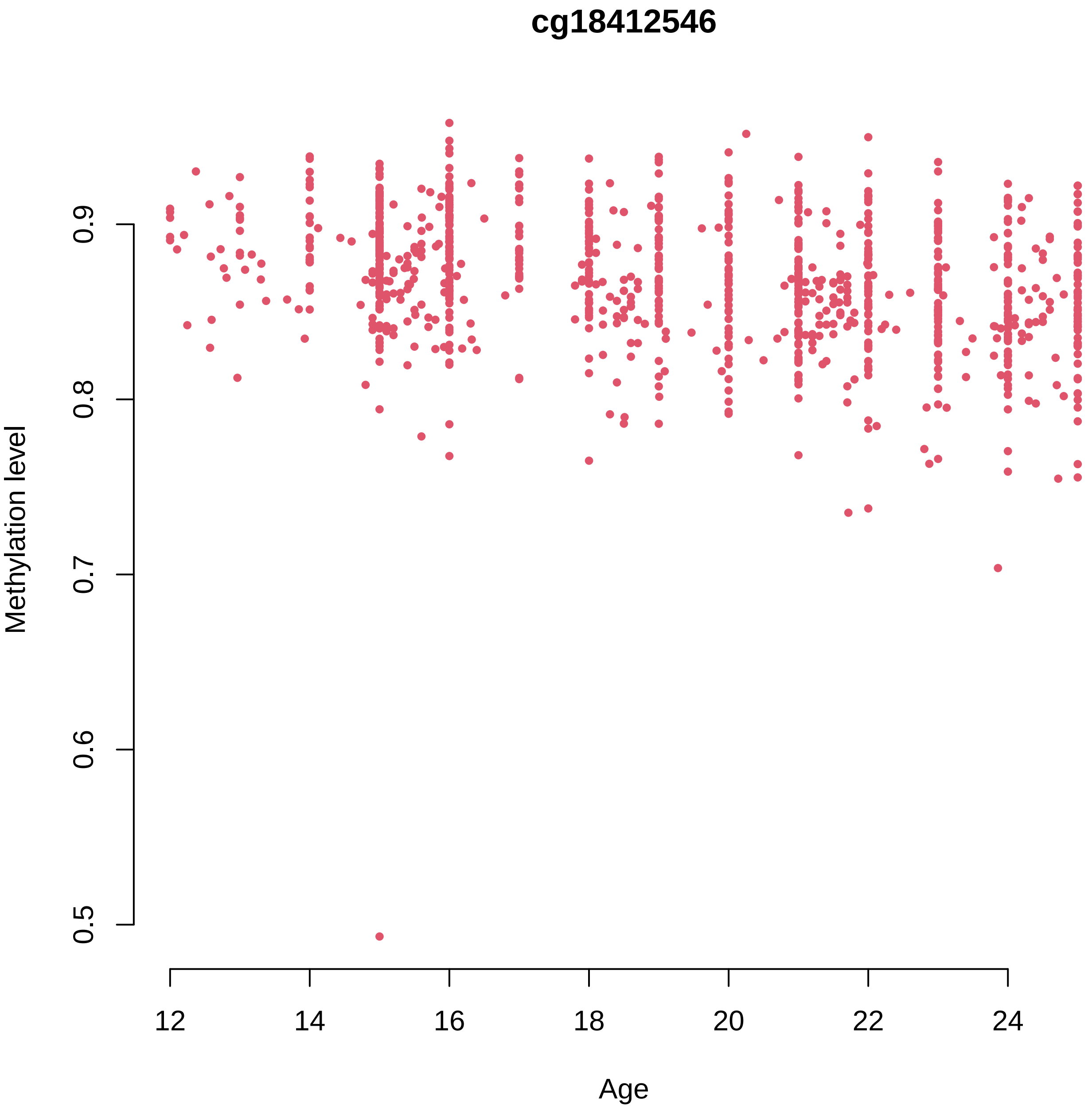
cg18412546
Methylation level
0.5
0.6
0.7
0.8
0.9
Age
12
14
16
18
20
22
24

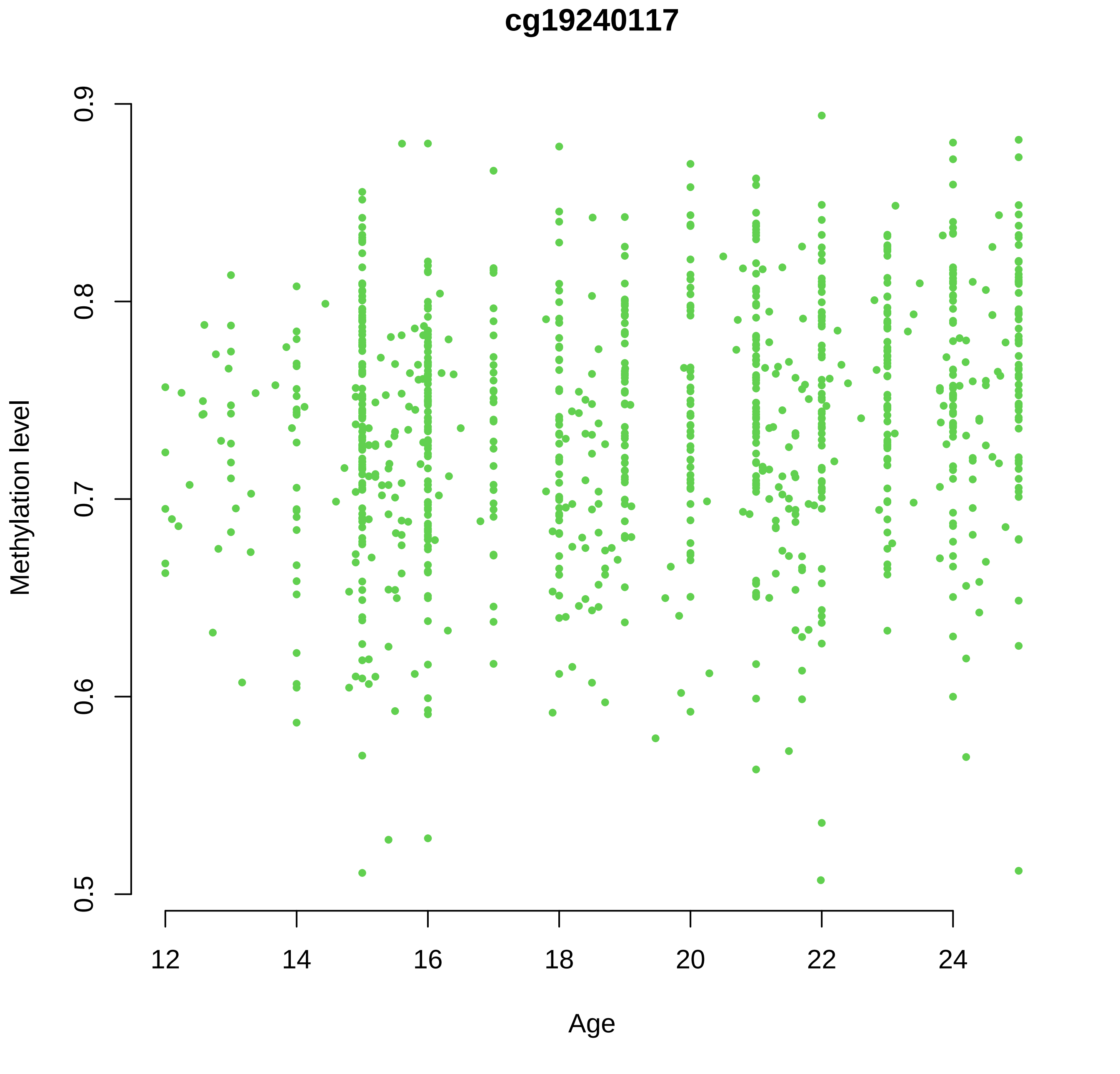
cg19240117
Methylation level
0.5
0.6
0.7
0.8
0.9
Age
12
14
16
18
20
22
24

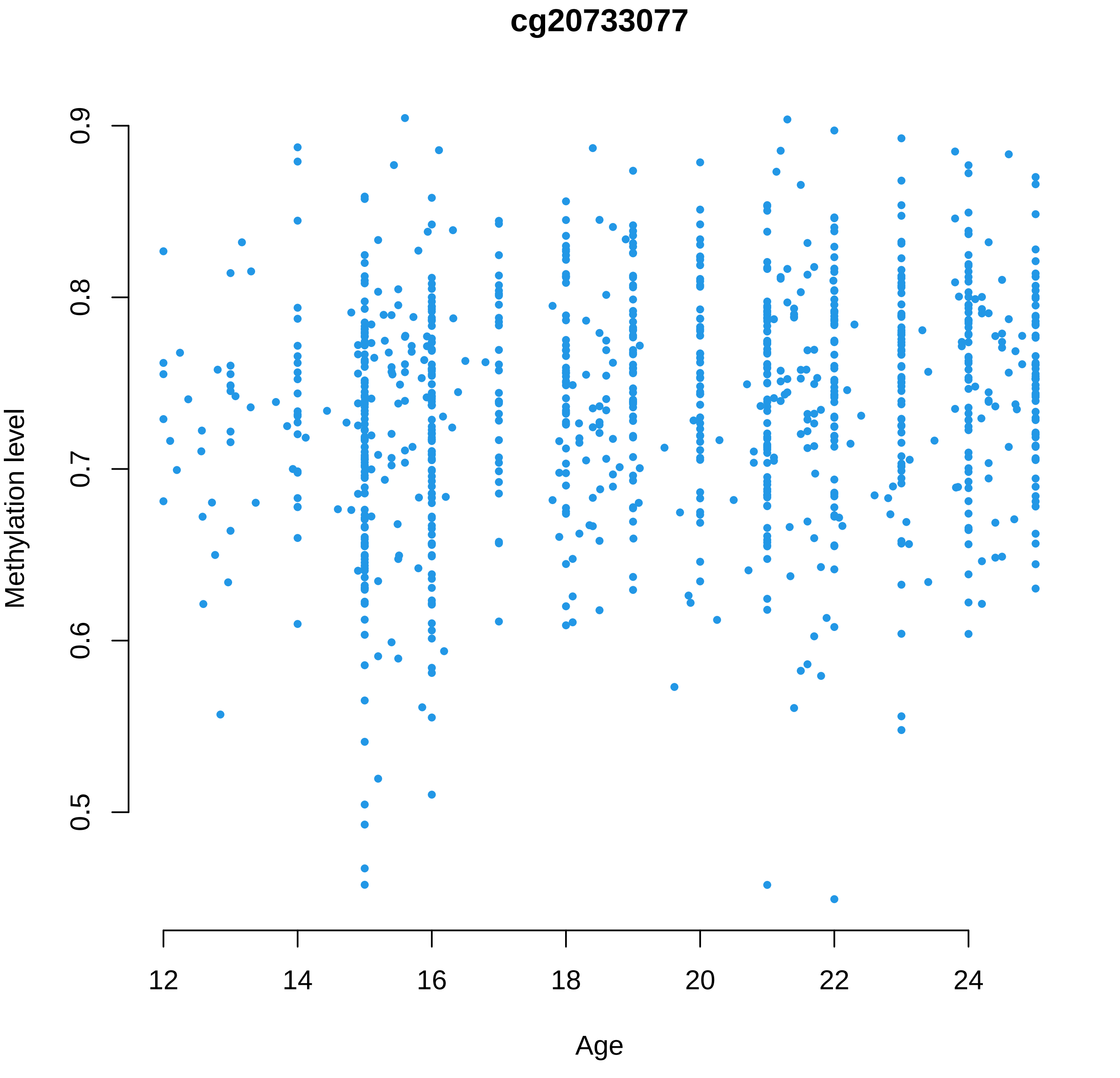
cg20733077
Methylation level
0.5
0.6
0.7
0.8
0.9
Age
12
14
16
18
20
22
24

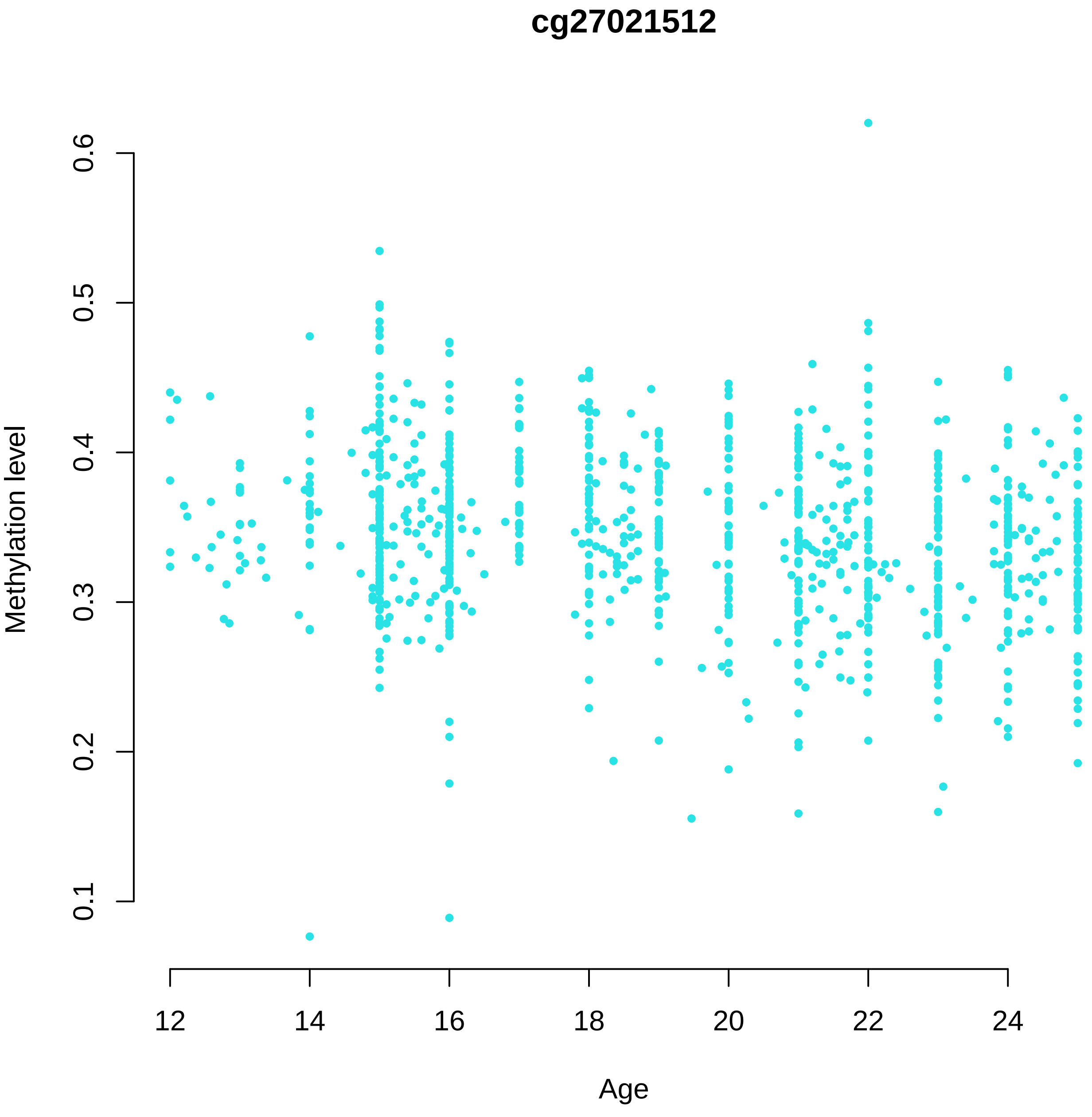
cg27021512
Methylation level
0.1
0.2
0.3
0.4
0.5
0.6
Age
12
14
16
18
20
22
24

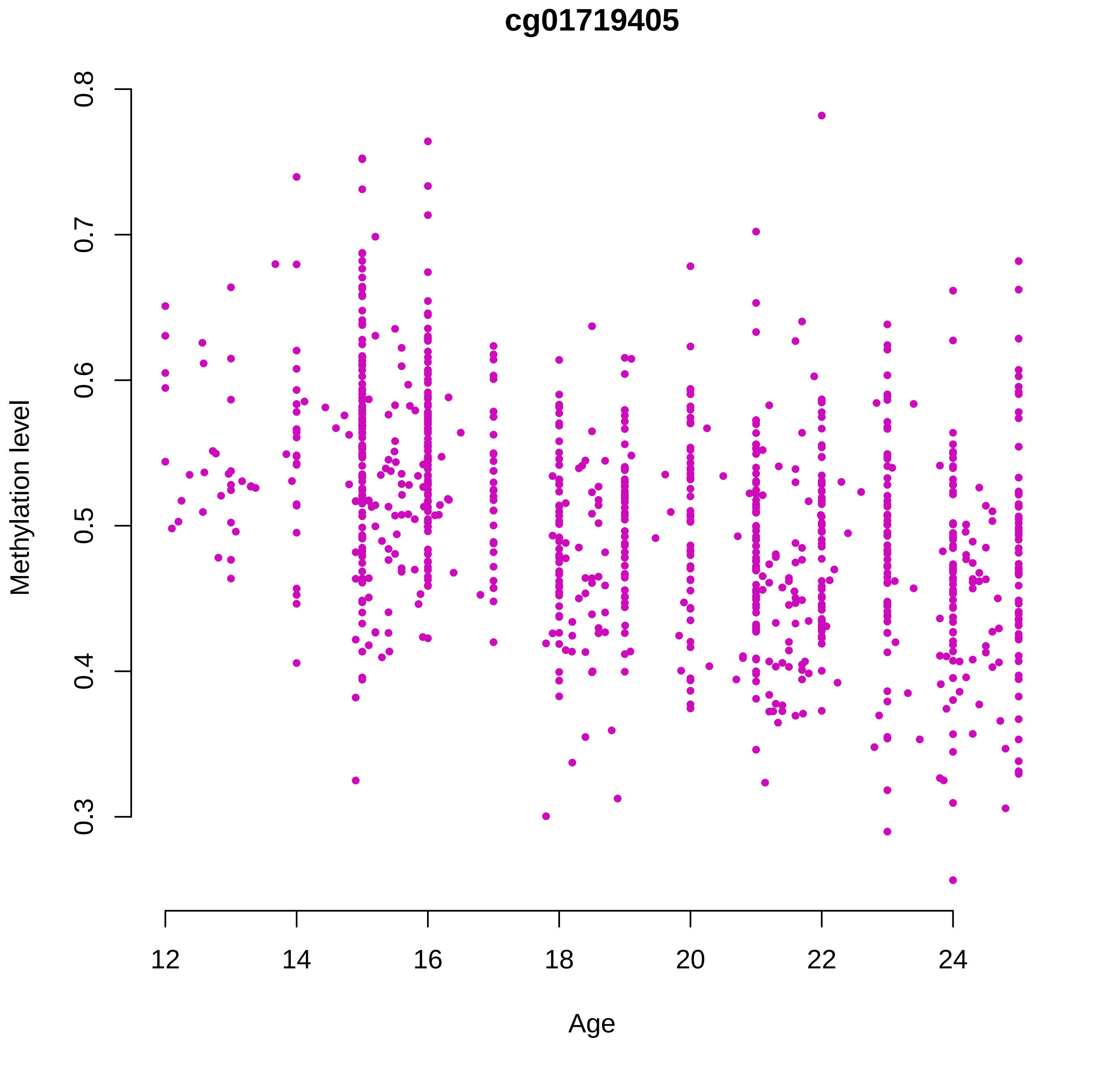
cg01719405
Methylation level
0.3
0.4
0.5
0.6
0.7
0.8
Age
12
14
16
18
20
22
24

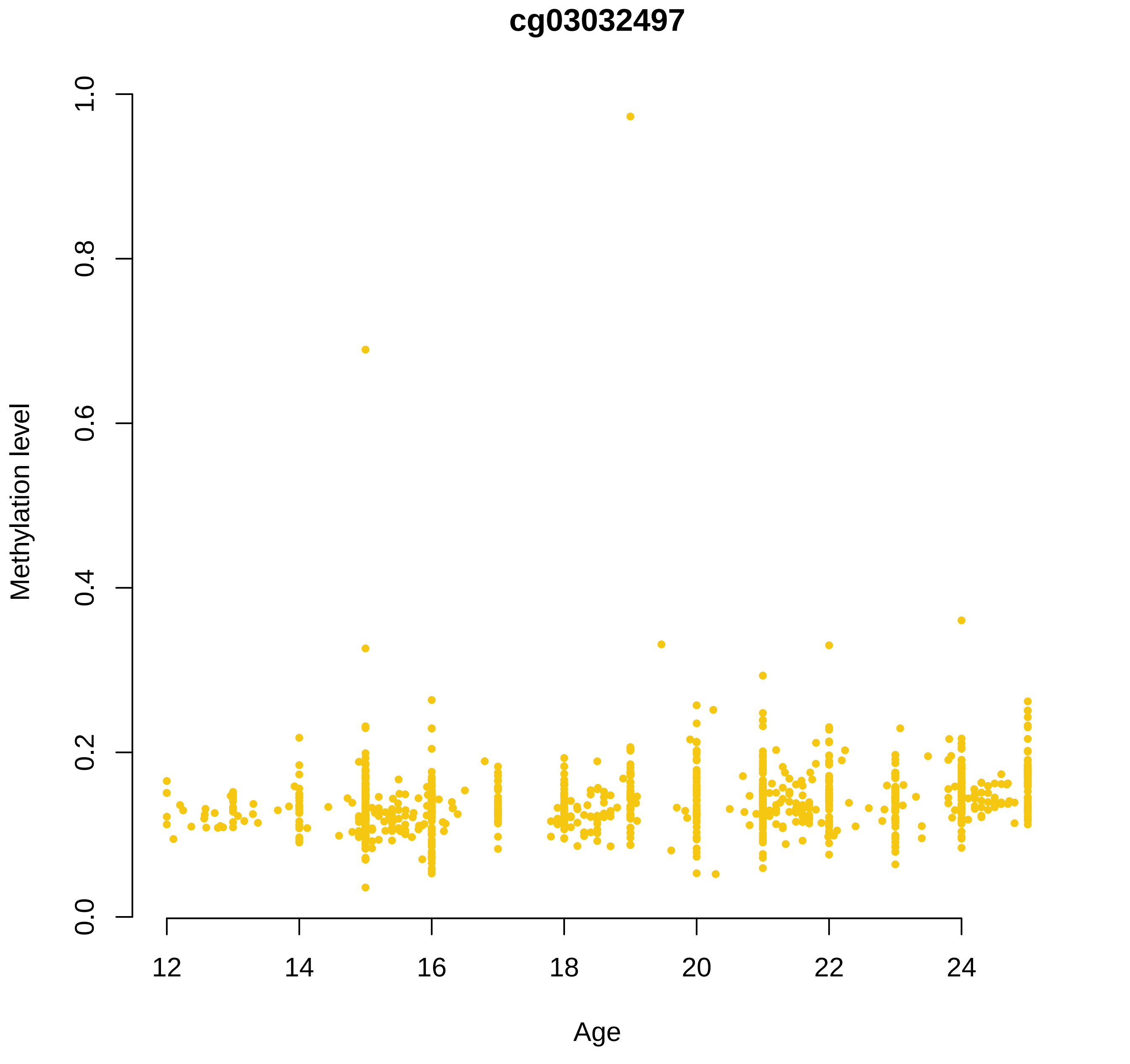

cg03032497
Methylation level
0.0
0.2
0.4
0.6
0.8
1.0
12
14
16
18
20
22
24
Age

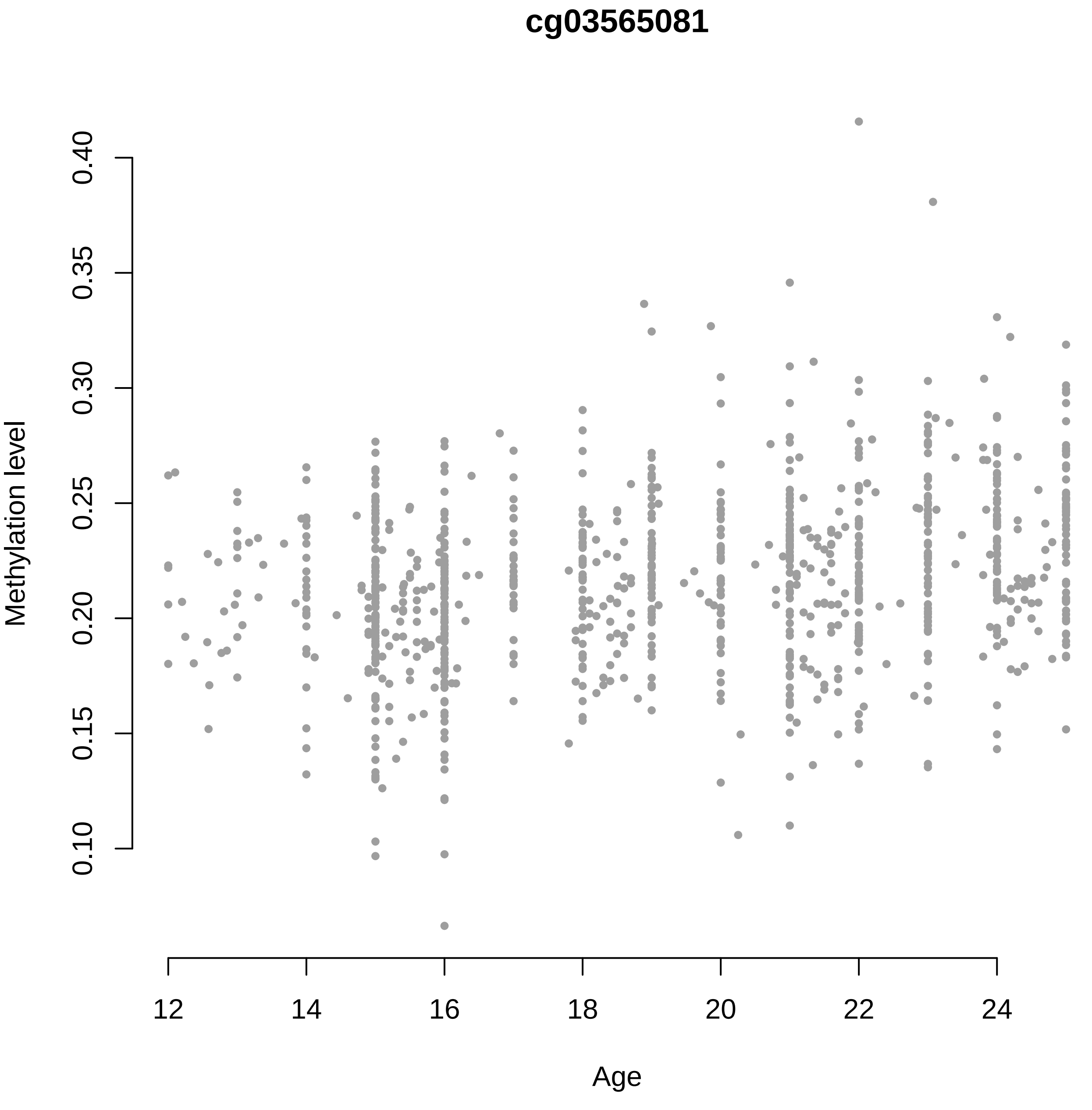
cg03565081
Methylation level
0.10
0.15
0.20
0.25
0.30
0.35
0.40
Age
12
14
16
18
20
22
24

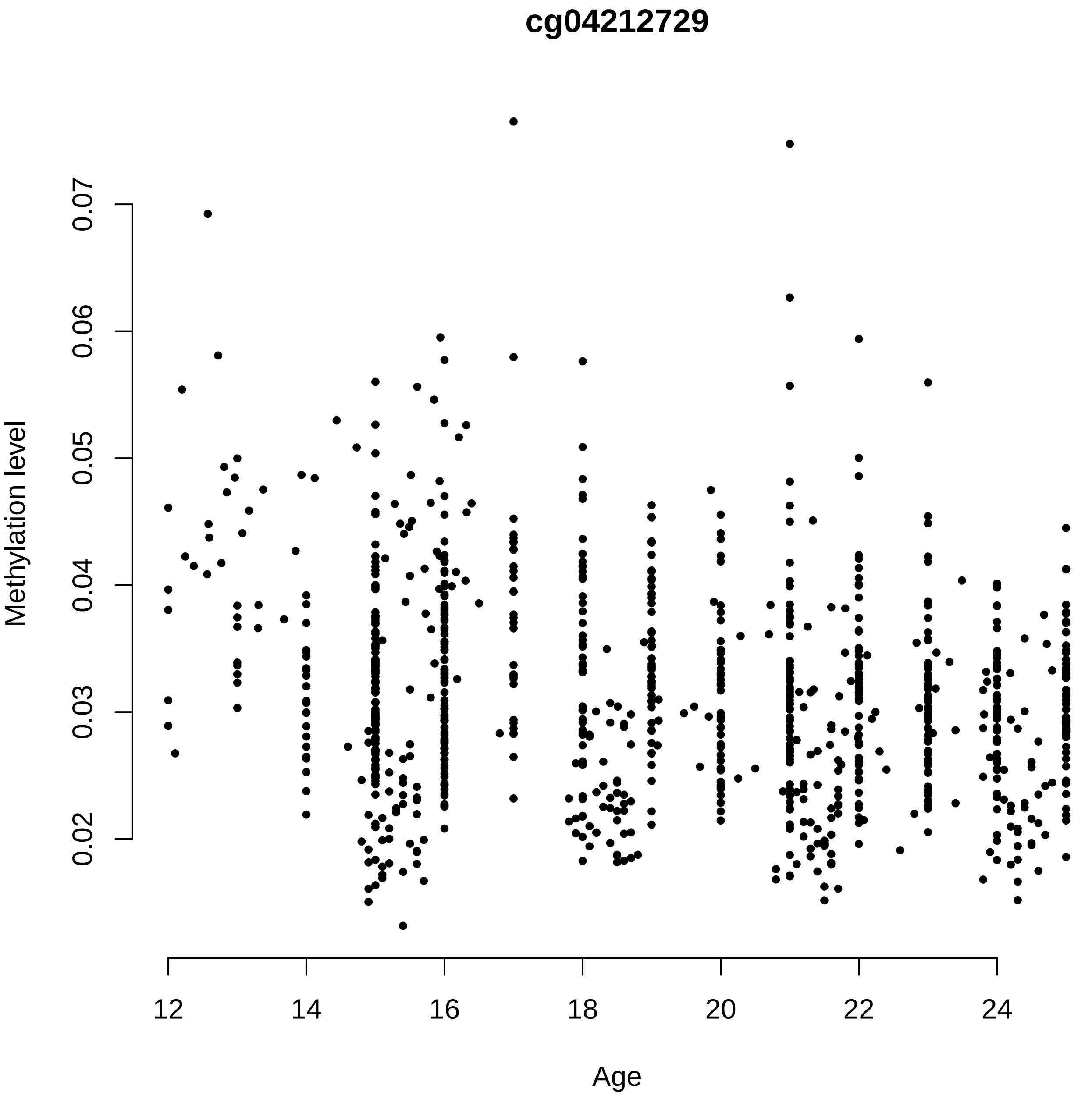

cg04212729
Methylation level
0.02
0.03
0.04
0.05
0.06
0.07
12
14
16
18
20
22
24
Age

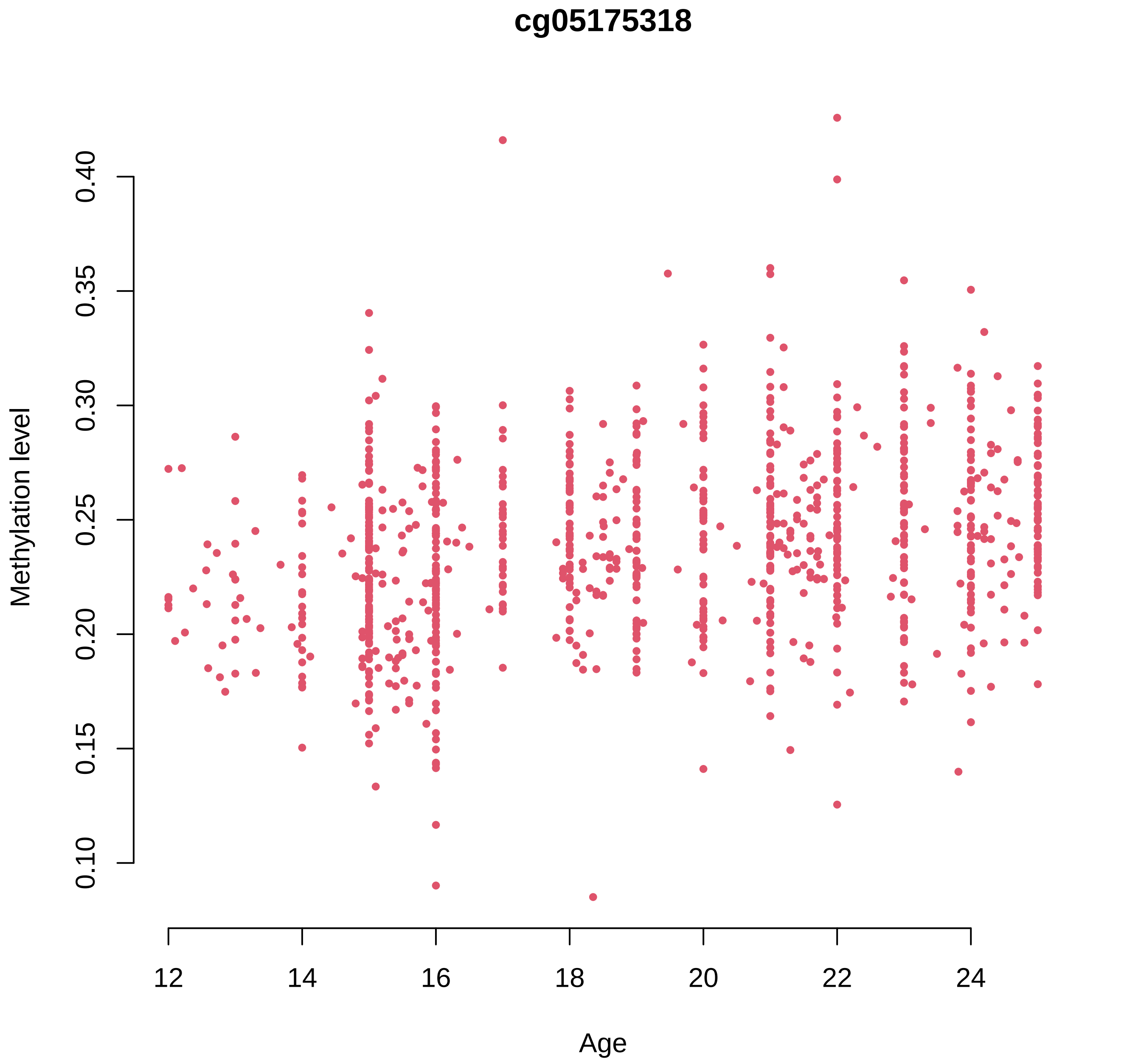
cg05175318
Methylation level
0.10
0.15
0.20
0.25
0.30
0.35
0.40
Age
12
14
16
18
20
22
24

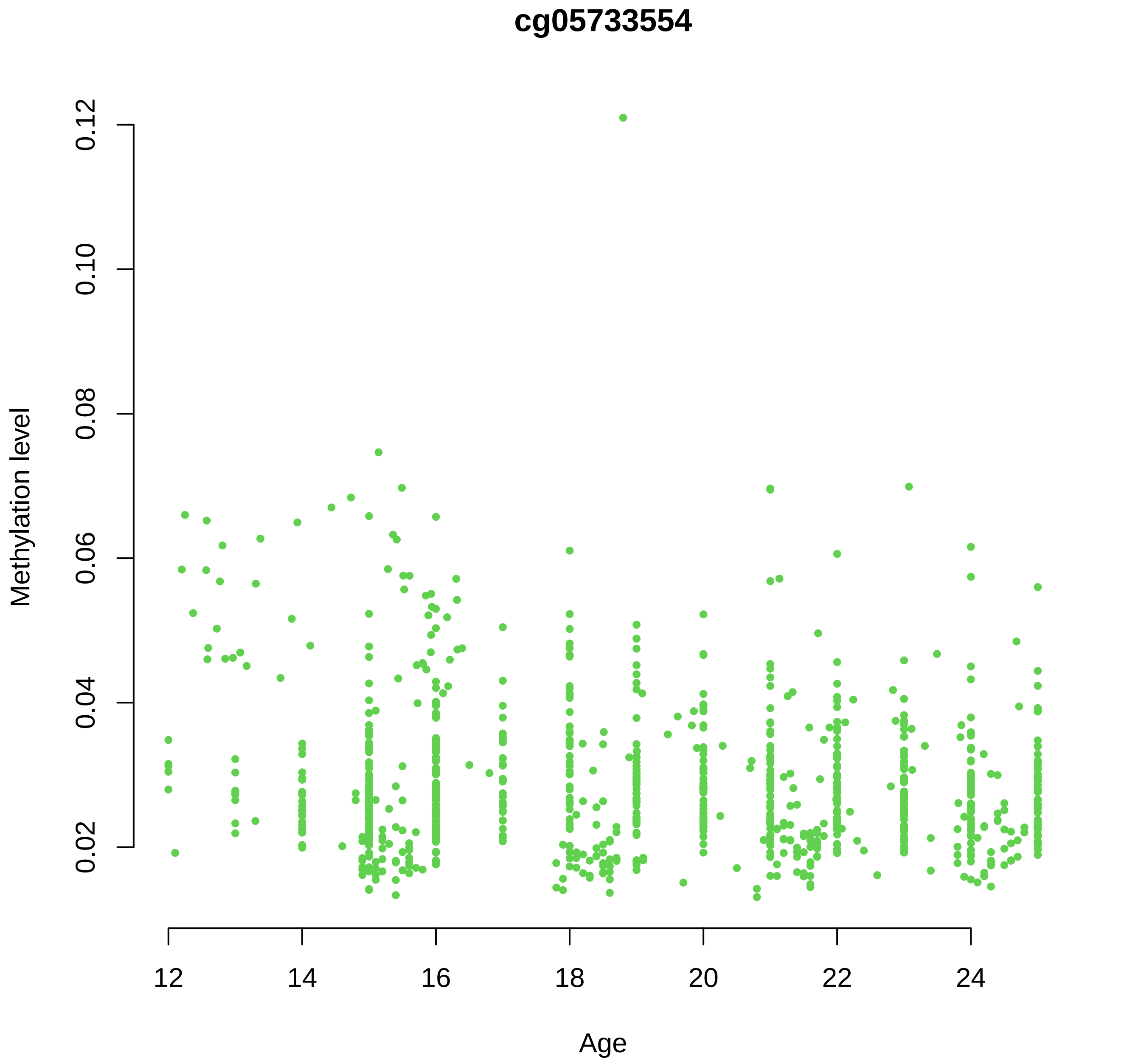
cg05733554
Methylation level
0.02
0.04
0.06
0.08
0.10
0.12
12
14
16
18
20
22
24
Age

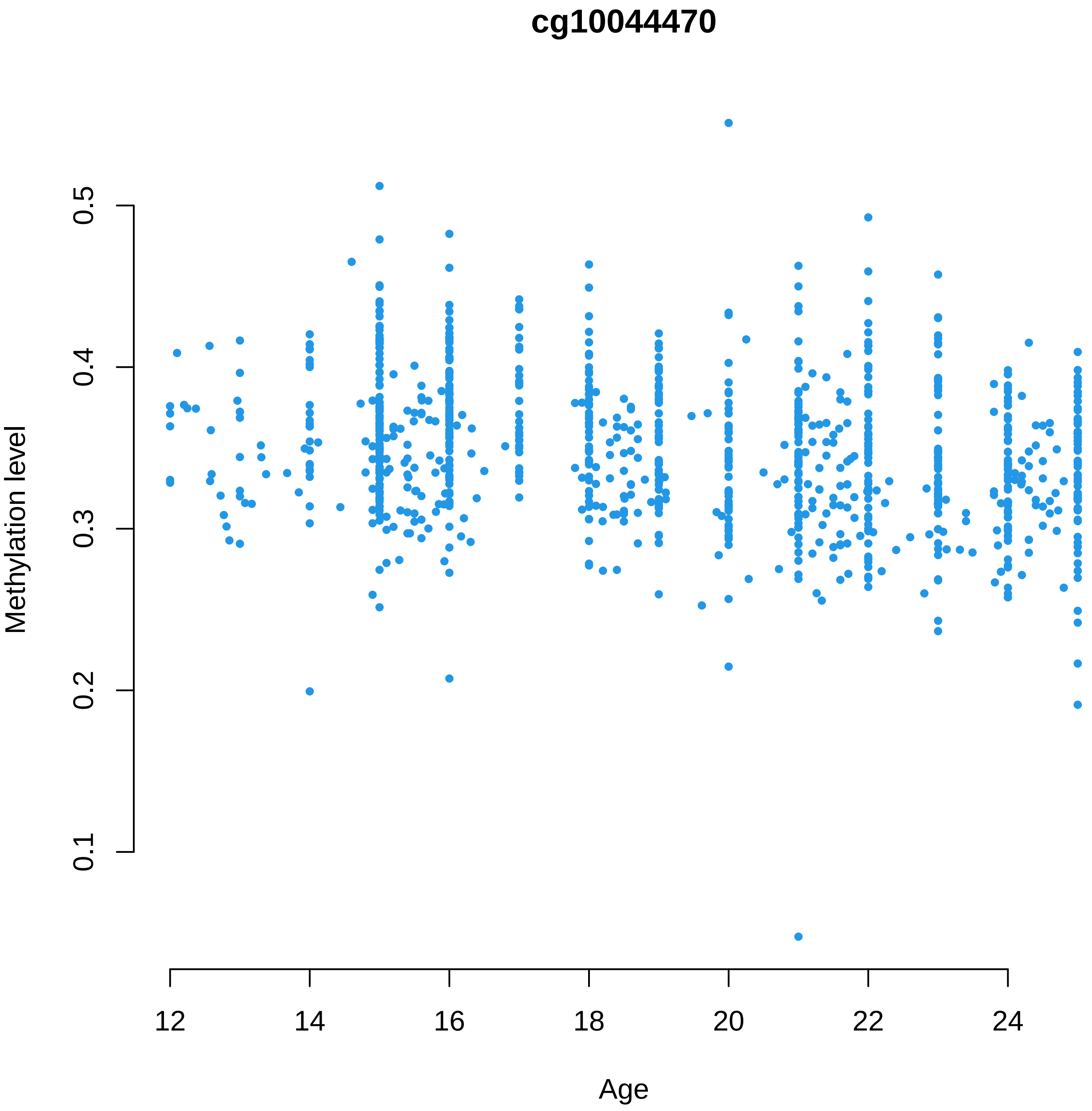
cg10044470
Methylation level
Age
0.1
0.2
0.3
0.4
0.5
12
14
16
18
20
22
24

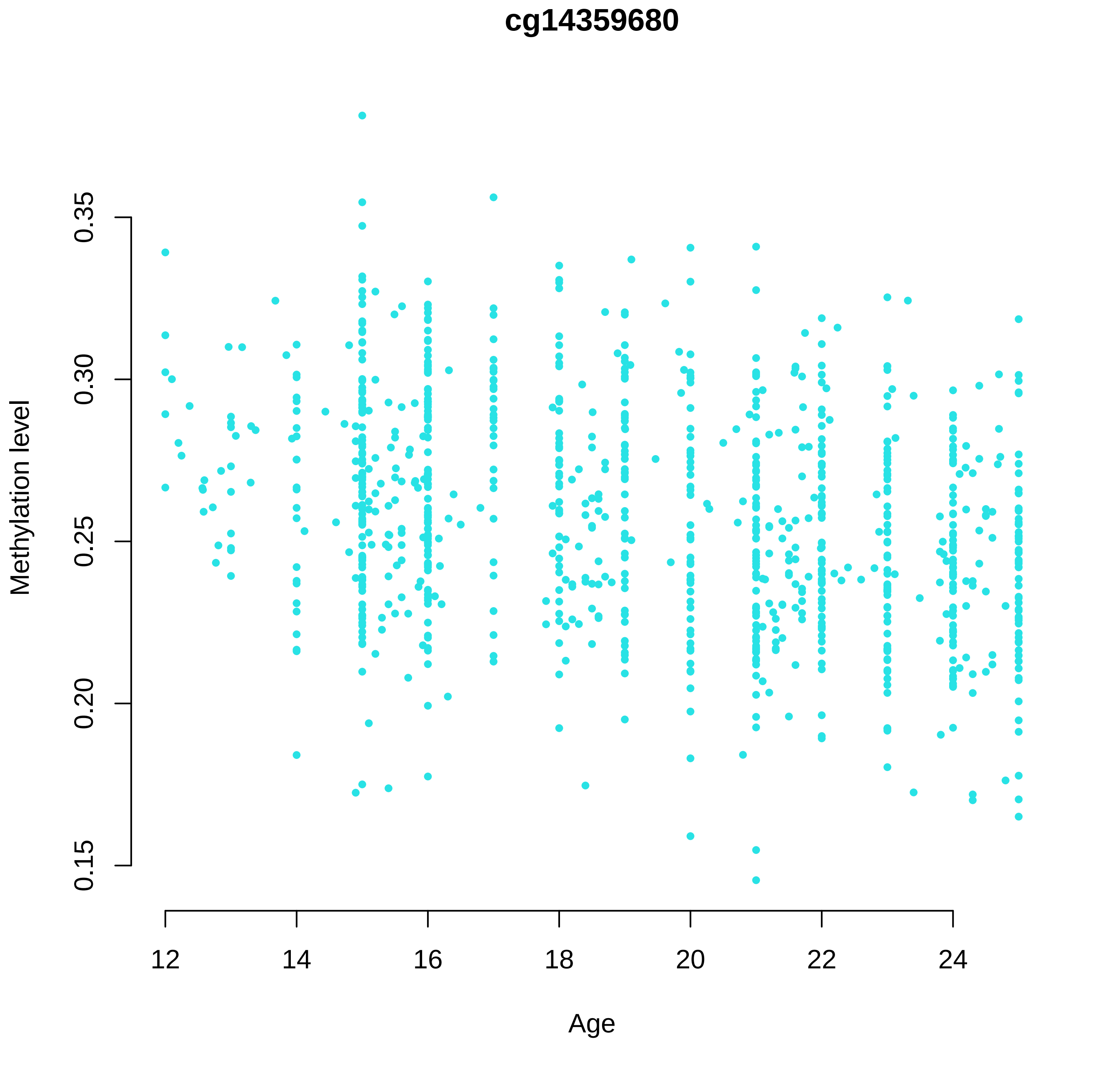
cg14359680
Methylation level
0.15
0.20
0.25
0.30
0.35
Age
12
14
16
18
20
22
24

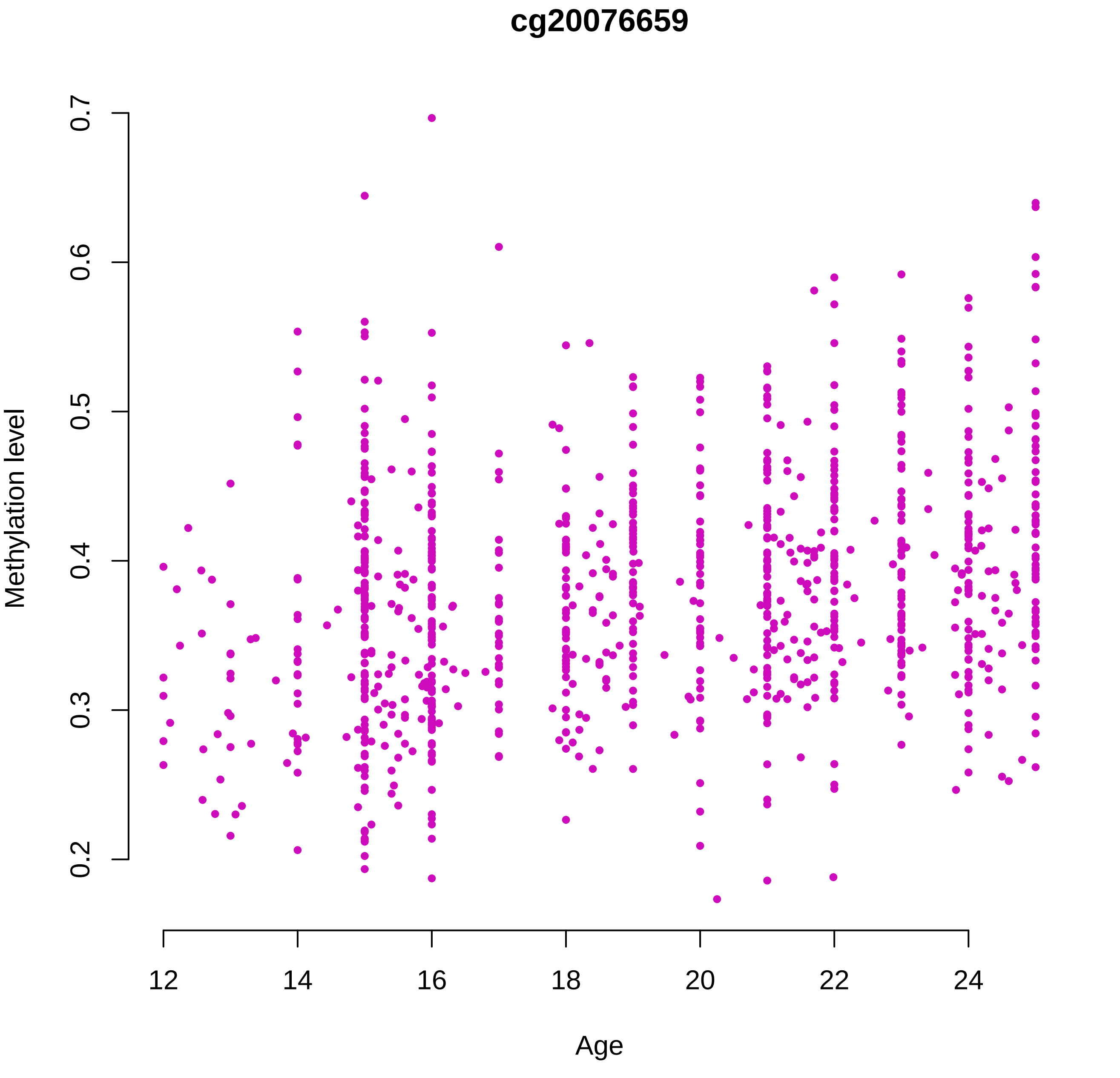

cg20076659
Methylation level
0.2
0.3
0.4
0.5
0.6
0.7
12
14
16
18
20
22
24
Age

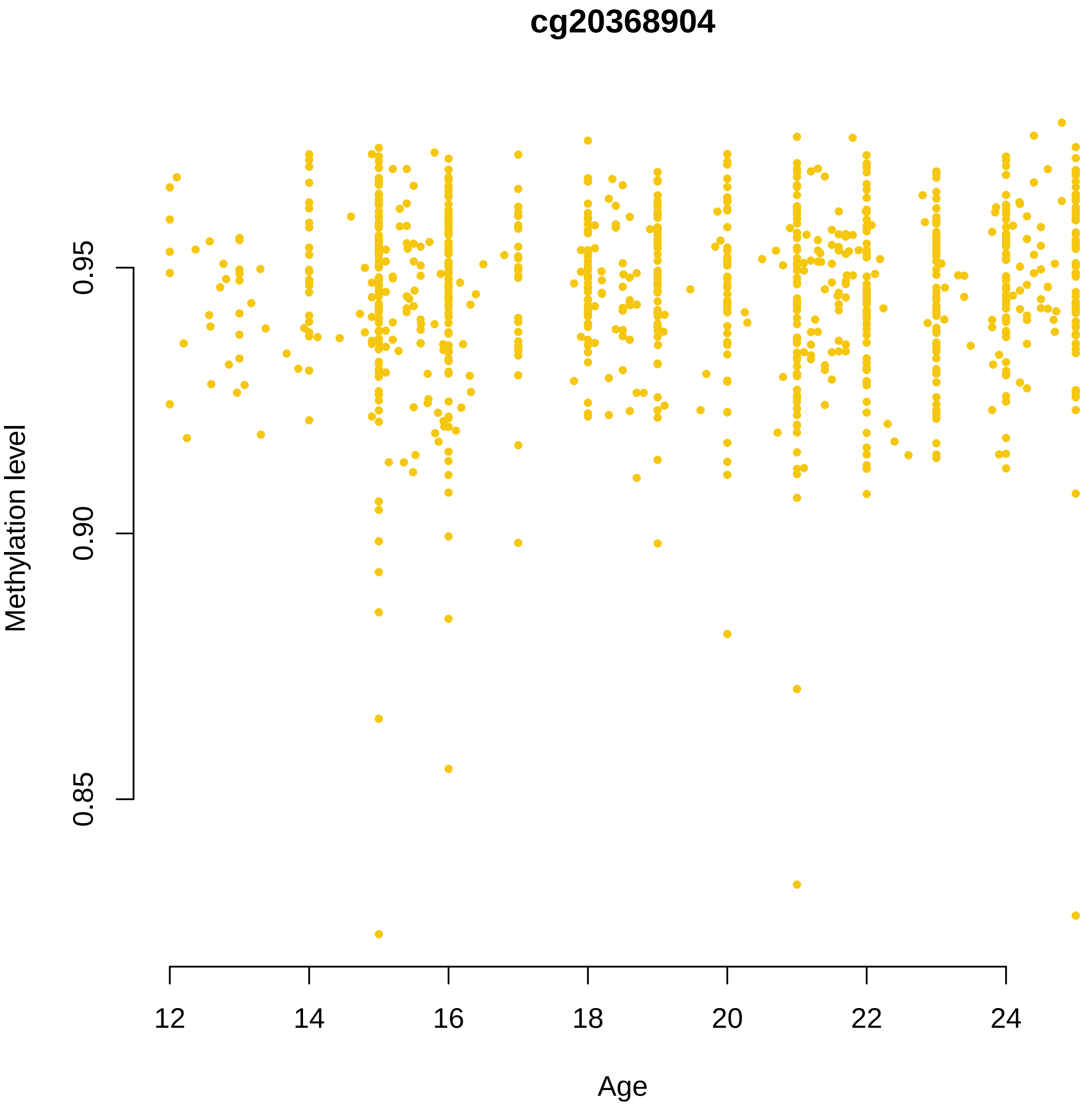

cg20368904
Methylation level
0.95
0.90
0.85
Age
12
14
16
18
20
22
24

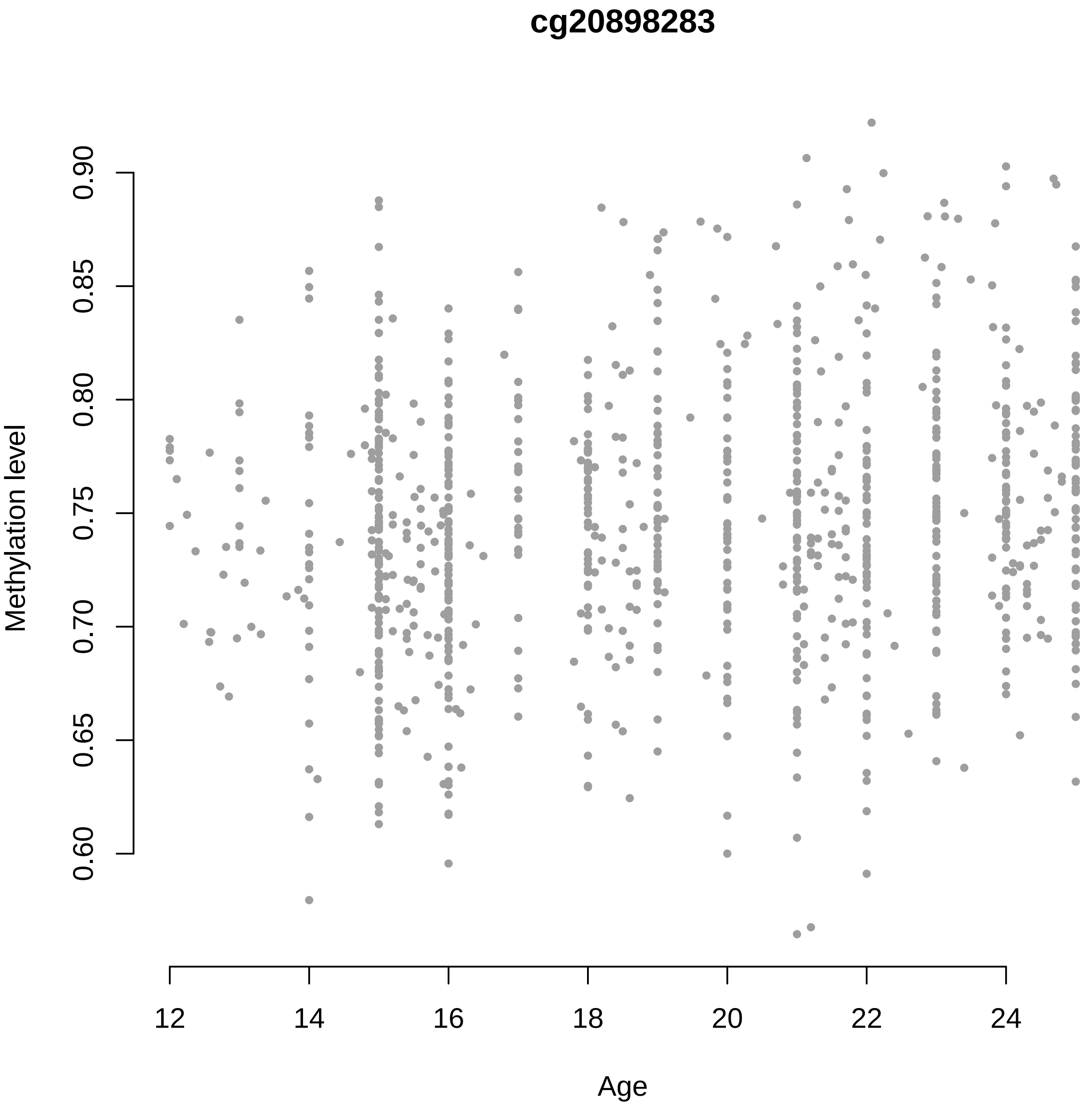
cg20898283
Methylation level
0.60
0.65
0.70
0.75
0.80
0.85
0.90
12
14
16
18
20
22
24
Age

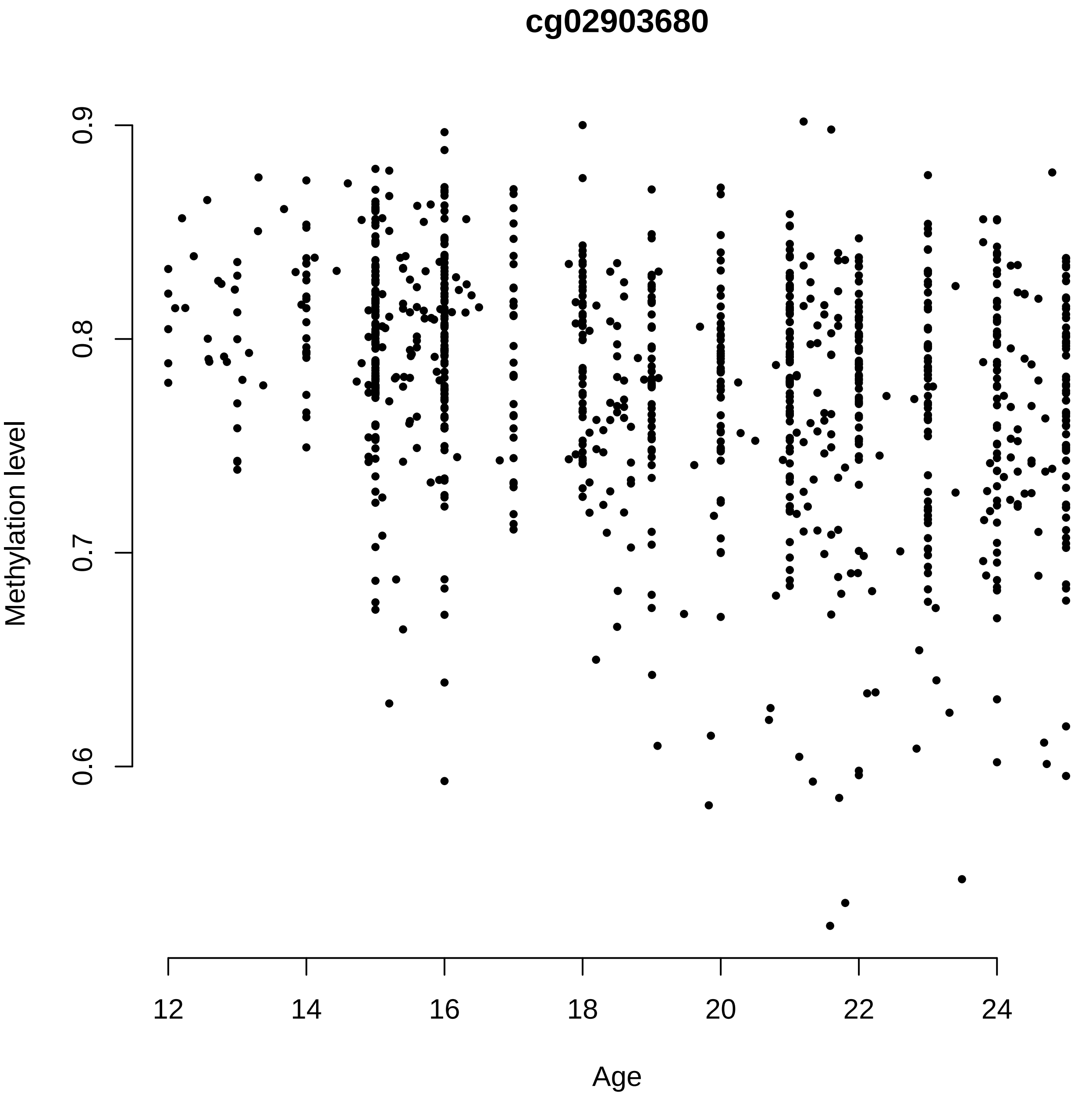
cg02903680
Methylation level
0.6
0.7
0.8
0.9
Age
12
14
16
18
20
22
24

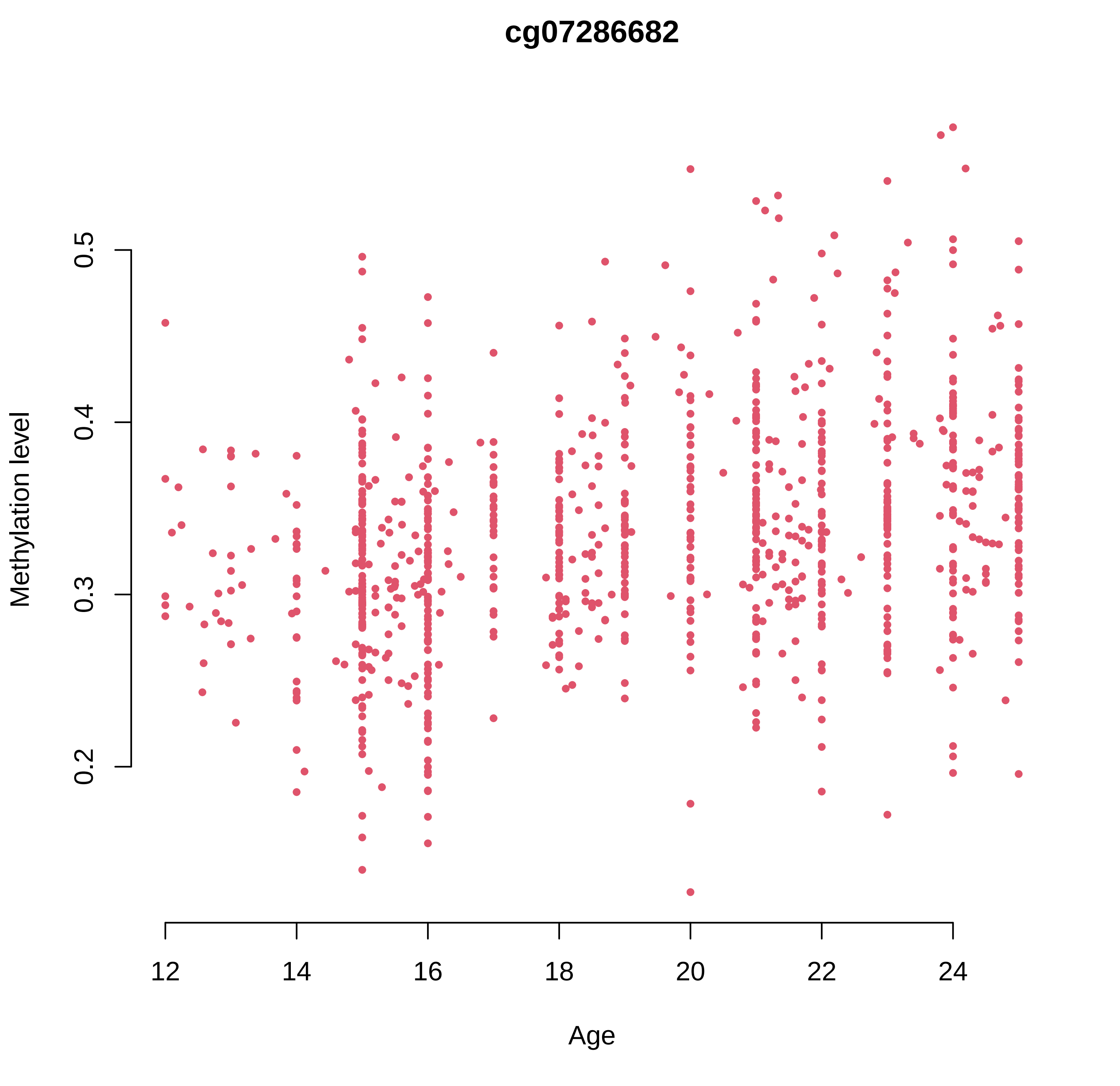

cg07286682
Methylation level
0.2
0.3
0.4
0.5
Age
12
14
16
18
20
22
24

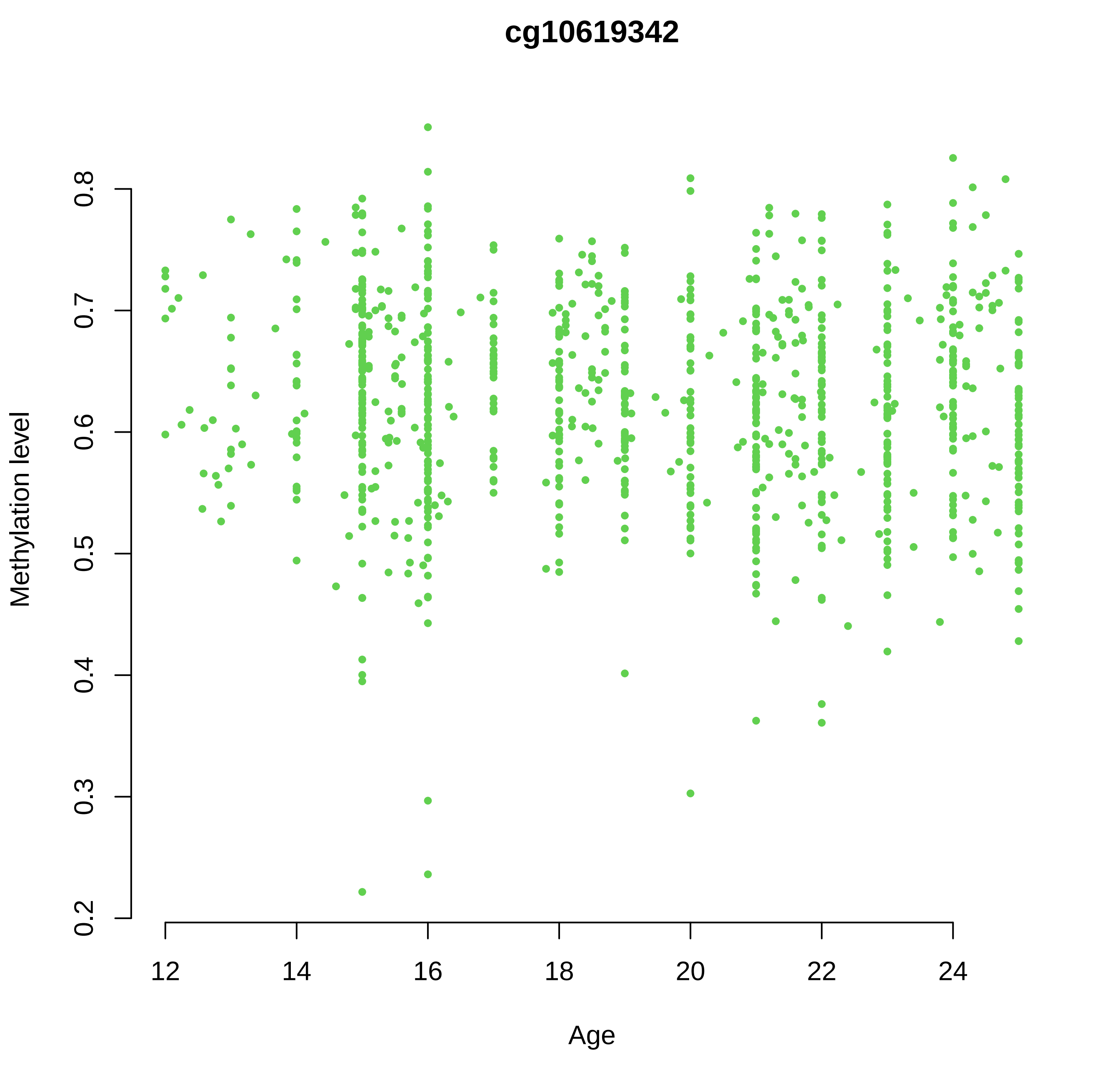

cg10619342
Methylation level
0.2
0.3
0.4
0.5
0.6
0.7
0.8
12
14
16
18
20
22
24
Age

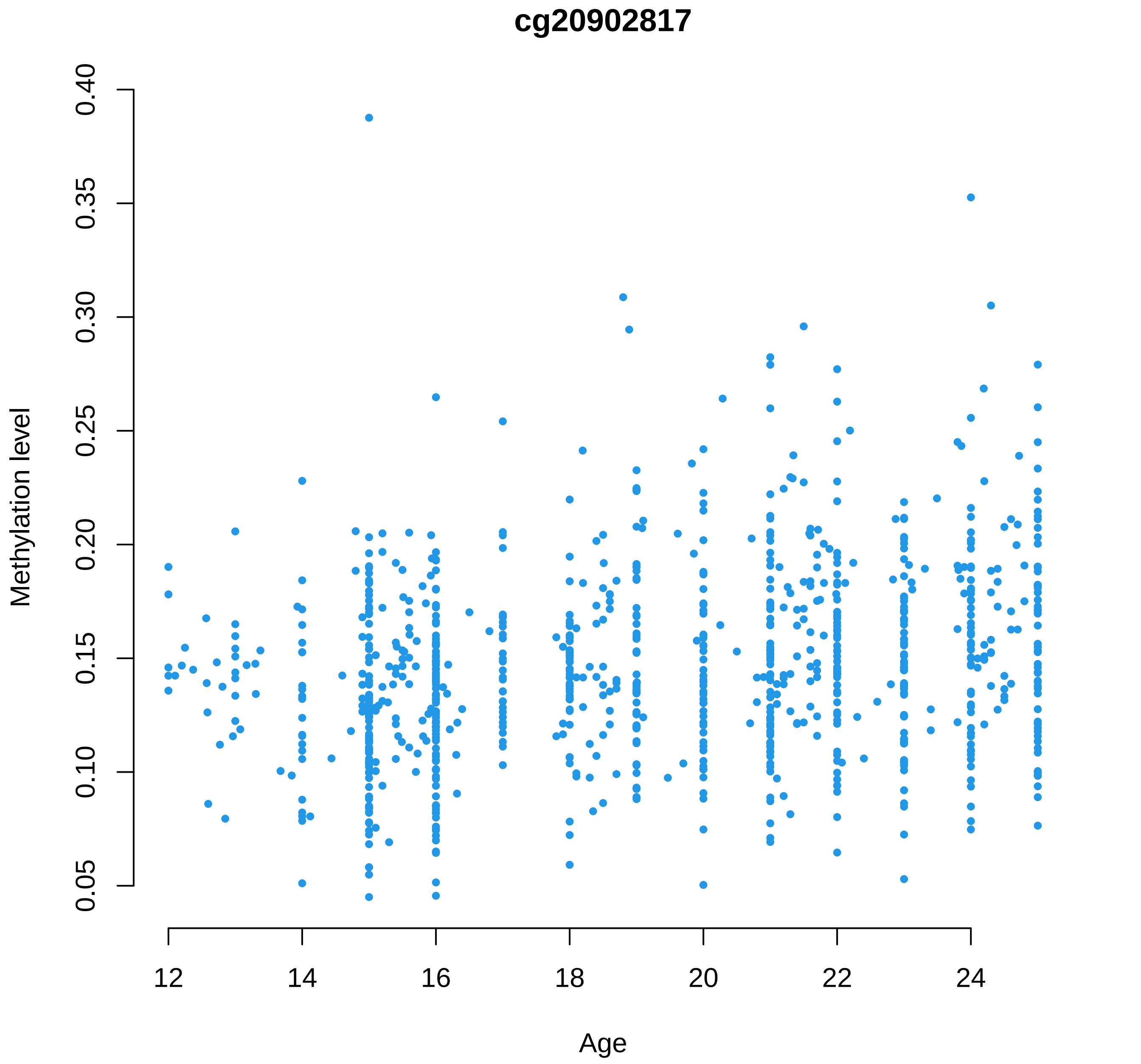

cg20902817
Methylation level
0.05
0.10
0.15
0.20
0.25
0.30
0.35
0.40
12
14
16
18
20
22
24
Age

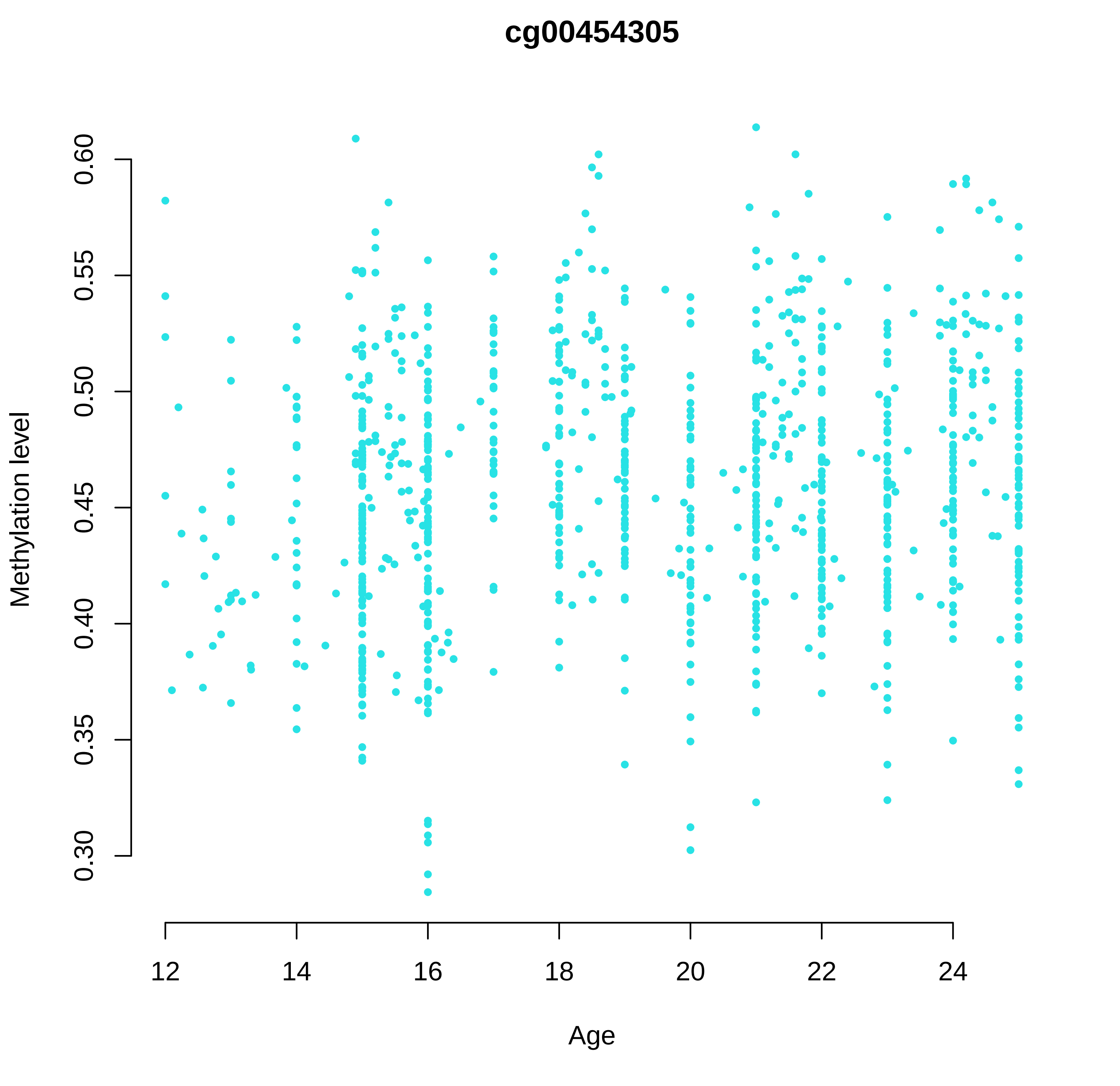
cg00454305
Methylation level
0.30
0.35
0.40
0.45
0.50
0.55
0.60
Age
12
14
16
18
20
22
24

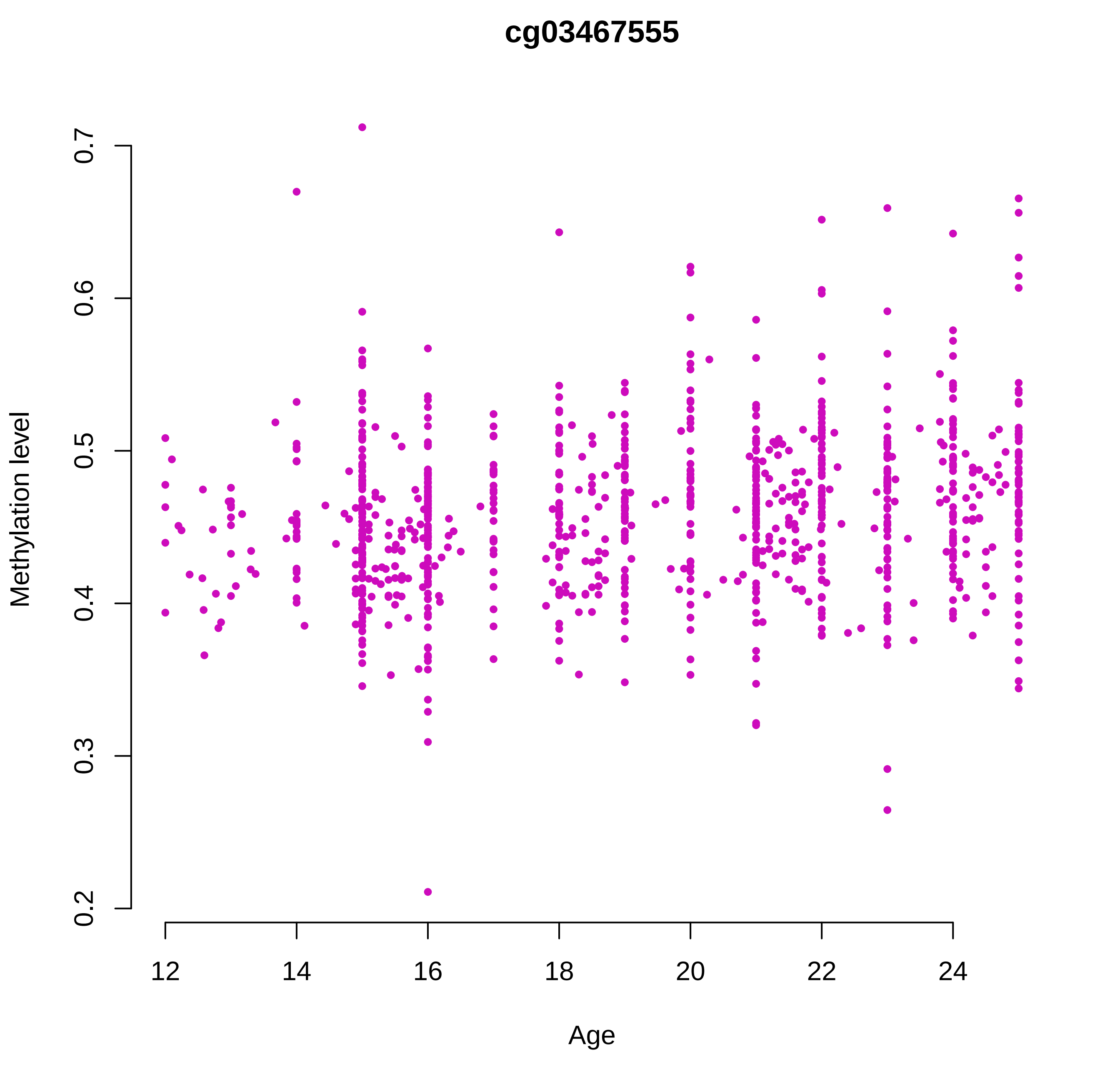
cg03467555
Methylation level
0.2
0.3
0.4
0.5
0.6
0.7
Age
12
14
16
18
20
22
24

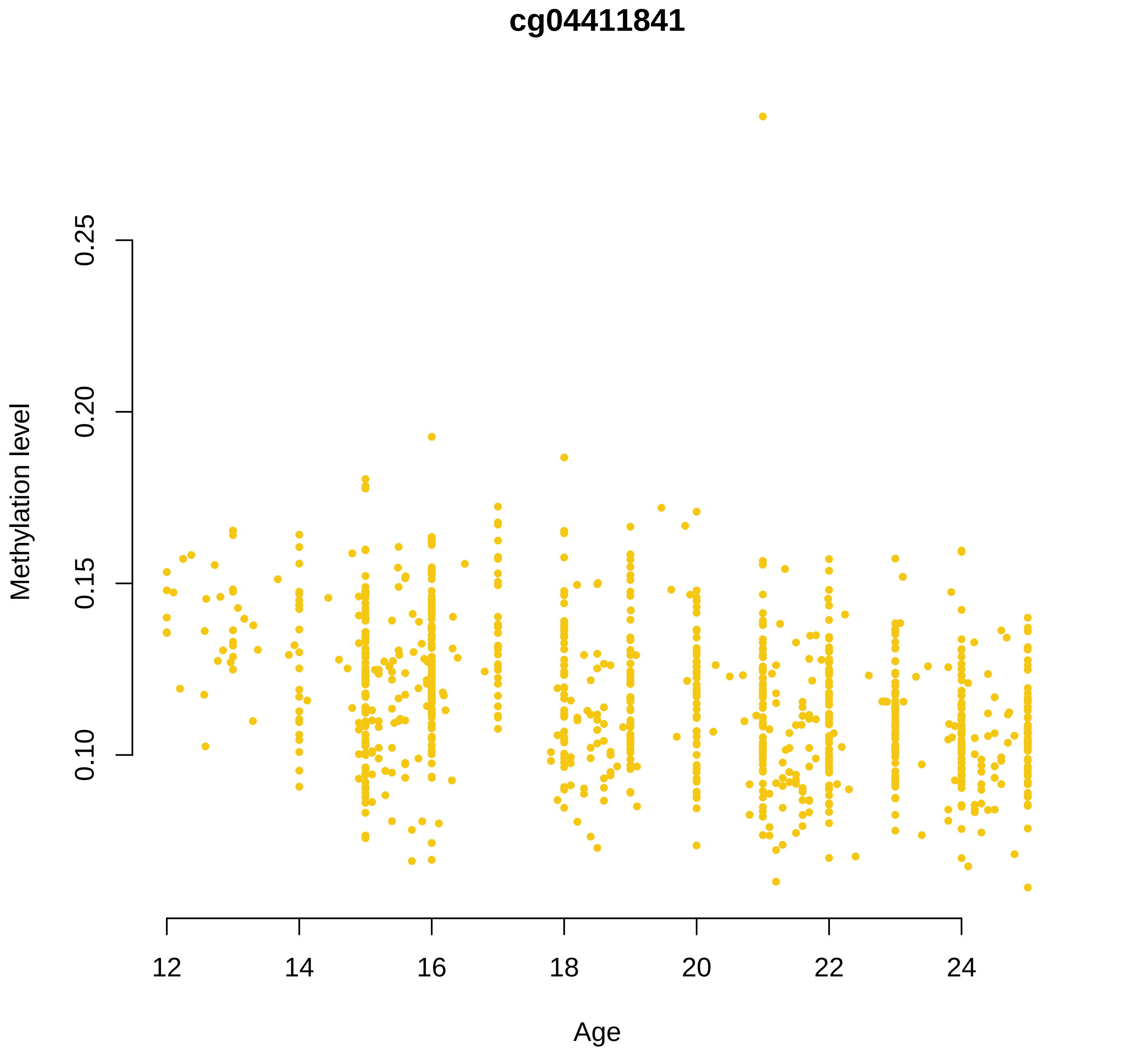
cg04411841
Methylation level
0.10
0.15
0.20
0.25
12
14
16
18
20
22
24
Age

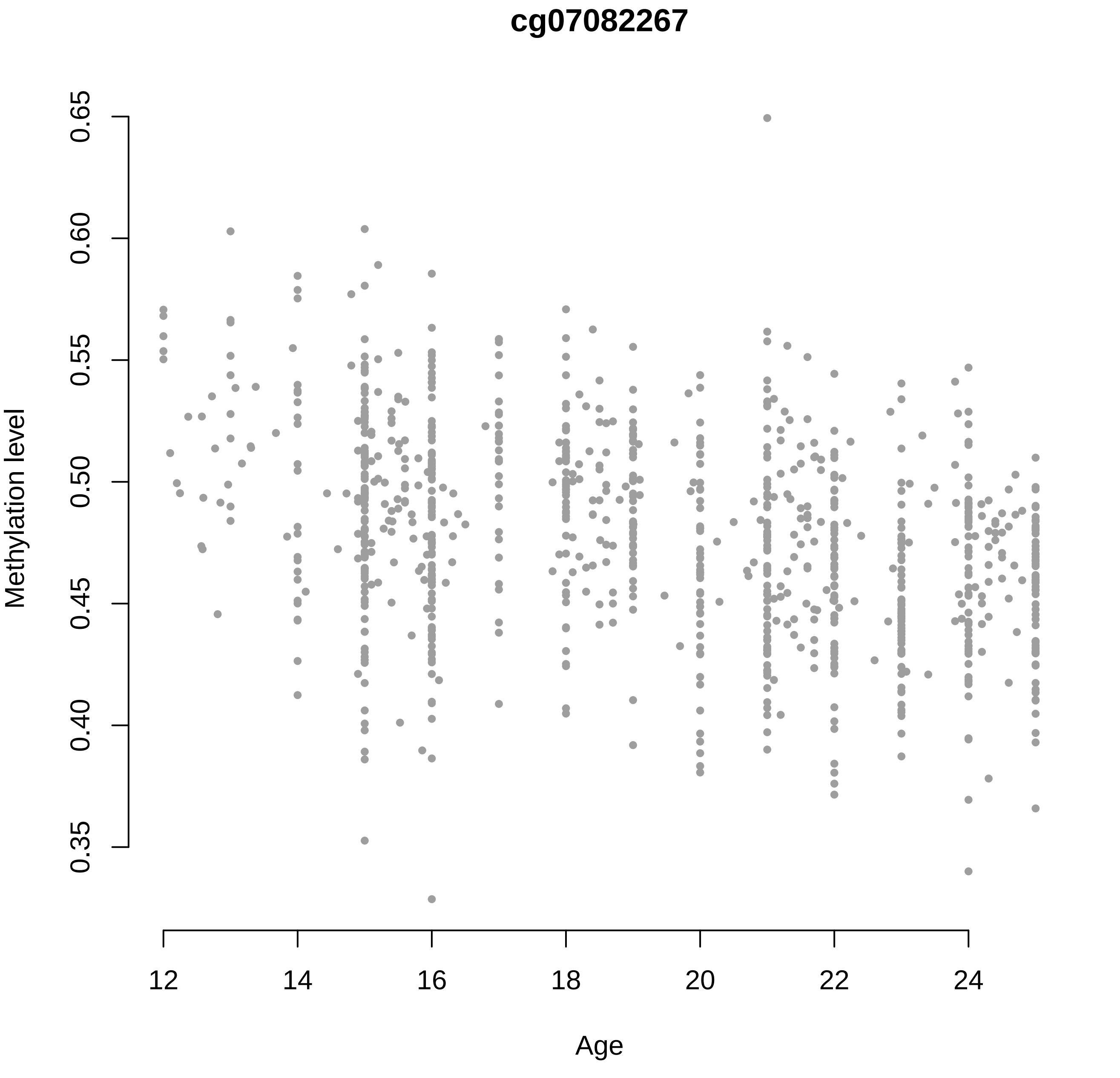
cg07082267
Methylation level
0.35
0.40
0.45
0.50
0.55
0.60
0.65
Age
12
14
16
18
20
22
24

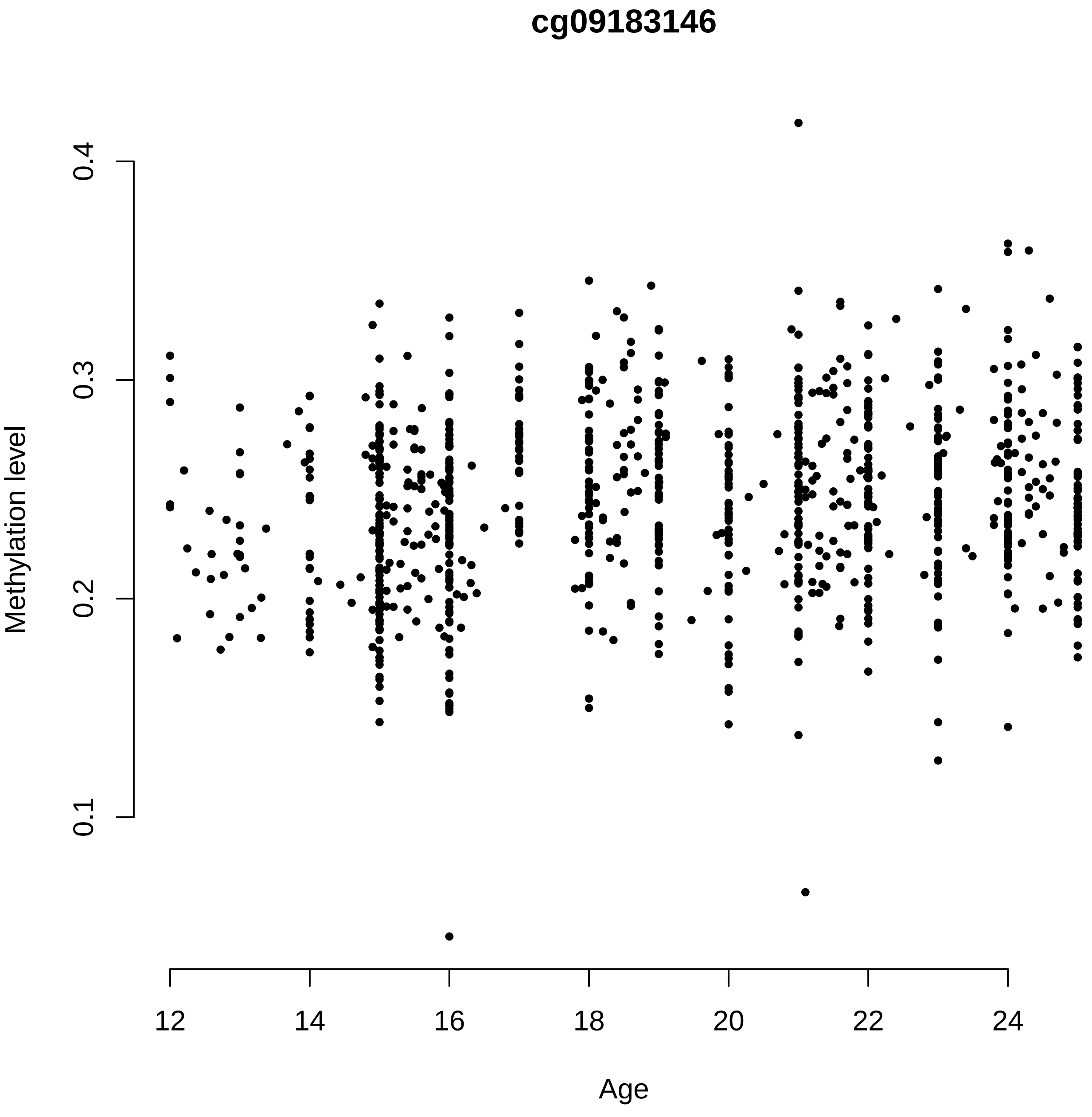
cg09183146
Methylation level
0.1
0.2
0.3
0.4
Age
12
14
16
18
20
22
24

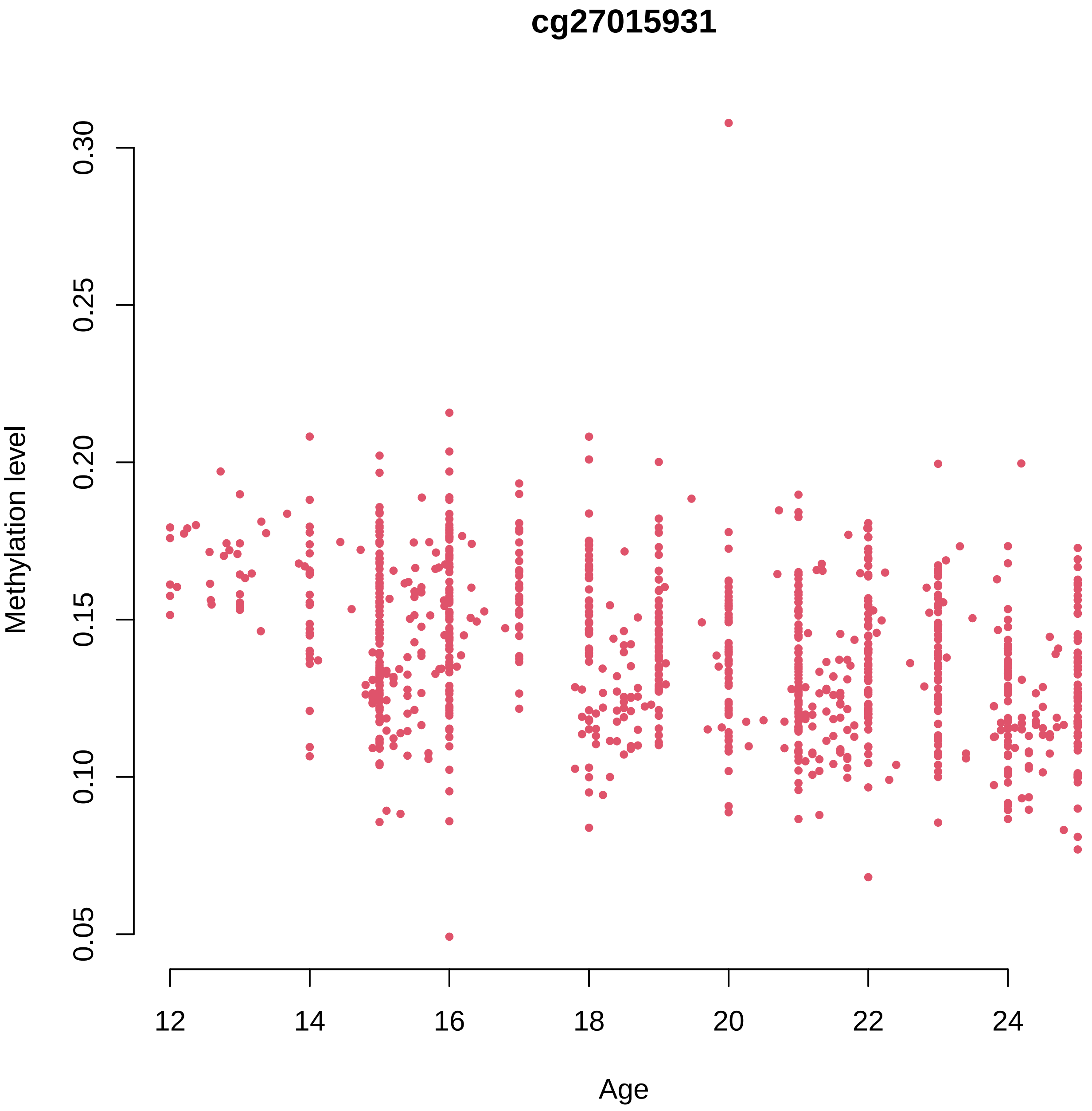
cg27015931
Methylation level
0.05
0.10
0.15
0.20
0.25
0.30
Age
12
14
16
18
20
22
24

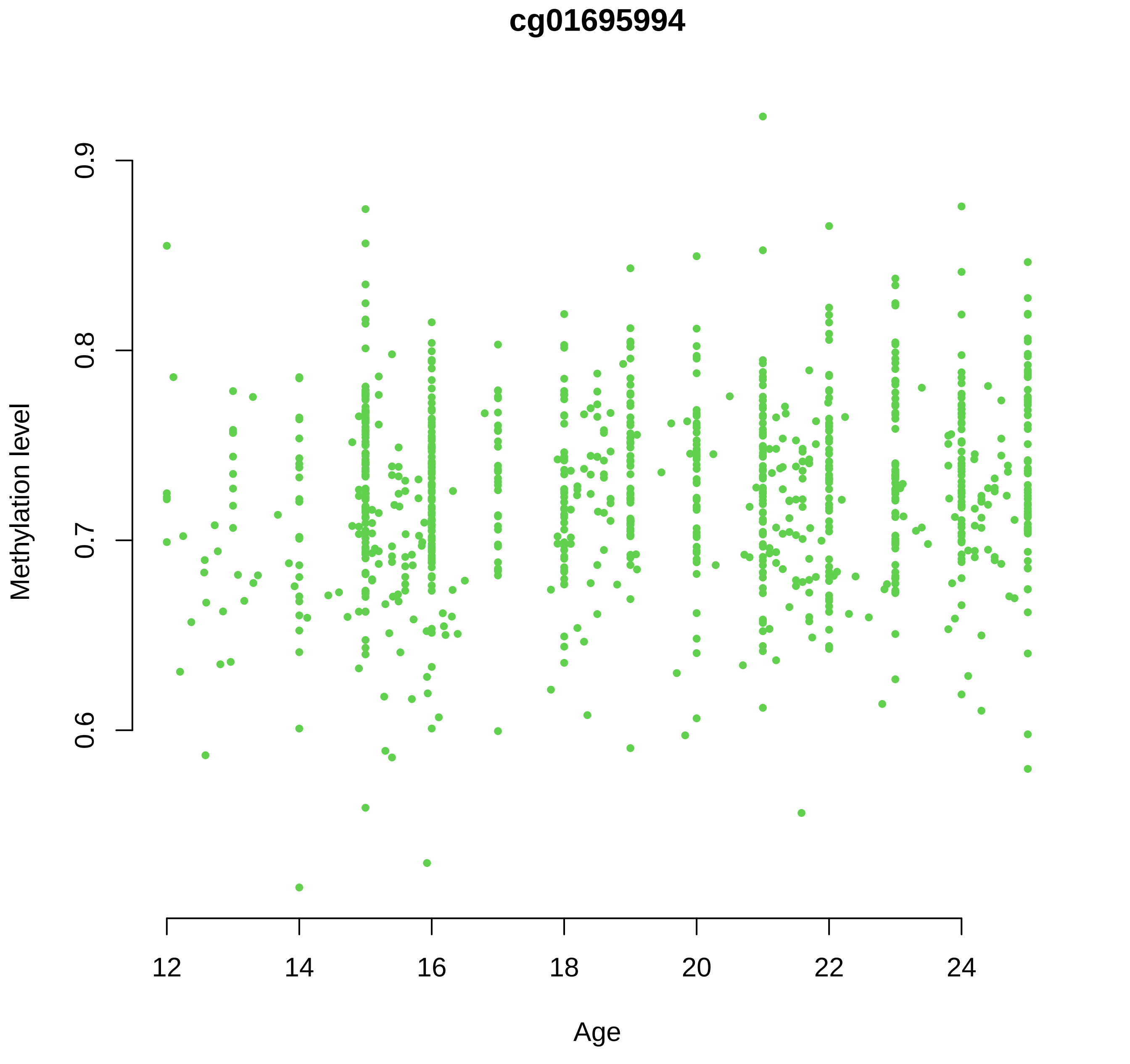
cg01695994
Methylation level
0.6
0.7
0.8
0.9
Age
12
14
16
18
20
22
24

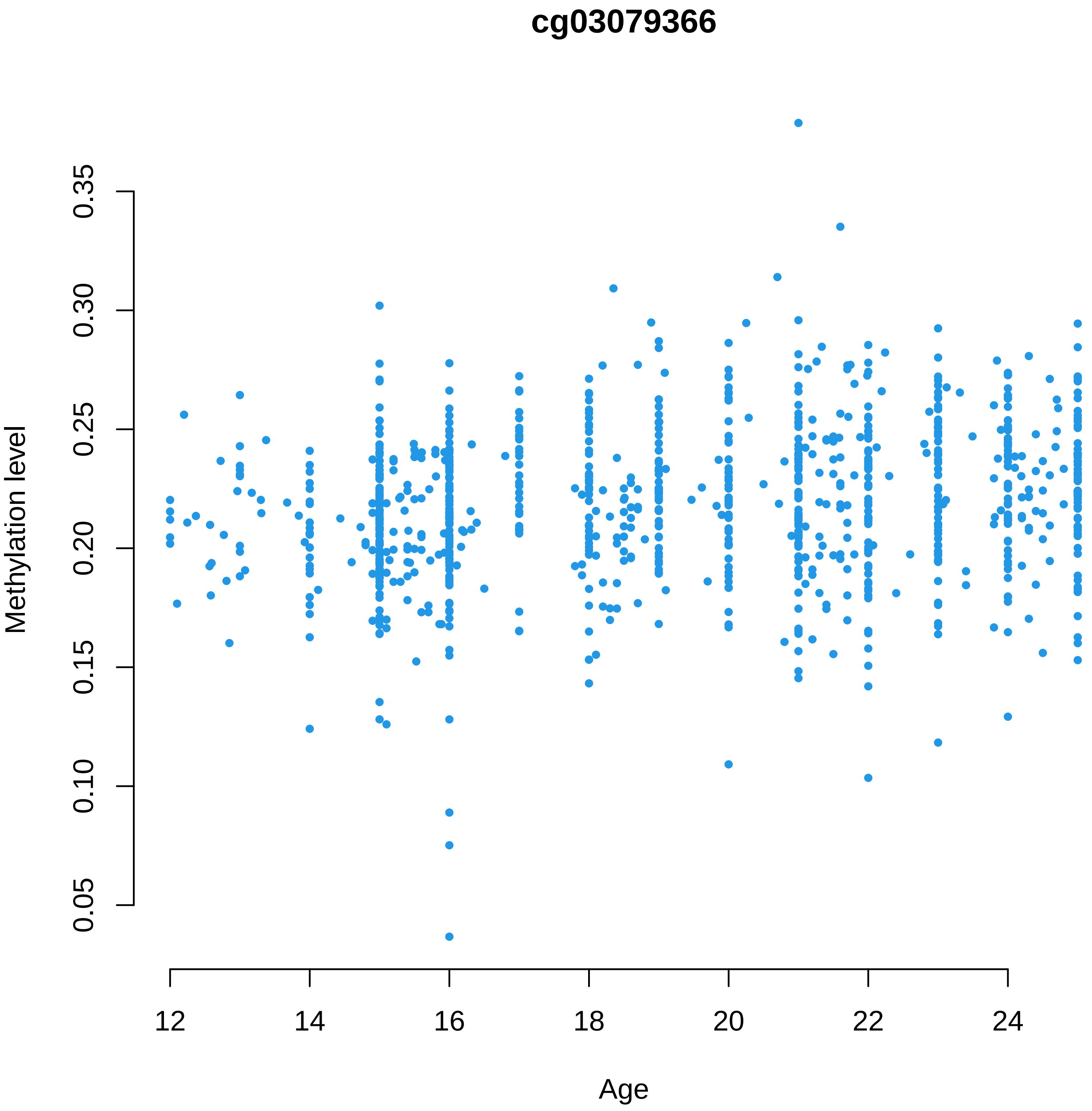
cg03079366
Methylation level
0.05
0.10
0.15
0.20
0.25
0.30
0.35
Age
12
14
16
18
20
22
24

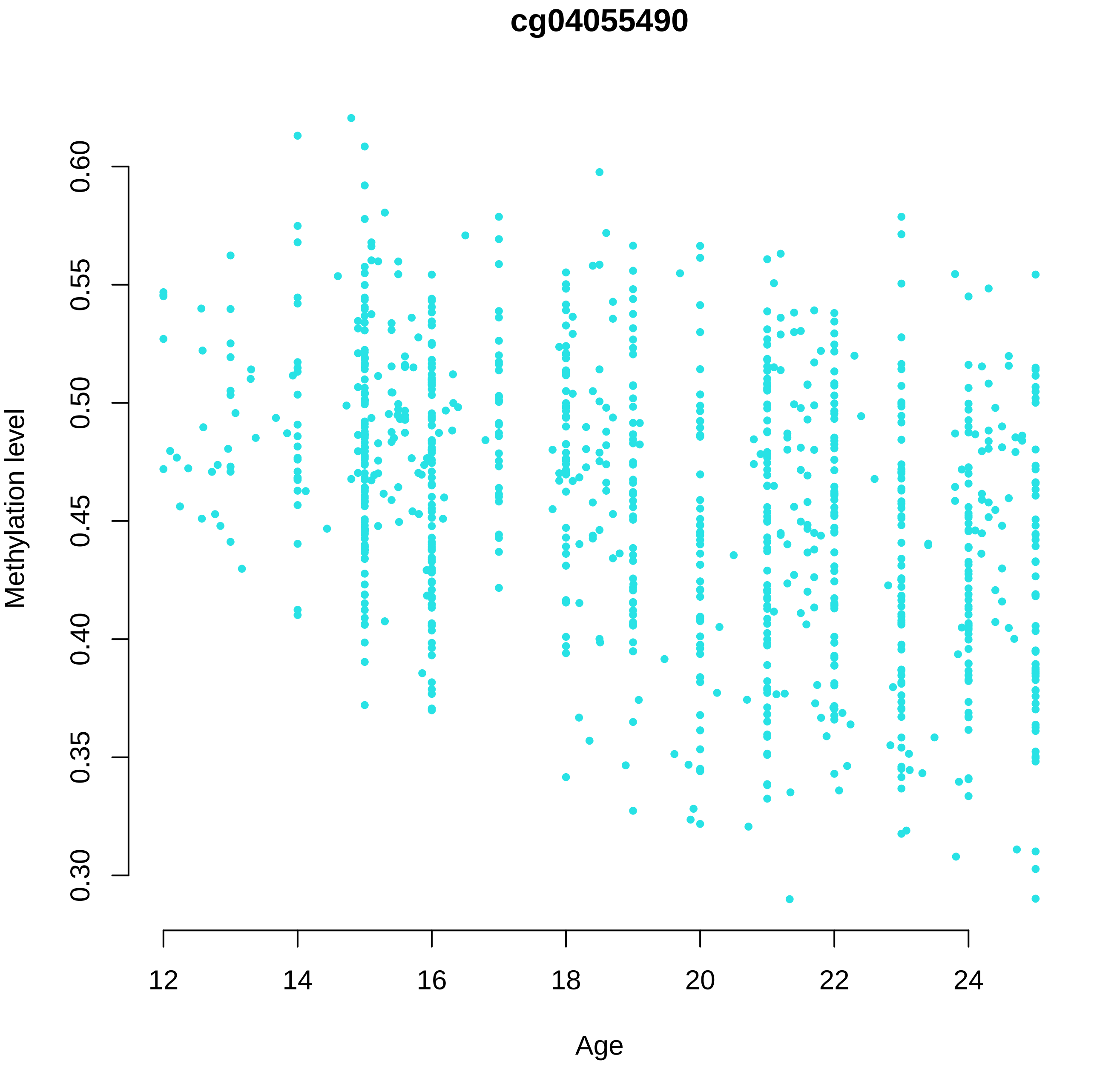

cg04055490
Methylation level
0.30
0.35
0.40
0.45
0.50
0.55
0.60
Age
12
14
16
18
20
22
24

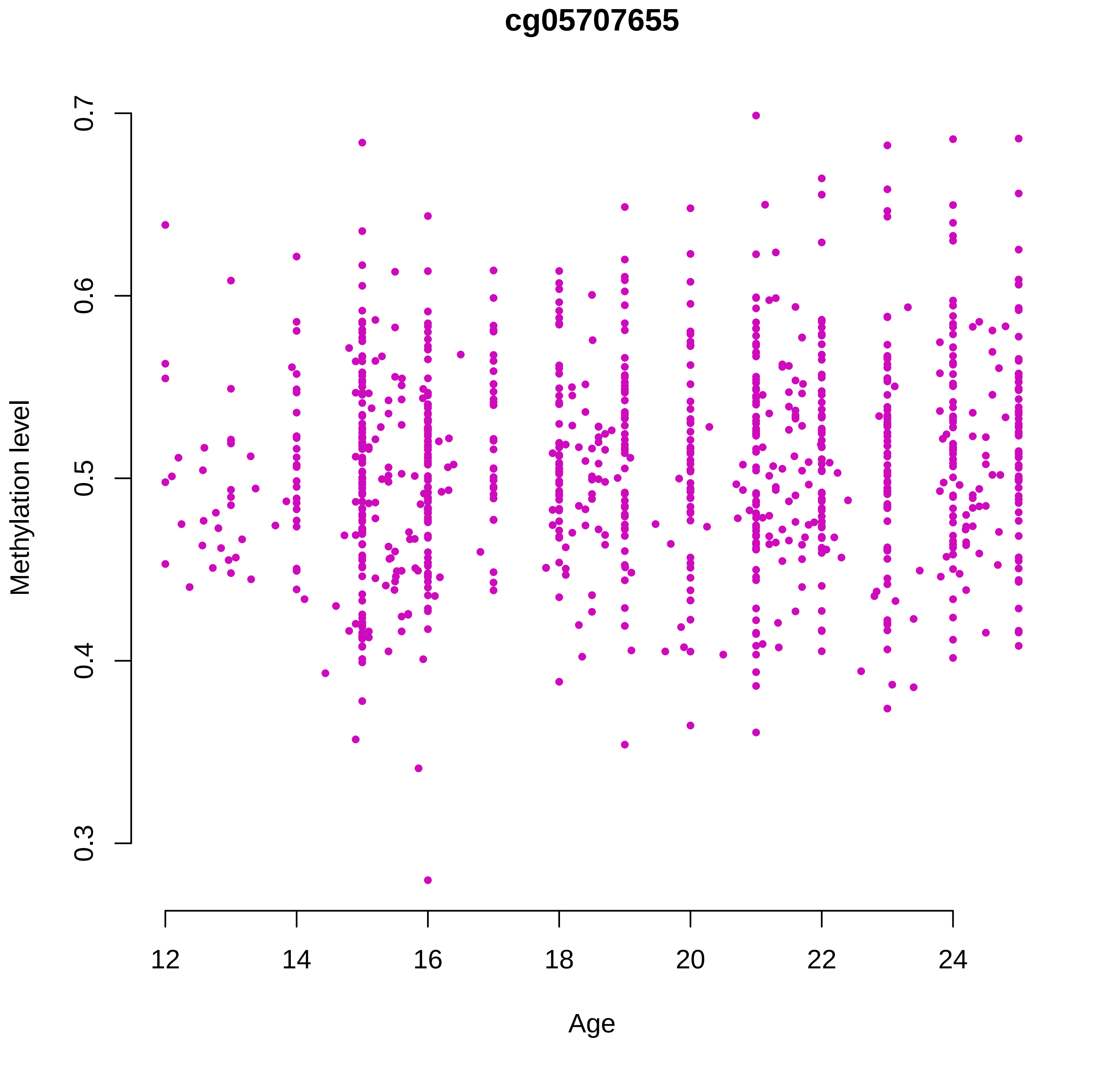
cg05707655
Methylation level
0.3
0.4
0.5
0.6
0.7
12
14
16
18
20
22
24
Age

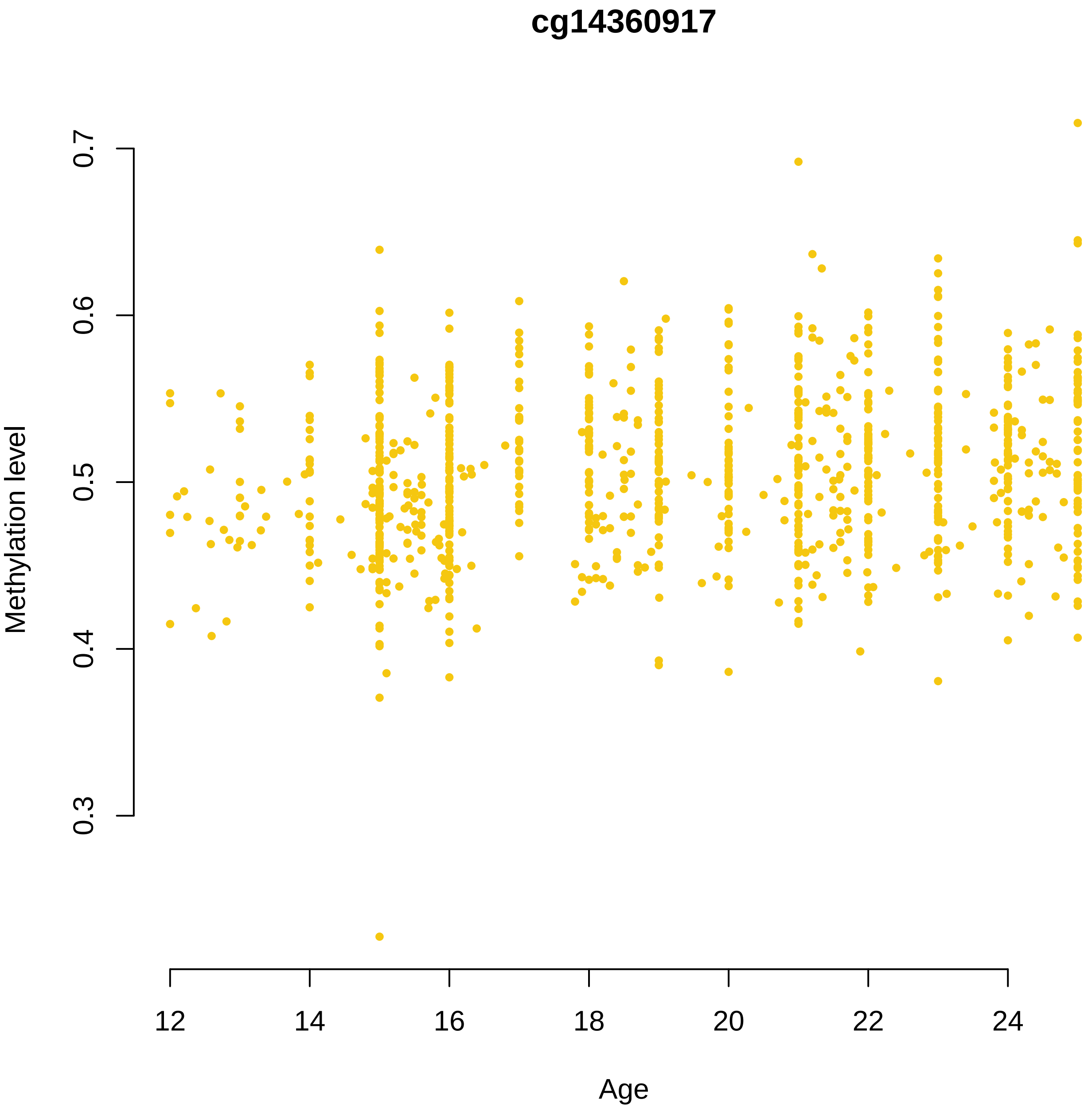

cg14360917
Methylation level
0.3
0.4
0.5
0.6
0.7
Age
12
14
16
18
20
22
24

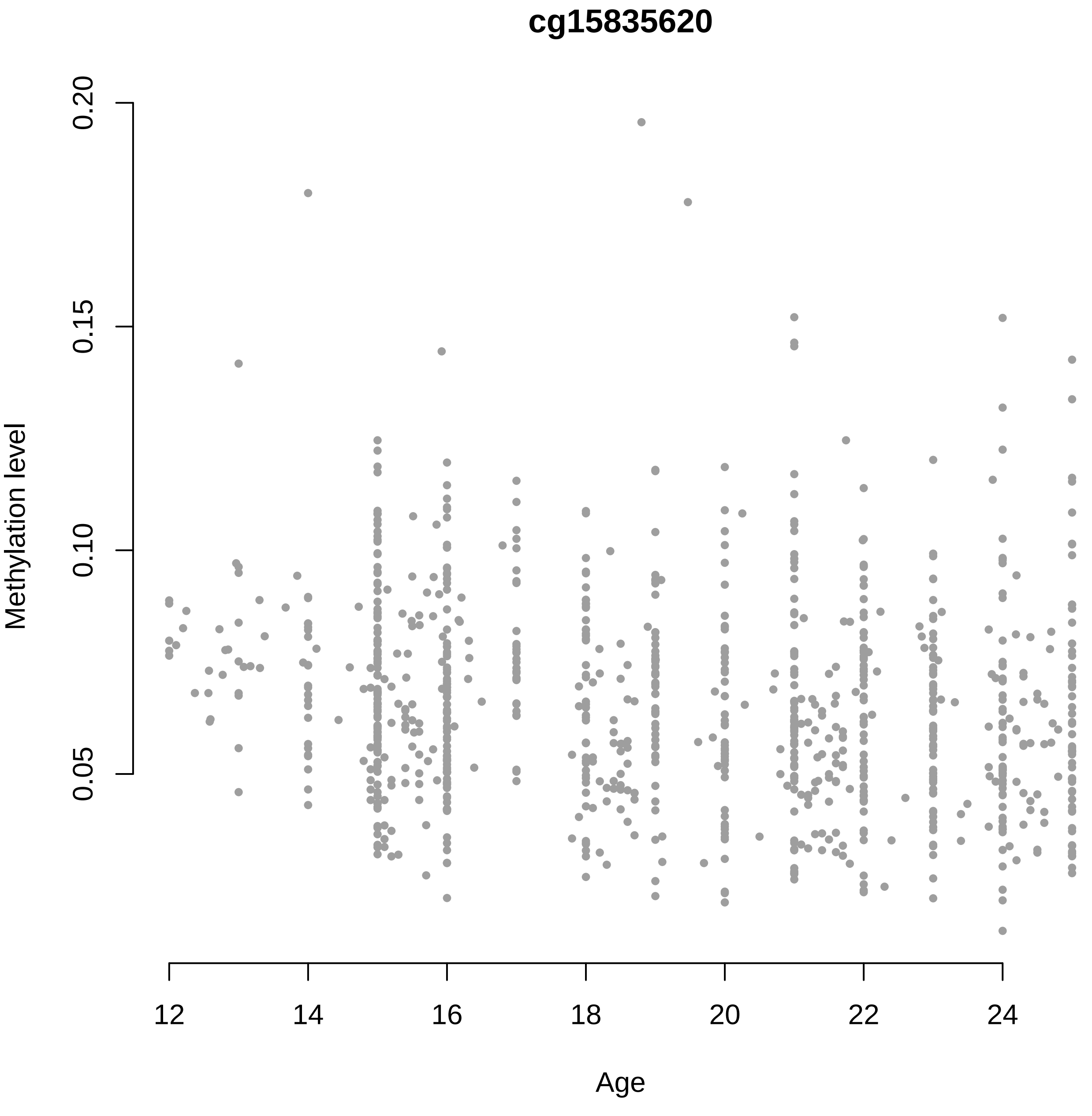
cg15835620
Methylation level
0.05
0.10
0.15
0.20
Age
12
14
16
18
20
22
24

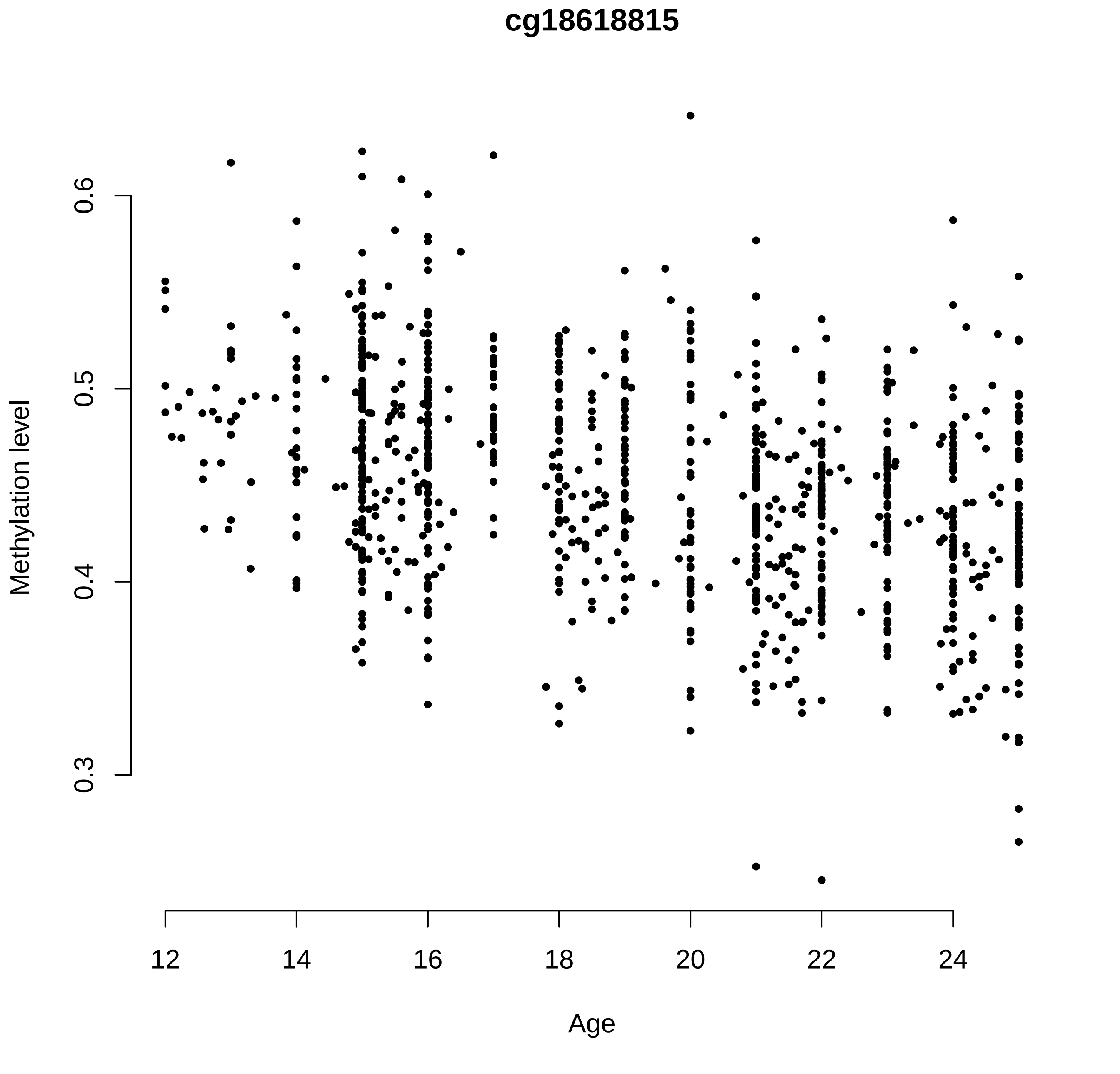
cg18618815
Methylation level
0.3
0.4
0.5
0.6
12
14
16
18
20
22
24
Age

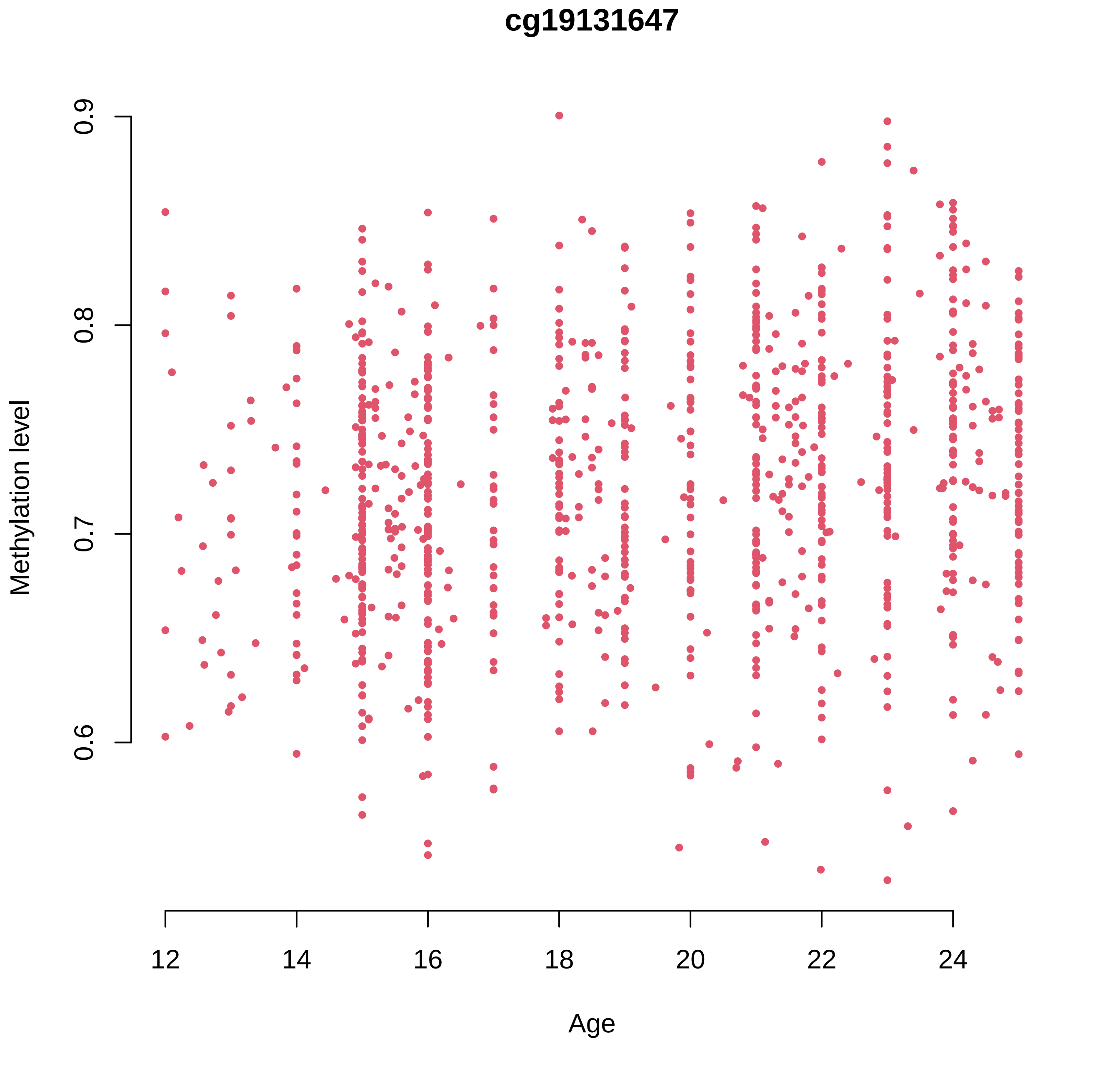
cg19131647
Methylation level
0.6
0.7
0.8
0.9
Age
12
14
16
18
20
22
24

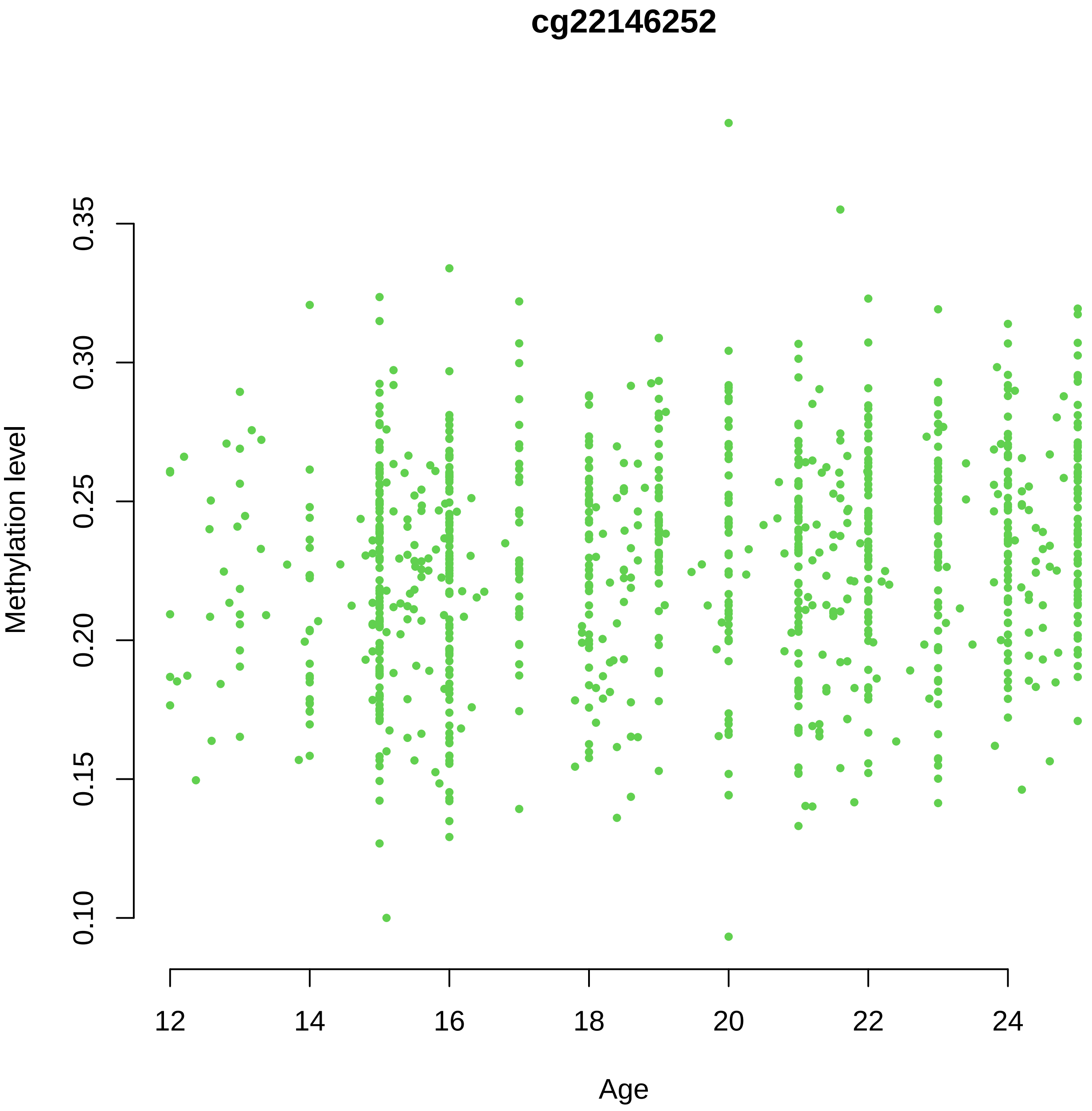
cg22146252
Methylation level
0.10
0.15
0.20
0.25
0.30
0.35
Age
12
14
16
18
20
22
24

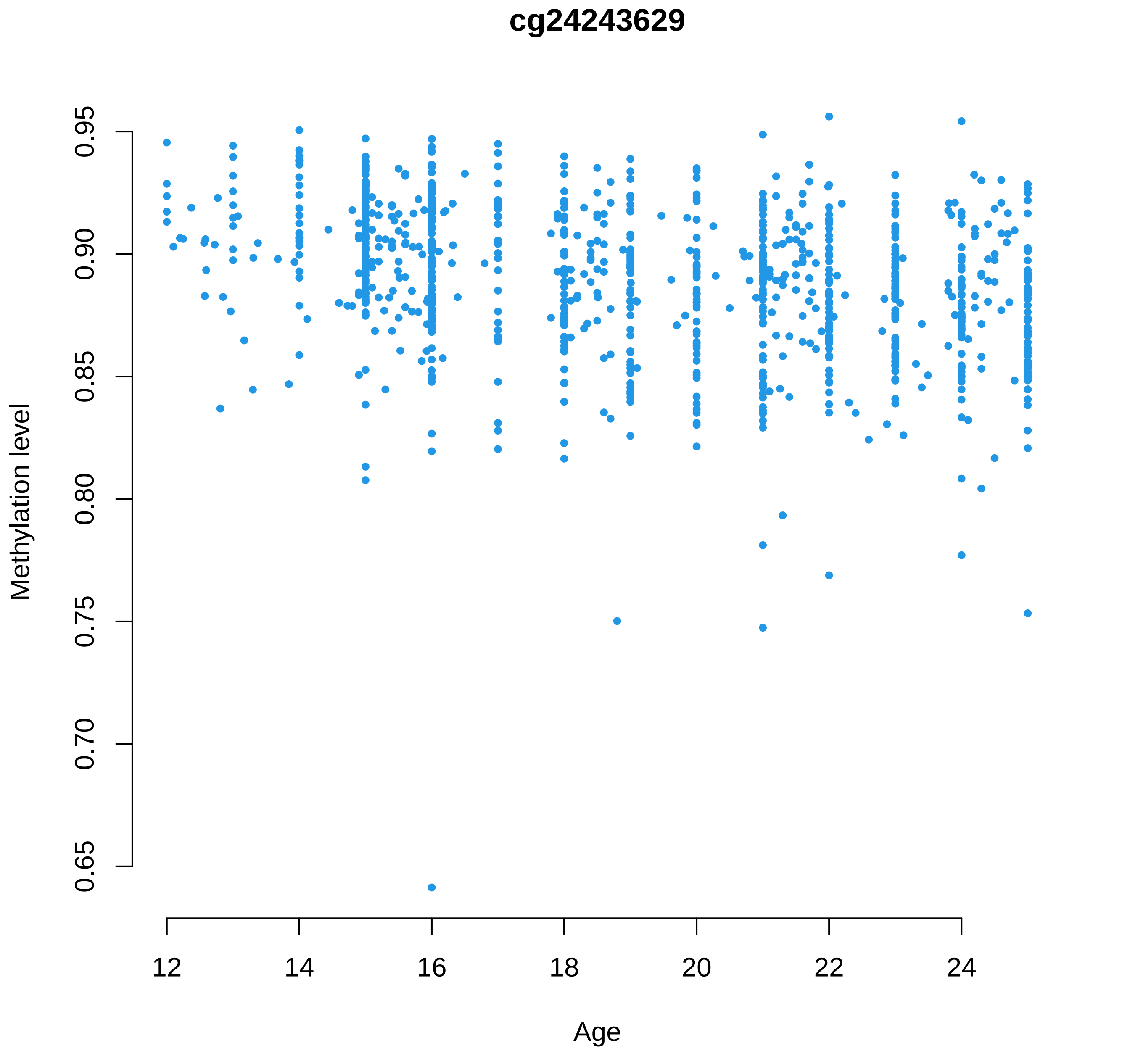

cg24243629
Methylation level
0.95
0.90
0.85
0.80
0.75
0.70
0.65
12
14
16
18
20
22
24
Age

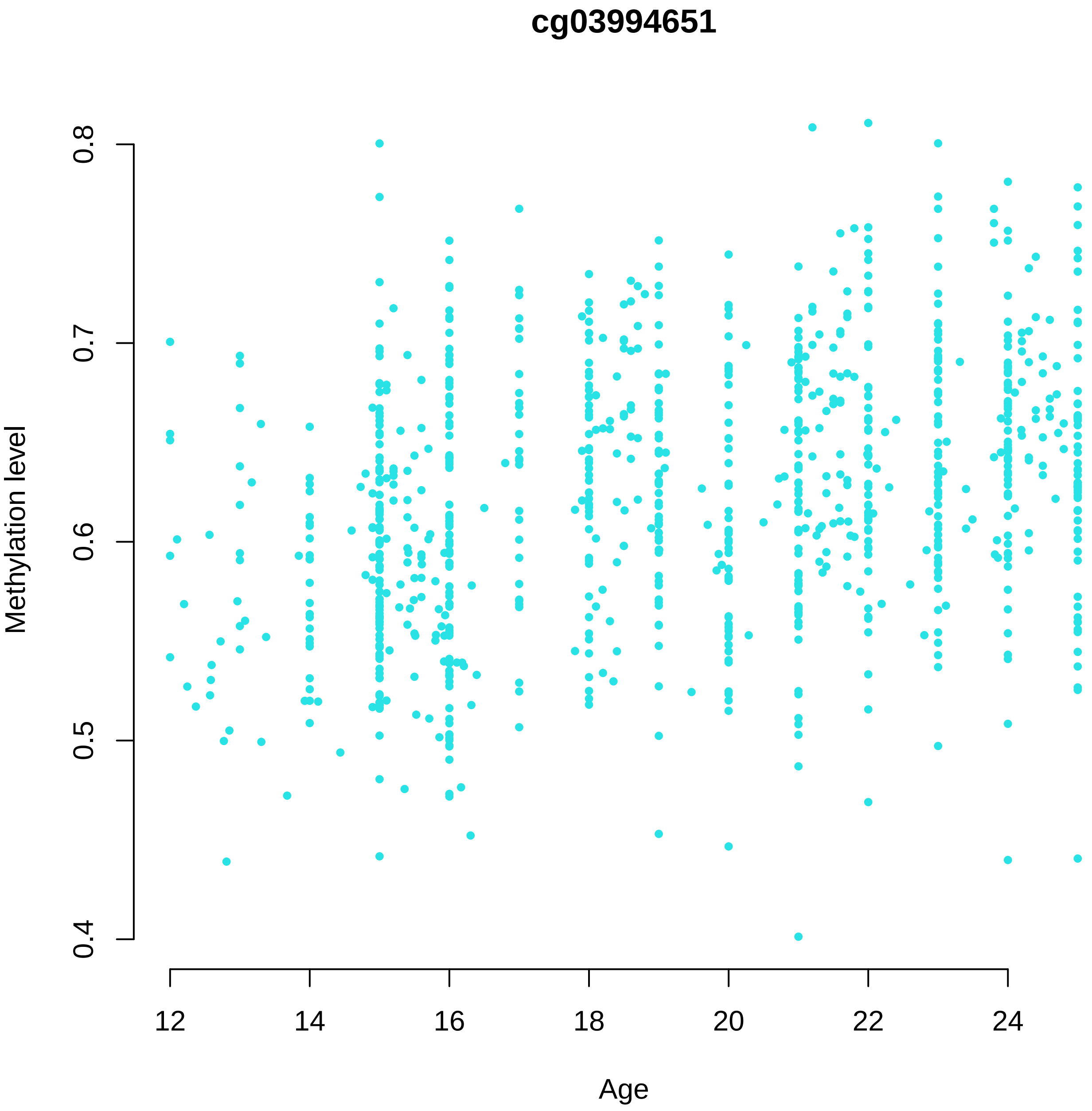
cg03994651
Methylation level
0.4
0.5
0.6
0.7
0.8
Age
12
14
16
18
20
22
24

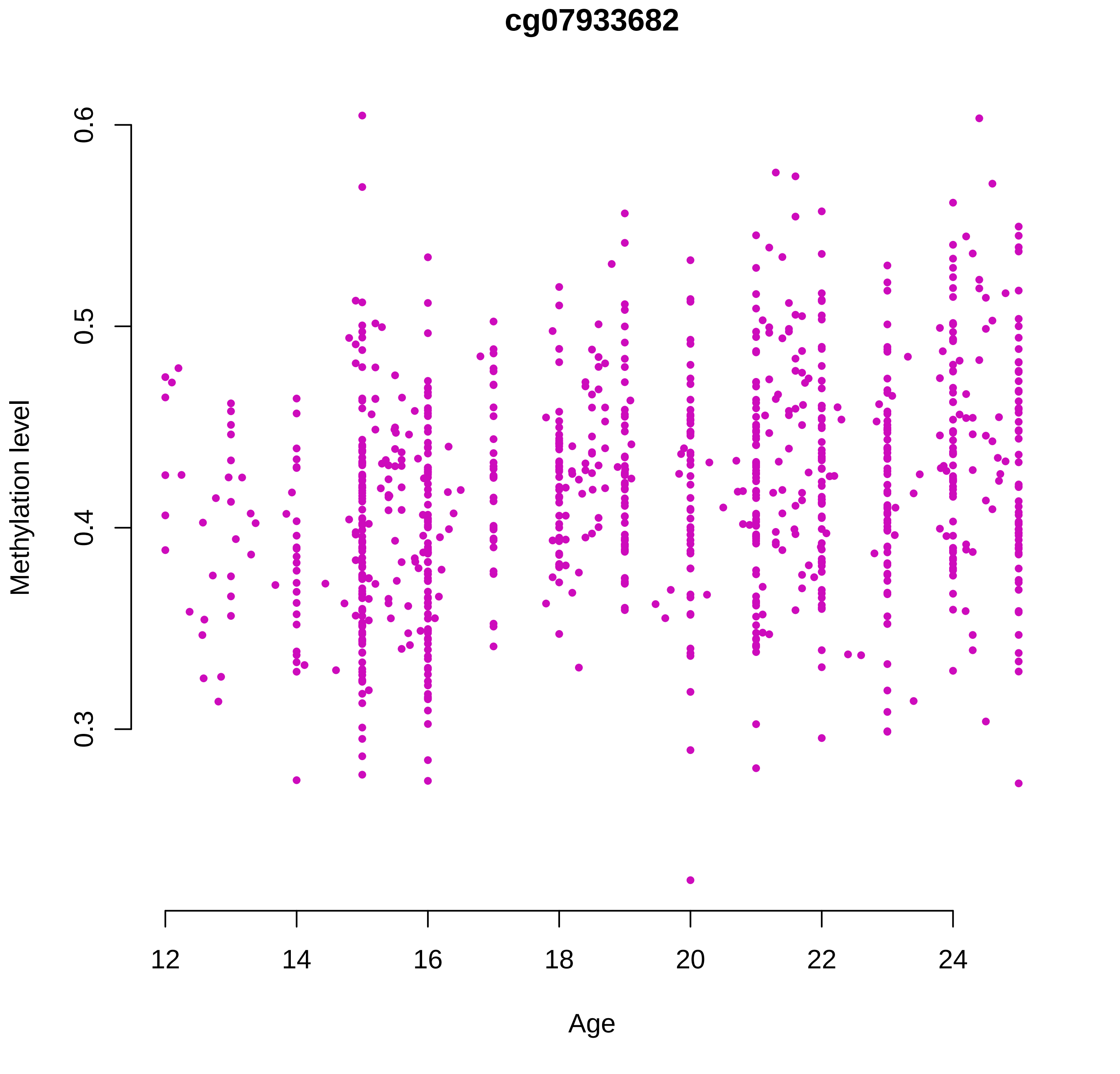
cg07933682
Methylation level
0.3
0.4
0.5
0.6
12
14
16
18
20
22
24
Age

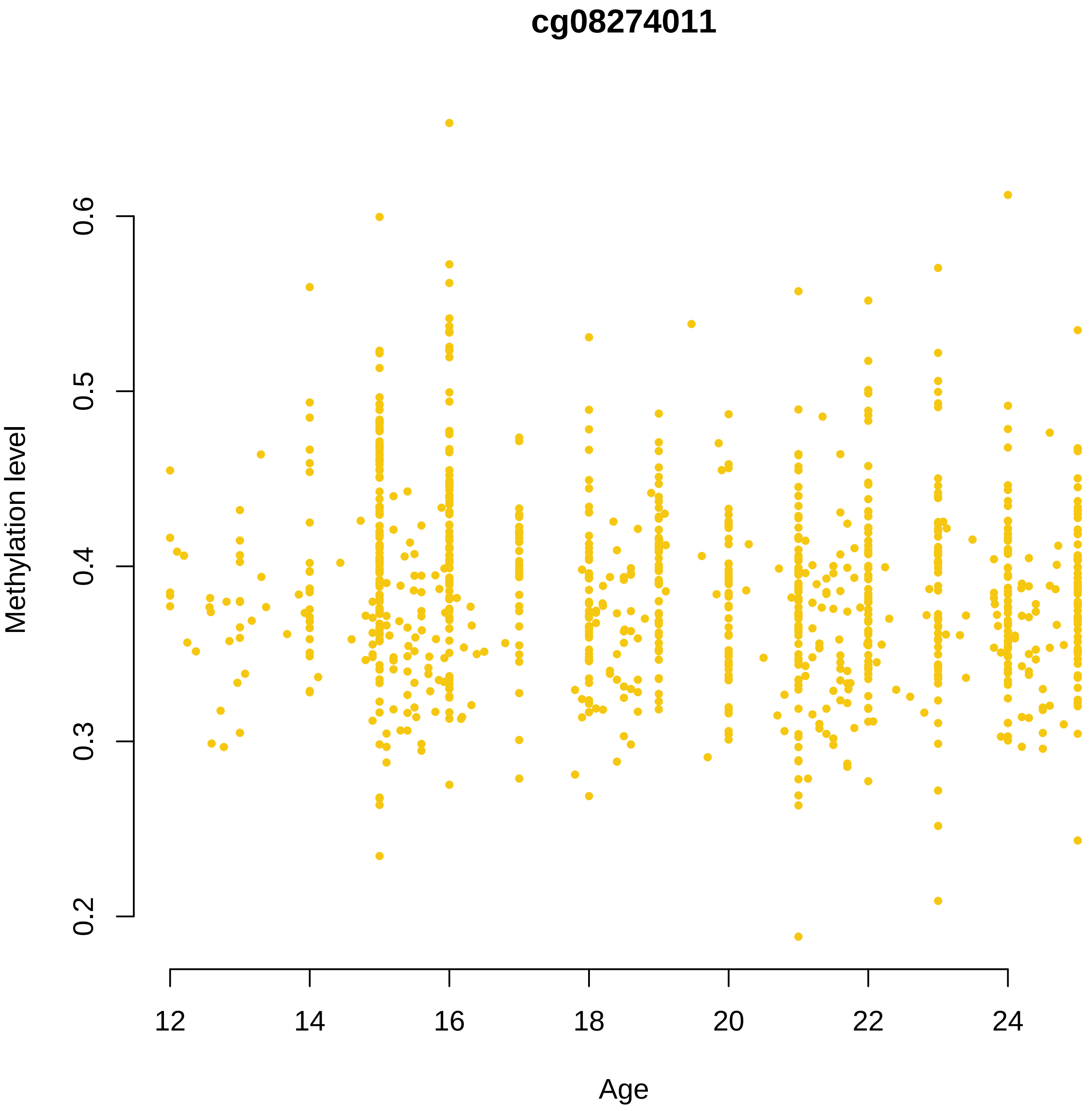

cg08274011
Methylation level
0.2
0.3
0.4
0.5
0.6
Age
12
14
16
18
20
22
24

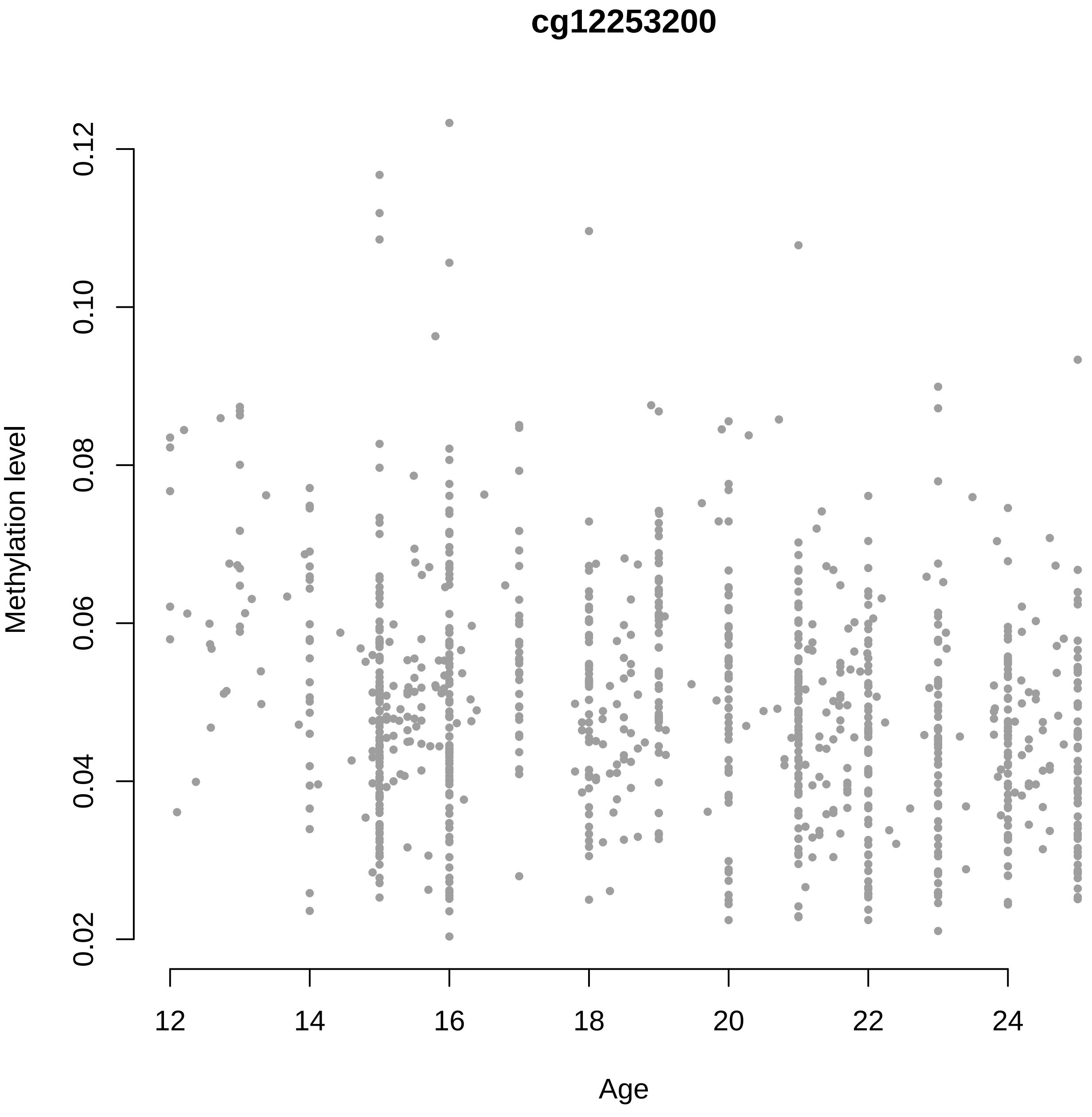
cg12253200
Methylation level
0.02
0.04
0.06
0.08
0.10
0.12
12
14
16
18
20
22
24
Age

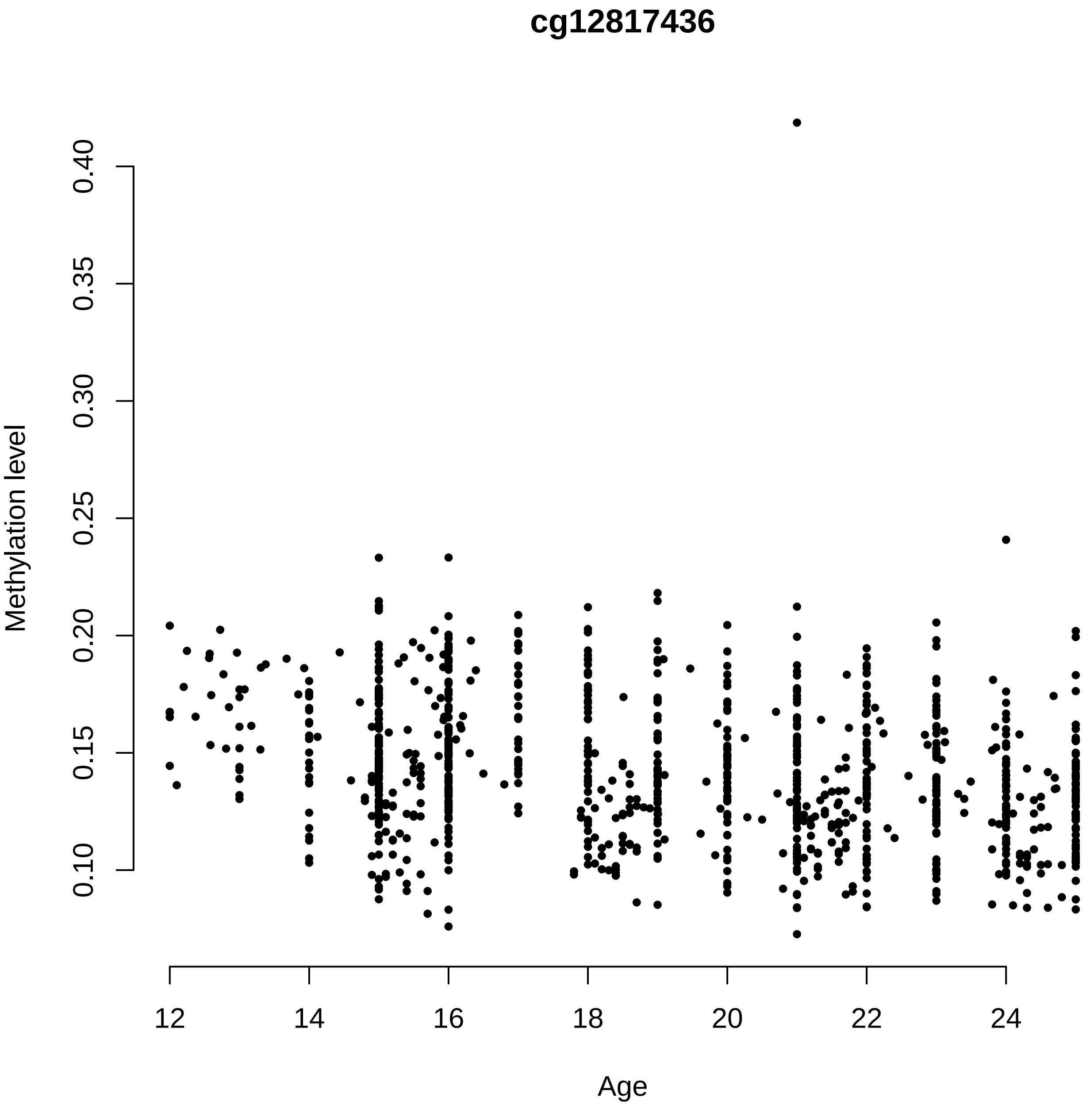

cg12817436
Methylation level
0.10
0.15
0.20
0.25
0.30
0.35
0.40
Age
12
14
16
18
20
22
24

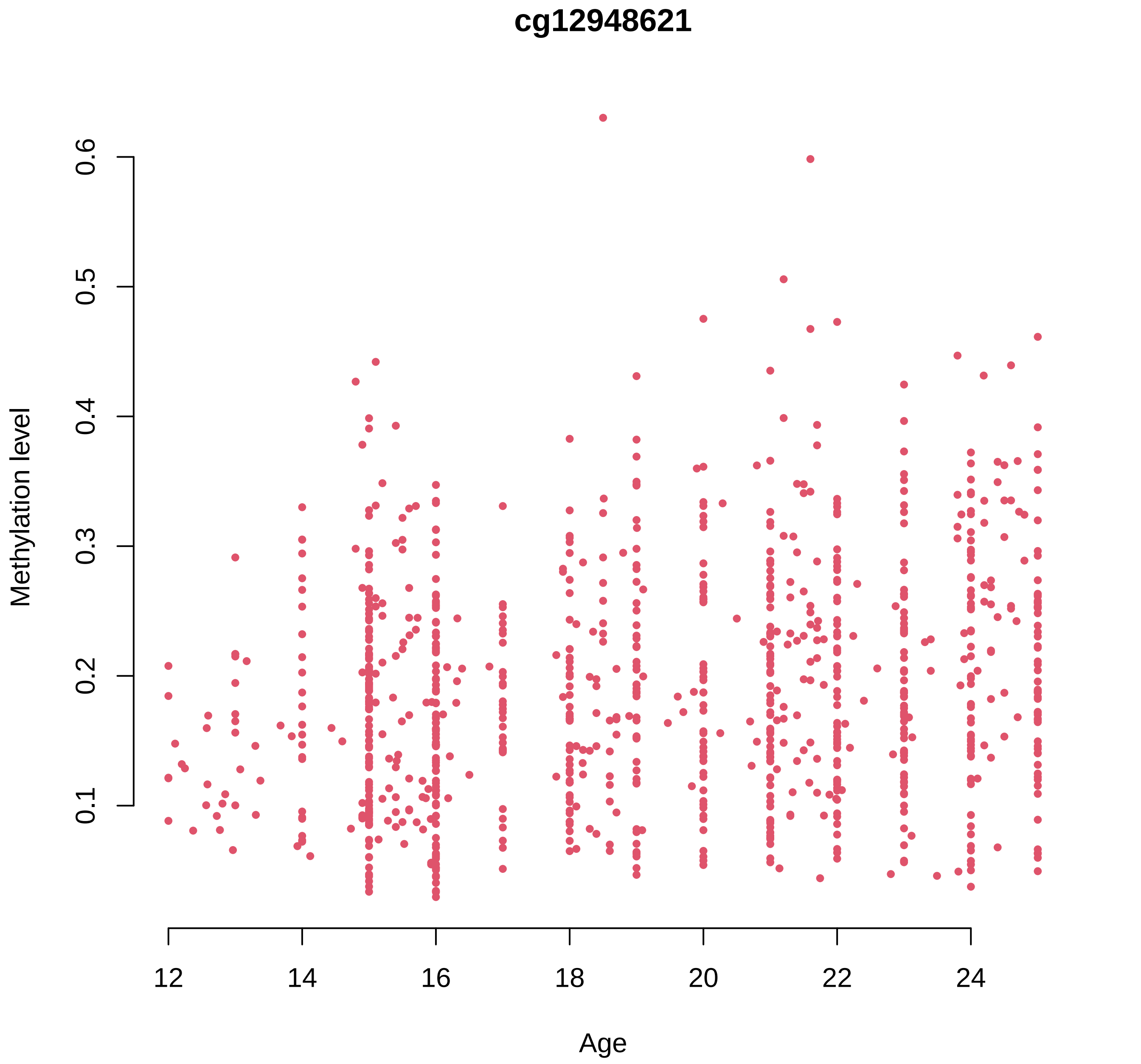
cg12948621
Methylation level
0.1
0.2
0.3
0.4
0.5
0.6
Age
12
14
16
18
20
22
24

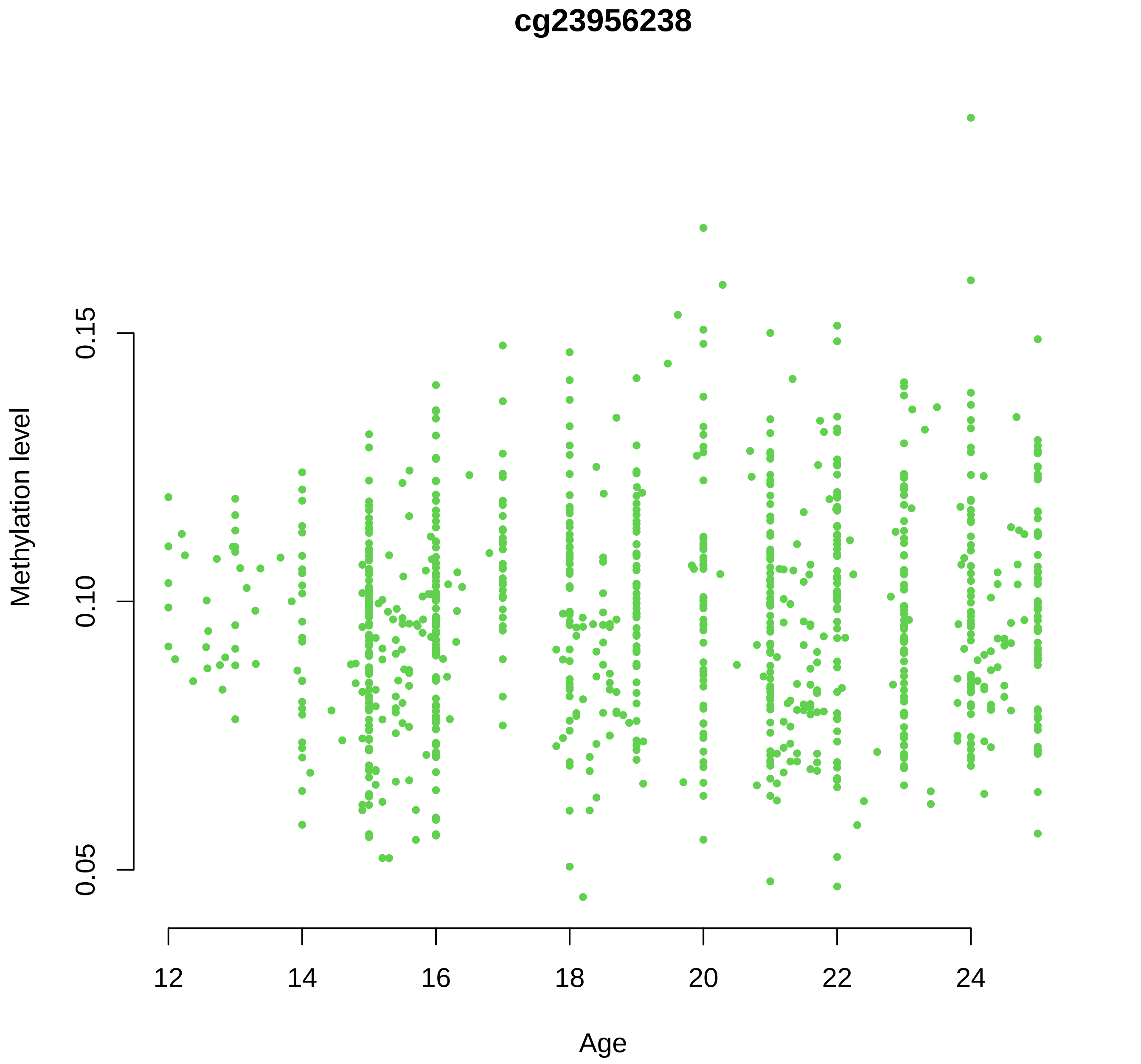

cg23956238
Methylation level
0.05
0.10
0.15
12
14
16
18
20
22
24
Age

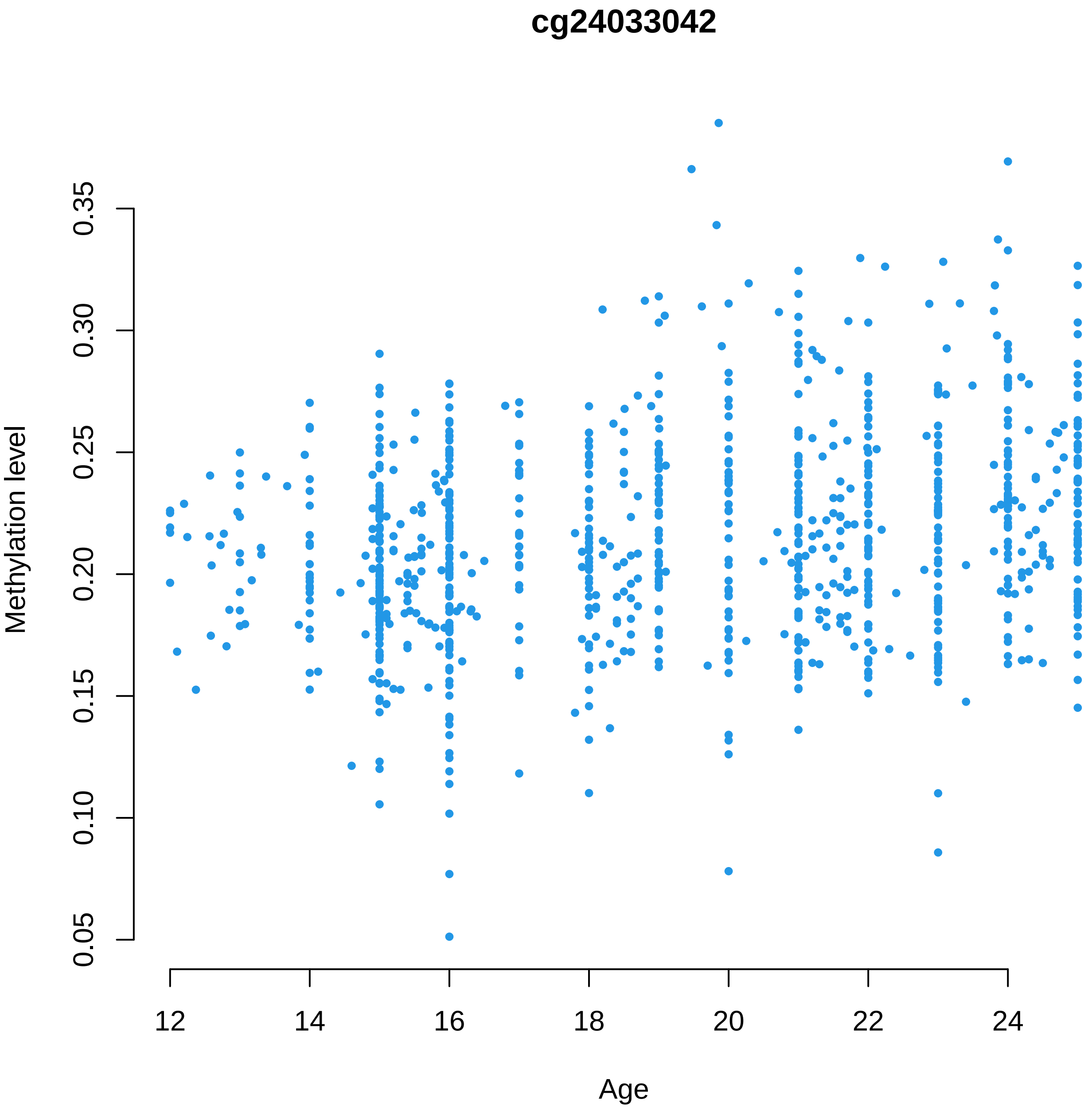
cg24033042
Methylation level
0.05
0.10
0.15
0.20
0.25
0.30
0.35
12
14
16
18
20
22
24
Age

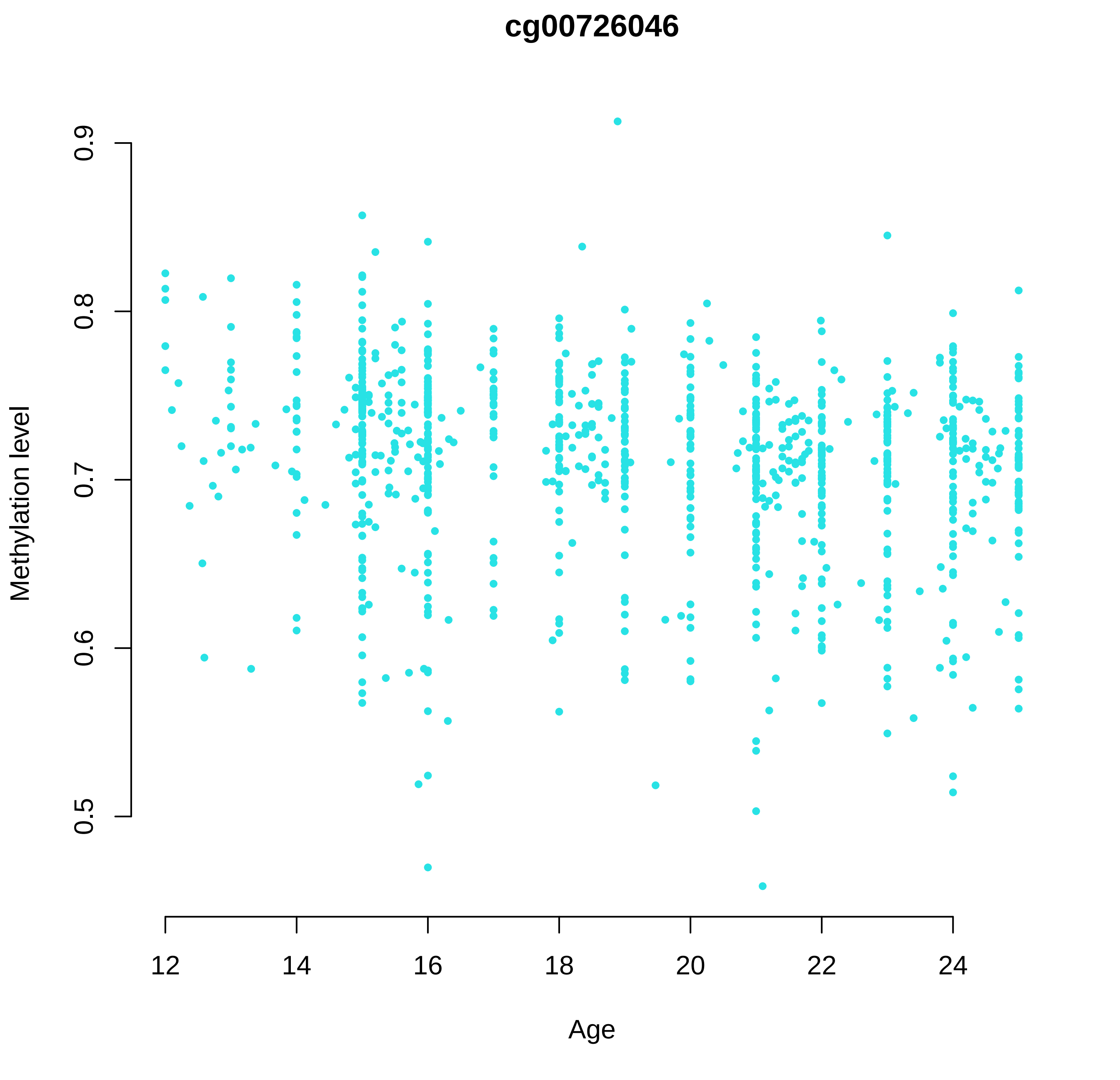

cg00726046
Methylation level
0.5
0.6
0.7
0.8
0.9
12
14
16
18
20
22
24
Age

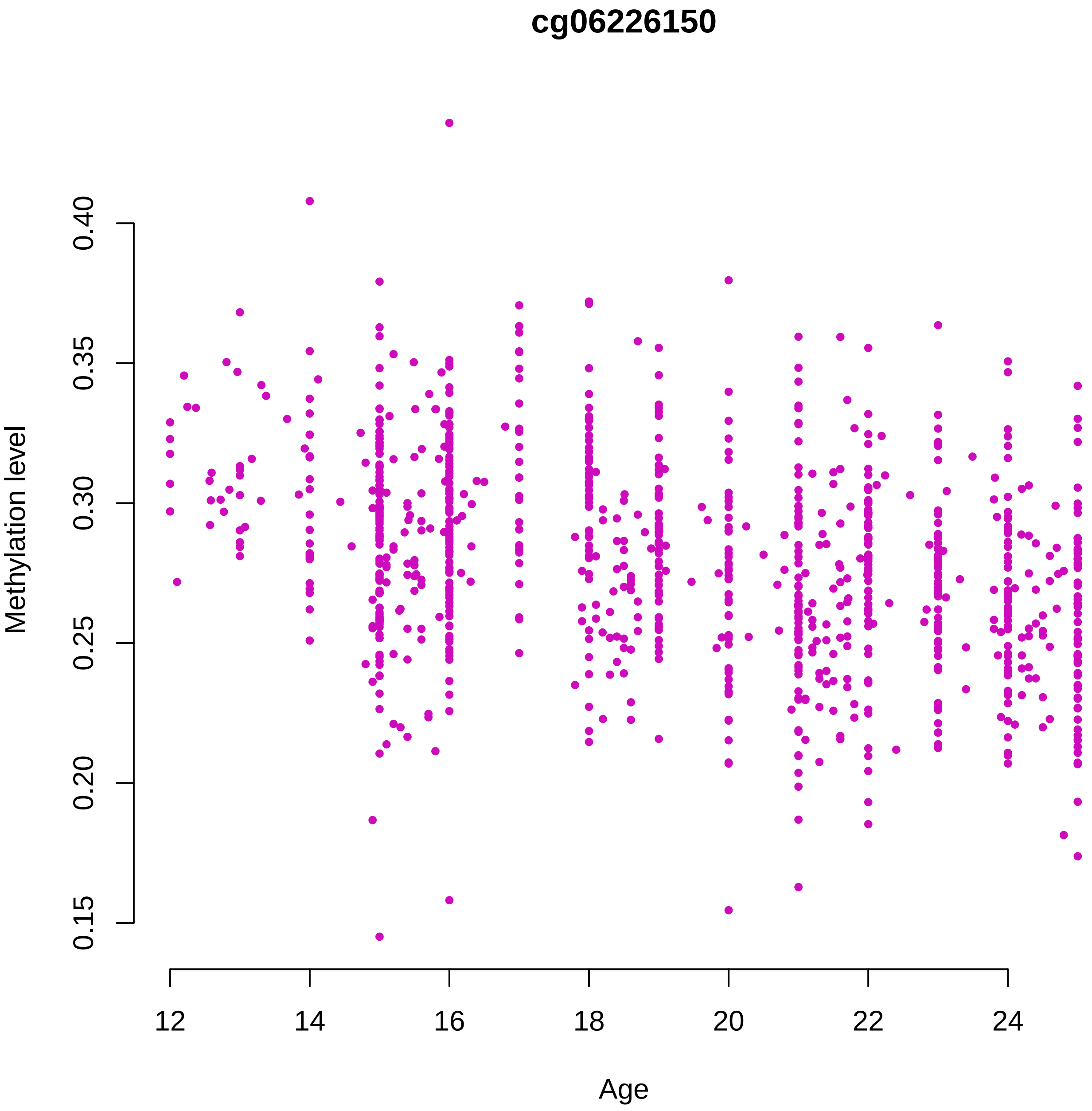
cg06226150
Methylation level
0.15
0.20
0.25
0.30
0.35
0.40
12
14
16
18
20
22
24
Age

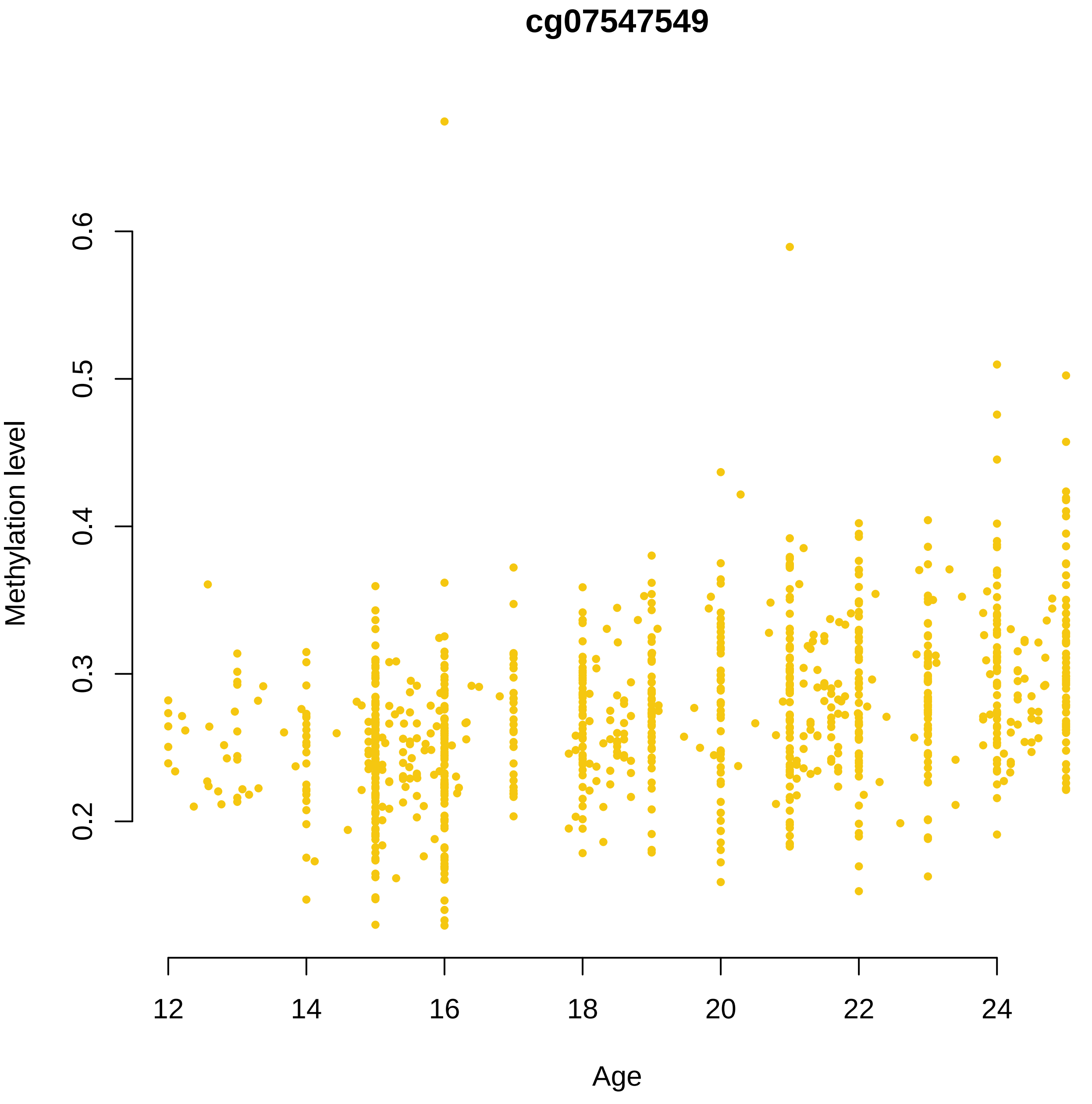
cg07547549
Methylation level
0.2
0.3
0.4
0.5
0.6
12
14
16
18
20
22
24
Age

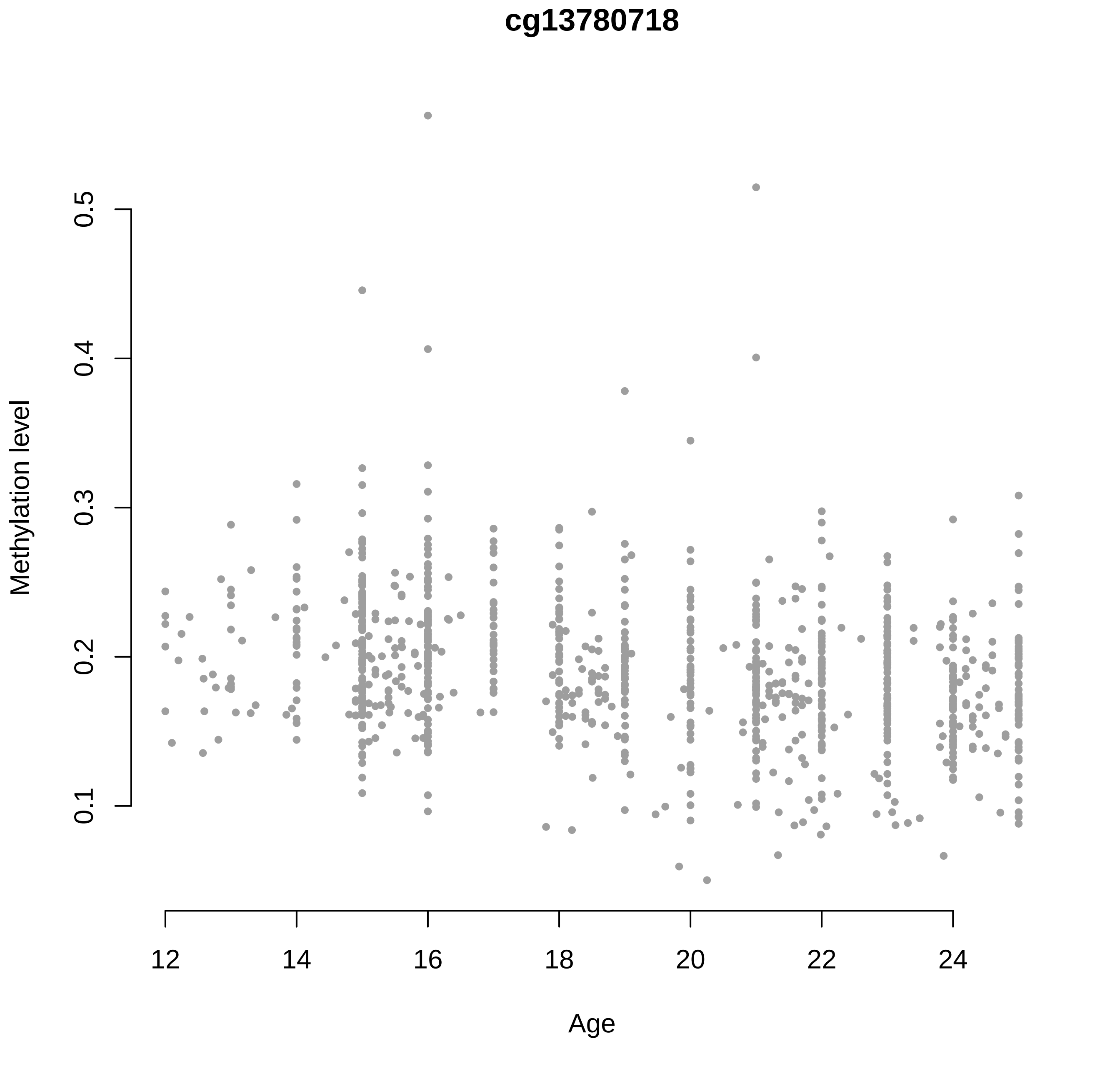
cg13780718
Methylation level
0.1
0.2
0.3
0.4
0.5
12
14
16
18
20
22
24
Age

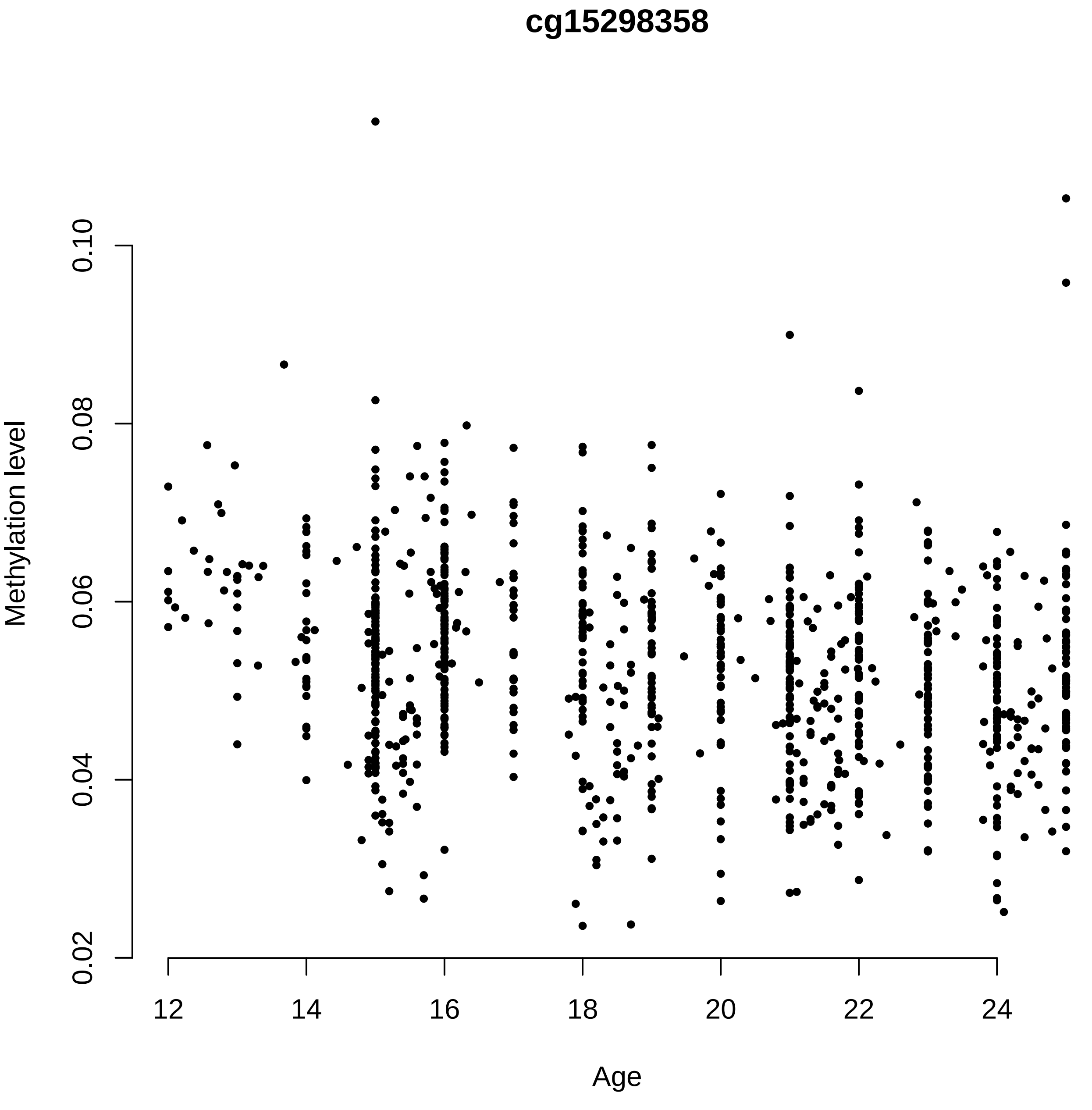
cg15298358
Methylation level
0.02
0.04
0.06
0.08
0.10
12
14
16
18
20
22
24
Age

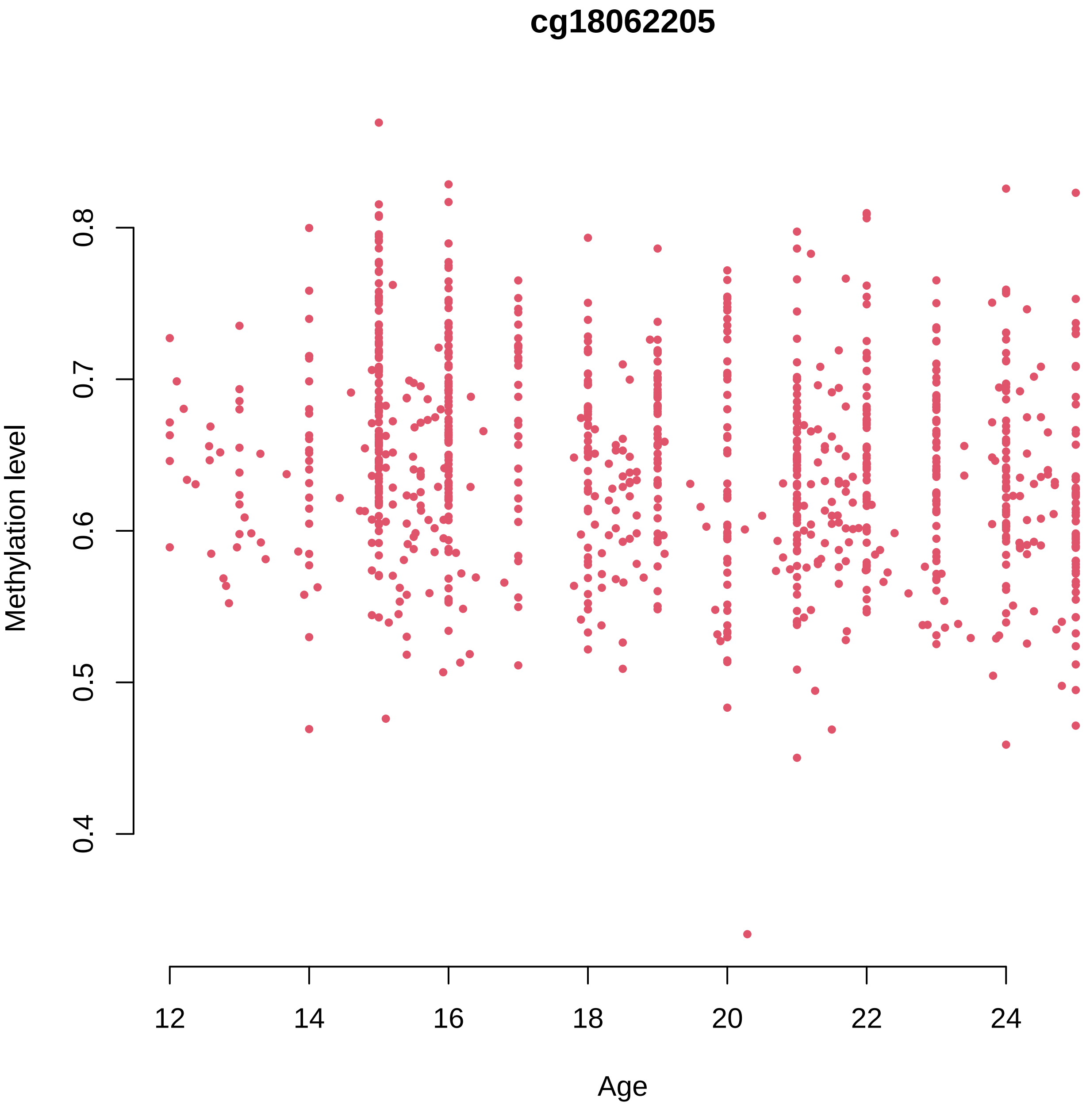
cg18062205
Methylation level
0.4
0.5
0.6
0.7
0.8
Age
12
14
16
18
20
22
24

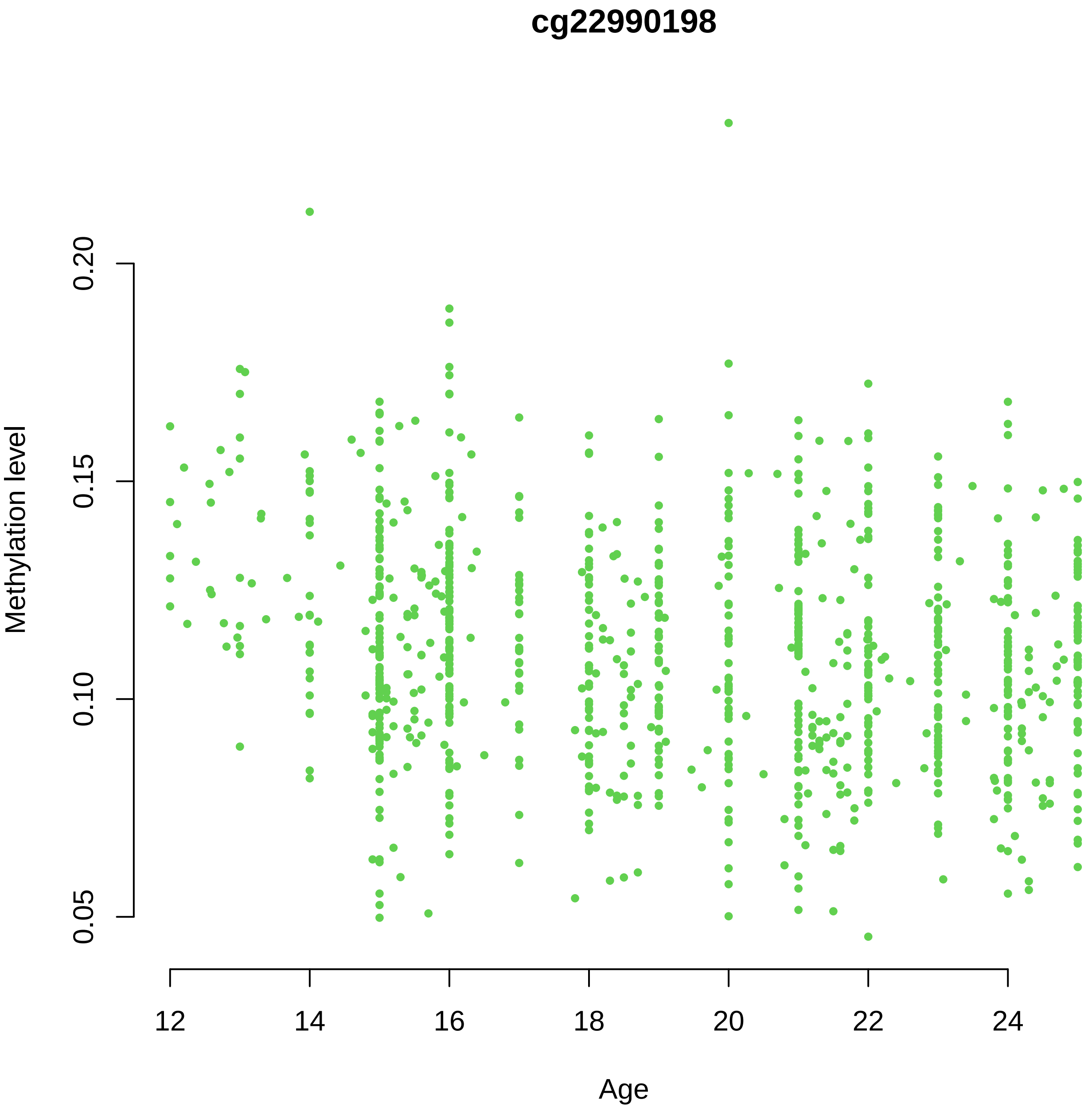
cg22990198
Methylation level
0.05
0.10
0.15
0.20
Age
12
14
16
18
20
22
24

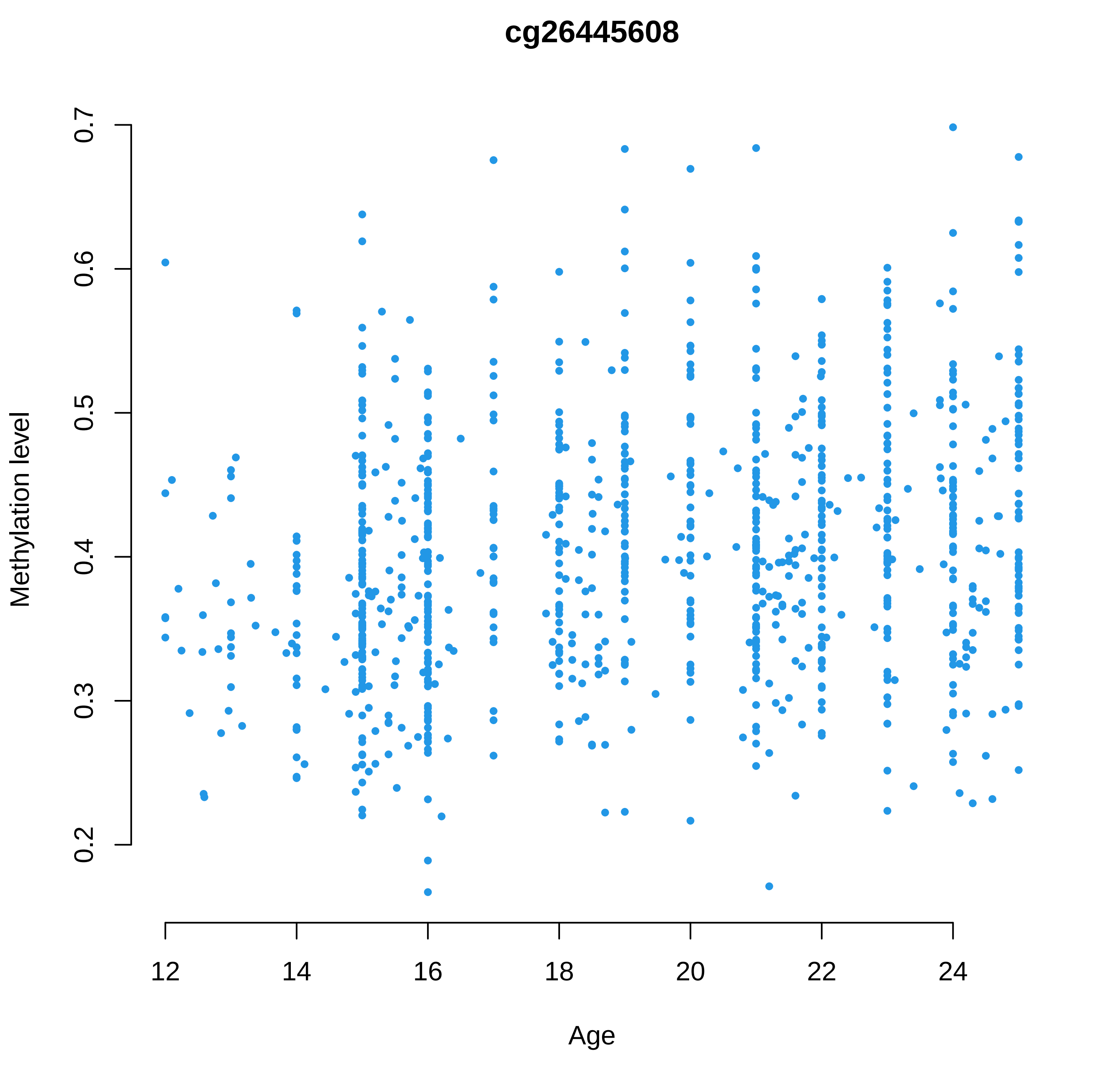
cg26445608
Methylation level
0.2
0.3
0.4
0.5
0.6
0.7
Age
12
14
16
18
20
22
24

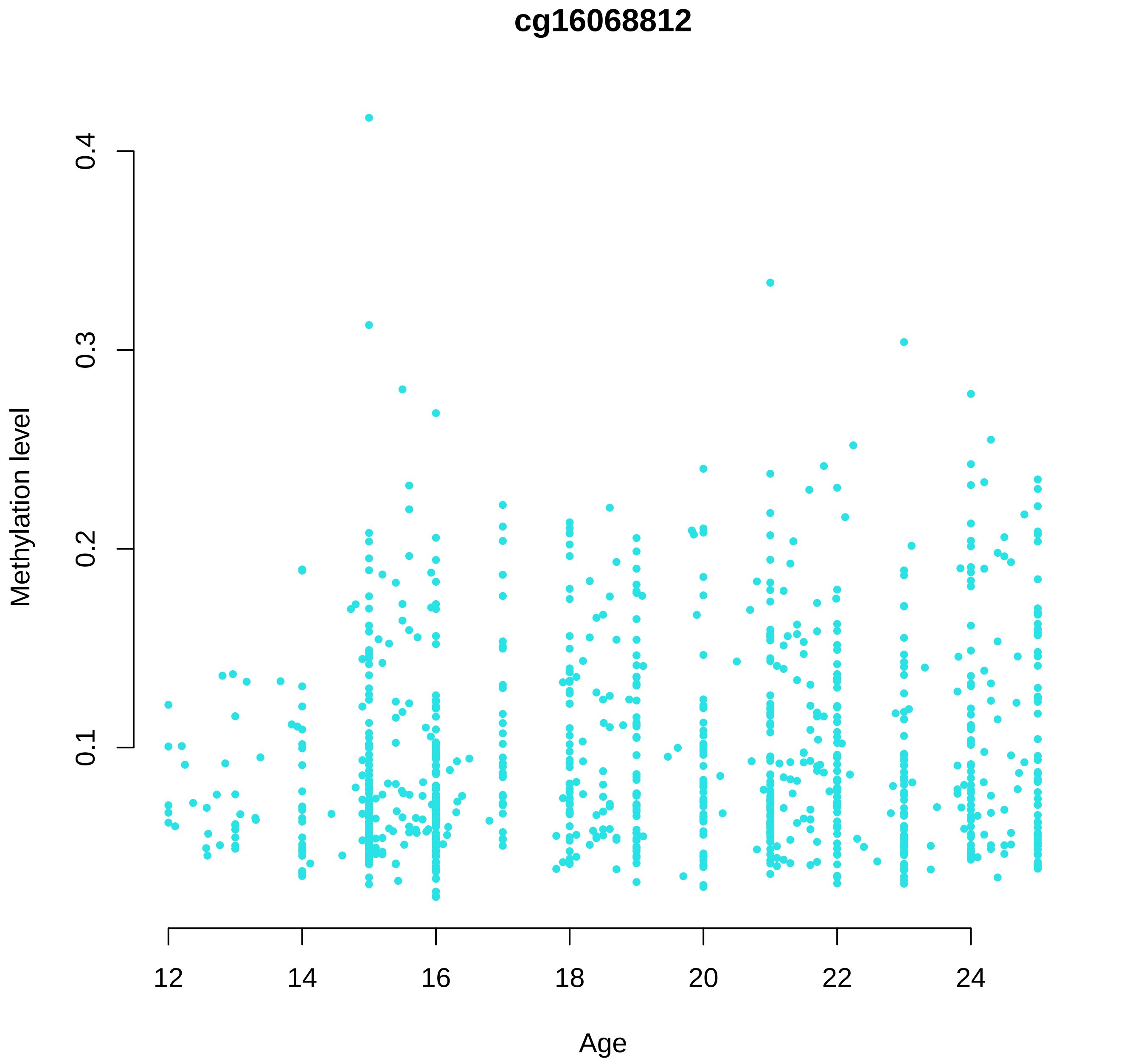
cg16068812
Methylation level
0.1
0.2
0.3
0.4
Age
12
14
16
18
20
22
24

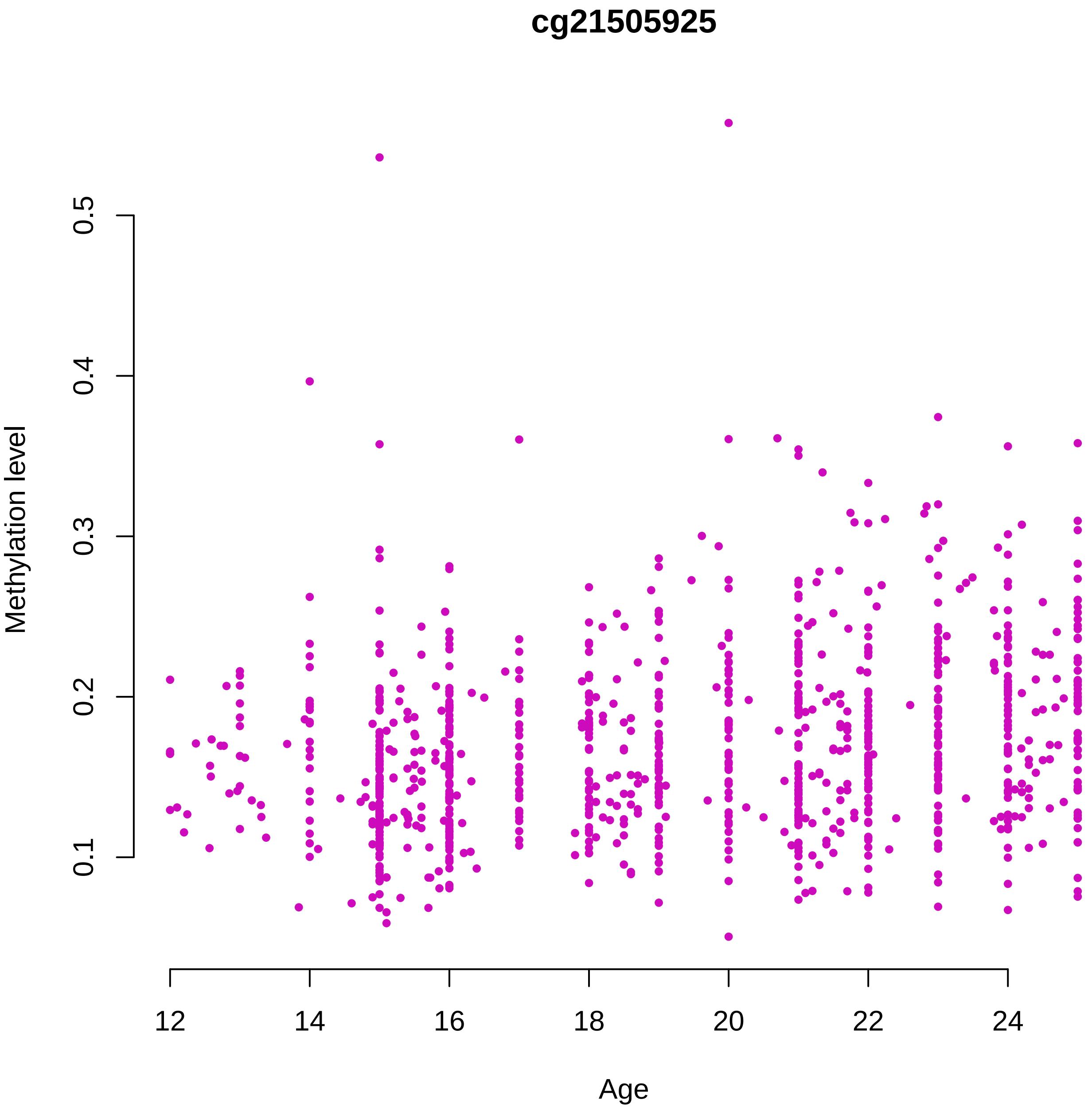
cg21505925
Methylation level
0.1
0.2
0.3
0.4
0.5
Age
12
14
16
18
20
22
24

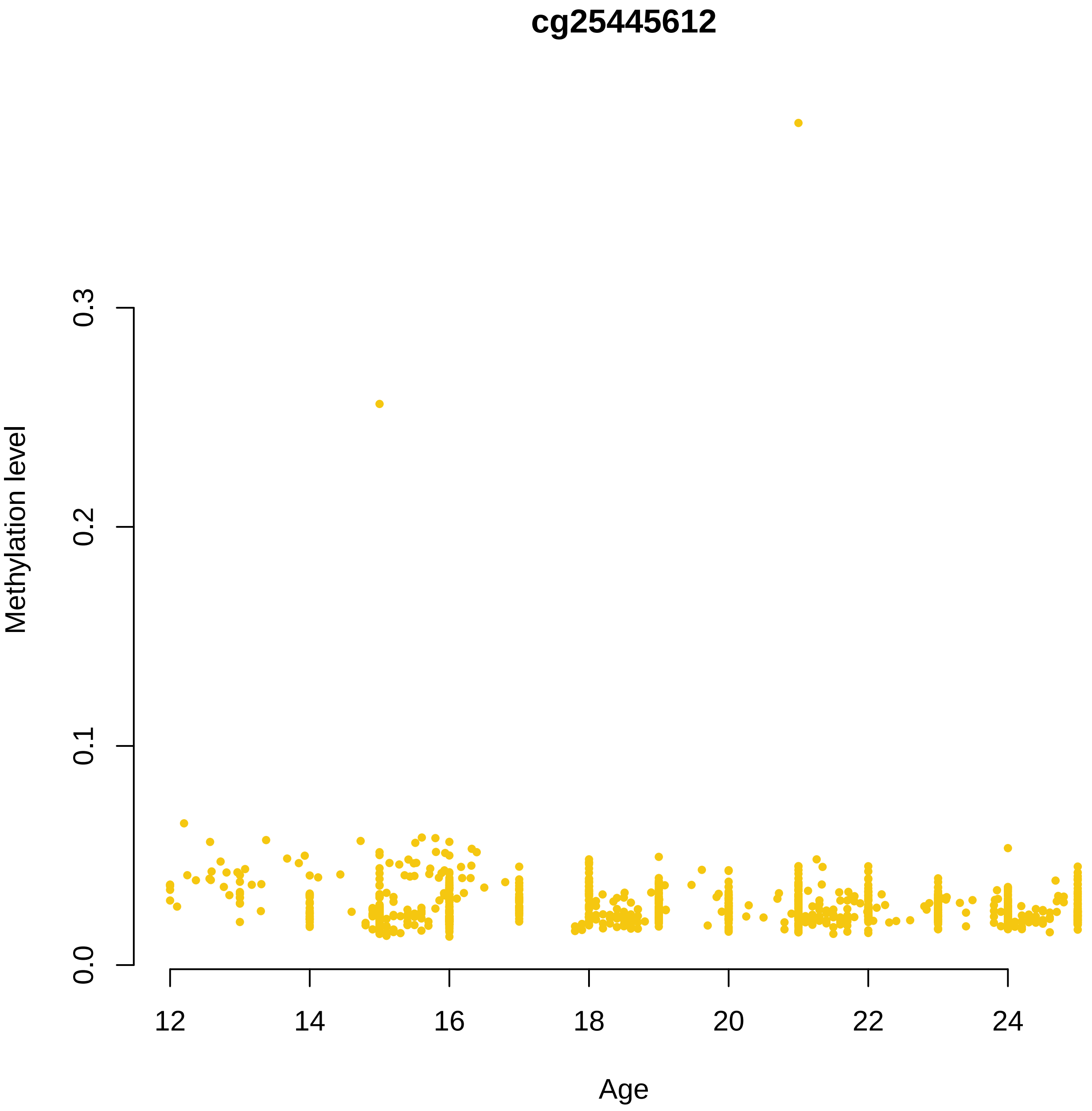

cg25445612
Methylation level
0.0
0.1
0.2
0.3
12
14
16
18
20
22
24
Age

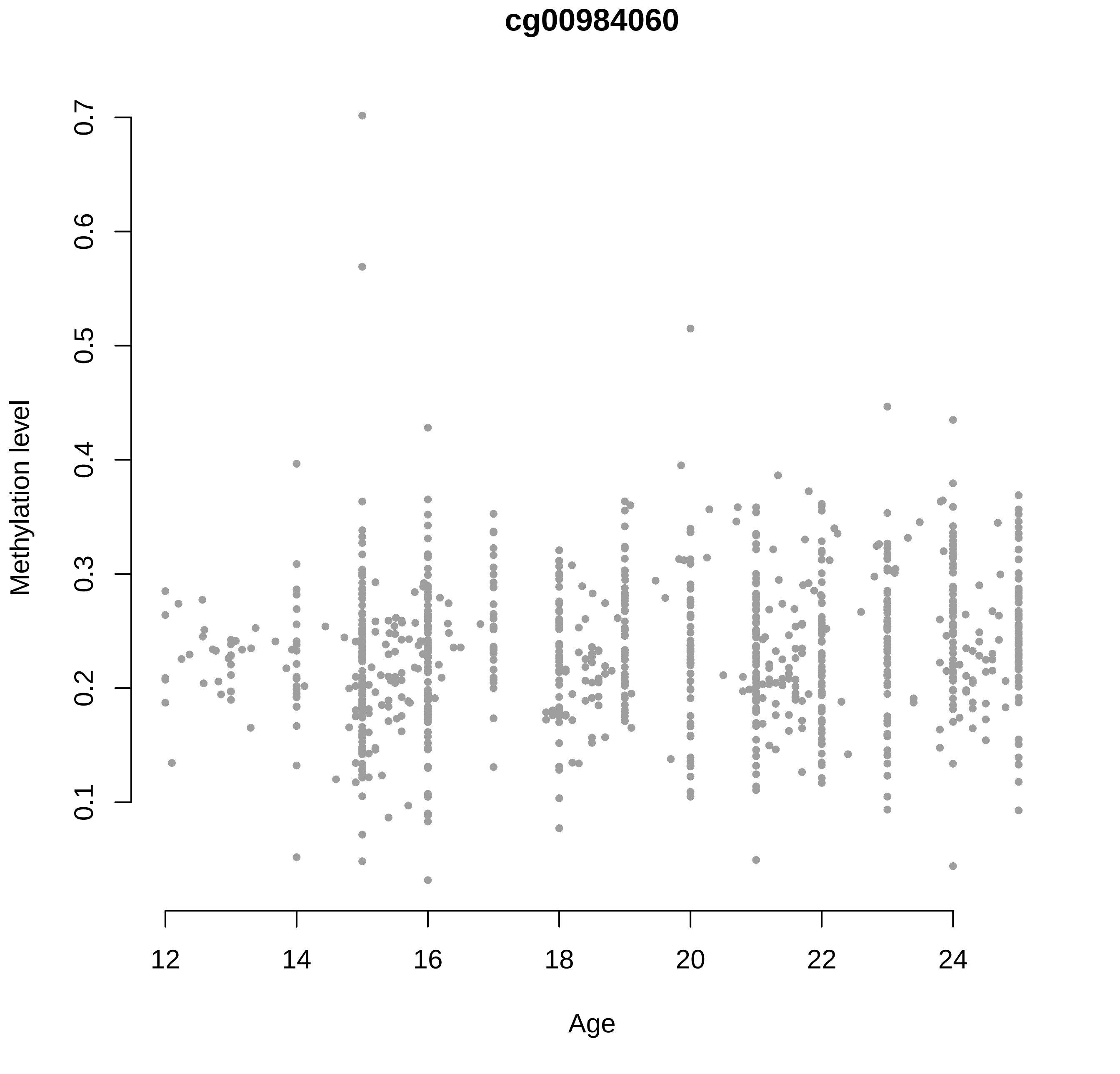
cg00984060
Methylation level
0.1
0.2
0.3
0.4
0.5
0.6
0.7
12
14
16
18
20
22
24
Age

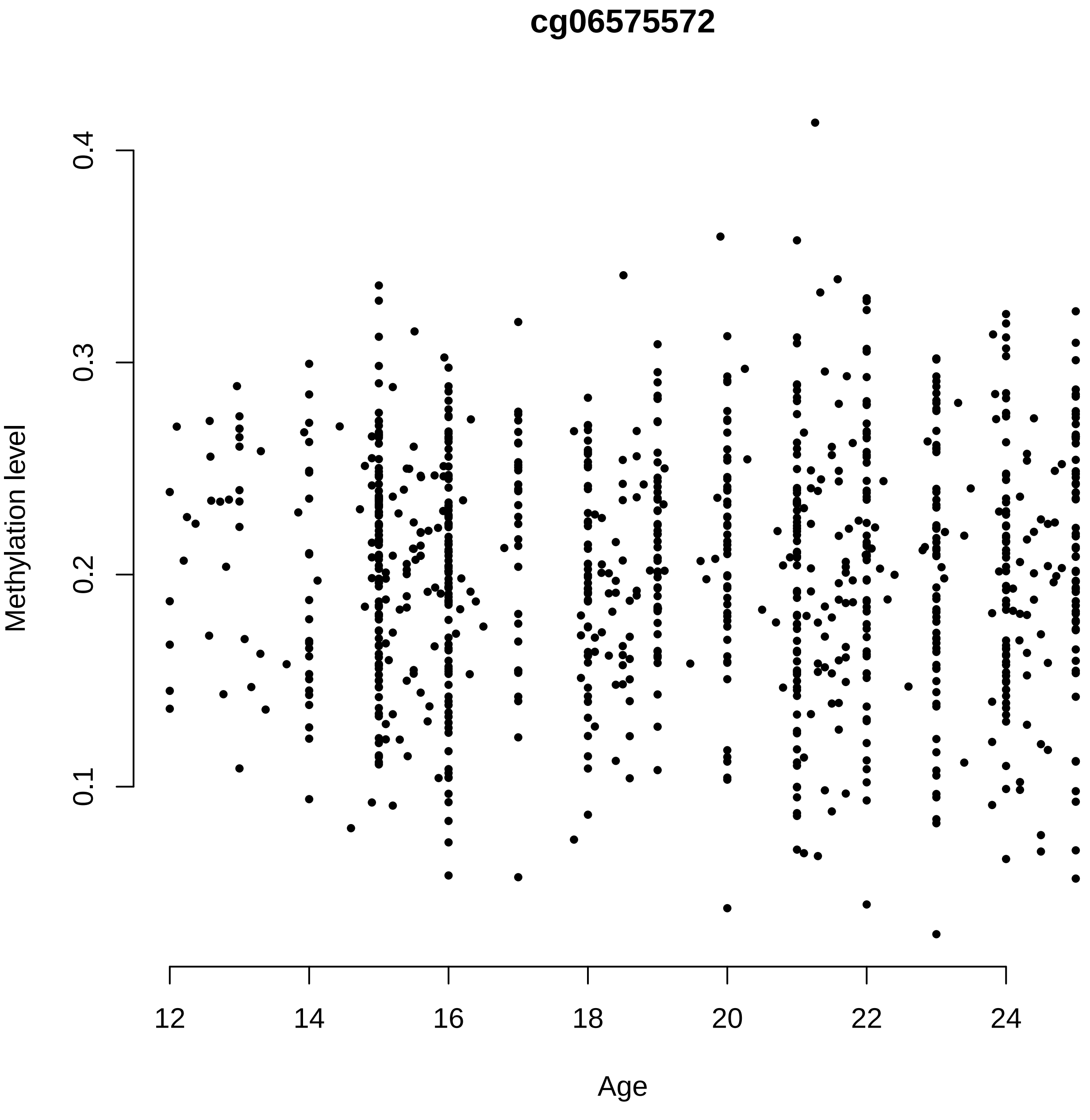

cg06575572
Methylation level
0.1
0.2
0.3
0.4
Age
12
14
16
18
20
22
24

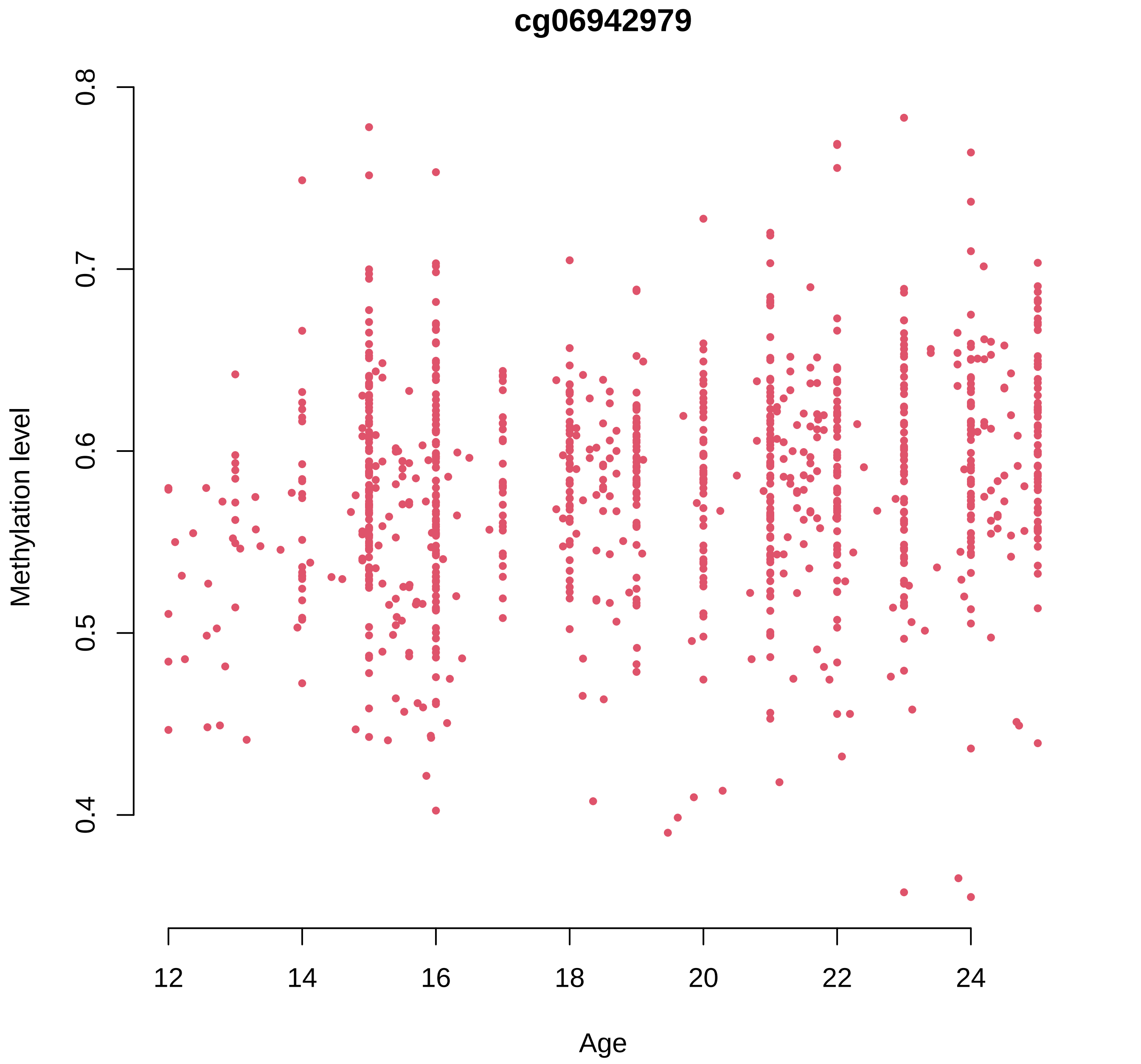

cg06942979
Methylation level
0.4
0.5
0.6
0.7
0.8
Age
12
14
16
18
20
22
24

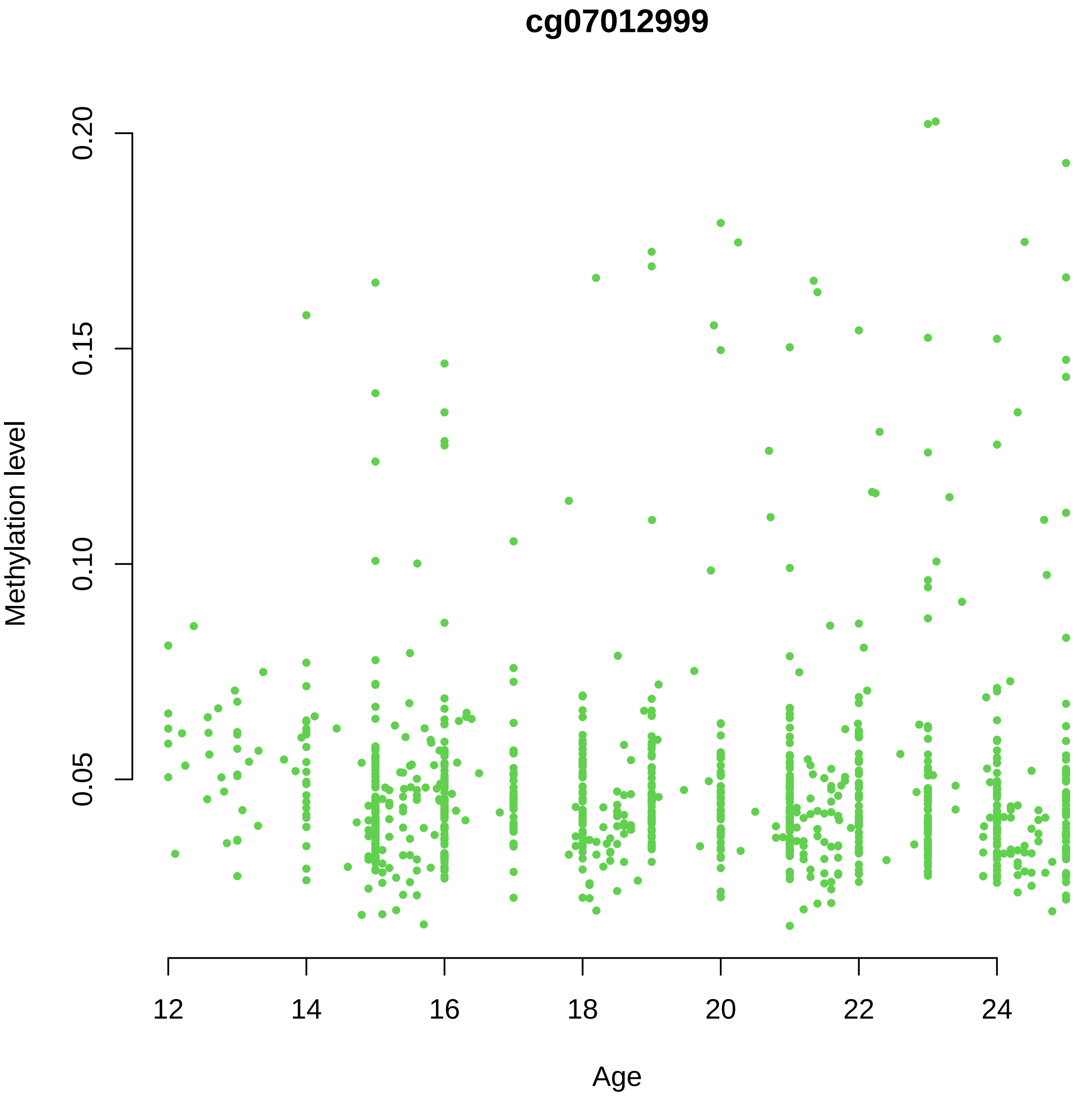
cg07012999
Methylation level
0.05
0.10
0.15
0.20
Age
12
14
16
18
20
22
24
